# Supplementary material for: ADAMTS-7 modulates atherosclerotic plaque formation by degradation of TIMP-1
Source: Circ Res. Author manuscript; Available in PMC 2023 Sep 30. (PMC7615141; doi:10.1161/CIRCRESAHA.123.322737)
Supplement: Supplemental Material [file EMS187870-supplement-Supplemental_Material.pdf]

ADAMTS-7 modulates atherosclerotic plaque formation by degradation of TIMP-1

Running title: *Sharifi et al. – ADAMTS-7 and TIMP-1 in atherosclerosis*

M. Amin Sharifi, MSc[1,2], Michael Wierer, PhD[3], Tan An Dang, MSc[1,2], Jelena Milic, MSc[4], Aldo Moggio, PhD[1], Nadja Sachs, PhD[2,5], Moritz von Scheidt, MD[1,2], Julia Hinterdobler, MSc[1,2], Philipp Müller, MSc[1,2], Julia Werner, DVM[1,2], Barbara Stiller, PhD[1], Zouhair Aherrahrou, PhD[6,7], Jeanette Erdmann, PhD[6,7], Andrea Zaliani[8,9], Mira Graettinger, PhD[8,9], Jeanette Reinshagen[8,9], Sheraz Gul, PhD[8,9], Philip Gribbon, PhD[8,9], Lars Maegdefessel, MD[2,5], Jürgen Bernhagen, PhD[2,4], Hendrik B. Sager, MD[1,2], Matthias Mann, PhD[3], Heribert Schunkert, MD[1,2], Thorsten Kessler, MD[1,2]

1 Department of Cardiology, German Heart Centre Munich, Technical University of Munich, Munich, Germany
2 German Centre for Cardiovascular Research (DZHK e.V.), partner site Munich Heart Alliance, Munich, Germany
3 Department of Proteomics and Signal Transduction, Max-Planck Institute of Biochemistry, Martinsried, Germany
4 Division of Vascular Biology, Institute for Stroke and Dementia Research, Ludwig Maximilian University of Munich, Munich, Germany
5 Vascular Biology and Experimental Vascular Medicine Unit, Department of Vascular and Endovascular Surgery, Klinikum rechts der Isar, Technical University Munich, Munich, Germany
6 Institute for Cardiogenetics and University Heart Centre Lübeck, University of Lübeck, Lübeck, Germany
7 German Centre for Cardiovascular Research (DZHK e.V.), partner site Hamburg/Kiel/Lübeck, Germany
8 Fraunhofer Institute for Translational Medicine and Pharmacology (ITMP), Hamburg, Germany
9 Fraunhofer Cluster of Excellence for Immune-Mediated Diseases (CIMD), Hamburg, Germany

Corresponding author:
Thorsten Kessler, MD
German Heart Centre Munich
Department of Cardiology
Technical University of Munich
Lazarettstr. 36 · 80636 Munich, Germany
Phone: +49 89 1218 4025 · Fax: +49 89 1218 4013
E-mail: thorsten.kessler@tum.de

Supplemental Material

Expanded Methods

Extraction of nucleic acids from mouse aorta, reverse transcription and qPCR

The Maxwell RSC Simply RNA (Promega, Madison, USA) kit was used according to the manufacturer's recommendations. In brief, 20-40 mg of aortic tissue was used. The tissue was snap-frozen in liquid nitrogen and placed in TissueLyser II racks (Qiagen, Hilden, Germany) for mechanical tissue homogenization (30 /s, 1-3 minutes). Then the lyophilizates were centrifuged for 2 min at 13,000 rpm and 200-300 µl of the resulting supernatant was mixed with 200 µl of lysis buffer (Promega). 15-20 µl Protease K was added to the mixture and incubated for 10 min at room temperature and then transferred to the first well of the Maxwell RSC 48 cartridge (Promega). The other wells were prepared as suggested by the user guide, and the SimplyRNA Tissue workflow was used to start the extraction.

An amount of 200 ng of RNA was used in 10 µl reaction volume to digest DNA using the Maxima H Minus cDNA Synthesis Master Mix (ThermoFisher, Waltham, USA) and reverse transcription was performed according to the manufacturer's recommendations. The yielded cDNA was used for quantitative PCR (qPCR) using TaqMan probes (*Gapdh* - Mm99999915, *Adamts7* – Mm01239067, *Timp1* – Mm01341361; all ThermoFisher). *Gapdh* was used as housekeeping gene. Reactions were run on a ViiA 7 Real-Time PCR System (ThermoFisher). Expression levels were reported as $2^{-\Delta Ct}$ values.

Human coronary artery smooth muscle cells

Human aortic smooth muscle cells (hCASMCs; #C-12511; Lot #452Z013.3; positive for smooth muscle α-actin positive, negative for CD31) were purchased from Promocell (Heidelberg, Germany). Passage number four was used for experiments.

Cloning of constructs and transfection of eukaryotic cell lines

Different constructs were cloned with the Gateway cloning system (ThermoFisher Scientific, Waltham, USA). A clone containing the consensus *ADAMTS7* coding sequence (NM_014272 Human Untagged Clone) was purchased from Origene (Rockville, USA). To generate Gateway-compatible constructs, the *ADAMTS7* coding sequence was flanked with attB sites by PCR as recommended by the manufacturer. The primers used are listed in **Supplemental Table S1**. A BP reaction was performed to insert the attB site-flanked PCR product into a pDONR221 (ThermoFisher Scientific) entry clone (pDONR221_ADAMTS7). Secondary to plasmid transformation of competent bacteria (New England Biolabs, Ipswich, USA), selection of clones, and plasmid purification, an LR reaction was performed to insert the sequence of interest into a mammalian expression vector (pDEST40; ThermoFisher Scientific). *TIMP1* (HsCD00039987) and *MMP9* (HsCD00042477) were purchased in

pDONR221 entry vectors from the Harvard Plasmid Repository and were directly utilized in the LR reactions. We used pDEST40 backbones containing different C-terminal tags (V5/His, FLAG, HA) as indicated in **Supplemental Table S2**. After plasmid transformation of competent bacteria, selection of clones, and endotoxin-free plasmid purification, sequencing on both strands was performed. Secondary to sequence verification, plasmids were used to transfect eukaryotic cell lines as described below. Cells were used 24-48 h after transfection for subsequent assays.

To overexpress *ADAMTS7* in vascular smooth muscle cells (VSMCs; details on the cell lines used can be found in the Supplemental Material), we used a lentiviral construct. To that end, we performed an LR reaction with pDONR221_ADAMTS7 and the pLenti6/V5-DEST vector (ThermoFisher Scientific) to generate the pLenti6_ADAMTS7_V5-DEST construct. Lentivirus production was performed according to the manufacturer's recommendations in HEK293 cells using Lipofectamine 3000 (ThermoFisher Scientific). Virus-containing supernatant was collected after 24 and 48 h and combined. After centrifugation at 2,000 rpm, the supernatant was filtered through a 45 µm pore-size filter and stored at -80 °C until further use. Cells were harvested five to six days after transduction.

Co-immunoprecipitation

Co-immunoprecipitation (Co-IP) was performed using the Dynabeads Co-IP Kit (ThermoFisher Scientific) according to the manufacturer's instructions. All precipitations were done with 50 mg of cells and antibody-coupled beads (5 µg IP antibody/mg beads). Negative control IPs were performed using IgG coupled beads (Sigma-Aldrich; 5 µg antibody/mg beads). Approximately 10 µg of IP lysate were used for immunoblotting as described above. The antibodies used for IP are listed in **Supplemental Table S4**.

Immunoblotting

Cells were washed with Dulbecco's phosphate buffered saline (PBS; Biochrom, Berlin, Germany), resuspended in radioimmunoprecipitation assay (RIPA) buffer (Sigma-Aldrich, St. Louis, Missouri, USA) or non-reducing lysis buffer (NRL buffer: 25 mM Tris, 150 mM NaCl, 5 mM $MgCl_2$, 1 mM DTT, 5 % v/v glycerol and 0.5 v/v % NP40), as appropriate. Lysates were separated from cellular debris by centrifugation. Before loading onto gels, samples were mixed 1:1 with 2x Laemmli buffer (Sigma-Aldrich) and boiled for 5 min at 95 °C. 10 µg of cell lysates were loaded on pre-cast gradient gels (4–20 %, Bio-Rad, Hercules, USA). Proteins were transferred to methanol-activated polyvinylidene difluoride (PVDF) membranes (Bio-Rad) at 100 V for 1,5 h. Membranes were blocked for 1 h at room temperature with 5% dry milk in PBS. The primary antibodies used are listed in **Supplemental Table S3**. Blots were incubated with primary antibodies overnight at 4 °C in 2.5 % dry milk and washed with PBS-T (PBS

containing 0.1 % v/v Tween 20, Sigma-Aldrich) before incubation with anti-mouse (#7076S) or anti-rabbit (#7074S) IgG HRP linked antibodies (Cell Signaling Technology, Danvers, USA) at 1:10,000 and 1:100,000 dilutions, respectively, for 1 h at room temperature. After another washing step with PBS-T, bands were visualized by enhanced chemiluminescence (GE Healthcare Life Sciences, Freiburg, Germany). Blots were analyzed using the ImageQuant 800 imaging system (Amersham Biosciences, Amersham, UK) and quantification was performed using ImageJ[15] (RRID: SCR_003070).

Immunohistochemistry and immunofluorescence

For immunohistochemistry, sections were fixed with ice-cold 100 % acetone for 10 min at -20 °C and washed with PBS-T (0.1 % v/v Tween 20, Sigma-Aldrich) before blocking with 5 % BSA in PBS (m/v) for 1h at room temperature. After washing, sections were further blocked with Dako REAL Peroxidase-Blocking solution (Agilent, Santa Clara, USA) for 20 min. Next, sections were incubated overnight with primary antibodies at 4 °C. Rabbit IgG served as isotype control for both ADAMTS-7 and TIMP-1 antibodies (**Suppl. Table S3**). After washing, biotinylated IgG (1:1000 in 5 % BSA) was added and incubated for 1h at room temperature. 50 % glycerol was then added to each specimen and covered with coverslips.

For immunofluorescence, aortic root sections were rinsed with PBS and fixed in 4 % v/v fresh paraformaldehyde (PFA) at room temperature for 10 min. Next, sections were permeabilized by incubation in PBS containing 0.5 % v/v Triton (x100) for 10 min at room temperature. After washing with PBS, blocking was performed for 1 h at room temperature using PBS containing 5 % m/v BSA and 0.1 % v/v Triton. Sections were incubated with 1:300 dilutions of rabbit anti-Adamts-7 (#201083; Abcam, Cambridge, UK) and goat anti-Timp-1 (#cnl0320021; R&D Systems, Minneapolis, USA) as primary antibodies overnight at 4 °C. Rabbit IgG (#ab171870; Abcam) and goat IgG (#02-6202, ThermoFisher Scientific) were utilized as isotype controls. Secondary anti-rabbit (#ab150073) and anti-goat (#ab175664; both Abcam) antibodies were added after washing with PBS. Finally, sections were mounted with DAPI mounting solution (Carl Roth, Karlsruhe, Germany).

Images were taken with a THUNDER imaging system (Leica, Wetzlar, Germany) or an Axioscan 7 slide scanner (Zeiss, Jena, Germany).

Matrix metalloproteinase activity assays

Fluorescein conjugate gelatine assay - To measure the activity of collagenase and gelatinase, the supernatant of human coronary artery smooth muscle cells overexpressing *ADAMTS7* or a mock plasmid was used with fluorescein conjugates of gelatine (DQ Gelatine from Pig Skin, ThermoFisher). Supernatants were plated on a flat black 96-well plate (Nunclon, ThermoFisher). 200 µl of serum free supernatant were incubated with 0.1 mg/ml of the

substrate overnight at 37 °C and fluorescence intensity was measured using an Infinite M200 PRO microplate reader (Tecan, Maennedorf, Switzerland) according to the manufacturer's recommendations. Background fluorescence was determined and subtracted from each value.

Gel zymography – Supernatant of human coronary artery smooth muscle cells overexpressing *ADAMTS7* or a mock plasmid was used. 1 ml of serum-free supernatant was concentrated using 30 µl Gelatine-agarose beads (Sigma-Aldrich) for 16 h at 4 °C. Beads were then washed with PBS and 20 µl sample buffer. Samples were loaded on pre-cast Novex 10% Zymogram Plus Protein Gels (ThermoFisher, Waltham, USA). Electrophoresis was performed at 125 V for 2 h. Afterwards, gels were incubated with renaturing buffer for 30 min, rinsed with water and subjected to developing buffer for 30 min. The developing buffer was then exchanged and further incubation was performed for 48 h. After incubation, the gel was stained with staining solution for 1 h and destained using destaining solution until areas of gelatinolytic activity appeared as sharp bands on a blue background. Gels were scanned for quantification using an ImageQuant 800 imaging system (Amersham Biosciences).

Colorimetric assay – Supernatant of HEK293 cells overexpressing *ADAMTS7* (or mock plasmid) and *TIMP1* were collected and subjected to concentration using 3K Amicon Ultra-15 filters (Sigma-Aldrich). Recombinant MMP-9 from the Matrix Metalloproteinase-9 Colorimetric Drug Discovery Kit (Enzo Life Sciences, New York, USA) was added to the supernatants. Assays were performed according to the manufacturer's recommendations. Absorbance was measured using an Infinite M200 PRO microplate reader (Tecan).

TIMP-1 degradation assays

In vitro degradation – HEK293 cells were transiently transfected with *TIMP1* and *ADAMTS7* constructs. After 48 h, supernatants were collected and concentrated with 3K Amicon Ultra-15 filters (Sigma-Aldrich). Cell lysates were prepared as described above. Same amounts of protein of each sample were loaded and the differences in TIMP-1 levels were analyzed using immunoblotting as described above. Quantification was done using ImageJ as described above.

Flow cytometry – HEK293 cells were transiently transfected with *TIMP1-GFP* and *ADAMTS7* constructs or a mock plasmid. After 48 h, cells were washed with PBS and harvested in PBS containing 0.5 % m/v BSA. Cells were filtered through 5 ml Falcon round-bottom polystyrene test tubes with cell strainer snap caps (Falcon, Vancouver, Canada) and DAPI (1:500) was added as viability dye. The numbers and intensity of GFP-positive cells were measured by flow cytometry using a LSRFortessa (BD Biosciences, Franklin Lakes, USA) flow cytometer. The geometric mean fluorescence intensity (geo MFI) index method was used for quantification.

Fluorescence microscopy – HEK293 cells were transiently transfected with *TIMP1-GFP* and *ADAMTS7* constructs. After 48 h, samples were washed with PBS and fixed in 4% v/v PFA for 10 min at room temperature. Permeabilization was induced with 0.5 % v/v Triton in PBS for 10 min at room temperature. Slides were mounted with ROTI Mount FluorCare DAPI (Carl Roth). Images were taken with 10x magnification using a Stellaris 5 confocal microscope (Leica, Wetzlar, Germany). Analysis was performed by measuring the GFP intensity based on the cell count. To obtain cell counts we used a custom macro in ImageJ. GFP intensity divided by cell count was used to quantify TIMP-1-GFP protein levels for each image.

Picrosirius red staining

Aortic root sections in OCT from Apoe-/-Adamts7-/- and Apoe-/- mice were brought to room temperature and rinsed with PBS. Nuclei were stained with Weigert's Hematoxylin Kit (Polysciences, Warrington, USA) for 8 min and washed for 10 min under running tap water. Sections were stained in Direct Red 80 stain (Sigma-Aldrich) for 1 h. Specimens were washed in two preparations of acidified water (1.3 % saturated Picric acid). Slides were dehydrated with three preparations of 100 % ethanol, cleared in xylene, and mounted with resinous medium. Images were taken under bright field and polarisation imaging. Quantification of bright field images was performed using a collagen imaging macro (https://imagej.nih.gov/ij/docs/examples/stained-sections/index.html). Polarisation images were analyzed using an in-house developed macro.

Homogeneous time-resolved fluorescence resonance energy transfer (FRET)

HEK293 cells were co-transfected with *ADAMTS7-FLAG* and *TIMP1-HA* constructs. Single transfections of constructs were performed as negative controls. After 24 h, cell lysates were prepared using a non-reducing lysis buffer and final protein concentrations of 1 µg/µl were used. Fluorophore-conjugated antibodies against HA (anti-HA-d2 mAb, #610HADAA) and FLAG (anti-FLAG-M2-Tb cryptate mAb, #61FG2TLA; both Cisbio, Codolet, France) were used. The FRET signal, i.e., the ratio of emission at 665/620 nm wavelength, was measured after 24 h of incubation with an Infinite F200 PRO filter-based microplate reader (Tecan).

Supplemental Tables

Supplemental Table S1: Gateway oligonucleotide primers which were used for cloning of different ADAMTS-7 constructs.

Name	Sequence (3'-5')	Purpose
ADAMTS7_for	GGG GACA AGT TTG TAC AAA AAA GCA GGC TTC GAA GGA GAT AGA ACC ATG CCC GGC GGC CCC AG	Cloning of the full-length *ADAMTS7* ORF into the pDONR221 entry vector
ADAMTS7_rev	GGG GAC CAC TTT GTA CAA GAA AGC TGG GTC GCG GCG GGC AAC CCG CTG	
$_{\Delta TSPr}$ADAMTS7_for	GGG GAC AAG TTT GTA CAA AAA AGC AGG CTT CGA AGG AGA TAG AAC CAT GAA GTG GGT GGA GAC CCT GG	Cloning of the N-terminal ADAMTS-7 part containing the catalytic domain but lacking the C-terminal disintegrin domain and thrombospondin repeats into the pDONR221 entry vector
$_{\Delta TSPr}$ADAMTS7_rev	GGG GAC CAC TTT GTA CAA GAA AGC TGG GTC AGG AGG GTC GTC CAG GCA C	
$_{\Delta cat}$ADAMTS7_for	GGG GAC AAG TTT GTA CAA AAA AGC AGG CTT CGA AGG AGA TAG AAC CAT GCA CAC CGT GAG CGG GAC CTT CG	Cloning of the C-terminal ADAMTS-7 part containing the disintegrin domain and thrombospondin repeats but lacking the catalytic domain into the pDONR221 entry vector
$_{\Delta cat}$ADAMTS7_rev	GGG GAC CAC TTT GTA CAA GAA AGC TGG GTC GCG GCG GGC AAC CCG CTG	

Supplemental Table S2: Gateway expression vectors and resulting constructs which were used for experiments. * The pcDNA-DEST40 vector was purchased from Thermo Fisher Scientific, Waltham, USA. [#] The pcDNA-DEST40-FLAG vector was cloned in-house via inserting a FLAG tag followed by a stop codon before the V5 tag using the pcDNA-DEST40 vector and *in vitro* mutagenesis. [†] The pcDNA-DEST40-HA vector was cloned in-house via inserting an HA tag followed by a stop codon before the V5 tag using the pcDNA-DEST40 vector and *in vitro* mutagenesis. [§] The pDEST-CMV-3xFLAG-gateway-EGFP vector was a gift from Robin Ketteler (Addgene plasmid # 122845 ; http://n2t.net/addgene:12284; RRID:Addgene_122845).

Vector (Tag)	Constructs	Purpose
pcDNA-DEST40 (C-terminal V5+6xHis)*	pDEST40_ADAMTS7-V5	full length ADAMTS-7 with C-terminal V5 tag; used for Co-IP with TIMP-1 (Figure 2); used for Co-IP with TIMP-1 and MMP9 (Figure 4)
	pDEST40_$_{\Delta TSPr}$ADAMTS7-V5	N-terminal ADAMTS-7 part containing the catalytic domain but lacking the C-terminal disintegrin domain and thrombospondin repeats with C-terminal V5 tag; used for Co-IP with TIMP-1 (Figure 2)
	pDEST40_$_{\Delta cat}$ADAMTS7-V5	C-terminal ADAMTS-7 part containing the disintegrin domain and thrombospondin repeats but lacking the catalytic domain with C-terminal V5 tag; used for Co-IP with TIMP-1 (Figure 2); used for Co-IP with TIMP-1 and MMP9 (Figure 4)
pcDNA-DEST40-FLAG (C-terminal FLAG)[#]	pDEST40_ADAMTS7-FLAG	full length ADAMTS-7 with C-terminal FLAG tag; used for FRET experiments with TIMP-1 (Figure 5)
	pDEST40_$_{\Delta cat}$ADAMTS7-FLAG	C-terminal ADAMTS-7 part containing the disintegrin domain and thrombospondin repeats but lacking the catalytic domain with C-terminal FLAG tag; used for FRET experiments with TIMP-1 (Figure 5)

	pDEST40_MMP9-FLAG	full length MMP9 with C-terminal FLAG tag; used for Co-IP with ADAMTS-7 and TIMP-1 (Figure 4)
pcDNA-DEST40-HA (C-terminal HA)†	pDEST40_TIMP1-HA	full length TIMP-1 with C-terminal HA tag; used for Co-IP with ADAMTS-7 (Figure 2); used for *in vitro* degradation experiments (Figure 3); used for Co-IP with ADAMTS-7 and MMP9 (Figure 4); used for FRET experiments with ADAMTS-7 (Figure 5)
pDEST-CMV-C-EGFP§ (N-terminal 3xFLAG, C-terminal EGFP)	pDEST-TIMP1-EGFP	full length TIMP-1 with C-terminal EGFP; used for *in vitro* degradation experiments (Figure 3)

Supplemental Table S3: Primary antibodies used for immunoblotting, immunohistochemistry and immunofluorescence.

Antibody	Host	Manufacturer	Dilution
For immunoblotting			
Anti-V5 (monoclonal)	mouse	Thermo Fisher Scientific, Waltham, USA (#R960-25; RRID: AB_2556564)	1:5000
Anti-V5 (monoclonal)	rabbit	Cell Signaling Technology, Danvers, USA (#D3H8Q; RRID:AB_2687461)	1:10000
Anti-HA (monoclonal)	mouse	Thermo Fisher Scientific, Waltham, USA (#MA5-27915; RRID: AB_2744968)	1:10000
Anti-HA (monoclonal)	rabbit	Cell Signaling Technology, Danvers, USA (#C29F4; AB_1549585)	1:5000
Anti-FLAG (monoclonal)	mouse	Sigma Aldrich, St. Louis, USA (#F1804; RRID: AB_262044)	1:5000
Anti-FLAG (monoclonal)	rabbit	Sigma Aldrich, St. Louis, USA (#F2555; RRID: AB_796202)	1:500
Anti-ADAMTS7 (polyclonal)	mouse	Abnova, Taipeh, Taiwan (#H00011173A01; RRID: N/A)	1:1000
Anti-TIMP1 (monoclonal)	rabbit	Thermo Fisher Scientific, Waltham, USA (#MA5-13688; RRID: AB_11004132)	1:20000
Anti-GAPDH (monoclonal)	rabbit	Cell Signaling Technology, Danvers, USA (#2118; RRID: AB_561053)	1:20000
Anti-Vinculin (monoclonal)	rabbit	Thermo Fisher Scientific, Waltham, USA (#VLN01; RRID: AB_10976821)	1 µg/ml
Anti-MMP9	mouse	Merckmilipore, Massachusetts, USA (#MAB13415; RRID: AB_94130)	1:500
For immunohistochemistry			
Anti-TIMP1	rabbit	Biorbyt, Cambridge,United Kingdom (#orb195994; RRID: N/A)	1:350
Rabbit IgG – Isotype Control (polyclonal)	rabbit	Abcam, Cambridge,United Kingdom (#ab171870; RRID: AB_2687657)	1:350
goat anti-rabbit -IgG biotinylated	goat	Biozol, Eching,Germany(#VEC-BA-1000; RRID: N/A)	1:1000
For immunofluorescence			
Anti-ADAMTS7 (polyclonal)	rabbit	Abcam, Cambridge,United Kingdom (#ab201083; RRID: N/A)	1:300
Anti-TIMP1 (polyclonal)	goat	R&D Systems, Minneapolis, USA (#AF980; RRID: AB_355759)	7:100
Rabbit anti-Goat IgG (Alexa Fluor 594)	rabbit	Thermo Fisher Scientific, Waltham, USA (#A11080; RRID: AB_2534124)	1:1000
Donkey anti-Rabbit IgG	donkey	Abcam, Cambridge,United Kingdom	1:1000

(Alexa Fluor 488)		(ab150073; RRID: AB_2636877)	
Donkey anti-Goat (Alexa Fluor 594)	donkey	Abcam, Cambridge,United Kingdom (ab150132; RRID: AB_2810222)	1:1000

Supplemental Table S4: Antibodies used for immunoprecipitation.

**Antibody**	**Host**	**Manufacturer**	**Dilution**
IgG control	mouse	Thermo Fisher Scientific, Waltham, USA (#31903; RRID: AB_10959891)	5 µg/mg beads
Anti-HA	mouse	Thermo Fisher Scientific, Waltham, USA (#MA5-27915; RRID: AB_2744968)	5 µg/mg beads
Anti-FLAG	mouse	Sigma Aldrich, St. Louis, USA (#F1804; RRID: AB_262044)	5 µg/mg beads

Supplemental Table S5: Distribution of data and statistical test used. * Kolmogorov-Smirnov test. # Shapiro-Wilk test. For small sample sizes (n<10), it was assumed that the central limit theorem applies.

**Figure, panel**	**Comparison**	**Normal distribution (p-values)**	**Test used**
1, A	Aorta proteome, Apoe-/- (n=3) vs. Apoe-/-Adamts7-/- (n=4)	Not applicable	Unpaired t-tests, permutation-based FDR correction for multiple hypothesis testing
1, B	Comp, Apoe-/- (n=3) vs. Apoe-/-Adamts7-/- (n=4)		
	Thbs1, Apoe-/- (n=3) vs. Apoe-/-Adamts7-/- (n=4)		
1, C	Timp1, Apoe-/- (n=3) vs. Apoe-/-Adamts7-/- (n=4)		
	Timp2, Apoe-/- (n=3) vs. Apoe-/-Adamts7-/- (n=4)		
3, B	*In vitro* TIMP-1 degradation, full-length ADAMTS-7 vs. mock, full-length ADAMTS-7 vs. $_{\Delta cat}$ADAMTS7; n=4 independent experiments	Yes (>0.28)#	Ordinary one-way ANOVA, Sidak's multiple comparisons test
3, D	TIMP-1-GFP fluorescence after co-expression with ADAMTS-7 in HEK293 cells (immunofluorescence); full-length ADAMTS-7 vs. mock, full-length ADAMTS-7 vs. $_{\Delta cat}$ADAMTS7; n=5 independent experiments	Yes (>0.10)*	Ordinary one-way ANOVA, Sidak's multiple comparisons test
3, F	TIMP-1-GFP fluorescence after co-expression with ADAMTS-7 in HEK293 cells (flow cytometry); full-length ADAMTS-7 vs. mock, full-length ADAMTS-7 vs. $_{\Delta cat}$ADAMTS7; n=6 independent experiments	Yes (>0.10)*	Ordinary one-way ANOVA, Sidak's multiple comparisons test
4, A	Endogenous MMP2/MMP9 activity in vascular smooth muscle cells, mock (n=10 independent experiments) vs. overexpression of full-length ADAMTS-7 (n=9 independent experiments)	Yes (>0.10)*	Student's t-test
4, C	Endogenous MMP9 activity after overexpression of TIMP-1, mock vs. full-length ADAMTS-7; n=8 independent experiments	No (0.01)*	Mann-Whitney test
4, D	Endogenous MMP2 activity after overexpression of TIMP-1, mock	Yes (>0.10)*	Student's t-test

	vs. full-length ADAMTS-7; n=8 independent experiments		
4, E	Recombinant MMP9 activity after overexpression of TIMP-1, mock vs. full-length ADAMTS-7; n=5 independent experiments	Yes (>0.10)*	Student’s t-test
4, G	TIMP-1 binding to MMP9, full-length ADAMTS-7 vs. $_{\Delta cat}$ADAMTS7; n=3 independent experiments	Yes (>0.75)#	Student’s t-test
4, I	Plaque collagen content, Apoe-/- (n=14) vs. Apoe-/-Adamts7-/- (n=9)	Yes (>0.05)*	Student’s t-test
5	*ADAMTS7* expression in fibrous caps of stable (n=10) and unstable human carotid artery plaques (n=10)	No (0.02)*	Mann-Whitney test
6, A	FRET signal, ADAMTS-7 and TIMP-1 (n=6 independent experiments) vs. mock and TIMP-1 (n=6 independent experiments), ADAMTS-7 and TIMP-1 vs. ADAMTS-7 (n=6 independent experiments) and mock (n=4 independent experiments)	Yes (>0.15)#	Ordinary one-way ANOVA, Sidak’s multiple comparisons test
6, B	FRET signal, ADAMTS-7 and TIMP-1, 0 vs. 400 vs. 800 ng untagged TIMP-1; n=7 independent experiments	Yes (>0.10)*	Ordinary one-way ANOVA, p for trend
S1	Numbers of detected proteins, Apoe-/- (n=3) vs. Apoe-/-Adamts7-/- (n=4)	Yes (>0.24)#	Student’s t-test
S3	Aorta *Adamts7* expression, Adamts7+/+ (n=5) vs. Adamts7-/- (n=6)	No (0.04)*	Mann-Whitney test
	Aorta *Timp1* expression, Adamts7+/+ (n=5) vs. Adamts7-/- (n=6)	No (0.04)#	Mann-Whitney test
S5	rMMP activity, mock vs. full-length ADAMTS-7 (n=3)	Yes (>0.10)#	Student’s t-test
S6	TIMP-1 fluorescence, Apoe-/- (n=7) vs. Apoe-/-Adamts7-/- (n=6)	Yes (>0.10)*	Student’s t-test
S7	FRET signal, ADAMTS-7 and TIMP-1 vs. $_{\Delta cat}$ADAMTS-7 and TIMP-1 (n=3)	Yes (>0.38)#	Student’s t-test

Supplemental Table S6: Differentially detected proteins between Apoe-/- and Apoe-/-Adamts7-/- mice after 16 weeks of Western diet. Data from Apoe-/- mice were reported as cohort #2 in Wierer et al.[12].

see next pages

	identifier	display_name	comparison	group1	group2	mean(group 1)	std(group1)	mean(group 2)	std(group2)	Fold-change	log2FC	T-statistics	pvalue	-LOG10 pvalue	test	padj1	correction1	s0
0	Aaas~P5874	Aaas	ko vs wt	ko	wt	24,9892089	0,93871885	24,6804429	0,55717736	1,23864773	0,30876594	0,1848853	0,66450729	0,17750025	Unpaired t-te	0,669861869	permutation FDR (250 perm	1
1	Aacs~Q9D2R	Aacs	ko vs wt	ko	wt	25,7604694	1,59482142	23,9209099	0,92222224	3,57900734	1,8395595	0,86433199	0,16500744	0,78249648	Unpaired t-te	0,075636727	permutation FDR (250 perm	1
2	Aak1~Q3UHJ	Aak1	ko vs wt	ko	wt	27,0868534	0,26632716	26,908058	0,33180627	1,13193834	0,17879537	0,13962723	0,56175383	0,25045396	Unpaired t-te	0,753886261	permutation FDR (250 perm	1
3	Aars1~Q8BG	Aars1	ko vs wt	ko	wt	27,7934884	0,56550878	24,548927	1,25251395	9,47785985	3,24456133	1,66907685	0,05287513	1,27674855	Unpaired t-te	0,0095	permutation FDR (250 perm	1
4	Abca1~P412	Abca1	ko vs wt	ko	wt	27,8842589	0,49211735	27,3680448	1,57954392	1,43019725	0,51621413	0,23982316	0,69352676	0,15893678	Unpaired t-te	0,59813912	permutation FDR (250 perm	1
5	Abca8b~Q8K	Abca8b	ko vs wt	ko	wt	24,5947454	1,00823898	25,0294437	1,20153011	0,7398485	-0,4346982	-0,214148	0,69594316	0,15742623	Unpaired t-te	0,624453475	permutation FDR (250 perm	1
6	Abcb1b~P06	Abcb1b	ko vs wt	ko	wt	26,2940843	0,21070092	25,8624141	0,16375556	1,34879416	0,43167019	0,36959738	0,0511309	1,29131658	Unpaired t-te	0,396064665	permutation FDR (250 perm	1
7	Abcb7~Q611	Abcb7	ko vs wt	ko	wt	25,0509412	1,32037986	25,6412959	0,08876483	0,66417959	-0,5903547	-0,3344973	0,49576484	0,30472427	Unpaired t-te	0,452900637	permutation FDR (250 perm	1
8	Abcc1~O353	Abcc1	ko vs wt	ko	wt	26,6096458	0,54347981	27,0328141	0,38534327	0,74578502	-0,4231683	-0,2989377	0,35542571	0,44925116	Unpaired t-te	0,48709108	permutation FDR (250 perm	1
9	Abcc3~B2RX	Abcc3	ko vs wt	ko	wt	25,7229832	1,97865117	24,4603489	0,63442256	2,39933438	1,26263423	0,56688997	0,36368683	0,43927242	Unpaired t-te	0,216174896	permutation FDR (250 perm	1
10	Abcc9~P701	Abcc9	ko vs wt	ko	wt	27,2957354	0,71246574	27,7190654	0,8840979	0,74570144	-0,42333	-0,2421321	0,60454752	0,21856955	Unpaired t-te	0,57894781	permutation FDR (250 perm	1
11	Abcd1~P484	Abcd1	ko vs wt	ko	wt	26,1517222	0,81859822	24,4112825	0,96151196	3,34136986	1,74043968	0,95208906	0,10693394	0,97088444	Unpaired t-te	0,059982879	permutation FDR (250 perm	1
12	Abcd3~P550	Abcd3	ko vs wt	ko	wt	26,0155783	1,59470123	26,863315	0,11670206	0,55565577	-0,8477367	-0,4405219	0,42576119	0,37083393	Unpaired t-te	0,333047717	permutation FDR (250 perm	1
13	Abce1~P612	Abce1	ko vs wt	ko	wt	29,1330175	0,40029395	29,28393	0,79239706	0,9006806	-0,1509125	-0,093962	0,82116874	0,08556759	Unpaired t-te	0,841935732	permutation FDR (250 perm	1
14	Abcf1~Q6P5	Abcf1	ko vs wt	ko	wt	29,2880519	0,27588725	29,2994336	0,0396834	0,9921418	-0,0113818	-0,0097972	0,94808602	0,02315226	Unpaired t-te	0,983991643	permutation FDR (250 perm	1
15	Abcf2~Q99L	Abcf2	ko vs wt	ko	wt	28,1613163	0,37297859	28,1221094	0,6799579	1,02754879	0,03920689	0,02567874	0,94565672	0,02426649	Unpaired t-te	0,955421953	permutation FDR (250 perm	1
16	Abcf3~Q8K2	Abcf3	ko vs wt	ko	wt	26,4217051	0,73591154	24,1869385	0,73638782	4,70686525	2,23476655	1,33654037	0,02636628	1,57895116	Unpaired t-te	0,014138486	permutation FDR (250 perm	1
17	Abcg1~Q643	Abcg1	ko vs wt	ko	wt	24,0901273	0,2222014	24,8720231	0,7653141	0,58160201	-0,7818959	-0,502453	0,28306966	0,54810667	Unpaired t-te	0,289989433	permutation FDR (250 perm	1
18	Abhd12~Q99	Abhd12	ko vs wt	ko	wt	28,1559824	0,89181577	27,318517	1,11171758	1,78690801	0,83746537	0,43174595	0,42783762	0,36872103	Unpaired t-te	0,335277587	permutation FDR (250 perm	1
19	Abhd14b~E9	Abhd14b	ko vs wt	ko	wt	25,7778191	2,21017377	26,7169414	0,10441006	0,52155009	-0,9391223	-0,4122251	0,51539919	0,28785627	Unpaired t-te	0,36407285	permutation FDR (250 perm	1
20	Abhd16a~Q9	Abhd16a	ko vs wt	ko	wt	27,1879168	0,66155808	27,5778329	0,25345507	0,76317397	-0,3899161	-0,2742204	0,40577805	0,39171145	Unpaired t-te	0,527025767	permutation FDR (250 perm	1
21	Abhd5~Q9DE	Abhd5	ko vs wt	ko	wt	25,4370606	1,32785872	24,9383841	0,29607416	1,41291677	0,49867649	0,27785887	0,5695955	0,24443345	Unpaired t-te	0,5272	permutation FDR (250 perm	1
22	Abi1~Q8CBW	Abi1	ko vs wt	ko	wt	27,3784878	1,16946022	27,8326559	0,28485882	0,72993093	-0,4541681	-0,2664379	0,55881713	0,25273029	Unpaired t-te	0,543867195	permutation FDR (250 perm	1
23	Abi3bp~Q59	Abi3bp	ko vs wt	ko	wt	31,4643837	0,88376986	31,9769784	0,17208024	0,70096064	-0,5125947	-0,3362263	0,39391848	0,40459365	Unpaired t-te	0,448021732	permutation FDR (250 perm	1
24	Abl1~P0052	Abl1	ko vs wt	ko	wt	24,5536691	1,30938922	23,8594885	0,55998969	1,61796527	0,69418064	0,3745441	0,45790898	0,33922084	Unpaired t-te	0,393611387	permutation FDR (250 perm	1
25	Ablim1~E9Q	Ablim1	ko vs wt	ko	wt	26,6398778	0,5390524	26,9839992	0,68098514	0,78778764	-0,3441213	-0,2187189	0,58408308	0,23352537	Unpaired t-te	0,617316022	permutation FDR (250 perm	1
26	Ablim2~Q8B	Ablim2	ko vs wt	ko	wt	24,5074414	0,24600055	24,8628737	0,78732085	0,78163537	-0,3554323	-0,2257356	0,59272604	0,22714599	Unpaired t-te	0,620999411	permutation FDR (250 perm	1
27	Abraxas1~Q	Abraxas1	ko vs wt	ko	wt	23,3444893	1,06655065	23,17427	1,03973413	1,12522955	0,17021935	0,08689049	0,8670651	0,06194829	Unpaired t-te	0,84952428	permutation FDR (250 perm	1
28	Abr~Q5SSL4	Abr	ko vs wt	ko	wt	27,9185682	0,38269636	27,8586113	0,13075942	1,0424346	0,05995687	0,0483712	0,81478415	0,08895743	Unpaired t-te	0,916681466	permutation FDR (250 perm	1
29	Acaa1a~Q92	Acaa1a	ko vs wt	ko	wt	28,2081648	0,88540704	27,9976532	0,20681931	1,15709843	0,21051159	0,13743678	0,71523592	0,14555068	Unpaired t-te	0,7588782	permutation FDR (250 perm	1
30	Acaa1b~Q8V	Acaa1b	ko vs wt	ko	wt	24,6024758	0,57130838	24,230709	0,4065218	1,29393655	0,37176688	0,25861602	0,43443172	0,36207847	Unpaired t-te	0,546494075	permutation FDR (250 perm	1
31	Acaa2~Q8BV	Acaa2	ko vs wt	ko	wt	32,6209271	0,3138314	32,6301394	0,23167866	0,99363488	-0,0092123	-0,0074038	0,97138998	0,01260638	Unpaired t-te	0,986569716	permutation FDR (250 perm	1
32	Acaca~Q5SW	Acaca	ko vs wt	ko	wt	31,2657509	0,05314885	31,6072277	0,24059163	0,78923303	-0,3414768	-0,2911465	0,17902347	0,74709004	Unpaired t-te	0,526669939	permutation FDR (250 perm	1
33	Acacb~E9Q4	Acacb	ko vs wt	ko	wt	28,1583202	0,06215561	28,2352994	0,36582175	0,94804062	-0,0769792	-0,0610388	0,79503488	0,09961382	Unpaired t-te	0,900832526	permutation FDR (250 perm	1
34	Acad10~Q8K	Acad10	ko vs wt	ko	wt	27,6081382	0,050228	26,4746018	0,86136767	2,19395881	1,13353644	0,70416099	0,20355523	0,69131774	Unpaired t-te	0,168351949	permutation FDR (250 perm	1
35	Acad11~Q80	Acad11	ko vs wt	ko	wt	27,7805164	0,71109475	27,3959037	0,76106565	1,3055093	0,38461274	0,22936253	0,59968037	0,22208016	Unpaired t-te	0,596126153	permutation FDR (250 perm	1
36	Acad8~Q9D7	Acad8	ko vs wt	ko	wt	27,281697	0,18558975	27,8193792	0,31954433	0,68887676	-0,5376822	-0,4301213	0,12370717	0,90760514	Unpaired t-te	0,346275792	permutation FDR (250 perm	1
37	Acad9~Q8JZ	Acad9	ko vs wt	ko	wt	25,3278478	1,51573143	24,3242575	0,46792422	2,0049833	1,00359022	0,51849865	0,34671005	0,46003357	Unpaired t-te	0,253263005	permutation FDR (250 perm	1
38	Acadl~P5117	Acadl	ko vs wt	ko	wt	31,5091343	0,35038051	31,4452939	0,41049013	1,04524451	0,06384046	0,04715655	0,86598204	0,06249112	Unpaired t-te	0,919815994	permutation FDR (250 perm	1
39	Acadm~P45	Acadm	ko vs wt	ko	wt	31,9443706	0,15950167	31,831448	0,42112504	1,08141671	0,11292255	0,08608907	0,74676616	0,12681537	Unpaired t-te	0,858457581	permutation FDR (250 perm	1
40	Acadsb~Q9D	Acadsb	ko vs wt	ko	wt	29,6217274	0,31758329	29,2691129	0,37881975	1,27687255	0,35261453	0,26620244	0,34199865	0,46597561	Unpaired t-te	0,542015239	permutation FDR (250 perm	1
41	Acads~Q074	Acads	ko vs wt	ko	wt	31,2670519	0,3472171	30,9357914	0,24279083	1,2581006	0,33126043	0,26208716	0,26502616	0,57671125	Unpaired t-te	0,541380067	permutation FDR (250 perm	1
42	Acadvl~P505	Acadvl	ko vs wt	ko	wt	32,3013936	0,2817141	32,7120336	0,22656339	0,75228955	-0,4106401	-0,3343164	0,13405687	0,87271093	Unpaired t-te	0,439384912	permutation FDR (250 perm	1
43	Acan~Q6128	Acan	ko vs wt	ko	wt	31,9733048	0,72257408	31,9129466	0,60998871	1,04272465	0,06035823	0,03772231	0,92400113	0,0343275	Unpaired t-te	0,933984595	permutation FDR (250 perm	1
44	Acap1~Q8K2	Acap1	ko vs wt	ko	wt	24,3308255	0,42899304	23,9585159	0,59937879	1,29442337	0,37230956	0,24972311	0,49815126	0,30263877	Unpaired t-te	0,569574699	permutation FDR (250 perm	1
45	Acap2~Q6ZC	Acap2	ko vs wt	ko	wt	27,0891192	0,3280773	26,2917322	0,25079581	1,73795061	0,79738708	0,63311046	0,02837058	1,54713176	Unpaired t-te	0,16921781	permutation FDR (250 perm	1
46	Acat1~Q8QZ	Acat1	ko vs wt	ko	wt	32,1996812	0,35978825	32,4208508	0,15338421	0,85786964	-0,2211697	-0,1791813	0,39444614	0,40401229	Unpaired t-te	0,681097835	permutation FDR (250 perm	1
47	Acat2~Q8CA	Acat2	ko vs wt	ko	wt	24,6140433	1,20241803	23,8170312	0,03255554	1,73749892	0,79701208	0,47032518	0,33424361	0,47593689	Unpaired t-te	0,304598039	permutation FDR (250 perm	1
48	Acbd3~Q8BM	Acbd3	ko vs wt	ko	wt	28,205873	0,56490821	27,8449587	0,33527912	1,28423869	0,36091337	0,25720518	0,41222652	0,38486407	Unpaired t-te	0,549030377	permutation FDR (250 perm	1
49	Ace~P09470	Ace	ko vs wt	ko	wt	31,6543135	0,37060432	31,5831233	0,25902653	1,05058298	0,07119011	0,05554543	0,81054033	0,09122537	Unpaired t-te	0,90311374	permutation FDR (250 perm	1
50	Acin1~Q9JIX	Acin1	ko vs wt	ko	wt	27,6089925	0,73424109	27,2291607	0,46634847	1,30119017	0,37983183	0,24711426	0,51108017	0,29151097	Unpaired t-te	0,564001208	permutation FDR (250 perm	1
51	Acly~Q3V11	Acly	ko vs wt	ko	wt	32,010583	0,02837957	31,9954832	0,30392736	1,01052138	0,01509985	0,01242241	0,95045697	0,02206754	Unpaired t-te	0,979829587	permutation FDR (250 perm	1
52	Aco1~P2827	Aco1	ko vs wt	ko	wt	27,2480942	0,86139274	26,6661461	0,072997	1,49686912	0,58194809	0,38796623	0,32701729	0,48542928	Unpaired t-te	0,390068325	permutation FDR (250 perm	1
53	Aco2~Q99KI	Aco2	ko vs wt	ko	wt	32,4366148	0,41499543	32,212425	0,24050607	1,16812109	0,22418983	0,1732778	0,4804567	0,31834575	Unpaired t-te	0,68893042	permutation FDR (250 perm	1
54	Acot11~Q8V	Acot11	ko vs wt	ko	wt	28,7936991	0,43527754	29,033083	0,25151234	0,84710697	-0,2393839	-0,1830332	0,47261775	0,32548997	Unpaired t-te	0,673700203	permutation FDR (250 perm	1
55	Acot13~Q9C	Acot13	ko vs wt	ko	wt	27,9373119	0,54168803	27,751425	0,66050917	1,13751608	0,18588694	0,11899889	0,75873705	0,11990871	Unpaired t-te	0,792624232	permutation FDR (250 perm	1
56	Acot2~Q9QY	Acot2	ko vs wt	ko	wt	26,176168	0,45687261	25,4162316	0,46164828	1,69341596	0,75993639	0,5352845	0,1401177	0,853507	Unpaired t-te	0,236597633	permutation FDR (250 perm	1
57	Acot7~Q91V	Acot7	ko vs wt	ko	wt	28,1185386	0,67903079	26,9557075	0,46238073	2,23896361	1,16283108	0,76984089	0,07174858	1,14418671	Unpaired t-te	0,106279472	permutation FDR (250 perm	1
58	Acot9~Q9R0	Acot9	ko vs wt	ko	wt	30,4265901	0,30449527	30,4533171	0,35982692	0,98164482	-0,026727	-0,0204138	0,93550315	0,02895474	Unpaired t-te	0,964590491	permutation FDR (250 perm	1
59	Acox1~Q9R0	Acox1	ko vs wt	ko	wt	26,5679374	0,7372328	25,5670454	1,17326113	2,00123707	1,00089208	0,51794253	0,36056725	0,44303173	Unpaired t-te	0,261956036	permutation FDR (250 perm	1
60	Acox3~Q9EP	Acox3	ko vs wt	ko	wt	25,3264787	1,27831818	25,5484806	0,6060186	0,85737486	-0,222002	-0,1197795	0,80604084	0,09364295	Unpaired t-te	0,788559933	permutation FDR (250 perm	1
61	Acp1~Q9D35	Acp1	ko vs wt	ko	wt	28,7160635	0,68841369	27,3553096	0,08074735	2,56819345	1,36075388	0,9709018	0,04028698	1,39483525	Unpaired t-te	0,061290938	permutation FDR (250 perm	1

62	Acp5~Q0511	Acp5	ko vs wt	ko	wt	24,6395326	0,88615667	23,9166033	0,41594962	1,65052988	0,72292926	0,45463253	0,28001807	0,55281395	Unpaired t-te	0,304679272	permutation FDR (250 perm	1
63	Acsf2~Q8VC	Acsf2	ko vs wt	ko	wt	28,2540296	1,18350226	27,7399987	0,49312576	1,42803454	0,51403087	0,29088559	0,53689166	0,27011334	Unpaired t-te	0,502678272	permutation FDR (250 perm	1
64	Acsf3~Q3UR	Acsf3	ko vs wt	ko	wt	26,4029994	1,4339212	27,3865859	0,34063898	0,50572099	-0,9835864	-0,5281843	0,32611169	0,48663363	Unpaired t-te	0,24823641	permutation FDR (250 perm	1
65	Acsl1~P4121	Acsl1	ko vs wt	ko	wt	32,133991	0,26858668	32,2645032	0,03456752	0,91350707	-0,1305122	-0,1128039	0,46412157	0,33336824	Unpaired t-te	0,806077821	permutation FDR (250 perm	1
66	Acsl4~Q9QU	Acsl4	ko vs wt	ko	wt	27,7505381	1,02058078	26,9617098	0,67780253	1,72767071	0,78882827	0,44831469	0,34660215	0,46016875	Unpaired t-te	0,308339267	permutation FDR (250 perm	1
67	Acsl5~Q8JZR	Acsl5	ko vs wt	ko	wt	28,2033049	0,75292794	28,0846548	0,29578362	1,08571845	0,11865003	0,08003909	0,81724178	0,08764944	Unpaired t-te	0,862739202	permutation FDR (250 perm	1
68	Acss2~Q9QX	Acss2	ko vs wt	ko	wt	24,8659134	1,16745149	24,195932	0,21266525	1,59105248	0,66998142	0,39629815	0,39769772	0,4004469	Unpaired t-te	0,378105756	permutation FDR (250 perm	1
69	Acta1~P6813	Acta1	ko vs wt	ko	wt	25,8195055	2,003491	23,2065169	0,5711856	6,11769666	2,61298858	1,17427058	0,10560631	0,97631013	Unpaired t-te	0,027289421	permutation FDR (250 perm	1
70	Acta2~P6273	Acta2	ko vs wt	ko	wt	37,9353484	0,36614621	38,2201472	0,08463303	0,82085608	-0,2847988	-0,2334986	0,27442563	0,56157534	Unpaired t-te	0,593091667	permutation FDR (250 perm	1
71	Actbl2~Q8BF	Actbl2	ko vs wt	ko	wt	31,3145905	0,44333169	31,9270789	0,20005203	0,65406755	-0,6124885	-0,4738986	0,09679915	1,01412846	Unpaired t-te	0,285162019	permutation FDR (250 perm	1
72	Actb~P6071C	Actb	ko vs wt	ko	wt	26,1300941	2,8234071	31,22557	0,16501667	0,02924886	-5,095476	-1,934306	0,05184319	1,28530828	Unpaired t-te	0,004649386	permutation FDR (250 perm	1
73	Actc1~P6803	Actc1	ko vs wt	ko	wt	34,5074802	0,22089184	34,1887637	0,4052621	1,24722047	0,31871651	0,24261705	0,38924947	0,40977197	Unpaired t-te	0,586006573	permutation FDR (250 perm	1
74	Actg1~P6326	Actg1	ko vs wt	ko	wt	35,5995858	0,19275548	35,7554757	0,09145725	0,89757853	-0,1558899	-0,138113	0,28455463	0,54583435	Unpaired t-te	0,753126126	permutation FDR (250 perm	1
75	Actl6a~Q9Z2	Actl6a	ko vs wt	ko	wt	26,6070028	0,53126225	25,4546192	1,34526488	2,22280849	1,15238365	0,57634335	0,35001816	0,45590943	Unpaired t-te	0,230146851	permutation FDR (250 perm	1
76	Actn1~A1BN	Actn1	ko vs wt	ko	wt	36,5770509	0,29969413	36,876547	0,05892153	0,81253614	-0,2994961	-0,2542468	0,18161146	0,74085674	Unpaired t-te	0,562708547	permutation FDR (250 perm	1
77	Actn1~Q7TPI	Actn1	ko vs wt	ko	wt	28,6617949	0,84678083	27,9371746	0,09955061	1,65246566	0,72462029	0,4850427	0,23517558	0,62860778	Unpaired t-te	0,288187522	permutation FDR (250 perm	1
78	Actn2~Q9JI9	Actn2	ko vs wt	ko	wt	28,5080539	3,10765075	25,698122	3,12124085	7,0125146	2,80993187	0,73092811	0,37578687	0,4250584	Unpaired t-te	0,126465825	permutation FDR (250 perm	1
79	Actn3~O889	Actn3	ko vs wt	ko	wt	28,3357277	1,34610396	23,8436422	0,29803716	22,503625	4,49208551	2,48835897	0,00788773	2,10304789	Unpaired t-te	0,002666667	permutation FDR (250 perm	1
80	Actn4~P5778	Actn4	ko vs wt	ko	wt	34,6572056	0,32705705	34,8493608	0,1085083	0,87529715	-0,1921552	-0,1596213	0,40029006	0,39762519	Unpaired t-te	0,717899601	permutation FDR (250 perm	1
81	Actr10~Q9Q	Actr10	ko vs wt	ko	wt	25,7832529	1,18545263	25,2550287	1,48709276	1,44215302	0,52822425	0,2342818	0,69752198	0,1564421	Unpaired t-te	0,590859016	permutation FDR (250 perm	1
82	Actr1a~P611	Actr1a	ko vs wt	ko	wt	28,3536125	0,6575499	28,3301396	0,26336966	1,01640325	0,0234729	0,01649705	0,95824511	0,01852339	Unpaired t-te	0,971004454	permutation FDR (250 perm	1
83	Actr1b~Q8R	Actr1b	ko vs wt	ko	wt	29,7154029	0,37663433	28,5520622	0,10910983	2,23975459	1,16334067	0,9452434	0,00882414	2,05432777	Unpaired t-te	0,061867795	permutation FDR (250 perm	1
84	Actr2~P6116	Actr2	ko vs wt	ko	wt	31,4586574	0,27360284	31,2054604	0,27880273	1,19184534	0,25319703	0,20213349	0,37004621	0,43174405	Unpaired t-te	0,641974832	permutation FDR (250 perm	1
85	Actr3~Q99JY	Actr3	ko vs wt	ko	wt	31,5350783	0,17657609	31,1865126	0,55820511	1,27329417	0,34856577	0,2476201	0,47324004	0,32491852	Unpaired t-te	0,58614098	permutation FDR (250 perm	1
86	Adam10~O3	Adam10	ko vs wt	ko	wt	25,17951	0,84698531	23,851354	0,33955304	2,51081541	1,32815597	0,85977494	0,0676361	1,16982142	Unpaired t-te	0,08034398	permutation FDR (250 perm	1
87	Adam15~O8	Adam15	ko vs wt	ko	wt	24,4318663	0,96824457	24,2564225	0,07438869	1,12931176	0,17544381	0,11235699	0,7748179	0,11080036	Unpaired t-te	0,80703138	permutation FDR (250 perm	1
88	Adamts2~Q8	Adamts2	ko vs wt	ko	wt	25,4657526	1,18721228	23,6164327	0,85698407	3,60330272	1,84931986	0,96575459	0,09955412	1,00194078	Unpaired t-te	0,051513453	permutation FDR (250 perm	1
89	Adamts4~E9	Adamts4	ko vs wt	ko	wt	24,5418644	1,11968377	24,6176141	1,0976936	0,94884887	-0,0757498	-0,037684	0,94355102	0,02523461	Unpaired t-te	0,934387679	permutation FDR (250 perm	1
90	Adamts5~Q9	Adamts5	ko vs wt	ko	wt	27,6721762	0,40889382	26,7657823	0,22294901	1,87435462	0,90639393	0,70598349	0,02538065	1,59549722	Unpaired t-te	0,134059983	permutation FDR (250 perm	1
91	Adamtsl1~Q	Adamtsl1	ko vs wt	ko	wt	26,7289971	0,04770696	26,5112204	0,23847308	1,16294007	0,21777675	0,18599716	0,32493901	0,48819814	Unpaired t-te	0,687720449	permutation FDR (250 perm	1
92	Adamtsl3~G	Adamtsl3	ko vs wt	ko	wt	29,1220757	0,63383094	28,5854586	0,43946863	1,45056714	0,53661707	0,36255863	0,31455194	0,50230764	Unpaired t-te	0,403703022	permutation FDR (250 perm	1
93	Adamtsl4~Q	Adamtsl4	ko vs wt	ko	wt	28,8956349	0,14446202	29,267032	0,21316671	0,77303354	-0,3713971	-0,3168191	0,11409041	0,94275087	Unpaired t-te	0,475020794	permutation FDR (250 perm	1
94	Adamtsl5~D	Adamtsl5	ko vs wt	ko	wt	29,8259655	0,81855452	29,8478979	0,13874666	0,98491256	-0,0219324	-0,0147925	0,96640269	0,01484187	Unpaired t-te	0,974374248	permutation FDR (250 perm	1
95	Adap2~Q8R2	Adap2	ko vs wt	ko	wt	26,7769343	0,34647074	25,3324687	1,16409986	2,7216199	1,44446559	0,78201812	0,21683055	0,66387954	Unpaired t-te	0,130627536	permutation FDR (250 perm	1
96	Adck1~Q9D0	Adck1	ko vs wt	ko	wt	23,9407698	0,54691475	24,9634542	0,72587334	0,49219969	-1,0226844	-0,6381326	0,17576046	0,75507883	Unpaired t-te	0,176155248	permutation FDR (250 perm	1
97	Adcy5~P843	Adcy5	ko vs wt	ko	wt	27,9247561	0,71364747	28,4606408	0,1348431	0,68973562	-0,5358846	-0,3766109	0,28679323	0,54243111	Unpaired t-te	0,399307439	permutation FDR (250 perm	1
98	Add1~Q9QY	Add1	ko vs wt	ko	wt	29,7090844	0,2733072	29,764764	0,08310538	0,96214107	-0,0556797	-0,0476554	0,75839597	0,12010399	Unpaired t-te	0,918711955	permutation FDR (250 perm	1
99	Add2~Q9QYE	Add2	ko vs wt	ko	wt	25,3335809	1,31099724	25,2481276	0,92824182	1,06102111	0,08545336	0,04268701	0,93534844	0,02902657	Unpaired t-te	0,925963733	permutation FDR (250 perm	1
100	Add3~Q9QYE	Add3	ko vs wt	ko	wt	30,4278097	0,14132863	30,4010977	0,14945581	1,01868784	0,02671202	0,02356564	0,85091902	0,07011177	Unpaired t-te	0,958079958	permutation FDR (250 perm	1
101	Adgre5~A0A	Adgre5	ko vs wt	ko	wt	26,628735	0,44630608	26,8868557	0,17833417	0,83617644	-0,2581207	-0,2005793	0,41637778	0,38051246	Unpaired t-te	0,644630409	permutation FDR (250 perm	1
102	Adh1~P0032	Adh1	ko vs wt	ko	wt	30,1537444	0,86797152	29,4639466	0,73547914	1,61305744	0,68979782	0,40053023	0,38580225	0,41363524	Unpaired t-te	0,362025693	permutation FDR (250 perm	1
103	Adh5~P2847	Adh5	ko vs wt	ko	wt	27,6997152	0,40430941	26,615362	0,37069999	2,1204245	1,08435312	0,80263258	0,03098446	1,50885601	Unpaired t-te	0,096154562	permutation FDR (250 perm	1
104	Adie~F8WHS	Adie	ko vs wt	ko	wt	27,8316945	0,38878441	27,6249924	0,19776585	1,15404714	0,20670215	0,16347044	0,47185192	0,32619427	Unpaired t-te	0,70736824	permutation FDR (250 perm	1
105	Adipoq~Q60	Adipoq	ko vs wt	ko	wt	29,1672329	0,44858641	30,045779	0,6231723	0,54391529	-0,8785461	-0,5813856	0,17440003	0,75845345	Unpaired t-te	0,211972717	permutation FDR (250 perm	1
106	Adissp~Q9D1	Adissp	ko vs wt	ko	wt	25,513163	1,40453914	24,884865	1,08216464	1,54574042	0,62829806	0,29707445	0,59790408	0,22336848	Unpaired t-te	0,490375	permutation FDR (250 perm	1
107	Adk~P55264	Adk	ko vs wt	ko	wt	26,6352944	0,77334407	26,5087441	0,44546128	1,09168024	0,12655034	0,08183455	0,82619688	0,08291645	Unpaired t-te	0,858198139	permutation FDR (250 perm	1
108	Adnp~A2BD	Adnp	ko vs wt	ko	wt	25,670422	0,9617812	24,8072912	1,31368932	1,81898141	0,8631308	0,41452148	0,47715466	0,32134083	Unpaired t-te	0,356718157	permutation FDR (250 perm	1
109	Adprh~P549	Adprh	ko vs wt	ko	wt	27,1729074	0,56428976	26,3906331	0,33961112	1,71983991	0,78227428	0,55688465	0,11164108	0,95217598	Unpaired t-te	0,215936628	permutation FDR (250 perm	1
110	Adsl~P54822	Adsl	ko vs wt	ko	wt	27,5339252	0,84911935	24,1568034	1,05636216	10,3899858	3,37712177	1,78356422	0,02301619	1,63796664	Unpaired t-te	0,00513394	permutation FDR (250 perm	1
111	Adss1~P286	Adss1	ko vs wt	ko	wt	25,8622299	1,03450403	24,4948561	1,26753942	2,58000497	1,36737384	0,65832139	0,27847742	0,55521001	Unpaired t-te	0,16366343	permutation FDR (250 perm	1
112	Adss2~P466	Adss2	ko vs wt	ko	wt	25,3260867	0,47460119	24,5241162	0,23834037	1,74348079	0,80197047	0,60677603	0,05816079	1,23536967	Unpaired t-te	0,184497458	permutation FDR (250 perm	1
113	Aebp1~Q640	Aebp1	ko vs wt	ko	wt	33,5995936	0,1673978	33,5856396	0,18321529	1,00971908	0,01395396	0,01201239	0,93533522	0,02903271	Unpaired t-te	0,97940183	permutation FDR (250 perm	1
114	Afap1~Q80Y	Afap1	ko vs wt	ko	wt	27,6217787	0,25487195	27,8242768	0,38890875	0,86904445	-0,2024981	-0,1543555	0,56057501	0,25136627	Unpaired t-te	0,731581039	permutation FDR (250 perm	1
115	Afdn~Q9QZC	Afdn	ko vs wt	ko	wt	26,5161222	0,08204166	26,4973469	0,21771064	1,01309914	0,01877536	0,01617078	0,91630572	0,0379596	Unpaired t-te	0,97240733	permutation FDR (250 perm	1
116	Afg1l~Q3V3	Afg1l	ko vs wt	ko	wt	27,3184269	0,73894787	27,2639265	0,27033268	1,03849939	0,05450037	0,03713829	0,91270915	0,0396676	Unpaired t-te	0,935718609	permutation FDR (250 perm	1
117	Afg2a~Q3UN	Afg2a	ko vs wt	ko	wt	26,7104392	0,26167939	26,5006002	0,29696414	1,1565591	0,20983899	0,16671254	0,46377315	0,3336944	Unpaired t-te	0,704692814	permutation FDR (250 perm	1
118	Afg3l2~Q8JZ	Afg3l2	ko vs wt	ko	wt	29,0766074	0,50826034	28,722584	0,53148936	1,27812015	0,35402347	0,23972137	0,49760152	0,3031183	Unpaired t-te	0,579241235	permutation FDR (250 perm	1
119	Aftph~Q80W	Aftph	ko vs wt	ko	wt	24,1806654	0,6025332	25,0488226	0,18480706	0,54784619	-0,8681572	-0,6329486	0,08327937	1,07946256	Unpaired t-te	0,176126833	permutation FDR (250 perm	1
120	Agap3~Q8VH	Agap3	ko vs wt	ko	wt	24,245919	0,24770292	26,3373196	0,56910794	0,23465277	-2,0914006	-1,4655142	0,02438838	1,61281706	Unpaired t-te	0,015659641	permutation FDR (250 perm	1
121	Agfg1~Q8K2	Agfg1	ko vs wt	ko	wt	25,7720638	1,38064688	26,8775922	0,11859866	0,46473224	-1,1055284	-0,6136655	0,25980753	0,58534826	Unpaired t-te	0,195020503	permutation FDR (250 perm	1
122	Agk~Q9ESW	Agk	ko vs wt	ko	wt	26,7420645	0,19393778	26,9163675	0,22664137	0,88619555	-0,174303	-0,1457992	0,42518966	0,37141731	Unpaired t-te	0,742029952	permutation FDR (250 perm	1
123	Agl~F8VPN4	Agl	ko vs wt	ko	wt	28,7309002	0,73864226	28,2800586	0,32295252	1,36683737	0,4508416	0,30385297	0,3993847	0,39860858	Unpaired t-te	0,48298337	permutation FDR (250 perm	1
124	Ago1~Q8CJG	Ago1	ko vs wt	ko	wt	27,1483399	0,4914937	26,580521	0,15999679	1,48228084	0,56781881	0,43494833	0,1387624	0,85772819	Unpaired t-te	0,329317291	permutation FDR (250 perm	1
125	Ago2~Q8CJG	Ago2	ko vs wt	ko	wt	29,3588705	0,40028537	29,298294	0,36297679	1,04288244	0,06057653	0,04502568	0,86829059	0,06133491	Unpaired t-te	0,922528453	permutation FDR (250 perm	1

126	Ago3~Q8CJF!	Ago3	ko vs wt	ko	wt	25,8145139	0,44818669	26,1240679	0,10789943	0,80689115	-0,309554	-0,2437866	0,32344457	0,49020014	Unpaired t-te	0,577834234	permutation FDR (250 perm	1
127	Agpat2~Q8K:	Agpat2	ko vs wt	ko	wt	27,4330682	0,19965163	27,7714926	0,27735918	0,79090462	-0,3384244	-0,2757047	0,22421222	0,64934072	Unpaired t-te	0,531371324	permutation FDR (250 perm	1
128	Agpat3~Q9D	Agpat3	ko vs wt	ko	wt	27,2164165	0,57893404	27,7249366	0,01975213	0,70294315	-0,5085201	-0,3810454	0,22551551	0,64682359	Unpaired t-te	0,397724638	permutation FDR (250 perm	1
129	Agpat4~Q8K	Agpat4	ko vs wt	ko	wt	24,2298899	0,40095143	24,4935692	1,11908651	0,83296092	-0,2636793	-0,1445231	0,77539951	0,11047448	Unpaired t-te	0,754061902	permutation FDR (250 perm	1
130	Agrn~M0QW	Agrn	ko vs wt	ko	wt	31,7694701	0,21604339	31,577626	0,05299465	1,14222275	0,19184403	0,16973742	0,22399014	0,64977111	Unpaired t-te	0,701774721	permutation FDR (250 perm	1
131	Ahctf1~Q8CJ	Ahctf1	ko vs wt	ko	wt	26,8364066	0,18943015	25,5875997	1,71441197	2,37644808	1,24880688	0,56323709	0,41130202	0,38583916	Unpaired t-te	0,248345859	permutation FDR (250 perm	1
132	Ahcyl1~Q80S	Ahcyl1	ko vs wt	ko	wt	28,8686584	0,5563298	28,8354727	0,38955355	1,02326913	0,03318564	0,02331868	0,9405365	0,02662435	Unpaired t-te	0,958097843	permutation FDR (250 perm	1
133	Ahcyl2~Q68F	Ahcyl2	ko vs wt	ko	wt	24,5981637	0,89491425	25,3641513	0,7707033	0,58805065	-0,7659877	-0,4374659	0,35745652	0,44677678	Unpaired t-te	0,321342551	permutation FDR (250 perm	1
134	Ahcy~P5024?	Ahcy	ko vs wt	ko	wt	26,5866821	0,76703845	24,1254704	1,10049843	5,50679041	2,4612117	1,29854773	0,06418489	1,19256722	Unpaired t-te	0,0193156	permutation FDR (250 perm	1
135	Ahnak~E9Q6	Ahnak	ko vs wt	ko	wt	35,1771076	0,3007209	35,6001766	0,08186125	0,74583629	-0,4230691	-0,357619	0,0884762	1,05317356	Unpaired t-te	0,419150765	permutation FDR (250 perm	1
136	Ahsa1~Q8BK	Ahsa1	ko vs wt	ko	wt	27,9671972	0,36657186	27,0972168	0,330443	1,82763802	0,86998036	0,66145199	0,04388227	1,35771089	Unpaired t-te	0,155651106	permutation FDR (250 perm	1
137	Ahsg~P2969!	Ahsg	ko vs wt	ko	wt	31,1996543	0,39325777	31,4782555	0,96505819	0,82438995	-0,2786012	-0,1620547	0,72963129	0,13689655	Unpaired t-te	0,723439295	permutation FDR (250 perm	1
138	Aida~Q8C4Q	Aida	ko vs wt	ko	wt	25,5053651	1,07391237	26,1865899	0,35483747	0,62363558	-0,6812249	-0,4081958	0,36732942	0,43494429	Unpaired t-te	0,35901151	permutation FDR (250 perm	1
139	Aifm1~Q9Z0	Aifm1	ko vs wt	ko	wt	29,416019	0,24749505	29,9723867	0,08017267	0,68001207	-0,5563677	-0,4822355	0,02362125	1,62669709	Unpaired t-te	0,282167669	permutation FDR (250 perm	1
140	Aifm2~Q8BU	Aifm2	ko vs wt	ko	wt	27,4610705	0,12710165	27,26578	0,49279023	1,14495464	0,19529044	0,14400906	0,63443988	0,19760953	Unpaired t-te	0,75724782	permutation FDR (250 perm	1
141	Aimp1~P312	Aimp1	ko vs wt	ko	wt	29,8788471	0,1338362	29,7470706	0,07241228	1,09564197	0,13177643	0,12059757	0,21649785	0,66454642	Unpaired t-te	0,786069852	permutation FDR (250 perm	1
142	Aimp2~Q8R(	Aimp2	ko vs wt	ko	wt	27,5567263	0,06720763	27,2177647	1,10713321	1,26484587	0,3389616	0,19001981	0,70739773	0,15033634	Unpaired t-te	0,683315835	permutation FDR (250 perm	1
143	Aip~O08915	Aip	ko vs wt	ko	wt	28,6913674	0,28013633	28,784358	0,0811949	0,93757721	-0,0929906	-0,0793689	0,61878562	0,20845979	Unpaired t-te	0,864342795	permutation FDR (250 perm	1
144	Ak1~Q9R0Y5	Ak1	ko vs wt	ko	wt	28,7951913	0,35196872	27,8021042	1,15464256	1,99043955	0,99308706	0,53932137	0,34758066	0,45894439	Unpaired t-te	0,260137234	permutation FDR (250 perm	1
145	Ak2~Q9WTP	Ak2	ko vs wt	ko	wt	27,7647817	0,81856204	28,070802	0,63207475	0,80886997	-0,3060203	-0,1854144	0,65824378	0,18161323	Unpaired t-te	0,669044993	permutation FDR (250 perm	1
146	Ak3~Q9WTP	Ak3	ko vs wt	ko	wt	30,3688989	0,48417098	30,1677106	0,36650388	1,14964492	0,20118834	0,14566343	0,62056501	0,20721271	Unpaired t-te	0,738569731	permutation FDR (250 perm	1
147	Akap12~Q9V	Akap12	ko vs wt	ko	wt	24,9115297	0,57451945	24,9356214	1,07008666	0,98343955	-0,0240917	-0,0131924	0,97870573	0,00934787	Unpaired t-te	0,977708159	permutation FDR (250 perm	1
148	Akap2~O549	Akap2	ko vs wt	ko	wt	27,4345444	0,51217068	27,8780801	0,55835248	0,7353303	-0,4435357	-0,2970225	0,41906768	0,37771583	Unpaired t-te	0,495366584	permutation FDR (250 perm	1
149	Akap8~Q9DB	Akap8	ko vs wt	ko	wt	24,3456237	0,85454002	26,4948552	0,25975473	0,22543267	-2,1492315	-1,4079938	0,01688441	1,77251424	Unpaired t-te	0,012308725	permutation FDR (250 perm	1
150	Akap9~Q70F	Akap9	ko vs wt	ko	wt	26,0242034	1,63730723	27,5346662	0,28118636	0,35099861	-1,5104628	-0,7682987	0,20861745	0,68064937	Unpaired t-te	0,119916482	permutation FDR (250 perm	1
151	Akr1a1~Q9JI	Akr1a1	ko vs wt	ko	wt	29,3050399	0,40623525	29,0954242	0,42871508	1,15638011	0,2096157	0,15153472	0,61269279	0,21275723	Unpaired t-te	0,73144025	permutation FDR (250 perm	1
152	Akr1b10~G5I	Akr1b10	ko vs wt	ko	wt	27,8079956	0,73537719	26,8483808	1,67859837	1,94479059	0,95961482	0,42449609	0,5114257	0,29121745	Unpaired t-te	0,359484438	permutation FDR (250 perm	1
153	Akr1b1~P453	Akr1b1	ko vs wt	ko	wt	29,8506676	0,8089466	30,2799768	0,51473173	0,74261729	-0,4293092	-0,2696463	0,50099041	0,30017059	Unpaired t-te	0,529900674	permutation FDR (250 perm	1
154	Akr1b8~P453	Akr1b8	ko vs wt	ko	wt	24,1252312	1,20163319	23,9548653	0,16860336	1,12534383	0,17036586	0,09998393	0,82355976	0,08430488	Unpaired t-te	0,828867071	permutation FDR (250 perm	1
155	Akr1e2~Q9D	Akr1e2	ko vs wt	ko	wt	24,4172883	0,94324819	27,1401705	0,12032364	0,15147145	-2,7228822	-1,7553496	0,01421718	1,84718653	Unpaired t-te	0,006251469	permutation FDR (250 perm	1
156	Akr7a2~Q8C	Akr7a2	ko vs wt	ko	wt	26,9340406	0,439226	27,061349	0,44205637	0,91553796	-0,1273084	-0,0907719	0,76665898	0,11539777	Unpaired t-te	0,842177424	permutation FDR (250 perm	1
157	Akt1~P3175C	Akt1	ko vs wt	ko	wt	27,0586901	0,47444154	24,6679522	1,42873431	5,24425537	2,39073794	1,16806901	0,13328193	0,87522874	Unpaired t-te	0,039694328	permutation FDR (250 perm	1
158	Akt2~Q6082	Akt2	ko vs wt	ko	wt	27,3003588	0,2885494	26,1099259	0,70205613	2,28221214	1,1904329	0,7813104	0,1270617	0,89598532	Unpaired t-te	0,126514584	permutation FDR (250 perm	1
159	Alb~P07724	Alb	ko vs wt	ko	wt	34,3850084	0,26001588	34,3367131	0,36641359	1,03404237	0,04829531	0,03716619	0,88119608	0,05492744	Unpaired t-te	0,936567941	permutation FDR (250 perm	1
160	Alcam~Q614	Alcam	ko vs wt	ko	wt	25,7945063	1,39469513	25,1372389	1,00242332	1,57709271	0,65726747	0,31709746	0,56701762	0,24640344	Unpaired t-te	0,46172335	permutation FDR (250 perm	1
161	Aldh18a1~Q!	Aldh18a1	ko vs wt	ko	wt	28,220403	0,96003231	27,6999828	0,47884032	1,43437296	0,5204202	0,31549966	0,46171425	0,33562672	Unpaired t-te	0,46520076	permutation FDR (250 perm	1
162	Aldh1a1~P24	Aldh1a1	ko vs wt	ko	wt	25,9767363	2,70307359	23,396877	0,89655541	5,97881382	2,57985929	0,96103091	0,20196128	0,69473188	Unpaired t-te	0,057592191	permutation FDR (250 perm	1
163	Aldh1l1~Q8R	Aldh1l1	ko vs wt	ko	wt	25,0870382	1,97642396	23,7166719	0,98551292	2,58536195	1,37036627	0,58636592	0,35543968	0,44923409	Unpaired t-te	0,19802313	permutation FDR (250 perm	1
164	Aldh1l2~Q8K	Aldh1l2	ko vs wt	ko	wt	24,9439425	1,16347751	24,8923025	0,79327402	1,03644244	0,05164	0,02753929	0,95523277	0,01989079	Unpaired t-te	0,9509512	permutation FDR (250 perm	1
165	Aldh2~P4773	Aldh2	ko vs wt	ko	wt	32,3517701	0,74110972	31,5996518	0,28789144	1,68426393	0,75211823	0,51031287	0,18442618	0,73417743	Unpaired t-te	0,256708953	permutation FDR (250 perm	1
166	Aldh3a2~P47	Aldh3a2	ko vs wt	ko	wt	28,5745656	0,53364511	27,8792253	0,60939036	1,61926631	0,69534028	0,45455375	0,26138636	0,58271709	Unpaired t-te	0,309964423	permutation FDR (250 perm	1
167	Aldh4a1~Q8(	Aldh4a1	ko vs wt	ko	wt	27,992497	0,94507105	27,2088736	1,10537246	1,72144895	0,78362339	0,40119399	0,45924793	0,3379528	Unpaired t-te	0,366936113	permutation FDR (250 perm	1
168	Aldh6a1~Q9I	Aldh6a1	ko vs wt	ko	wt	30,2572074	0,20659382	30,2630081	0,88437512	0,99598738	-0,0058006	-0,0035443	0,99350688	0,00282912	Unpaired t-te	0,993603538	permutation FDR (250 perm	1
169	Aldh7a1~Q9I	Aldh7a1	ko vs wt	ko	wt	25,2384524	0,70700224	26,267409	0,14618499	0,49006444	-1,0289566	-0,7240702	0,08268739	1,08256074	Unpaired t-te	0,136539804	permutation FDR (250 perm	1
170	Aldh8a1~Q8I	Aldh8a1	ko vs wt	ko	wt	24,1258416	0,44766706	23,7060442	0,38639124	1,33773962	0,41979734	0,30506316	0,31833003	0,4971224	Unpaired t-te	0,480433962	permutation FDR (250 perm	1
171	Aldh9a1~Q9J	Aldh9a1	ko vs wt	ko	wt	28,1777921	1,13484686	26,7846778	0,09435239	2,6264504	1,39311434	0,83993784	0,12290292	0,91043781	Unpaired t-te	0,09594582	permutation FDR (250 perm	1
172	Aldoa~P050€	Aldoa	ko vs wt	ko	wt	32,7978664	0,24384622	32,6532347	0,27203467	1,10544843	0,14463173	0,11679172	0,57709086	0,23875581	Unpaired t-te	0,79575007	permutation FDR (250 perm	1
173	Aldob~Q91Y!	Aldob	ko vs wt	ko	wt	27,8121965	0,80651366	27,3214508	0,36460402	1,40517096	0,49074567	0,32027792	0,40335923	0,394308	Unpaired t-te	0,459904732	permutation FDR (250 perm	1
174	Alg2~Q9DBE	Alg2	ko vs wt	ko	wt	26,3184637	1,02211578	25,4791538	1,17890994	1,78919404	0,83930986	0,41522256	0,45892675	0,33825663	Unpaired t-te	0,351963278	permutation FDR (250 perm	1
175	Alg5~Q9DB2	Alg5	ko vs wt	ko	wt	27,798083	0,40147438	26,8231987	0,77904561	1,96548352	0,97488427	0,61019962	0,2107787	0,67617329	Unpaired t-te	0,20254657	permutation FDR (250 perm	1
176	Alox12~P396	Alox12	ko vs wt	ko	wt	26,9321716	3,11078865	25,5325625	1,78087306	2,63830077	1,39960904	0,43826866	0,55205359	0,25801876	Unpaired t-te	0,318881274	permutation FDR (250 perm	1
177	Alox5~P4899	Alox5	ko vs wt	ko	wt	24,7151658	0,44768325	25,1272466	0,58862981	0,75153865	-0,4120808	-0,2765739	0,45400202	0,34294222	Unpaired t-te	0,528640294	permutation FDR (250 perm	1
178	Alpl~P09242	Alpl	ko vs wt	ko	wt	26,7533873	0,83552333	28,2673202	0,93734108	0,35015536	-1,5139329	-0,8319417	0,13954437	0,85528768	Unpaired t-te	0,090555977	permutation FDR (250 perm	1
179	Alyref~O085	Alyref	ko vs wt	ko	wt	27,8821547	0,59221706	27,4342448	1,24559097	1,36406265	0,44790991	0,23031103	0,67225031	0,17246899	Unpaired t-te	0,60743432	permutation FDR (250 perm	1
180	Ambp~Q074	Ambp	ko vs wt	ko	wt	28,9988212	0,81352084	29,3383908	0,82889311	0,79027705	-0,3395696	-0,1939189	0,67341413	0,17171777	Unpaired t-te	0,656477855	permutation FDR (250 perm	1
181	Ampd2~Q9D	Ampd2	ko vs wt	ko	wt	26,8464393	0,22683223	24,4790245	1,79345397	5,16015655	2,36741483	1,04066415	0,20179007	0,69510021	Unpaired t-te	0,063716535	permutation FDR (250 perm	1
182	Ampd3~O08	Ampd3	ko vs wt	ko	wt	27,0914648	0,1611468	27,3912526	0,06140253	0,81237191	-0,2997877	-0,2718743	0,04130741	1,38397208	Unpaired t-te	0,530789216	permutation FDR (250 perm	1
183	Anapc1~P53!	Anapc1	ko vs wt	ko	wt	26,3334352	0,3311441	26,5215454	0,14647806	0,87775479	-0,1881101	-0,1545129	0,43117806	0,36534335	Unpaired t-te	0,724303668	permutation FDR (250 perm	1
184	Anapc2~Q8B	Anapc2	ko vs wt	ko	wt	23,9362169	0,24083063	24,8448796	0,75182228	0,53267861	-0,9086628	-0,5864228	0,2247913	0,6482205	Unpaired t-te	0,226626308	permutation FDR (250 perm	1
185	Anapc4~Q91	Anapc4	ko vs wt	ko	wt	25,1671458	0,60639147	23,9569857	0,35604646	2,31363314	1,21016012	0,84554167	0,03832226	1,4165489	Unpaired t-te	0,080852106	permutation FDR (250 perm	1
186	Anapc7~Q9V	Anapc7	ko vs wt	ko	wt	24,5065611	0,16660091	24,6034918	1,15182483	0,93502007	-0,0969308	-0,0532551	0,91645596	0,0378884	Unpaired t-te	0,913802787	permutation FDR (250 perm	1
187	Angpt1~O08	Angpt1	ko vs wt	ko	wt	25,1841316	0,7256226	24,7287555	0,52741675	1,37114023	0,45537612	0,29174199	0,4540172	0,3429277	Unpaired t-te	0,497522565	permutation FDR (250 perm	1
188	Angptl2~Q9F	Angptl2	ko vs wt	ko	wt	30,2380414	0,40575456	29,4536641	0,64395577	1,72234882	0,78437736	0,51874315	0,22146235	0,65470009	Unpaired t-te	0,261349206	permutation FDR (250 perm	1
189	Angptl4~Q9Z	Angptl4	ko vs wt	ko	wt	24,6402719	0,44895199	24,0899969	0,19673086	1,46436486	0,55027505	0,42519451	0,12782833	0,8933729	Unpaired t-te	0,335723193	permutation FDR (250 perm	1

190	Angptl6~Q8I	Angptl6	ko vs wt	ko	wt	23,6318173	0,78229935	24,3831067	0,66527385	0,59407239	-0,7512894	-0,4547359	0,30434736	0,51663046	Unpaired t-t	0,303510862	permutation FDR (250 perm	1
191	Angptl8~Q8I	Angptl8	ko vs wt	ko	wt	24,8058842	0,4754679	24,448104	1,59966409	1,28145267	0,3577802	0,16533533	0,7848431	0,10521715	Unpaired t-t	0,72084738	permutation FDR (250 perm	1
192	Ank1~Q0235	Ank1	ko vs wt	ko	wt	32,0639819	0,63996384	31,7076167	0,51115397	1,28019641	0,35636517	0,23493417	0,52218047	0,28217937	Unpaired t-t	0,583607421	permutation FDR (250 perm	1
193	Ank2~Q8C8R	Ank2	ko vs wt	ko	wt	29,1784013	0,20416865	29,5764783	0,35658437	0,75886912	-0,398077	-0,3114024	0,25165925	0,59918709	Unpaired t-t	0,486609023	permutation FDR (250 perm	1
194	Ank3~S4R2K	Ank3	ko vs wt	ko	wt	26,1165904	0,42485208	26,4473728	0,66690008	0,79510515	-0,3307824	-0,215979	0,57680156	0,23897358	Unpaired t-t	0,626963792	permutation FDR (250 perm	1
195	Ankfy1~Q81(	Ankfy1	ko vs wt	ko	wt	28,3941877	0,70542956	27,6379592	0,18384992	1,6890693	0,75622852	0,52974812	0,15984943	0,79628891	Unpaired t-t	0,24663104	permutation FDR (250 perm	1
196	Ankhd1~E9PI	Ankhd1	ko vs wt	ko	wt	26,795847	0,22752362	26,7671961	0,36508869	1,02005778	0,02865088	0,02221591	0,92738043	0,03274207	Unpaired t-t	0,961939012	permutation FDR (250 perm	1
197	Ankle2~Q6P1	Ankle2	ko vs wt	ko	wt	26,3207594	0,12075121	26,8037263	0,34890944	0,71550466	-0,4829669	-0,3844124	0,18234047	0,73911692	Unpaired t-t	0,406142435	permutation FDR (250 perm	1
198	Ankmy2~Q3T	Ankmy2	ko vs wt	ko	wt	25,7005042	0,87216527	24,9790396	1,00980121	1,64885503	0,72146456	0,3850416	0,45705643	0,34003018	Unpaired t-t	0,385093209	permutation FDR (250 perm	1
199	Ankrd17~Q9	Ankrd17	ko vs wt	ko	wt	26,3567569	0,31219519	24,3981605	0,86588656	3,88683632	1,95859635	1,19553876	0,07495816	1,12518106	Unpaired t-t	0,035562116	permutation FDR (250 perm	1
200	Ankrd28~Q5(	Ankrd28	ko vs wt	ko	wt	24,1144389	0,5387007	24,2777193	1,58489033	0,89299224	-0,1632805	-0,0754864	0,89965466	0,04592417	Unpaired t-t	0,876932282	permutation FDR (250 perm	1
201	Ankrd35~E9(	Ankrd35	ko vs wt	ko	wt	25,1119999	0,68135017	23,9711245	0,53573324	2,20514789	1,14087541	0,73789402	0,09254263	1,03365816	Unpaired t-t	0,120248668	permutation FDR (250 perm	1
202	Ankrd44~B2I	Ankrd44	ko vs wt	ko	wt	25,2833607	1,31611159	24,4903534	1,47286545	1,73268242	0,79300725	0,34641163	0,57214914	0,24249075	Unpaired t-t	0,430333549	permutation FDR (250 perm	1
203	Anln~Q8K29	Anln	ko vs wt	ko	wt	25,4028913	0,36233503	24,8770338	0,83964276	1,43978915	0,52585755	0,32271225	0,47572647	0,32264268	Unpaired t-t	0,47729808	permutation FDR (250 perm	1
204	Ano10~Q8BI	Ano10	ko vs wt	ko	wt	28,0274436	0,4122678	27,57358	0,26652576	1,36970339	0,45386351	0,34816166	0,19521903	0,70947786	Unpaired t-t	0,4219987	permutation FDR (250 perm	1
205	Ano6~Q6P9J	Ano6	ko vs wt	ko	wt	28,2144408	0,36418584	28,0463622	0,86546022	1,12356115	0,16807865	0,10204603	0,81510509	0,08878639	Unpaired t-t	0,829011583	permutation FDR (250 perm	1
206	Anp32b~Q9E	Anp32b	ko vs wt	ko	wt	27,8707721	0,32018183	25,3276593	2,01906321	5,82845192	2,54311274	1,04242545	0,21524379	0,66706936	Unpaired t-t	0,062794521	permutation FDR (250 perm	1
207	Anpep~P974	Anpep	ko vs wt	ko	wt	31,1167792	0,32109137	30,6934955	0,39051528	1,34097628	0,42328371	0,31763932	0,27709084	0,55737783	Unpaired t-t	0,468926968	permutation FDR (250 perm	1
208	Anxa11~P97	Anxa11	ko vs wt	ko	wt	32,4901356	0,33710901	32,7222166	0,36074414	0,85140588	-0,232081	-0,1757049	0,50874663	0,29349846	Unpaired t-t	0,687947081	permutation FDR (250 perm	1
209	Anxa1~P101(	Anxa1	ko vs wt	ko	wt	33,4339865	0,39604461	33,5312796	0,32758783	0,93478533	-0,097293	-0,0734018	0,77757431	0,1092581	Unpaired t-t	0,874301907	permutation FDR (250 perm	1
210	Anxa2~P073	Anxa2	ko vs wt	ko	wt	34,4030733	0,17320264	34,3863368	0,31649404	1,01166841	0,0167365	0,01344167	0,95013785	0,02221338	Unpaired t-t	0,977120293	permutation FDR (250 perm	1
211	Anxa3~O356	Anxa3	ko vs wt	ko	wt	28,6781177	1,91645805	29,5780026	0,9825117	0,5359295	-0,8998849	-0,3901521	0,52328356	0,28126291	Unpaired t-t	0,373405369	permutation FDR (250 perm	1
212	Anxa4~P974	Anxa4	ko vs wt	ko	wt	31,027034	0,325433	30,8986344	0,43397312	1,09308044	0,12839957	0,09442413	0,74190146	0,12965378	Unpaired t-t	0,838428178	permutation FDR (250 perm	1
213	Anxa5~P480	Anxa5	ko vs wt	ko	wt	32,2206556	0,34121556	32,4260237	0,27609566	0,86731734	-0,2053681	-0,1607766	0,49350586	0,30670768	Unpaired t-t	0,712670289	permutation FDR (250 perm	1
214	Anxa6~P148	Anxa6	ko vs wt	ko	wt	34,2109646	0,2310529	34,1223211	0,14833896	1,06336991	0,08864355	0,07578329	0,62375694	0,20498461	Unpaired t-t	0,869262756	permutation FDR (250 perm	1
215	Anxa7~Q070	Anxa7	ko vs wt	ko	wt	32,1235893	0,37621114	32,2039648	0,2083765	0,94581141	-0,0803756	-0,0636655	0,77212514	0,1123123	Unpaired t-t	0,889360713	permutation FDR (250 perm	1
216	Anxa8~O356	Anxa8	ko vs wt	ko	wt	28,4352815	0,51289115	27,6295821	1,35935895	1,74799293	0,80569935	0,40168673	0,49516299	0,30525182	Unpaired t-t	0,38591287	permutation FDR (250 perm	1
217	Aoc1l3~Q6W	Aoc1l3	ko vs wt	ko	wt	29,3348166	4,16555453	28,3090803	2,36237703	2,03599817	1,02573626	0,26112113	0,74070851	0,13035266	Unpaired t-t	0,543547837	permutation FDR (250 perm	1
218	Aoc3~O7042	Aoc3	ko vs wt	ko	wt	32,9478256	0,55894885	33,4176677	0,23189539	0,72204366	-0,469842	-0,34497	0,2592981	0,58620066	Unpaired t-t	0,429665697	permutation FDR (250 perm	1
219	Aox1~G3X8P	Aox1	ko vs wt	ko	wt	23,8341098	0,96763526	25,2306878	0,95866112	0,37982901	-1,396578	-0,7434855	0,18191086	0,74014137	Unpaired t-t	0,121170948	permutation FDR (250 perm	1
220	Ap1b1~O356	Ap1b1	ko vs wt	ko	wt	29,2394196	0,30820517	29,1018843	0,24339962	1,10002423	0,13753531	0,11024358	0,60311004	0,21960344	Unpaired t-t	0,805513904	permutation FDR (250 perm	1
221	Ap1g1~P228	Ap1g1	ko vs wt	ko	wt	28,32722	0,34718413	27,4933273	0,64687088	1,78248837	0,83389267	0,55615107	0,20091815	0,69698084	Unpaired t-t	0,237453067	permutation FDR (250 perm	1
222	Ap1m1~P35	Ap1m1	ko vs wt	ko	wt	27,9036496	0,35327587	27,4059963	0,65598767	1,41191502	0,49765325	0,33029	0,40363732	0,39400868	Unpaired t-t	0,462336609	permutation FDR (250 perm	1
223	Ap2a1~P174	Ap2a1	ko vs wt	ko	wt	31,4966929	0,26678443	31,3361073	0,26488427	1,11774075	0,16058561	0,1292439	0,54160449	0,26631774	Unpaired t-t	0,770877173	permutation FDR (250 perm	1
224	Ap2a2~P174	Ap2a2	ko vs wt	ko	wt	31,5491804	0,1753179	31,330687	0,15207892	1,16351797	0,21849349	0,19037836	0,20307959	0,69233372	Unpaired t-t	0,660963912	permutation FDR (250 perm	1
225	Ap2b1~Q9DI	Ap2b1	ko vs wt	ko	wt	32,7103186	0,05309468	32,6627851	0,16393699	1,03349649	0,0475335	0,0424442	0,7258398	0,13915922	Unpaired t-t	0,930152781	permutation FDR (250 perm	1
226	Ap2m1~P84(	Ap2m1	ko vs wt	ko	wt	31,1975922	0,15614269	31,518452	0,37542949	0,80059258	-0,3208598	-0,2506017	0,35129495	0,4543281	Unpaired t-t	0,578566287	permutation FDR (250 perm	1
227	Ap2s1~P627	Ap2s1	ko vs wt	ko	wt	28,4532225	0,58363562	27,4523238	0,5352486	2,00124629	1,00089873	0,66427941	0,11115707	0,9540629	Unpaired t-t	0,154452195	permutation FDR (250 perm	1
228	Ap3b1~Q9Z1	Ap3b1	ko vs wt	ko	wt	30,3328587	0,50663365	30,1599722	0,35863058	1,12731167	0,17288643	0,12463636	0,67391359	0,17139578	Unpaired t-t	0,777288553	permutation FDR (250 perm	1
229	Ap3d1~O547	Ap3d1	ko vs wt	ko	wt	30,8403944	0,25514298	30,7536836	0,40587953	1,06194631	0,08671083	0,06556107	0,80524278	0,09407316	Unpaired t-t	0,889415834	permutation FDR (250 perm	1
230	Ap3m1~Q9JI	Ap3m1	ko vs wt	ko	wt	29,3851748	0,2946219	29,5325932	0,33073614	0,90286464	-0,1474184	-0,1143502	0,63743104	0,19556679	Unpaired t-t	0,800922219	permutation FDR (250 perm	1
231	Ap3m2~Q8R	Ap3m2	ko vs wt	ko	wt	25,1429228	0,87664175	24,885264	1,15446782	1,19553703	0,25765881	0,13142489	0,8035993	0,09496045	Unpaired t-t	0,769927089	permutation FDR (250 perm	1
232	Ap3s1~Q9DC	Ap3s1	ko vs wt	ko	wt	25,5179672	0,88913315	24,0999361	0,31566518	2,67220582	1,41803113	0,90912885	0,064049	1,19348761	Unpaired t-t	0,068819302	permutation FDR (250 perm	1
233	Ap4m1~Q9JI	Ap4m1	ko vs wt	ko	wt	25,0567165	0,70674873	23,7749545	0,23675647	2,43135734	1,28176195	0,88946415	0,04482127	1,34851586	Unpaired t-t	0,074355065	permutation FDR (250 perm	1
234	Ap5z1~A0A0	Ap5z1	ko vs wt	ko	wt	24,2426431	0,8888562	23,9274273	0,12636346	1,24419776	0,31521582	0,20725583	0,58555603	0,23243154	Unpaired t-t	0,640058548	permutation FDR (250 perm	1
235	Apaf1~O888	Apaf1	ko vs wt	ko	wt	23,7079003	0,83684427	25,4586218	0,77458718	0,29715313	-1,7507215	-1,011766	0,06741979	1,17121258	Unpaired t-t	0,044811156	permutation FDR (250 perm	1
236	Apcs~P1224	Apcs	ko vs wt	ko	wt	24,5512359	1,10563992	25,7431349	2,19401676	0,43772632	-1,191899	-0,4451374	0,53414612	0,27233992	Unpaired t-t	0,334385724	permutation FDR (250 perm	1
237	Apeh~Q8R14	Apeh	ko vs wt	ko	wt	24,2062006	0,81301646	23,5957258	0,7777225	1,52676161	0,61047482	0,35430511	0,44182317	0,35475151	Unpaired t-t	0,417167427	permutation FDR (250 perm	1
238	Apex1~P283	Apex1	ko vs wt	ko	wt	27,7868243	0,14051666	27,2467428	0,46403072	1,4540546	0,54008144	0,40364835	0,23756007	0,62422655	Unpaired t-t	0,386821262	permutation FDR (250 perm	1
239	Api5~O3584	Api5	ko vs wt	ko	wt	28,2319903	0,21815503	28,9329717	0,21096058	0,61515361	-0,7009814	-0,5864808	0,01965533	1,70651963	Unpaired t-t	0,199912375	permutation FDR (250 perm	1
240	Apip~Q9WV(	Apip	ko vs wt	ko	wt	24,8495903	1,30441389	24,9690792	1,89526266	0,92051367	-0,119489	-0,0470937	0,9425899	0,02567722	Unpaired t-t	0,920652232	permutation FDR (250 perm	1
241	Apmap~Q9D	Apmap	ko vs wt	ko	wt	27,6901398	0,05487824	27,9301359	0,54144328	0,84674757	-0,2399962	-0,1733867	0,59517055	0,22535857	Unpaired t-t	0,710529025	permutation FDR (250 perm	1
242	Apoa1~Q006	Apoa1	ko vs wt	ko	wt	32,0268887	1,01084553	30,4108792	0,19682557	3,06526023	1,61600956	1,01002015	0,06618487	1,1792413	Unpaired t-t	0,052230168	permutation FDR (250 perm	1
243	Apoa2~P098	Apoa2	ko vs wt	ko	wt	27,5744818	1,33266235	27,5878998	0,25332685	0,99074251	-0,0134179	-0,0074961	0,98741887	0,00549858	Unpaired t-t	0,986681261	permutation FDR (250 perm	1
244	Apoa4~P067	Apoa4	ko vs wt	ko	wt	32,3760895	0,85653617	31,4779827	0,3423937	1,86361888	0,89810685	0,57919693	0,17408558	0,7592372	Unpaired t-t	0,205855449	permutation FDR (250 perm	1
245	Apoa5~Q8C7	Apoa5	ko vs wt	ko	wt	25,4478954	1,52145044	24,4608294	1,18342786	1,9821498	0,987066	0,44599039	0,45369844	0,34323272	Unpaired t-t	0,311574765	permutation FDR (250 perm	1
246	Apob~E9Q41	Apob	ko vs wt	ko	wt	33,9138707	0,98744482	32,8240962	0,25988189	2,12840777	1,08977458	0,68154074	0,15094922	0,82116913	Unpaired t-t	0,152761826	permutation FDR (250 perm	1
247	Apoc1~P349	Apoc1	ko vs wt	ko	wt	25,2266717	1,80368866	25,3491937	2,60709854	0,91858043	-0,122522	-0,039304	0,95722244	0,01898738	Unpaired t-t	0,932883338	permutation FDR (250 perm	1
248	Apoc2~Q050	Apoc2	ko vs wt	ko	wt	24,0554115	0,83842411	25,5035948	0,1868112	0,36648262	-1,4481833	-0,9643212	0,0537129	1,2699214	Unpaired t-t	0,060211652	permutation FDR (250 perm	1
249	Apoc3~E9QP	Apoc3	ko vs wt	ko	wt	26,5015385	2,19738705	25,3378988	1,02022388	2,24021892	1,16363972	0,47313419	0,46488361	0,33265577	Unpaired t-t	0,285450953	permutation FDR (250 perm	1
250	Apof~Q91V8	Apof	ko vs wt	ko	wt	27,3968756	0,36452299	27,2918677	0,81256476	1,07550025	0,10500786	0,06514535	0,87646988	0,057263	Unpaired t-t	0,891828247	permutation FDR (250 perm	1
251	Apoh~Q0133	Apoh	ko vs wt	ko	wt	28,5862272	0,60092794	27,3267026	0,12469772	2,39416833	1,25952459	0,92750193	0,03221663	1,49191992	Unpaired t-t	0,069230138	permutation FDR (250 perm	1
252	Apool~Q78IK	Apool	ko vs wt	ko	wt	27,0075113	0,78672675	28,3311685	0,09218536	0,39952088	-1,3236572	-0,9073165	0,06031357	1,21958499	Unpaired t-t	0,076266799	permutation FDR (250 perm	1
253	Appl1~Q8K3I	Appl1	ko vs wt	ko	wt	27,9903139	0,52057591	28,607624	0,05028309	0,65188526	-0,61731	-0,4738877	0,13160904	0,88071429	Unpaired t-t	0,299586255	permutation FDR (250 perm	1

254	App~P12023	App	ko vs wt	ko	wt	27,2252439	0,28817288	28,1661452	0,63253425	0,52090733	-0,9409014	-0,6369442	0,15882668	0,79907654	Unpaired t-te	0,1896212	permutation FDR (250 perm		1
255	Aprt~P08030	Aprt	ko vs wt	ko	wt	27,508957	0,28009242	28,099829	0,49949534	0,66394149	-0,590872	-0,4255603	0,23044361	0,63743533	Unpaired t-te	0,352840245	permutation FDR (250 perm		1
256	Aqr~Q8CFQ3	Aqr	ko vs wt	ko	wt	26,4148237	0,66338849	26,1734395	0,0111441	1,18212634	0,24138423	0,1745255	0,57331767	0,24160467	Unpaired t-te	0,697033295	permutation FDR (250 perm		1
257	Araf~P04627	Araf	ko vs wt	ko	wt	24,3002691	1,11367505	23,7645547	0,97975993	1,44965982	0,5357144	0,27540431	0,59719506	0,22388379	Unpaired t-te	0,522538084	permutation FDR (250 perm		1
258	Arap1~Q4LD	Arap1	ko vs wt	ko	wt	26,8816805	1,14220847	24,4730247	2,02438913	5,30979338	2,40865572	0,93501646	0,22843957	0,64122866	Unpaired t-te	0,072783443	permutation FDR (250 perm		1
259	Arap3~Q8R5	Arap3	ko vs wt	ko	wt	23,6905701	0,681223	24,0665018	0,69788647	0,77060759	-0,3759317	-0,2304859	0,58188031	0,23516634	Unpaired t-te	0,59304881	permutation FDR (250 perm		1
260	Arcn1~Q5XJY	Arcn1	ko vs wt	ko	wt	30,266229	0,15653532	30,3218242	0,23974665	0,96219738	-0,0555952	-0,0466359	0,79040139	0,1021523	Unpaired t-te	0,921579904	permutation FDR (250 perm		1
261	Arf3~P61205	Arf3	ko vs wt	ko	wt	28,0553146	1,17070645	26,4062626	0,83651985	3,13627484	1,64905199	0,86875259	0,1260715	0,89938307	Unpaired t-te	0,07440321	permutation FDR (250 perm		1
262	Arf4~P61750	Arf4	ko vs wt	ko	wt	27,5772354	0,63920883	27,6181153	0,3067262	0,97206192	-0,0408799	-0,0286261	0,92793158	0,03248405	Unpaired t-te	0,94939773	permutation FDR (250 perm		1
263	Arf6~P62331	Arf6	ko vs wt	ko	wt	26,8525228	0,49607222	26,5253033	0,1378898	1,25459303	0,32721946	0,25121466	0,34547911	0,46157821	Unpaired t-te	0,56475581	permutation FDR (250 perm		1
264	Arfgap2~Q9	Arfgap2	ko vs wt	ko	wt	25,5556682	1,28504451	26,823598	0,01056246	0,41525524	-1,2679297	-0,727876	0,1859843	0,73052372	Unpaired t-te	0,138699873	permutation FDR (250 perm		1
265	Arfgap3~Q9	Arfgap3	ko vs wt	ko	wt	27,4318783	0,30695621	27,4459221	0,86374313	0,99031281	-0,0140438	-0,0085845	0,98412377	0,00695028	Unpaired t-te	0,985603129	permutation FDR (250 perm		1
266	Arfgef1~G3X	Arfgef1	ko vs wt	ko	wt	24,741215	1,01245808	25,2290921	0,40630818	0,71307362	-0,4878771	-0,2954445	0,49314648	0,30702406	Unpaired t-te	0,496036137	permutation FDR (250 perm		1
267	Arfgef2~A2A	Arfgef2	ko vs wt	ko	wt	27,8343421	0,45634253	26,8714286	1,02303942	1,94924231	0,96291344	0,54405462	0,314016	0,50304823	Unpaired t-te	0,250347794	permutation FDR (250 perm		1
268	Arfip1~G5E8	Arfip1	ko vs wt	ko	wt	28,9100905	0,22022465	28,4988826	0,08298623	1,32979875	0,41120793	0,3606978	0,0408615	1,38868575	Unpaired t-te	0,410830991	permutation FDR (250 perm		1
269	Arg1~Q6117	Arg1	ko vs wt	ko	wt	25,8558452	2,31831618	23,972149	0,3281575	3,6901927	1,88369616	0,7987025	0,25481942	0,59376748	Unpaired t-te	0,108761905	permutation FDR (250 perm		1
270	Arglu1~Q3U	Arglu1	ko vs wt	ko	wt	27,0313086	0,10578261	27,1773212	0,52306775	0,90374485	-0,1460126	-0,1062008	0,73273552	0,13505275	Unpaired t-te	0,823920508	permutation FDR (250 perm		1
271	Arhgap10~Q	Arhgap10	ko vs wt	ko	wt	27,766549	0,61562322	27,8309614	0,26699849	0,9563348	-0,0644123	-0,0459281	0,87991878	0,05555741	Unpaired t-te	0,920789952	permutation FDR (250 perm		1
272	Arhgap17~Q	Arhgap17	ko vs wt	ko	wt	28,3193021	0,30476978	28,1424181	0,27962164	1,13043966	0,17688399	0,13986459	0,53648059	0,27044599	Unpaired t-te	0,750089653	permutation FDR (250 perm		1
273	Arhgap1~Q5	Arhgap1	ko vs wt	ko	wt	29,7165832	0,41120291	29,6457033	0,34595229	1,05035713	0,07087993	0,05286047	0,84392346	0,07369694	Unpaired t-te	0,908875638	permutation FDR (250 perm		1
274	Arhgap23~Q	Arhgap23	ko vs wt	ko	wt	24,1914615	0,75058899	24,5495336	0,41642844	0,78020651	-0,3580721	-0,2349665	0,52556218	0,2793759	Unpaired t-te	0,583360862	permutation FDR (250 perm		1
275	Arhgap24~Q	Arhgap24	ko vs wt	ko	wt	24,6223067	0,71239602	24,8429965	0,74354501	0,85815499	-0,2206899	-0,1323452	0,75693195	0,12094316	Unpaired t-te	0,765686517	permutation FDR (250 perm		1
276	Arhgap25~Q	Arhgap25	ko vs wt	ko	wt	26,5201933	0,28917918	25,2405124	0,9269168	2,42785267	1,27968088	0,76336898	0,18411574	0,73490908	Unpaired t-te	0,137214953	permutation FDR (250 perm		1
277	Arhgap27~A	Arhgap27	ko vs wt	ko	wt	24,0099876	0,19495545	24,6642735	0,49783852	0,63538993	-0,6542859	-0,4777267	0,19666091	0,70628196	Unpaired t-te	0,308351832	permutation FDR (250 perm		1
278	Arhgap35~Q	Arhgap35	ko vs wt	ko	wt	26,3976893	0,23615401	26,7320605	0,28390231	0,79312978	-0,3343711	-0,2690743	0,24492872	0,61096028	Unpaired t-te	0,538207149	permutation FDR (250 perm		1
279	Arhgap45~Q	Arhgap45	ko vs wt	ko	wt	27,4609274	1,3785541	26,113813	0,03273865	2,5440277	1,34711438	0,74996131	0,18910131	0,72330545	Unpaired t-te	0,130006914	permutation FDR (250 perm		1
280	Arhgap5~P9	Arhgap5	ko vs wt	ko	wt	26,9147016	0,3172768	27,1496647	0,12836334	0,8497067	-0,2349632	-0,1950817	0,31072073	0,50762978	Unpaired t-te	0,653939959	permutation FDR (250 perm		1
281	Arhgap6~O5	Arhgap6	ko vs wt	ko	wt	25,1418042	0,66742152	24,3796487	1,14908099	1,69602272	0,76215549	0,40128972	0,46085037	0,33644006	Unpaired t-te	0,377579652	permutation FDR (250 perm		1
282	Arhgdia~Q9	Arhgdia	ko vs wt	ko	wt	29,7717914	0,728188	30,2097015	0,80976183	0,7382032	-0,4379101	-0,256034	0,57119113	0,24321854	Unpaired t-te	0,555665657	permutation FDR (250 perm		1
283	Arhgdib~Q61	Arhgdib	ko vs wt	ko	wt	29,2134194	1,00053516	27,3001246	0,30699239	3,76668334	1,91329475	1,18316418	0,03888282	1,4102423	Unpaired t-te	0,025986486	permutation FDR (250 perm		1
284	Arhgef10l~A	Arhgef10l	ko vs wt	ko	wt	26,3580062	0,33507059	26,0353992	0,20181811	1,2505884	0,32260704	0,26008428	0,23782678	0,62373924	Unpaired t-te	0,544834094	permutation FDR (250 perm		1
285	Arhgef10~Q	Arhgef10	ko vs wt	ko	wt	25,5748793	0,98777932	24,8406482	0,73918903	1,66351057	0,73423103	0,41398093	0,38668961	0,4126375	Unpaired t-te	0,344879046	permutation FDR (250 perm		1
286	Arhgef11~Q	Arhgef11	ko vs wt	ko	wt	25,1871061	0,27307761	25,2649801	0,29179034	0,9474528	-0,077874	-0,061821	0,77888741	0,10852531	Unpaired t-te	0,893036957	permutation FDR (250 perm		1
287	Arhgef12~Q	Arhgef12	ko vs wt	ko	wt	28,5872294	0,47020804	29,3275452	0,31511253	0,59860834	-0,7403157	-0,5478921	0,08885772	1,05130484	Unpaired t-te	0,222498314	permutation FDR (250 perm		1
288	Arhgef17~Q	Arhgef17	ko vs wt	ko	wt	29,7311717	0,44253293	30,4439472	0,21067319	0,61014522	-0,7127754	-0,5500857	0,06536006	1,18468756	Unpaired t-te	0,223243729	permutation FDR (250 perm		1
289	Arhgef18~Q	Arhgef18	ko vs wt	ko	wt	25,5606047	0,04382937	25,8169905	0,29190437	0,83718256	-0,2563858	-0,2122482	0,3397396	0,46885383	Unpaired t-te	0,646288051	permutation FDR (250 perm		1
290	Arhgef1~Q6	Arhgef1	ko vs wt	ko	wt	28,604719	0,42555578	28,6380257	0,25108435	0,97717797	-0,0333068	-0,0255591	0,91683691	0,03770791	Unpaired t-te	0,954114301	permutation FDR (250 perm		1
291	Arhgef25~Q	Arhgef25	ko vs wt	ko	wt	25,8893843	0,29084096	24,4204223	1,16148391	2,76822656	1,46896202	0,79909455	0,21198268	0,67369963	Unpaired t-te	0,125718764	permutation FDR (250 perm		1
292	Arhgef2~Q6	Arhgef2	ko vs wt	ko	wt	29,9224126	0,31322268	29,8333562	0,66143161	1,06367425	0,08905639	0,05931372	0,87193313	0,05951682	Unpaired t-te	0,901478331	permutation FDR (250 perm		1
293	Arhgef40~Q	Arhgef40	ko vs wt	ko	wt	26,1337525	0,33867812	24,8122462	0,93065947	2,49926907	1,32150623	0,78357388	0,17449371	0,75822021	Unpaired t-te	0,127607298	permutation FDR (250 perm		1
294	Arhgef5~E9C	Arhgef5	ko vs wt	ko	wt	26,2661608	0,40075088	25,7903253	1,19527262	1,3907234	0,47583551	0,25360539	0,63515295	0,19712168	Unpaired t-te	0,577163112	permutation FDR (250 perm		1
295	Arhgef6~Q8	Arhgef6	ko vs wt	ko	wt	28,0000252	0,39738972	27,8636442	0,56749555	1,09914441	0,13638095	0,09326856	0,78562783	0,10478314	Unpaired t-te	0,840529816	permutation FDR (250 perm		1
296	Arhgef7~Q9	Arhgef7	ko vs wt	ko	wt	28,6756223	0,39321641	28,2223926	0,5325784	1,36910183	0,45322975	0,31480262	0,37003132	0,43176152	Unpaired t-te	0,475632126	permutation FDR (250 perm		1
297	Arid1b~E9Q	Arid1b	ko vs wt	ko	wt	26,5091579	0,27445006	26,9085005	0,46952266	0,75820373	-0,3993425	-0,2919433	0,35922981	0,44462763	Unpaired t-te	0,513370828	permutation FDR (250 perm		1
298	Arih1~Q9Z1	Arih1	ko vs wt	ko	wt	28,5378751	0,2862589	29,171041	0,31889205	0,64475995	-0,633166	-0,4948262	0,08684042	1,06127809	Unpaired t-te	0,270760644	permutation FDR (250 perm		1
299	Arl15~Q8BG	Arl15	ko vs wt	ko	wt	24,6841847	0,33583414	24,408189	0,88251261	1,21082954	0,27599578	0,16692021	0,70779058	0,15009522	Unpaired t-te	0,716737653	permutation FDR (250 perm		1
300	Arl1~P61211	Arl1	ko vs wt	ko	wt	25,8541578	0,18141863	26,0403125	0,11161073	0,87894488	-0,1861554	-0,1645723	0,21532813	0,66689924	Unpaired t-te	0,704632302	permutation FDR (250 perm		1
301	Arl3~Q9WUL	Arl3	ko vs wt	ko	wt	26,3433816	0,45602458	24,4419948	0,40324888	3,73572133	1,90138684	1,36977314	0,00552032	2,25803541	Unpaired t-te	0,012010144	permutation FDR (250 perm		1
302	Arl8a~Q8VE	Arl8a	ko vs wt	ko	wt	27,5941156	0,83634407	27,5686124	0,57010579	1,01783468	0,02550326	0,01565557	0,96922901	0,01357359	Unpaired t-te	0,972106834	permutation FDR (250 perm		1
303	Armc10~Q9D	Armc10	ko vs wt	ko	wt	24,9569559	0,89354273	24,4419952	1,20344753	1,42895521	0,51496069	0,25810892	0,63636049	0,1962968	Unpaired t-te	0,556562084	permutation FDR (250 perm		1
304	Armc1~Q9D	Armc1	ko vs wt	ko	wt	24,6177451	1,03339724	24,7685247	1,25072872	0,90076357	-0,1507796	-0,072952	0,89492377	0,04821396	Unpaired t-te	0,876560544	permutation FDR (250 perm		1
305	Armc6~Q8B	Armc6	ko vs wt	ko	wt	25,1513943	1,73306294	24,3688758	0,59193065	1,72013102	0,78251846	0,37538039	0,51091307	0,29165298	Unpaired t-te	0,395165401	permutation FDR (250 perm		1
306	Armc8~Q9D	Armc8	ko vs wt	ko	wt	25,8601655	0,18518838	25,4550783	0,22079089	1,32416886	0,40508711	0,34063133	0,10330874	0,98586294	Unpaired t-te	0,438245501	permutation FDR (250 perm		1
307	Arpc1a~Q9R	Arpc1a	ko vs wt	ko	wt	26,4480557	0,60242453	24,9623051	0,84946561	2,8006285	1,48575062	0,87701737	0,11312213	0,94645242	Unpaired t-te	0,082465887	permutation FDR (250 perm		1
308	Arpc1b~Q9W	Arpc1b	ko vs wt	ko	wt	31,9673894	0,12256326	31,5940794	0,30663807	1,29532129	0,37330999	0,30397844	0,22095654	0,65569315	Unpaired t-te	0,50372067	permutation FDR (250 perm		1
309	Arpc2~Q9CV	Arpc2	ko vs wt	ko	wt	31,3495739	0,17920653	31,1510463	0,10119687	1,14752662	0,19852762	0,17634375	0,17677599	0,75257673	Unpaired t-te	0,683885846	permutation FDR (250 perm		1
310	Arpc3~Q9JM	Arpc3	ko vs wt	ko	wt	30,3873081	0,23509041	30,2572815	0,02959014	1,09431387	0,13002658	0,11432589	0,41072294	0,38645104	Unpaired t-te	0,803841915	permutation FDR (250 perm		1
311	Arpc4~P5999	Arpc4	ko vs wt	ko	wt	31,0023055	0,26766139	30,6476249	0,46071618	1,27870244	0,35468058	0,26068533	0,39998985	0,39795103	Unpaired t-te	0,557849879	permutation FDR (250 perm		1
312	Arpc5l~Q9D8	Arpc5l	ko vs wt	ko	wt	24,9445899	1,16176979	24,7019693	0,25454563	1,18313989	0,24262067	0,14318313	0,74724168	0,12653891	Unpaired t-te	0,748432036	permutation FDR (250 perm		1
313	Arpc5~Q9CP	Arpc5	ko vs wt	ko	wt	28,3217582	0,90040104	27,9464682	0,28611408	1,29710033	0,37529008	0,24090609	0,53935283	0,26812704	Unpaired t-te	0,579270003	permutation FDR (250 perm		1
314	Arrb1~Q8BW	Arrb1	ko vs wt	ko	wt	27,3584055	0,79603241	27,6307012	0,57794698	0,82800092	-0,2722957	-0,1686034	0,67657094	0,16968666	Unpaired t-te	0,697508611	permutation FDR (250 perm		1
315	Arrb2~Q91Y	Arrb2	ko vs wt	ko	wt	26,3612341	1,3338991	24,8280404	0,70848918	2,89425826	1,53319366	0,79906482	0,15859061	0,79972254	Unpaired t-te	0,096091333	permutation FDR (250 perm		1
316	Arrdc1~Q99	Arrdc1	ko vs wt	ko	wt	24,4108741	0,6704252	24,4198374	0,75727242	0,99380633	-0,0089634	-0,0053973	0,98983813	0,00443582	Unpaired t-te	0,990311898	permutation FDR (250 perm		1
317	Art3~Q8R2G	Art3	ko vs wt	ko	wt	24,6607021	0,71427594	26,6503171	0,48439761	0,25180608	-1,989615	-1,2952531	0,01384186	1,85880566	Unpaired t-te	0,014812352	permutation FDR (250 perm		1

318	Asah1~Q9W	Asah1	ko vs wt	ko	wt	27,8337982	0,6894261	27,1331152	0,16880303	1,62527407	0,70068302	0,4949893	0,17668011	0,75281235	Unpaired t-t	0,27443503	permutation FDR (250 perm	1
319	Asap1~Q9QV	Asap1	ko vs wt	ko	wt	26,5143835	0,24715023	26,5677728	0,44700547	0,96366976	-0,0533893	-0,0396417	0,88799912	0,05158747	Unpaired t-t	0,9330768	permutation FDR (250 perm	1
320	Asap2~Q7SI	Asap2	ko vs wt	ko	wt	28,5363733	0,16924529	28,8686155	0,22254825	0,79430106	-0,3322422	-0,2803176	0,15763812	0,80233876	Unpaired t-t	0,522964373	permutation FDR (250 perm	1
321	Ascc1~Q9D8	Ascc1	ko vs wt	ko	wt	24,9421639	0,56266761	25,0760236	0,17849077	0,91138992	-0,1338597	-0,0992647	0,72121494	0,14193528	Unpaired t-t	0,827641379	permutation FDR (250 perm	1
322	Ascc3~E9PZJ	Ascc3	ko vs wt	ko	wt	27,3740511	0,31274467	27,2713259	0,45787355	1,07379993	0,10272522	0,07494298	0,79850354	0,09772315	Unpaired t-t	0,874517429	permutation FDR (250 perm	1
323	Asph~Q8BSY	Asph	ko vs wt	ko	wt	31,3273765	0,20706827	31,3360282	0,34176036	0,99402106	-0,0086517	-0,0068144	0,97642939	0,01035915	Unpaired t-t	0,987689502	permutation FDR (250 perm	1
324	Aspn~Q99M	Aspn	ko vs wt	ko	wt	34,8897885	0,28859769	35,3499081	0,21048999	0,726926	-0,4601196	-0,3760933	0,09494896	1,02250977	Unpaired t-t	0,38783007	permutation FDR (250 perm	1
325	Asrgl1~Q8C	Asrgl1	ko vs wt	ko	wt	25,1132811	0,38529053	24,1447212	0,89078245	1,95688616	0,96855983	0,5806699	0,25979076	0,5853763	Unpaired t-t	0,225270553	permutation FDR (250 perm	1
326	Ass1~P1646	Ass1	ko vs wt	ko	wt	28,697007	0,42993196	28,0798729	1,08798425	1,53382521	0,61713409	0,34126461	0,51264141	0,29018632	Unpaired t-t	0,454127985	permutation FDR (250 perm	1
327	Atad1~Q9D5	Atad1	ko vs wt	ko	wt	26,8219919	0,15844919	26,8712939	0,34187206	0,96640372	-0,0493021	-0,0391762	0,86272934	0,06412543	Unpaired t-t	0,934293149	permutation FDR (250 perm	1
328	Atad2b~E9Q	Atad2b	ko vs wt	ko	wt	26,9771134	0,31651588	26,6499772	0,16152685	1,25452065	0,32713622	0,26913752	0,19284171	0,71479902	Unpaired t-t	0,532095588	permutation FDR (250 perm	1
329	Atad3~Q925	Atad3	ko vs wt	ko	wt	26,2574423	2,53405947	27,792564	0,28864619	0,34505022	-1,5351217	-0,6196983	0,37252469	0,42884494	Unpaired t-t	0,190882239	permutation FDR (250 perm	1
330	Atat1~Q8K3	Atat1	ko vs wt	ko	wt	24,1685819	0,3249178	23,9567917	0,46830384	1,15812433	0,21179014	0,15340611	0,61376023	0,21200126	Unpaired t-t	0,732452894	permutation FDR (250 perm	1
331	Ate1~J3QNU	Ate1	ko vs wt	ko	wt	27,4701851	0,12370016	26,8266992	0,08138458	1,56209905	0,64348593	0,58942472	0,00090786	3,04198096	Unpaired t-t	0,194518002	permutation FDR (250 perm	1
332	Atg3~Q9CPX	Atg3	ko vs wt	ko	wt	26,4710173	0,2038536	26,893547	0,18401677	0,7461152	-0,4225297	-0,3594617	0,06573032	1,18223427	Unpaired t-t	0,409586387	permutation FDR (250 perm	1
333	Atg4b~Q8BG	Atg4b	ko vs wt	ko	wt	24,8755865	0,75689469	24,0880955	0,34152224	1,72607001	0,78749098	0,52524562	0,18218866	0,73947866	Unpaired t-t	0,243014344	permutation FDR (250 perm	1
334	Atg7~Q9D90	Atg7	ko vs wt	ko	wt	27,2500318	0,41134099	26,8919285	0,18058857	1,28173971	0,35810331	0,28205071	0,24845422	0,60475363	Unpaired t-t	0,514445404	permutation FDR (250 perm	1
335	Atic~Q9CWJ	Atic	ko vs wt	ko	wt	29,387781	0,79053961	28,5474337	0,31112439	1,79048112	0,8403473	0,55775055	0,16889964	0,77237127	Unpaired t-t	0,219834773	permutation FDR (250 perm	1
336	Atl2~Q6PA0	Atl2	ko vs wt	ko	wt	26,7996001	0,76447832	27,4019395	0,10860617	0,65868498	-0,6023394	-0,4159794	0,26665349	0,57405272	Unpaired t-t	0,357904504	permutation FDR (250 perm	1
337	Atl3~Q91YH	Atl3	ko vs wt	ko	wt	30,3986367	0,42798664	31,0234856	0,31015649	0,64848769	-0,6248489	-0,4696743	0,11756943	0,92970557	Unpaired t-t	0,286368959	permutation FDR (250 perm	1
338	Atox1~O089	Atox1	ko vs wt	ko	wt	24,1402449	0,17738027	25,6011615	0,13737504	0,36326224	-1,4609167	-1,2802122	0,00016488	3,7828228	Unpaired t-t	0,015968944	permutation FDR (250 perm	1
339	Atp13a1~Q9	Atp13a1	ko vs wt	ko	wt	27,751952	1,08275584	27,6938506	0,72479644	1,04109478	0,05810141	0,03212928	0,94548763	0,02434415	Unpaired t-t	0,943876059	permutation FDR (250 perm	1
340	Atp1a1~Q8V	Atp1a1	ko vs wt	ko	wt	32,3200163	0,3667507	32,4406524	0,04148943	0,91978202	-0,1206361	-0,0993899	0,61062191	0,21422761	Unpaired t-t	0,830296112	permutation FDR (250 perm	1
341	Atp1a2~Q6P	Atp1a2	ko vs wt	ko	wt	28,1003185	0,29089699	28,136622	0,6226798	0,97515034	-0,0363034	-0,0246753	0,94415328	0,0249575	Unpaired t-t	0,957423461	permutation FDR (250 perm	1
342	Atp1a4~Q9W	Atp1a4	ko vs wt	ko	wt	24,4892581	0,55111188	24,6817985	1,31175432	0,87506351	-0,1925404	-0,0972128	0,85931485	0,06584769	Unpaired t-t	0,838173913	permutation FDR (250 perm	1
343	Atp1b3~P97	Atp1b3	ko vs wt	ko	wt	27,8975125	0,2442012	28,6048178	0,20814768	0,61246305	-0,7073053	-0,5875528	0,01965033	1,70663016	Unpaired t-t	0,197064815	permutation FDR (250 perm	1
344	Atp2a1~Q8R	Atp2a1	ko vs wt	ko	wt	28,0848317	0,93351066	27,1361512	0,84227757	1,93010655	0,94868049	0,52609738	0,29533941	0,5296786	Unpaired t-t	0,24191458	permutation FDR (250 perm	1
345	Atp2a2~O55	Atp2a2	ko vs wt	ko	wt	32,4482687	0,22350886	32,6279443	0,06679602	0,8829015	-0,1796756	-0,1579684	0,26530793	0,57624976	Unpaired t-t	0,72112041	permutation FDR (250 perm	1
346	Atp2a3~Q64	Atp2a3	ko vs wt	ko	wt	28,8758384	1,11823633	27,6824024	0,28282502	2,28696769	1,19343598	0,71212537	0,16111579	0,79286189	Unpaired t-t	0,139551244	permutation FDR (250 perm	1
347	Atp2b1~G5E	Atp2b1	ko vs wt	ko	wt	27,5205794	0,55203617	27,3357309	0,36967788	1,13669754	0,18484842	0,13089356	0,67260189	0,17224191	Unpaired t-t	0,765926966	permutation FDR (250 perm	1
348	Atp2b4~E9Q	Atp2b4	ko vs wt	ko	wt	30,306626	0,77562663	30,8951071	0,23674091	0,6650427	-0,5884811	-0,3981403	0,2892876	0,53867017	Unpaired t-t	0,371267473	permutation FDR (250 perm	1
349	Atp2b4~Q6Q	Atp2b4	ko vs wt	ko	wt	26,7750578	0,85143342	26,7853541	0,33269227	0,99288854	-0,0102963	-0,0066644	0,98578964	0,00621575	Unpaired t-t	0,987679083	permutation FDR (250 perm	1
350	Atp5c1~Q8C	Atp5c1	ko vs wt	ko	wt	30,9175707	0,26150678	30,6875	0,26178033	1,17289243	0,23007071	0,18570986	0,38664431	0,41268838	Unpaired t-t	0,670988106	permutation FDR (250 perm	1
351	Atp5f1a~Q0	Atp5f1a	ko vs wt	ko	wt	33,5799167	0,16133782	33,5744876	0,21084668	1,0037703	0,00542916	0,00461743	0,97703745	0,01008879	Unpaired t-t	0,991842749	permutation FDR (250 perm	1
352	Atp5f1b~P56	Atp5f1b	ko vs wt	ko	wt	33,2404567	0,33053475	32,9139535	0,21704939	1,25397032	0,32650321	0,26227395	0,23997801	0,61982856	Unpaired t-t	0,541295517	permutation FDR (250 perm	1
353	Atp5f1e~P56	Atp5f1e	ko vs wt	ko	wt	23,7538422	0,73265141	25,4955267	2,21237496	0,29902035	-1,7416844	-0,664622	0,3823693	0,41751698	Unpaired t-t	0,179466352	permutation FDR (250 perm	1
354	Atp5me~Q0	Atp5me	ko vs wt	ko	wt	27,7576661	0,37511646	27,8013459	0,64788689	0,97017721	-0,0436798	-0,0289897	0,93691818	0,02829833	Unpaired t-t	0,950239578	permutation FDR (250 perm	1
355	Atp5mf~P56	Atp5mf	ko vs wt	ko	wt	29,0916699	1,02352851	28,5092115	0,73559125	1,49739866	0,58245837	0,32589778	0,49280809	0,30732217	Unpaired t-t	0,450007027	permutation FDR (250 perm	1
356	Atp5mg~Q9	Atp5mg	ko vs wt	ko	wt	29,9478564	0,49223622	29,466417	0,17855993	1,39613589	0,48143937	0,3672368	0,19540242	0,70907006	Unpaired t-t	0,40368594	permutation FDR (250 perm	1
357	Atp5mk~Q78	Atp5mk	ko vs wt	ko	wt	25,1622681	1,55344628	28,9737917	0,10021194	0,07122248	-3,8115236	-2,0064056	0,02348558	1,62919879	Unpaired t-t	0,004081752	permutation FDR (250 perm	1
358	Atp5pb~Q9C	Atp5pb	ko vs wt	ko	wt	30,2170387	0,55409676	29,8479771	0,05421241	1,2915125	0,36906161	0,27912766	0,33302695	0,47752062	Unpaired t-t	0,528826487	permutation FDR (250 perm	1
359	Atp5pd~Q9D	Atp5pd	ko vs wt	ko	wt	30,5652139	0,39852729	30,3332018	0,12252209	1,17447187	0,23201216	0,18622651	0,40142594	0,39639457	Unpaired t-t	0,673033643	permutation FDR (250 perm	1
360	Atp5po~Q9D	Atp5po	ko vs wt	ko	wt	31,5208596	0,56955987	30,9149292	0,62136656	1,5219599	0,60593034	0,39122579	0,33112804	0,48000404	Unpaired t-t	0,37672423	permutation FDR (250 perm	1
361	Atp6ap1~Q9	Atp6ap1	ko vs wt	ko	wt	24,573046	0,95610903	24,3086277	1,16218184	1,20115167	0,26441833	0,13287528	0,80358208	0,09496976	Unpaired t-t	0,765923099	permutation FDR (250 perm	1
362	Atp6v0a1~Q	Atp6v0a1	ko vs wt	ko	wt	27,8416553	0,59387729	27,622644	0,50333603	1,1639357	0,21901136	0,14657399	0,67720737	0,16927832	Unpaired t-t	0,737392584	permutation FDR (250 perm	1
363	Atp6v0c~E9	Atp6v0c	ko vs wt	ko	wt	25,4544292	1,54618397	25,4378922	1,73590453	1,01152848	0,01653694	0,00656813	0,99183597	0,00356015	Unpaired t-t	0,987904142	permutation FDR (250 perm	1
364	Atp6v0d1~P	Atp6v0d1	ko vs wt	ko	wt	28,1005529	0,50638805	27,615694	0,92642446	1,39944905	0,48485897	0,28232785	0,5506723	0,25910677	Unpaired t-t	0,527505673	permutation FDR (250 perm	1
365	Atp6v0d2~Q	Atp6v0d2	ko vs wt	ko	wt	26,9636197	0,61421743	26,2908874	0,0130203	1,5940891	0,67273227	0,49657736	0,154051	0,81233548	Unpaired t-t	0,27942695	permutation FDR (250 perm	1
366	Atp6v1a~P5	Atp6v1a	ko vs wt	ko	wt	29,7945377	0,87537372	29,370193	0,40912413	1,34196276	0,42434464	0,26817571	0,50190614	0,29937749	Unpaired t-t	0,534115632	permutation FDR (250 perm	1
367	Atp6v1b2~P	Atp6v1b2	ko vs wt	ko	wt	28,7485023	1,1305536	28,2758623	0,7853356	1,38764637	0,47263996	0,25451982	0,60505918	0,21820214	Unpaired t-t	0,552982712	permutation FDR (250 perm	1
368	Atp6v1c1~Q	Atp6v1c1	ko vs wt	ko	wt	27,0946455	0,77059248	26,5691593	0,84262778	1,43941859	0,5254862	0,30137916	0,5185868	0,28517854	Unpaired t-t	0,48960025	permutation FDR (250 perm	1
369	Atp6v1e1~P	Atp6v1e1	ko vs wt	ko	wt	27,7919943	0,43829753	24,6505612	1,37281176	8,82400179	3,14143308	1,56823444	0,07514563	1,12409625	Unpaired t-t	0,013139116	permutation FDR (250 perm	1
370	Atp6v1h~Q8	Atp6v1h	ko vs wt	ko	wt	28,2238551	0,78484486	26,8653304	0,13833168	2,56422829	1,35852471	0,92822815	0,05472651	1,26180226	Unpaired t-t	0,069926531	permutation FDR (250 perm	1
371	Atrx~Q61687	Atrx	ko vs wt	ko	wt	25,0429361	1,12510975	26,7783564	0,02276897	0,3003215	-1,7354203	-1,0519089	0,07554149	1,12181446	Unpaired t-t	0,047901546	permutation FDR (250 perm	1
372	Atxn2l~Q7T	Atxn2l	ko vs wt	ko	wt	27,4722146	0,61400987	26,8139978	0,32996106	1,57813081	0,65821679	0,46210447	0,18389961	0,73541919	Unpaired t-t	0,295001755	permutation FDR (250 perm	1
373	Auh~Q9JLZ3	Auh	ko vs wt	ko	wt	27,7559186	0,26244757	27,9255636	0,05745724	0,88906139	-0,1696451	-0,1466406	0,3498037	0,4561756	Unpaired t-t	0,742685504	permutation FDR (250 perm	1
374	Aup1~P7029	Aup1	ko vs wt	ko	wt	25,7812486	0,21869099	26,2706886	0,15317978	0,71230154	-0,48944	-0,4196321	0,03226978	1,49120395	Unpaired t-t	0,338436687	permutation FDR (250 perm	1
375	Axl~Q00993	Axl	ko vs wt	ko	wt	29,7943173	0,3231088	29,4758138	0,67704854	1,24703632	0,31850348	0,21039907	0,5851313	0,23274667	Unpaired t-t	0,640789227	permutation FDR (250 perm	1
376	Azgp1~Q647	Azgp1	ko vs wt	ko	wt	26,343342	0,6805265	23,1812774	0,32592535	8,95109752	3,16206459	2,17248465	0,00132569	2,87755722	Unpaired t-t	0,003067308	permutation FDR (250 perm	1
377	B2m~P0188	B2m	ko vs wt	ko	wt	28,0713932	0,4141733	27,6064028	0,70882434	1,38030815	0,46499039	0,29896498	0,46552993	0,33205239	Unpaired t-t	0,503493168	permutation FDR (250 perm	1
378	B3galt1~O54	B3galt1	ko vs wt	ko	wt	26,2942061	0,52532511	26,6664935	0,41886696	0,77255663	-0,3722874	-0,2614513	0,42126381	0,37544585	Unpaired t-t	0,543229503	permutation FDR (250 perm	1
379	B3glct~Q8B	B3glct	ko vs wt	ko	wt	25,4235646	1,05691538	23,6707399	0,37533448	3,37017764	1,75282464	1,05247686	0,0575493	1,23995996	Unpaired t-t	0,040249554	permutation FDR (250 perm	1
380	Babam2~Q8	Babam2	ko vs wt	ko	wt	24,7558849	0,71029028	24,5879569	0,20298278	1,12344379	0,16792795	0,11706549	0,72032772	0,14246987	Unpaired t-t	0,796425105	permutation FDR (250 perm	1
381	Bach1~P973	Bach1	ko vs wt	ko	wt	24,2941449	0,43595815	24,3124623	0,19642031	0,9873836	-0,0183174	-0,0142274	0,95193761	0,02139151	Unpaired t-t	0,975001309	permutation FDR (250 perm	1

382	Bag3~Q9JLV	Bag3	ko vs wt	ko	wt	29,3416993	0,27295599	29,5032204	0,15160267	0,89408185	-0,1615212	-0,1356642	0,43648793	0,36002776	Unpaired t-te	0,756686357	permutation FDR (250 perm	1
383	Bag6~Q9Z1R	Bag6	ko vs wt	ko	wt	25,4657478	0,95992334	26,463997	0,17825102	0,50060713	-0,9982493	-0,6364908	0,16875205	0,77275094	Unpaired t-te	0,178411348	permutation FDR (250 perm	1
384	Baiap2l1~Q9	Baiap2l1	ko vs wt	ko	wt	23,3903315	0,11289246	24,1176121	0,46877283	0,60404144	-0,7272806	-0,5436312	0,1549508	0,80980618	Unpaired t-te	0,2592026	permutation FDR (250 perm	1
385	Baiap2~Q8B	Baiap2	ko vs wt	ko	wt	24,8853964	1,14750248	24,0097837	0,20092528	1,83478716	0,87561271	0,52195186	0,27996402	0,55289778	Unpaired t-te	0,25598259	permutation FDR (250 perm	1
386	Banf1~O549	Banf1	ko vs wt	ko	wt	31,4806973	0,58861866	31,9227131	0,18232554	0,73610538	-0,4420158	-0,324184	0,29392586	0,5317622	Unpaired t-te	0,459248015	permutation FDR (250 perm	1
387	Baz1b~Q9Z2	Baz1b	ko vs wt	ko	wt	28,7064767	0,86177581	28,4779606	0,52525352	1,17162924	0,22851611	0,14098231	0,72801138	0,13786183	Unpaired t-te	0,747139469	permutation FDR (250 perm	1
388	Bbs1~Q3V3N	Bbs1	ko vs wt	ko	wt	24,1904228	1,09418636	24,8427548	0,64300523	0,63625103	-0,652332	-0,3668213	0,44070384	0,35585316	Unpaired t-te	0,398811594	permutation FDR (250 perm	1
389	Bbs9~Q811G	Bbs9	ko vs wt	ko	wt	26,3813986	0,216317	25,2191394	1,00241589	2,23807631	1,16225923	0,67583678	0,24045325	0,61896935	Unpaired t-te	0,177791798	permutation FDR (250 perm	1
390	Bcam~Q9R0	Bcam	ko vs wt	ko	wt	30,2914764	1,03683606	30,8551421	0,14228422	0,67658088	-0,5636657	-0,3507535	0,41828181	0,37853102	Unpaired t-te	0,432117457	permutation FDR (250 perm	1
391	Bcap31~Q61	Bcap31	ko vs wt	ko	wt	26,6876993	2,03040752	28,2574758	0,44518545	0,33686057	-1,5697765	-0,7090909	0,27630882	0,55860525	Unpaired t-te	0,142345233	permutation FDR (250 perm	1
392	Bcar1~Q611	Bcar1	ko vs wt	ko	wt	23,9293134	0,47668929	26,086263	0,07632598	0,22422987	-2,1569496	-1,6845133	0,00351285	2,45434034	Unpaired t-te	0,007014054	permutation FDR (250 perm	1
393	Bcas2~Q9D2	Bcas2	ko vs wt	ko	wt	26,0061927	1,47684428	25,1729312	0,91033365	1,7817088	0,83326156	0,40286249	0,47090153	0,3270699	Unpaired t-te	0,357622757	permutation FDR (250 perm	1
394	Bcas3~Q8CC	Bcas3	ko vs wt	ko	wt	26,5931322	0,32118891	27,1412141	0,11037641	0,68392883	-0,5480819	-0,456281	0,05319112	1,27416084	Unpaired t-te	0,307467319	permutation FDR (250 perm	1
395	Bcat2~O358	Bcat2	ko vs wt	ko	wt	26,2675643	0,86823668	24,5502763	1,29969723	3,28817718	1,71728804	0,83899265	0,19430632	0,71151307	Unpaired t-te	0,094911985	permutation FDR (250 perm	1
396	Bccip~Q9CW	Bccip	ko vs wt	ko	wt	24,2838578	0,84342001	24,5395328	0,39903115	0,83759516	-0,255675	-0,1636017	0,67033086	0,17371079	Unpaired t-te	0,707732189	permutation FDR (250 perm	1
397	Bckdha~Q3U	Bckdha	ko vs wt	ko	wt	26,2889729	0,50334993	25,9467158	0,1137928	1,26773848	0,34225716	0,26296264	0,3290727	0,48270814	Unpaired t-te	0,549179472	permutation FDR (250 perm	1
398	Bckdhb~Q6P	Bckdhb	ko vs wt	ko	wt	26,6548341	0,84990348	26,4241255	0,73120623	1,17341111	0,23070855	0,13469529	0,76013014	0,11911205	Unpaired t-te	0,75942391	permutation FDR (250 perm	1
399	Bckdk~O550	Bckdk	ko vs wt	ko	wt	27,262452	0,40358027	26,6041705	0,82267009	1,5782015	0,65828142	0,40468631	0,37978807	0,42045868	Unpaired t-te	0,378036145	permutation FDR (250 perm	1
400	Bcl2l13~P59	Bcl2l13	ko vs wt	ko	wt	24,4245358	0,57200023	24,2419595	0,52495865	1,13490877	0,18257633	0,12197433	0,7298086	0,13679102	Unpaired t-te	0,784106234	permutation FDR (250 perm	1
401	Bclaf1~Q8K0	Bclaf1	ko vs wt	ko	wt	26,3371113	0,66086858	28,361131	0,19679447	0,24587217	-2,0240197	-1,4394196	0,0089423	2,04855074	Unpaired t-te	0,011354616	permutation FDR (250 perm	1
402	Bcr~Q6PAJ1	Bcr	ko vs wt	ko	wt	26,6562428	0,27863984	26,3298131	0,26889529	1,25390649	0,32642976	0,2613397	0,25436278	0,59454644	Unpaired t-te	0,545372263	permutation FDR (250 perm	1
403	Bcs1l~Q9CZ	Bcs1l	ko vs wt	ko	wt	26,2616998	0,26517739	26,2619243	0,14763223	0,99984437	-0,0002245	-0,0001894	0,9990813	0,00039917	Unpaired t-te	0,999492855	permutation FDR (250 perm	1
404	Bgn~P28653	Bgn	ko vs wt	ko	wt	35,8594562	0,24464897	35,8353313	0,07795066	1,01686269	0,02412489	0,02094862	0,88157751	0,0547395	Unpaired t-te	0,963672011	permutation FDR (250 perm	1
405	Bhmt~O354	Bhmt	ko vs wt	ko	wt	25,5536751	1,70017138	25,1107116	0,43672512	1,35939392	0,44296358	0,21831354	0,69154461	0,1601798	Unpaired t-te	0,618339823	permutation FDR (250 perm	1
406	Bin1~O0853	Bin1	ko vs wt	ko	wt	25,036676	1,19696726	24,4043443	0,51677718	1,55006814	0,63233164	0,354897	0,46009092	0,33715634	Unpaired t-te	0,41659322	permutation FDR (250 perm	1
407	Bin2~D3Z6Q	Bin2	ko vs wt	ko	wt	25,7402287	2,48311213	24,495773	0,62569465	2,36929145	1,24445568	0,497714	0,4589581	0,33822696	Unpaired t-te	0,27196	permutation FDR (250 perm	1
408	Birc6~O8873	Birc6	ko vs wt	ko	wt	26,1518276	0,72551554	24,8673445	1,13169524	2,4359476	1,2844831	0,67489637	0,2475867	0,6062727	Unpaired t-te	0,161573691	permutation FDR (250 perm	1
409	Blmh~Q8R01	Blmh	ko vs wt	ko	wt	25,1916976	1,11163767	24,5680022	0,64256776	1,54081685	0,62369539	0,34914349	0,46427394	0,33322569	Unpaired t-te	0,421131707	permutation FDR (250 perm	1
410	Bloc1s5~Q8	Bloc1s5	ko vs wt	ko	wt	24,8243732	0,44125306	26,8150683	3,13144615	0,25161764	-1,990695	-0,6165292	0,46392486	0,33355235	Unpaired t-te	0,212954975	permutation FDR (250 perm	1
411	Bltp3b~A2RS	Bltp3b	ko vs wt	ko	wt	25,9832471	0,33661584	26,4784044	0,41115256	0,70948431	-0,4951573	-0,3668635	0,2352962	0,62838509	Unpaired t-te	0,407403083	permutation FDR (250 perm	1
412	Blvra~Q9CY6	Blvra	ko vs wt	ko	wt	27,2201553	0,52790685	26,9342928	0,17806923	1,2191389	0,28586251	0,21497108	0,43563257	0,36087965	Unpaired t-te	0,621680613	permutation FDR (250 perm	1
413	Blvrb~Q923	Blvrb	ko vs wt	ko	wt	28,2773561	1,32522908	28,1619789	0,80421055	1,08325816	0,11537711	0,05906767	0,90841853	0,04171402	Unpaired t-te	0,8967308	permutation FDR (250 perm	1
414	Bmp1~P980	Bmp1	ko vs wt	ko	wt	28,1889584	0,5197995	28,236436	1,15247316	0,96762667	-0,0474776	-0,0254105	0,96039667	0,01754935	Unpaired t-te	0,956095764	permutation FDR (250 perm	1
415	Bmp2k~Q91	Bmp2k	ko vs wt	ko	wt	24,6680466	0,59223513	24,1770254	0,26251964	1,40543937	0,49102122	0,35349018	0,26903795	0,57018646	Unpaired t-te	0,418050814	permutation FDR (250 perm	1
416	Bms1~Q6PG	Bms1	ko vs wt	ko	wt	24,5692414	1,10854979	24,0209259	0,68603775	1,46237726	0,54831554	0,30409793	0,52525148	0,27963271	Unpaired t-te	0,480310692	permutation FDR (250 perm	1
417	Bmx~P97504	Bmx	ko vs wt	ko	wt	25,513585	0,13627192	24,8990146	0,57727041	1,531102	0,61457039	0,43410911	0,26908213	0,57011515	Unpaired t-te	0,357942431	permutation FDR (250 perm	1
418	Bpgm~P153	Bpgm	ko vs wt	ko	wt	24,946186	0,50601101	24,2030787	0,24045712	1,673777	0,74310732	0,55537752	0,08413287	1,07503427	Unpaired t-te	0,21897594	permutation FDR (250 perm	1
419	Bphl~Q8R16	Bphl	ko vs wt	ko	wt	25,5188077	1,17419946	25,793248	0,10187998	0,82677099	-0,2744403	-0,1631882	0,71368977	0,14649053	Unpaired t-te	0,716787896	permutation FDR (250 perm	1
420	Bpnt1~Q9Z0	Bpnt1	ko vs wt	ko	wt	23,7287788	0,46645773	25,4611373	1,93970549	0,30095954	-1,7323586	-0,7224879	0,33314206	0,47737053	Unpaired t-te	0,155801883	permutation FDR (250 perm	1
421	Bpnt2~Q80V	Bpnt2	ko vs wt	ko	wt	24,4556947	0,53558996	24,6876931	0,36946355	0,85145463	-0,2319984	-0,1651459	0,5914104	0,22811105	Unpaired t-te	0,703832665	permutation FDR (250 perm	1
422	Braf~F6SZ47	Braf	ko vs wt	ko	wt	29,3013226	0,65173211	29,2926407	0,70774451	1,00603599	0,00868192	0,00533901	0,98959466	0,00454266	Unpaired t-te	0,990376464	permutation FDR (250 perm	1
423	Brap~Q99M	Brap	ko vs wt	ko	wt	24,9591639	0,76915964	25,6104721	1,10581013	0,63670271	-0,6513082	-0,3429332	0,51724635	0,28630256	Unpaired t-te	0,439956354	permutation FDR (250 perm	1
424	Brcc3~P4673	Brcc3	ko vs wt	ko	wt	27,074553	0,4885716	26,769931	0,40128805	1,23509499	0,304622	0,21757094	0,48223214	0,31674385	Unpaired t-te	0,612782737	permutation FDR (250 perm	1
425	Brix1~Q9DC	Brix1	ko vs wt	ko	wt	26,3960836	0,16306278	26,7420363	0,45105885	0,78678821	-0,3459528	-0,2596169	0,39316886	0,40542089	Unpaired t-te	0,566847151	permutation FDR (250 perm	1
426	Brk1~Q91VR	Brk1	ko vs wt	ko	wt	24,6483066	1,42304002	24,1576171	0,32404948	1,40511624	0,49068948	0,26481573	0,6004707	0,22150818	Unpaired t-te	0,546219386	permutation FDR (250 perm	1
427	Brox~Q8K2Q	Brox	ko vs wt	ko	wt	27,428436	0,37315825	27,1133667	0,75564445	1,24407138	0,31506927	0,19990175	0,62700903	0,2027262	Unpaired t-te	0,657965055	permutation FDR (250 perm	1
428	Btk~P35991	Btk	ko vs wt	ko	wt	26,4870669	2,01623606	24,137368	0,33757857	5,09717849	2,34969887	1,07375701	0,13536229	0,86850229	Unpaired t-te	0,042034259	permutation FDR (250 perm	1
429	Bub3~Q9WV	Bub3	ko vs wt	ko	wt	28,034675	0,26329757	27,6961724	0,11558158	1,26444356	0,33850265	0,2886789	0,11447783	0,94127862	Unpaired t-te	0,50508098	permutation FDR (250 perm	1
430	Bud31~E0CX	Bud31	ko vs wt	ko	wt	24,6291911	0,89254615	25,0294376	0,70762992	0,75772877	-0,4002466	-0,2329354	0,6020866	0,22034104	Unpaired t-te	0,586560764	permutation FDR (250 perm	1
431	Bzw1~Q9CQ	Bzw1	ko vs wt	ko	wt	29,7906179	0,7651856	29,1520957	0,44481653	1,55673373	0,6385222	0,4140037	0,29285983	0,53334019	Unpaired t-te	0,344899489	permutation FDR (250 perm	1
432	C1qa~P9808	C1qa	ko vs wt	ko	wt	30,526237	0,43462038	29,7504057	0,81113381	1,71217641	0,77583135	0,47712746	0,30985552	0,50884076	Unpaired t-te	0,301587947	permutation FDR (250 perm	1
433	C1qbp~Q8R5	C1qbp	ko vs wt	ko	wt	24,57335	1,4286038	24,2187737	0,26741335	1,27861001	0,3545763	0,19205715	0,70092777	0,15432673	Unpaired t-te	0,665451904	permutation FDR (250 perm	1
434	C1qb~P1410	C1qb	ko vs wt	ko	wt	29,3440948	0,56568674	29,5477087	1,06702938	0,86837257	-0,2036139	-0,1117433	0,8216921	0,08529089	Unpaired t-te	0,809944506	permutation FDR (250 perm	1
435	C1qc~Q0210	C1qc	ko vs wt	ko	wt	31,2384513	0,32650945	30,5123049	1,23789316	1,65421461	0,72614642	0,38311158	0,49617326	0,30436664	Unpaired t-te	0,410471704	permutation FDR (250 perm	1
436	C1qtnf2~Q9	C1qtnf2	ko vs wt	ko	wt	25,7221126	2,09815634	25,842671	2,05780248	0,91983157	-0,1205584	-0,0416677	0,95205359	0,0213386	Unpaired t-te	0,928037294	permutation FDR (250 perm	1
437	C1qtnf3~D3Y	C1qtnf3	ko vs wt	ko	wt	27,9817379	0,48260573	27,0743252	0,15633618	1,87567868	0,9074127	0,69813817	0,04052703	1,3922552	Unpaired t-te	0,143342282	permutation FDR (250 perm	1
438	C1qtnf7~Q8	C1qtnf7	ko vs wt	ko	wt	26,9216501	0,32683738	26,7530458	0,44407854	1,12397066	0,16860438	0,12339788	0,67314482	0,17189149	Unpaired t-te	0,785400391	permutation FDR (250 perm	1
439	C1ra~Q8CG1	C1ra	ko vs wt	ko	wt	27,3201782	0,47940099	26,7732094	0,69898551	1,46101282	0,54696884	0,3491708	0,40069839	0,3971824	Unpaired t-te	0,433318284	permutation FDR (250 perm	1
440	C1s1~E9Q6C	C1s1	ko vs wt	ko	wt	28,3865881	0,18403404	27,3934078	0,42805132	1,99056824	0,99318033	0,75196175	0,06803865	1,16724431	Unpaired t-te	0,13679898	permutation FDR (250 perm	1
441	C3~P01027	C3	ko vs wt	ko	wt	33,3474843	0,44307958	33,0292962	0,26397717	1,24676374	0,3181881	0,24166068	0,36162161	0,44174563	Unpaired t-te	0,572918724	permutation FDR (250 perm	1
442	C4bpa~P086	C4bpa	ko vs wt	ko	wt	28,0870889	1,18058513	27,3011918	0,34471738	1,72416416	0,78589715	0,45588735	0,34283553	0,46491418	Unpaired t-te	0,310242065	permutation FDR (250 perm	1
443	C4b~P01029	C4b	ko vs wt	ko	wt	32,2841768	0,42204341	31,935778	0,16934606	1,27314682	0,3483988	0,274006	0,26488861	0,57693671	Unpaired t-te	0,526741104	permutation FDR (250 perm	1
444	C5~P06684	C5	ko vs wt	ko	wt	29,8761365	1,14075373	27,9800342	0,31148703	3,72206253	1,89610229	1,11899596	0,05811557	1,2357075	Unpaired t-te	0,033967378	permutation FDR (250 perm	1
445	C6~Q91X70	C6	ko vs wt	ko	wt	28,7192185	0,54519603	26,1626807	1,58029945	5,88294202	2,55653782	1,18307429	0,14080248	0,85138969	Unpaired t-te	0,037368165	permutation FDR (250 perm	1

446	C7~D3YXF5	C7	ko vs wt	ko	wt	27,7448589	0,37820585	25,6116002	2,40260248	4,38707287	2,13325867	0,78634708	0,33564555	0,4741191	Unpaired t-te	0,133453763	permutation FDR (250 perm	1
447	C8a~Q8K182	C8a	ko vs wt	ko	wt	25,9050239	1,5867929	24,5903611	0,32327622	2,48744176	1,31466274	0,67618917	0,24872902	0,60427353	Unpaired t-te	0,157613377	permutation FDR (250 perm	1
448	C8b~Q8BH35	C8b	ko vs wt	ko	wt	26,8781512	0,54945282	25,3013242	2,09935718	2,9831302	1,57682694	0,62622533	0,40007644	0,39785702	Unpaired t-te	0,20427116	permutation FDR (250 perm	1
449	C8g~Q8VCG4	C8g	ko vs wt	ko	wt	28,0035074	0,63895175	27,2415081	0,06781896	1,69583911	0,76199931	0,55539191	0,1299997	0,88605766	Unpaired t-te	0,232968437	permutation FDR (250 perm	1
450	C9~P06683	C9	ko vs wt	ko	wt	28,7134189	0,53490674	28,0515533	0,10649243	1,58212715	0,66186555	0,50222127	0,119207	0,92369826	Unpaired t-te	0,270786533	permutation FDR (250 perm	1
451	Ca2~P00920	Ca2	ko vs wt	ko	wt	28,357084	0,59272012	25,8545464	1,03110881	5,66681311	2,50253762	1,38612632	0,05583971	1,25305688	Unpaired t-te	0,016845455	permutation FDR (250 perm	1
452	Ca3~P16015	Ca3	ko vs wt	ko	wt	29,6703298	0,96070095	27,7517078	1,99080518	3,7806179	1,91862205	0,76346544	0,30551158	0,51497232	Unpaired t-te	0,130153713	permutation FDR (250 perm	1
453	Cab39~Q061	Cab39	ko vs wt	ko	wt	27,5836587	0,55028787	25,3336601	1,72566067	4,75682397	2,24999864	0,99517438	0,20089748	0,69702551	Unpaired t-te	0,068244604	permutation FDR (250 perm	1
454	Cacna2d1~O	Cacna2d1	ko vs wt	ko	wt	27,9732961	0,9222534	28,5346985	0,22416832	0,67764313	-0,5614024	-0,3609014	0,37668062	0,42402672	Unpaired t-te	0,416070404	permutation FDR (250 perm	1
455	Cacnb2~Q8C	Cacnb2	ko vs wt	ko	wt	25,5410086	0,31250399	24,3045235	0,34047287	2,35623766	1,23648506	0,95051712	0,01451024	1,83832529	Unpaired t-te	0,058930608	permutation FDR (250 perm	1
456	Cacul1~Q8R	Cacul1	ko vs wt	ko	wt	25,0499369	0,84818435	24,0613477	0,35151466	1,98424364	0,98858918	0,6381415	0,14095607	0,85091621	Unpaired t-te	0,169796474	permutation FDR (250 perm	1
457	Cacybp~Q9C	Cacybp	ko vs wt	ko	wt	28,2187243	0,36050258	28,1305408	0,19385541	1,06303088	0,0881835	0,07059085	0,7384406	0,13168443	Unpaired t-te	0,878810223	permutation FDR (250 perm	1
458	Cad~B2RQC6	Cad	ko vs wt	ko	wt	30,3923128	0,69340079	29,9080235	0,32550094	1,39889659	0,48428932	0,33130039	0,3466363	0,46012596	Unpaired t-te	0,444973111	permutation FDR (250 perm	1
459	Cald1~E9QA	Cald1	ko vs wt	ko	wt	31,4285351	1,0309471	32,4276329	0,40881383	0,50031279	-0,9990978	-0,60125	0,20179524	0,69508907	Unpaired t-te	0,191442547	permutation FDR (250 perm	1
460	Cald1~E9QA	Cald1	ko vs wt	ko	wt	27,6558659	0,46858221	28,0547195	1,08688864	0,75846076	-0,3988536	-0,2197816	0,66412202	0,17775212	Unpaired t-te	0,627370218	permutation FDR (250 perm	1
461	Calm3~P622	Calm3	ko vs wt	ko	wt	25,3228811	0,82153503	26,3284347	0,24956455	0,49807898	-1,0055536	-0,6676639	0,12197443	0,91373119	Unpaired t-te	0,158094617	permutation FDR (250 perm	1
462	Calr~P14211	Calr	ko vs wt	ko	wt	30,4457349	0,61033088	30,1281991	0,5960474	1,24620013	0,31753577	0,20494548	0,59184565	0,22779154	Unpaired t-te	0,636805865	permutation FDR (250 perm	1
463	Calu~O3588	Calu	ko vs wt	ko	wt	26,0568103	1,22654536	25,4303067	0,30034699	1,54381894	0,62650356	0,36020291	0,4504985	0,34630665	Unpaired t-te	0,416811726	permutation FDR (250 perm	1
464	Camk1~Q91	Camk1	ko vs wt	ko	wt	27,9059457	0,52461104	27,5874446	0,64283322	1,24703422	0,31850105	0,2059869	0,59362394	0,22648859	Unpaired t-te	0,638349634	permutation FDR (250 perm	1
465	Camk2d~Q6	Camk2d	ko vs wt	ko	wt	31,0000983	0,28602277	31,199081	0,26329093	0,87116464	-0,1989827	-0,1593316	0,46387975	0,33359459	Unpaired t-te	0,716163335	permutation FDR (250 perm	1
466	Camk2g~Q92	Camk2g	ko vs wt	ko	wt	30,0514858	0,24758212	30,458812	0,13903426	0,75401955	-0,4073262	-0,3471077	0,06706756	1,17348747	Unpaired t-te	0,424423927	permutation FDR (250 perm	1
467	Cand1~Q6ZC	Cand1	ko vs wt	ko	wt	31,1063037	0,23529916	30,9765675	0,06140321	1,09409358	0,12973614	0,1135427	0,41991974	0,37683371	Unpaired t-te	0,803367483	permutation FDR (250 perm	1
468	Cand2~Q6ZC	Cand2	ko vs wt	ko	wt	24,9952468	0,53412531	25,0509273	1,09915284	0,96214048	-0,0556805	-0,0303244	0,95162947	0,02153212	Unpaired t-te	0,948673884	permutation FDR (250 perm	1
469	Canx~P3556	Canx	ko vs wt	ko	wt	30,5508016	0,34821027	30,101981	0,6469452	1,36492397	0,44882059	0,29927643	0,44003624	0,35651156	Unpaired t-te	0,504802979	permutation FDR (250 perm	1
470	Cap1~P4012	Cap1	ko vs wt	ko	wt	32,654738	0,26137218	33,2876475	0,3407128	0,64487459	-0,6329095	-0,4928132	0,09923977	1,00331426	Unpaired t-te	0,27688968	permutation FDR (250 perm	1
471	Cap2~Q9CYT	Cap2	ko vs wt	ko	wt	29,3630629	0,37343066	29,5211984	0,68605045	0,89618247	-0,1581356	-0,1032983	0,78654403	0,10427696	Unpaired t-te	0,823986751	permutation FDR (250 perm	1
472	Capg~Q99LB	Capg	ko vs wt	ko	wt	30,8413617	0,16721281	29,9434554	0,37828485	1,86335973	0,89790622	0,69909915	0,06431635	1,19167859	Unpaired t-te	0,158508557	permutation FDR (250 perm	1
473	Capn1~O353	Capn1	ko vs wt	ko	wt	29,2229456	0,42611195	29,4883016	0,46749537	0,83199341	-0,265356	-0,1879202	0,55459102	0,25602717	Unpaired t-te	0,668240348	permutation FDR (250 perm	1
474	Capn2~O085	Capn2	ko vs wt	ko	wt	31,9698029	0,34671125	32,1319576	0,40452688	0,89368933	-0,1621547	-0,1201922	0,66750876	0,17554303	Unpaired t-te	0,789761027	permutation FDR (250 perm	1
475	Capn6~O356	Capn6	ko vs wt	ko	wt	24,4727149	0,71612005	24,54367	0,90277667	0,95200758	-0,070955	-0,0403026	0,93061877	0,03122819	Unpaired t-te	0,930772014	permutation FDR (250 perm	1
476	Capns1~O88	Capns1	ko vs wt	ko	wt	29,5283001	0,83023955	29,2824496	0,25479731	1,18579161	0,2458505	0,16259085	0,65751556	0,18209396	Unpaired t-te	0,713265504	permutation FDR (250 perm	1
477	Caprin1~Q60	Caprin1	ko vs wt	ko	wt	27,6092147	0,58215865	28,3335372	0,39042076	0,60528118	-0,7243226	-0,5047711	0,15673757	0,8048269	Unpaired t-te	0,2562219	permutation FDR (250 perm	1
478	Capza1~Q5R	Capza1	ko vs wt	ko	wt	28,9443515	0,7294562	29,492695	0,48436159	0,68380483	-0,5483435	-0,3554125	0,35878379	0,44516719	Unpaired t-te	0,412592157	permutation FDR (250 perm	1
479	Capza2~P47	Capza2	ko vs wt	ko	wt	31,1648087	0,25072847	31,2333533	0,14915074	0,95359951	-0,0685446	-0,0581328	0,71784205	0,14397111	Unpaired t-te	0,898318794	permutation FDR (250 perm	1
480	Capzb~P477	Capzb	ko vs wt	ko	wt	30,9169666	0,21741498	31,3895611	0,35896389	0,72066741	-0,4725945	-0,368303	0,19435672	0,71140043	Unpaired t-te	0,413515844	permutation FDR (250 perm	1
481	Card9~A2AI	Card9	ko vs wt	ko	wt	25,5774709	0,48606719	24,8754493	0,38897749	1,6267828	0,70202164	0,50398387	0,13602735	0,86637376	Unpaired t-te	0,257913799	permutation FDR (250 perm	1
482	Carhsp1~Q9	Carhsp1	ko vs wt	ko	wt	25,0329014	0,35421668	24,18232	0,40983038	1,80322758	0,85058148	0,62787996	0,07699148	1,11355731	Unpaired t-te	0,177928262	permutation FDR (250 perm	1
483	Carm1~Q9W	Carm1	ko vs wt	ko	wt	25,135334	1,34420576	24,1609085	0,59036598	1,96485863	0,97442551	0,51797317	0,3252248	0,48781635	Unpaired t-te	0,248541408	permutation FDR (250 perm	1
484	Carmil1~D3Z	Carmil1	ko vs wt	ko	wt	26,7476552	0,21508004	26,9207458	0,12120943	0,88694064	-0,1730905	-0,150398	0,30443445	0,5165062	Unpaired t-te	0,730828271	permutation FDR (250 perm	1
485	Cars1~Q9ER	Cars1	ko vs wt	ko	wt	27,6602604	0,33192497	27,0762701	0,62256988	1,4989895	0,58399028	0,39455433	0,31746486	0,49830434	Unpaired t-te	0,386696346	permutation FDR (250 perm	1
486	Caskin2~Q8	Caskin2	ko vs wt	ko	wt	24,4579376	0,88797438	25,7238249	0,40215348	0,41584355	-1,2658873	-0,7980345	0,08946122	1,0483652	Unpaired t-te	0,097826248	permutation FDR (250 perm	1
487	Cask~O7058	Cask	ko vs wt	ko	wt	27,3622992	0,64764539	27,205096	0,23169736	1,1151233	0,15720324	0,11163131	0,71959827	0,14290989	Unpaired t-te	0,805247288	permutation FDR (250 perm	1
488	Casp12~O08	Casp12	ko vs wt	ko	wt	26,7756892	0,22028022	26,8613086	0,24297834	0,94237984	-0,0856194	-0,0705406	0,70926543	0,1491912	Unpaired t-te	0,880310954	permutation FDR (250 perm	1
489	Casp1~P294	Casp1	ko vs wt	ko	wt	25,4133083	1,10226023	25,3098646	0,51763548	1,07433484	0,10344371	0,0596512	0,89395065	0,04868646	Unpaired t-te	0,896183288	permutation FDR (250 perm	1
490	Casp8~O891	Casp8	ko vs wt	ko	wt	25,4140948	0,58487064	23,5547941	0,89186366	3,62831747	1,85930069	1,08391502	0,07679392	1,11467316	Unpaired t-te	0,041403675	permutation FDR (250 perm	1
491	Cat~P24270	Cat	ko vs wt	ko	wt	27,2978938	1,67516651	24,7357765	1,18279963	5,90573778	2,5621173	1,12440993	0,10163084	0,9929745	Unpaired t-te	0,027806623	permutation FDR (250 perm	1
492	Cav1~P4981	Cav1	ko vs wt	ko	wt	29,7192367	1,03879589	30,821422	0,12938227	0,46581036	-1,1021854	-0,6859989	0,16278685	0,78838068	Unpaired t-te	0,155478902	permutation FDR (250 perm	1
493	Cav2~Q9WV	Cav2	ko vs wt	ko	wt	28,4308532	1,42551484	28,2232845	0,48667825	1,1547405	0,20756868	0,10970444	0,82754148	0,08221023	Unpaired t-te	0,808260966	permutation FDR (250 perm	1
494	Cavin1~O547	Cavin1	ko vs wt	ko	wt	33,7785268	0,44537873	34,2684973	0,25088335	0,71203964	-0,4899705	-0,3733401	0,17902031	0,74709769	Unpaired t-te	0,392181577	permutation FDR (250 perm	1
495	Cavin2~Q63	Cavin2	ko vs wt	ko	wt	29,3824018	0,3833025	28,8506756	0,55150531	1,44565784	0,53172613	0,36711556	0,3146528	0,5021684	Unpaired t-te	0,410891141	permutation FDR (250 perm	1
496	Cavin3~Q91	Cavin3	ko vs wt	ko	wt	31,467056	0,66725852	31,4619731	0,35048628	1,00352944	0,00508294	0,00348606	0,99159552	0,00366545	Unpaired t-te	0,993590424	permutation FDR (250 perm	1
497	Cbfb~Q0802	Cbfb	ko vs wt	ko	wt	25,0842986	1,00552542	24,7667848	0,56050905	1,24618119	0,31751385	0,18645136	0,67091348	0,17333348	Unpaired t-te	0,667055749	permutation FDR (250 perm	1
498	Cbl~P22682	Cbl	ko vs wt	ko	wt	24,9025061	1,52712299	24,6025862	0,42611137	1,23107606	0,2999199	0,15525833	0,76504121	0,11631517	Unpaired t-te	0,726348864	permutation FDR (250 perm	1
499	Cbr1~P4875	Cbr1	ko vs wt	ko	wt	30,3605797	0,30890232	29,8995748	0,55383375	1,37649927	0,46100384	0,32230884	0,36666999	0,43572463	Unpaired t-te	0,471532304	permutation FDR (250 perm	1
500	Cbr2~P0807	Cbr2	ko vs wt	ko	wt	30,3622202	0,39931286	30,4042935	0,05133129	0,97125816	-0,0420733	-0,0341121	0,86792316	0,06151872	Unpaired t-te	0,942326444	permutation FDR (250 perm	1
501	Cbr3~Q8K35	Cbr3	ko vs wt	ko	wt	28,1319066	1,28598019	28,0905078	0,10676257	1,02911111	0,04139875	0,0237067	0,95918386	0,01809814	Unpaired t-te	0,958861652	permutation FDR (250 perm	1
502	Cbr4~Q91VT	Cbr4	ko vs wt	ko	wt	27,6811676	0,43756759	27,482826	0,25728837	1,14737864	0,19834157	0,15125331	0,55246672	0,25769388	Unpaired t-te	0,729212156	permutation FDR (250 perm	1
503	Cbx3~P2319	Cbx3	ko vs wt	ko	wt	25,9751047	2,18150448	25,3908606	1,34543424	1,49925324	0,58424409	0,22658975	0,72651843	0,13875337	Unpaired t-te	0,59641047	permutation FDR (250 perm	1
504	Cbx5~Q6168	Cbx5	ko vs wt	ko	wt	25,7540271	1,65789691	26,9391964	0,38194443	0,43977293	-1,1851693	-0,5942011	0,3085986	0,51060604	Unpaired t-te	0,20254197	permutation FDR (250 perm	1
505	Cc2d1a~Q8K	Cc2d1a	ko vs wt	ko	wt	27,0526439	0,25757719	27,4359564	0,4714412	0,76667525	-0,3833125	-0,2808097	0,3757057	0,42515222	Unpaired t-te	0,530136113	permutation FDR (250 perm	1
506	Cc2d1b~Q8B	Cc2d1b	ko vs wt	ko	wt	26,362934	0,31838977	25,9322525	0,04472115	1,34787013	0,4306815	0,36297792	0,09925389	1,00325246	Unpaired t-te	0,417228664	permutation FDR (250 perm	1
507	Ccar1~Q8CH	Ccar1	ko vs wt	ko	wt	28,6923019	0,44997372	28,4783192	0,44219097	1,15988573	0,21398269	0,15213625	0,62440845	0,20453123	Unpaired t-te	0,729591593	permutation FDR (250 perm	1
508	Ccar2~Q8VD	Ccar2	ko vs wt	ko	wt	27,5471034	0,43972812	27,9350115	0,43930078	0,76423695	-0,3879081	-0,2768437	0,38489415	0,41465869	Unpaired t-te	0,522181818	permutation FDR (250 perm	1
509	Ccdc141~E9	Ccdc141	ko vs wt	ko	wt	27,7549549	0,93977584	28,2400254	0,55316237	0,71446219	-0,4850704	-0,290658	0,5012652	0,29993244	Unpaired t-te	0,499406532	permutation FDR (250 perm	1

510	Ccdc22~Q9JI	Ccdc22	ko vs wt	ko	wt	27,5181985	0,39503011	27,0929049	0,65702921	1,34284579	0,42529363	0,28024981	0,4720885	0,32597658	Unpaired t-te	0,528524831	permutation FDR (250 perm	1
511	Ccdc3~Q9D6	Ccdc3	ko vs wt	ko	wt	27,7322934	0,59496924	28,5128803	0,63975623	0,58212994	-0,7805869	-0,4978188	0,24019881	0,61942916	Unpaired t-te	0,267972731	permutation FDR (250 perm	1
512	Ccdc47~Q9D	Ccdc47	ko vs wt	ko	wt	26,5512486	1,17852431	26,0824057	1,05970451	1,38399898	0,46884288	0,23300522	0,66437554	0,17758637	Unpaired t-te	0,587620299	permutation FDR (250 perm	1
513	Ccdc6~D3YZ	Ccdc6	ko vs wt	ko	wt	24,3976208	1,13213498	25,1007121	0,72503632	0,61425463	-0,7030913	-0,3840535	0,43602482	0,36048878	Unpaired t-te	0,379021063	permutation FDR (250 perm	1
514	Ccdc80~Q8R	Ccdc80	ko vs wt	ko	wt	29,3598042	0,24650589	29,6037846	0,34664704	0,84441238	-0,2439804	-0,1900991	0,44698495	0,3497071	Unpaired t-te	0,668804644	permutation FDR (250 perm	1
515	Ccdc88a~Q5	Ccdc88a	ko vs wt	ko	wt	24,3901179	1,15654298	24,5070528	1,53600415	0,92214477	-0,1169348	-0,0514009	0,93197164	0,0305973	Unpaired t-te	0,913003753	permutation FDR (250 perm	1
516	Ccdc93~Q7T	Ccdc93	ko vs wt	ko	wt	27,4890132	0,21820748	26,7306115	0,65390758	1,69161557	0,75840174	0,51269758	0,23819972	0,62305875	Unpaired t-te	0,27914945	permutation FDR (250 perm	1
517	Ccm2~Q8K2	Ccm2	ko vs wt	ko	wt	24,0270195	0,48802255	24,5035032	0,5807446	0,7187273	-0,4764836	-0,3180763	0,39593964	0,40237101	Unpaired t-te	0,467558647	permutation FDR (250 perm	1
518	Ccn1~P1840	Ccn1	ko vs wt	ko	wt	27,4370862	1,21238307	23,868862	0,51218611	11,8615794	3,56822421	1,99552304	0,00869293	2,06083362	Unpaired t-te	0,003344383	permutation FDR (250 perm	1
519	Ccn2~P2926	Ccn2	ko vs wt	ko	wt	31,2986245	0,5741041	31,8772404	0,20545059	0,6696059	-0,5786159	-0,4248599	0,18440656	0,73422364	Unpaired t-te	0,339031132	permutation FDR (250 perm	1
520	Ccn3~Q6429	Ccn3	ko vs wt	ko	wt	25,6025652	1,59441388	28,2711686	0,16827842	0,15727886	-2,6686034	-1,3839911	0,06134127	1,21224726	Unpaired t-te	0,015947205	permutation FDR (250 perm	1
521	Ccn5~A2AHD	Ccn5	ko vs wt	ko	wt	30,8268543	0,46289565	31,687005	0,0883276	0,55089502	-0,8601507	-0,674917	0,04519344	1,34492462	Unpaired t-te	0,158880652	permutation FDR (250 perm	1
522	Ccny~Q8BGL	Ccny	ko vs wt	ko	wt	23,8795991	0,64986379	25,101392	0,42289193	0,42874955	-1,2217929	-0,825656	0,05150832	1,28812263	Unpaired t-te	0,086761538	permutation FDR (250 perm	1
523	Ccs~Q9WU8	Ccs	ko vs wt	ko	wt	24,8971329	1,24531112	24,1377028	0,22076626	1,6928217	0,75943003	0,43752714	0,37201595	0,42943844	Unpaired t-te	0,333171301	permutation FDR (250 perm	1
524	Cct2~P80314	Cct2	ko vs wt	ko	wt	31,5251395	0,339417	31,5300962	0,16068166	0,99657022	-0,0049566	-0,0040412	0,98346318	0,0072419	Unpaired t-te	0,992931008	permutation FDR (250 perm	1
525	Cct3~P80318	Cct3	ko vs wt	ko	wt	32,6182855	0,33084158	32,3623853	0,28203069	1,19408056	0,25590017	0,20052594	0,39908691	0,39893252	Unpaired t-te	0,642699444	permutation FDR (250 perm	1
526	Cct4~P80315	Cct4	ko vs wt	ko	wt	32,0389481	0,10625303	32,0454303	0,34308826	0,99551696	-0,0064822	-0,0051848	0,98144442	0,00813429	Unpaired t-te	0,991150221	permutation FDR (250 perm	1
527	Cct5~P80316	Cct5	ko vs wt	ko	wt	31,5825936	0,22600675	31,369682	0,22764895	1,15902494	0,21291161	0,17636583	0,3589006	0,44502581	Unpaired t-te	0,686033391	permutation FDR (250 perm	1
528	Cct6a~P8031	Cct6a	ko vs wt	ko	wt	32,2506963	0,38225135	32,155013	0,35619306	1,06857137	0,09568328	0,07167951	0,78780109	0,10358342	Unpaired t-te	0,877100897	permutation FDR (250 perm	1
529	Cct6b~Q613	Cct6b	ko vs wt	ko	wt	28,6038107	0,74982356	28,4511975	0,73203773	1,11158104	0,15261313	0,09112334	0,8312977	0,08024342	Unpaired t-te	0,841085416	permutation FDR (250 perm	1
530	Cct7~P80313	Cct7	ko vs wt	ko	wt	31,9692342	0,13035071	32,0297627	0,2668248	0,95891279	-0,0605285	-0,0503092	0,78758056	0,10370501	Unpaired t-te	0,915706873	permutation FDR (250 perm	1
531	Cct8~P42932	Cct8	ko vs wt	ko	wt	32,3759197	0,30951524	31,9830718	0,08780176	1,31298262	0,39284782	0,33035279	0,11232094	0,94953928	Unpaired t-te	0,451987245	permutation FDR (250 perm	1
532	Ccz1~Q8C1Y	Ccz1	ko vs wt	ko	wt	27,4858668	0,44687963	24,803141	0,75694123	6,42067842	2,68272574	1,68282729	0,02132655	1,67107939	Unpaired t-te	0,00759751	permutation FDR (250 perm	1
533	Cd109~Q8R4	Cd109	ko vs wt	ko	wt	28,9113922	0,27604484	29,1916007	0,51472942	0,82347199	-0,2802085	-0,200531	0,53529141	0,27140972	Unpaired t-te	0,655564103	permutation FDR (250 perm	1
534	Cd163~Q2VL	Cd163	ko vs wt	ko	wt	26,0602417	0,55319557	25,1928332	1,58662754	1,82438285	0,86740851	0,4003748	0,52495149	0,27988083	Unpaired t-te	0,388517756	permutation FDR (250 perm	1
535	Cd180~Q621	Cd180	ko vs wt	ko	wt	25,2210514	1,24702496	24,4970089	0,8936093	1,6518041	0,7240426	0,36980044	0,48397692	0,31517535	Unpaired t-te	0,395312211	permutation FDR (250 perm	1
536	Cd200~E9PZ	Cd200	ko vs wt	ko	wt	28,1417285	0,85631965	29,7219636	0,18117945	0,33442738	-1,5802351	-1,0460106	0,04542314	1,34272287	Unpaired t-te	0,045260718	permutation FDR (250 perm	1
537	Cd2ap~Q9JL	Cd2ap	ko vs wt	ko	wt	25,5412311	1,3732946	27,1799049	0,15638856	0,32115156	-1,6386738	-0,910098	0,12975074	0,88689015	Unpaired t-te	0,075630739	permutation FDR (250 perm	1
538	Cd36~Q0885	Cd36	ko vs wt	ko	wt	29,6042335	0,23627776	29,7140732	0,54098346	0,92669099	-0,1098398	-0,078115	0,80745053	0,09288408	Unpaired t-te	0,87224659	permutation FDR (250 perm	1
539	Cd38~P5652	Cd38	ko vs wt	ko	wt	27,0775807	0,37160673	25,3474658	1,19297988	3,31754232	1,73011487	0,92498736	0,17028109	0,76883357	Unpaired t-te	0,084470019	permutation FDR (250 perm	1
540	Cd44~P1537	Cd44	ko vs wt	ko	wt	29,3925006	0,68778707	28,7520957	0,40306211	1,55876663	0,64040495	0,43015202	0,24789422	0,60573359	Unpaired t-te	0,327239931	permutation FDR (250 perm	1
541	Cd47~Q6173	Cd47	ko vs wt	ko	wt	24,9934388	0,45766621	23,9974617	0,62702734	1,99443088	0,99597713	0,65691608	0,13860897	0,85820866	Unpaired t-te	0,167425352	permutation FDR (250 perm	1
542	Cd55~F7BP0	Cd55	ko vs wt	ko	wt	28,7402187	0,38096161	29,3987017	0,63671538	0,63354414	-0,658483	-0,438673	0,28146672	0,55057295	Unpaired t-te	0,336284834	permutation FDR (250 perm	1
543	Cd55~Q6147	Cd55	ko vs wt	ko	wt	26,278584	0,65579548	26,3620672	0,19378786	0,94377626	-0,0834832	-0,0595179	0,84654286	0,07235105	Unpaired t-te	0,897436036	permutation FDR (250 perm	1
544	Cd5l~Q9QW	Cd5l	ko vs wt	ko	wt	31,7695581	0,42779503	31,2976343	1,43104828	1,38695772	0,47192381	0,23115271	0,69064774	0,16074341	Unpaired t-te	0,612404375	permutation FDR (250 perm	1
545	Cd74~P0444	Cd74	ko vs wt	ko	wt	24,4146524	0,98193601	25,0915157	0,50382988	0,62552384	-0,6768632	-0,4054121	0,36094298	0,4425614	Unpaired t-te	0,355803523	permutation FDR (250 perm	1
546	Cd93~O8910	Cd93	ko vs wt	ko	wt	24,7041682	1,3893687	24,1661412	0,63494896	1,45198546	0,53802701	0,28033127	0,5864016	0,23180486	Unpaired t-te	0,516623921	permutation FDR (250 perm	1
547	Cd9~P40240	Cd9	ko vs wt	ko	wt	29,1161187	0,78077617	27,3375184	1,51381208	3,4309314	1,77860028	0,82286527	0,23240183	0,63376046	Unpaired t-te	0,106554392	permutation FDR (250 perm	1
548	Cdc16~Q8R3	Cdc16	ko vs wt	ko	wt	27,835494	0,44779307	27,9419138	0,19947205	0,92889038	-0,1064197	-0,0822087	0,73431871	0,1341154	Unpaired t-te	0,858290093	permutation FDR (250 perm	1
549	Cdc23~Q8BG	Cdc23	ko vs wt	ko	wt	26,7965369	0,59273129	25,4130877	1,58082567	2,60891374	1,38344924	0,63782135	0,34137204	0,46677205	Unpaired t-te	0,192685537	permutation FDR (250 perm	1
550	Cdc37~Q610	Cdc37	ko vs wt	ko	wt	28,8057854	0,33800715	28,7805995	0,58421924	1,0176108	0,02518589	0,01728756	0,95961937	0,01790099	Unpaired t-te	0,970934452	permutation FDR (250 perm	1
551	Cdc42bpa~Q	Cdc42bpa	ko vs wt	ko	wt	29,6793796	0,57496634	29,6487293	0,21804624	1,02147247	0,03065033	0,02243775	0,93715239	0,02818978	Unpaired t-te	0,960655272	permutation FDR (250 perm	1
552	Cdc42bpb~Q	Cdc42bpb	ko vs wt	ko	wt	30,7457174	0,24571332	30,7243851	0,37378984	1,01489631	0,02133234	0,01640982	0,94759062	0,02337924	Unpaired t-te	0,971732844	permutation FDR (250 perm	1
553	Cdc42~P607	Cdc42	ko vs wt	ko	wt	31,4933911	0,25119442	31,1741764	0,63640509	1,2476512	0,31921466	0,21674019	0,5583803	0,25306991	Unpaired t-te	0,633867764	permutation FDR (250 perm	1
554	Cdc5l~Q6A0	Cdc5l	ko vs wt	ko	wt	27,0220933	0,31311862	26,4822197	0,28175805	1,45384518	0,53987365	0,42542369	0,10627823	0,97355571	Unpaired t-te	0,334469983	permutation FDR (250 perm	1
555	Cdc73~Q8JZ	Cdc73	ko vs wt	ko	wt	25,4568916	0,26622312	25,1162566	0,30029211	1,26631379	0,34063494	0,26988877	0,264824	0,57704266	Unpaired t-te	0,535529052	permutation FDR (250 perm	1
556	Cdh13~Q9W	Cdh13	ko vs wt	ko	wt	28,4042378	0,46308697	27,6616869	1,42986422	1,67313163	0,74255095	0,3629601	0,54325496	0,2649963	Unpaired t-te	0,431806264	permutation FDR (250 perm	1
557	Cdh1~P0980	Cdh1	ko vs wt	ko	wt	25,433149	1,72728507	26,6770881	0,08183042	0,42221827	-1,2439391	-0,6223038	0,30090232	0,52157446	Unpaired t-te	0,190510688	permutation FDR (250 perm	1
558	Cdk11b~P24	Cdk11b	ko vs wt	ko	wt	26,84559	0,56081552	26,8734623	0,29507115	0,98086578	-0,0278724	-0,0201217	0,94524847	0,02445402	Unpaired t-te	0,964605201	permutation FDR (250 perm	1
559	Cdk17~Q8K0	Cdk17	ko vs wt	ko	wt	24,9149657	0,41819267	25,4509377	0,08266753	0,68969385	-0,535972	-0,4293206	0,11053756	0,95649012	Unpaired t-te	0,340918346	permutation FDR (250 perm	1
560	Cdk4~P3028	Cdk4	ko vs wt	ko	wt	25,6412785	0,99304636	23,8875453	0,37994853	3,37230088	1,75373326	1,07382687	0,04817428	1,31718478	Unpaired t-te	0,036527473	permutation FDR (250 perm	1
561	Cdk5rap3~Q	Cdk5rap3	ko vs wt	ko	wt	25,2438505	1,28942351	25,3947296	1,52630215	0,90070147	-0,1508791	-0,0652843	0,9142282	0,03894539	Unpaired t-te	0,888363341	permutation FDR (250 perm	1
562	Cdk5~P4961	Cdk5	ko vs wt	ko	wt	28,9683983	0,60009409	28,1597721	0,48501479	1,75154273	0,80862619	0,54361385	0,16026093	0,79517235	Unpaired t-te	0,227039223	permutation FDR (250 perm	1
563	Cdkl5~Q3UT	Cdkl5	ko vs wt	ko	wt	23,9152098	0,78709962	25,4145765	0,9750862	0,35370863	-1,4993667	-0,8212218	0,15031669	0,82299278	Unpaired t-te	0,096130719	permutation FDR (250 perm	1
564	Cdkn2aip~Q8	Cdkn2aip	ko vs wt	ko	wt	25,0804555	1,05454805	25,2037024	1,38474178	0,91811903	-0,1232469	-0,0572438	0,92072051	0,03587218	Unpaired t-te	0,902408394	permutation FDR (250 perm	1
565	Cds2~Q99L4	Cds2	ko vs wt	ko	wt	26,9797338	0,33017624	26,6637472	0,11353302	1,24486271	0,31598665	0,26182932	0,20181938	0,69503713	Unpaired t-te	0,546974172	permutation FDR (250 perm	1
566	Celf1~P2865	Celf1	ko vs wt	ko	wt	28,5550118	0,209914	27,3824539	0,24276357	2,25410999	1,17255791	0,96895122	0,00564774	2,24812524	Unpaired t-te	0,056244009	permutation FDR (250 perm	1
567	Celf2~Q9Z0	Celf2	ko vs wt	ko	wt	28,3509398	0,38747186	28,9904966	0,55048013	0,64191011	-0,6395568	-0,4413923	0,24160879	0,61688727	Unpaired t-te	0,328958018	permutation FDR (250 perm	1
568	Cemip~Q8BI	Cemip	ko vs wt	ko	wt	27,7903381	1,26601922	24,9346587	0,8715131	7,23844326	2,85567946	1,45991988	0,03059245	1,51438578	Unpaired t-te	0,008832685	permutation FDR (250 perm	1
569	Cenpv~Q9CX	Cenpv	ko vs wt	ko	wt	29,100947	0,53565307	29,7180511	0,30983122	0,65197834	-0,6171041	-0,4475019	0,16545299	0,78132538	Unpaired t-te	0,309770492	permutation FDR (250 perm	1
570	Cep135~Q6P	Cep135	ko vs wt	ko	wt	26,5281204	0,12341674	26,4223119	0,1003597	1,07609731	0,10580855	0,09614023	0,3427143	0,46506777	Unpaired t-te	0,831769506	permutation FDR (250 perm	1
571	Cep170b~Q8	Cep170b	ko vs wt	ko	wt	24,2692867	0,34569898	23,432447	0,65331341	1,78613324	0,8368397	0,55669266	0,20302243	0,69245597	Unpaired t-te	0,237377679	permutation FDR (250 perm	1
572	Cep68~Q8C0	Cep68	ko vs wt	ko	wt	24,3597062	0,35684095	24,9065849	0,48211977	0,68449944	-0,5468787	-0,3910949	0,25281507	0,59719705	Unpaired t-te	0,382115372	permutation FDR (250 perm	1
573	Cep97~Q9CZ	Cep97	ko vs wt	ko	wt	24,9369194	0,59306265	24,4408896	0,88137083	1,4103271	0,49602981	0,2898912	0,53281756	0,27342147	Unpaired t-te	0,512902349	permutation FDR (250 perm	1

574	Cept1~Q8BG	Cept1	ko vs wt	ko	wt	25,3661849	1,64537151	24,9912916	0,85086114	1,29674365	0,37489331	0,17646567	0,7530585	0,12317128	Unpaired t-te	0,684024207	permutation FDR (250 perm	1
575	Cercam~A3K	Cercam	ko vs wt	ko	wt	23,7474075	0,49325708	23,102811	0,46993592	1,56330206	0,64459656	0,44837419	0,20808614	0,68175684	Unpaired t-te	0,312033438	permutation FDR (250 perm	1
576	Cert1~Q9EQ(	Cert1	ko vs wt	ko	wt	25,7691111	0,92955733	24,6681474	0,97948241	2,14497922	1,10096367	0,58680649	0,27530028	0,56019335	Unpaired t-te	0,201419256	permutation FDR (250 perm	1
577	Ces1c~P2395	Ces1c	ko vs wt	ko	wt	24,1045001	0,90974323	24,7350628	0,12576832	0,64592444	-0,6305627	-0,4114021	0,31763627	0,49806991	Unpaired t-te	0,362866982	permutation FDR (250 perm	1
578	Ces1d~Q8VC	Ces1d	ko vs wt	ko	wt	27,4488663	0,40924738	26,8331948	0,68626852	1,53227101	0,61567149	0,39985641	0,33806892	0,47099475	Unpaired t-te	0,378721553	permutation FDR (250 perm	1
579	Ces3a~Q638	Ces3a	ko vs wt	ko	wt	24,5141132	0,4155272	24,4295342	0,55837311	1,06037822	0,08457895	0,05785151	0,86502112	0,06297329	Unpaired t-te	0,901795962	permutation FDR (250 perm	1
580	Cfhr1~Q614(	Cfhr1	ko vs wt	ko	wt	25,0388204	0,95563715	24,6737233	1,23820172	1,28796825	0,36509703	0,17941904	0,74427189	0,12826839	Unpaired t-te	0,684729683	permutation FDR (250 perm	1
581	Cfhr2~Q4LDF	Cfhr2	ko vs wt	ko	wt	25,1868891	1,40511905	27,210612	0,00436099	0,24592275	-2,0237229	-1,1173063	0,08812543	1,05489875	Unpaired t-te	0,037977011	permutation FDR (250 perm	1
582	Cfhr4~E9Q8E	Cfhr4	ko vs wt	ko	wt	30,7965648	0,16100942	29,7077354	0,17651195	2,1270138	1,08882939	0,94219844	0,00217324	2,66289261	Unpaired t-te	0,060938667	permutation FDR (250 perm	1
583	Cfh~P06909	Cfh	ko vs wt	ko	wt	32,4349249	0,10024568	32,314135	0,30724559	1,0873301	0,12078999	0,09861755	0,63885462	0,19459796	Unpaired t-te	0,837030553	permutation FDR (250 perm	1
584	Cfl1~P18760	Cfl1	ko vs wt	ko	wt	31,7227283	0,54526371	31,1887925	0,26008426	1,44787374	0,5339358	0,3912792	0,20792686	0,68208941	Unpaired t-te	0,372948993	permutation FDR (250 perm	1
585	Cfl2~P45591	Cfl2	ko vs wt	ko	wt	27,242592	1,07622502	26,5414464	0,90002842	1,62579522	0,70114555	0,37108546	0,46800571	0,32974885	Unpaired t-te	0,39504655	permutation FDR (250 perm	1
586	Cfp~P11680	Cfp	ko vs wt	ko	wt	25,5077061	1,14445399	23,5499684	0,34528238	3,88452372	1,95773772	1,14862684	0,0532969	1,27329805	Unpaired t-te	0,029741726	permutation FDR (250 perm	1
587	Cgnl1~B2RS(	Cgnl1	ko vs wt	ko	wt	24,9580768	0,90608054	23,9376505	0,68053833	2,02851837	1,02042636	0,59646392	0,21138886	0,67491791	Unpaired t-te	0,190111241	permutation FDR (250 perm	1
588	Chadl~E9Q7	Chadl	ko vs wt	ko	wt	25,3950052	1,16646314	24,8764109	0,65877895	1,43255874	0,5185943	0,28511969	0,55501047	0,25569882	Unpaired t-te	0,507659232	permutation FDR (250 perm	1
589	Chad~O5522	Chad	ko vs wt	ko	wt	31,4468401	1,67117477	29,7336268	3,81160778	3,2789032	1,71321331	0,44352606	0,59902103	0,22255793	Unpaired t-te	0,339077975	permutation FDR (250 perm	1
590	Chchd3~Q9C	Chchd3	ko vs wt	ko	wt	25,4255802	0,86082905	25,0887237	0,47983333	1,26300163	0,3368565	0,21030248	0,60043045	0,22153729	Unpaired t-te	0,625441272	permutation FDR (250 perm	1
591	Chd4~Q6PDC	Chd4	ko vs wt	ko	wt	30,4026582	0,33373467	29,9677859	0,24367279	1,35179116	0,43487229	0,34555222	0,15379799	0,81304933	Unpaired t-te	0,426078597	permutation FDR (250 perm	1
592	Cherp~Q8CG	Cherp	ko vs wt	ko	wt	27,6920905	0,379745	27,6556099	0,37045613	1,02560884	0,03648061	0,02719195	0,91969019	0,03635844	Unpaired t-te	0,951929325	permutation FDR (250 perm	1
593	Chil3~O3574	Chil3	ko vs wt	ko	wt	25,370927	2,05249067	23,8650837	0,49335773	2,83990633	1,50584335	0,67366814	0,29850796	0,52504408	Unpaired t-te	0,157499796	permutation FDR (250 perm	1
594	Chmp3~Q9C(	Chmp3	ko vs wt	ko	wt	27,8650901	0,19926242	27,247908	0,756524	1,53387633	0,61718217	0,39890933	0,36786103	0,43431621	Unpaired t-te	0,393750579	permutation FDR (250 perm	1
595	Chmp4b~Q9I	Chmp4b	ko vs wt	ko	wt	25,0717131	1,19069005	24,752335	2,25832394	1,24779253	0,31937808	0,11662259	0,86695723	0,06200233	Unpaired t-te	0,802184958	permutation FDR (250 perm	1
596	Chmp5~Q9D	Chmp5	ko vs wt	ko	wt	27,5653325	0,29410912	27,2707823	0,39140267	1,22650247	0,29455013	0,22235199	0,42299276	0,37366707	Unpaired t-te	0,612151345	permutation FDR (250 perm	1
597	Chordc1~Q9I	Chordc1	ko vs wt	ko	wt	24,9456892	0,71628891	25,9576172	0,13586668	0,49588311	-1,011928	-0,7103431	0,08901541	1,0505348	Unpaired t-te	0,14286745	permutation FDR (250 perm	1
598	Chrdl1~Q920	Chrdl1	ko vs wt	ko	wt	29,492206	1,30015908	29,2665539	0,70022482	1,16930565	0,22565209	0,11881192	0,8122237	0,09032434	Unpaired t-te	0,78920938	permutation FDR (250 perm	1
599	Chrd~Q9Z0E	Chrd	ko vs wt	ko	wt	24,490958	0,82780804	23,5237048	0,30676977	1,95511461	0,96725318	0,63432346	0,1373475	0,86217924	Unpaired t-te	0,173165405	permutation FDR (250 perm	1
600	Chst14~Q80	Chst14	ko vs wt	ko	wt	25,3525254	1,18433106	24,9356231	1,06322286	1,33505791	0,41690232	0,20677094	0,70008955	0,15484641	Unpaired t-te	0,63296268	permutation FDR (250 perm	1
601	Cilp2~D3Z7H	Cilp2	ko vs wt	ko	wt	29,0247941	1,14459264	29,822624	0,53671344	0,57521377	-0,7978299	-0,4527837	0,34740306	0,45916635	Unpaired t-te	0,306456803	permutation FDR (250 perm	1
602	Cilp~Q66K08	Cilp	ko vs wt	ko	wt	26,5836018	0,73963957	24,5472035	0,31556118	4,10220127	2,03639828	1,37426013	0,0110805	1,9554407	Unpaired t-te	0,012275168	permutation FDR (250 perm	1
603	Cisd1~Q91W	Cisd1	ko vs wt	ko	wt	29,1903615	0,56521884	29,5101178	0,40436585	0,8012052	-0,3197563	-0,2230017	0,49439599	0,30592506	Unpaired t-te	0,602154211	permutation FDR (250 perm	1
604	Cisd2~Q9CQI	Cisd2	ko vs wt	ko	wt	24,6812864	1,2324813	24,0357238	0,25201015	1,56434925	0,64556264	0,37239407	0,43711456	0,35940473	Unpaired t-te	0,403770039	permutation FDR (250 perm	1
605	Cisd3~B1AR1	Cisd3	ko vs wt	ko	wt	29,1968062	0,49947548	28,8702306	0,12982369	1,25403322	0,32657556	0,25070453	0,34774898	0,45873414	Unpaired t-te	0,565832982	permutation FDR (250 perm	1
606	Ckap4~Q8BM	Ckap4	ko vs wt	ko	wt	31,3218349	0,2593361	31,1489123	0,56494349	1,12733995	0,17292263	0,12121198	0,716587	0,14473107	Unpaired t-te	0,794875	permutation FDR (250 perm	1
607	Ckap5~Z4YL7	Ckap5	ko vs wt	ko	wt	31,0203321	0,11341996	30,950548	0,41429034	1,04955956	0,06978404	0,05367272	0,8360204	0,07778312	Unpaired t-te	0,91275409	permutation FDR (250 perm	1
608	Ckb~Q04447	Ckb	ko vs wt	ko	wt	29,1259289	0,2267997	28,9730126	0,30219227	1,11181467	0,15291632	0,12227324	0,57952978	0,23692424	Unpaired t-te	0,78778765	permutation FDR (250 perm	1
609	Ckmt2~Q6P8	Ckmt2	ko vs wt	ko	wt	26,3179161	1,30606583	24,5694708	0,27532059	3,35996299	1,74844534	0,98294457	0,10017395	0,9992452	Unpaired t-te	0,056988568	permutation FDR (250 perm	1
610	Ckm~P07310	Ckm	ko vs wt	ko	wt	29,994431	0,78795208	29,5472639	0,54250708	1,36336058	0,44716717	0,28034258	0,48625886	0,31313247	Unpaired t-te	0,514338272	permutation FDR (250 perm	1
611	Clasp1~Q80T	Clasp1	ko vs wt	ko	wt	27,7592706	0,17285726	27,9082061	0,21398631	0,9019157	-0,1489355	-0,126082	0,46144	0,33588477	Unpaired t-te	0,778941242	permutation FDR (250 perm	1
612	Clasp2~Q8BI	Clasp2	ko vs wt	ko	wt	27,92493	0,35395082	28,5084488	0,6954231	0,66733414	-0,5835188	-0,3807601	0,36128131	0,4421545	Unpaired t-te	0,402833114	permutation FDR (250 perm	1
613	Clca3a1~Q9(	Clca3a1	ko vs wt	ko	wt	24,5114271	1,36164127	24,0795891	0,9092274	1,34895111	0,43183807	0,21425176	0,688356	0,1621869	Unpaired t-te	0,617507965	permutation FDR (250 perm	1
614	Clec11a~O8	Clec11a	ko vs wt	ko	wt	29,2459958	0,61437306	28,7925835	0,46632552	1,36927508	0,4534123	0,30547186	0,39282391	0,40580208	Unpaired t-te	0,478651982	permutation FDR (250 perm	1
615	Clec1b~Q9JL	Clec1b	ko vs wt	ko	wt	25,4012958	2,374027	24,0727256	0,74594081	2,5115365	1,32857024	0,53818061	0,41895238	0,37783534	Unpaired t-te	0,238170669	permutation FDR (250 perm	1
616	Clec3a~Q9EF	Clec3a	ko vs wt	ko	wt	27,4720886	0,62763014	28,056755	0,09291032	0,66680352	-0,5846664	-0,4273034	0,20505416	0,68813141	Unpaired t-te	0,344844687	permutation FDR (250 perm	1
617	Clec3b~Q8CF	Clec3b	ko vs wt	ko	wt	24,7258206	1,73769837	24,143045	0,45676991	1,4977279	0,58277555	0,28373138	0,61285904	0,2126394	Unpaired t-te	0,517908166	permutation FDR (250 perm	1
618	Clic1~Q9Z1Q	Clic1	ko vs wt	ko	wt	30,8205248	0,27027456	30,5772493	0,27343861	1,18367704	0,2432755	0,19486003	0,37989432	0,4203372	Unpaired t-te	0,654507724	permutation FDR (250 perm	1
619	Clic4~Q9QYB	Clic4	ko vs wt	ko	wt	31,4057183	0,44206961	31,3943204	0,26870353	1,0079317	0,01139788	0,00864664	0,97282139	0,01196689	Unpaired t-te	0,985733681	permutation FDR (250 perm	1
620	Clint1~Q5SU	Clint1	ko vs wt	ko	wt	27,4811295	0,42210329	28,224875	0,16185084	0,59718692	-0,7437455	-0,5859787	0,04849735	1,31428198	Unpaired t-te	0,201625767	permutation FDR (250 perm	1
621	Clip1~Q922J	Clip1	ko vs wt	ko	wt	24,903626	0,74472528	24,600779	0,81486777	1,23357636	0,30284702	0,17618229	0,69514219	0,15792635	Unpaired t-te	0,687460299	permutation FDR (250 perm	1
622	Clptm1l~Q8E	Clptm1l	ko vs wt	ko	wt	25,5281414	2,16165567	24,0402046	0,41313007	2,80487552	1,48793674	0,65209883	0,32225806	0,49179621	Unpaired t-te	0,170702443	permutation FDR (250 perm	1
623	Clpx~Q9JHS4	Clpx	ko vs wt	ko	wt	25,202219	2,05499799	26,9383308	0,08585234	0,30017759	-1,7361118	-0,7934677	0,23961105	0,62049315	Unpaired t-te	0,112862921	permutation FDR (250 perm	1
624	Cltc~Q68FD5	Cltc	ko vs wt	ko	wt	34,7358483	0,14577152	34,6956575	0,11291692	1,02824978	0,04019077	0,03601292	0,74341911	0,12876628	Unpaired t-te	0,937098726	permutation FDR (250 perm	1
625	Clu~Q06890	Clu	ko vs wt	ko	wt	33,9913801	0,10153064	33,5593367	0,25335972	1,34914307	0,43204335	0,36352028	0,12615956	0,89907984	Unpaired t-te	0,428274522	permutation FDR (250 perm	1
626	Clybl~Q8R4N	Clybl	ko vs wt	ko	wt	25,0473445	0,6684164	24,6516046	1,21152098	1,31561727	0,39573985	0,20403335	0,70367532	0,15262768	Unpaired t-te	0,648340723	permutation FDR (250 perm	1
627	Cma1~A4QP	Cma1	ko vs wt	ko	wt	29,664951	0,70200309	29,8968474	1,22177046	0,85151488	-0,2318964	-0,1186614	0,82424942	0,08394135	Unpaired t-te	0,79767364	permutation FDR (250 perm	1
628	Cmas~Q99K	Cmas	ko vs wt	ko	wt	29,2274417	0,11739239	29,2663149	0,28493214	0,97341489	-0,0388733	-0,0320585	0,86891097	0,06102472	Unpaired t-te	0,946989151	permutation FDR (250 perm	1
629	Cmpk1~Q9DI	Cmpk1	ko vs wt	ko	wt	27,3688114	0,59440692	27,1217867	0,48487038	1,18675709	0,24702467	0,16633528	0,63312202	0,19851258	Unpaired t-te	0,702357122	permutation FDR (250 perm	1
630	Cmtr1~Q9DB	Cmtr1	ko vs wt	ko	wt	26,4530684	0,42600764	26,433955	0,1877856	1,01333655	0,0191134	0,01493806	0,94845365	0,02298389	Unpaired t-te	0,973302277	permutation FDR (250 perm	1
631	Cnbp~P5399	Cnbp	ko vs wt	ko	wt	24,2198601	0,59844267	25,2911484	1,20048457	0,47589383	-1,0712883	-0,5589837	0,33618956	0,47341577	Unpaired t-te	0,237098706	permutation FDR (250 perm	1
632	Cndp2~Q9D1	Cndp2	ko vs wt	ko	wt	26,6970253	1,02800996	24,723149	0,71420726	3,92822158	1,97387631	1,10935327	0,05242559	1,28045671	Unpaired t-te	0,02965257	permutation FDR (250 perm	1
633	Cnmd~G5E8	Cnmd	ko vs wt	ko	wt	28,8834805	0,42085641	28,0323491	0,80141439	1,80391497	0,85113134	0,52650078	0,26925346	0,5698387	Unpaired t-te	0,259838209	permutation FDR (250 perm	1
634	Cnn1~Q0809	Cnn1	ko vs wt	ko	wt	33,7167785	0,27739565	34,0942152	0,11493103	0,76980412	-0,3774367	-0,3199717	0,09840391	1,00698766	Unpaired t-te	0,461253807	permutation FDR (250 perm	1
635	Cnn2~Q0809	Cnn2	ko vs wt	ko	wt	31,3142957	0,15773612	31,4939175	0,04890401	0,88293442	-0,1796218	-0,1636774	0,14255179	0,84602733	Unpaired t-te	0,711024307	permutation FDR (250 perm	1
636	Cnn3~Q9DA	Cnn3	ko vs wt	ko	wt	31,2051095	0,56243589	31,8449684	0,04148573	0,64177571	-0,6398589	-0,4825319	0,14302919	0,84457533	Unpaired t-te	0,291662447	permutation FDR (250 perm	1
637	Cnnm3~Q32I	Cnnm3	ko vs wt	ko	wt	24,5731689	0,33993191	24,3265582	0,75039541	1,18641664	0,24661074	0,15750402	0,69707799	0,15671863	Unpaired t-te	0,730803293	permutation FDR (250 perm	1

638	Cnot10~Q8B	Cnot10	ko vs wt	ko	wt	26,4472138	0,41226009	26,2695322	0,10348055	1,13106475	0,17768153	0,14225768	0,51963036	0,28430548	Unpaired t-te	0,749467155	permutation FDR (250 perm	1
639	Cnot1~Q6ZQ	Cnot1	ko vs wt	ko	wt	27,7708569	0,37480557	28,1828507	0,53588127	0,75158396	-0,4119938	-0,2868317	0,40922695	0,38803577	Unpaired t-te	0,516127004	permutation FDR (250 perm	1
640	Cnot3~Q8K0	Cnot3	ko vs wt	ko	wt	24,3087294	0,20596092	24,0691499	0,68984609	1,18064845	0,23957945	0,15949852	0,67579801	0,17018309	Unpaired t-te	0,730974737	permutation FDR (250 perm	1
641	Cnpy2~Q9QX	Cnpy2	ko vs wt	ko	wt	25,3493919	0,80584919	24,3821919	0,77625317	1,95504265	0,96720008	0,56247356	0,24410213	0,61242844	Unpaired t-te	0,215244998	permutation FDR (250 perm	1
642	Cnp~P16330	Cnp	ko vs wt	ko	wt	28,3233178	0,57738652	28,3040498	0,53225687	1,01344512	0,01926797	0,01282167	0,97106492	0,01275173	Unpaired t-te	0,977560669	permutation FDR (250 perm	1
643	Coasy~Q9DB	Coasy	ko vs wt	ko	wt	26,5513283	0,87887907	27,0487831	1,07465804	0,70835535	-0,4974548	-0,2599385	0,61735453	0,20946536	Unpaired t-te	0,55185801	permutation FDR (250 perm	1
644	Cobll1~Q3U	Cobll1	ko vs wt	ko	wt	27,2715067	0,51421245	27,4537622	0,26672578	0,88132405	-0,1822555	-0,1348319	0,62755354	0,20234922	Unpaired t-te	0,758881575	permutation FDR (250 perm	1
645	Cog1~Q9Z16	Cog1	ko vs wt	ko	wt	27,5006127	0,46628348	27,083888	0,10275713	1,33489359	0,41672475	0,32586098	0,22084417	0,65591406	Unpaired t-te	0,460429206	permutation FDR (250 perm	1
646	Cog2~Q921L	Cog2	ko vs wt	ko	wt	25,1431399	1,72025084	24,3251516	0,22251853	1,76294597	0,81798826	0,40785752	0,47292905	0,32520401	Unpaired t-te	0,366583249	permutation FDR (250 perm	1
647	Cog3~E9QL6	Cog3	ko vs wt	ko	wt	25,2876675	1,48679777	25,9030619	0,13296716	0,65275141	-0,6153944	-0,3302293	0,52639008	0,27869231	Unpaired t-te	0,458452842	permutation FDR (250 perm	1
648	Cog4~Q8R1L	Cog4	ko vs wt	ko	wt	26,3511506	0,54486169	24,9266581	0,98119805	2,68420055	1,42449247	0,80854571	0,16386505	0,78551367	Unpaired t-te	0,109807501	permutation FDR (250 perm	1
649	Cog7~Q3UM	Cog7	ko vs wt	ko	wt	26,7643563	0,4806269	26,4024742	0,26139782	1,28510132	0,36188211	0,27139509	0,32895634	0,48286174	Unpaired t-te	0,527625882	permutation FDR (250 perm	1
650	Cog8~Q9JJA	Cog8	ko vs wt	ko	wt	24,6634096	0,44397442	23,6096469	0,33797959	2,0759371	1,05376273	0,78030132	0,03052967	1,51527789	Unpaired t-te	0,103118052	permutation FDR (250 perm	1
651	Col10a1~Q0	Col10a1	ko vs wt	ko	wt	30,9545349	0,66606229	32,3010549	0,1882373	0,39323944	-1,3465201	-0,957059	0,03357702	1,4739579	Unpaired t-te	0,059749465	permutation FDR (250 perm	1
652	Col11a2~Q6	Col11a2	ko vs wt	ko	wt	26,4280519	0,69908715	26,5632791	0,43715758	0,91052645	-0,1352272	-0,08965	0,80090523	0,09641887	Unpaired t-te	0,842730368	permutation FDR (250 perm	1
653	Col12a1~Q6	Col12a1	ko vs wt	ko	wt	36,0007386	0,35861537	34,4301253	1,35166137	2,97030968	1,57061335	0,79406605	0,23880696	0,62195303	Unpaired t-te	0,12742311	permutation FDR (250 perm	1
654	Col14a1~B7	Col14a1	ko vs wt	ko	wt	34,5726487	0,45761776	34,064509	0,73960047	1,42221505	0,50813963	0,32040567	0,44922753	0,34753364	Unpaired t-te	0,471605421	permutation FDR (250 perm	1
655	Col15a1~O3	Col15a1	ko vs wt	ko	wt	34,1308572	0,26938858	33,6646219	0,4987159	1,3814997	0,46623525	0,33652981	0,31904454	0,49614869	Unpaired t-te	0,453933142	permutation FDR (250 perm	1
656	Col16a1~Q8	Col16a1	ko vs wt	ko	wt	31,0745353	0,28474828	31,3922569	0,166241	0,80233601	-0,3177215	-0,264305	0,17765479	0,75042307	Unpaired t-te	0,538716774	permutation FDR (250 perm	1
657	Col18a1~E9	Col18a1	ko vs wt	ko	wt	34,7919309	0,51249025	34,8492435	0,13479437	0,96105264	-0,0573126	-0,0437214	0,86364439	0,06366505	Unpaired t-te	0,925977588	permutation FDR (250 perm	1
658	Col19a1~Q0	Col19a1	ko vs wt	ko	wt	24,550562	1,03885529	26,3289693	0,48738679	0,29150504	-1,7784073	-1,0512257	0,05451821	1,26345837	Unpaired t-te	0,038039879	permutation FDR (250 perm	1
659	Col1a1~P110	Col1a1	ko vs wt	ko	wt	35,2711418	0,54381	35,7033284	0,50459761	0,74113767	-0,4321865	-0,2929533	0,40924843	0,38801297	Unpaired t-te	0,498100809	permutation FDR (250 perm	1
660	Col1a2~Q01	Col1a2	ko vs wt	ko	wt	35,809643	0,57013417	36,2292803	0,44429355	0,74761258	-0,4196373	-0,2884052	0,39970813	0,39825702	Unpaired t-te	0,50304472	permutation FDR (250 perm	1
661	Col28a1~Q2	Col28a1	ko vs wt	ko	wt	25,4439737	1,51956628	26,8489929	0,4029356	0,3776131	-1,4050193	-0,7308568	0,20995163	0,67788075	Unpaired t-te	0,131434483	permutation FDR (250 perm	1
662	Col2a1~P284	Col2a1	ko vs wt	ko	wt	30,6655859	1,11113814	30,4495062	1,44887315	1,16157295	0,21607976	0,09782747	0,86794656	0,06150701	Unpaired t-te	0,831620832	permutation FDR (250 perm	1
663	Col3a1~P081	Col3a1	ko vs wt	ko	wt	30,5759053	0,51546674	30,2125081	0,38288417	1,28645162	0,3633972	0,2591383	0,40826498	0,38905787	Unpaired t-te	0,546040742	permutation FDR (250 perm	1
664	Col4a1~P024	Col4a1	ko vs wt	ko	wt	34,091518	0,84925504	34,6014013	0,54599594	0,70227929	-0,5098832	-0,3139534	0,45111752	0,34571031	Unpaired t-te	0,46556962	permutation FDR (250 perm	1
665	Col4a2~P081	Col4a2	ko vs wt	ko	wt	34,7274884	0,45468648	35,1755132	0,0820081	0,7330458	-0,4480247	-0,3530974	0,18616947	0,73009153	Unpaired t-te	0,428322434	permutation FDR (250 perm	1
666	Col4a3~Q9Q	Col4a3	ko vs wt	ko	wt	28,4157433	0,48962792	28,7148754	0,68239322	0,81274116	-0,2991321	-0,1918456	0,62625335	0,20324994	Unpaired t-te	0,665382917	permutation FDR (250 perm	1
667	Col4a4~Q9Q	Col4a4	ko vs wt	ko	wt	28,6469959	0,0373796	28,8987121	0,36901974	0,8398967	-0,2517162	-0,1994855	0,43662669	0,35988972	Unpaired t-te	0,668394775	permutation FDR (250 perm	1
668	Col4a5~Q63	Col4a5	ko vs wt	ko	wt	30,7900076	1,60660168	30,7370356	1,15480204	1,0373998	0,052972	0,02369276	0,96747725	0,01435924	Unpaired t-te	0,957236191	permutation FDR (250 perm	1
669	Col4a6~B1A	Col4a6	ko vs wt	ko	wt	32,474211	0,84312315	32,935208	0,51461441	0,72648403	-0,460997	-0,2867334	0,48257388	0,31643619	Unpaired t-te	0,505162892	permutation FDR (250 perm	1
670	Col5a1~O88	Col5a1	ko vs wt	ko	wt	29,4434643	0,7880582	30,0821384	1,00940135	0,64230297	-0,6386741	-0,3458952	0,49730035	0,30338124	Unpaired t-te	0,434210187	permutation FDR (250 perm	1
671	Col5a2~Q3U	Col5a2	ko vs wt	ko	wt	30,2715174	0,31886219	29,9589864	0,66551794	1,24188451	0,31253102	0,20761769	0,58584711	0,23221571	Unpaired t-te	0,645636257	permutation FDR (250 perm	1
672	Col5a3~Q9JL	Col5a3	ko vs wt	ko	wt	24,9510048	1,21506492	23,5984351	0,51864806	2,55366568	1,35256966	0,75495204	0,15654821	0,80535189	Unpaired t-te	0,1160412	permutation FDR (250 perm	1
673	Col6a1~Q04	Col6a1	ko vs wt	ko	wt	37,4722839	0,36010161	37,7146943	0,09382893	0,8453318	-0,2424104	-0,198985	0,33553167	0,47426648	Unpaired t-te	0,650951298	permutation FDR (250 perm	1
674	Col6a2~D3Z	Col6a2	ko vs wt	ko	wt	24,8024316	0,91178411	25,1810874	0,22799558	0,76915388	-0,3786558	-0,2442071	0,53395804	0,27249287	Unpaired t-te	0,576809853	permutation FDR (250 perm	1
675	Col6a2~Q02	Col6a2	ko vs wt	ko	wt	37,0076608	0,36109965	37,3395362	0,08836513	0,79450302	-0,3318754	-0,2725556	0,21139968	0,67489567	Unpaired t-te	0,534578158	permutation FDR (250 perm	1
676	Col6a3~E9PV	Col6a3	ko vs wt	ko	wt	38,6365685	0,23409756	38,8130137	0,13340236	0,88488068	-0,1764452	-0,1514786	0,33431735	0,47584109	Unpaired t-te	0,728828174	permutation FDR (250 perm	1
677	Col6a4~A2A	Col6a4	ko vs wt	ko	wt	24,3992986	0,57298854	24,5716039	0,9415571	0,88742351	-0,1723053	-0,0988306	0,8316834	0,08004197	Unpaired t-te	0,832070543	permutation FDR (250 perm	1
678	Col6a5~A6H	Col6a5	ko vs wt	ko	wt	31,3402061	1,13344274	30,5848656	0,47633789	1,68802995	0,7553405	0,43510675	0,35847952	0,44553565	Unpaired t-te	0,325688491	permutation FDR (250 perm	1
679	Col6a6~Q8C	Col6a6	ko vs wt	ko	wt	33,8670739	0,59541241	34,2272032	0,08805938	0,77909475	-0,3601293	-0,2668899	0,37423861	0,42685141	Unpaired t-te	0,545984185	permutation FDR (250 perm	1
680	Col8a1~Q00	Col8a1	ko vs wt	ko	wt	30,1714871	0,59588385	29,1217056	0,11704684	2,07021631	1,04978152	0,77540513	0,05175583	1,28604069	Unpaired t-te	0,116429722	permutation FDR (250 perm	1
681	Col9a1~G3X	Col9a1	ko vs wt	ko	wt	28,732797	2,36407199	27,4162524	0,06842599	2,49068855	1,31654463	0,55650085	0,40600964	0,39146365	Unpaired t-te	0,233370453	permutation FDR (250 perm	1
682	Col9a2~Q07	Col9a2	ko vs wt	ko	wt	25,8441908	2,46244604	25,4564004	1,37861368	1,30838797	0,3877904	0,14237087	0,83114334	0,08032407	Unpaired t-te	0,744713923	permutation FDR (250 perm	1
683	Colec12~Q8	Colec12	ko vs wt	ko	wt	23,865889	0,63626838	25,5477373	0,98018196	0,31168308	-1,6818483	-0,942515	0,11798366	0,92817814	Unpaired t-te	0,068030864	permutation FDR (250 perm	1
684	Colgalt1~Q8	Colgalt1	ko vs wt	ko	wt	28,7981151	0,88544188	28,3401818	0,0914461	1,37357277	0,45793334	0,3022097	0,43778488	0,35873924	Unpaired t-te	0,496261601	permutation FDR (250 perm	1
685	Commd3~Q6	Commd3	ko vs wt	ko	wt	24,6490291	1,33130617	25,1954673	1,12007495	0,68470852	-0,5464381	-0,2597553	0,64261516	0,19204903	Unpaired t-te	0,546018851	permutation FDR (250 perm	1
686	Commd5~Q8	Commd5	ko vs wt	ko	wt	24,9966915	1,14072642	24,8323419	1,38278261	1,12066075	0,16434961	0,07542792	0,89635073	0,04752202	Unpaired t-te	0,872742842	permutation FDR (250 perm	1
687	Comp~Q9R0	Comp	ko vs wt	ko	wt	33,1301847	0,82362717	30,3161492	2,23229399	7,03248951	2,81403549	1,06248499	0,21021863	0,6773288	Unpaired t-te	0,054232301	permutation FDR (250 perm	1
688	Comt~O8858	Comt	ko vs wt	ko	wt	27,4459641	0,64043409	27,6741254	0,30035049	0,85372227	-0,2281613	-0,1599551	0,6176321	0,20927014	Unpaired t-te	0,714994286	permutation FDR (250 perm	1
689	Copa~Q8CIE	Copa	ko vs wt	ko	wt	32,6342192	0,38630554	32,4154732	0,26626651	1,16372162	0,21874599	0,16932399	0,48736641	0,31214441	Unpaired t-te	0,696383688	permutation FDR (250 perm	1
690	Copb1~Q9JIF	Copb1	ko vs wt	ko	wt	31,3968684	0,30517379	30,7211077	0,33185979	1,59743887	0,67576072	0,52245086	0,08209539	1,08568122	Unpaired t-te	0,248030679	permutation FDR (250 perm	1
691	Copb2~O550	Copb2	ko vs wt	ko	wt	31,6145722	0,35861847	31,4464369	0,40105828	1,12360527	0,16813529	0,12444041	0,65734103	0,18220926	Unpaired t-te	0,780872483	permutation FDR (250 perm	1
692	Cope~O8907	Cope	ko vs wt	ko	wt	28,5690336	0,52327577	28,8229441	0,22969309	0,83862021	-0,2539105	-0,1890616	0,49647303	0,30410434	Unpaired t-te	0,663901192	permutation FDR (250 perm	1
693	Copg1~Q9Q	Copg1	ko vs wt	ko	wt	30,9530654	0,32988906	30,7887277	0,39087126	1,12065154	0,16433775	0,12303887	0,65144855	0,18611988	Unpaired t-te	0,784693318	permutation FDR (250 perm	1
694	Copg2~Q9Q	Copg2	ko vs wt	ko	wt	28,2679759	0,49524442	28,1687633	0,27766321	1,07118867	0,0992126	0,07366276	0,78661085	0,10424007	Unpaired t-te	0,873443052	permutation FDR (250 perm	1
695	Cops2~P612	Cops2	ko vs wt	ko	wt	29,2759151	0,28640719	28,7732123	0,29627752	1,41686547	0,50270278	0,39679893	0,12957375	0,88748298	Unpaired t-te	0,369340504	permutation FDR (250 perm	1
696	Cops3~O885	Cops3	ko vs wt	ko	wt	24,2443582	0,72461386	25,3918969	0,94771018	0,45139466	-1,1475387	-0,6410833	0,22951805	0,63918315	Unpaired t-te	0,174421931	permutation FDR (250 perm	1
697	Cops4~O885	Cops4	ko vs wt	ko	wt	27,7767446	0,50878336	27,5351103	0,52739339	1,18233123	0,24163426	0,16385098	0,63645187	0,19623444	Unpaired t-te	0,709034621	permutation FDR (250 perm	1
698	Cops5~O358	Cops5	ko vs wt	ko	wt	27,7197927	0,18925177	27,4177377	0,21810391	1,23289932	0,30205499	0,25403984	0,18776552	0,72638415	Unpaired t-te	0,559016631	permutation FDR (250 perm	1
699	Cops6~O885	Cops6	ko vs wt	ko	wt	27,4624141	0,18169771	27,6168185	0,50883196	0,89850325	-0,1544044	-0,1123121	0,7150393	0,14567009	Unpaired t-te	0,811257412	permutation FDR (250 perm	1
700	Cops7a~Q9C	Cops7a	ko vs wt	ko	wt	25,3195181	1,09157213	24,8978332	0,4458219	1,33949098	0,42168487	0,24737086	0,57975564	0,23675502	Unpaired t-te	0,566499849	permutation FDR (250 perm	1
701	Copz1~P619	Copz1	ko vs wt	ko	wt	25,3642259	0,56937161	25,2665355	0,95890695	1,07005904	0,0976904	0,0557106	0,90520225	0,04325437	Unpaired t-te	0,906769148	permutation FDR (250 perm	1

702	Copz2~Q9JHI	Copz2	ko vs wt	ko	wt	27,3675961	0,20119254	25,7036684	1,28011171	3,16878032	1,66392764	0,86998277	0,20577583	0,68660564	Unpaired t-te	0,10459808	permutation FDR (250 perm	1
703	Coq10a~E9Q	Coq10a	ko vs wt	ko	wt	26,1151905	0,49078663	24,6965147	1,12304086	2,67340023	1,41867584	0,76970204	0,20831904	0,68127103	Unpaired t-te	0,129587549	permutation FDR (250 perm	1
704	Coq3~Q8BM	Coq3	ko vs wt	ko	wt	24,1343351	0,60319481	25,2358051	0,84754235	0,46604137	-1,1014701	-0,6505481	0,20136297	0,69602039	Unpaired t-te	0,17155058	permutation FDR (250 perm	1
705	Coq8a~Q609	Coq8a	ko vs wt	ko	wt	28,8071393	0,45134438	29,0114534	0,3080722	0,86795124	-0,2043141	-0,1525137	0,57371696	0,24130231	Unpaired t-te	0,726213636	permutation FDR (250 perm	1
706	Coro1a~O89	Coro1a	ko vs wt	ko	wt	30,174976	1,06753049	29,1679038	0,90432149	2,00982828	1,00707224	0,53337057	0,31106761	0,5071452	Unpaired t-te	0,234835556	permutation FDR (250 perm	1
707	Coro1b~Q9W	Coro1b	ko vs wt	ko	wt	30,7819151	0,2224032	30,8480049	0,14171074	0,95522346	-0,0660898	-0,0568331	0,70172599	0,15383244	Unpaired t-te	0,901247914	permutation FDR (250 perm	1
708	Coro1c~Q9W	Coro1c	ko vs wt	ko	wt	32,3940243	0,63826424	33,0071921	0,33192765	0,65375959	-0,6131679	-0,4267296	0,22230513	0,65305051	Unpaired t-te	0,331833734	permutation FDR (250 perm	1
709	Coro2b~Q8B	Coro2b	ko vs wt	ko	wt	24,6394028	0,61790358	25,1154033	0,75077825	0,71896802	-0,4760005	-0,2903131	0,50111639	0,3000614	Unpaired t-te	0,50745832	permutation FDR (250 perm	1
710	Coro7~Q9D2	Coro7	ko vs wt	ko	wt	25,6093114	0,76382882	24,4611026	0,61835812	2,21638547	1,14820881	0,7083264	0,12594272	0,89982694	Unpaired t-te	0,133247312	permutation FDR (250 perm	1
711	Cotl1~Q9CQI	Cotl1	ko vs wt	ko	wt	27,5820773	0,45746935	27,2906183	0,71526764	1,22387741	0,29145905	0,18557397	0,64378414	0,19125972	Unpaired t-te	0,678359954	permutation FDR (250 perm	1
712	Cox4i1~P197	Cox4i1	ko vs wt	ko	wt	30,0311664	0,4370456	30,1044577	0,27543625	0,95046713	-0,0732914	-0,0555763	0,82728051	0,08234721	Unpaired t-te	0,903013713	permutation FDR (250 perm	1
713	Cox5a~P1278	Cox5a	ko vs wt	ko	wt	28,8246031	1,38504187	25,0764557	0,64241216	13,4370765	3,74814738	1,95249525	0,01170528	1,93161804	Unpaired t-te	0,003579323	permutation FDR (250 perm	1
714	Cox5b~P1953	Cox5b	ko vs wt	ko	wt	28,0133983	0,60880464	28,171274	0,77149129	0,89634392	-0,1578757	-0,0957427	0,82104071	0,08563531	Unpaired t-te	0,835281938	permutation FDR (250 perm	1
715	Cox6a1~Q9D	Cox6a1	ko vs wt	ko	wt	26,1885912	0,22089106	26,7241276	1,20927059	0,68990214	-0,5355364	-0,2872215	0,59641047	0,22445474	Unpaired t-te	0,53347858	permutation FDR (250 perm	1
716	Cox6b1~P563	Cox6b1	ko vs wt	ko	wt	29,1625064	0,17028745	28,0131778	0,08322293	2,21810635	1,14932854	1,03117478	0,00025493	3,59357448	Unpaired t-te	0,04092819	permutation FDR (250 perm	1
717	Cox6c~Q9CP	Cox6c	ko vs wt	ko	wt	29,0458555	0,28968343	28,7724005	0,47484426	1,20869901	0,27345503	0,19885986	0,51888001	0,28493306	Unpaired t-te	0,656210956	permutation FDR (250 perm	1
718	Cox7a1~P563	Cox7a1	ko vs wt	ko	wt	28,5096524	0,50454204	28,3653317	0,44057196	1,10521012	0,14432068	0,10117086	0,74980206	0,12505337	Unpaired t-te	0,821806024	permutation FDR (250 perm	1
719	Cpa3~P1508	Cpa3	ko vs wt	ko	wt	31,3594555	0,96005763	31,8681013	1,08650895	0,70288191	-0,5086458	-0,2611978	0,62037594	0,20734505	Unpaired t-te	0,548303767	permutation FDR (250 perm	1
720	Cpb2~Q9JHH	Cpb2	ko vs wt	ko	wt	30,0926151	1,80818301	28,0538267	0,55216598	4,10900305	2,0387884	0,96415823	0,14537818	0,83750077	Unpaired t-te	0,05776477	permutation FDR (250 perm	1
721	Cpeb4~Q7TN	Cpeb4	ko vs wt	ko	wt	23,9230093	0,56466907	24,0679117	0,7611657	0,90444058	-0,1449024	-0,0889374	0,83094792	0,08042619	Unpaired t-te	0,847855144	permutation FDR (250 perm	1
722	Cpe~Q00493	Cpe	ko vs wt	ko	wt	25,6024956	1,21559053	25,2373325	1,22842948	1,28802721	0,36516307	0,17251329	0,75913217	0,1196826	Unpaired t-te	0,69301954	permutation FDR (250 perm	1
723	Cpn1~Q9JJN	Cpn1	ko vs wt	ko	wt	28,4303916	0,87986933	27,158372	0,14647233	2,41499396	1,27201958	0,83771277	0,08493151	1,07093114	Unpaired t-te	0,094516159	permutation FDR (250 perm	1
724	Cpn2~Q9DBE	Cpn2	ko vs wt	ko	wt	28,9490496	0,89151423	25,4296044	0,03157378	11,4672313	3,51944519	2,32275957	0,00634208	2,19776798	Unpaired t-te	0,003062992	permutation FDR (250 perm	1
725	Cpne1~Q8C1	Cpne1	ko vs wt	ko	wt	27,8603363	0,69113218	27,6981504	0,35593848	1,11898123	0,16218583	0,11019778	0,74574021	0,12741244	Unpaired t-te	0,805822024	permutation FDR (250 perm	1
726	Cpne2~P591	Cpne2	ko vs wt	ko	wt	24,8127194	1,11501893	24,5563582	0,29949283	1,19446221	0,25636122	0,15280562	0,72633787	0,13886131	Unpaired t-te	0,731462958	permutation FDR (250 perm	1
727	Cpne3~Q8BT	Cpne3	ko vs wt	ko	wt	29,6787953	0,10397597	29,5027782	0,28055364	1,12976056	0,17601704	0,14579816	0,47286565	0,32526224	Unpaired t-te	0,751585586	permutation FDR (250 perm	1
728	Cpq~Q9WVJ	Cpq	ko vs wt	ko	wt	24,7679595	0,64515981	25,1993367	1,77429226	0,74155353	-0,4313773	-0,1868453	0,76874341	0,1142186	Unpaired t-te	0,682962792	permutation FDR (250 perm	1
729	Cpsf2~O3521	Cpsf2	ko vs wt	ko	wt	25,8372771	1,44565688	24,0294422	0,21876088	3,50116464	1,8078349	0,97780869	0,1172769	0,93078752	Unpaired t-te	0,059238197	permutation FDR (250 perm	1
730	Cpsf3~Q9QX	Cpsf3	ko vs wt	ko	wt	25,1635467	1,16224882	24,6972753	1,65643261	1,38153429	0,46627137	0,19842403	0,75069039	0,12453914	Unpaired t-te	0,654152142	permutation FDR (250 perm	1
731	Cpsf6~Q6NV	Cpsf6	ko vs wt	ko	wt	27,32496	1,07710321	28,0143567	0,80952456	0,62011312	-0,6893967	-0,373614	0,45229577	0,34457747	Unpaired t-te	0,391439364	permutation FDR (250 perm	1
732	Cpsf7~Q8BT	Cpsf7	ko vs wt	ko	wt	25,7391847	0,69244648	26,2983023	0,22378913	0,67871716	-0,5591176	-0,3910015	0,26548122	0,5759662	Unpaired t-te	0,378519411	permutation FDR (250 perm	1
733	Cpt1a~P9774	Cpt1a	ko vs wt	ko	wt	30,0395022	0,39770477	29,7922328	0,15120929	1,18695846	0,24726945	0,19729648	0,38236794	0,41751853	Unpaired t-te	0,650635939	permutation FDR (250 perm	1
734	Cpt1b~Q924	Cpt1b	ko vs wt	ko	wt	29,3087738	0,62853012	29,4383431	0,17439333	0,91410426	-0,1295694	-0,0936694	0,75380253	0,12274241	Unpaired t-te	0,839413319	permutation FDR (250 perm	1
735	Cpt2~P52825	Cpt2	ko vs wt	ko	wt	30,3862293	0,21933861	30,6268942	0,26914958	0,8463552	-0,2406648	-0,1958857	0,35656605	0,44786001	Unpaired t-te	0,655983683	permutation FDR (250 perm	1
736	Cpxm1~Q9Z1	Cpxm1	ko vs wt	ko	wt	24,3801628	1,62369109	23,5961943	0,09030981	1,72186083	0,78396854	0,40418854	0,46479056	0,3327427	Unpaired t-te	0,372722148	permutation FDR (250 perm	1
737	Cpz~Q8R4V4	Cpz	ko vs wt	ko	wt	24,7199478	1,27314848	23,774522	0,95735183	1,92575712	0,94542576	0,47288275	0,38813229	0,41102023	Unpaired t-te	0,28330742	permutation FDR (250 perm	1
738	Cp~G3X9T8	Cp	ko vs wt	ko	wt	29,6068189	0,98763954	28,6646369	0,5570212	1,92143214	0,94218203	0,55650885	0,23342961	0,63184406	Unpaired t-te	0,216551828	permutation FDR (250 perm	1
739	Cr1l~Q64735	Cr1l	ko vs wt	ko	wt	28,151522	0,27104573	27,8338657	0,5035416	1,24630422	0,31765627	0,22870593	0,47822232	0,32037016	Unpaired t-te	0,607872189	permutation FDR (250 perm	1
740	Cracr2b~Q80	Cracr2b	ko vs wt	ko	wt	24,3090374	0,45363931	24,271217	0,04230485	1,02656178	0,03782045	0,02993044	0,89479886	0,04827458	Unpaired t-te	0,948698178	permutation FDR (250 perm	1
741	Crat~P47934	Crat	ko vs wt	ko	wt	29,8939982	0,60377267	30,4524743	0,33430075	0,67901902	-0,5584761	-0,3929666	0,2438141	0,61294119	Unpaired t-te	0,369538151	permutation FDR (250 perm	1
742	Crebbp~P454	Crebbp	ko vs wt	ko	wt	24,4406881	0,46113397	25,2412196	0,59662368	0,57413762	-0,8005315	-0,5340935	0,19303051	0,71437405	Unpaired t-te	0,243967659	permutation FDR (250 perm	1
743	Creg1~O886	Creg1	ko vs wt	ko	wt	24,956131	0,89187363	24,1895047	1,14897871	1,70128673	0,76662631	0,39076012	0,47536184	0,32297569	Unpaired t-te	0,381614691	permutation FDR (250 perm	1
744	Crip1~P6325	Crip1	ko vs wt	ko	wt	32,3435192	0,27807992	32,3708583	0,88779884	0,98122837	-0,0273391	-0,0165896	0,96977749	0,0133279	Unpaired t-te	0,972097408	permutation FDR (250 perm	1
745	Crip2~Q9DCT	Crip2	ko vs wt	ko	wt	31,1415643	0,3372911	31,7321071	0,64001011	0,66409303	-0,5905427	-0,3956273	0,32379848	0,48972519	Unpaired t-te	0,385945442	permutation FDR (250 perm	1
746	Crispld1~Q8	Crispld1	ko vs wt	ko	wt	24,7657505	0,48690057	26,2646885	0,51014273	0,35381375	-1,498938	-1,0285538	0,02898614	1,53780955	Unpaired t-te	0,043512309	permutation FDR (250 perm	1
747	Crispld2~Q8	Crispld2	ko vs wt	ko	wt	24,635068	1,33976108	24,6436058	0,89412146	0,99409953	-0,0085378	-0,004271	0,99351175	0,00282699	Unpaired t-te	0,992418641	permutation FDR (250 perm	1
748	Crkl~P47941	Crkl	ko vs wt	ko	wt	25,5279274	1,4922447	26,4270203	0,1680514	0,53622379	-0,8990929	-0,4808744	0,37474968	0,42625873	Unpaired t-te	0,292369902	permutation FDR (250 perm	1
749	Crk~Q64010	Crk	ko vs wt	ko	wt	25,0663158	0,71018204	25,7979669	0,09279466	0,60221431	-0,7316511	-0,5169799	0,17182449	0,76491494	Unpaired t-te	0,261558442	permutation FDR (250 perm	1
750	Crlf1~Q9JM5	Crlf1	ko vs wt	ko	wt	27,4904211	1,46267723	25,6505588	0,8906979	3,57975859	1,8398623	0,89597432	0,14160609	0,84891806	Unpaired t-te	0,06726935	permutation FDR (250 perm	1
751	Crlf2~A0A0G	Crlf2	ko vs wt	ko	wt	24,143591	1,12860284	24,6201559	0,74649253	0,71868683	-0,4765648	-0,2592035	0,59440178	0,2259199	Unpaired t-te	0,545896594	permutation FDR (250 perm	1
752	Crocc~Q8CJ4	Crocc	ko vs wt	ko	wt	25,1887176	1,23863145	26,1961599	0,43333456	0,4974273	-1,0074424	-0,5666139	0,26503304	0,57669998	Unpaired t-te	0,215211778	permutation FDR (250 perm	1
753	Crp~P14847	Crp	ko vs wt	ko	wt	24,7689602	0,43616869	25,3373889	0,70780361	0,67435085	-0,5684287	-0,3643133	0,38475598	0,41481462	Unpaired t-te	0,417700326	permutation FDR (250 perm	1
754	Crtap~Q9CYD	Crtap	ko vs wt	ko	wt	28,8937712	0,525713	28,3306134	0,27207443	1,47749969	0,56315782	0,41428271	0,18096126	0,74241438	Unpaired t-te	0,345847411	permutation FDR (250 perm	1
755	Cryab~P2392	Cryab	ko vs wt	ko	wt	30,1506319	0,38424281	30,1446754	0,39935096	1,00413726	0,0059565	0,00438267	0,98752923	0,00545004	Unpaired t-te	0,992278053	permutation FDR (250 perm	1
756	Cryl1~Q99KP	Cryl1	ko vs wt	ko	wt	25,6373599	1,55749991	24,4210419	1,89660748	2,32352951	1,21631797	0,46518971	0,49631698	0,30424087	Unpaired t-te	0,300778128	permutation FDR (250 perm	1
757	Cryzl1~D3Z6	Cryzl1	ko vs wt	ko	wt	25,3213609	1,26201436	24,9212261	0,80400552	1,3196312	0,4001348	0,20795098	0,68311563	0,16550578	Unpaired t-te	0,628947059	permutation FDR (250 perm	1
758	Cryzl2~Q3UN	Cryzl2	ko vs wt	ko	wt	25,2177168	2,08402299	24,1912752	1,46608741	2,03699381	1,0264416	0,39658298	0,54666003	0,26228268	Unpaired t-te	0,364598584	permutation FDR (250 perm	1
759	Cryz~P47199	Cryz	ko vs wt	ko	wt	26,0715822	0,21492973	24,5422162	0,64812488	2,88658953	1,52936597	1,03700174	0,07022113	1,15353219	Unpaired t-te	0,059365147	permutation FDR (250 perm	1
760	Csad~Q9DBE	Csad	ko vs wt	ko	wt	25,8605899	0,62060272	25,1503506	0,73759869	1,63607545	0,71023928	0,43498817	0,32799062	0,48413858	Unpaired t-te	0,330360137	permutation FDR (250 perm	1
761	Csde1~Q91W	Csde1	ko vs wt	ko	wt	28,7862519	0,537055	28,83727	0,29250465	0,96525493	-0,0510181	-0,0371656	0,89664832	0,04737786	Unpaired t-te	0,93487656	permutation FDR (250 perm	1
762	Cse1l~Q9ER	Cse1l	ko vs wt	ko	wt	31,5952269	2,25087393	31,206793	3,11560541	1,30897169	0,3884339	0,1091783	0,88795806	0,05160755	Unpaired t-te	0,811680355	permutation FDR (250 perm	1
763	Csk~P41241	Csk	ko vs wt	ko	wt	28,2305843	0,88410834	27,4640724	0,08143957	1,7011518	0,76651188	0,50639022	0,23028424	0,63773579	Unpaired t-te	0,270814762	permutation FDR (250 perm	1
764	Csnk1a1~E9F	Csnk1a1	ko vs wt	ko	wt	28,0133204	0,66262394	28,4790593	0,09433875	0,72410016	-0,4657388	-0,3354649	0,31206115	0,50576029	Unpaired t-te	0,450623683	permutation FDR (250 perm	1
765	Csnk1d~Q9D	Csnk1d	ko vs wt	ko	wt	26,4533567	0,41189476	26,2146961	0,1613372	1,17989675	0,23866061	0,18885022	0,41437288	0,38260868	Unpaired t-te	0,665446833	permutation FDR (250 perm	1

766	Csnk1e~Q9JN	Csnk1e	ko vs wt	ko	wt	24,3099964	0,48575779	24,214415	0,8329921	1,06849592	0,09558141	0,05784491	0,89307465	0,04911224	Unpaired t-te	0,902346867	permutation FDR (250 perm	1
767	Csnk1g1~Q8I	Csnk1g1	ko vs wt	ko	wt	24,1081263	0,7938403	24,1637014	0,28760161	0,96221078	-0,0555751	-0,0370151	0,91700792	0,03762691	Unpaired t-te	0,935830149	permutation FDR (250 perm	1
768	Csnk2a1~Q60	Csnk2a1	ko vs wt	ko	wt	29,0262659	0,19777926	28,4054578	0,86219537	1,53773627	0,6208081	0,38315208	0,41631585	0,38057706	Unpaired t-te	0,411431373	permutation FDR (250 perm	1
769	Csnk2a2~O54	Csnk2a2	ko vs wt	ko	wt	27,7247044	0,35135323	27,0596985	0,84097541	1,58557479	0,66500593	0,40840332	0,38225139	0,41765093	Unpaired t-te	0,377267738	permutation FDR (250 perm	1
770	Csnk2b~P678	Csnk2b	ko vs wt	ko	wt	27,9004254	0,2612077	27,2795726	0,27193496	1,53778387	0,62085275	0,49892875	0,06150075	1,2111196	Unpaired t-te	0,266541143	permutation FDR (250 perm	1
771	Cspg4~Q8VH	Cspg4	ko vs wt	ko	wt	32,3167129	0,34405663	32,3998685	0,06982705	0,9439906	-0,0831556	-0,0690268	0,70904341	0,14932718	Unpaired t-te	0,884060754	permutation FDR (250 perm	1
772	Csrp1~P9731	Csrp1	ko vs wt	ko	wt	31,9623027	0,85023829	32,8382121	0,70861988	0,54491027	-0,8759094	-0,5148001	0,26968117	0,56914938	Unpaired t-te	0,249132433	permutation FDR (250 perm	1
773	Csrp2~P9731	Csrp2	ko vs wt	ko	wt	32,7714444	0,63721093	33,2172117	0,81828145	0,73419373	-0,4457673	-0,2644458	0,55519619	0,25555353	Unpaired t-te	0,546088754	permutation FDR (250 perm	1
774	Cst3~P21460	Cst3	ko vs wt	ko	wt	28,9928457	0,28553274	28,364586	0,74398356	1,54569935	0,62825973	0,4049891	0,35547647	0,44918915	Unpaired t-te	0,38212871	permutation FDR (250 perm	1
775	Cstb~Q6242(	Cstb	ko vs wt	ko	wt	29,1508423	0,10563724	28,7184725	0,05828805	1,34944843	0,43236985	0,40272509	0,00225057	2,64770748	Unpaired t-te	0,358555179	permutation FDR (250 perm	1
776	Cstf1~Q99LC	Cstf1	ko vs wt	ko	wt	26,5221576	0,44862808	26,2883535	0,14842562	1,17593153	0,23380406	0,18273492	0,45092315	0,34589747	Unpaired t-te	0,677976266	permutation FDR (250 perm	1
777	Cstf2~Q8BIQ	Cstf2	ko vs wt	ko	wt	25,2201808	0,61015024	24,8979256	0,70082716	1,25028342	0,32225516	0,20040644	0,62502622	0,20410177	Unpaired t-te	0,646369635	permutation FDR (250 perm	1
778	Cstf3~Q99LI	Cstf3	ko vs wt	ko	wt	26,4347872	0,38747364	27,1369519	0,34343288	0,61464926	-0,7021647	-0,5278722	0,09135219	1,03928104	Unpaired t-te	0,240013274	permutation FDR (250 perm	1
779	Cs~Q9CZU6	Cs	ko vs wt	ko	wt	32,4024853	0,33822713	32,0497617	0,32701385	1,27696901	0,35272352	0,27077226	0,30331985	0,51809917	Unpaired t-te	0,531137255	permutation FDR (250 perm	1
780	Ctbp1~O887	Ctbp1	ko vs wt	ko	wt	27,7534092	0,59263903	26,8184538	0,84685298	1,91183156	0,93495542	0,55333398	0,26087721	0,58356386	Unpaired t-te	0,232374861	permutation FDR (250 perm	1
781	Ctbp2~P5654	Ctbp2	ko vs wt	ko	wt	26,7734563	0,28467096	26,5200948	0,11586289	1,19198119	0,25336147	0,21405233	0,23540374	0,62818665	Unpaired t-te	0,621358869	permutation FDR (250 perm	1
782	Ctcf~Q61164	Ctcf	ko vs wt	ko	wt	26,9094285	0,15310473	26,6730316	0,48260326	1,17804682	0,23639688	0,17478322	0,56424041	0,24853581	Unpaired t-te	0,704383844	permutation FDR (250 perm	1
783	Cthrc1~Q9D1	Cthrc1	ko vs wt	ko	wt	27,3309335	0,92918315	26,0701725	0,56724913	2,3962211	1,26076103	0,75502111	0,11895705	0,92460981	Unpaired t-te	0,112345479	permutation FDR (250 perm	1
784	Ctif~Q6PEE2	Ctif	ko vs wt	ko	wt	24,5048253	0,71107578	24,7948355	0,38425094	0,81789629	-0,2900102	-0,1943367	0,5823409	0,23482271	Unpaired t-te	0,653237679	permutation FDR (250 perm	1
785	Ctnna1~P262	Ctnna1	ko vs wt	ko	wt	30,5552797	0,20533631	30,5932474	0,05757596	0,9740261	-0,0379677	-0,0337386	0,77833659	0,10883255	Unpaired t-te	0,942329624	permutation FDR (250 perm	1
786	Ctnna3~Q65(	Ctnna3	ko vs wt	ko	wt	25,5076916	0,96749712	26,4563797	0,28722093	0,51810335	-0,9486882	-0,5950273	0,19043798	0,72024643	Unpaired t-te	0,199308699	permutation FDR (250 perm	1
787	Ctnnal1~O88	Ctnnal1	ko vs wt	ko	wt	27,5617354	0,61189014	28,0561261	0,36437156	0,70986141	-0,4943907	-0,3439841	0,31004897	0,5085697	Unpaired t-te	0,427537415	permutation FDR (250 perm	1
788	Ctnnb1~Q02	Ctnnb1	ko vs wt	ko	wt	28,4709381	0,8297341	28,6564213	0,39884168	0,8793545	-0,1854832	-0,1192129	0,7532715	0,12304847	Unpaired t-te	0,789418994	permutation FDR (250 perm	1
789	Ctnnbl1~Q9C	Ctnnbl1	ko vs wt	ko	wt	25,7027503	1,63345772	25,3400032	1,23807527	1,28587203	0,36274708	0,15862769	0,78949283	0,10265181	Unpaired t-te	0,716135922	permutation FDR (250 perm	1
790	Ctnnd1~P309	Ctnnd1	ko vs wt	ko	wt	30,1171047	0,25275287	30,237934	0,30815434	0,91965882	-0,1208294	-0,0957255	0,67074857	0,17344024	Unpaired t-te	0,835086753	permutation FDR (250 perm	1
791	Ctps1~P7069	Ctps1	ko vs wt	ko	wt	28,0556632	0,3979279	27,5958396	0,25911896	1,37537364	0,45982361	0,35538908	0,17843821	0,74851215	Unpaired t-te	0,412487422	permutation FDR (250 perm	1
792	Ctps2~P7030	Ctps2	ko vs wt	ko	wt	27,2452975	0,71036618	26,7989748	0,29523979	1,3625627	0,44632262	0,30565721	0,38323183	0,41653842	Unpaired t-te	0,481525307	permutation FDR (250 perm	1
793	Ctr9~Q6201ε	Ctr9	ko vs wt	ko	wt	26,7352041	0,53113672	26,4749093	0,09612729	1,19772341	0,26029479	0,19807913	0,46311423	0,33431187	Unpaired t-te	0,654864471	permutation FDR (250 perm	1
794	Ctsa~G3X8T3	Ctsa	ko vs wt	ko	wt	28,2404095	1,06267706	28,387666	0,22676672	0,90296592	-0,1472566	-0,0901123	0,82966228	0,08109866	Unpaired t-te	0,845762898	permutation FDR (250 perm	1
795	Ctsb~P10605	Ctsb	ko vs wt	ko	wt	33,5148218	0,13058214	32,5851653	0,58675363	1,90482232	0,92965643	0,65390864	0,15034737	0,82290417	Unpaired t-te	0,189637709	permutation FDR (250 perm	1
796	Ctsd~P18242	Ctsd	ko vs wt	ko	wt	31,9282638	0,54309178	31,864169	0,37158723	1,04542882	0,06409484	0,04548668	0,88163349	0,05471192	Unpaired t-te	0,921099947	permutation FDR (250 perm	1
797	Ctsf~Q9R013	Ctsf	ko vs wt	ko	wt	25,6533837	1,36230155	26,5489281	0,22029091	0,53754431	-0,8955444	-0,4970272	0,34010753	0,46838375	Unpaired t-te	0,276469751	permutation FDR (250 perm	1
798	Ctsh~P49935	Ctsh	ko vs wt	ko	wt	27,3905073	0,40555166	25,4655475	0,91968264	3,79726268	1,9249598	1,13823359	0,0835865	1,07786385	Unpaired t-te	0,04020915	permutation FDR (250 perm	1
799	Ctsk~P55097	Ctsk	ko vs wt	ko	wt	24,8875181	0,55852465	23,675457	0,57430222	2,31668371	1,21206109	0,79816968	0,0764449	1,11665147	Unpaired t-te	0,099933579	permutation FDR (250 perm	1
800	Ctsl~P06797	Ctsl	ko vs wt	ko	wt	27,1651722	1,2106835	27,6830164	0,05434522	0,69841466	-0,5178442	-0,3046064	0,51279586	0,29005549	Unpaired t-te	0,493783598	permutation FDR (250 perm	1
801	Ctss~F6WR0	Ctss	ko vs wt	ko	wt	26,8692742	0,94547532	25,8296123	1,18169359	2,05660092	1,04026187	0,52062934	0,36165824	0,44170163	Unpaired t-te	0,253247453	permutation FDR (250 perm	1
802	Ctsz~Q9WUl	Ctsz	ko vs wt	ko	wt	26,465591	0,35446014	25,1919777	2,18621402	2,41766312	1,27361323	0,4976269	0,49735486	0,30333364	Unpaired t-te	0,298666199	permutation FDR (250 perm	1
803	Cttn~Q60598	Cttn	ko vs wt	ko	wt	29,9711814	0,38767203	30,0927766	0,5075731	0,91917075	-0,1215952	-0,0854511	0,78993058	0,10241107	Unpaired t-te	0,854225144	permutation FDR (250 perm	1
804	Cul1~Q9WTX	Cul1	ko vs wt	ko	wt	28,2521096	0,39341168	28,4405673	0,30831206	0,87754335	-0,1884577	-0,1433322	0,57618129	0,23944085	Unpaired t-te	0,742780791	permutation FDR (250 perm	1
805	Cul2~Q9D4H	Cul2	ko vs wt	ko	wt	28,3839927	0,61904851	27,6796803	0,13573875	1,62936707	0,70431237	0,51406982	0,14171942	0,84857064	Unpaired t-te	0,260514079	permutation FDR (250 perm	1
806	Cul3~Q9JLV5	Cul3	ko vs wt	ko	wt	27,6808497	0,61816678	28,1290268	0,15478268	0,73296839	-0,4481771	-0,3263496	0,30397165	0,51716692	Unpaired t-te	0,458527787	permutation FDR (250 perm	1
807	Cul4a~Q3TCI	Cul4a	ko vs wt	ko	wt	27,7160983	0,17017513	27,6337937	0,21186696	1,05870789	0,08230458	0,06979955	0,67201854	0,17261875	Unpaired t-te	0,882787286	permutation FDR (250 perm	1
808	Cul5~Q9D5V	Cul5	ko vs wt	ko	wt	27,9693408	0,22151641	27,3509279	0,53315309	1,53518536	0,61841286	0,44232416	0,23708389	0,62509795	Unpaired t-te	0,341115739	permutation FDR (250 perm	1
809	Cux1~P7040	Cux1	ko vs wt	ko	wt	24,1696148	0,35241952	23,9494447	0,54959677	1,16487092	0,2201701	0,15303764	0,64930239	0,187553	Unpaired t-te	0,734213273	permutation FDR (250 perm	1
810	Cwc22~Q8C5	Cwc22	ko vs wt	ko	wt	23,7623915	0,6404122	23,9298138	0,49471014	0,89043225	-0,1674223	-0,1109493	0,75582139	0,12158082	Unpaired t-te	0,804308377	permutation FDR (250 perm	1
811	Cxcl12~H7BX	Cxcl12	ko vs wt	ko	wt	30,2040389	1,08735217	29,148899	0,40271106	2,0779197	1,0551399	0,62458437	0,19888092	0,70140689	Unpaired t-te	0,177916404	permutation FDR (250 perm	1
812	Cyb5a~P563	Cyb5a	ko vs wt	ko	wt	27,3648795	0,95908271	26,8054914	0,75797257	1,47364406	0,5593881	0,31592558	0,50135655	0,29985331	Unpaired t-te	0,463364155	permutation FDR (250 perm	1
813	Cyb5b~Q9CQ	Cyb5b	ko vs wt	ko	wt	25,5037247	1,14033185	23,9610201	0,44852478	2,91340152	1,54270455	0,89133483	0,09886205	1,00497041	Unpaired t-te	0,072046559	permutation FDR (250 perm	1
814	Cyb5r1~Q9DI	Cyb5r1	ko vs wt	ko	wt	26,1103711	0,49438194	26,3448737	0,61119134	0,84997802	-0,2345026	-0,1544887	0,67623387	0,16990308	Unpaired t-te	0,728102244	permutation FDR (250 perm	1
815	Cyb5r3~Q9D(	Cyb5r3	ko vs wt	ko	wt	31,8568187	0,36897616	31,4648362	0,70315655	1,31219531	0,39198246	0,25438191	0,52558079	0,27936052	Unpaired t-te	0,569315647	permutation FDR (250 perm	1
816	Cybb~Q6109	Cybb	ko vs wt	ko	wt	26,7051296	0,99667769	24,8136634	0,24590446	3,71012085	1,89146618	1,18133244	0,04116005	1,38552407	Unpaired t-te	0,027465426	permutation FDR (250 perm	1
817	Cyc1~Q9D0M	Cyc1	ko vs wt	ko	wt	29,1744729	0,24784309	28,9301213	0,53213288	1,18456034	0,24435168	0,1742178	0,59297779	0,22696157	Unpaired t-te	0,701539696	permutation FDR (250 perm	1
818	Cycs~P62897	Cycs	ko vs wt	ko	wt	29,2672886	0,27001607	29,0160914	0,31244896	1,19019443	0,25119727	0,19773116	0,40737088	0,39001001	Unpaired t-te	0,651527559	permutation FDR (250 perm	1
819	Cyfip1~Q7TM	Cyfip1	ko vs wt	ko	wt	30,6670006	0,30431851	30,3816761	0,3086879	1,21868441	0,28532458	0,22287438	0,36304042	0,44004502	Unpaired t-te	0,606114929	permutation FDR (250 perm	1
820	Cyfip2~Q5SC	Cyfip2	ko vs wt	ko	wt	24,801554	0,52059685	24,4144091	0,81995725	1,30780262	0,38714482	0,23419694	0,59413177	0,22611723	Unpaired t-te	0,596010711	permutation FDR (250 perm	1
821	Cygb~Q9CX8	Cygb	ko vs wt	ko	wt	27,0903969	1,09859275	27,6783055	0,60566585	0,6653067	-0,5879085	-0,3330319	0,47810731	0,32047461	Unpaired t-te	0,441033045	permutation FDR (250 perm	1
822	Cyp20a1~Q8I	Cyp20a1	ko vs wt	ko	wt	26,9871607	0,81469852	23,6497999	0,17975711	10,1075457	3,33736082	2,24399916	0,00410409	2,3867826	Unpaired t-te	0,002873239	permutation FDR (250 perm	1
823	Cyp2d22~Q9	Cyp2d22	ko vs wt	ko	wt	24,2062309	0,48397424	24,9615335	0,0963741	0,59242214	-0,7553025	-0,5865914	0,07009344	1,15432262	Unpaired t-te	0,208757991	permutation FDR (250 perm	1
824	Cyp4f16~Q9	Cyp4f16	ko vs wt	ko	wt	24,3613905	0,46396331	24,7075248	0,40478872	0,78668922	-0,3461343	-0,2486558	0,42050568	0,37622813	Unpaired t-te	0,562813651	permutation FDR (250 perm	1
825	Cyrib~Q921M	Cyrib	ko vs wt	ko	wt	24,9782528	1,44515805	23,9547771	0,79904561	2,03281042	1,02347567	0,50978298	0,35772098	0,44645559	Unpaired t-te	0,253061908	permutation FDR (250 perm	1
826	D6Wsu163e	D6Wsu163e	ko vs wt	ko	wt	23,7862837	0,55724989	24,5772337	0,69338817	0,57796338	-0,79095	-0,4985716	0,25515281	0,59319965	Unpaired t-te	0,270826227	permutation FDR (250 perm	1
827	Daam1~Q8B	Daam1	ko vs wt	ko	wt	27,5825104	1,29122112	26,911682	0,21159588	1,59198684	0,67082841	0,38107602	0,43821927	0,35830853	Unpaired t-te	0,395370297	permutation FDR (250 perm	1
828	Dab2ip~Q3U	Dab2ip	ko vs wt	ko	wt	24,5846764	1,23243688	26,8709557	0,26064693	0,20500353	-2,2862793	-1,3177177	0,04479572	1,34876345	Unpaired t-te	0,017761062	permutation FDR (250 perm	1
829	Dab2~P9807	Dab2	ko vs wt	ko	wt	27,4601542	0,34137127	24,9221979	2,05018201	5,80765727	2,53795632	1,03041879	0,22043469	0,65672005	Unpaired t-te	0,065271496	permutation FDR (250 perm	1

830	Dag1~Q6216	Dag1	ko vs wt	ko	wt	29,571039	0,49111012	30,2439262	0,46042771	0,62725011	-0,6728873	-0,469981	0,18664996	0,7289721	Unpaired t-te	0,289323001	permutation FDR (250 perm	1
831	Daglb~Q91W	Daglb	ko vs wt	ko	wt	25,0577612	0,50264096	24,4039358	0,28704653	1,57333444	0,65382537	0,48283505	0,12538759	0,90174544	Unpaired t-te	0,274719829	permutation FDR (250 perm	1
832	Dap3~Q9ER8	Dap3	ko vs wt	ko	wt	26,3420547	0,34325852	26,2744737	0,32255859	1,0479581	0,06758104	0,05189939	0,83288897	0,07941289	Unpaired t-te	0,910670244	permutation FDR (250 perm	1
833	Dapk3~O547	Dapk3	ko vs wt	ko	wt	29,5325322	0,42381718	29,6761682	0,39936183	0,90523488	-0,1436359	-0,1045647	0,71832743	0,14367755	Unpaired t-te	0,815888182	permutation FDR (250 perm	1
834	Dars1~Q922I	Dars1	ko vs wt	ko	wt	31,3741758	0,20496539	30,9808078	0,13140921	1,31345604	0,39336792	0,3419227	0,04748001	1,32348915	Unpaired t-te	0,429761397	permutation FDR (250 perm	1
835	Daxx~O3561	Daxx	ko vs wt	ko	wt	24,5254472	0,23608811	23,9755603	0,47946245	1,46397101	0,54988698	0,40272791	0,24081184	0,61832216	Unpaired t-te	0,38022861	permutation FDR (250 perm	1
836	Dazap1~Q9JI	Dazap1	ko vs wt	ko	wt	29,0152167	0,38867921	28,1596788	0,62901866	1,80943322	0,85553786	0,57104911	0,18389477	0,73543063	Unpaired t-te	0,223010109	permutation FDR (250 perm	1
837	Dbi~P31786	Dbi	ko vs wt	ko	wt	26,1795986	1,1218895	26,9107957	0,6593209	0,60240386	-0,7311971	-0,4066592	0,40215337	0,39560828	Unpaired t-te	0,353704584	permutation FDR (250 perm	1
838	Dbn1~Q9QX	Dbn1	ko vs wt	ko	wt	27,6649049	0,53912962	28,1016278	0,06035864	0,73881089	-0,436723	-0,3323162	0,25562189	0,59240196	Unpaired t-te	0,455339064	permutation FDR (250 perm	1
839	Dbnl~Q6241	Dbnl	ko vs wt	ko	wt	28,6937378	0,45198276	27,4334306	0,66880884	2,39546754	1,26030726	0,81830815	0,09623895	1,01664913	Unpaired t-te	0,101899678	permutation FDR (250 perm	1
840	Dbt~P53395	Dbt	ko vs wt	ko	wt	28,3838854	0,52549361	28,4576585	0,55380059	0,95014983	-0,0737731	-0,0493342	0,88860449	0,0512915	Unpaired t-te	0,915072805	permutation FDR (250 perm	1
841	Dcaf1~Q80TI	Dcaf1	ko vs wt	ko	wt	23,73529	0,64142319	24,0484666	0,89885343	0,80486757	-0,3131767	-0,1804427	0,69631707	0,15719296	Unpaired t-te	0,684081844	permutation FDR (250 perm	1
842	Dcaf7~P6196	Dcaf7	ko vs wt	ko	wt	26,8064219	0,47291992	26,0876205	0,06438213	1,64581415	0,71880143	0,56296676	0,07630715	1,11743478	Unpaired t-te	0,227009339	permutation FDR (250 perm	1
843	Dcaf8~Q8N7	Dcaf8	ko vs wt	ko	wt	25,4822635	1,13292295	26,9709146	0,42313507	0,35634556	-1,4886511	-0,8658587	0,10529943	0,977574	Unpaired t-te	0,079730959	permutation FDR (250 perm	1
844	Dcakd~Q8BH	Dcakd	ko vs wt	ko	wt	25,2772374	0,4681054	24,4774284	0,2420945	1,74087071	0,79980906	0,60595486	0,05710032	1,24336149	Unpaired t-te	0,184791243	permutation FDR (250 perm	1
845	Dclk1~Q9JLM	Dclk1	ko vs wt	ko	wt	27,0796876	0,38489633	24,6709097	1,9696937	5,31024301	2,40877788	0,999327	0,22368651	0,65036021	Unpaired t-te	0,070956256	permutation FDR (250 perm	1
846	Dcn~P28654	Dcn	ko vs wt	ko	wt	35,8052481	0,40751945	36,2258973	0,16471852	0,74708848	-0,4206492	-0,3331789	0,17991599	0,74493025	Unpaired t-te	0,44406274	permutation FDR (250 perm	1
847	Dcp1a~Q91Y	Dcp1a	ko vs wt	ko	wt	24,7542767	0,3796815	25,0139131	0,46722199	0,83529843	-0,2596364	-0,1859213	0,55128534	0,25862356	Unpaired t-te	0,674340389	permutation FDR (250 perm	1
848	Dcp1b~Q3U5	Dcp1b	ko vs wt	ko	wt	24,299908	0,56486579	24,3231795	0,19810113	0,9839988	-0,0232715	-0,0171754	0,95086651	0,02188045	Unpaired t-te	0,970325118	permutation FDR (250 perm	1
849	Dcps~Q9DAR	Dcps	ko vs wt	ko	wt	25,0649499	0,76548496	24,4431159	1,39212131	1,53883019	0,62183404	0,29909699	0,60730057	0,21659631	Unpaired t-te	0,50472874	permutation FDR (250 perm	1
850	Dctn1~E9Q5	Dctn1	ko vs wt	ko	wt	30,8870692	0,20251768	30,9549278	0,11538217	0,95405305	-0,0678586	-0,0593909	0,65460099	0,18402334	Unpaired t-te	0,896029103	permutation FDR (250 perm	1
851	Dctn2~Q99K	Dctn2	ko vs wt	ko	wt	28,44948	0,629222	28,3436435	0,25702877	1,0761182	0,10583655	0,07526377	0,80648338	0,09340458	Unpaired t-te	0,871859247	permutation FDR (250 perm	1
852	Dctn3~Q9Z0	Dctn3	ko vs wt	ko	wt	25,6817861	1,28118949	24,4445513	0,87228609	2,35746251	1,23723483	0,63024065	0,2552426	0,59304684	Unpaired t-te	0,170252303	permutation FDR (250 perm	1
853	Dctn4~Q8CB	Dctn4	ko vs wt	ko	wt	27,8628837	0,22417899	27,5034924	0,413525	1,28288455	0,35939135	0,2723134	0,3484027	0,45791849	Unpaired t-te	0,542674794	permutation FDR (250 perm	1
854	Dctn6~D3Z6	Dctn6	ko vs wt	ko	wt	26,0748612	1,66450483	26,3145071	0,23807776	0,84695319	-0,2396459	-0,1213006	0,8209654	0,08567514	Unpaired t-te	0,790614182	permutation FDR (250 perm	1
855	Dcun1d1~Q9	Dcun1d1	ko vs wt	ko	wt	25,171993	1,11063607	24,3703486	0,6854219	1,74308683	0,80164444	0,44442318	0,36462877	0,43814907	Unpaired t-te	0,312883008	permutation FDR (250 perm	1
856	Ddah2~Q99L	Ddah2	ko vs wt	ko	wt	25,3860396	1,49399548	25,7584405	1,7574534	0,77249583	-0,3724009	-0,1482062	0,81825564	0,08711099	Unpaired t-te	0,738048118	permutation FDR (250 perm	1
857	Ddb1~Q3U1	Ddb1	ko vs wt	ko	wt	29,288107	0,30022113	29,1660743	0,52663557	1,08826709	0,12203268	0,0865019	0,78658481	0,10425444	Unpaired t-te	0,854705012	permutation FDR (250 perm	1
858	Ddhd1~Q80Y	Ddhd1	ko vs wt	ko	wt	23,9861955	0,5805384	25,0582376	0,9547007	0,47564526	-1,0720421	-0,6113019	0,25045395	0,60127211	Unpaired t-te	0,197589823	permutation FDR (250 perm	1
859	Ddi2~A2ADY	Ddi2	ko vs wt	ko	wt	26,4472758	0,3458294	27,2175642	0,68807842	0,58630026	-0,7702884	-0,5048026	0,24953615	0,60286654	Unpaired t-te	0,277906927	permutation FDR (250 perm	1
860	Ddost~O547	Ddost	ko vs wt	ko	wt	30,4638401	0,88017586	30,0971539	0,26943558	1,28938775	0,36668619	0,23768962	0,5382282	0,26903355	Unpaired t-te	0,584383742	permutation FDR (250 perm	1
861	Ddx17~Q3U7	Ddx17	ko vs wt	ko	wt	31,3641923	0,1775299	30,8226086	0,21723497	1,45556944	0,54158366	0,45716189	0,04715735	1,32645062	Unpaired t-te	0,30918074	permutation FDR (250 perm	1
862	Ddx17~Q501	Ddx17	ko vs wt	ko	wt	24,0730876	0,73911043	24,3884454	0,18904728	0,80365167	-0,3153578	-0,2179129	0,52398513	0,28068103	Unpaired t-te	0,618621869	permutation FDR (250 perm	1
863	Ddx19a~Q61	Ddx19a	ko vs wt	ko	wt	28,6779522	0,21405845	28,9487028	0,35028251	0,82888817	-0,2707506	-0,2120527	0,40023665	0,39768315	Unpaired t-te	0,634454038	permutation FDR (250 perm	1
864	Ddx1~Q91VR	Ddx1	ko vs wt	ko	wt	31,5502219	0,19079477	31,5891512	0,15937079	0,97337708	-0,0389293	-0,0336297	0,8151928	0,08873967	Unpaired t-te	0,941898197	permutation FDR (250 perm	1
865	Ddx21~Q9JIK	Ddx21	ko vs wt	ko	wt	27,8339521	0,75025435	27,4564743	0,224766	1,29906871	0,37747774	0,25829927	0,46195689	0,33539855	Unpaired t-te	0,553862383	permutation FDR (250 perm	1
866	Ddx23~D3Z0	Ddx23	ko vs wt	ko	wt	27,9180144	0,34595255	27,7330749	0,41310033	1,13676936	0,18493957	0,13660121	0,63050841	0,20030911	Unpaired t-te	0,759091983	permutation FDR (250 perm	1
867	Ddx24~Q9ES	Ddx24	ko vs wt	ko	wt	24,4457561	0,83410241	24,1571445	0,1521837	1,22146419	0,28861157	0,19325211	0,59628775	0,22454411	Unpaired t-te	0,663553359	permutation FDR (250 perm	1
868	Ddx39b~Q9Z	Ddx39b	ko vs wt	ko	wt	30,7601546	0,26631726	30,4445708	0,211754	1,24451514	0,31558378	0,25982111	0,20318023	0,69211855	Unpaired t-te	0,54546472	permutation FDR (250 perm	1
869	Ddx3x~Q621	Ddx3x	ko vs wt	ko	wt	32,5603944	0,15520186	32,6892089	0,09317652	0,91458265	-0,1288145	-0,1159216	0,29959366	0,52346738	Unpaired t-te	0,794520089	permutation FDR (250 perm	1
870	Ddx3y~Q620	Ddx3y	ko vs wt	ko	wt	29,7431321	0,02857944	29,1266411	0,43925861	1,53314163	0,61649098	0,47023024	0,18525561	0,73222863	Unpaired t-te	0,32571468	permutation FDR (250 perm	1
871	Ddx42~Q810	Ddx42	ko vs wt	ko	wt	26,8965891	0,22046814	26,7629599	0,14243641	1,09704996	0,13362923	0,11496818	0,44785926	0,34885844	Unpaired t-te	0,796643794	permutation FDR (250 perm	1
872	Ddx46~Q569	Ddx46	ko vs wt	ko	wt	29,1379997	0,32451068	29,1913865	0,32547658	0,96367143	-0,0533868	-0,0411692	0,86543989	0,06276309	Unpaired t-te	0,92858147	permutation FDR (250 perm	1
873	Ddx47~Q9CW	Ddx47	ko vs wt	ko	wt	25,1061709	0,32485679	24,7528146	0,79750922	1,27752925	0,35335633	0,22163784	0,60253025	0,22002115	Unpaired t-te	0,625090695	permutation FDR (250 perm	1
874	Ddx54~Q8K4	Ddx54	ko vs wt	ko	wt	25,2517616	1,06793277	24,0016422	1,05856919	2,37861108	1,2501194	0,63465403	0,26218244	0,5813964	Unpaired t-te	0,171728109	permutation FDR (250 perm	1
875	Ddx5~Q6165	Ddx5	ko vs wt	ko	wt	32,4535374	0,28552658	32,3994842	0,19427215	1,03817758	0,05405324	0,04450353	0,81114963	0,09089903	Unpaired t-te	0,923073718	permutation FDR (250 perm	1
876	Ddx6~P5482	Ddx6	ko vs wt	ko	wt	30,6340034	0,27170024	30,4727372	0,24217405	1,11826819	0,16126623	0,1308734	0,52088463	0,28325846	Unpaired t-te	0,766729161	permutation FDR (250 perm	1
877	Decr1~Q9CQ	Decr1	ko vs wt	ko	wt	33,0175012	0,29922478	33,123841	0,17660408	0,92894186	-0,1063398	-0,0876548	0,63931997	0,19428173	Unpaired t-te	0,846594239	permutation FDR (250 perm	1
878	Degs1~O090	Degs1	ko vs wt	ko	wt	23,8238268	0,2450111	25,3727406	0,51850391	0,34176728	-1,5489138	-1,1119426	0,03760393	1,42476681	Unpaired t-te	0,042985882	permutation FDR (250 perm	1
879	Dek~Q7TNV	Dek	ko vs wt	ko	wt	27,77215	0,44155194	28,1254086	0,52670454	0,78281394	-0,3532586	-0,2434035	0,48012013	0,31865009	Unpaired t-te	0,576430159	permutation FDR (250 perm	1
880	Dennd2b~Q9	Dennd2b	ko vs wt	ko	wt	24,788238	1,01036237	25,2728951	1,1957121	0,71466691	-0,4846571	-0,239077	0,66295566	0,17851552	Unpaired t-te	0,582548937	permutation FDR (250 perm	1
881	Dennd4c~A6	Dennd4c	ko vs wt	ko	wt	26,6262276	0,41825533	26,0421415	0,20303587	1,49908908	0,58408611	0,45598431	0,09666333	1,01473826	Unpaired t-te	0,302637701	permutation FDR (250 perm	1
882	Dennd5a~Q6	Dennd5a	ko vs wt	ko	wt	25,516801	1,27526051	24,8483272	0,62207584	1,58939061	0,66847373	0,35984663	0,47361067	0,32457852	Unpaired t-te	0,409186496	permutation FDR (250 perm	1
883	Deptor~Q57	Deptor	ko vs wt	ko	wt	26,0257136	0,30344035	25,7527011	0,30487003	1,20832833	0,27301252	0,21366065	0,37803097	0,42247262	Unpaired t-te	0,622123748	permutation FDR (250 perm	1
884	Dera~Q91YP	Dera	ko vs wt	ko	wt	25,2906376	1,18598654	24,0835159	0,63170218	2,30876564	1,20712173	0,66414986	0,2022713	0,69406574	Unpaired t-te	0,153065789	permutation FDR (250 perm	1
885	Derl1~Q99J	Derl1	ko vs wt	ko	wt	24,1275464	0,5862591	25,2837683	0,59457932	0,44868599	-1,156222	-0,7509159	0,09489321	1,02276486	Unpaired t-te	0,118629234	permutation FDR (250 perm	1
886	Des~P31001	Des	ko vs wt	ko	wt	33,198008	0,30557268	33,386999	0,22400473	0,87721902	-0,188991	-0,15277	0,46185102	0,33549809	Unpaired t-te	0,726212621	permutation FDR (250 perm	1
887	Dgkz~Q80UP	Dgkz	ko vs wt	ko	wt	24,694881	0,81673687	25,0182576	0,67613945	0,79919719	-0,3233766	-0,1934629	0,65138238	0,186164	Unpaired t-te	0,65477237	permutation FDR (250 perm	1
888	Dguok~Q9Q	Dguok	ko vs wt	ko	wt	26,0535066	1,49913408	27,2832252	0,51599146	0,42640062	-1,2297185	-0,634109	0,26105105	0,58327456	Unpaired t-te	0,174091379	permutation FDR (250 perm	1
889	Dhfr~P00375	Dhfr	ko vs wt	ko	wt	25,2218328	0,89476924	25,2090102	0,09520297	1,00892753	0,01282255	0,00843055	0,98186203	0,00794954	Unpaired t-te	0,985474837	permutation FDR (250 perm	1
890	Dhrs11~Q3U	Dhrs11	ko vs wt	ko	wt	25,6846523	1,30427695	24,2743626	0,67499705	2,65790517	1,41028963	0,7455746	0,17763	0,75048368	Unpaired t-te	0,117926786	permutation FDR (250 perm	1
891	Dhrs1~Q99L	Dhrs1	ko vs wt	ko	wt	29,6087802	0,25025584	29,3087284	0,07362919	1,23118856	0,30005174	0,26010498	0,12744718	0,89466979	Unpaired t-te	0,551340819	permutation FDR (250 perm	1
892	Dhrs4~Q99L	Dhrs4	ko vs wt	ko	wt	28,3513999	0,47241414	28,6610686	0,5363639	0,80682705	-0,3096686	-0,2110673	0,54446666	0,26402871	Unpaired t-te	0,628730588	permutation FDR (250 perm	1
893	Dhrs7b~Q99	Dhrs7b	ko vs wt	ko	wt	27,2078958	0,45837603	25,5511888	1,30318313	3,15296026	1,65670699	0,84580309	0,20792913	0,68208465	Unpaired t-te	0,105899727	permutation FDR (250 perm	1

894	Dhrs7~Q9CXI	Dhrs7	ko vs wt	ko	wt	28,2911583	0,38793758	27,8275703	0,61341989	1,37896701	0,46358794	0,31151604	0,41103038	0,38612608	Unpaired t-te	0,48357116	permutation FDR (250 perm	1
895	Dhx15~O352	Dhx15	ko vs wt	ko	wt	30,1222833	0,32728404	30,2160779	0,31102231	0,93705487	-0,0937946	-0,0727117	0,76102531	0,1186009	Unpaired t-te	0,875762592	permutation FDR (250 perm	1
896	Dhx16~G3X8	Dhx16	ko vs wt	ko	wt	25,0967807	0,25874768	25,2008105	0,0424976	0,93043046	-0,1040298	-0,0902738	0,540439	0,26725332	Unpaired t-te	0,845953361	permutation FDR (250 perm	1
897	Dhx29~Q6PG	Dhx29	ko vs wt	ko	wt	27,4591294	0,05917079	27,520725	0,47955464	0,95820376	-0,0615956	-0,045939	0,8729398	0,05901571	Unpaired t-te	0,926	permutation FDR (250 perm	1
898	Dhx37~Q6NZ	Dhx37	ko vs wt	ko	wt	24,0030704	0,49428603	23,7511004	0,37812675	1,19083212	0,25197004	0,18113502	0,54827019	0,26100536	Unpaired t-te	0,677006952	permutation FDR (250 perm	1
899	Dhx38~Q80X	Dhx38	ko vs wt	ko	wt	24,9900676	0,94392436	24,4887102	0,31341375	1,41554477	0,50135738	0,31565408	0,44311161	0,35348687	Unpaired t-te	0,469912164	permutation FDR (250 perm	1
900	Dhx57~Q6P5	Dhx57	ko vs wt	ko	wt	24,9380872	1,18576168	24,738818	0,91279436	1,1481166	0,19926916	0,10266897	0,84080181	0,07530636	Unpaired t-te	0,818558628	permutation FDR (250 perm	1
901	Dhx58~Q99J8	Dhx58	ko vs wt	ko	wt	24,5140328	0,37917602	23,5103709	0,8023469	2,00508284	1,00366184	0,62412278	0,21111659	0,67547764	Unpaired t-te	0,19606489	permutation FDR (250 perm	1
902	Dhx9~E9QNN	Dhx9	ko vs wt	ko	wt	30,4499877	0,37064834	30,419265	0,17206634	1,02152373	0,03072272	0,02465383	0,90601096	0,04286655	Unpaired t-te	0,955879968	permutation FDR (250 perm	1
903	Diaph1~E9PV	Diaph1	ko vs wt	ko	wt	29,2948935	0,69184501	28,9301199	0,31216029	1,28767958	0,36477365	0,25046803	0,46432398	0,33317889	Unpaired t-te	0,560963433	permutation FDR (250 perm	1
904	Diaph2~O705	Diaph2	ko vs wt	ko	wt	28,9690839	0,71047115	29,6293075	0,3406944	0,63278022	-0,6602236	-0,4473962	0,22856696	0,64098656	Unpaired t-te	0,311795193	permutation FDR (250 perm	1
905	Dicer1~Q8R4	Dicer1	ko vs wt	ko	wt	24,6275392	1,21574441	24,600277	0,71017683	1,01907639	0,02726221	0,01463311	0,97604107	0,01053191	Unpaired t-te	0,973851871	permutation FDR (250 perm	1
906	Dip2a~Q8BW	Dip2a	ko vs wt	ko	wt	26,2013873	0,05809172	26,4314619	0,31211447	0,85259083	-0,2300745	-0,1880874	0,4069071	0,39050474	Unpaired t-te	0,684967147	permutation FDR (250 perm	1
907	Dip2b~Q3UH	Dip2b	ko vs wt	ko	wt	26,8780271	0,27186088	26,8539315	0,50572746	1,01684211	0,02409568	0,01732838	0,9549719	0,02000941	Unpaired t-te	0,970976403	permutation FDR (250 perm	1
908	Dip2c~B2RQ	Dip2c	ko vs wt	ko	wt	27,5091384	0,33972814	27,5457337	0,00751526	0,97495305	-0,0365953	-0,0305926	0,86393023	0,06352133	Unpaired t-te	0,948121597	permutation FDR (250 perm	1
909	Dipk2a~Q3U	Dipk2a	ko vs wt	ko	wt	24,9611483	1,11425487	23,8224027	0,23998667	2,20189485	1,13874557	0,68379963	0,17445683	0,75831203	Unpaired t-te	0,153495481	permutation FDR (250 perm	1
910	Dis3~Q9CSH	Dis3	ko vs wt	ko	wt	26,8437449	0,71225547	27,2159608	0,98121455	0,77259494	-0,3722159	-0,2060389	0,67258686	0,17225162	Unpaired t-te	0,640518735	permutation FDR (250 perm	1
911	Dkc1~Q9ESX	Dkc1	ko vs wt	ko	wt	28,3738452	0,1437516	28,6254048	0,26301105	0,83998787	-0,2515596	-0,2089963	0,3102192	0,50833132	Unpaired t-te	0,640929742	permutation FDR (250 perm	1
912	Dlat~Q8BMF	Dlat	ko vs wt	ko	wt	30,4322818	0,25875743	30,3150338	0,26275834	1,08466382	0,11724796	0,09467619	0,64724552	0,18893095	Unpaired t-te	0,835443833	permutation FDR (250 perm	1
913	Dlc1~Q9R0Z	Dlc1	ko vs wt	ko	wt	26,6808866	0,28803379	25,2100116	0,83979205	2,77189954	1,47087497	0,90981864	0,1225302	0,91175686	Unpaired t-te	0,087244487	permutation FDR (250 perm	1
914	Dld~O08749	Dld	ko vs wt	ko	wt	30,3531772	0,58068263	29,9852062	0,16578808	1,29053647	0,36797091	0,27153272	0,36314501	0,43991992	Unpaired t-te	0,53464301	permutation FDR (250 perm	1
915	Dlg1~Q811D	Dlg1	ko vs wt	ko	wt	27,8780514	0,34570606	27,9716279	0,65079824	0,93719655	-0,0935764	-0,0623177	0,86487598	0,06304616	Unpaired t-te	0,895228903	permutation FDR (250 perm	1
916	Dlst~Q9D2G	Dlst	ko vs wt	ko	wt	29,4739402	0,178801	29,2370214	0,34005762	1,17847306	0,23691878	0,18778047	0,43763167	0,35889125	Unpaired t-te	0,677938061	permutation FDR (250 perm	1
917	Dmd~P11531	Dmd	ko vs wt	ko	wt	34,3254876	0,76887802	35,1236918	0,07878246	0,57506454	-0,7982042	-0,5514769	0,16960285	0,77056685	Unpaired t-te	0,235918579	permutation FDR (250 perm	1
918	Dmpk~D6RI3	Dmpk	ko vs wt	ko	wt	25,5530316	0,45066429	24,9687137	1,07689403	1,49932987	0,58431783	0,32377474	0,53036448	0,27542557	Unpaired t-te	0,476299748	permutation FDR (250 perm	1
919	Dmtn~Q9W	Dmtn	ko vs wt	ko	wt	25,7093598	1,38564436	25,4911252	1,53292422	1,1633092	0,21823461	0,09297681	0,87919533	0,05591463	Unpaired t-te	0,839503714	permutation FDR (250 perm	1
920	Dmxl1~Q6PN	Dmxl1	ko vs wt	ko	wt	26,3600595	0,22365362	26,2829606	0,49242878	1,05489462	0,07709889	0,05622028	0,85094909	0,07009642	Unpaired t-te	0,906919936	permutation FDR (250 perm	1
921	Dnah1~E9Q8	Dnah1	ko vs wt	ko	wt	26,7804382	0,2593065	27,861326	0,3630375	0,47273783	-1,0808878	-0,8332646	0,03001834	1,52261326	Unpaired t-te	0,095144595	permutation FDR (250 perm	1
922	Dnaja1~P63C	Dnaja1	ko vs wt	ko	wt	29,431603	0,32895189	29,3166524	0,53401033	1,08293795	0,11495058	0,08079896	0,80323304	0,09515843	Unpaired t-te	0,863840611	permutation FDR (250 perm	1
923	Dnaja2~Q9Q	Dnaja2	ko vs wt	ko	wt	29,3431105	0,49733246	29,7014866	0,64952178	0,7800421	-0,3583761	-0,2324625	0,54907272	0,26037013	Unpaired t-te	0,594664286	permutation FDR (250 perm	1
924	Dnaja3~Q99I	Dnaja3	ko vs wt	ko	wt	27,7669759	0,35392927	27,2999171	1,02244977	1,38228855	0,46705881	0,26669209	0,58972766	0,2293485	Unpaired t-te	0,557447942	permutation FDR (250 perm	1
925	Dnaja4~Q9JN	Dnaja4	ko vs wt	ko	wt	27,3022503	0,44828461	24,8795222	2,18849005	5,36183996	2,42272816	0,94306628	0,25609352	0,59160142	Unpaired t-te	0,083058252	permutation FDR (250 perm	1
926	Dnajb11~Q9	Dnajb11	ko vs wt	ko	wt	28,3476234	0,06930625	28,6671312	0,80711613	0,80134323	-0,3195078	-0,2032339	0,63222589	0,19912772	Unpaired t-te	0,662511493	permutation FDR (250 perm	1
927	Dnajb1~Q9Q	Dnajb1	ko vs wt	ko	wt	24,4303823	1,00581997	24,7376728	0,97735344	0,80815815	-0,3072905	-0,1615039	0,74928627	0,12535222	Unpaired t-te	0,712969974	permutation FDR (250 perm	1
928	Dnajb4~Q9D	Dnajb4	ko vs wt	ko	wt	30,5688942	0,34130712	30,5424365	0,26239669	1,01850825	0,02645766	0,020822	0,92600077	0,03338865	Unpaired t-te	0,963245204	permutation FDR (250 perm	1
929	Dnajb5~O89	Dnajb5	ko vs wt	ko	wt	27,3064518	0,36472842	27,1325862	0,1695156	1,12807701	0,17386556	0,13995439	0,50820666	0,29395965	Unpaired t-te	0,749806597	permutation FDR (250 perm	1
930	Dnajb6~O54	Dnajb6	ko vs wt	ko	wt	26,222757	0,30407985	27,2390712	0,21402431	0,49437779	-1,0163141	-0,8250737	0,00717077	2,14443423	Unpaired t-te	0,087003361	permutation FDR (250 perm	1
931	Dnajc10~Q9	Dnajc10	ko vs wt	ko	wt	30,0478412	0,2669911	29,9251372	0,39342114	1,08877362	0,12270401	0,09309559	0,72376782	0,14040073	Unpaired t-te	0,840962637	permutation FDR (250 perm	1
932	Dnajc11~Q5	Dnajc11	ko vs wt	ko	wt	27,773113	0,6771038	27,8632588	0,41097094	0,9394278	-0,0901458	-0,0606183	0,860508	0,06524509	Unpaired t-te	0,893758965	permutation FDR (250 perm	1
933	Dnajc13~G3	Dnajc13	ko vs wt	ko	wt	28,8400402	1,32406135	28,2488339	0,83638112	1,50650591	0,59120633	0,30063697	0,56755379	0,24599297	Unpaired t-te	0,484674608	permutation FDR (250 perm	1
934	Dnajc1~Q61	Dnajc1	ko vs wt	ko	wt	25,0739899	0,53166778	23,6434155	0,4790301	2,69554028	1,43057447	0,98178237	0,02929142	1,53325953	Unpaired t-te	0,050033822	permutation FDR (250 perm	1
935	Dnajc3~Q91	Dnajc3	ko vs wt	ko	wt	27,9686305	0,56907684	28,3027618	0,32296539	0,79326167	-0,3341313	-0,2386433	0,44257442	0,35401369	Unpaired t-te	0,577818073	permutation FDR (250 perm	1
936	Dnajc7~Q9Q	Dnajc7	ko vs wt	ko	wt	25,997448	1,22838435	25,1158245	1,49887837	1,84244752	0,88162352	0,38748213	0,53029791	0,27548009	Unpaired t-te	0,383734755	permutation FDR (250 perm	1
937	Dnm1l~Q8K1	Dnm1l	ko vs wt	ko	wt	30,4643161	0,13046394	29,8601627	0,082896	1,52008639	0,60415332	0,55151475	0,00146258	2,8348806	Unpaired t-te	0,219580609	permutation FDR (250 perm	1
938	Dnm1~P3905	Dnm1	ko vs wt	ko	wt	28,0724407	0,26480269	27,7428435	0,18466065	1,2566624	0,32959713	0,27442292	0,16218395	0,78999213	Unpaired t-te	0,52222973	permutation FDR (250 perm	1
939	Dnm2~P3905	Dnm2	ko vs wt	ko	wt	31,1281733	0,3765572	30,9156468	0,13846702	1,15871559	0,2125265	0,17160881	0,42210521	0,37457929	Unpaired t-te	0,695774777	permutation FDR (250 perm	1
940	Dnm3~Q8BZ	Dnm3	ko vs wt	ko	wt	27,5826453	0,83888538	28,2002324	0,42581936	0,65176008	-0,6175871	-0,3932935	0,33112416	0,48000913	Unpaired t-te	0,369945546	permutation FDR (250 perm	1
941	Dnpep~Q9Z2	Dnpep	ko vs wt	ko	wt	26,0543165	1,01629623	24,9152403	0,36806494	2,20239951	1,1390762	0,69375942	0,14956088	0,82518199	Unpaired t-te	0,143948052	permutation FDR (250 perm	1
942	Dnttip1~Q99	Dnttip1	ko vs wt	ko	wt	24,6580629	1,6010788	24,842568	0,60657691	0,87995093	-0,184505	-0,0913823	0,86483767	0,0630654	Unpaired t-te	0,84112167	permutation FDR (250 perm	1
943	Dock10~Q8B	Dock10	ko vs wt	ko	wt	25,8225955	1,43038058	26,5975805	0,26910558	0,58439473	-0,7749849	-0,4194845	0,42206231	0,37462343	Unpaired t-te	0,352876954	permutation FDR (250 perm	1
944	Dock11~A2A	Dock11	ko vs wt	ko	wt	28,9419867	0,09202414	29,3009642	0,5171209	0,77971698	-0,3589775	-0,2621232	0,4301795	0,36635029	Unpaired t-te	0,568747664	permutation FDR (250 perm	1
945	Dock1~Q8BU	Dock1	ko vs wt	ko	wt	29,915784	0,45700881	29,3185254	0,749636	1,51283917	0,59725862	0,37513617	0,38761541	0,41159897	Unpaired t-te	0,405177011	permutation FDR (250 perm	1
946	Dock2~Q8C3	Dock2	ko vs wt	ko	wt	29,2845911	0,89739565	27,5556196	2,12353836	3,31491403	1,72897145	0,66795841	0,37070342	0,4309734	Unpaired t-te	0,17444418	permutation FDR (250 perm	1
947	Dock4~P5976	Dock4	ko vs wt	ko	wt	24,4733908	0,65245195	24,5552015	0,41714216	0,94487106	-0,0818106	-0,0553359	0,87094232	0,06001061	Unpaired t-te	0,903456989	permutation FDR (250 perm	1
948	Dock5~B2RY	Dock5	ko vs wt	ko	wt	27,7000641	0,31925503	27,6600543	0,48989064	1,02812083	0,04000982	0,02873461	0,92497672	0,0338692	Unpaired t-te	0,950178845	permutation FDR (250 perm	1
949	Dock6~Q8VD	Dock6	ko vs wt	ko	wt	25,8862919	0,10293486	26,3140559	0,18747939	0,74341308	-0,427764	-0,3735019	0,06539423	1,18446057	Unpaired t-te	0,409652914	permutation FDR (250 perm	1
950	Dock7~Q8R1	Dock7	ko vs wt	ko	wt	30,2741173	0,19075552	30,4771682	0,05732376	0,86871157	-0,2030508	-0,1817245	0,16311064	0,78751772	Unpaired t-te	0,679761987	permutation FDR (250 perm	1
951	Dock8~Q8C1	Dock8	ko vs wt	ko	wt	25,5484373	1,19098878	26,3536258	0,17682617	0,57228732	-0,8051885	-0,4739488	0,32811087	0,48394738	Unpaired t-te	0,297546985	permutation FDR (250 perm	1
952	Dock9~F8VPI	Dock9	ko vs wt	ko	wt	27,9129163	0,1663914	28,0502253	0,11301382	0,9092135	-0,137309	-0,1220569	0,3219298	0,49223883	Unpaired t-te	0,782681208	permutation FDR (250 perm	1
953	Dok1~P9746	Dok1	ko vs wt	ko	wt	28,2744377	0,29292563	27,5605701	0,20684861	1,64019529	0,7138676	0,58341793	0,02430272	1,6143452	Unpaired t-te	0,198382421	permutation FDR (250 perm	1
954	Dpep1~P314	Dpep1	ko vs wt	ko	wt	29,1847575	1,21104635	29,4723385	0,65405057	0,81927463	-0,2875809	-0,1564374	0,74602618	0,12724593	Unpaired t-te	0,719878097	permutation FDR (250 perm	1
955	Dpm1~O701	Dpm1	ko vs wt	ko	wt	28,6172405	0,75673524	28,0298746	0,38430546	1,50250096	0,58736591	0,38782383	0,30829602	0,51103208	Unpaired t-te	0,376198259	permutation FDR (250 perm	1
956	Dpt~Q9QZZ6	Dpt	ko vs wt	ko	wt	33,0066897	0,34946889	33,138839	0,16607275	0,91247104	-0,1321493	-0,1071378	0,59790777	0,2233658	Unpaired t-te	0,811487236	permutation FDR (250 perm	1
957	Dpy30~Q99L	Dpy30	ko vs wt	ko	wt	25,1584096	0,47052024	25,3245396	0,62130129	0,89123017	-0,16613	-0,1095461	0,7660959	0,11571686	Unpaired t-te	0,810261931	permutation FDR (250 perm	1

958	Dpysl2~O085	Dpysl2	ko vs wt	ko	wt	32,1492388	0,15477506	32,0923037	0,1692563	1,04025352	0,05693518	0,04953634	0,72235848	0,14124723	Unpaired t-te	0,914976171	permutation FDR (250 perm	1
959	Dpysl3~E9PW	Dpysl3	ko vs wt	ko	wt	32,8138585	0,09368481	32,7333157	0,19602179	1,05741579	0,08054278	0,07011109	0,63094593	0,20000786	Unpaired t-te	0,884821264	permutation FDR (250 perm	1
960	Drap1~Q9D6	Drap1	ko vs wt	ko	wt	23,9195018	0,63609789	24,4545575	0,57335492	0,69013207	-0,5350556	-0,34586	0,37686197	0,42381769	Unpaired t-te	0,427188594	permutation FDR (250 perm	1
961	Drg1~P3223	Drg1	ko vs wt	ko	wt	26,8100819	0,81455235	27,0297679	0,20761231	0,85875231	-0,219686	-0,1471773	0,68139804	0,16659912	Unpaired t-te	0,740732617	permutation FDR (250 perm	1
962	Drg2~Q9QXB	Drg2	ko vs wt	ko	wt	27,8883801	0,37887622	27,8063079	0,43692065	1,0585374	0,08207224	0,05953527	0,83932967	0,07606743	Unpaired t-te	0,897119311	permutation FDR (250 perm	1
963	Dsp~E9Q557	Dsp	ko vs wt	ko	wt	30,6717655	0,72843968	32,0994612	0,3705789	0,37172416	-1,4276957	-0,9546489	0,03699215	1,43189044	Unpaired t-te	0,054983535	permutation FDR (250 perm	1
964	Dstn~Q9R0P	Dstn	ko vs wt	ko	wt	34,2458624	0,51039584	34,2873081	0,56323127	0,9716807	-0,0414458	-0,027715	0,93735695	0,028095	Unpaired t-te	0,951250857	permutation FDR (250 perm	1
965	Dst~S4R1P5	Dst	ko vs wt	ko	wt	29,8159995	0,33050643	30,6744008	0,39695636	0,55156344	-0,8584013	-0,6408805	0,06896713	1,16135782	Unpaired t-te	0,172247308	permutation FDR (250 perm	1
966	Dtd1~Q9DD1	Dtd1	ko vs wt	ko	wt	26,4069456	0,57631562	26,5305304	0,28012647	0,917904	-0,1235848	-0,0890873	0,76350227	0,11718967	Unpaired t-te	0,84472268	permutation FDR (250 perm	1
967	Dtna~Q9D2N	Dtna	ko vs wt	ko	wt	30,3900563	0,68578818	31,4541572	0,22678433	0,47827063	-1,0641009	-0,745596	0,06889477	1,16181372	Unpaired t-te	0,122927817	permutation FDR (250 perm	1
968	Dtnb~G3UYN	Dtnb	ko vs wt	ko	wt	25,1055156	0,96425203	25,3785685	0,10070799	0,82756647	-0,2730529	-0,1748941	0,65895628	0,1811434	Unpaired t-te	0,695726594	permutation FDR (250 perm	1
969	Dtx3l~Q3UIR	Dtx3l	ko vs wt	ko	wt	27,5765393	0,61370649	27,3127772	0,24321937	1,20060545	0,26376212	0,18923189	0,53785959	0,26933108	Unpaired t-te	0,664793955	permutation FDR (250 perm	1
970	Dusp12~F7B	Dusp12	ko vs wt	ko	wt	25,0681706	0,66553786	23,9280355	1,15261658	2,20401666	1,14013513	0,59973649	0,29807304	0,52567731	Unpaired t-te	0,20654337	permutation FDR (250 perm	1
971	Dusp3~Q9D7	Dusp3	ko vs wt	ko	wt	30,2697818	0,45426886	30,0288171	0,60832363	1,1817826	0,24096466	0,16023706	0,66117509	0,17968352	Unpaired t-te	0,719003705	permutation FDR (250 perm	1
972	Dvl1~P51141	Dvl1	ko vs wt	ko	wt	24,583423	0,54164721	24,4522343	0,71831053	1,09519566	0,13118864	0,08217409	0,83823238	0,07663557	Unpaired t-te	0,860480175	permutation FDR (250 perm	1
973	Dvl2~Q6083	Dvl2	ko vs wt	ko	wt	24,3923237	0,09225141	25,1045776	0,29351154	0,61036583	-0,7122539	-0,58657	0,06721307	1,1725463	Unpaired t-te	0,226701532	permutation FDR (250 perm	1
974	Dync1h1~Q9	Dync1h1	ko vs wt	ko	wt	34,5373412	0,15318492	34,5295086	0,09577269	1,00544395	0,00783266	0,00704762	0,94667762	0,02379789	Unpaired t-te	0,986947368	permutation FDR (250 perm	1
975	Dync1i2~O8	Dync1i2	ko vs wt	ko	wt	28,7407877	0,18356933	28,6664415	0,33148124	1,05288374	0,07434615	0,05913441	0,79248366	0,10100968	Unpaired t-te	0,900894751	permutation FDR (250 perm	1
976	Dync1li1~Q8	Dync1li1	ko vs wt	ko	wt	29,3504109	0,26566482	29,4427863	0,05392885	0,93797712	-0,0923754	-0,079768	0,59582966	0,22487788	Unpaired t-te	0,864605895	permutation FDR (250 perm	1
977	Dync1li2~Q6	Dync1li2	ko vs wt	ko	wt	26,8789192	0,40691987	26,4920563	0,45117867	1,30754713	0,38686295	0,27708245	0,38443076	0,41518187	Unpaired t-te	0,523958244	permutation FDR (250 perm	1
978	Dync2h1~Q4	Dync2h1	ko vs wt	ko	wt	25,3200109	0,29752554	25,3015696	0,26339652	1,0128646	0,01844133	0,0147134	0,94502197	0,0245581	Unpaired t-te	0,973768124	permutation FDR (250 perm	1
979	Dynll1~P631	Dynll1	ko vs wt	ko	wt	31,6148214	0,09857366	30,9977513	1,01561889	1,53375709	0,61707001	0,3586777	0,4809679	0,31788391	Unpaired t-te	0,442208267	permutation FDR (250 perm	1
980	Dynll2~Q9D0	Dynll2	ko vs wt	ko	wt	27,7158708	0,32974147	28,0668594	0,38297764	0,78404662	-0,3509886	-0,2636976	0,35109007	0,45458146	Unpaired t-te	0,545310618	permutation FDR (250 perm	1
981	Dynlrb1~P62	Dynlrb1	ko vs wt	ko	wt	24,7513845	0,6996563	23,9391725	0,12842225	1,75590164	0,81221203	0,57439603	0,1362618	0,86562588	Unpaired t-te	0,216613414	permutation FDR (250 perm	1
982	Dyrk1a~Q612	Dyrk1a	ko vs wt	ko	wt	25,2474716	0,56489832	25,2374568	0,35372892	1,00696587	0,01001479	0,00709758	0,98150647	0,00810683	Unpaired t-te	0,987109721	permutation FDR (250 perm	1
983	Dysf~Q9ESD	Dysf	ko vs wt	ko	wt	30,2958309	0,17934415	30,6443423	0,13736048	0,78539405	-0,3485114	-0,3051845	0,05854647	1,23249928	Unpaired t-te	0,479257395	permutation FDR (250 perm	1
984	Ebf1~Q5SW	Ebf1	ko vs wt	ko	wt	24,5731829	1,27412629	23,7062446	0,69599943	1,82378836	0,86693832	0,45989835	0,37376617	0,4274	Unpaired t-te	0,296769986	permutation FDR (250 perm	1
985	Ece1~Q4PZA	Ece1	ko vs wt	ko	wt	24,6586961	0,3468666	25,0202587	0,91779983	0,77832111	-0,3615626	-0,2153211	0,63999134	0,1938259	Unpaired t-te	0,636400235	permutation FDR (250 perm	1
986	Ech1~O3545	Ech1	ko vs wt	ko	wt	28,5301937	0,32532092	27,3200067	0,5986821	2,31367627	1,21018702	0,82712284	0,08556881	1,06768448	Unpaired t-te	0,104163078	permutation FDR (250 perm	1
987	Echdc1~Q9D	Echdc1	ko vs wt	ko	wt	26,4661	0,75762685	28,5665072	0,55283519	0,23319241	-2,1004073	-1,3238085	0,01608688	1,79352829	Unpaired t-te	0,013394435	permutation FDR (250 perm	1
988	Echs1~Q8BH	Echs1	ko vs wt	ko	wt	31,3825076	0,35480546	30,9962511	0,09320287	1,30699758	0,38625647	0,31785749	0,15511141	0,80935625	Unpaired t-te	0,469570164	permutation FDR (250 perm	1
989	Eci1~P42125	Eci1	ko vs wt	ko	wt	29,9875894	0,23501847	29,846324	0,49432295	1,10287204	0,14126541	0,10274209	0,73487044	0,13378922	Unpaired t-te	0,826566225	permutation FDR (250 perm	1
990	Eci2~Q9WU	Eci2	ko vs wt	ko	wt	27,6620437	0,34284111	26,7575128	0,13055597	1,87193572	0,9045309	0,74238699	0,01328948	1,87649206	Unpaired t-te	0,122568532	permutation FDR (250 perm	1
991	Ecm1~Q615	Ecm1	ko vs wt	ko	wt	32,640754	0,2198323	32,0911366	0,9104093	1,4636974	0,54961732	0,33186469	0,48480732	0,31443083	Unpaired t-te	0,471495745	permutation FDR (250 perm	1
992	Ecm2~Q5FW	Ecm2	ko vs wt	ko	wt	29,5725538	0,46931641	29,0290875	0,72359847	1,45747011	0,54346629	0,34418843	0,41480915	0,38215168	Unpaired t-te	0,440279846	permutation FDR (250 perm	1
993	Ecpas~Q6PD	Ecpas	ko vs wt	ko	wt	29,3078582	0,55300143	29,0584582	0,34748179	1,1887126	0,24939995	0,17777768	0,56308071	0,24942935	Unpaired t-te	0,681348341	permutation FDR (250 perm	1
994	Edc4~G5E89	Edc4	ko vs wt	ko	wt	28,4482036	0,20318573	28,7732108	0,18691539	0,7982944	-0,3250072	-0,2761974	0,13144056	0,88127059	Unpaired t-te	0,521987097	permutation FDR (250 perm	1
995	Edf1~Q9JMG	Edf1	ko vs wt	ko	wt	24,2993059	0,67481542	25,5267126	0,63426512	0,42708444	-1,2274068	-0,7699753	0,10045873	0,99801233	Unpaired t-te	0,108937868	permutation FDR (250 perm	1
996	Eea1~Q8BL6	Eea1	ko vs wt	ko	wt	27,0189183	0,53312401	26,4771549	1,15909961	1,45575078	0,54176339	0,28886379	0,58650697	0,23172682	Unpaired t-te	0,521600492	permutation FDR (250 perm	1
997	Eed~Q921E6	Eed	ko vs wt	ko	wt	25,8948109	0,12476236	26,179257	0,11335376	0,82105679	-0,2844461	-0,256775	0,05048823	1,29680988	Unpaired t-te	0,551501062	permutation FDR (250 perm	1
998	Eef1a1~P101	Eef1a1	ko vs wt	ko	wt	35,5465231	0,27556127	35,8287718	0,15514918	0,82230834	-0,2822486	-0,236537	0,20540679	0,6873852	Unpaired t-te	0,580629752	permutation FDR (250 perm	1
999	Eef1a2~P626	Eef1a2	ko vs wt	ko	wt	28,9374582	0,8866985	29,0943252	0,25143555	0,89697079	-0,1568671	-0,1017343	0,78776827	0,10360152	Unpaired t-te	0,823085336	permutation FDR (250 perm	1
1000	Eef1b~O702	Eef1b	ko vs wt	ko	wt	28,1122598	0,69901482	28,6180724	0,25131362	0,70426355	-0,5058127	-0,3510227	0,31460573	0,50223338	Unpaired t-te	0,424638492	permutation FDR (250 perm	1
1001	Eef1d~Q80T	Eef1d	ko vs wt	ko	wt	28,7894916	0,2945	28,5978235	0,09249246	1,14208349	0,19166812	0,16213185	0,35457802	0,45028819	Unpaired t-te	0,713925714	permutation FDR (250 perm	1
1002	Eef1g~Q9D8	Eef1g	ko vs wt	ko	wt	31,5551016	0,25266685	31,6161425	0,29878131	0,95857226	-0,0610409	-0,0485708	0,82437436	0,08387552	Unpaired t-te	0,91654667	permutation FDR (250 perm	1
1003	Eef2~P58252	Eef2	ko vs wt	ko	wt	33,9190466	0,28735487	33,7507946	0,10971791	1,12369614	0,16825197	0,142207	0,40841453	0,38889882	Unpaired t-te	0,747321479	permutation FDR (250 perm	1
1004	Eefsec~Q9JH	Eefsec	ko vs wt	ko	wt	27,0490714	0,28867079	27,3486767	0,25257736	0,81247462	-0,2996053	-0,2407854	0,27848558	0,55519729	Unpaired t-te	0,575623685	permutation FDR (250 perm	1
1005	Efemp1~Q8E	Efemp1	ko vs wt	ko	wt	32,3938119	0,56844873	32,265781	0,07922561	1,09280117	0,12803093	0,09605147	0,72490903	0,13971649	Unpaired t-te	0,836437104	permutation FDR (250 perm	1
1006	Efemp2~G5E	Efemp2	ko vs wt	ko	wt	30,827876	0,9827588	31,6300105	0,22022232	0,57350004	-0,8021345	-0,5050027	0,25517925	0,59315464	Unpaired t-te	0,267873654	permutation FDR (250 perm	1
1007	Efhd1~Q9D4	Efhd1	ko vs wt	ko	wt	27,2086739	0,23358787	28,0254245	0,80744046	0,56771915	-0,8167507	-0,5147616	0,28668573	0,54259393	Unpaired t-te	0,279055319	permutation FDR (250 perm	1
1008	Efhd2~Q8C8	Efhd2	ko vs wt	ko	wt	28,9423267	0,67082305	29,1961639	0,35846436	0,83866284	-0,2538372	-0,1735199	0,60794767	0,2161338	Unpaired t-te	0,688859114	permutation FDR (250 perm	1
1009	Efl1~Q8C0D	Efl1	ko vs wt	ko	wt	26,8513867	0,10953132	26,6492421	0,07338076	1,15040716	0,20214456	0,18685929	0,05644994	1,24833651	Unpaired t-te	0,66590122	permutation FDR (250 perm	1
1010	Efr3a~Q8BG	Efr3a	ko vs wt	ko	wt	26,7911269	0,4770451	27,4884931	0,23064269	0,61669704	-0,6973662	-0,5282731	0,08572529	1,06689104	Unpaired t-te	0,239790406	permutation FDR (250 perm	1
1011	Eftud2~O088	Eftud2	ko vs wt	ko	wt	29,2378722	0,20088767	29,1948237	0,60691945	1,03028859	0,0430485	0,02980057	0,93066551	0,03120638	Unpaired t-te	0,950117709	permutation FDR (250 perm	1
1012	Egfl7~Q9QX	Egfl7	ko vs wt	ko	wt	26,1286414	0,18966248	26,1500777	0,40075567	0,98525131	-0,0214363	-0,0164415	0,94880919	0,02282112	Unpaired t-te	0,971930872	permutation FDR (250 perm	1
1013	Egfr~Q0127	Egfr	ko vs wt	ko	wt	27,3971461	0,85907783	26,4721396	0,38933066	1,89869285	0,92500654	0,5902028	0,17043838	0,76843261	Unpaired t-te	0,196481481	permutation FDR (250 perm	1
1014	Ehbp1l1~E9C	Ehbp1l1	ko vs wt	ko	wt	26,4042804	0,7607761	23,5063226	0,65500439	7,4537057	2,89795786	1,76886818	0,00721125	2,14198923	Unpaired t-te	0,004308555	permutation FDR (250 perm	1
1015	Ehd1~Q9WV	Ehd1	ko vs wt	ko	wt	31,5012047	0,59451017	31,076078	0,17216727	1,34269046	0,42512675	0,31163292	0,31251754	0,5051256	Unpaired t-te	0,477498269	permutation FDR (250 perm	1
1016	Ehd2~Q8BH	Ehd2	ko vs wt	ko	wt	34,2581525	0,3712665	34,3676137	0,05568188	0,92693417	-0,1094612	-0,0898743	0,64802122	0,18841077	Unpaired t-te	0,846935528	permutation FDR (250 perm	1
1017	Ehd3~Q9QX	Ehd3	ko vs wt	ko	wt	27,6990941	1,30110844	26,6323551	0,04831066	2,09469335	1,06673906	0,60887918	0,2507065	0,60083441	Unpaired t-te	0,199022736	permutation FDR (250 perm	1
1018	Ehd4~Q9EQ	Ehd4	ko vs wt	ko	wt	31,3210453	0,23143276	31,2001557	0,43679404	1,08740513	0,12088954	0,09045073	0,74517428	0,12774214	Unpaired t-te	0,847897942	permutation FDR (250 perm	1
1019	Ehhadh~Q9D	Ehhadh	ko vs wt	ko	wt	28,1557912	0,6087622	26,9729676	0,90710984	2,2702067	1,18282366	0,68315792	0,1988634	0,70144515	Unpaired t-te	0,156093137	permutation FDR (250 perm	1
1020	Eif1ax~Q8B	Eif1ax	ko vs wt	ko	wt	26,4424121	2,54647594	23,9117992	1,07423366	5,77817115	2,53061294	0,95320659	0,1952796	0,70934313	Unpaired t-te	0,056931954	permutation FDR (250 perm	1
1021	Eif2ak4~Q9C	Eif2ak4	ko vs wt	ko	wt	24,4821749	0,2802264	24,0342648	0,55426192	1,36406279	0,44791006	0,31454286	0,37637506	0,42437917	Unpaired t-te	0,484990596	permutation FDR (250 perm	1

1022	Eif2a~Q8BJW	Eif2a	ko vs wt	ko	wt	28,7471141	0,36178243	28,8679931	0,18755432	0,91962719	-0,120879	-0,0969031	0,64687583	0,18917907	Unpaired t-te	0,829856079	permutation FDR (250 perm	1
1023	Eif2b1~Q99L	Eif2b1	ko vs wt	ko	wt	25,2100815	1,29338833	24,7397551	1,17818459	1,38542286	0,47032638	0,22197941	0,69331641	0,15906852	Unpaired t-te	0,606069905	permutation FDR (250 perm	1
1024	Eif2b3~B1AL	Eif2b3	ko vs wt	ko	wt	26,501377	0,29736141	26,9860737	0,22195191	0,7146473	-0,4846967	-0,393229	0,09233409	1,03463791	Unpaired t-te	0,368531092	permutation FDR (250 perm	1
1025	Eif2b4~Q617	Eif2b4	ko vs wt	ko	wt	26,5522802	0,96856167	26,1491509	0,67917696	1,32237312	0,4031293	0,23206774	0,60801722	0,21608412	Unpaired t-te	0,58721194	permutation FDR (250 perm	1
1026	Eif2b5~Q8CH	Eif2b5	ko vs wt	ko	wt	24,2475155	0,66625946	24,5797071	0,80706454	0,7943289	-0,3321916	-0,1967706	0,656348	0,18286584	Unpaired t-te	0,653992422	permutation FDR (250 perm	1
1027	Eif2d~Q6121	Eif2d	ko vs wt	ko	wt	26,0662867	0,10208056	25,2417327	0,21559709	1,77098755	0,82455407	0,70871721	0,02094699	1,67887847	Unpaired t-te	0,152519951	permutation FDR (250 perm	1
1028	Eif2s1~Q6ZW	Eif2s1	ko vs wt	ko	wt	29,9675862	0,23491015	29,4933631	0,21106535	1,38916994	0,4742231	0,39463854	0,07012068	1,15415385	Unpaired t-te	0,369007059	permutation FDR (250 perm	1
1029	Eif2s2~Q99L	Eif2s2	ko vs wt	ko	wt	28,4819037	0,1481922	28,7063026	0,55391118	0,85595157	-0,2243989	-0,1601808	0,62760659	0,2023125	Unpaired t-te	0,730292364	permutation FDR (250 perm	1
1030	Eif2s3x~Q9Z	Eif2s3x	ko vs wt	ko	wt	31,2894873	0,28516703	31,5110668	0,30904595	0,85762597	-0,2215795	-0,1739778	0,46297281	0,33444451	Unpaired t-te	0,691645773	permutation FDR (250 perm	1
1031	Eif3a~P2311	Eif3a	ko vs wt	ko	wt	32,2156891	0,30935427	31,9199436	0,29680349	1,2275191	0,29574547	0,23185115	0,33842967	0,47053158	Unpaired t-te	0,590271399	permutation FDR (250 perm	1
1032	Eif3b~Q8JZQ	Eif3b	ko vs wt	ko	wt	30,5482963	0,4243616	30,3957719	0,25376108	1,11151268	0,1525244	0,11699478	0,63704845	0,19582754	Unpaired t-te	0,7926756	permutation FDR (250 perm	1
1033	Eif3c~Q8R1B	Eif3c	ko vs wt	ko	wt	31,2101456	0,21340104	30,8636014	0,36820472	1,27151127	0,34654425	0,26904762	0,31791343	0,49769112	Unpaired t-te	0,54606326	permutation FDR (250 perm	1
1034	Eif3d~O7019	Eif3d	ko vs wt	ko	wt	29,9631817	0,17655518	29,9914895	0,18783078	0,98056977	-0,0283078	-0,0242481	0,87374746	0,05861408	Unpaired t-te	0,956771376	permutation FDR (250 perm	1
1035	Eif3e~P6022	Eif3e	ko vs wt	ko	wt	28,7082302	0,3871941	27,815343	0,19079654	1,85688849	0,89288718	0,70802085	0,0210833	1,67606142	Unpaired t-te	0,134065124	permutation FDR (250 perm	1
1036	Eif3f~Q9DCH	Eif3f	ko vs wt	ko	wt	30,2542753	0,41267369	30,1394791	0,3961921	1,08282207	0,1147962	0,08392981	0,76915899	0,11398388	Unpaired t-te	0,854883594	permutation FDR (250 perm	1
1037	Eif3g~Q9Z1D	Eif3g	ko vs wt	ko	wt	24,0632781	0,91334959	24,8794726	1,63381134	0,56793807	-0,8161945	-0,3595671	0,56854871	0,24523233	Unpaired t-te	0,426423613	permutation FDR (250 perm	1
1038	Eif3h~Q91W	Eif3h	ko vs wt	ko	wt	29,2376145	0,33459522	28,6327376	0,28031837	1,52084896	0,60487688	0,47375184	0,08344904	1,07857867	Unpaired t-te	0,283494172	permutation FDR (250 perm	1
1039	Eif3i~Q9QZD	Eif3i	ko vs wt	ko	wt	28,8667565	0,20025069	28,6094073	0,05019558	1,19528045	0,25734916	0,22958327	0,10927489	0,9614796	Unpaired t-te	0,599214731	permutation FDR (250 perm	1
1040	Eif3k~Q9DBZ	Eif3k	ko vs wt	ko	wt	27,6789872	0,7292926	27,6747332	0,18241023	1,002953	0,00425401	0,00295341	0,99281149	0,00313321	Unpaired t-te	0,994443288	permutation FDR (250 perm	1
1041	Eif3l~Q8QZY	Eif3l	ko vs wt	ko	wt	30,0488488	0,50318374	29,583068	0,27002076	1,3810646	0,46578081	0,34562703	0,24015036	0,61951676	Unpaired t-te	0,426373013	permutation FDR (250 perm	1
1042	Eif3m~Q99J	Eif3m	ko vs wt	ko	wt	29,554518	0,31898373	29,2274109	0,11141794	1,25449531	0,32710708	0,27251897	0,17784577	0,74995647	Unpaired t-te	0,530835784	permutation FDR (250 perm	1
1043	Eif4a1~P608	Eif4a1	ko vs wt	ko	wt	32,4252808	0,33896307	32,1365493	0,08648502	1,22156573	0,28873149	0,23960466	0,24004274	0,61971143	Unpaired t-te	0,583220826	permutation FDR (250 perm	1
1044	Eif4a2~P106	Eif4a2	ko vs wt	ko	wt	28,0917967	0,39425437	27,9219272	0,62826233	1,12495668	0,16986945	0,11330913	0,75573423	0,12163091	Unpaired t-te	0,805644247	permutation FDR (250 perm	1
1045	Eif4a3~Q91V	Eif4a3	ko vs wt	ko	wt	29,3040166	0,50596286	28,9200809	0,34932894	1,3048968	0,38393571	0,27770018	0,36168625	0,441668	Unpaired t-te	0,518333436	permutation FDR (250 perm	1
1046	Eif4e2~Q8BM	Eif4e2	ko vs wt	ko	wt	25,0422034	1,56265412	23,4653908	0,17189076	2,98310058	1,57681262	0,8254054	0,17848057	0,74840905	Unpaired t-te	0,100062702	permutation FDR (250 perm	1
1047	Eif4e3~Q9DE	Eif4e3	ko vs wt	ko	wt	26,8452235	0,55891776	25,2603116	1,28336491	2,99989493	1,58491197	0,80733437	0,21573718	0,66607501	Unpaired t-te	0,115960591	permutation FDR (250 perm	1
1048	Eif4e~P6307	Eif4e	ko vs wt	ko	wt	27,4385619	0,46626676	27,4534158	0,12755691	0,98975687	-0,0148539	-0,0115693	0,96101564	0,01726954	Unpaired t-te	0,980465238	permutation FDR (250 perm	1
1049	Eif4g1~Q6N	Eif4g1	ko vs wt	ko	wt	30,7231202	0,09443017	30,6797657	0,21996121	1,03050716	0,04335453	0,03722012	0,81268236	0,09007917	Unpaired t-te	0,937919873	permutation FDR (250 perm	1
1050	Eif4g2~G3XA	Eif4g2	ko vs wt	ko	wt	29,854182	0,12715731	29,794117	0,53093094	1,04251274	0,06006502	0,04344555	0,88872907	0,05123061	Unpaired t-te	0,928659212	permutation FDR (250 perm	1
1051	Eif4g3~Q80X	Eif4g3	ko vs wt	ko	wt	26,9669074	0,3602376	27,0033431	0,28945616	0,97506101	-0,0364356	-0,0282052	0,90566153	0,04303408	Unpaired t-te	0,950146213	permutation FDR (250 perm	1
1052	Eif4h~Q9WU	Eif4h	ko vs wt	ko	wt	26,0888842	2,39154238	27,4154914	0,48644103	0,39870477	-1,3266072	-0,547516	0,41312544	0,38391806	Unpaired t-te	0,235412287	permutation FDR (250 perm	1
1053	Eif5a~P6324	Eif5a	ko vs wt	ko	wt	29,611522	0,34426633	29,3598613	0,02086755	1,1905768	0,25166069	0,20983804	0,29505668	0,53009455	Unpaired t-te	0,637029007	permutation FDR (250 perm	1
1054	Eif5b~Q05D	Eif5b	ko vs wt	ko	wt	28,8779052	0,43147692	28,9681257	0,4099513	0,93937916	-0,0902205	-0,0652725	0,82390731	0,08412165	Unpaired t-te	0,88748066	permutation FDR (250 perm	1
1055	Eif5~P59325	Eif5	ko vs wt	ko	wt	28,7462226	0,31659288	28,7349779	0,19932107	1,00782469	0,0112447	0,00913599	0,96303596	0,01635749	Unpaired t-te	0,984586945	permutation FDR (250 perm	1
1056	Elavl1~P703	Elavl1	ko vs wt	ko	wt	29,5155348	0,16545239	29,4311345	0,26802033	1,06024691	0,08440028	0,06962389	0,71734603	0,1442713	Unpaired t-te	0,883977217	permutation FDR (250 perm	1
1057	Elmo1~Q8BF	Elmo1	ko vs wt	ko	wt	28,5882116	0,4611286	28,2399822	0,87215951	1,27299731	0,34822937	0,20830574	0,64295097	0,19182215	Unpaired t-te	0,64271115	permutation FDR (250 perm	1
1058	Elmo2~Q8BH	Elmo2	ko vs wt	ko	wt	25,3700843	1,57329912	26,7233458	0,40981949	0,3914062	-1,3532615	-0,6927548	0,23657853	0,62602467	Unpaired t-te	0,147882109	permutation FDR (250 perm	1
1059	Eln~P54320	Eln	ko vs wt	ko	wt	32,6294324	0,24299407	32,7445397	0,15743085	0,92331366	-0,1151073	-0,0976236	0,54873315	0,2606388	Unpaired t-te	0,827881964	permutation FDR (250 perm	1
1060	Elob~P62869	Elob	ko vs wt	ko	wt	24,5460676	0,74695944	25,2901726	0,87467796	0,59703816	-0,744105	-0,4242335	0,38206609	0,4178615	Unpaired t-te	0,341306931	permutation FDR (250 perm	1
1061	Eloc~P83940	Eloc	ko vs wt	ko	wt	27,3027641	0,7893651	25,4991394	1,17969616	3,49096812	1,8036247	0,92467611	0,14870497	0,82767451	Unpaired t-te	0,071486555	permutation FDR (250 perm	1
1062	Elp1~Q7TT3	Elp1	ko vs wt	ko	wt	27,8132828	0,60733486	27,5425365	1,27227499	1,20643168	0,27074622	0,13774553	0,79986455	0,09698355	Unpaired t-te	0,764330523	permutation FDR (250 perm	1
1063	Elp3~Q9CZX	Elp3	ko vs wt	ko	wt	26,5113418	0,28107773	26,2736863	0,14959458	1,17907506	0,23765556	0,1990896	0,27670759	0,55797893	Unpaired t-te	0,644973099	permutation FDR (250 perm	1
1064	Emc1~Q8C7	Emc1	ko vs wt	ko	wt	28,5858367	1,03604037	27,8470864	0,77061051	1,66872975	0,73875033	0,40834254	0,40359029	0,39405929	Unpaired t-te	0,35203399	permutation FDR (250 perm	1
1065	Emc3~Q99KI	Emc3	ko vs wt	ko	wt	25,3922464	1,4087003	24,3862196	1,17379463	2,00837231	1,00602674	0,46531006	0,4281117	0,3684429	Unpaired t-te	0,292087201	permutation FDR (250 perm	1
1066	Emc7~Q9EP	Emc7	ko vs wt	ko	wt	26,1686157	0,87842434	26,4759408	0,40853386	0,80813873	-0,3073251	-0,19406	0,62317295	0,2053914	Unpaired t-te	0,654609152	permutation FDR (250 perm	1
1067	Emc8~O703	Emc8	ko vs wt	ko	wt	24,8039943	1,44104456	24,3421775	0,25783088	1,37727512	0,46181678	0,24939767	0,62232741	0,20598107	Unpaired t-te	0,570649804	permutation FDR (250 perm	1
1068	Emd~O0857	Emd	ko vs wt	ko	wt	25,9188908	2,18363339	26,6262202	1,44609591	0,61245279	-0,7073295	-0,2696362	0,68119404	0,16672916	Unpaired t-te	0,529870018	permutation FDR (250 perm	1
1069	Emilin1~Q99	Emilin1	ko vs wt	ko	wt	33,830608	0,19525315	33,0381354	0,3364079	1,7320404	0,79247258	0,62733545	0,05967373	1,22421685	Unpaired t-te	0,189483041	permutation FDR (250 perm	1
1070	Emilin2~Q8K	Emilin2	ko vs wt	ko	wt	28,5407812	0,90779471	28,0554332	0,90451566	1,39992352	0,48534801	0,26566685	0,58670712	0,23157864	Unpaired t-te	0,539765425	permutation FDR (250 perm	1
1071	Eml1~Q05B	Eml1	ko vs wt	ko	wt	29,9764859	0,73123103	30,6565215	0,0918249	0,62414989	-0,6800356	-0,4765022	0,20555821	0,68706516	Unpaired t-te	0,295736325	permutation FDR (250 perm	1
1072	Eml2~Q7TN	Eml2	ko vs wt	ko	wt	26,6154527	0,97225857	26,5186891	1,07181048	1,06937181	0,09676355	0,04979791	0,92322531	0,0346923	Unpaired t-te	0,914669523	permutation FDR (250 perm	1
1073	Eml3~Q8VC	Eml3	ko vs wt	ko	wt	24,5996249	0,10162309	24,7070029	0,42879623	0,92827364	-0,1073779	-0,0820412	0,7591801	0,11965519	Unpaired t-te	0,866775109	permutation FDR (250 perm	1
1074	Enah~Q0317	Enah	ko vs wt	ko	wt	29,3731842	0,96735706	29,008635	0,18139028	1,28747927	0,36454921	0,23174783	0,56594315	0,2472272	Unpaired t-te	0,597825751	permutation FDR (250 perm	1
1075	Endod1~Q8C	Endod1	ko vs wt	ko	wt	29,3461629	0,28619997	30,1621369	0,17565183	0,56802247	-0,815974	-0,6761956	0,01101631	1,95796391	Unpaired t-te	0,14680433	permutation FDR (250 perm	1
1076	Eno1~P1718	Eno1	ko vs wt	ko	wt	31,868342	0,29757022	31,863074	0,42223441	1,00365817	0,005268	0,00391828	0,98867607	0,00494598	Unpaired t-te	0,992916992	permutation FDR (250 perm	1
1077	Eno3~P2155	Eno3	ko vs wt	ko	wt	27,2236251	0,73209543	24,0283917	0,4986715	9,15927445	3,19523332	2,06084737	0,00214273	2,66903236	Unpaired t-te	0,003050109	permutation FDR (250 perm	1
1078	Enpep~P164	Enpep	ko vs wt	ko	wt	27,9297241	0,7627991	27,8848734	0,4219481	1,03157639	0,04485066	0,02927678	0,93617485	0,02864303	Unpaired t-te	0,948831924	permutation FDR (250 perm	1
1079	Enpp1~P068	Enpp1	ko vs wt	ko	wt	29,0634772	0,18250862	28,5479253	1,04620836	1,42954093	0,51555193	0,29506516	0,55909516	0,25251426	Unpaired t-te	0,522353808	permutation FDR (250 perm	1
1080	Enpp3~Q6DY	Enpp3	ko vs wt	ko	wt	24,8423346	0,83394312	24,4174623	1,22295058	1,34245364	0,42487226	0,21352943	0,69480164	0,15813917	Unpaired t-te	0,629518824	permutation FDR (250 perm	1
1081	Entpd1~P557	Entpd1	ko vs wt	ko	wt	29,3507094	0,88862831	29,6377768	0,39671222	0,81956633	-0,2870674	-0,1811458	0,64664088	0,18933684	Unpaired t-te	0,678255282	permutation FDR (250 perm	1
1082	Entpd2~O55	Entpd2	ko vs wt	ko	wt	27,4328904	0,99560273	26,5678106	0,95550299	1,82144038	0,86507977	0,45842374	0,37992529	0,4203018	Unpaired t-te	0,301624387	permutation FDR (250 perm	1
1083	Epb41l1~A2A	Epb41l1	ko vs wt	ko	wt	27,8958151	0,30996079	27,1707552	0,36959354	1,65296931	0,72505994	0,55064731	0,08810429	1,05500297	Unpaired t-te	0,229472036	permutation FDR (250 perm	1
1084	Epb41l2~O7	Epb41l2	ko vs wt	ko	wt	31,3636366	0,14693479	31,4070121	0,15454697	0,97038186	-0,0433755	-0,0381041	0,76905517	0,1140425	Unpaired t-te	0,933907545	permutation FDR (250 perm	1
1085	Epb41l3~Q9	Epb41l3	ko vs wt	ko	wt	27,44043	0,11033053	28,0732875	0,89707144	0,64489781	-0,6328575	-0,3864742	0,42369565	0,37294599	Unpaired t-te	0,410484789	permutation FDR (250 perm	1

1086	Epb41l5~Q8	Epb41l5	ko vs wt	ko	wt	23,6378703	0,3838838	23,7274058	0,36864295	0,93982528	-0,0895355	-0,0667102	0,8053604	0,09400973	Unpaired t-t	0,885585478	permutation FDR (250 perm	1
1087	Epb41~A2A8	Epb41	ko vs wt	ko	wt	30,5368252	0,74161039	30,0244283	0,18071863	1,4264181	0,51239691	0,35415109	0,32348357	0,49014777	Unpaired t-t	0,425366266	permutation FDR (250 perm	1
1088	Epb42~P492	Epb42	ko vs wt	ko	wt	28,7502823	1,03393765	28,2662776	0,46553715	1,39862068	0,48400474	0,2878081	0,51292092	0,28994958	Unpaired t-t	0,506129693	permutation FDR (250 perm	1
1089	Ephb2~P547	Ephb2	ko vs wt	ko	wt	24,6058899	1,08262433	25,8839933	0,06561316	0,41233723	-1,2781034	-0,7856679	0,1331587	0,87563044	Unpaired t-t	0,115915808	permutation FDR (250 perm	1
1090	Ephx1~Q9D3	Ephx1	ko vs wt	ko	wt	32,4822897	0,19350534	32,3998644	0,18050014	1,05879648	0,08242531	0,07047175	0,64981671	0,18720913	Unpaired t-t	0,879490079	permutation FDR (250 perm	1
1091	Ephx2~P3491	Ephx2	ko vs wt	ko	wt	28,4593168	0,90346548	28,712164	0,76484316	0,83923851	-0,2528472	-0,1443701	0,75091219	0,12441085	Unpaired t-t	0,741342001	permutation FDR (250 perm	1
1092	Eppk1~Q8R0	Eppk1	ko vs wt	ko	wt	27,1352778	0,58545898	27,3195756	0,28177267	0,88007732	-0,1842978	-0,1323629	0,65992713	0,18050402	Unpaired t-t	0,764335114	permutation FDR (250 perm	1
1093	Eprs1~Q8CG	Eprs1	ko vs wt	ko	wt	32,517539	0,10985949	32,5943063	0,08559384	0,94817984	-0,0767674	-0,0705796	0,42222411	0,37445698	Unpaired t-t	0,878712344	permutation FDR (250 perm	1
1094	Eps15l1~Q6	Eps15l1	ko vs wt	ko	wt	26,820439	0,58748104	27,0148505	0,79960113	0,87392933	-0,1944115	-0,1171621	0,78518567	0,10502764	Unpaired t-t	0,797611158	permutation FDR (250 perm	1
1095	Eps15~P425	Eps15	ko vs wt	ko	wt	27,5628572	0,32260155	27,22986	0,5342672	1,25962753	0,3329972	0,2343067	0,48739123	0,31212229	Unpaired t-t	0,596936347	permutation FDR (250 perm	1
1096	Eps8l2~Q99	Eps8l2	ko vs wt	ko	wt	26,8442842	0,49028651	26,2360515	0,24925361	1,52439073	0,60823274	0,45613375	0,13127523	0,88181722	Unpaired t-t	0,301913104	permutation FDR (250 perm	1
1097	Epx~P49290	Epx	ko vs wt	ko	wt	26,1340369	2,09307334	24,8470289	0,33925003	2,4402145	1,28700797	0,57661228	0,36790849	0,43426019	Unpaired t-t	0,216072425	permutation FDR (250 perm	1
1098	Erap1~Q9EQ	Erap1	ko vs wt	ko	wt	26,2954565	0,84847135	24,7404346	0,59447372	2,93838189	1,55502191	0,94501611	0,06105862	1,21425304	Unpaired t-t	0,055759956	permutation FDR (250 perm	1
1099	Erc1~V9GXF	Erc1	ko vs wt	ko	wt	28,0590991	0,1195571	28,0170204	0,25362855	1,02959625	0,04207871	0,03529597	0,84271893	0,07431725	Unpaired t-t	0,94140403	permutation FDR (250 perm	1
1100	Ercc1~P0790	Ercc1	ko vs wt	ko	wt	24,6667939	0,45949674	24,2146227	0,7243976	1,36809757	0,45217112	0,28675609	0,48912589	0,31057935	Unpaired t-t	0,518624769	permutation FDR (250 perm	1
1101	Ercc3~P4913	Ercc3	ko vs wt	ko	wt	24,6280263	0,50599905	24,1124508	0,49041924	1,42956424	0,51557546	0,35472969	0,31391248	0,50319142	Unpaired t-t	0,41694363	permutation FDR (250 perm	1
1102	Ercc4~Q9QZ	Ercc4	ko vs wt	ko	wt	24,1237456	0,58042144	24,3745003	0,12838717	0,84045664	-0,2507547	-0,1861322	0,51629606	0,28710119	Unpaired t-t	0,675228273	permutation FDR (250 perm	1
1103	Ergic1~Q9DC	Ergic1	ko vs wt	ko	wt	28,0017265	0,51676607	28,2199981	0,21370989	0,85959468	-0,2182715	-0,1635676	0,54716193	0,26188413	Unpaired t-t	0,708993419	permutation FDR (250 perm	1
1104	Erh~P84089	Erh	ko vs wt	ko	wt	24,5257779	0,89643442	24,0375614	0,66864093	1,4027097	0,48821646	0,28701681	0,51755704	0,28604178	Unpaired t-t	0,50491964	permutation FDR (250 perm	1
1105	Eri3~Q8C460	Eri3	ko vs wt	ko	wt	25,192042	1,212333	26,5868094	0,00870671	0,38030599	-1,3947674	-0,8204669	0,14031295	0,85290224	Unpaired t-t	0,103809087	permutation FDR (250 perm	1
1106	Erlin1~Q91X	Erlin1	ko vs wt	ko	wt	25,0891763	1,00825961	24,768719	0,75093191	1,24872631	0,3204573	0,17923509	0,70123949	0,15413363	Unpaired t-t	0,679537439	permutation FDR (250 perm	1
1107	Erlin2~Q8BF	Erlin2	ko vs wt	ko	wt	28,4060857	0,78903105	28,8036321	0,10278685	0,75914828	-0,3975464	-0,2720482	0,44939829	0,34736859	Unpaired t-t	0,539137183	permutation FDR (250 perm	1
1108	Ero1a~Q8R1	Ero1a	ko vs wt	ko	wt	25,386792	1,27363179	24,264623	0,51825996	2,17673979	1,12216896	0,61603833	0,23937423	0,62092261	Unpaired t-t	0,181632653	permutation FDR (250 perm	1
1109	Erp44~Q9D1	Erp44	ko vs wt	ko	wt	29,2152818	0,85535097	27,5663824	0,84119549	3,1359432	1,64889943	0,92995408	0,09456887	1,0242518	Unpaired t-t	0,061490736	permutation FDR (250 perm	1
1110	Esd~Q9R0P3	Esd	ko vs wt	ko	wt	29,1807295	0,87894495	28,3135522	0,66398123	1,82409056	0,86717736	0,51271632	0,26597566	0,57515811	Unpaired t-t	0,250115244	permutation FDR (250 perm	1
1111	Esyt1~Q3U7	Esyt1	ko vs wt	ko	wt	31,9620137	0,27021494	31,9565265	0,20102839	1,00381071	0,00548725	0,00453096	0,98027552	0,00865184	Unpaired t-t	0,991832335	permutation FDR (250 perm	1
1112	Esyt2~Q3TZ	Esyt2	ko vs wt	ko	wt	30,9526561	0,15902879	31,1336797	0,3106147	0,88207695	-0,1810236	-0,146216	0,50776501	0,29433723	Unpaired t-t	0,74802708	permutation FDR (250 perm	1
1113	Etf1~Q8BWY	Etf1	ko vs wt	ko	wt	29,101285	0,24359027	29,275388	0,45470233	0,88631842	-0,174103	-0,1288759	0,6564745	0,18278214	Unpaired t-t	0,778784555	permutation FDR (250 perm	1
1114	Etfa~Q99LC5	Etfa	ko vs wt	ko	wt	32,4607129	0,3272471	32,8295916	0,14969785	0,7743841	-0,3688788	-0,3032127	0,15554704	0,80813825	Unpaired t-t	0,483262233	permutation FDR (250 perm	1
1115	Etfb~Q9DCW	Etfb	ko vs wt	ko	wt	31,4724286	0,27504744	31,4600824	0,14417037	1,00859444	0,01234618	0,01038624	0,95048911	0,02205285	Unpaired t-t	0,982393939	permutation FDR (250 perm	1
1116	Etfdh~Q921	Etfdh	ko vs wt	ko	wt	30,6513214	0,3872565	30,9813777	0,27807493	0,79550544	-0,3300563	-0,2543306	0,31834809	0,49709776	Unpaired t-t	0,553188599	permutation FDR (250 perm	1
1117	Ethe1~Q9DC	Ethe1	ko vs wt	ko	wt	26,5511255	0,55891688	26,4291474	0,49475588	1,08822595	0,12197814	0,08264425	0,80874282	0,09218956	Unpaired t-t	0,856818829	permutation FDR (250 perm	1
1118	Etv6~P97360	Etv6	ko vs wt	ko	wt	24,5469572	0,70433953	23,9761988	0,30761059	1,48530412	0,57075836	0,39061762	0,27753895	0,55667606	Unpaired t-t	0,374967136	permutation FDR (250 perm	1
1119	Evi5~E9PWR	Evi5	ko vs wt	ko	wt	27,1160881	0,14019561	25,2991672	1,10566258	3,52328453	1,81692099	1,01731281	0,14431963	0,8406746	Unpaired t-t	0,068636877	permutation FDR (250 perm	1
1120	Ewsr1~Q615	Ewsr1	ko vs wt	ko	wt	28,4960952	0,2535064	28,0388433	0,05056844	1,37292414	0,45725192	0,39738003	0,04860018	1,31336214	Unpaired t-t	0,376246484	permutation FDR (250 perm	1
1121	Exoc1~Q8R3	Exoc1	ko vs wt	ko	wt	29,9678873	0,55856464	29,2880355	0,39387795	1,60197515	0,67985177	0,47671828	0,17166783	0,76531109	Unpaired t-t	0,27967234	permutation FDR (250 perm	1
1122	Exoc2~Q9D4	Exoc2	ko vs wt	ko	wt	27,7499506	0,28440852	27,755628	0,34669564	0,99607248	-0,0056774	-0,0043839	0,98564078	0,00628134	Unpaired t-t	0,992330122	permutation FDR (250 perm	1
1123	Exoc3~Q6KA	Exoc3	ko vs wt	ko	wt	27,2623004	0,34779541	27,1135023	0,16984608	1,10864552	0,14879815	0,1205844	0,55483876	0,25583321	Unpaired t-t	0,786742666	permutation FDR (250 perm	1
1124	Exoc4~O353	Exoc4	ko vs wt	ko	wt	28,9222426	0,10054661	28,6665029	0,39865035	1,19394775	0,25573971	0,19858595	0,46155973	0,33577209	Unpaired t-t	0,667235772	permutation FDR (250 perm	1
1125	Exoc5~Q3TP	Exoc5	ko vs wt	ko	wt	28,0427327	0,54593684	27,6328179	0,86471714	1,32860731	0,40991476	0,24285379	0,5924544	0,22734507	Unpaired t-t	0,582651901	permutation FDR (250 perm	1
1126	Exoc6b~A6H	Exoc6b	ko vs wt	ko	wt	27,0184386	0,4716065	27,2330522	0,52320064	0,86177693	-0,2146136	-0,1470606	0,66480757	0,17730404	Unpaired t-t	0,739183258	permutation FDR (250 perm	1
1127	Exoc6~Q3U9	Exoc6	ko vs wt	ko	wt	24,0833291	1,14018656	24,2868732	0,46139351	0,86841461	-0,2035441	-0,1173368	0,79465035	0,09982392	Unpaired t-t	0,79371923	permutation FDR (250 perm	1
1128	Exoc7~O352	Exoc7	ko vs wt	ko	wt	28,369589	0,2851842	28,1506392	0,43410684	1,16388605	0,21894982	0,16238583	0,57235599	0,24233376	Unpaired t-t	0,717306332	permutation FDR (250 perm	1
1129	Exoc8~Q6PG	Exoc8	ko vs wt	ko	wt	27,4838579	0,62455022	27,4195722	0,23550163	1,04556712	0,06428568	0,04601104	0,8790239	0,05599932	Unpaired t-t	0,920983431	permutation FDR (250 perm	1
1130	Exosc10~P56	Exosc10	ko vs wt	ko	wt	26,9332745	0,33366213	26,8530962	0,09761166	1,05714865	0,08017826	0,06655831	0,71655197	0,1447523	Unpaired t-t	0,886367524	permutation FDR (250 perm	1
1131	Exosc1~Q9D	Exosc1	ko vs wt	ko	wt	25,8597686	0,17875677	25,2970547	0,34244713	1,47704506	0,56271383	0,44545883	0,13090311	0,88305004	Unpaired t-t	0,332991418	permutation FDR (250 perm	1
1132	Exosc2~Q8V	Exosc2	ko vs wt	ko	wt	25,6189713	0,3619926	24,4550662	0,63147853	2,24063109	1,16390513	0,77956783	0,10294411	0,98739851	Unpaired t-t	0,120278863	permutation FDR (250 perm	1
1133	Exosc5~Q9C	Exosc5	ko vs wt	ko	wt	26,5018134	0,11778691	25,7765001	0,502112	1,65325968	0,72531335	0,53273076	0,17380756	0,75993133	Unpaired t-t	0,267842124	permutation FDR (250 perm	1
1134	Exosc8~Q9D	Exosc8	ko vs wt	ko	wt	25,0065803	0,94319545	24,2609305	0,73161854	1,67672925	0,74564975	0,42581454	0,36741692	0,43484084	Unpaired t-t	0,331971154	permutation FDR (250 perm	1
1135	Ezr~P26040	Ezr	ko vs wt	ko	wt	27,0668273	0,53823376	26,9319671	0,7172508	1,09798645	0,13486024	0,08456237	0,83338828	0,07915261	Unpaired t-t	0,856051465	permutation FDR (250 perm	1
1136	F10~Q3U3V1	F10	ko vs wt	ko	wt	27,9277547	0,93008655	23,8627082	0,16651682	16,7378986	4,06504651	2,62304522	0,00373083	2,42819468	Unpaired t-t	0,002418605	permutation FDR (250 perm	1
1137	F12~Q80YC5	F12	ko vs wt	ko	wt	27,2948427	1,00407189	27,8668994	0,02425248	0,67265719	-0,5720566	-0,3620715	0,39655686	0,40169453	Unpaired t-t	0,420916368	permutation FDR (250 perm	1
1138	F13a1~Q8B	F13a1	ko vs wt	ko	wt	32,0989196	0,40325298	31,5436576	0,42709729	1,46943544	0,55526197	0,40197717	0,2171508	0,66323856	Unpaired t-t	0,364084992	permutation FDR (250 perm	1
1139	F13b~Q0796	F13b	ko vs wt	ko	wt	29,8716678	0,98865487	28,3417355	0,45295898	2,88772274	1,52993223	0,92469756	0,0721552	1,14173239	Unpaired t-t	0,061861449	permutation FDR (250 perm	1
1140	F2~P19221	F2	ko vs wt	ko	wt	30,5616105	1,21871333	28,8928576	0,87876304	3,17939629	1,66875285	0,86074876	0,1359539	0,86660834	Unpaired t-t	0,076461921	permutation FDR (250 perm	1
1141	F5~O88783	F5	ko vs wt	ko	wt	26,9898272	2,5955754	24,2883418	1,06009422	6,50471319	2,70148545	1,00968194	0,1774348	0,7509612	Unpaired t-t	0,046308492	permutation FDR (250 perm	1
1142	F9~P16294	F9	ko vs wt	ko	wt	24,3575518	0,4853627	24,9546443	0,90925574	0,6610849	-0,5970925	-0,3509512	0,4619308	0,33542308	Unpaired t-t	0,436828562	permutation FDR (250 perm	1
1143	FAM120A~Q	FAM120A	ko vs wt	ko	wt	30,3747779	0,15893447	30,5516928	0,30203329	0,88459261	-0,1769149	-0,1435476	0,50663645	0,29530356	Unpaired t-t	0,752191441	permutation FDR (250 perm	1
1144	Fabp3~P114	Fabp3	ko vs wt	ko	wt	23,7302215	0,39867923	25,9261524	0,50861238	0,21825235	-2,1959309	-1,5388494	0,00914072	2,03901955	Unpaired t-t	0,009233716	permutation FDR (250 perm	1
1145	Fabp4~P041	Fabp4	ko vs wt	ko	wt	31,7573108	0,67608586	31,7250574	0,50687006	1,02260808	0,03225333	0,02108162	0,95385685	0,0205168	Unpaired t-t	0,962904311	permutation FDR (250 perm	1
1146	Fabp5~Q058	Fabp5	ko vs wt	ko	wt	28,7923743	1,53122721	27,2679451	1,80863761	2,87672864	1,52442914	0,59671213	0,38518217	0,41433382	Unpaired t-t	0,197744025	permutation FDR (250 perm	1
1147	Fahd2~Q3TC	Fahd2	ko vs wt	ko	wt	24,8027417	0,59016792	23,596228	0,34764961	2,30779284	1,20651372	0,84956532	0,03549159	1,44987457	Unpaired t-t	0,079948768	permutation FDR (250 perm	1
1148	Fah~P35505	Fah	ko vs wt	ko	wt	25,2655538	1,28474407	25,101465	0,82548919	1,12045818	0,1640888	0,08441313	0,86881473	0,06107282	Unpaired t-t	0,852886575	permutation FDR (250 perm	1
1149	Fam114a1~	Fam114a1	ko vs wt	ko	wt	27,9670913	0,4245549	28,0511392	0,45627518	0,94340693	-0,0840479	-0,0598126	0,84563819	0,07281541	Unpaired t-t	0,896499057	permutation FDR (250 perm	1

1150	Fam114a2~	Fam114a2	ko vs wt	ko	wt	26,386115	0,17286004	24,5180328	0,89575614	3,65046997	1,86808221	1,13823501	0,09484517	1,0229848	Unpaired t-te	0,047049914	permutation FDR (250 perm	1
1151	Fam120c~Q8	Fam120c	ko vs wt	ko	wt	27,1621822	0,25176218	27,0133825	0,21979624	1,10864673	0,14879972	0,1226912	0,51777676	0,28585744	Unpaired t-te	0,782157718	permutation FDR (250 perm	1
1152	Fam162a~Q9	Fam162a	ko vs wt	ko	wt	26,3720983	0,46220921	24,6267202	1,17206314	3,35282711	1,74537809	0,93301948	0,16033848	0,79496225	Unpaired t-te	0,079893228	permutation FDR (250 perm	1
1153	Fam171a1~A	Fam171a1	ko vs wt	ko	wt	23,97879	0,40798981	23,5980161	0,36336234	1,30204009	0,38077387	0,28235372	0,32847978	0,48349135	Unpaired t-te	0,513370405	permutation FDR (250 perm	1
1154	Fam180a~Q8	Fam180a	ko vs wt	ko	wt	25,8909246	0,39309058	24,0343974	0,1972559	3,62134886	1,85652717	1,46600379	0,00120935	2,91744732	Unpaired t-te	0,009150527	permutation FDR (250 perm	1
1155	Fam91a1~Q3	Fam91a1	ko vs wt	ko	wt	26,4160307	0,20076521	26,2002201	0,31323569	1,16135627	0,21581062	0,17265019	0,44977085	0,3470087	Unpaired t-te	0,699753299	permutation FDR (250 perm	1
1156	Fam98a~Q3	Fam98a	ko vs wt	ko	wt	28,8041572	0,43896631	28,5984069	0,15331707	1,15328596	0,20575028	0,1612904	0,4969207	0,30371291	Unpaired t-te	0,714828571	permutation FDR (250 perm	1
1157	Fam98b~Q8	Fam98b	ko vs wt	ko	wt	27,3385113	0,31703879	27,5274086	0,23929798	0,87727601	-0,1888973	-0,1512059	0,4832389	0,31583811	Unpaired t-te	0,72933144	permutation FDR (250 perm	1
1158	Fap~P97321	Fap	ko vs wt	ko	wt	24,7679468	0,54146259	24,8746799	0,36711888	0,92868869	-0,106733	-0,075894	0,80328961	0,09512785	Unpaired t-te	0,869011733	permutation FDR (250 perm	1
1159	Far1~Q922J9	Far1	ko vs wt	ko	wt	24,9895999	1,78773057	24,8948412	1,76085689	1,06788681	0,09475873	0,03620487	0,95588354	0,01959502	Unpaired t-te	0,937227176	permutation FDR (250 perm	1
1160	Farp1~F8VPU	Farp1	ko vs wt	ko	wt	29,7257192	0,83736674	28,6871771	1,24289344	2,05415085	1,03854213	0,51847904	0,37233764	0,42906306	Unpaired t-te	0,25955074	permutation FDR (250 perm	1
1161	Farp2~Q91V	Farp2	ko vs wt	ko	wt	25,0809988	1,15135897	24,796481	0,85561095	1,21800306	0,28451776	0,14983778	0,76452619	0,11660763	Unpaired t-te	0,731616345	permutation FDR (250 perm	1
1162	Farsa~Q8C0	Farsa	ko vs wt	ko	wt	30,0870689	0,22838098	29,7598481	0,1953603	1,25459415	0,32722075	0,27475192	0,15099383	0,82104079	Unpaired t-te	0,523145226	permutation FDR (250 perm	1
1163	Farsb~Q9WU	Farsb	ko vs wt	ko	wt	29,8894478	0,22443222	29,9228522	0,244879	0,97711182	-0,0334044	-0,0274646	0,88468434	0,05321166	Unpaired t-te	0,951750989	permutation FDR (250 perm	1
1164	Fasn~P19096	Fasn	ko vs wt	ko	wt	33,721942	0,46624289	34,1083319	0,41074832	0,76504159	-0,3863899	-0,2767831	0,37735498	0,42324992	Unpaired t-te	0,520725707	permutation FDR (250 perm	1
1165	Fat1~F2Z4A3	Fat1	ko vs wt	ko	wt	30,7761514	0,22067236	31,064111	0,33264403	0,81905963	-0,2879596	-0,2271864	0,3568634	0,44749799	Unpaired t-te	0,606675355	permutation FDR (250 perm	1
1166	Fat4~Q2PZL6	Fat4	ko vs wt	ko	wt	25,5347906	0,22889681	26,6380072	0,10768195	0,46547754	-1,1032165	-0,9572213	0,00114709	2,94040163	Unpaired t-te	0,055117453	permutation FDR (250 perm	1
1167	Fau~P62862	Fau	ko vs wt	ko	wt	27,5349918	0,87976521	28,5776145	0,5073297	0,48544417	-1,0426227	-0,6428634	0,15555379	0,80811942	Unpaired t-te	0,163658576	permutation FDR (250 perm	1
1168	Fblim1~Q71	Fblim1	ko vs wt	ko	wt	32,6816615	0,62008223	33,1008292	0,23668468	0,74785597	-0,4191676	-0,3004371	0,34653935	0,46024744	Unpaired t-te	0,489863665	permutation FDR (250 perm	1
1169	Fbln1~Q0887	Fbln1	ko vs wt	ko	wt	27,8766774	0,30642743	26,3147419	0,34546634	2,95249691	1,56193555	1,19999637	0,00694823	2,15812565	Unpaired t-te	0,023835278	permutation FDR (250 perm	1
1170	Fbln1~Q0887	Fbln1	ko vs wt	ko	wt	31,461424	0,28906088	30,5162358	0,11823036	1,92543999	0,94518816	0,79651258	0,00593829	2,22633884	Unpaired t-te	0,10001845	permutation FDR (250 perm	1
1171	Fbln2~P3788	Fbln2	ko vs wt	ko	wt	33,4402531	0,15240149	33,0798329	0,458867	1,28379976	0,36042019	0,26973789	0,38327911	0,41648485	Unpaired t-te	0,553876932	permutation FDR (250 perm	1
1172	Fbln2~P3788	Fbln2	ko vs wt	ko	wt	24,6648743	0,44952555	24,344287	0,34261624	1,24883889	0,32058737	0,23658953	0,40863873	0,38866047	Unpaired t-te	0,580359174	permutation FDR (250 perm	1
1173	Fbln5~Q9W	Fbln5	ko vs wt	ko	wt	34,9660019	0,80974924	35,4263378	0,12708353	0,72681698	-0,460336	-0,3118666	0,40046284	0,39743777	Unpaired t-te	0,481849827	permutation FDR (250 perm	1
1174	Fbln7~Q501F	Fbln7	ko vs wt	ko	wt	28,5885537	0,48201715	28,6227378	0,52066891	0,97658394	-0,034184	-0,0233895	0,94446488	0,02481419	Unpaired t-te	0,95851131	permutation FDR (250 perm	1
1175	Fbl~P35550	Fbl	ko vs wt	ko	wt	30,5225694	0,09505479	30,1104567	0,51080699	1,330633	0,41211272	0,30183878	0,37199149	0,42946699	Unpaired t-te	0,513627663	permutation FDR (250 perm	1
1176	Fbn1~A2AQ5	Fbn1	ko vs wt	ko	wt	37,5893868	0,51283684	38,0732578	0,17165305	0,71505643	-0,483871	-0,3665691	0,20634675	0,68540236	Unpaired t-te	0,405531999	permutation FDR (250 perm	1
1177	Fbn2~Q6155	Fbn2	ko vs wt	ko	wt	29,9109913	0,42108434	29,463593	0,50980633	1,36357903	0,44739831	0,31181823	0,36568883	0,43688831	Unpaired t-te	0,477245592	permutation FDR (250 perm	1
1178	Fbxl22~Q8C7	Fbxl22	ko vs wt	ko	wt	24,9638722	0,93522669	24,2276636	0,35682727	1,66579242	0,73620864	0,46128445	0,28197267	0,54979299	Unpaired t-te	0,300508938	permutation FDR (250 perm	1
1179	Fbxl8~D6RGI	Fbxl8	ko vs wt	ko	wt	23,2080132	1,77931956	24,3023575	0,27357448	0,46834895	-1,0943443	-0,5350412	0,36748576	0,43475948	Unpaired t-te	0,246831625	permutation FDR (250 perm	1
1180	Fbxo2~Q80U	Fbxo2	ko vs wt	ko	wt	25,4445008	0,91474482	23,9588378	0,79512205	2,80045847	1,48566304	0,83870319	0,11620178	0,93478724	Unpaired t-te	0,084259313	permutation FDR (250 perm	1
1181	Fbxo30~Q8B	Fbxo30	ko vs wt	ko	wt	26,4027362	0,30019949	26,533636	0,15342831	0,91326163	-0,1308999	-0,1086779	0,55180917	0,25821109	Unpaired t-te	0,807994448	permutation FDR (250 perm	1
1182	Fbxo6~Q9QZ	Fbxo6	ko vs wt	ko	wt	25,3441355	1,12693435	24,7667389	1,25130124	1,49215416	0,5773966	0,27517701	0,62704952	0,20269816	Unpaired t-te	0,526757055	permutation FDR (250 perm	1
1183	Fbxw8~Q8BI	Fbxw8	ko vs wt	ko	wt	25,6399781	0,49980831	25,1483337	0,74623103	1,40604663	0,49164444	0,30700629	0,47034343	0,32758492	Unpaired t-te	0,489078511	permutation FDR (250 perm	1
1184	Fcgrt~Q6155	Fcgrt	ko vs wt	ko	wt	25,235968	2,1124071	26,9013717	0,29601821	0,31525612	-1,6654037	-0,7443376	0,26638347	0,57449273	Unpaired t-te	0,130222798	permutation FDR (250 perm	1
1185	Fcho2~Q3UC	Fcho2	ko vs wt	ko	wt	26,4741607	0,73985726	26,6968075	0,06971837	0,85699172	-0,2226468	-0,1556979	0,63950791	0,19415408	Unpaired t-te	0,729696109	permutation FDR (250 perm	1
1186	Fcn1~O7016	Fcn1	ko vs wt	ko	wt	26,7818845	0,43572438	24,2058138	0,15293678	5,96313372	2,57607069	2,02231146	0,00070641	3,15094021	Unpaired t-te	0,003531532	permutation FDR (250 perm	1
1187	Fcsk~Q7TMC	Fcsk	ko vs wt	ko	wt	26,1093946	0,27843079	24,615806	1,03472663	2,81588524	1,49358854	0,8539118	0,17293918	0,7621066	Unpaired t-te	0,106259445	permutation FDR (250 perm	1
1188	Fdps~Q920E	Fdps	ko vs wt	ko	wt	25,0840784	0,58201111	24,3182481	0,95044076	1,70034831	0,76583031	0,43727097	0,38313052	0,41665326	Unpaired t-te	0,337121534	permutation FDR (250 perm	1
1189	Fdxr~Q6157	Fdxr	ko vs wt	ko	wt	25,82331	0,19872858	24,3134634	0,19568961	2,84779745	1,50984654	1,27979759	0,00077135	3,11275011	Unpaired t-te	0,017113122	permutation FDR (250 perm	1
1190	Fech~P2231	Fech	ko vs wt	ko	wt	27,4755543	0,49110513	27,5392196	0,20946004	0,95683012	-0,0636653	-0,0482352	0,85116386	0,06998682	Unpaired t-te	0,916400107	permutation FDR (250 perm	1
1191	Fermt2~A6X	Fermt2	ko vs wt	ko	wt	24,2527411	1,19277809	24,6722499	0,4158346	0,7476791	-0,4195089	-0,2398841	0,60524483	0,21806891	Unpaired t-te	0,579758084	permutation FDR (250 perm	1
1192	Fermt2~Q8C	Fermt2	ko vs wt	ko	wt	33,6055765	0,62330562	34,2026768	0,10108096	0,66108133	-0,5971003	-0,4368297	0,19553935	0,70876583	Unpaired t-te	0,334482333	permutation FDR (250 perm	1
1193	Fermt3~Q8K	Fermt3	ko vs wt	ko	wt	29,9984187	2,65749776	28,1490941	0,76319699	3,60331467	1,84932465	0,70411624	0,32370412	0,48985177	Unpaired t-te	0,141905002	permutation FDR (250 perm	1
1194	Fer~P70451-	Fer	ko vs wt	ko	wt	26,7901421	0,33668758	27,0467386	0,5029391	0,83706035	-0,2565965	-0,1825932	0,56921904	0,24472058	Unpaired t-te	0,681600231	permutation FDR (250 perm	1
1195	Fetub~Q9QX	Fetub	ko vs wt	ko	wt	26,2516018	2,04714578	24,952829	0,48097512	2,46019509	1,29877273	0,58244074	0,35865952	0,44531764	Unpaired t-te	0,209772883	permutation FDR (250 perm	1
1196	Fga~E9PV24	Fga	ko vs wt	ko	wt	35,462273	0,69238485	35,3173794	0,78873676	1,1056491	0,14489359	0,08592976	0,84334454	0,07399496	Unpaired t-te	0,852177534	permutation FDR (250 perm	1
1197	Fgb~Q8K0E8	Fgb	ko vs wt	ko	wt	36,0148667	0,71743772	35,4657864	0,46327663	1,46315269	0,54908033	0,35932329	0,34614736	0,46073897	Unpaired t-te	0,40791735	permutation FDR (250 perm	1
1198	Fgd2~Q8BY3	Fgd2	ko vs wt	ko	wt	23,4338367	1,97320221	24,2929372	0,68802597	0,55129618	-0,8591005	-0,383739	0,52620015	0,27884903	Unpaired t-te	0,385472703	permutation FDR (250 perm	1
1199	Fgf23~Q9EP	Fgf23	ko vs wt	ko	wt	25,234319	1,34910478	29,571203	2,04164348	0,04948434	-4,336884	-1,6425223	0,07339969	1,13430574	Unpaired t-te	0,007823227	permutation FDR (250 perm	1
1200	Fgf2~P1565	Fgf2	ko vs wt	ko	wt	29,3151728	0,98947282	30,1600501	0,80643066	0,55675816	-0,8448773	-0,467515	0,34501363	0,46216374	Unpaired t-te	0,28972596	permutation FDR (250 perm	1
1201	Fgg~Q8VCM	Fgg	ko vs wt	ko	wt	35,7336688	0,65619853	35,6019466	0,32817594	1,09560074	0,13172215	0,09120284	0,77954983	0,10815612	Unpaired t-te	0,84056499	permutation FDR (250 perm	1
1202	Fgl1~Q71KU	Fgl1	ko vs wt	ko	wt	24,0109366	0,20945125	24,2555912	0,9177401	0,84401789	-0,2446545	-0,1473724	0,74442026	0,12818181	Unpaired t-te	0,751601351	permutation FDR (250 perm	1
1203	Fgl2~P12804	Fgl2	ko vs wt	ko	wt	28,8400266	0,50892572	28,3836399	0,35630267	1,37210103	0,45638671	0,32903309	0,29151085	0,53534528	Unpaired t-te	0,445710813	permutation FDR (250 perm	1
1204	Fhip2a~Q8C	Fhip2a	ko vs wt	ko	wt	26,4990167	0,23870016	26,2197834	0,08872374	1,21354978	0,27923329	0,24251174	0,13706233	0,86308189	Unpaired t-te	0,575728284	permutation FDR (250 perm	1
1205	Fhl1~A2AEX	Fhl1	ko vs wt	ko	wt	34,0592983	0,5340423	34,5278974	0,65264534	0,72266598	-0,4685991	-0,3013473	0,44970684	0,3470705	Unpaired t-te	0,491695381	permutation FDR (250 perm	1
1206	Fhl1~P97447	Fhl1	ko vs wt	ko	wt	29,7732313	1,19130285	30,7766523	0,72756593	0,49881578	-1,003421	-0,5397871	0,29573058	0,52910376	Unpaired t-te	0,229369168	permutation FDR (250 perm	1
1207	Fhl2~O7043	Fhl2	ko vs wt	ko	wt	29,0478745	0,58283979	29,1848257	0,93461263	0,909439	-0,1369512	-0,0786349	0,86514012	0,06291355	Unpaired t-te	0,868348158	permutation FDR (250 perm	1
1208	Fhl3~Q9R05	Fhl3	ko vs wt	ko	wt	28,7554889	0,20172568	28,7079187	0,05605794	1,03352278	0,04757019	0,0423589	0,72041155	0,14241933	Unpaired t-te	0,927551412	permutation FDR (250 perm	1
1209	Fhod1~Q6P9	Fhod1	ko vs wt	ko	wt	25,2747289	1,40963564	24,7407719	0,39155334	1,44789498	0,53395696	0,28712619	0,57108199	0,24330154	Unpaired t-te	0,512411619	permutation FDR (250 perm	1
1210	Fh~P97807-2	Fh	ko vs wt	ko	wt	29,2372363	0,59410125	29,0194338	0,66434115	1,16296085	0,21780254	0,13770515	0,72729271	0,13829076	Unpaired t-te	0,755821097	permutation FDR (250 perm	1
1211	Fibp~Q9JI19	Fibp	ko vs wt	ko	wt	24,4153303	0,90896251	24,0842143	0,26253168	1,25798606	0,33111594	0,21270979	0,58631822	0,23186661	Unpaired t-te	0,627366686	permutation FDR (250 perm	1
1212	Filip1l~E0CY	Filip1l	ko vs wt	ko	wt	30,9578761	0,92376819	31,9573145	0,13327339	0,50019468	-0,9994384	-0,6483126	0,15678866	0,80468534	Unpaired t-te	0,173866455	permutation FDR (250 perm	1
1213	Filip1~Q9CS	Filip1	ko vs wt	ko	wt	27,867058	0,59351092	28,7753315	0,48974973	0,53282232	-0,9082736	-0,6107349	0,12397503	0,90666578	Unpaired t-te	0,181655551	permutation FDR (250 perm	1

1214	Fis1~Q9CQ9	Fis1	ko vs wt	ko	wt	25,1235318	1,19286655	27,6642776	0,25618411	0,17185387	-2,5407458	-1,4839701	0,03059962	1,51428399	Unpaired t-t	0,010925373	permutation FDR (250 perm	1
1215	Fjx1~Q8BQB	Fjx1	ko vs wt	ko	wt	25,6454499	0,8171654	24,8920742	1,42263114	1,6857327	0,75337579	0,3568654	0,54815041	0,26110026	Unpaired t-t	0,428740453	permutation FDR (250 perm	1
1216	Fkbp10~Q61	Fkbp10	ko vs wt	ko	wt	28,1334416	0,59961467	27,2568532	0,78068033	1,83602842	0,87658839	0,53075244	0,25865482	0,58727942	Unpaired t-t	0,246505124	permutation FDR (250 perm	1
1217	Fkbp11~Q9D	Fkbp11	ko vs wt	ko	wt	24,4516018	1,44783668	24,2739345	0,20717431	1,1310536	0,1776673	0,09610659	0,84686408	0,07218629	Unpaired t-t	0,836224608	permutation FDR (250 perm	1
1218	Fkbp15~Q6P	Fkbp15	ko vs wt	ko	wt	25,4213347	1,72949585	26,2712469	0,11325713	0,55481849	-0,8499122	-0,4245886	0,45780311	0,33932127	Unpaired t-t	0,350829932	permutation FDR (250 perm	1
1219	Fkbp1a~P26	Fkbp1a	ko vs wt	ko	wt	26,8625795	0,23573873	25,5963238	0,74421859	2,40536482	1,26625572	0,82034884	0,1300706	0,88582085	Unpaired t-t	0,116297225	permutation FDR (250 perm	1
1220	Fkbp2~P458	Fkbp2	ko vs wt	ko	wt	24,952259	0,65475758	23,6098941	0,50885682	2,53566642	1,34236496	0,88203757	0,05101773	1,29227888	Unpaired t-t	0,071162602	permutation FDR (250 perm	1
1221	Fkbp3~Q624	Fkbp3	ko vs wt	ko	wt	24,8589086	0,8452795	23,7427051	0,40922312	2,16775774	1,11620354	0,71215509	0,11094758	0,95488216	Unpaired t-t	0,132598018	permutation FDR (250 perm	1
1222	Fkbp4~P304	Fkbp4	ko vs wt	ko	wt	28,2809609	0,1792738	27,3712525	1,23204659	1,87866579	0,90970844	0,48457942	0,40612987	0,39133507	Unpaired t-t	0,311578947	permutation FDR (250 perm	1
1223	Fkbp5~Q643	Fkbp5	ko vs wt	ko	wt	27,458281	0,72203779	27,1247944	0,52557639	1,26005489	0,33348659	0,21398258	0,57661627	0,23911311	Unpaired t-t	0,618326844	permutation FDR (250 perm	1
1224	Fkbp8~O354	Fkbp8	ko vs wt	ko	wt	25,06113	1,34564917	26,9843449	0,64786244	0,2636663	-1,9232149	-1,0111984	0,09078525	1,04198471	Unpaired t-t	0,044306977	permutation FDR (250 perm	1
1225	Fkbp9~Q9Z2	Fkbp9	ko vs wt	ko	wt	28,160941	0,50471087	28,5097854	0,44077293	0,78521281	-0,3488444	-0,2445157	0,45339674	0,3435216	Unpaired t-t	0,569340163	permutation FDR (250 perm	1
1226	Flii~Q9JJ28	Flii	ko vs wt	ko	wt	31,029933	0,38312744	31,1486215	0,23527506	0,92102451	-0,1186885	-0,0929594	0,68600683	0,16367156	Unpaired t-t	0,837314254	permutation FDR (250 perm	1
1227	Flna~B7FAU	Flna	ko vs wt	ko	wt	38,5043238	0,52013305	39,2390884	0,13565648	0,60091605	-0,7347646	-0,5586514	0,0877346	1,05682911	Unpaired t-t	0,225136024	permutation FDR (250 perm	1
1228	Flnb~Q80X9	Flnb	ko vs wt	ko	wt	33,7544528	0,31970749	33,62842	0,2944284	1,09128873	0,12603286	0,09859909	0,67147965	0,17296715	Unpaired t-t	0,826868966	permutation FDR (250 perm	1
1229	Flnc~Q8VHX	Flnc	ko vs wt	ko	wt	34,3341424	0,29210995	34,8010414	0,25751073	0,72351811	-0,466899	-0,3740606	0,12330572	0,90901678	Unpaired t-t	0,392804506	permutation FDR (250 perm	1
1230	Flot1~O0891	Flot1	ko vs wt	ko	wt	29,695007	0,48849647	29,7890386	0,04708479	0,93690089	-0,0940316	-0,0732338	0,76179544	0,11816163	Unpaired t-t	0,877549089	permutation FDR (250 perm	1
1231	Flot2~Q6063	Flot2	ko vs wt	ko	wt	30,0977964	0,32354212	30,2466704	0,49880375	0,90195411	-0,1488741	-0,1064056	0,73308133	0,13484784	Unpaired t-t	0,816911757	permutation FDR (250 perm	1
1232	Fmnl1~Q9JL	Fmnl1	ko vs wt	ko	wt	25,9380203	1,19514774	25,7809914	0,22060296	1,11498853	0,15702887	0,09196791	0,8373697	0,07708276	Unpaired t-t	0,842639209	permutation FDR (250 perm	1
1233	Fmnl3~Q6ZP	Fmnl3	ko vs wt	ko	wt	27,1310275	0,37888413	26,8317744	0,12005866	1,23050722	0,29925312	0,24237996	0,27385997	0,56247144	Unpaired t-t	0,577606488	permutation FDR (250 perm	1
1234	Fmo1~P5028	Fmo1	ko vs wt	ko	wt	29,8863438	0,49441929	30,0088335	0,66107598	0,91860099	-0,1224898	-0,0791422	0,83564352	0,07797895	Unpaired t-t	0,865664847	permutation FDR (250 perm	1
1235	Fmo2~Q8K2	Fmo2	ko vs wt	ko	wt	33,4126477	0,43946097	33,9055211	0,12055853	0,71060834	-0,4928735	-0,3888054	0,1462886	0,83478951	Unpaired t-t	0,382514171	permutation FDR (250 perm	1
1236	Fmo3~P975	Fmo3	ko vs wt	ko	wt	30,1790827	0,68467736	30,4210409	0,20736204	0,84559678	-0,2419582	-0,1701993	0,59849689	0,2229381	Unpaired t-t	0,69967642	permutation FDR (250 perm	1
1237	Fmo5~P978	Fmo5	ko vs wt	ko	wt	29,4819235	0,70930936	29,9454566	0,51737036	0,72520808	-0,4635331	-0,2992218	0,43740478	0,35911647	Unpaired t-t	0,487175439	permutation FDR (250 perm	1
1238	Fmod~P506	Fmod	ko vs wt	ko	wt	32,3847679	0,21145247	31,1424771	0,7517254	2,36573871	1,24229074	0,80386908	0,1381817	0,85954948	Unpaired t-t	0,123299912	permutation FDR (250 perm	1
1239	Fmr1~P3592	Fmr1	ko vs wt	ko	wt	27,1888534	0,4022415	27,4196433	0,45864197	0,85216818	-0,2307899	-0,1649814	0,59467591	0,22571966	Unpaired t-t	0,70815794	permutation FDR (250 perm	1
1240	Fn1~A0A087	Fn1	ko vs wt	ko	wt	38,705596	0,1022449	38,322155	0,26359433	1,30444936	0,38344094	0,32073317	0,16697858	0,77733925	Unpaired t-t	0,481936498	permutation FDR (250 perm	1
1241	Fn1~A0A087	Fn1	ko vs wt	ko	wt	29,3535276	0,62992473	29,6514236	0,7000422	0,81343784	-0,297896	-0,1845419	0,65323164	0,18493279	Unpaired t-t	0,675260075	permutation FDR (250 perm	1
1242	Fn3krp~Q8K	Fn3krp	ko vs wt	ko	wt	27,2880264	0,36652635	27,1406172	0,48297381	1,10757869	0,14740921	0,10515997	0,73484302	0,13380543	Unpaired t-t	0,817803596	permutation FDR (250 perm	1
1243	Fnbp1~Q80T	Fnbp1	ko vs wt	ko	wt	27,3247058	0,90018089	27,8507729	0,48265817	0,69444528	-0,5260671	-0,3243799	0,43759629	0,35892637	Unpaired t-t	0,452006371	permutation FDR (250 perm	1
1244	Fndc1~E9Q0	Fndc1	ko vs wt	ko	wt	28,3874611	0,74935022	27,9179081	0,81441572	1,38468035	0,46955297	0,27295055	0,54971845	0,25985969	Unpaired t-t	0,530057633	permutation FDR (250 perm	1
1245	Fndc3a~Q8B	Fndc3a	ko vs wt	ko	wt	26,6345557	1,1374934	25,0541744	0,73048014	2,99048879	1,58038131	0,8609981	0,11721264	0,93102555	Unpaired t-t	0,076224988	permutation FDR (250 perm	1
1246	Fndc3b~Q6N	Fndc3b	ko vs wt	ko	wt	28,6976294	0,30503229	28,2354554	0,13453592	1,37761619	0,462174	0,38509299	0,07560974	1,12142224	Unpaired t-t	0,380925543	permutation FDR (250 perm	1
1247	Fnta~Q6123	Fnta	ko vs wt	ko	wt	27,2149326	0,96936861	27,0104843	0,24306058	1,15224564	0,20444831	0,12895197	0,74666988	0,12687137	Unpaired t-t	0,774560538	permutation FDR (250 perm	1
1248	Focad~A2AK	Focad	ko vs wt	ko	wt	24,3307975	0,77862578	25,0416048	0,81691521	0,61097814	-0,7108073	-0,410407	0,38471456	0,41486138	Unpaired t-t	0,354002033	permutation FDR (250 perm	1
1249	Foxk1~P4212	Foxk1	ko vs wt	ko	wt	27,0336548	0,21146819	26,4742823	0,50898616	1,47362813	0,5593725	0,40532704	0,25599382	0,59177052	Unpaired t-t	0,380753507	permutation FDR (250 perm	1
1250	Foxp1~P584	Foxp1	ko vs wt	ko	wt	25,5876731	0,32692716	25,6380051	0,20688388	0,96571405	-0,050332	-0,0406295	0,84141025	0,0749922	Unpaired t-t	0,929119276	permutation FDR (250 perm	1
1251	Frg1~P9737	Frg1	ko vs wt	ko	wt	25,1412275	0,97386261	24,1678887	0,47379672	1,96337917	0,97333882	0,58829582	0,20163686	0,69543007	Unpaired t-t	0,196993827	permutation FDR (250 perm	1
1252	Frmd4b~Q92	Frmd4b	ko vs wt	ko	wt	24,7159535	0,46238223	24,5282312	0,64673129	1,13896408	0,18772225	0,1227324	0,74419438	0,12831361	Unpaired t-t	0,787531713	permutation FDR (250 perm	1
1253	Frmd6~Q8C	Frmd6	ko vs wt	ko	wt	25,6528313	0,21256427	24,8559321	0,87748123	1,73736302	0,79689923	0,48814853	0,32682921	0,48567914	Unpaired t-t	0,30539027	permutation FDR (250 perm	1
1254	Frmd8~Q3UI	Frmd8	ko vs wt	ko	wt	25,3019308	0,84914703	26,163561	0,37270434	0,55033034	-0,8616302	-0,5535332	0,18967977	0,72197898	Unpaired t-t	0,221687922	permutation FDR (250 perm	1
1255	Fryl~F8VQ05	Fryl	ko vs wt	ko	wt	27,1868886	0,52546376	26,6990348	0,6156521	1,40235714	0,48785381	0,3187308	0,41225669	0,38483228	Unpaired t-t	0,466413924	permutation FDR (250 perm	1
1256	Fry~E9Q8I9	Fry	ko vs wt	ko	wt	30,1518331	0,27455843	30,5442764	0,04463775	0,7618383	-0,3924433	-0,3378389	0,08726731	1,05914839	Unpaired t-t	0,446949185	permutation FDR (250 perm	1
1257	Frzb~P97401	Frzb	ko vs wt	ko	wt	26,2424408	0,60181347	27,6611183	0,36234292	0,37405504	-1,4186775	-0,990898	0,02211144	1,65538295	Unpaired t-t	0,046907418	permutation FDR (250 perm	1
1258	Fstl5~Q8BF	Fstl5	ko vs wt	ko	wt	24,1000163	0,56668612	23,7477775	0,7571381	1,27654005	0,3522388	0,21643815	0,60894268	0,21542359	Unpaired t-t	0,62257673	permutation FDR (250 perm	1
1259	Fth1~P0952	Fth1	ko vs wt	ko	wt	30,3634644	0,79074323	29,7021816	0,44248385	1,58148825	0,66128284	0,42568312	0,28709773	0,54197025	Unpaired t-t	0,332221078	permutation FDR (250 perm	1
1260	Ftl1~Q9CPX	Ftl1	ko vs wt	ko	wt	30,0313142	1,43604788	28,0028933	1,68719573	4,0795805	2,02842081	0,82697036	0,23857079	0,62238273	Unpaired t-t	0,093336475	permutation FDR (250 perm	1
1261	Fubp1~Q91V	Fubp1	ko vs wt	ko	wt	25,8766081	1,00014876	25,0296598	0,97597476	1,79869225	0,84694837	0,44580194	0,39578615	0,4025394	Unpaired t-t	0,315224192	permutation FDR (250 perm	1
1262	Fubp3~A2AJ	Fubp3	ko vs wt	ko	wt	26,9053607	0,46767849	26,7125854	0,76972522	1,14296034	0,19277534	0,11991693	0,77192255	0,11242627	Unpaired t-t	0,794254464	permutation FDR (250 perm	1
1263	Fundc2~Q9D	Fundc2	ko vs wt	ko	wt	24,3113444	0,72122082	27,6460734	0,82628296	0,09911664	-3,334729	-1,9416558	0,0104044	1,98278296	Unpaired t-t	0,003741325	permutation FDR (250 perm	1
1264	Fus~P56959	Fus	ko vs wt	ko	wt	29,9913014	0,5036791	29,9606007	0,05191634	1,02150817	0,03070075	0,02374184	0,92300087	0,03479789	Unpaired t-t	0,958691215	permutation FDR (250 perm	1
1265	Fut8~Q9WT	Fut8	ko vs wt	ko	wt	23,6831243	0,48975126	25,7615411	1,86472817	0,23677411	-2,0784168	-0,8849828	0,2528552	0,5971281	Unpaired t-t	0,09644879	permutation FDR (250 perm	1
1266	Fxr1~Q6158	Fxr1	ko vs wt	ko	wt	28,3866303	0,21059491	28,7827875	0,20574605	0,75987959	-0,3961573	-0,3330165	0,09925094	1,00326535	Unpaired t-t	0,44353009	permutation FDR (250 perm	1
1267	Fxr1~Q6158	Fxr1	ko vs wt	ko	wt	25,432081	1,51761931	24,1045955	0,90917508	2,50964882	1,3274855	0,63615019	0,27687087	0,55772274	Unpaired t-t	0,167224145	permutation FDR (250 perm	1
1268	Fxr2~Q6P5B	Fxr2	ko vs wt	ko	wt	25,5046917	0,71981629	26,1087405	0,31719765	0,65790502	-0,6040488	-0,4102898	0,2636615	0,57895328	Unpaired t-t	0,352913664	permutation FDR (250 perm	1
1269	Fyco1~Q8VD	Fyco1	ko vs wt	ko	wt	27,2118875	0,33135086	27,1771191	0,34825304	1,02439233	0,03476836	0,02650371	0,91642202	0,03790448	Unpaired t-t	0,952667018	permutation FDR (250 perm	1
1270	Fyn~P39688	Fyn	ko vs wt	ko	wt	24,8921041	0,74801693	24,5785496	0,44851001	1,24276585	0,31355451	0,20416255	0,58407672	0,2335301	Unpaired t-t	0,636133881	permutation FDR (250 perm	1
1271	Fyttd1~Q91Z	Fyttd1	ko vs wt	ko	wt	24,0605949	0,66515893	25,0781174	0,83599625	0,4939639	-1,0175225	-0,5968125	0,22960455	0,63901952	Unpaired t-t	0,199421316	permutation FDR (250 perm	1
1272	Fzd1~O7042	Fzd1	ko vs wt	ko	wt	23,969113	0,72087407	24,4473687	0,14388896	0,71785495	-0,4782357	-0,3347928	0,33791658	0,4711905	Unpaired t-t	0,449853721	permutation FDR (250 perm	1
1273	Fzd2~Q9JIP6	Fzd2	ko vs wt	ko	wt	24,4513884	0,17514769	24,1409227	1,19780056	1,24010792	0,31046568	0,16754866	0,74989448	0,12499984	Unpaired t-t	0,71974822	permutation FDR (250 perm	1
1274	G3bp1~P978	G3bp1	ko vs wt	ko	wt	28,3712517	0,90927456	28,1116667	0,09980516	1,19713435	0,25958507	0,16969757	0,65660563	0,1826954	Unpaired t-t	0,70458436	permutation FDR (250 perm	1
1275	G3bp2~P973	G3bp2	ko vs wt	ko	wt	28,3453268	0,80277975	28,6237597	0,266988	0,82448608	-0,278433	-0,1855651	0,60822072	0,21593879	Unpaired t-t	0,673366193	permutation FDR (250 perm	1
1276	G6pdx~Q006	G6pdx	ko vs wt	ko	wt	29,8806221	0,74909337	28,8558361	0,38189445	2,03465765	1,02478607	0,67872416	0,10404016	0,982799	Unpaired t-t	0,146786517	permutation FDR (250 perm	1
1277	Gaa~P70699	Gaa	ko vs wt	ko	wt	26,2517809	1,81612167	28,0424363	0,59048626	0,28904072	-1,7906553	-0,8412306	0,18996313	0,72133069	Unpaired t-t	0,088327429	permutation FDR (250 perm	1

1278	Gabarap~Q9	Gabarap	ko vs wt	ko	wt	25,480189	1,14747916	26,6001085	0,06875612	0,46011951	-1,1199195	-0,6729157	0,18942729	0,72255747	Unpaired t-t	0,163246964	permutation FDR (250 perm	1
1279	Gak~Q99KY4	Gak	ko vs wt	ko	wt	26,8702808	0,29503832	26,918914	0,46956248	0,96685189	-0,0486332	-0,0354166	0,90443202	0,04362407	Unpaired t-t	0,94021851	permutation FDR (250 perm	1
1280	Galk1~Q9R0	Galk1	ko vs wt	ko	wt	27,1924489	0,22890666	27,0098387	0,40015472	1,13493536	0,18261014	0,1391533	0,60141377	0,22082663	Unpaired t-t	0,759542053	permutation FDR (250 perm	1
1281	Galk2~Q68FI	Galk2	ko vs wt	ko	wt	24,6524126	1,50515864	24,7665456	1,14924871	0,92393735	-0,1141331	-0,052121	0,92736956	0,03274716	Unpaired t-t	0,910270677	permutation FDR (250 perm	1
1282	Galnt1~O08	Galnt1	ko vs wt	ko	wt	25,4661622	1,254436	24,9705708	1,10374613	1,40989864	0,49559145	0,24002646	0,66264289	0,17872046	Unpaired t-t	0,576867528	permutation FDR (250 perm	1
1283	Galnt2~Q6P	Galnt2	ko vs wt	ko	wt	28,4441976	0,91675287	27,1986849	0,76429726	2,37102807	1,24551274	0,70910698	0,1635365	0,7863853	Unpaired t-t	0,13320086	permutation FDR (250 perm	1
1284	Gamt~O359	Gamt	ko vs wt	ko	wt	24,7826582	0,42229734	24,7060237	0,73233694	1,05455514	0,07663454	0,04873833	0,90226461	0,04466608	Unpaired t-t	0,918445574	permutation FDR (250 perm	1
1285	Ganab~Q8B	Ganab	ko vs wt	ko	wt	28,824249	0,61600757	28,6884272	0,31036162	1,09871852	0,13582182	0,09579001	0,75915045	0,11967215	Unpaired t-t	0,832145455	permutation FDR (250 perm	1
1286	Gapdh~P168	Gapdh	ko vs wt	ko	wt	33,4554135	0,39787938	33,0624133	0,24143319	1,31312132	0,39300022	0,30554979	0,22853799	0,64104159	Unpaired t-t	0,478313405	permutation FDR (250 perm	1
1287	Gapvd1~Q6P	Gapvd1	ko vs wt	ko	wt	27,6513394	0,28730589	26,7334838	0,66323262	1,88930502	0,91785563	0,61294694	0,18007317	0,74455099	Unpaired t-t	0,205078514	permutation FDR (250 perm	1
1288	Gar1~Q9CY6	Gar1	ko vs wt	ko	wt	27,2218793	0,55627501	25,2044715	1,64853706	4,04855707	2,01740781	0,91321557	0,22039791	0,65679253	Unpaired t-t	0,086544231	permutation FDR (250 perm	1
1289	Gars1~Q9CZ	Gars1	ko vs wt	ko	wt	27,4861454	0,98991507	26,2969363	1,00460036	2,28027708	1,18920914	0,62205856	0,25838519	0,58773238	Unpaired t-t	0,178365642	permutation FDR (250 perm	1
1290	Gart~Q6473	Gart	ko vs wt	ko	wt	28,2849982	0,64547716	27,2948768	0,31469847	1,98635213	0,9901214	0,69043803	0,07557659	1,12161272	Unpaired t-t	0,141751997	permutation FDR (250 perm	1
1291	Gas6~Q6159	Gas6	ko vs wt	ko	wt	26,8548269	0,68933729	26,5113226	0,7606434	1,26883481	0,34350425	0,20580303	0,63478696	0,197372	Unpaired t-t	0,636680926	permutation FDR (250 perm	1
1292	Gas7~Q6078	Gas7	ko vs wt	ko	wt	26,0026282	0,08199835	25,167099	1,26043817	1,78451147	0,83552917	0,44149008	0,44754669	0,34916165	Unpaired t-t	0,353858353	permutation FDR (250 perm	1
1293	Gask1b~Q3U	Gask1b	ko vs wt	ko	wt	29,6708411	0,37318556	30,0794265	0,37114882	0,75336173	-0,4085853	-0,3050155	0,29087354	0,53629578	Unpaired t-t	0,482530946	permutation FDR (250 perm	1
1294	Gatad2b~Q8	Gatad2b	ko vs wt	ko	wt	25,6665759	0,87535138	24,5846085	0,59050845	2,11692105	1,08196746	0,65353427	0,15977323	0,79649599	Unpaired t-t	0,157395349	permutation FDR (250 perm	1
1295	Gatd1~Q8BF	Gatd1	ko vs wt	ko	wt	27,4719691	0,61465591	25,9062722	0,27195127	2,96020449	1,56569684	1,11546705	0,01460799	1,83540944	Unpaired t-t	0,030726804	permutation FDR (250 perm	1
1296	Gatd3~Q9D1	Gatd3	ko vs wt	ko	wt	26,1797045	0,93628968	26,1485275	1,08705334	1,02184544	0,031177	0,01607302	0,97518517	0,01091291	Unpaired t-t	0,971683687	permutation FDR (250 perm	1
1297	Gbe1~Q9D6	Gbe1	ko vs wt	ko	wt	25,1532421	0,42569572	23,5813081	0,53293211	2,97302998	1,57193401	1,08416393	0,02933716	1,53258194	Unpaired t-t	0,037966061	permutation FDR (250 perm	1
1298	Gbf1~Q6DFZ	Gbf1	ko vs wt	ko	wt	27,0601338	0,72870039	27,0634409	0,10923878	0,99771038	-0,003307	-0,0023162	0,99428842	0,00248762	Unpaired t-t	0,996093652	permutation FDR (250 perm	1
1299	Gbp2~Q9Z0	Gbp2	ko vs wt	ko	wt	27,5185669	0,97975738	26,7365681	0,34329509	1,71951154	0,7819988	0,48404523	0,27275564	0,56422626	Unpaired t-t	0,279561702	permutation FDR (250 perm	1
1300	Gbp9~Q8BT	Gbp9	ko vs wt	ko	wt	24,9210251	1,43833416	24,7327888	0,53472125	1,13936999	0,18823631	0,09842678	0,84638122	0,07243398	Unpaired t-t	0,828550469	permutation FDR (250 perm	1
1301	Gcdh~Q6075	Gcdh	ko vs wt	ko	wt	25,8661371	1,4787902	27,4468821	0,22554912	0,33430922	-1,5807449	-0,8459748	0,16035178	0,79492622	Unpaired t-t	0,092550684	permutation FDR (250 perm	1
1302	Gclm~O0917	Gclm	ko vs wt	ko	wt	25,7780699	2,03465075	24,5924081	1,49757802	2,27467719	1,18566182	0,45928269	0,48751167	0,31201499	Unpaired t-t	0,296690042	permutation FDR (250 perm	1
1303	Gcn1~E9PVA	Gcn1	ko vs wt	ko	wt	30,1600787	0,25749452	29,961709	0,71007402	1,14740107	0,19836977	0,13019425	0,73631413	0,13293686	Unpaired t-t	0,780041399	permutation FDR (250 perm	1
1304	Gc~P21614	Gc	ko vs wt	ko	wt	28,7190559	1,25764793	27,3937041	1,79897953	2,50593986	1,32535179	0,53773026	0,42697249	0,3696001	Unpaired t-t	0,24401323	permutation FDR (250 perm	1
1305	Gdap2~Q9D	Gdap2	ko vs wt	ko	wt	24,1882891	0,80707199	23,8540953	0,86933949	1,26067273	0,3341938	0,18866502	0,68683703	0,1631463	Unpaired t-t	0,666612838	permutation FDR (250 perm	1
1306	Gda~Q9R11	Gda	ko vs wt	ko	wt	24,246721	0,63318958	24,6074014	0,33534444	0,77879721	-0,3606804	-0,2512153	0,44711976	0,34957613	Unpaired t-t	0,558435572	permutation FDR (250 perm	1
1307	Gdi1~P5039	Gdi1	ko vs wt	ko	wt	28,8742266	0,82812524	29,1227268	1,13750605	0,84177107	-0,2485002	-0,1283766	0,80595115	0,09369128	Unpaired t-t	0,776354062	permutation FDR (250 perm	1
1308	Gdi2~Q6159	Gdi2	ko vs wt	ko	wt	31,9193727	0,34940197	31,8261578	0,27369285	1,06674467	0,0932149	0,07284979	0,75266605	0,12339767	Unpaired t-t	0,875001089	permutation FDR (250 perm	1
1309	Gemin5~Q8	Gemin5	ko vs wt	ko	wt	24,3137142	0,69559967	24,7292357	0,44267016	0,7497484	-0,4155216	-0,2753293	0,45164874	0,34519919	Unpaired t-t	0,521100523	permutation FDR (250 perm	1
1310	Get3~O5498	Get3	ko vs wt	ko	wt	27,6368518	0,57048559	27,1611911	0,75442669	1,39055492	0,47566073	0,29236373	0,49616785	0,30437138	Unpaired t-t	0,50644093	permutation FDR (250 perm	1
1311	Gfap~P0399	Gfap	ko vs wt	ko	wt	24,7426846	0,43968093	24,1748785	0,69635905	1,48226777	0,56780609	0,36538746	0,37946782	0,42082505	Unpaired t-t	0,415711864	permutation FDR (250 perm	1
1312	Gfpt1~P478	Gfpt1	ko vs wt	ko	wt	24,3865448	0,84642796	25,3444848	1,06707976	0,51479145	-0,95794	-0,504453	0,35267911	0,45262026	Unpaired t-t	0,266822853	permutation FDR (250 perm	1
1313	Gfpt2~Q9Z2	Gfpt2	ko vs wt	ko	wt	27,304804	0,74503715	27,0526584	0,42762444	1,19097706	0,25214563	0,16525548	0,65217442	0,18563624	Unpaired t-t	0,703912894	permutation FDR (250 perm	1
1314	Gfus~P2359	Gfus	ko vs wt	ko	wt	25,4202963	1,55923213	24,3534137	0,16161832	2,09490175	1,06688258	0,5593246	0,32214424	0,49194963	Unpaired t-t	0,230508197	permutation FDR (250 perm	1
1315	Gga2~Q6P5	Gga2	ko vs wt	ko	wt	24,2224562	0,38260926	25,1170366	1,22341475	0,53790361	-0,8945804	-0,4726121	0,41122794	0,38591738	Unpaired t-t	0,316785838	permutation FDR (250 perm	1
1316	Git1~Q5F25	Git1	ko vs wt	ko	wt	25,6075547	1,00314159	26,9990325	0,16634417	0,38117414	-1,3914778	-0,8746001	0,09333756	1,02994357	Unpaired t-t	0,083798547	permutation FDR (250 perm	1
1317	Git2~E9PVA	Git2	ko vs wt	ko	wt	28,3575179	0,5288512	28,0609725	0,41113451	1,22819984	0,29654532	0,2086002	0,51391945	0,28910495	Unpaired t-t	0,628303619	permutation FDR (250 perm	1
1318	Git2~Q9JLQ	Git2	ko vs wt	ko	wt	24,3806393	0,89681649	24,617926	0,81486353	0,84833931	-0,2372867	-0,1337084	0,77286937	0,1118939	Unpaired t-t	0,761955556	permutation FDR (250 perm	1
1319	Gja1~P2324	Gja1	ko vs wt	ko	wt	24,5099852	0,9137317	24,9751405	1,49706908	0,7243931	-0,4651553	-0,2131047	0,72044593	0,14239861	Unpaired t-t	0,63274662	permutation FDR (250 perm	1
1320	Gk~B1ASZ3	Gk	ko vs wt	ko	wt	27,464487	0,47262401	27,5633008	0,38280317	0,93380042	-0,0988139	-0,0713791	0,80773039	0,09273358	Unpaired t-t	0,877181398	permutation FDR (250 perm	1
1321	Gla~P51569	Gla	ko vs wt	ko	wt	25,4392199	1,46245724	24,8196055	0,877986	1,53646453	0,61961446	0,30254241	0,58033351	0,23632235	Unpaired t-t	0,482512096	permutation FDR (250 perm	1
1322	Glb1~P2378	Glb1	ko vs wt	ko	wt	25,873125	0,86053542	24,5588725	0,76439445	2,4867346	1,31425254	0,7578615	0,13834283	0,85904334	Unpaired t-t	0,11304809	permutation FDR (250 perm	1
1323	Glg1~Q6154	Glg1	ko vs wt	ko	wt	29,1844117	0,57331664	29,5356987	0,70910818	0,78388451	-0,351287	-0,2194423	0,59289288	0,22702376	Unpaired t-t	0,615606848	permutation FDR (250 perm	1
1324	Glipr2~Q9CY	Glipr2	ko vs wt	ko	wt	25,8968419	2,28762917	27,7806446	0,03338175	0,27096853	-1,8838028	-0,8116431	0,2490436	0,60372461	Unpaired t-t	0,106394174	permutation FDR (250 perm	1
1325	Glo1~Q9CPU	Glo1	ko vs wt	ko	wt	26,6665291	0,81506585	25,3078513	1,16786735	2,56450042	1,35867781	0,69658846	0,24080309	0,61833795	Unpaired t-t	0,149180274	permutation FDR (250 perm	1
1326	Glod4~Q9CP	Glod4	ko vs wt	ko	wt	27,4566166	0,10737661	26,7270847	1,08695287	1,65810096	0,72953185	0,41191172	0,44285565	0,35373781	Unpaired t-t	0,384257248	permutation FDR (250 perm	1
1327	Glrx3~Q9CQ	Glrx3	ko vs wt	ko	wt	27,466185	0,70985207	26,3368653	1,18015332	2,18755572	1,12931976	0,58523039	0,31225664	0,50548831	Unpaired t-t	0,21393802	permutation FDR (250 perm	1
1328	Gls~D3Z7P3	Gls	ko vs wt	ko	wt	27,4066504	0,50195265	27,5199828	0,45994459	0,92445026	-0,1133324	-0,0789435	0,80613552	0,09359194	Unpaired t-t	0,863771647	permutation FDR (250 perm	1
1329	Gltp~Q9JL62	Gltp	ko vs wt	ko	wt	25,0912984	1,55401499	24,2669205	0,42625742	1,77077127	0,82437788	0,42352115	0,43734566	0,35917518	Unpaired t-t	0,343749062	permutation FDR (250 perm	1
1330	Glud1~P264	Glud1	ko vs wt	ko	wt	31,8353884	0,29803142	31,9363244	0,27890255	0,93242787	-0,100936	-0,0799983	0,71739471	0,14424183	Unpaired t-t	0,862201203	permutation FDR (250 perm	1
1331	Glul~P15105	Glul	ko vs wt	ko	wt	28,5007585	0,6001541	28,2034619	0,29925118	1,22883958	0,29729659	0,21144826	0,49902166	0,3018806	Unpaired t-t	0,624267373	permutation FDR (250 perm	1
1332	Glyr1~D3YYT	Glyr1	ko vs wt	ko	wt	29,2230116	0,45834796	28,7195453	0,15252648	1,4176155	0,50346628	0,39157043	0,15439967	0,81135363	Unpaired t-t	0,377277108	permutation FDR (250 perm	1
1333	Gm20390~E	Gm20390	ko vs wt	ko	wt	31,2990914	0,09348524	30,7617593	0,20912444	1,45128628	0,53733213	0,46425167	0,05402509	1,26740453	Unpaired t-t	0,318322938	permutation FDR (250 perm	1
1334	Gm20441~G	Gm20441	ko vs wt	ko	wt	28,4215423	0,39360124	27,6972304	0,5605225	1,65211251	0,72431194	0,49716873	0,20298539	0,69253522	Unpaired t-t	0,275337843	permutation FDR (250 perm	1
1335	Gm20547~B	Gm20547	ko vs wt	ko	wt	29,5608693	0,82626436	28,4979911	0,71161826	2,08909507	1,06287815	0,62766788	0,18971037	0,72190893	Unpaired t-t	0,173160622	permutation FDR (250 perm	1
1336	Gm49368~Q	Gm49368	ko vs wt	ko	wt	28,1326014	0,87164596	26,9236012	0,59562681	2,31177366	1,20900015	0,72997003	0,12480686	0,90376153	Unpaired t-t	0,122860915	permutation FDR (250 perm	1
1337	Gmds~Q8K0	Gmds	ko vs wt	ko	wt	26,9615526	0,39174771	24,97827	1,0036029	3,95391705	1,9832826	1,13666539	0,09606613	1,01742968	Unpaired t-t	0,041968104	permutation FDR (250 perm	1
1338	Gmfg~Q9ER	Gmfg	ko vs wt	ko	wt	24,2663507	0,53819037	25,078495	1,08357226	0,56953473	-0,8121443	-0,4445698	0,40656721	0,39086765	Unpaired t-t	0,335147361	permutation FDR (250 perm	1
1339	Gmip~Q6PG	Gmip	ko vs wt	ko	wt	24,8814972	0,88743323	24,6346181	0,72953139	1,18663735	0,2468791	0,14294736	0,74857875	0,12576251	Unpaired t-t	0,743979661	permutation FDR (250 perm	1
1340	Gmppa~Q92	Gmppa	ko vs wt	ko	wt	26,9681966	0,80633066	27,5200859	0,0175334	0,68212625	-0,5518893	-0,3765363	0,32120307	0,49322032	Unpaired t-t	0,403183968	permutation FDR (250 perm	1
1341	Gmppb~Q8B	Gmppb	ko vs wt	ko	wt	27,6494111	0,47794741	25,3299607	1,38109921	4,99142053	2,31945046	1,15119418	0,131876	0,87983425	Unpaired t-t	0,041334914	permutation FDR (250 perm	1

1342	Gmpr2~Q99I	Gmpr2	ko vs wt	ko	wt	27,3340459	0,68775625	25,777625	0,16495446	2,9412326	1,55642088	1,1008362	0,0250878	1,6005375	Unpaired t-te	0,036427611	permutation FDR (250 perm	1
1343	Gmps~Q3TH	Gmps	ko vs wt	ko	wt	25,2312278	0,804723	26,9266029	0,24940342	0,30877437	-1,6953751	-1,1325518	0,02914312	1,53546393	Unpaired t-te	0,031347409	permutation FDR (250 perm	1
1344	Gna11~P212	Gna11	ko vs wt	ko	wt	29,4446962	0,50901273	29,5339829	0,01933677	0,93998739	-0,0892867	-0,0689901	0,78126497	0,10720165	Unpaired t-te	0,884925996	permutation FDR (250 perm	1
1345	Gna13~P276	Gna13	ko vs wt	ko	wt	28,6450673	0,71034359	27,9049994	0,25019612	1,67025438	0,74006784	0,51157399	0,17265989	0,76280855	Unpaired t-te	0,257174339	permutation FDR (250 perm	1
1346	Gnai1~B2RS	Gnai1	ko vs wt	ko	wt	27,4838058	0,7831897	27,8974479	0,21950882	0,75072574	-0,4136421	-0,2798523	0,43976588	0,35677847	Unpaired t-te	0,522138249	permutation FDR (250 perm	1
1347	Gnai2~P087	Gnai2	ko vs wt	ko	wt	31,9305055	0,04332481	31,84657	0,08196341	1,05990537	0,08393547	0,07895169	0,28312627	0,54801983	Unpaired t-te	0,869024829	permutation FDR (250 perm	1
1348	Gnao1~P188	Gnao1	ko vs wt	ko	wt	29,0143411	0,11362859	28,4795896	0,22193905	1,44869264	0,53475154	0,45701552	0,05924344	1,22735974	Unpaired t-te	0,322118785	permutation FDR (250 perm	1
1349	Gnaq~P2127	Gnaq	ko vs wt	ko	wt	28,7979437	0,21507897	27,6218473	0,42262285	2,25964547	1,17609643	0,88855078	0,04271405	1,36942922	Unpaired t-te	0,08655	permutation FDR (250 perm	1
1350	Gnas~P6309	Gnas	ko vs wt	ko	wt	29,6755707	0,16856949	29,3764505	0,45889694	1,23039389	0,29912024	0,22342916	0,45778626	0,33933724	Unpaired t-te	0,623316642	permutation FDR (250 perm	1
1351	Gnaz~O7044	Gnaz	ko vs wt	ko	wt	25,3053053	1,43082759	24,3832264	0,76877921	1,8948438	0,92207892	0,46361133	0,39552929	0,40282135	Unpaired t-te	0,293342235	permutation FDR (250 perm	1
1352	Gnb1~P6287	Gnb1	ko vs wt	ko	wt	32,0002248	0,47363819	31,7406133	0,4437106	1,19715627	0,25961149	0,18331636	0,56319441	0,24934167	Unpaired t-te	0,675050159	permutation FDR (250 perm	1
1353	Gnb2~P6288	Gnb2	ko vs wt	ko	wt	30,6580104	0,26678042	30,3030533	0,62365362	1,27894758	0,35495714	0,24194237	0,51266252	0,29016844	Unpaired t-te	0,590808587	permutation FDR (250 perm	1
1354	Gnb4~P2938	Gnb4	ko vs wt	ko	wt	28,6389462	0,7767085	28,3199699	0,67687043	1,24744504	0,31897625	0,19263333	0,64873258	0,18793429	Unpaired t-te	0,656977855	permutation FDR (250 perm	1
1355	Gne~Q91WG	Gne	ko vs wt	ko	wt	24,7809883	0,63985055	23,7860974	0,82061858	1,99292986	0,99489094	0,58943275	0,23001017	0,63825296	Unpaired t-te	0,205009575	permutation FDR (250 perm	1
1356	Gng12~Q9D	Gng12	ko vs wt	ko	wt	26,0582765	1,3161009	25,7392667	0,87668068	1,24747402	0,31900977	0,16106384	0,75812025	0,1202619	Unpaired t-te	0,711147841	permutation FDR (250 perm	1
1357	Gnl1~P3691	Gnl1	ko vs wt	ko	wt	24,8892946	1,45471015	26,3625598	0,13857784	0,36016622	-1,4732652	-0,7982693	0,1773879	0,751076	Unpaired t-te	0,110138149	permutation FDR (250 perm	1
1358	Gnl3l~Q6PG	Gnl3l	ko vs wt	ko	wt	24,5386023	0,22479739	23,9184374	0,8855804	1,53705083	0,62016488	0,37826271	0,42741503	0,36915021	Unpaired t-te	0,416552803	permutation FDR (250 perm	1
1359	Gnpda1~O8	Gnpda1	ko vs wt	ko	wt	26,0224801	0,74778359	24,4439574	0,35125774	2,98663864	1,57852269	1,05369667	0,02818754	1,54994284	Unpaired t-te	0,037716019	permutation FDR (250 perm	1
1360	Gnpnat1~Q9	Gnpnat1	ko vs wt	ko	wt	25,9724771	0,45682399	25,1093078	0,75703875	1,81903	0,86316933	0,54057719	0,24465393	0,6114478	Unpaired t-te	0,246086574	permutation FDR (250 perm	1
1361	Golga2~Q92	Golga2	ko vs wt	ko	wt	27,6270804	0,74566051	27,3756828	0,79766562	1,19035977	0,25139767	0,14705424	0,74064799	0,13038815	Unpaired t-te	0,738906931	permutation FDR (250 perm	1
1362	Golga3~E9Q	Golga3	ko vs wt	ko	wt	28,2007687	0,21330293	28,5876501	0,47209712	0,76478102	-0,3868814	-0,2853498	0,36888124	0,43311343	Unpaired t-te	0,526900613	permutation FDR (250 perm	1
1363	Golga4~Q91	Golga4	ko vs wt	ko	wt	24,6088103	0,78398161	25,3157589	2,12273609	0,61261451	-0,7069486	-0,2753169	0,69004548	0,16112228	Unpaired t-te	0,544865753	permutation FDR (250 perm	1
1364	Golgb1~E9Q	Golgb1	ko vs wt	ko	wt	29,4373248	0,67836903	29,4053923	0,3827314	1,02238069	0,0319325	0,02163355	0,94918222	0,02265041	Unpaired t-te	0,961697161	permutation FDR (250 perm	1
1365	Golim4~D3Y	Golim4	ko vs wt	ko	wt	24,4203474	0,87780375	23,9167319	0,87710963	1,41776214	0,50361551	0,27964014	0,56144281	0,25069448	Unpaired t-te	0,51888882	permutation FDR (250 perm	1
1366	Gosr1~O886	Gosr1	ko vs wt	ko	wt	25,1640923	0,83370185	24,4342512	0,49042702	1,65845642	0,7298411	0,45808322	0,27405517	0,562162	Unpaired t-te	0,298248247	permutation FDR (250 perm	1
1367	Got1~P0520	Got1	ko vs wt	ko	wt	23,884286	0,3877362	24,7721112	0,45318307	0,54042817	-0,8878252	-0,6383112	0,08872028	1,05197708	Unpaired t-te	0,173143085	permutation FDR (250 perm	1
1368	Got2~P0520	Got2	ko vs wt	ko	wt	28,8062491	0,24133847	29,5069297	0,35921595	0,61528185	-0,7006807	-0,5432848	0,08912335	1,05000852	Unpaired t-te	0,240751381	permutation FDR (250 perm	1
1369	Gp1bb~P564	Gp1bb	ko vs wt	ko	wt	25,5762214	1,96677223	24,6756498	0,83798748	1,86680538	0,90057153	0,39484488	0,51764414	0,2859687	Unpaired t-te	0,370433613	permutation FDR (250 perm	1
1370	Gp5~Q9QZU	Gp5	ko vs wt	ko	wt	25,1475342	0,73161214	24,6212232	0,74854887	1,44024178	0,52631103	0,31380591	0,47832978	0,32027258	Unpaired t-te	0,470145294	permutation FDR (250 perm	1
1371	Gp9~O8818	Gp9	ko vs wt	ko	wt	25,0275348	1,41570443	24,4961205	0,87339845	1,44534541	0,53141431	0,2624989	0,62619667	0,20328924	Unpaired t-te	0,541101891	permutation FDR (250 perm	1
1372	Gpc6~Q8R3	Gpc6	ko vs wt	ko	wt	24,8341378	1,203404	25,5029301	1,4006853	0,62903303	-0,6687923	-0,3026443	0,61104208	0,21392888	Unpaired t-te	0,48914107	permutation FDR (250 perm	1
1373	Gpd1l~Q3UL	Gpd1l	ko vs wt	ko	wt	27,1910391	0,38797448	27,3252516	0,72854404	0,91116707	-0,1342125	-0,0859372	0,82778469	0,08208261	Unpaired t-te	0,856090859	permutation FDR (250 perm	1
1374	Gpd1~P1370	Gpd1	ko vs wt	ko	wt	31,3892021	0,86077662	31,5093833	0,44591243	0,92007213	-0,1201811	-0,0756543	0,84666264	0,0722896	Unpaired t-te	0,869766503	permutation FDR (250 perm	1
1375	Gpd2~Q6452	Gpd2	ko vs wt	ko	wt	31,8597723	0,11052559	31,9453278	0,02123762	0,94242165	-0,0855554	-0,0802919	0,27481058	0,56096654	Unpaired t-te	0,864010923	permutation FDR (250 perm	1
1376	Gphn~Q8BU	Gphn	ko vs wt	ko	wt	25,4520839	1,61189886	24,8058413	1,24977873	1,56508673	0,64624261	0,28302105	0,6364428	0,19624062	Unpaired t-te	0,511017327	permutation FDR (250 perm	1
1377	Gpi~P06745	Gpi	ko vs wt	ko	wt	30,362004	0,36817564	30,4356925	0,24363193	0,95020554	-0,0736885	-0,0578579	0,79844544	0,09775476	Unpaired t-te	0,898946688	permutation FDR (250 perm	1
1378	Gpld1~Q8VC	Gpld1	ko vs wt	ko	wt	26,3404958	1,85153982	24,1654381	0,49119622	4,51603838	2,17505775	1,02403975	0,13279642	0,87681364	Unpaired t-te	0,047651975	permutation FDR (250 perm	1
1379	Gpnmb~Q99	Gpnmb	ko vs wt	ko	wt	29,2348929	0,18135011	29,8292317	0,82666594	0,66234792	-0,5943389	-0,3728965	0,41681984	0,38005162	Unpaired t-te	0,424668401	permutation FDR (250 perm	1
1380	Gps1~Q99LD	Gps1	ko vs wt	ko	wt	28,8408194	0,37178427	28,5846143	0,3929769	1,194333	0,25620514	0,18962333	0,50491126	0,29678494	Unpaired t-te	0,664573089	permutation FDR (250 perm	1
1381	Gpx1~P1135	Gpx1	ko vs wt	ko	wt	31,3074852	0,23664943	31,1502045	0,37558551	1,11518318	0,1572807	0,12110967	0,63429615	0,19770792	Unpaired t-te	0,791863763	permutation FDR (250 perm	1
1382	Gpx3~P4641	Gpx3	ko vs wt	ko	wt	33,0848013	0,31605368	32,5071296	0,12895185	1,49243867	0,57767165	0,47979868	0,04364639	1,36005166	Unpaired t-te	0,280992215	permutation FDR (250 perm	1
1383	Gpx4~O7032	Gpx4	ko vs wt	ko	wt	30,1357773	0,14203666	30,1671388	0,20407365	0,97849647	-0,0313615	-0,0268972	0,86124921	0,06487116	Unpaired t-te	0,952751317	permutation FDR (250 perm	1
1384	Gpx7~Q99LJ	Gpx7	ko vs wt	ko	wt	29,1383205	0,68010282	28,535284	0,29248875	1,51891005	0,60303644	0,4176743	0,23985394	0,62005314	Unpaired t-te	0,34390184	permutation FDR (250 perm	1
1385	Gpx8~Q9D7E	Gpx8	ko vs wt	ko	wt	27,3607693	0,69044596	27,127403	0,28462183	1,17557475	0,23336627	0,16132572	0,62700757	0,20272721	Unpaired t-te	0,713262075	permutation FDR (250 perm	1
1386	Grb14~Q9JL	Grb14	ko vs wt	ko	wt	24,0695771	0,55434441	25,4783395	0,26672754	0,37663463	-1,4087624	-1,0271771	0,01479864	1,82977811	Unpaired t-te	0,041731202	permutation FDR (250 perm	1
1387	Grb2~Q6063	Grb2	ko vs wt	ko	wt	28,6215366	0,6698168	28,2810571	0,38378494	1,2661774	0,34047955	0,23123535	0,50400657	0,2975638	Unpaired t-te	0,589020286	permutation FDR (250 perm	1
1388	Grem2~O88	Grem2	ko vs wt	ko	wt	24,5635644	0,16878345	24,520176	0,17544514	1,03053131	0,04338834	0,03747629	0,79637798	0,09888075	Unpaired t-te	0,934841518	permutation FDR (250 perm	1
1389	Grhpr~Q91Z	Grhpr	ko vs wt	ko	wt	27,5443546	0,3667043	27,5827228	0,46159698	0,97375567	-0,0383683	-0,027622	0,92666761	0,03307602	Unpaired t-te	0,951647679	permutation FDR (250 perm	1
1390	Grip2~G3XA	Grip2	ko vs wt	ko	wt	25,8015892	0,22225112	25,5093749	0,40143832	1,22451827	0,2922143	0,22280669	0,42134881	0,37535822	Unpaired t-te	0,617524484	permutation FDR (250 perm	1
1391	Grk2~Q99M	Grk2	ko vs wt	ko	wt	27,0225218	0,51174236	27,0123871	0,79609992	1,0070496	0,01013474	0,00619576	0,98825288	0,00513191	Unpaired t-te	0,989114874	permutation FDR (250 perm	1
1392	Grk5~Q8VEB	Grk5	ko vs wt	ko	wt	24,1952836	0,68626738	24,3891044	0,71100683	0,87428726	-0,1938207	-0,1181748	0,77652293	0,10984572	Unpaired t-te	0,792443328	permutation FDR (250 perm	1
1393	Grk6~O7029	Grk6	ko vs wt	ko	wt	25,9032901	0,33842902	26,0142007	0,31732015	0,92600345	-0,1109105	-0,0854782	0,72633239	0,13886459	Unpaired t-te	0,851594521	permutation FDR (250 perm	1
1394	Grn~P28798	Grn	ko vs wt	ko	wt	29,5161622	0,58296409	30,3207659	0,61350348	0,57251931	-0,8046037	-0,5194115	0,21444715	0,66867973	Unpaired t-te	0,249692195	permutation FDR (250 perm	1
1395	Grpel1~Q99	Grpel1	ko vs wt	ko	wt	24,2320052	0,509012	25,1505544	1,85102036	0,52904079	-0,9185491	-0,3922984	0,55845687	0,25301036	Unpaired t-te	0,399755183	permutation FDR (250 perm	1
1396	Grwd1~Q81	Grwd1	ko vs wt	ko	wt	24,4929518	0,76578442	24,4268264	0,61655475	1,04690132	0,06612546	0,04079495	0,91947361	0,03646073	Unpaired t-te	0,928912673	permutation FDR (250 perm	1
1397	Gsk3a~Q2NL	Gsk3a	ko vs wt	ko	wt	27,7768572	0,13598157	27,9023894	0,24552392	0,91666578	-0,1255323	-0,1054417	0,55975474	0,25200222	Unpaired t-te	0,820315091	permutation FDR (250 perm	1
1398	Gsk3b~Q9W	Gsk3b	ko vs wt	ko	wt	28,118933	0,15572969	28,2723845	0,32968277	0,89909691	-0,1534515	-0,1227751	0,5886041	0,23017672	Unpaired t-te	0,79187828	permutation FDR (250 perm	1
1399	Gsn~P13020	Gsn	ko vs wt	ko	wt	30,2714044	0,5990919	29,5094624	0,91514242	1,6957717	0,76194196	0,43947785	0,37242177	0,42896493	Unpaired t-te	0,332944731	permutation FDR (250 perm	1
1400	Gsn~P13020	Gsn	ko vs wt	ko	wt	33,3097689	0,11141038	33,3620534	0,23368462	0,96440798	-0,0522845	-0,0444098	0,78994698	0,10240205	Unpaired t-te	0,926475733	permutation FDR (250 perm	1
1401	Gspt1~Q8R0	Gspt1	ko vs wt	ko	wt	30,0425348	0,30562681	29,9157526	0,27892263	1,09185575	0,12678227	0,10025159	0,65404911	0,18438964	Unpaired t-te	0,823361679	permutation FDR (250 perm	1
1402	Gsr~P47791	Gsr	ko vs wt	ko	wt	24,4901832	0,2176862	24,9197644	0,29234832	0,74247729	-0,4295812	-0,3458984	0,16203924	0,79037979	Unpaired t-te	0,435109823	permutation FDR (250 perm	1
1403	Gstk1~Q9DC	Gstk1	ko vs wt	ko	wt	27,7210009	0,23633351	25,4758641	2,08921859	4,74082054	2,24513678	0,90398902	0,26736435	0,5728965	Unpaired t-te	0,095153558	permutation FDR (250 perm	1
1404	Gstm1~P106	Gstm1	ko vs wt	ko	wt	30,2319471	1,05297568	30,8503679	0,5414197	0,65138354	-0,6184208	-0,3598738	0,4307807	0,36574377	Unpaired t-te	0,408529836	permutation FDR (250 perm	1
1405	Gstm2~P156	Gstm2	ko vs wt	ko	wt	30,7687567	0,57045969	31,3533887	0,39409702	0,6668194	-0,584632	-0,4084254	0,23345506	0,63179671	Unpaired t-te	0,351681741	permutation FDR (250 perm	1

1406	Gstm5~P487	Gstm5	ko vs wt	ko	wt	27,0784331	0,23447504	26,0782703	1,37257464	2,00022559	1,00016272	0,50514482	0,41132929	0,38581037	Unpaired t-te	0,291658228	permutation FDR (250 perm	1
1407	Gsto1~O091	Gsto1	ko vs wt	ko	wt	25,0178725	0,86330297	23,8866846	0,39902316	2,19039013	1,13118785	0,71924286	0,11113744	0,9541396	Unpaired t-te	0,12971725	permutation FDR (250 perm	1
1408	Gstp1~P1915	Gstp1	ko vs wt	ko	wt	30,5930578	0,27871409	30,5275001	0,08344304	1,04648938	0,06555766	0,05596561	0,72272462	0,14102715	Unpaired t-te	0,904137634	permutation FDR (250 perm	1
1409	Gstt1~Q644	Gstt1	ko vs wt	ko	wt	28,1719663	1,90681866	28,7882891	1,44062914	0,65233153	-0,6163227	-0,2465392	0,69841182	0,15588842	Unpaired t-te	0,564963186	permutation FDR (250 perm	1
1410	Gstt2~Q611	Gstt2	ko vs wt	ko	wt	24,8865903	1,21155224	24,5987874	0,51205109	1,22077971	0,28780289	0,16099556	0,73188186	0,13555902	Unpaired t-te	0,713598857	permutation FDR (250 perm	1
1411	Gstt3~Q99L	Gstt3	ko vs wt	ko	wt	27,3579452	0,19955358	27,0910981	0,88130466	1,20317552	0,26684712	0,1633354	0,71229708	0,14733884	Unpaired t-te	0,725622513	permutation FDR (250 perm	1
1412	Gstz1~Q9W	Gstz1	ko vs wt	ko	wt	28,2032898	0,43482284	28,2442205	1,20652552	0,97202766	-0,0409307	-0,0216644	0,96691038	0,01461378	Unpaired t-te	0,963685759	permutation FDR (250 perm	1
1413	Gtf2f2~Q8R	Gtf2f2	ko vs wt	ko	wt	24,657545	0,47953239	24,2741593	0,63276398	1,30439941	0,3833857	0,25120918	0,5121451	0,29060698	Unpaired t-te	0,565870365	permutation FDR (250 perm	1
1414	Gtf2h2~Q9J	Gtf2h2	ko vs wt	ko	wt	23,788451	0,65807392	24,8938519	1,99175101	0,46477331	-1,1054009	-0,4495823	0,51874466	0,28504636	Unpaired t-te	0,338015058	permutation FDR (250 perm	1
1415	Gtf2i~Q9ESZ	Gtf2i	ko vs wt	ko	wt	27,770331	0,39951626	28,1967835	0,32666334	0,7440892	-0,4264525	-0,3215031	0,25053829	0,6011259	Unpaired t-te	0,456456888	permutation FDR (250 perm	1
1416	Gtf3c2~Q8B	Gtf3c2	ko vs wt	ko	wt	25,215418	0,98759457	23,92864	0,56722661	2,43982556	1,286778	0,75820919	0,12536796	0,90181343	Unpaired t-te	0,11136548	permutation FDR (250 perm	1
1417	Gtf3c3~Q3T	Gtf3c3	ko vs wt	ko	wt	24,3032221	0,71215072	24,6846938	1,24242731	0,76765413	-0,3814716	-0,1936422	0,72140959	0,14181809	Unpaired t-te	0,666598489	permutation FDR (250 perm	1
1418	Gtpbp10~Q8	Gtpbp10	ko vs wt	ko	wt	24,40961	0,2400073	24,5659758	0,43610343	0,89728252	-0,1563658	-0,1168588	0,67712129	0,16933353	Unpaired t-te	0,801469342	permutation FDR (250 perm	1
1419	Gtpbp1~O08	Gtpbp1	ko vs wt	ko	wt	28,3860972	0,31153225	28,3952988	0,35853654	0,99364229	-0,0092015	-0,0070195	0,97784922	0,00972811	Unpaired t-te	0,987249609	permutation FDR (250 perm	1
1420	Gtpbp2~Q3U	Gtpbp2	ko vs wt	ko	wt	24,6350944	1,23890678	23,7857199	0,36494991	1,8017196	0,8493745	0,4824866	0,33062209	0,48066814	Unpaired t-te	0,283264498	permutation FDR (250 perm	1
1421	Gtpbp3~E9P	Gtpbp3	ko vs wt	ko	wt	24,0929684	1,07584077	23,3751881	0,61069912	1,64464971	0,71778034	0,40864306	0,3872252	0,41203639	Unpaired t-te	0,352068004	permutation FDR (250 perm	1
1422	Gtpbp4~Q99	Gtpbp4	ko vs wt	ko	wt	26,5336124	0,25684556	26,7574361	0,43729605	0,85629293	-0,2238237	-0,1666677	0,56165001	0,25053423	Unpaired t-te	0,71170489	permutation FDR (250 perm	1
1423	Gucy1a1~Q9	Gucy1a1	ko vs wt	ko	wt	28,8271964	0,46377401	29,0000479	0,31363404	0,88708765	-0,1728514	-0,1282589	0,64016536	0,19370783	Unpaired t-te	0,770766125	permutation FDR (250 perm	1
1424	Gucy1b1~O5	Gucy1b1	ko vs wt	ko	wt	27,541909	0,47996827	26,7334641	0,49769815	1,75132275	0,80844498	0,55834523	0,1423544	0,8466291	Unpaired t-te	0,219753476	permutation FDR (250 perm	1
1425	Gulp1~Q8K2	Gulp1	ko vs wt	ko	wt	28,0365464	0,84463249	29,2531755	0,26563997	0,43028692	-1,2166291	-0,7990607	0,08333201	1,07918816	Unpaired t-te	0,1029375	permutation FDR (250 perm	1
1426	Gusb~P1226	Gusb	ko vs wt	ko	wt	24,8896236	1,29913043	25,2893834	1,12812166	0,76325666	-0,3897598	-0,1860476	0,73738729	0,13230435	Unpaired t-te	0,668666473	permutation FDR (250 perm	1
1427	Gvin1~L7N4	Gvin1	ko vs wt	ko	wt	28,0963534	1,7210926	26,6554182	0,65183057	2,7149681	1,44093525	0,6876769	0,25643868	0,59101647	Unpaired t-te	0,146176667	permutation FDR (250 perm	1
1428	Gxylt2~Q810	Gxylt2	ko vs wt	ko	wt	25,9689016	0,30888171	25,9129457	0,02678078	1,03954767	0,05595591	0,04744698	0,77503758	0,11067724	Unpaired t-te	0,920650267	permutation FDR (250 perm	1
1429	Gyg1~Q9R0	Gyg1	ko vs wt	ko	wt	29,2814257	0,69519396	28,8818058	0,20423464	1,31916034	0,39961993	0,28012822	0,40539311	0,39212364	Unpaired t-te	0,521218193	permutation FDR (250 perm	1
1430	Gys1~Q9Z1	Gys1	ko vs wt	ko	wt	26,5801784	0,14173909	26,6178488	0,09337522	0,97422675	-0,0376705	-0,0340864	0,73477864	0,13384348	Unpaired t-te	0,940566278	permutation FDR (250 perm	1
1431	H1-0~P1092	H1-0	ko vs wt	ko	wt	31,8534367	1,08863156	32,7134051	0,76493626	0,55096463	-0,8599684	-0,4701288	0,34734686	0,45923662	Unpaired t-te	0,285755822	permutation FDR (250 perm	1
1432	H1-1~P4327	H1-1	ko vs wt	ko	wt	28,3323849	1,23621402	29,8522965	1,20334837	0,34870729	-1,5199116	-0,7201325	0,2373683	0,62457727	Unpaired t-te	0,130417962	permutation FDR (250 perm	1
1433	H1-2~P1586	H1-2	ko vs wt	ko	wt	33,5922698	0,6801942	34,5217097	0,86114229	0,52506213	-0,9294399	-0,5389395	0,2760332	0,55903869	Unpaired t-te	0,2396369	permutation FDR (250 perm	1
1434	H1-3~P4327	H1-3	ko vs wt	ko	wt	28,0954198	0,62206594	30,1206035	1,25478846	0,24567387	-2,0251837	-1,0347341	0,13612453	0,86606361	Unpaired t-te	0,05477735	permutation FDR (250 perm	1
1435	H1-4~P4327	H1-4	ko vs wt	ko	wt	30,5471233	0,20904953	31,0168058	1,24282271	0,72212352	-0,4696825	-0,2488967	0,64766481	0,1886497	Unpaired t-te	0,587819701	permutation FDR (250 perm	1
1436	H1-5~P4327	H1-5	ko vs wt	ko	wt	29,6533059	0,7712588	30,7712546	0,95324345	0,46074846	-1,1179488	-0,6183869	0,24476096	0,61125786	Unpaired t-te	0,185174355	permutation FDR (250 perm	1
1437	H2-Aa~P144	H2-Aa	ko vs wt	ko	wt	24,6981759	1,00104883	24,2866259	0,41802809	1,33011406	0,41154997	0,2495515	0,55805177	0,25332551	Unpaired t-te	0,563064935	permutation FDR (250 perm	1
1438	H2-Ab1~P14	H2-Ab1	ko vs wt	ko	wt	25,9382729	1,14218511	24,8100949	0,38159677	2,1858252	1,12817804	0,6587863	0,1898756	0,72153084	Unpaired t-te	0,161545085	permutation FDR (250 perm	1
1439	H2-D1~P018	H2-D1	ko vs wt	ko	wt	29,3321303	1,01383478	28,5323073	0,3216153	1,74088758	0,79982305	0,49130272	0,27436291	0,56167461	Unpaired t-te	0,274523318	permutation FDR (250 perm	1
1440	H2-K1~P019	H2-K1	ko vs wt	ko	wt	28,4504392	0,84192055	27,0789973	0,73114998	2,58729022	1,37144189	0,80218897	0,11521766	0,93848095	Unpaired t-te	0,095960729	permutation FDR (250 perm	1
1441	H2-Q10~P01	H2-Q10	ko vs wt	ko	wt	29,6277046	1,244079	27,5395753	0,09558959	4,25196367	2,08812927	1,21301121	0,061476	1,21129441	Unpaired t-te	0,027316035	permutation FDR (250 perm	1
1442	H2-Q4~Q8H	H2-Q4	ko vs wt	ko	wt	25,5705591	0,49554512	23,6276733	0,36077331	3,84473924	1,94288575	1,40450998	0,00395599	2,4027445	Unpaired t-te	0,01055036	permutation FDR (250 perm	1
1443	H2az2~Q3TH	H2az2	ko vs wt	ko	wt	30,6779093	0,37371614	30,9062525	0,15327862	0,85361458	-0,2283433	-0,1839316	0,39441486	0,40404673	Unpaired t-te	0,674658162	permutation FDR (250 perm	1
1444	H2bc7~P108	H2bc7	ko vs wt	ko	wt	24,203507	0,6143374	25,0509698	1,7721082	0,5557613	-0,8474627	-0,3680938	0,57370797	0,24130911	Unpaired t-te	0,425840156	permutation FDR (250 perm	1
1445	H2bc8~Q6ZV	H2bc8	ko vs wt	ko	wt	33,0673204	0,36419979	33,3248916	0,48228699	0,83649502	-0,2575711	-0,183895	0,56022776	0,25163538	Unpaired t-te	0,678391204	permutation FDR (250 perm	1
1446	H3-3b~P842	H3-3b	ko vs wt	ko	wt	34,3807831	0,24718539	34,5620179	0,09810256	0,88194781	-0,1812348	-0,1564151	0,31388101	0,50323496	Unpaired t-te	0,721706318	permutation FDR (250 perm	1
1447	H3c7~P8422	H3c7	ko vs wt	ko	wt	27,1910612	1,0614275	28,0417372	0,94732726	0,55452487	-0,8506759	-0,4458735	0,39530043	0,40307271	Unpaired t-te	0,31351009	permutation FDR (250 perm	1
1448	H3c8~P6843	H3c8	ko vs wt	ko	wt	24,0089234	0,35382891	25,6117452	0,4459051	0,32923241	-1,6028217	-1,165099	0,01617421	1,79117705	Unpaired t-te	0,028787958	permutation FDR (250 perm	1
1449	H6pd~Q8CF	H6pd	ko vs wt	ko	wt	26,8921941	0,90982037	25,9889739	0,36141532	1,87023591	0,90322026	0,57015969	0,19308435	0,71425293	Unpaired t-te	0,211517817	permutation FDR (250 perm	1
1450	Habp2~Q3V1	Habp2	ko vs wt	ko	wt	25,1016211	1,43146013	24,3193628	0,33256766	1,7198209	0,78225833	0,42073653	0,42135111	0,37535586	Unpaired t-te	0,349137415	permutation FDR (250 perm	1
1451	Hacd3~Q8K2	Hacd3	ko vs wt	ko	wt	30,2422386	1,0158122	29,9858103	0,6535778	1,19451774	0,25642828	0,14680845	0,74526631	0,12768851	Unpaired t-te	0,73657774	permutation FDR (250 perm	1
1452	Hace1~Q3U	Hace1	ko vs wt	ko	wt	24,7723327	0,38635152	23,499245	0,05811964	2,41678252	1,27308766	1,03771821	0,00937019	2,02825167	Unpaired t-te	0,048305413	permutation FDR (250 perm	1
1453	Hadha~Q8B	Hadha	ko vs wt	ko	wt	34,3191684	0,27742354	34,4938242	0,18220534	0,8859789	-0,1746557	-0,1448755	0,43430504	0,36220513	Unpaired t-te	0,73958609	permutation FDR (250 perm	1
1454	Hadhb~Q99J	Hadhb	ko vs wt	ko	wt	33,280153	0,28858953	33,4118937	0,39284279	0,91272958	-0,1317406	-0,099508	0,70846551	0,14968128	Unpaired t-te	0,828809708	permutation FDR (250 perm	1
1455	Hadh~Q6142	Hadh	ko vs wt	ko	wt	31,2052232	0,31794294	30,8105804	0,26139867	1,31461719	0,39464276	0,31308416	0,19276996	0,71496065	Unpaired t-te	0,467834334	permutation FDR (250 perm	1
1456	Hapln1~Q9Q	Hapln1	ko vs wt	ko	wt	32,7746174	0,79598515	32,3398454	0,67796855	1,35169719	0,43477199	0,2612668	0,54326835	0,26498559	Unpaired t-te	0,544209564	permutation FDR (250 perm	1
1457	Hars1~Q610	Hars1	ko vs wt	ko	wt	28,3303054	0,34534854	28,0814098	0,28624732	1,1882971	0,24889558	0,19382585	0,42292844	0,37373311	Unpaired t-te	0,654012241	permutation FDR (250 perm	1
1458	Haus5~Q9D7	Haus5	ko vs wt	ko	wt	24,5692226	0,56970414	25,0113475	0,20355012	0,73604967	-0,442125	-0,3253235	0,28503762	0,54509782	Unpaired t-te	0,455961808	permutation FDR (250 perm	1
1459	Hba-a1~Q91	Hba-a1	ko vs wt	ko	wt	36,4995343	0,44993724	36,1477706	0,57097606	1,27611975	0,35176371	0,23766347	0,50870331	0,29353544	Unpaired t-te	0,586182796	permutation FDR (250 perm	1
1460	Hbb-bs~A8D	Hbb-bs	ko vs wt	ko	wt	36,5935241	0,40786526	35,9913541	0,34795115	1,51799818	0,60217006	0,44919034	0,14089531	0,85110348	Unpaired t-te	0,309297976	permutation FDR (250 perm	1
1461	Hbq1b~Q3U	Hbq1b	ko vs wt	ko	wt	24,7269346	0,9496178	24,0432869	0,66182835	1,60619575	0,68364773	0,39727729	0,38654932	0,41279508	Unpaired t-te	0,364031039	permutation FDR (250 perm	1
1462	Hbs1l~Q69Z	Hbs1l	ko vs wt	ko	wt	26,7325964	0,32365945	26,7530679	0,34219469	0,98591041	-0,0204715	-0,0156783	0,949737	0,02239664	Unpaired t-te	0,972336214	permutation FDR (250 perm	1
1463	Hcfc1~Q611	Hcfc1	ko vs wt	ko	wt	28,5741989	0,06924653	28,7420311	0,33494718	0,89017924	-0,1678322	-0,1353274	0,5536015	0,25680194	Unpaired t-te	0,773802691	permutation FDR (250 perm	1
1464	Hcls1~P4971	Hcls1	ko vs wt	ko	wt	24,8510482	1,99389785	23,5087571	0,33891718	2,5355365	1,34229105	0,61690196	0,33058045	0,48072283	Unpaired t-te	0,190792072	permutation FDR (250 perm	1
1465	Hdac1~O091	Hdac1	ko vs wt	ko	wt	28,5895859	0,37541649	28,1997531	0,3317973	1,31024152	0,38983277	0,29545951	0,28083613	0,55154702	Unpaired t-te	0,493737449	permutation FDR (250 perm	1
1466	Hdac6~Q9Z2	Hdac6	ko vs wt	ko	wt	24,9567223	1,69405071	24,3048116	0,59431559	1,57124777	0,6519107	0,31576827	0,57333688	0,24159012	Unpaired t-te	0,469049305	permutation FDR (250 perm	1
1467	Hdgfl2~Q3U	Hdgfl2	ko vs wt	ko	wt	25,8536764	1,91214564	25,8069445	1,9091761	1,03292237	0,04673184	0,01703107	0,97982226	0,0088527	Unpaired t-te	0,970504457	permutation FDR (250 perm	1
1468	Hdgfl3~Q9J	Hdgfl3	ko vs wt	ko	wt	24,4428419	1,90897931	24,8220598	0,52404643	0,76885426	-0,3792179	-0,1753386	0,76217047	0,11794788	Unpaired t-te	0,691239793	permutation FDR (250 perm	1
1469	Hdgf~P5185	Hdgf	ko vs wt	ko	wt	27,2731421	0,5597638	27,1527404	0,42286424	1,08703749	0,1204017	0,08359566	0,79559853	0,09930603	Unpaired t-te	0,854504519	permutation FDR (250 perm	1

1470	Hdhd2~Q3U(	Hdhd2	ko vs wt	ko	wt	24,6937247	0,90493747	24,9757603	0,98353185	0,82242981	-0,2820355	-0,1508333	0,76186438	0,11812233	Unpaired t-t	0,733346198	permutation FDR (250 perm	1
1471	Hdhd5~Q91V	Hdhd5	ko vs wt	ko	wt	24,9396183	1,13816303	24,8790828	0,82897399	1,04285274	0,06053544	0,03218994	0,94787648	0,02324825	Unpaired t-t	0,94376589	permutation FDR (250 perm	1
1472	Hdlbp~Q8VD	Hdlbp	ko vs wt	ko	wt	31,9324611	0,48895531	31,5962122	0,5042574	1,26246978	0,33624885	0,23113288	0,49893762	0,30195375	Unpaired t-t	0,592134604	permutation FDR (250 perm	1
1473	Heatr1~G3X!	Heatr1	ko vs wt	ko	wt	24,8269961	0,51567927	23,8605258	0,81198615	1,95405396	0,96647031	0,58689093	0,22988398	0,63849129	Unpaired t-t	0,211763547	permutation FDR (250 perm	1
1474	Heatr5a~Q5I	Heatr5a	ko vs wt	ko	wt	24,0293663	0,24922425	24,7415563	0,75286543	0,61039286	-0,71219	-0,4590452	0,31128121	0,5068471	Unpaired t-t	0,328624914	permutation FDR (250 perm	1
1475	Heatr5b~Q8(	Heatr5b	ko vs wt	ko	wt	26,7163205	0,78555169	24,9548651	1,04552655	3,39039982	1,76145542	0,943302	0,12295616	0,91024973	Unpaired t-t	0,064426555	permutation FDR (250 perm	1
1476	Heca~Q3V1N	Heca	ko vs wt	ko	wt	24,068526	0,81177016	24,1632678	0,68677266	0,93643987	-0,0947417	-0,0565657	0,89414415	0,04859246	Unpaired t-t	0,901903122	permutation FDR (250 perm	1
1477	Hectd1~Q69.	Hectd1	ko vs wt	ko	wt	26,8402888	0,38354205	24,6283842	1,01229461	4,63286473	2,21190456	1,26447317	0,07939925	1,1001836	Unpaired t-t	0,028834532	permutation FDR (250 perm	1
1478	Hectd4~E9Q.	Hectd4	ko vs wt	ko	wt	24,8550168	0,9281461	23,7649535	0,56946279	2,1288338	1,09006332	0,65261717	0,16519806	0,78199505	Unpaired t-t	0,158212072	permutation FDR (250 perm	1
1479	Herc4~Q6PA'	Herc4	ko vs wt	ko	wt	24,6228678	1,07273026	25,2592888	0,90876037	0,64330688	-0,636421	-0,3362908	0,50944525	0,29290248	Unpaired t-t	0,437352185	permutation FDR (250 perm	1
1480	Hexa~P2941(	Hexa	ko vs wt	ko	wt	28,0567812	0,27951159	27,2283683	0,69790535	1,77573077	0,82841286	0,54529216	0,22938947	0,63942653	Unpaired t-t	0,251688047	permutation FDR (250 perm	1
1481	Hexb~P2006(	Hexb	ko vs wt	ko	wt	28,6346532	0,84817168	27,5408156	1,19839554	2,13441049	1,09383766	0,55280327	0,33816652	0,47086939	Unpaired t-t	0,232397026	permutation FDR (250 perm	1
1482	Hgf~Q08048	Hgf	ko vs wt	ko	wt	24,777784	1,1704751	25,2909469	2,28947489	0,70068458	-0,5131629	-0,1863143	0,79088268	0,10188793	Unpaired t-t	0,680019642	permutation FDR (250 perm	1
1483	Hgsnat~Q3U	Hgsnat	ko vs wt	ko	wt	23,7400024	1,57330874	24,2663597	0,20994798	0,69430563	-0,5263572	-0,274087	0,60557809	0,21782985	Unpaired t-t	0,535546455	permutation FDR (250 perm	1
1484	Hgs~Q99LI8	Hgs	ko vs wt	ko	wt	27,9057975	0,72870466	27,3339125	0,13627661	1,48646446	0,57188497	0,39946918	0,26923519	0,56986818	Unpaired t-t	0,374425218	permutation FDR (250 perm	1
1485	Hibadh~Q99I	Hibadh	ko vs wt	ko	wt	26,8993784	1,07305645	26,7191897	0,92241343	1,13303204	0,18018866	0,09485722	0,84963445	0,07076788	Unpaired t-t	0,833981823	permutation FDR (250 perm	1
1486	Hibch~Q8QZ	Hibch	ko vs wt	ko	wt	26,5956372	1,2712339	26,1879538	0,80499699	1,32655401	0,40768341	0,21136522	0,67907445	0,16808261	Unpaired t-t	0,622973785	permutation FDR (250 perm	1
1487	Hint1~P7034	Hint1	ko vs wt	ko	wt	24,5633982	0,94924284	26,631233	0,31273501	0,23851719	-2,0678348	-1,2997252	0,02595101	1,58584572	Unpaired t-t	0,017212942	permutation FDR (250 perm	1
1488	Hint2~Q9D0!	Hint2	ko vs wt	ko	wt	24,6575384	1,13177962	23,8382964	0,92332171	1,76447868	0,819242	0,42586608	0,41733317	0,37951709	Unpaired t-t	0,332420185	permutation FDR (250 perm	1
1489	Hint3~Q9CPS	Hint3	ko vs wt	ko	wt	25,3068576	0,41531996	26,2045963	0,80357745	0,53672735	-0,8977387	-0,5552779	0,25042412	0,60132385	Unpaired t-t	0,239087823	permutation FDR (250 perm	1
1490	Hip1r~Q9JKY	Hip1r	ko vs wt	ko	wt	26,1746419	0,49897747	26,5165091	0,33660315	0,78901944	-0,3418673	-0,248868	0,4022434	0,39551107	Unpaired t-t	0,561113327	permutation FDR (250 perm	1
1491	Hip1~Q8VD7	Hip1	ko vs wt	ko	wt	30,5623392	0,37102568	30,872407	0,19835649	0,80660386	-0,3100678	-0,2468603	0,28212586	0,5495571	Unpaired t-t	0,564987326	permutation FDR (250 perm	1
1492	Hist1h2ap~P.	Hist1h2ap	ko vs wt	ko	wt	28,5810314	1,1162664	24,2976296	0,76241978	19,4729798	4,28340176	2,32764047	0,00378304	2,42215888	Unpaired t-t	0,002222222	permutation FDR (250 perm	1
1493	Hist1h4m~P(	Hist1h4m	ko vs wt	ko	wt	34,3876793	0,18438503	34,2516272	0,25559473	1,09889385	0,13605203	0,11246251	0,55821831	0,25319593	Unpaired t-t	0,80566537	permutation FDR (250 perm	1
1494	Hist2h2aa2~	Hist2h2aa2	ko vs wt	ko	wt	34,2357186	0,20883124	34,0615088	0,32420214	1,12834619	0,17420977	0,13836956	0,54793625	0,26126996	Unpaired t-t	0,759427286	permutation FDR (250 perm	1
1495	Hk1~P17710	Hk1	ko vs wt	ko	wt	30,7021883	0,82746011	30,1869372	0,41894639	1,42924288	0,51525111	0,32983919	0,40386891	0,39375958	Unpaired t-t	0,446163788	permutation FDR (250 perm	1
1496	Hk2~O08528	Hk2	ko vs wt	ko	wt	28,1624793	0,55840685	28,5863141	0,30677434	0,74544052	-0,4238348	-0,3052289	0,32666697	0,48589477	Unpaired t-t	0,479436301	permutation FDR (250 perm	1
1497	Hk3~Q3TRM	Hk3	ko vs wt	ko	wt	27,6635882	1,11046943	26,1243409	0,41087865	2,90642835	1,53924734	0,90337363	0,09238047	1,03441981	Unpaired t-t	0,069767109	permutation FDR (250 perm	1
1498	Hmcn2~A2A.	Hmcn2	ko vs wt	ko	wt	25,7727969	0,1206292	25,8269308	0,45005973	0,96317254	-0,0541338	-0,0408319	0,88209847	0,05448293	Unpaired t-t	0,932612277	permutation FDR (250 perm	1
1499	Hmgb1~P631	Hmgb1	ko vs wt	ko	wt	30,9385251	1,01866693	31,7836632	0,73968836	0,55665754	-0,845138	-0,4729209	0,33231604	0,4784487	Unpaired t-t	0,283340644	permutation FDR (250 perm	1
1500	Hmgb2~P30(	Hmgb2	ko vs wt	ko	wt	23,7514831	0,64126581	27,3183947	0,13270927	0,08438254	-3,5669117	-2,581086	0,00158137	2,80096535	Unpaired t-t	0,002475645	permutation FDR (250 perm	1
1501	Hmgcl~P380	Hmgcl	ko vs wt	ko	wt	27,5252416	1,07385287	25,0232438	1,41073156	5,66469313	2,5019978	1,15060423	0,11009163	0,9582457	Unpaired t-t	0,030973042	permutation FDR (250 perm	1
1502	Hmgcs2~P54	Hmgcs2	ko vs wt	ko	wt	24,5729454	0,61650879	25,2299175	1,07627382	0,63420799	-0,656972	-0,357018	0,49346495	0,30674369	Unpaired t-t	0,42858123	permutation FDR (250 perm	1
1503	Hmox1~P14!	Hmox1	ko vs wt	ko	wt	24,6807053	1,93388644	24,7382531	0,07706288	0,96089602	-0,0575478	-0,0271726	0,96216453	0,01675066	Unpaired t-t	0,952468915	permutation FDR (250 perm	1
1504	Hmox2~O70.	Hmox2	ko vs wt	ko	wt	27,9644527	0,87741957	27,5678471	0,34100528	1,31640704	0,39660564	0,25406528	0,51695218	0,28654963	Unpaired t-t	0,557157385	permutation FDR (250 perm	1
1505	Hnrnpa0~Q9	Hnrnpa0	ko vs wt	ko	wt	30,3363416	0,28978648	29,9808609	0,31221683	1,27941188	0,35548070	0,27837072	0,26768713	0,57237251	Unpaired t-t	0,521455439	permutation FDR (250 perm	1
1506	Hnrnpa1~P4!	Hnrnpa1	ko vs wt	ko	wt	27,6304958	0,31007481	25,9287984	1,0956015	3,25283432	1,70169734	0,947956	0,15320581	0,81472475	Unpaired t-t	0,079954635	permutation FDR (250 perm	1
1507	Hnrnpa2b1~(	Hnrnpa2b1	ko vs wt	ko	wt	30,5582975	0,41670384	30,6649488	0,18530126	0,92874128	-0,1066513	-0,083717	0,71494882	0,14572505	Unpaired t-t	0,855279102	permutation FDR (250 perm	1
1508	Hnrnpab~Q9	Hnrnpab	ko vs wt	ko	wt	28,2313261	0,17120285	28,0034509	0,22260188	1,17110884	0,22787516	0,19215938	0,29577223	0,5290426	Unpaired t-t	0,663879035	permutation FDR (250 perm	1
1509	Hnrnpc~Q9Z.	Hnrnpc	ko vs wt	ko	wt	30,0786384	0,7022414	29,7201014	0,16039052	1,28212509	0,35853703	0,25231264	0,44938546	0,34738098	Unpaired t-t	0,564880435	permutation FDR (250 perm	1
1510	Hnrnpdl~Q9Z	Hnrnpdl	ko vs wt	ko	wt	28,2115318	0,29910867	28,6942825	0,55871428	0,71561192	-0,4827507	-0,3373132	0,35047924	0,4553377	Unpaired t-t	0,452996178	permutation FDR (250 perm	1
1511	Hnrnpd~Q60	Hnrnpd	ko vs wt	ko	wt	29,7363976	0,45138933	29,6757066	0,128488	1,04296521	0,06069103	0,04756377	0,83756587	0,07698103	Unpaired t-t	0,919164482	permutation FDR (250 perm	1
1512	Hnrnpf~Q9Z.	Hnrnpf	ko vs wt	ko	wt	28,0448675	1,12082863	28,3825014	0,45996217	0,79133806	-0,3376339	-0,1958152	0,66374818	0,17799666	Unpaired t-t	0,652642753	permutation FDR (250 perm	1
1513	Hnrnph1~O3	Hnrnph1	ko vs wt	ko	wt	27,5825653	0,56158848	26,8554731	0,81585825	1,65529943	0,72709221	0,43754082	0,34661341	0,46015464	Unpaired t-t	0,333589568	permutation FDR (250 perm	1
1514	Hnrnph2~P7(	Hnrnph2	ko vs wt	ko	wt	29,3125197	0,30822341	29,3259229	0,60112893	0,99075263	-0,0134032	-0,0091752	0,97880483	0,0093039	Unpaired t-t	0,985394256	permutation FDR (250 perm	1
1515	Hnrnpk~B2M	Hnrnpk	ko vs wt	ko	wt	31,997544	0,36558844	32,0424311	0,60491901	0,96936571	-0,044887	-0,030391	0,93100456	0,03104819	Unpaired t-t	0,948419662	permutation FDR (250 perm	1
1516	Hnrnpk~P61!	Hnrnpk	ko vs wt	ko	wt	24,010955	0,42523961	24,8187093	1,18665589	0,57127043	-0,8077542	-0,4309695	0,44044496	0,35610836	Unpaired t-t	0,355974255	permutation FDR (250 perm	1
1517	Hnrnpll~Q92	Hnrnpll	ko vs wt	ko	wt	28,7267409	0,49200262	28,7178919	0,65531617	1,00615249	0,00884898	0,00573301	0,98790469	0,00528495	Unpaired t-t	0,989591252	permutation FDR (250 perm	1
1518	Hnrnpl~Q8R(	Hnrnpl	ko vs wt	ko	wt	31,1754285	0,14287788	31,2720519	0,30047939	0,93521931	-0,0966234	-0,0786885	0,70409639	0,15236788	Unpaired t-t	0,870343699	permutation FDR (250 perm	1
1519	Hnrnpm~Q9I	Hnrnpm	ko vs wt	ko	wt	32,6708811	0,50132504	32,2770554	0,05964472	1,31387293	0,39382575	0,30470154	0,26745825	0,572744	Unpaired t-t	0,492496569	permutation FDR (250 perm	1
1520	Hnrnpr~Q8VI	Hnrnpr	ko vs wt	ko	wt	30,3684399	0,22700465	30,5513575	0,16074434	0,8809197	-0,1829176	-0,1558758	0,34013205	0,46835245	Unpaired t-t	0,720249431	permutation FDR (250 perm	1
1521	Hnrnpul1~Q8	Hnrnpul1	ko vs wt	ko	wt	29,9457131	0,22509378	30,1480541	0,07991175	0,86913908	-0,202341	-0,1772261	0,22608482	0,64572859	Unpaired t-t	0,68590553	permutation FDR (250 perm	1
1522	Hnrnpul2~Q(	Hnrnpul2	ko vs wt	ko	wt	30,753327	0,28527801	30,8757052	0,09426979	0,91867201	-0,1223782	-0,1039144	0,52970685	0,27596441	Unpaired t-t	0,81870354	permutation FDR (250 perm	1
1523	Hnrnpu~Q8V	Hnrnpu	ko vs wt	ko	wt	33,1851881	0,1764025	33,5947509	0,35968629	0,75285153	-0,4095627	-0,3214853	0,24335882	0,61375291	Unpaired t-t	0,47631864	permutation FDR (250 perm	1
1524	Hook3~Q8BL	Hook3	ko vs wt	ko	wt	28,3884478	0,11685883	28,1044968	0,51874415	1,2175247	0,28395104	0,20681657	0,5215439	0,28270913	Unpaired t-t	0,653952187	permutation FDR (250 perm	1
1525	Hp1bp3~Q3T	Hp1bp3	ko vs wt	ko	wt	31,7825758	0,44242955	31,4577222	0,24943576	1,25253737	0,32485365	0,24790148	0,3443729	0,46297103	Unpaired t-t	0,563011779	permutation FDR (250 perm	1
1526	Hpcal1~P627	Hpcal1	ko vs wt	ko	wt	27,0228894	1,20096633	26,3418689	0,57379924	1,6032734	0,68102046	0,37763862	0,43848861	0,35804168	Unpaired t-t	0,388057086	permutation FDR (250 perm	1
1527	Hprt1~P0049	Hprt1	ko vs wt	ko	wt	25,0199407	0,79321233	23,9003643	0,34175727	2,17283173	1,11957645	0,73762602	0,09053977	1,0431606	Unpaired t-t	0,122897887	permutation FDR (250 perm	1
1528	Hps4~Q99KG	Hps4	ko vs wt	ko	wt	24,1157056	0,79226557	24,4500095	0,41167665	0,79316679	-0,3343038	-0,2167725	0,56583669	0,2473089	Unpaired t-t	0,614466174	permutation FDR (250 perm	1
1529	Hpx~Q91X72	Hpx	ko vs wt	ko	wt	28,1294341	0,80528105	27,094827	0,27401326	2,04855572	1,03460713	0,68804676	0,11000413	0,95859101	Unpaired t-t	0,147118136	permutation FDR (250 perm	1
1530	Hp~Q61646	Hp	ko vs wt	ko	wt	24,6258429	1,60596311	26,1951632	0,98725608	0,3369671	-1,5693203	-0,7263281	0,23460326	0,62966596	Unpaired t-t	0,124518616	permutation FDR (250 perm	1
1531	Hras~Q6141.	Hras	ko vs wt	ko	wt	25,2737486	1,35768653	25,1366369	0,394698	1,09970134	0,13711176	0,07484004	0,87767691	0,05666533	Unpaired t-t	0,873372207	permutation FDR (250 perm	1
1532	Hrg~Q9ESB3	Hrg	ko vs wt	ko	wt	31,3410057	0,44681029	31,1591699	0,18108267	1,13432642	0,18183586	0,1411773	0,56000997	0,25180424	Unpaired t-t	0,748582228	permutation FDR (250 perm	1
1533	Hs1bp3~Q3T	Hs1bp3	ko vs wt	ko	wt	26,6106517	0,44649128	26,3200002	0,29371462	1,22319256	0,29065153	0,21836469	0,42013087	0,37661541	Unpaired t-t	0,610243643	permutation FDR (250 perm	1

1534	Hsd11b1~P5(	Hsd11b1	ko vs wt	ko	wt	24,8126211	1,13593843	24,3297872	0,47761907	1,39748602	0,48283385	0,27786045	0,54582042	0,26295022	Unpaired t-te	0,520698647	permutation FDR (250 perm	1
1535	Hsd17b10~Q	Hsd17b10	ko vs wt	ko	wt	30,3497238	0,16179814	30,1832656	0,23589563	1,12229992	0,16645827	0,13974239	0,44366426	0,35294556	Unpaired t-te	0,755599437	permutation FDR (250 perm	1
1536	Hsd17b11~Q	Hsd17b11	ko vs wt	ko	wt	28,8903155	0,57743497	28,5321458	0,78696774	1,28179873	0,35816974	0,21724463	0,61508917	0,21106192	Unpaired t-te	0,621539187	permutation FDR (250 perm	1
1537	Hsd17b12~O	Hsd17b12	ko vs wt	ko	wt	30,1884538	0,08800371	29,9776748	0,4747554	1,15731289	0,21077896	0,15735339	0,59559866	0,22504629	Unpaired t-te	0,736133673	permutation FDR (250 perm	1
1538	Hsd17b4~P5:	Hsd17b4	ko vs wt	ko	wt	30,4563585	0,41287909	30,4682666	0,1597045	0,99177991	-0,0119081	-0,0094227	0,96606567	0,01499335	Unpaired t-te	0,984045953	permutation FDR (250 perm	1
1539	Hsd17b8~P5(	Hsd17b8	ko vs wt	ko	wt	27,2851309	0,22316193	27,4156475	0,17483458	0,9135043	-0,1305166	-0,1107423	0,49843586	0,30239072	Unpaired t-te	0,805055385	permutation FDR (250 perm	1
1540	Hsdl2~Q2TP/	Hsdl2	ko vs wt	ko	wt	28,762937	0,45434742	28,7389542	0,37340388	1,01676255	0,0239828	0,01747775	0,95122591	0,02171633	Unpaired t-te	0,969366903	permutation FDR (250 perm	1
1541	Hsp90aa1~P(	Hsp90aa1	ko vs wt	ko	wt	32,0513411	0,11089983	32,1370899	0,27172618	0,9422953	-0,0857488	-0,0713072	0,70636728	0,15096943	Unpaired t-te	0,883258817	permutation FDR (250 perm	1
1542	Hsp90ab1~P:	Hsp90ab1	ko vs wt	ko	wt	33,8985143	0,14024678	34,1020193	0,15693102	0,86843817	-0,203505	-0,1789262	0,21347948	0,67064387	Unpaired t-te	0,683470436	permutation FDR (250 perm	1
1543	Hsp90b1~P0{	Hsp90b1	ko vs wt	ko	wt	33,2295359	0,37281722	33,0655502	0,14302988	1,12037804	0,16398562	0,13247908	0,52697953	0,27820625	Unpaired t-te	0,765452093	permutation FDR (250 perm	1
1544	Hspa12a~Q8	Hspa12a	ko vs wt	ko	wt	26,9627237	0,20057559	27,6729291	0,25581508	0,6112331	-0,7102054	-0,5846366	0,03515881	1,45396581	Unpaired t-te	0,207789634	permutation FDR (250 perm	1
1545	Hspa12b~Q9	Hspa12b	ko vs wt	ko	wt	30,1055974	0,18272772	30,1399767	0,26347627	0,97645178	-0,0343793	-0,0283166	0,88185823	0,05460123	Unpaired t-te	0,95082775	permutation FDR (250 perm	1
1546	Hspa13~Q8B	Hspa13	ko vs wt	ko	wt	26,4173885	0,35487589	26,0309025	0,49464558	1,30720554	0,386486	0,27500869	0,4040533	0,39356134	Unpaired t-te	0,532689951	permutation FDR (250 perm	1
1547	Hspa14~Q99	Hspa14	ko vs wt	ko	wt	26,7672617	0,43129253	26,5581304	0,21001619	1,15599191	0,2091313	0,16212643	0,50544073	0,29632976	Unpaired t-te	0,710359268	permutation FDR (250 perm	1
1548	Hspa1a~Q61	Hspa1a	ko vs wt	ko	wt	32,052856	0,35528661	32,2125368	0,24632031	0,8952231	-0,1596808	-0,1258224	0,57877637	0,23748921	Unpaired t-te	0,775719809	permutation FDR (250 perm	1
1549	Hspa2~P171!	Hspa2	ko vs wt	ko	wt	30,3846654	0,69212854	30,8732913	0,12271735	0,71270361	-0,4886259	-0,3468104	0,31133625	0,50677031	Unpaired t-te	0,435477309	permutation FDR (250 perm	1
1550	Hspa4l~P487	Hspa4l	ko vs wt	ko	wt	25,7212013	1,80052391	26,962781	0,23687968	0,42290934	-1,2415797	-0,604781	0,31956642	0,49543886	Unpaired t-te	0,199618315	permutation FDR (250 perm	1
1551	Hspa4~Q3U2	Hspa4	ko vs wt	ko	wt	30,226711	0,10847362	30,1061339	0,4239755	1,08716964	0,12057708	0,09230657	0,72907273	0,13722915	Unpaired t-te	0,848524554	permutation FDR (250 perm	1
1552	Hspa5~P200:	Hspa5	ko vs wt	ko	wt	33,8104938	0,19770677	33,7229298	0,19250181	1,0625745	0,087564	0,07435517	0,6458583	0,18986275	Unpaired t-te	0,873448596	permutation FDR (250 perm	1
1553	Hspa8~P63(	Hspa8	ko vs wt	ko	wt	34,2041575	0,13884834	34,4513405	0,19774275	0,84253996	-0,247183	-0,2128731	0,21467625	0,66821601	Unpaired t-te	0,629714286	permutation FDR (250 perm	1
1554	Hspa9~P386	Hspa9	ko vs wt	ko	wt	31,9363722	0,14859808	31,8225178	0,07644264	1,08211543	0,1138544	0,10337232	0,31519885	0,50141538	Unpaired t-te	0,817468326	permutation FDR (250 perm	1
1555	Hspb1~P146(	Hspb1	ko vs wt	ko	wt	31,5830359	0,19430599	31,8337542	0,31979381	0,84047788	-0,2507182	-0,200186	0,39414063	0,40434879	Unpaired t-te	0,65389621	permutation FDR (250 perm	1
1556	Hspb6~Q5EB	Hspb6	ko vs wt	ko	wt	30,4443868	0,40084883	29,747656	0,13047929	1,62082782	0,69673084	0,55776697	0,05067616	1,29519633	Unpaired t-te	0,222731561	permutation FDR (250 perm	1
1557	Hspb7~P353{	Hspb7	ko vs wt	ko	wt	25,1306266	1,39018439	29,1586324	0,13090795	0,06129844	-4,0280059	-2,227949	0,01457482	1,83639691	Unpaired t-te	0,003229907	permutation FDR (250 perm	1
1558	Hspb8~Q9JK!	Hspb8	ko vs wt	ko	wt	29,661611	0,48685542	29,5294252	0,33759491	1,09595286	0,13218574	0,09657234	0,73466796	0,1339089	Unpaired t-te	0,830071133	permutation FDR (250 perm	1
1559	Hspd1~P63(	Hspd1	ko vs wt	ko	wt	31,5544997	0,30455423	31,7065499	0,23538585	0,89997057	-0,1520503	-0,1224121	0,55814485	0,25325308	Unpaired t-te	0,782065996	permutation FDR (250 perm	1
1560	Hspe1~Q644	Hspe1	ko vs wt	ko	wt	24,5209749	0,90526933	24,0469917	0,85891307	1,38893903	0,47398327	0,2631381	0,58299599	0,23433443	Unpaired t-te	0,542505334	permutation FDR (250 perm	1
1561	Hspg2~E9PZ:	Hspg2	ko vs wt	ko	wt	38,1263451	0,45596038	38,2773415	0,11268921	0,90062822	-0,1509964	-0,1184244	0,61586094	0,21051734	Unpaired t-te	0,794234375	permutation FDR (250 perm	1
1562	Hsph1~Q616	Hsph1	ko vs wt	ko	wt	29,6909409	0,38064672	29,2477061	0,24413214	1,35964956	0,44323485	0,34642354	0,17366997	0,76027527	Unpaired t-te	0,424758127	permutation FDR (250 perm	1
1563	Htatsf1~Q8B	Htatsf1	ko vs wt	ko	wt	24,3549657	0,79128732	27,0777133	0,07525201	0,15148559	-2,7227475	-1,8649743	0,00892965	2,04916553	Unpaired t-te	0,00515912	permutation FDR (250 perm	1
1564	Htra1~Q9R1:	Htra1	ko vs wt	ko	wt	32,8326767	0,31245859	33,1006793	0,55702301	0,83046856	-0,2680025	-0,1869931	0,58216437	0,23495438	Unpaired t-te	0,678285384	permutation FDR (250 perm	1
1565	Htra2~Q9JIY!	Htra2	ko vs wt	ko	wt	27,4435261	0,53238098	27,6912524	0,34291495	0,84222273	-0,2477263	-0,1780283	0,55472292	0,25592389	Unpaired t-te	0,680774603	permutation FDR (250 perm	1
1566	Htra3~Q9D2:	Htra3	ko vs wt	ko	wt	29,8065771	1,01539703	29,4625831	0,57483403	1,26926556	0,34399394	0,20077059	0,65048624	0,18676189	Unpaired t-te	0,64151595	permutation FDR (250 perm	1
1567	Htra4~A2RT(	Htra4	ko vs wt	ko	wt	28,396607	0,20609549	28,802426	0,86676954	0,75480765	-0,4058191	-0,2498359	0,57823888	0,23789271	Unpaired t-te	0,584621452	permutation FDR (250 perm	1
1568	Huwe1~Q7TI	Huwe1	ko vs wt	ko	wt	28,3965151	0,35348417	28,163523	0,58179032	1,17526988	0,23299209	0,15966805	0,64701306	0,18908695	Unpaired t-te	0,722521047	permutation FDR (250 perm	1
1569	Hyou1~Q9JKI	Hyou1	ko vs wt	ko	wt	30,0781054	0,46781434	29,8334093	0,22187333	1,18484318	0,24469612	0,18645612	0,4719107	0,32614018	Unpaired t-te	0,668193904	permutation FDR (250 perm	1
1570	Iah1~Q9DB2!	Iah1	ko vs wt	ko	wt	25,3359758	1,08047918	24,5294589	0,70908835	1,74898377	0,8065169	0,44797969	0,35985133	0,44387688	Unpaired t-te	0,30878185	permutation FDR (250 perm	1
1571	Iars1~Q8BU:	Iars1	ko vs wt	ko	wt	30,416702	0,25124652	30,1816567	0,02684166	1,17694371	0,23504532	0,20504809	0,20350339	0,69142835	Unpaired t-te	0,644590643	permutation FDR (250 perm	1
1572	Iars2~Q8BIJ(	Iars2	ko vs wt	ko	wt	25,595834	1,06569705	26,4806606	0,29743024	0,5415526	-0,8848266	-0,536183	0,25120127	0,59997812	Unpaired t-te	0,240931123	permutation FDR (250 perm	1
1573	Ibsp~Q61711	Ibsp	ko vs wt	ko	wt	25,9519087	0,69522884	25,4758371	2,7010905	1,39095093	0,47607152	0,16128826	0,82836527	0,08177812	Unpaired t-te	0,728664584	permutation FDR (250 perm	1
1574	Icam1~P135!	Icam1	ko vs wt	ko	wt	27,3137506	0,57418735	25,322074	1,20661022	3,97698905	1,99167659	1,03985423	0,13113951	0,88226644	Unpaired t-te	0,054513216	permutation FDR (250 perm	1
1575	Ica~Q9DBD0	Ica	ko vs wt	ko	wt	24,4485232	0,49923037	23,8277674	0,55335072	1,53768058	0,62075585	0,41774203	0,27093441	0,56713584	Unpaired t-te	0,347339912	permutation FDR (250 perm	1
1576	Ide~F6RPJ9	Ide	ko vs wt	ko	wt	24,9117303	0,44817084	24,664476	0,87435234	1,186946	0,2472543	0,14803673	0,73913262	0,13127763	Unpaired t-te	0,745221124	permutation FDR (250 perm	1
1577	Idh1~O8884	Idh1	ko vs wt	ko	wt	27,9801029	0,46829178	27,961719	0,20115847	1,01282427	0,01838388	0,01408185	0,95465888	0,02015178	Unpaired t-te	0,975348862	permutation FDR (250 perm	1
1578	Idh2~P54071	Idh2	ko vs wt	ko	wt	30,8439834	0,35137688	30,6969511	0,2077415	1,10728937	0,1470323	0,11758195	0,58300508	0,23432766	Unpaired t-te	0,791527638	permutation FDR (250 perm	1
1579	Idh3a~Q9D6I	Idh3a	ko vs wt	ko	wt	30,8792314	0,29190188	31,0393622	0,52407403	0,89494389	-0,1601309	-0,1138022	0,72178919	0,14158962	Unpaired t-te	0,806028905	permutation FDR (250 perm	1
1580	Idh3b~Q91V/	Idh3b	ko vs wt	ko	wt	31,0672263	0,21496067	31,1809101	0,23342055	0,92422514	-0,1136838	-0,0942255	0,61099559	0,21396193	Unpaired t-te	0,836997523	permutation FDR (250 perm	1
1581	Idh3g~P7040	Idh3g	ko vs wt	ko	wt	30,8491508	0,26871936	30,7542665	0,38320583	1,06797976	0,09488431	0,07230717	0,77943419	0,10822055	Unpaired t-te	0,878709081	permutation FDR (250 perm	1
1582	Ifi47~Q6163!	Ifi47	ko vs wt	ko	wt	28,212508	1,45339084	26,5347624	0,56381054	3,19927641	1,67774564	0,86974523	0,14201758	0,84765788	Unpaired t-te	0,077969261	permutation FDR (250 perm	1
1583	Ifih1~Q8R5F	Ifih1	ko vs wt	ko	wt	25,0209608	1,05479247	23,472323	0,41901053	2,92540786	1,54863778	0,92332782	0,08051834	1,09410516	Unpaired t-te	0,063699895	permutation FDR (250 perm	1
1584	Ifitm3~Q9CC	Ifitm3	ko vs wt	ko	wt	25,4459387	0,88051648	24,2555274	0,26347406	2,28217798	1,19041131	0,77227758	0,09715603	1,01253024	Unpaired t-te	0,113320755	permutation FDR (250 perm	1
1585	Ift140~E9PY	Ift140	ko vs wt	ko	wt	24,3744952	0,74074591	23,7256729	0,05290365	1,56788778	0,6488223	0,45394321	0,22654898	0,6448379	Unpaired t-te	0,319620892	permutation FDR (250 perm	1
1586	Igf1r~Q6075	Igf1r	ko vs wt	ko	wt	24,1429338	0,39412382	24,4344501	0,28070606	0,81704289	-0,2915163	-0,2239069	0,37884747	0,42153561	Unpaired t-te	0,60048057	permutation FDR (250 perm	1
1587	Igf2bp2~Q5S	Igf2bp2	ko vs wt	ko	wt	24,3236772	1,18524561	24,4907931	0,29825414	0,89062135	-0,1671159	-0,0973833	0,82829656	0,08181414	Unpaired t-te	0,83227438	permutation FDR (250 perm	1
1588	Igf2r~Q0711	Igf2r	ko vs wt	ko	wt	27,1118852	0,5601881	26,7795098	0,13869774	1,25908475	0,3323754	0,24841812	0,38785476	0,41133088	Unpaired t-te	0,569909064	permutation FDR (250 perm	1
1589	Igfals~P7038	Igfals	ko vs wt	ko	wt	25,6235782	0,24265195	24,8936847	0,69406206	1,65851657	0,72989342	0,48325152	0,27215713	0,56518028	Unpaired t-te	0,305411271	permutation FDR (250 perm	1
1590	Igfbp5~Q070	Igfbp5	ko vs wt	ko	wt	26,0589109	1,1637347	27,5073837	1,04149306	0,36640909	-1,4484728	-0,7253661	0,21093336	0,67585473	Unpaired t-te	0,126770435	permutation FDR (250 perm	1
1591	Igfbp6~P478	Igfbp6	ko vs wt	ko	wt	28,6116153	0,79601352	28,6284122	0,17325526	0,98842475	-0,016797	-0,0113829	0,97379307	0,01153332	Unpaired t-te	0,981065342	permutation FDR (250 perm	1
1592	Igfbp7~F8WI	Igfbp7	ko vs wt	ko	wt	32,4087742	0,42893338	32,8601575	0,31620165	0,73134129	-0,4513833	-0,3384581	0,23459777	0,62967613	Unpaired t-te	0,434287556	permutation FDR (250 perm	1
1593	Igha~A0A07!	Igha	ko vs wt	ko	wt	27,9014346	0,27104171	27,5285692	0,82807138	1,29492219	0,37286541	0,23215796	0,59406605	0,22616526	Unpaired t-te	0,60973913	permutation FDR (250 perm	1
1594	Ighg1~A0A0	Ighg1	ko vs wt	ko	wt	28,5417447	0,29381415	28,60558	1,61855887	0,9567174	-0,0638353	-0,0295945	0,96082767	0,0173545	Unpaired t-te	0,950644157	permutation FDR (250 perm	1
1595	Ighg2b~A0A(	Ighg2b	ko vs wt	ko	wt	32,3221061	0,42509782	32,5796211	0,40766185	0,83652757	-0,257515	-0,186796	0,53044905	0,27535632	Unpaired t-te	0,668406386	permutation FDR (250 perm	1
1596	Ighg2c~A0A(	Ighg2c	ko vs wt	ko	wt	32,0272568	0,52282268	31,8555456	0,59995832	1,1263937	0,17171117	0,11291866	0,75860817	0,11998248	Unpaired t-te	0,803492205	permutation FDR (250 perm	1
1597	Ighg3~A0A0	Ighg3	ko vs wt	ko	wt	31,4075443	1,87673595	29,4197575	0,23305129	3,96628076	1,9877868	0,94837437	0,16331854	0,78696452	Unpaired t-te	0,066456758	permutation FDR (250 perm	1

1598	Ighm~A0A07	Ighm	ko vs wt	ko	wt	35,1433745	0,48593017	35,4144518	0,31966988	0,82870046	-0,2710774	-0,1992801	0,48570688	0,31362574	Unpaired t-te	0,643671249	permutation FDR (250 perm	1
1599	Ighv1-18~A0.	Ighv1-18	ko vs wt	ko	wt	24,6830231	1,0209535	24,9852459	1,01106511	0,81100188	-0,3022228	-0,156869	0,75951419	0,11946411	Unpaired t-te	0,720806378	permutation FDR (250 perm	1
1600	Ighv1-19~A0.	Ighv1-19	ko vs wt	ko	wt	25,0809933	0,42048647	24,0595182	0,14168835	2,02999345	1,02147507	0,80900639	0,01823404	1,73911712	Unpaired t-te	0,097452359	permutation FDR (250 perm	1
1601	Ighv1-22~A0.	Ighv1-22	ko vs wt	ko	wt	25,8319642	0,97414938	25,2409342	3,16018604	1,5063217	0,59102992	0,17886794	0,81910489	0,08666048	Unpaired t-te	0,697724455	permutation FDR (250 perm	1
1602	Ighv1-31~A0.	Ighv1-31	ko vs wt	ko	wt	28,7805886	0,54263689	29,9861762	0,84057624	0,4335927	-1,2055876	-0,7210928	0,16750676	0,77596765	Unpaired t-te	0,140385847	permutation FDR (250 perm	1
1603	Ighv1-62-2~A	Ighv1-62-2	ko vs wt	ko	wt	26,2046691	0,68216416	26,8420094	0,02369545	0,6428971	-0,6373403	-0,4571356	0,20402288	0,69032114	Unpaired t-te	0,31725052	permutation FDR (250 perm	1
1604	Ighv1-72~A0.	Ighv1-72	ko vs wt	ko	wt	26,6545142	1,77796505	27,6314022	0,29800486	0,50807452	-0,976888	-0,4770162	0,41516085	0,38178361	Unpaired t-te	0,294169301	permutation FDR (250 perm	1
1605	Ighv1-76~A0.	Ighv1-76	ko vs wt	ko	wt	24,4912091	0,72215067	24,6918909	1,85186402	0,87013926	-0,2006818	-0,0845247	0,89522515	0,04806773	Unpaired t-te	0,860890347	permutation FDR (250 perm	1
1606	Ighv1-77~A0.	Ighv1-77	ko vs wt	ko	wt	25,5300024	2,00420698	28,9992493	0,59198307	0,09029269	-3,4692469	-1,555356	0,05170866	1,28643672	Unpaired t-te	0,008528529	permutation FDR (250 perm	1
1607	Ighv1-78~A0.	Ighv1-78	ko vs wt	ko	wt	28,272645	1,25412532	27,4856761	1,74458125	1,7254455	0,7869689	0,32380154	0,61678238	0,20986804	Unpaired t-te	0,463552882	permutation FDR (250 perm	1
1608	Ighv10-1~A0.	Ighv10-1	ko vs wt	ko	wt	23,9225234	0,41589607	24,6342542	1,07438107	0,61058718	-0,7117308	-0,3961221	0,451994	0,34486733	Unpaired t-te	0,392011928	permutation FDR (250 perm	1
1609	Ighv14-2~A0.	Ighv14-2	ko vs wt	ko	wt	24,123466	0,52856714	25,777784	2,85004527	0,31768789	-1,654318	-0,5444951	0,49897635	0,30192004	Unpaired t-te	0,260499639	permutation FDR (250 perm	1
1610	Ighv14-4~A0.	Ighv14-4	ko vs wt	ko	wt	25,0258877	0,93464444	24,0334078	0,81277918	1,98960199	0,99247985	0,55497037	0,26738366	0,57286514	Unpaired t-te	0,218904439	permutation FDR (250 perm	1
1611	Ighv3-6~P18!	Ighv3-6	ko vs wt	ko	wt	24,3334921	0,40212298	25,3201081	1,03728401	0,50466013	-0,986616	-0,5576189	0,30936237	0,50953251	Unpaired t-te	0,243463235	permutation FDR (250 perm	1
1612	Ighv4-1~A0A	Ighv4-1	ko vs wt	ko	wt	25,3612487	1,4529544	28,1028131	1,11677067	0,14952261	-2,7415644	-1,2739158	0,06422417	1,19230152	Unpaired t-te	0,016342328	permutation FDR (250 perm	1
1613	Ighv5-12~A0.	Ighv5-12	ko vs wt	ko	wt	24,8647246	1,65928499	24,1988517	0,89286798	1,58652787	0,66587286	0,31009449	0,5877832	0,23078283	Unpaired t-te	0,471602774	permutation FDR (250 perm	1
1614	Ighv5-16~A0.	Ighv5-16	ko vs wt	ko	wt	24,6289286	1,08774632	24,2842932	0,80626317	1,26983005	0,34463543	0,18647179	0,70149555	0,15397508	Unpaired t-te	0,666614402	permutation FDR (250 perm	1
1615	Ighv5-4~A0A	Ighv5-4	ko vs wt	ko	wt	26,9564415	0,66943802	28,6834289	0,33418938	0,30208209	-1,7269875	-1,1885535	0,01411508	1,8503167	Unpaired t-te	0,022345593	permutation FDR (250 perm	1
1616	Ighv5-6~A0A	Ighv5-6	ko vs wt	ko	wt	25,9367358	1,92116277	25,6512412	2,10461899	1,21882802	0,28549458	0,0999606	0,88517877	0,05296901	Unpaired t-te	0,8253234	permutation FDR (250 perm	1
1617	Ighv5-9-1~A(	Ighv5-9-1	ko vs wt	ko	wt	26,7997202	0,70416542	28,1061428	1,47773163	0,40432222	-1,3064226	-0,6158834	0,3392758	0,46944712	Unpaired t-te	0,201073321	permutation FDR (250 perm	1
1618	Ighv6-3~A0A	Ighv6-3	ko vs wt	ko	wt	24,0120801	0,63698071	25,3026761	1,45662458	0,40878211	-1,290596	-0,6164261	0,33748697	0,47174298	Unpaired t-te	0,202331928	permutation FDR (250 perm	1
1619	Ighv6-6~A0A	Ighv6-6	ko vs wt	ko	wt	25,1791302	1,5623206	24,2837637	0,26135501	1,86008242	0,89536654	0,46615625	0,39754735	0,40061113	Unpaired t-te	0,305428071	permutation FDR (250 perm	1
1620	Ighv7-3~A0A	Ighv7-3	ko vs wt	ko	wt	24,9763063	1,13996473	24,3567685	0,81431387	1,53638282	0,61953773	0,33051063	0,51045487	0,29204265	Unpaired t-te	0,443309859	permutation FDR (250 perm	1
1621	Ighv9-3~A0A	Ighv9-3	ko vs wt	ko	wt	26,9418454	2,65331359	27,0111786	0,75043644	0,95307838	-0,0693332	-0,026451	0,96810383	0,01407806	Unpaired t-te	0,953302424	permutation FDR (250 perm	1
1622	Igkc~P01837	Igkc	ko vs wt	ko	wt	30,9945878	0,64002032	31,4121599	0,58799295	0,74868353	-0,4175721	-0,2683201	0,49000036	0,3098036	Unpaired t-te	0,534040991	permutation FDR (250 perm	1
1623	Igkv1-135~A(	Igkv1-135	ko vs wt	ko	wt	24,8487609	1,20167021	25,4132162	1,5755093	0,67621066	-0,5644553	-0,244097	0,69225972	0,15973094	Unpaired t-te	0,577479123	permutation FDR (250 perm	1
1624	Igkv13-85~A(	Igkv13-85	ko vs wt	ko	wt	26,1931676	2,34706962	25,2051263	1,49214219	1,98349022	0,98804129	0,36359699	0,59005245	0,22910938	Unpaired t-te	0,402387118	permutation FDR (250 perm	1
1625	Igkv16-104~/	Igkv16-104	ko vs wt	ko	wt	24,7250756	1,35078778	25,5965893	1,62556422	0,54657305	-0,8715138	-0,3647963	0,56666611	0,24667276	Unpaired t-te	0,409155206	permutation FDR (250 perm	1
1626	Igkv19-93~A(	Igkv19-93	ko vs wt	ko	wt	26,4177373	0,30822073	25,2062109	0,90701719	2,31582527	1,21152641	0,72738681	0,19303425	0,71436564	Unpaired t-te	0,150363561	permutation FDR (250 perm	1
1627	Igkv4-50~A0/	Igkv4-50	ko vs wt	ko	wt	25,6075734	1,38199019	24,1963248	0,16049737	2,65967235	1,41124853	0,78145551	0,17467049	0,75778047	Unpaired t-te	0,116619517	permutation FDR (250 perm	1
1628	Igkv4-57-1~A	Igkv4-57-1	ko vs wt	ko	wt	25,0600618	1,33366655	26,0792353	1,19084343	0,49339894	-1,0191735	-0,4760208	0,41603171	0,38087357	Unpaired t-te	0,282450654	permutation FDR (250 perm	1
1629	Igkv5-39~A0/	Igkv5-39	ko vs wt	ko	wt	29,8232393	0,68142999	30,4103032	1,06552421	0,66569632	-0,5870639	-0,3173366	0,53812681	0,26911537	Unpaired t-te	0,475800945	permutation FDR (250 perm	1
1630	Igkv6-17~P01	Igkv6-17	ko vs wt	ko	wt	25,1119928	1,52903056	24,361127	0,82814433	1,68280242	0,7508658	0,36461296	0,51123996	0,2913752	Unpaired t-te	0,401983569	permutation FDR (250 perm	1
1631	Igkv8-27~A0/	Igkv8-27	ko vs wt	ko	wt	28,9459391	1,08916825	28,6163421	0,06634722	1,25666234	0,32959706	0,20213496	0,63696856	0,195882	Unpaired t-te	0,64981797	permutation FDR (250 perm	1
1632	Igkv8-28~A0/	Igkv8-28	ko vs wt	ko	wt	26,0449266	1,51301651	25,3459138	0,77150701	1,62339357	0,6990128	0,34435903	0,52915578	0,27641645	Unpaired t-te	0,4281528	permutation FDR (250 perm	1
1633	Igkv9-120~P(	Igkv9-120	ko vs wt	ko	wt	24,3957756	0,30530991	24,9007384	0,85975749	0,70467857	-0,5049628	-0,3092279	0,49794991	0,30281434	Unpaired t-te	0,498078407	permutation FDR (250 perm	1
1634	Iglc1~A0A0G	Iglc1	ko vs wt	ko	wt	26,717565	0,32647997	28,6479165	1,03129828	0,26236524	-1,9303515	-1,1010415	0,10977391	0,95950086	Unpaired t-te	0,049369553	permutation FDR (250 perm	1
1635	Iglc2~Q99JC1	Iglc2	ko vs wt	ko	wt	24,7576724	1,75933729	23,8628992	0,53862007	1,85931764	0,89477326	0,42918706	0,45820821	0,33893714	Unpaired t-te	0,336369736	permutation FDR (250 perm	1
1636	Igsf8~Q8R36	Igsf8	ko vs wt	ko	wt	26,3876357	0,20828424	26,2157144	0,43891946	1,12655773	0,17192124	0,12898815	0,64606716	0,18972233	Unpaired t-te	0,779835524	permutation FDR (250 perm	1
1637	Igtp~Q9DCE9	Igtp	ko vs wt	ko	wt	26,7473497	2,52709909	25,819298	0,68100168	1,90270471	0,92805168	0,36588857	0,58157164	0,23539678	Unpaired t-te	0,409083879	permutation FDR (250 perm	1
1638	Ikbip~E9QMI	Ikbip	ko vs wt	ko	wt	27,196056	0,83672517	25,7537258	1,17635183	2,71759444	1,44233017	0,73516621	0,22203576	0,65357707	Unpaired t-te	0,132615252	permutation FDR (250 perm	1
1639	Ikbip~Q9DBZ	Ikbip	ko vs wt	ko	wt	25,3364951	1,78736767	25,8862864	1,11448053	0,68311893	-0,5497913	-0,2392027	0,68965081	0,16137075	Unpaired t-te	0,576626014	permutation FDR (250 perm	1
1640	Ikbkb~Q5D0E	Ikbkb	ko vs wt	ko	wt	27,5853836	0,4864293	26,7764575	0,24850083	1,75190696	0,80892616	0,60762965	0,061442	1,21153464	Unpaired t-te	0,183793427	permutation FDR (250 perm	1
1641	Ikbkg~O8852	Ikbkg	ko vs wt	ko	wt	24,8193514	0,51449988	23,8078505	0,50604778	2,01600728	1,01150085	0,69041696	0,09001358	1,04569196	Unpaired t-te	0,143661216	permutation FDR (250 perm	1
1642	Ik~Q9Z1M8	Ik	ko vs wt	ko	wt	27,5492106	0,61212462	26,7743108	0,49573526	1,71107119	0,77489979	0,51736881	0,18247386	0,73879935	Unpaired t-te	0,24702853	permutation FDR (250 perm	1
1643	Il17b~Q9QXT	Il17b	ko vs wt	ko	wt	24,8870332	0,35444226	24,8089056	0,8577981	1,05564715	0,07812769	0,04763464	0,91212552	0,03994539	Unpaired t-te	0,921551042	permutation FDR (250 perm	1
1644	Il17d~A0A0B	Il17d	ko vs wt	ko	wt	24,4526947	1,07089455	23,619207	0,69859325	1,78198816	0,83348775	0,46527598	0,34048042	0,46790786	Unpaired t-te	0,290658691	permutation FDR (250 perm	1
1645	Il1rn~Q3TBV	Il1rn	ko vs wt	ko	wt	24,8943307	0,82959819	24,9010594	0,70678806	0,99534691	-0,0067287	-0,0039762	0,99264254	0,00320712	Unpaired t-te	0,992799584	permutation FDR (250 perm	1
1646	Il33~Q8BVZ5	Il33	ko vs wt	ko	wt	26,500656	0,72402356	24,7220573	1,12174647	3,43092764	1,7785987	0,93778197	0,1384261	0,858782	Unpaired t-te	0,069420676	permutation FDR (250 perm	1
1647	Il6st~Q0056(	Il6st	ko vs wt	ko	wt	24,9282692	0,60201112	25,1968743	0,25672079	0,83012176	-0,2686051	-0,1929467	0,52794335	0,27741268	Unpaired t-te	0,657571803	permutation FDR (250 perm	1
1648	Ilf2~Q9CXY6	Ilf2	ko vs wt	ko	wt	27,8602946	0,68053719	28,0717029	0,06117871	0,86369372	-0,2114083	-0,1515165	0,62905906	0,20130858	Unpaired t-te	0,736419145	permutation FDR (250 perm	1
1649	Ilf3~Q9Z1X4-	Ilf3	ko vs wt	ko	wt	29,966361	0,03768917	29,9423205	0,1654827	1,01680327	0,02404058	0,0214836	0,85752359	0,06675392	Unpaired t-te	0,964464408	permutation FDR (250 perm	1
1650	Ilk~O55222	Ilk	ko vs wt	ko	wt	34,594514	0,12500454	34,7592449	0,14154003	0,89209488	-0,1647309	-0,1466371	0,25420739	0,59481183	Unpaired t-te	0,740151541	permutation FDR (250 perm	1
1651	Ilvbl~Q8BU3	Ilvbl	ko vs wt	ko	wt	26,5493492	0,13405208	27,0311699	0,08602692	0,71607339	-0,4818206	-0,4386411	0,00451216	2,34561527	Unpaired t-te	0,31830156	permutation FDR (250 perm	1
1652	Immt~Q8CA(	Immt	ko vs wt	ko	wt	32,2286225	0,23352254	32,4400719	0,30937838	0,86366913	-0,2114494	-0,1682211	0,46330496	0,33413305	Unpaired t-te	0,704486827	permutation FDR (250 perm	1
1653	Impa1~Q924	Impa1	ko vs wt	ko	wt	24,1180987	0,55771199	27,5105792	0,79968501	0,09522733	-3,3924804	-2,0551592	0,01096672	1,95992344	Unpaired t-te	0,00368336	permutation FDR (250 perm	1
1654	Impact~O55(	Impact	ko vs wt	ko	wt	26,9577492	0,1977477	26,896853	0,16095706	1,0431135	0,06089615	0,05244203	0,72172381	0,14162896	Unpaired t-te	0,909872683	permutation FDR (250 perm	1
1655	Impdh2~P24!	Impdh2	ko vs wt	ko	wt	26,7824985	0,30323959	25,0598628	0,98508649	3,30038799	1,72263563	1,00256617	0,12433552	0,9054048	Unpaired t-te	0,067165289	permutation FDR (250 perm	1
1656	Ina~P46660	Ina	ko vs wt	ko	wt	23,7180829	0,50840041	27,8055731	0,23612452	0,05882241	-4,0874902	-3,0556339	0,00012528	3,90212428	Unpaired t-te	0,000584795	permutation FDR (250 perm	1
1657	Inf2~Q0GNC	Inf2	ko vs wt	ko	wt	27,6244594	1,00032416	27,2785543	0,10964406	1,27094806	0,34590507	0,21855128	0,59321916	0,22678483	Unpaired t-te	0,6219835	permutation FDR (250 perm	1
1658	Inhbb~Q049!	Inhbb	ko vs wt	ko	wt	24,7643544	1,17114822	25,2433443	0,5211463	0,71747977	-0,4789899	-0,2706066	0,56435027	0,24845126	Unpaired t-te	0,530922794	permutation FDR (250 perm	1
1659	Inhbe~O0871	Inhbe	ko vs wt	ko	wt	26,2937898	0,72295927	25,1974282	0,99369819	2,13814793	1,09636167	0,60329921	0,26278597	0,58039783	Unpaired t-te	0,197614495	permutation FDR (250 perm	1
1660	Inmt~P4093(	Inmt	ko vs wt	ko	wt	25,7414088	0,71262834	27,4491031	0,48027709	0,30614896	-1,7076943	-1,1135991	0,02397277	1,62028184	Unpaired t-te	0,02899152	permutation FDR (250 perm	1
1661	Inpp1~P4944	Inpp1	ko vs wt	ko	wt	24,415378	0,68660983	25,0040943	0,72913734	0,66493428	-0,5887163	-0,3567205	0,41529843	0,38163971	Unpaired t-te	0,41625163	permutation FDR (250 perm	1

1662	Inpp4a~E9Q	Inpp4a	ko vs wt	ko	wt	28,5264271	0,59983006	28,6061877	0,15163885	0,94621463	-0,0797606	-0,0585385	0,83791251	0,07680132	Unpaired t-te	0,900061389	permutation FDR (250 perm	1
1663	Inpp5a~Q7T	Inpp5a	ko vs wt	ko	wt	26,621045	0,64793833	26,5669917	0,64961669	1,03817761	0,05405328	0,03394446	0,93141316	0,03085763	Unpaired t-te	0,941606575	permutation FDR (250 perm	1
1664	Inpp5d~Q9E	Inpp5d	ko vs wt	ko	wt	27,8776417	1,66097081	25,4862096	0,2038131	5,24677901	2,39143203	1,21409077	0,08672917	1,06183481	Unpaired t-te	0,026623742	permutation FDR (250 perm	1
1665	Inppl1~Q6P5	Inppl1	ko vs wt	ko	wt	26,5869219	0,3089727	26,7640674	0,03343165	0,88445118	-0,1771456	-0,1501304	0,39525853	0,40311875	Unpaired t-te	0,738462932	permutation FDR (250 perm	1
1666	Insr~P15208	Insr	ko vs wt	ko	wt	25,9728871	0,17782008	24,0993227	1,32581246	3,66436807	1,87356442	0,96421628	0,18233916	0,73912004	Unpaired t-te	0,07994077	permutation FDR (250 perm	1
1667	Ints4~Q8CIM	Ints4	ko vs wt	ko	wt	24,6203353	1,1095726	24,5729773	0,45187849	1,03337079	0,04735801	0,02759988	0,95023092	0,02217084	Unpaired t-te	0,951259298	permutation FDR (250 perm	1
1668	Ints5~Q8CHT	Ints5	ko vs wt	ko	wt	24,5714594	1,01324359	23,9935927	0,52590591	1,49264043	0,57786667	0,34128917	0,44436326	0,35226186	Unpaired t-te	0,431603487	permutation FDR (250 perm	1
1669	Ipo4~Q8VI75	Ipo4	ko vs wt	ko	wt	25,2419801	1,52935238	25,6821085	1,2082268	0,73706902	-0,4401284	-0,1974878	0,73521047	0,13358832	Unpaired t-te	0,646555004	permutation FDR (250 perm	1
1670	Ipo5~Q8BKC	Ipo5	ko vs wt	ko	wt	28,0552433	1,07170521	28,2492209	0,46195483	0,87419221	-0,1939776	-0,1141259	0,79416039	0,10009178	Unpaired t-te	0,800224268	permutation FDR (250 perm	1
1671	Ipo7~Q9EPL	Ipo7	ko vs wt	ko	wt	23,811821	0,92772621	25,1685652	0,85977513	0,39046248	-1,3567442	-0,7494805	0,16133497	0,79227149	Unpaired t-te	0,117815907	permutation FDR (250 perm	1
1672	Ipo9~Q91YE	Ipo9	ko vs wt	ko	wt	26,5223731	0,37648404	26,1086732	0,39360033	1,33209771	0,41369991	0,30573343	0,30418587	0,51686097	Unpaired t-te	0,482500785	permutation FDR (250 perm	1
1673	Iqgap1~Q9JK	Iqgap1	ko vs wt	ko	wt	34,0688902	0,18676138	34,1613674	0,39689112	0,9379109	-0,0924772	-0,071101	0,7813765	0,10713966	Unpaired t-te	0,883314875	permutation FDR (250 perm	1
1674	Iqgap2~Q3U	Iqgap2	ko vs wt	ko	wt	28,0340826	1,32685304	27,3337116	0,28562155	1,62492253	0,70037094	0,39078077	0,43462456	0,36188574	Unpaired t-te	0,382822274	permutation FDR (250 perm	1
1675	Iqsec1~Q8RC	Iqsec1	ko vs wt	ko	wt	27,0270433	0,31342821	27,6088098	0,63868657	0,66814516	-0,5817665	-0,3913602	0,32781457	0,48437174	Unpaired t-te	0,392760769	permutation FDR (250 perm	1
1676	Irag1~Q9WU	Irag1	ko vs wt	ko	wt	30,7392598	0,25651583	30,5980032	0,21267784	1,10286531	0,1412566	0,11663812	0,53435628	0,27216908	Unpaired t-te	0,793667876	permutation FDR (250 perm	1
1677	Irak4~Q8R4K	Irak4	ko vs wt	ko	wt	26,4655257	0,23467155	24,8963145	0,85559845	2,96742422	1,56921119	0,96865792	0,11565129	0,93684951	Unpaired t-te	0,07517	permutation FDR (250 perm	1
1678	Irf2bp1~Q8R	Irf2bp1	ko vs wt	ko	wt	24,7397684	0,79925107	24,8160147	0,7001902	0,94852235	-0,0762463	-0,0454712	0,91500677	0,03857569	Unpaired t-te	0,921576697	permutation FDR (250 perm	1
1679	Irf2bpl~Q8K3	Irf2bpl	ko vs wt	ko	wt	26,2772226	0,16783395	23,9799597	1,32104762	4,91524356	2,29726291	1,18468492	0,13163734	0,88062091	Unpaired t-te	0,041809805	permutation FDR (250 perm	1
1680	Irgm1~Q607	Irgm1	ko vs wt	ko	wt	27,3185289	0,86750968	25,9874862	0,55168469	2,51584446	1,33104273	0,81416766	0,09025552	1,04452623	Unpaired t-te	0,090390892	permutation FDR (250 perm	1
1681	Isg15~Q6433	Isg15	ko vs wt	ko	wt	28,5016168	0,72034343	27,5663523	0,61412461	1,91224116	0,93526448	0,5840725	0,18411022	0,7349221	Unpaired t-te	0,199505964	permutation FDR (250 perm	1
1682	Isg20~Q9JL1	Isg20	ko vs wt	ko	wt	24,5669967	1,7659655	24,6718167	1,22866177	0,92992093	-0,10482	-0,0448038	0,94066852	0,02656339	Unpaired t-te	0,922563719	permutation FDR (250 perm	1
1683	Islr~Q6GU68	Islr	ko vs wt	ko	wt	27,6707147	0,07896265	27,9817723	0,51448731	0,80605062	-0,3110577	-0,2276071	0,48320142	0,3158718	Unpaired t-te	0,62181261	permutation FDR (250 perm	1
1684	Isoc1~Q91V	Isoc1	ko vs wt	ko	wt	28,230257	0,28027633	25,398101	2,275876	7,12137583	2,83215599	1,08204834	0,21954364	0,65847913	Unpaired t-te	0,056951551	permutation FDR (250 perm	1
1685	Isoc2a~P850	Isoc2a	ko vs wt	ko	wt	24,5366703	1,86781966	24,0669592	1,22420021	1,38483209	0,46971106	0,19712227	0,74791124	0,12614994	Unpaired t-te	0,646688065	permutation FDR (250 perm	1
1686	Ist1~Q9CX00	Ist1	ko vs wt	ko	wt	25,9017925	1,97228018	27,8799398	0,62695935	0,25381562	-1,9781473	-0,8902742	0,18363104	0,73605392	Unpaired t-te	0,074660661	permutation FDR (250 perm	1
1687	Isyna1~Q9JH	Isyna1	ko vs wt	ko	wt	25,7849219	1,50093262	27,911547	0,7403123	0,22899292	-2,1266251	-1,0567579	0,09376926	1,02793949	Unpaired t-te	0,036950061	permutation FDR (250 perm	1
1688	Itch~Q8C863	Itch	ko vs wt	ko	wt	27,7315096	0,14838322	27,4485394	0,50903618	1,21669719	0,28297016	0,20654794	0,51710902	0,28641789	Unpaired t-te	0,652879557	permutation FDR (250 perm	1
1689	Itfg1~Q99K	Itfg1	ko vs wt	ko	wt	25,0152488	1,0057819	24,4090938	0,36019283	1,52219697	0,60615505	0,37094378	0,39272168	0,40591513	Unpaired t-te	0,399336406	permutation FDR (250 perm	1
1690	Itga10~E9PX	Itga10	ko vs wt	ko	wt	24,5942174	0,24219534	23,6917962	1,00123863	1,86920039	0,90242124	0,52415783	0,32968671	0,48189857	Unpaired t-te	0,273523877	permutation FDR (250 perm	1
1691	Itga11~P616	Itga11	ko vs wt	ko	wt	25,8214135	1,57579705	23,8791256	0,25320542	3,84314634	1,94228791	1,00780954	0,12073215	0,91817706	Unpaired t-te	0,053728231	permutation FDR (250 perm	1
1692	Itga1~Q3V3	Itga1	ko vs wt	ko	wt	28,1693262	0,42042173	28,1708145	0,51389741	0,99896892	-0,0014883	-0,0010357	0,99745852	0,00110515	Unpaired t-te	0,998210554	permutation FDR (250 perm	1
1693	Itga2b~Q9Q	Itga2b	ko vs wt	ko	wt	26,364675	3,46095586	24,8366669	0,20304411	2,88387393	1,5280081	0,50877041	0,50059448	0,30051395	Unpaired t-te	0,269724534	permutation FDR (250 perm	1
1694	Itga2~Q6246	Itga2	ko vs wt	ko	wt	25,4322808	2,20153785	24,0496271	1,14480065	2,60747557	1,38265374	0,55153003	0,40301774	0,39467583	Unpaired t-te	0,220900225	permutation FDR (250 perm	1
1695	Itga3~Q6247	Itga3	ko vs wt	ko	wt	29,5618181	0,35021399	29,3866948	0,41151666	1,12906089	0,17512329	0,12930534	0,64830477	0,18822078	Unpaired t-te	0,772531539	permutation FDR (250 perm	1
1696	Itga5~P1168	Itga5	ko vs wt	ko	wt	29,1600467	0,23409707	29,0496973	0,42725658	1,07948967	0,11034943	0,08290902	0,76216939	0,1179485	Unpaired t-te	0,861732568	permutation FDR (250 perm	1
1697	Itga6~Q6173	Itga6	ko vs wt	ko	wt	27,3660219	2,17878066	27,103246	0,85594069	1,199785	0,2627759	0,10967499	0,85941307	0,06579805	Unpaired t-te	0,807455707	permutation FDR (250 perm	1
1698	Itga7~Q6173	Itga7	ko vs wt	ko	wt	29,0423376	0,25629036	29,330117	0,40893705	0,81916192	-0,2877794	-0,2172211	0,44000831	0,35653912	Unpaired t-te	0,625189635	permutation FDR (250 perm	1
1699	Itga8~A2AR	Itga8	ko vs wt	ko	wt	33,2239004	0,72612055	33,8274639	0,17889561	0,65812636	-0,6035635	-0,4197549	0,24904949	0,60371434	Unpaired t-te	0,349895238	permutation FDR (250 perm	1
1700	Itga9~B8JK3	Itga9	ko vs wt	ko	wt	29,0701966	1,15498063	27,0110214	0,92064401	4,1674799	2,05917524	1,06587637	0,07988197	1,09755126	Unpaired t-te	0,035300248	permutation FDR (250 perm	1
1701	Itgal~P24063	Itgal	ko vs wt	ko	wt	23,9511593	0,80374534	24,6310662	1,24158359	0,62420556	-0,6799069	-0,341143	0,54107309	0,26674406	Unpaired t-te	0,444066581	permutation FDR (250 perm	1
1702	Itgav~P4340	Itgav	ko vs wt	ko	wt	31,3101279	0,18803567	31,1863973	0,15089042	1,08954867	0,12373065	0,10738492	0,45446606	0,34249854	Unpaired t-te	0,810281909	permutation FDR (250 perm	1
1703	Itgax~Q9QX	Itgax	ko vs wt	ko	wt	28,3256335	0,93624288	27,6868307	0,38205434	1,55703656	0,63880282	0,39818414	0,34637726	0,46045063	Unpaired t-te	0,366635721	permutation FDR (250 perm	1
1704	Itgb1~P0905	Itgb1	ko vs wt	ko	wt	33,3479619	0,31410971	33,4152955	0,292782	0,95440029	-0,0673336	-0,0528013	0,81751825	0,08750254	Unpaired t-te	0,909450443	permutation FDR (250 perm	1
1705	Itgb2~P1183	Itgb2	ko vs wt	ko	wt	28,1951906	1,46694606	27,4195227	0,86493605	1,71198243	0,77566789	0,37935808	0,49161224	0,30837731	Unpaired t-te	0,384833056	permutation FDR (250 perm	1
1706	Itgb3~O5489	Itgb3	ko vs wt	ko	wt	30,3233427	0,70791612	29,8866649	0,34774815	1,35348389	0,43667771	0,2956552	0,4048988	0,39265351	Unpaired t-te	0,493313377	permutation FDR (250 perm	1
1707	Itgb4~A2A86	Itgb4	ko vs wt	ko	wt	25,2309184	0,89422536	24,9562134	1,32704426	1,20974671	0,27470502	0,13264281	0,81319307	0,08980633	Unpaired t-te	0,769211441	permutation FDR (250 perm	1
1708	Itgb5~O7030	Itgb5	ko vs wt	ko	wt	30,4786665	0,468229	30,3571497	0,48241199	1,08787802	0,1215168	0,08466611	0,79331934	0,10055196	Unpaired t-te	0,853774856	permutation FDR (250 perm	1
1709	Itih1~Q6170	Itih1	ko vs wt	ko	wt	33,282265	0,25077184	32,8615868	0,11429449	1,33855667	0,42067821	0,36084551	0,05735564	1,24142385	Unpaired t-te	0,408713443	permutation FDR (250 perm	1
1710	Itih2~Q6170	Itih2	ko vs wt	ko	wt	31,5248059	0,52997443	30,7147513	0,80916125	1,75327773	0,81005455	0,4912871	0,29693247	0,52734232	Unpaired t-te	0,282118146	permutation FDR (250 perm	1
1711	Itih3~Q6170	Itih3	ko vs wt	ko	wt	30,7952227	0,63675437	30,1874335	0,79556844	1,52392212	0,60778917	0,363505	0,4217278	0,37496777	Unpaired t-te	0,411583007	permutation FDR (250 perm	1
1712	Itih4~E9PVD	Itih4	ko vs wt	ko	wt	31,3724376	0,71680165	30,8782734	0,57659929	1,40850457	0,49416424	0,31257447	0,43512963	0,36138134	Unpaired t-te	0,468407208	permutation FDR (250 perm	1
1713	Itih5~Q8BJD	Itih5	ko vs wt	ko	wt	28,5347819	0,35488575	28,1962436	0,47790766	1,26447479	0,33853827	0,24264583	0,4473211	0,34938062	Unpaired t-te	0,579189222	permutation FDR (250 perm	1
1714	Itm2b~O890	Itm2b	ko vs wt	ko	wt	26,3104192	0,75086778	27,026294	0,1951545	0,60883581	-0,7158749	-0,4920282	0,1988326	0,70151242	Unpaired t-te	0,276885409	permutation FDR (250 perm	1
1715	Itm2c~Q91V	Itm2c	ko vs wt	ko	wt	24,8268839	0,52204396	24,1159987	1,07750428	1,63680813	0,71088522	0,39073364	0,45744976	0,3396566	Unpaired t-te	0,393594174	permutation FDR (250 perm	1
1716	Itpa~Q9D892	Itpa	ko vs wt	ko	wt	25,8995383	1,54588397	27,7102399	0,45801863	0,28505227	-1,8107016	-0,9288217	0,13402359	0,87281877	Unpaired t-te	0,065803636	permutation FDR (250 perm	1
1717	Itpkb~B2RXC	Itpkb	ko vs wt	ko	wt	24,2768291	1,07223544	24,8034128	1,40800397	0,69419664	-0,5265837	-0,2423998	0,67972282	0,16766815	Unpaired t-te	0,579107517	permutation FDR (250 perm	1
1718	Itpr1~P1188	Itpr1	ko vs wt	ko	wt	32,1638282	0,19435433	32,3214399	0,25288644	0,89650791	-0,1576118	-0,1301384	0,50205067	0,29925245	Unpaired t-te	0,772435222	permutation FDR (250 perm	1
1719	Itpr2~Q9Z32	Itpr2	ko vs wt	ko	wt	27,7960167	0,22681813	27,3712328	0,37538689	1,3423715	0,42478399	0,32776949	0,2474756	0,60646762	Unpaired t-te	0,462792142	permutation FDR (250 perm	1
1720	Itpr3~P7022	Itpr3	ko vs wt	ko	wt	28,8701914	0,04820444	28,871267	0,20596331	0,9992547	-0,0010756	-0,0009367	0,99483015	0,00225106	Unpaired t-te	0,998578633	permutation FDR (250 perm	1
1721	Itprid2~Q922	Itprid2	ko vs wt	ko	wt	24,5222006	0,92892859	24,7666118	0,86272934	0,84416023	-0,2444112	-0,1348645	0,77690331	0,10963303	Unpaired t-te	0,759705204	permutation FDR (250 perm	1
1722	Itsn1~Q9Z0R	Itsn1	ko vs wt	ko	wt	24,4903858	1,90736462	27,5690592	0,06888007	0,118366	-3,0786734	-1,4644352	0,06794638	1,16783367	Unpaired t-te	0,01268325	permutation FDR (250 perm	1
1723	Itsn2~Q9Z0R	Itsn2	ko vs wt	ko	wt	27,4054018	0,45651902	27,5472364	0,10938473	0,90636582	-0,1418346	-0,1112703	0,63649148	0,1962074	Unpaired t-te	0,806881422	permutation FDR (250 perm	1
1724	Ivd~Q9JHI5	Ivd	ko vs wt	ko	wt	26,6181429	0,33342383	26,1644718	0,22308793	1,36952071	0,45367108	0,36326235	0,12793978	0,89299439	Unpaired t-te	0,402624179	permutation FDR (250 perm	1
1725	Jak1~B1ASP	Jak1	ko vs wt	ko	wt	27,5671579	0,37228761	26,9382621	0,25057579	1,54638099	0,62889581	0,49187974	0,07356814	1,13331022	Unpaired t-te	0,267514193	permutation FDR (250 perm	1

1726	Jak2~Q6212(	Jak2	ko vs wt	ko	wt	25,4688031	0,7308343	24,2408412	1,28075106	2,34235847	1,22796188	0,61425729	0,31014784	0,50843124	Unpaired t-te	0,197501928	permutation FDR (250 perm	1
1727	Jchain~P015!	Jchain	ko vs wt	ko	wt	30,4341285	0,52069683	30,6506468	0,92011581	0,86063992	-0,2165183	-0,1261236	0,78321535	0,10611881	Unpaired t-te	0,783778523	permutation FDR (250 perm	1
1728	Jph2~Q9ET7!	Jph2	ko vs wt	ko	wt	27,7510029	0,23867529	28,2763662	0,16175998	0,69478413	-0,5253633	-0,4455683	0,03250775	1,48801311	Unpaired t-te	0,311337748	permutation FDR (250 perm	1
1729	Jup~Q02257	Jup	ko vs wt	ko	wt	25,5756023	1,16067313	24,7332588	1,44841628	1,7929603	0,84234355	0,37876322	0,53269765	0,27351922	Unpaired t-te	0,394155372	permutation FDR (250 perm	1
1730	Kalrn~A2CG4	Kalrn	ko vs wt	ko	wt	29,3058056	0,49895394	28,8785064	0,52608441	1,34471385	0,42729921	0,29058149	0,41365626	0,3833604	Unpaired t-te	0,504175047	permutation FDR (250 perm	1
1731	Kank1~E9Q2.	Kank1	ko vs wt	ko	wt	28,678184	0,25529545	29,0994818	0,44118442	0,74675256	-0,4212978	-0,3132251	0,31207911	0,5057353	Unpaired t-te	0,483521957	permutation FDR (250 perm	1
1732	Kank2~Q8BX	Kank2	ko vs wt	ko	wt	31,7150606	0,7303988	32,5232992	0,32272124	0,57107866	-0,8082386	-0,5462988	0,15997654	0,7959437	Unpaired t-te	0,22714595	permutation FDR (250 perm	1
1733	Kank4~Q6P9.	Kank4	ko vs wt	ko	wt	24,9630743	1,24949919	27,0491663	0,31566427	0,2355178	-2,086092	-1,1885609	0,05788841	1,2374084	Unpaired t-te	0,026635326	permutation FDR (250 perm	1
1734	Kars1~Q99M	Kars1	ko vs wt	ko	wt	29,3221411	0,3944155	29,5106362	0,37614291	0,8775206	-0,1884951	-0,1396118	0,6163023	0,21020621	Unpaired t-te	0,750617075	permutation FDR (250 perm	1
1735	Kat7~Q5SVQ	Kat7	ko vs wt	ko	wt	25,3964159	0,86773409	24,1380336	0,06709481	2,39227348	1,25838233	0,83712011	0,08617703	1,0646085	Unpaired t-te	0,09667194	permutation FDR (250 perm	1
1736	Katnb1~Q8B	Katnb1	ko vs wt	ko	wt	24,4076689	0,62686623	24,8485762	0,64378431	0,73667114	-0,4409074	-0,2787794	0,48862514	0,31102419	Unpaired t-te	0,520239926	permutation FDR (250 perm	1
1737	Kcnab2~P62	Kcnab2	ko vs wt	ko	wt	24,3838176	1,19570328	24,723629	0,98693935	0,79014457	-0,3398115	-0,171481	0,74393126	0,12846719	Unpaired t-te	0,692874713	permutation FDR (250 perm	1
1738	Kctd10~Q92.	Kctd10	ko vs wt	ko	wt	24,1809163	1,20465522	24,7351879	0,99093034	0,68100077	-0,5542717	-0,278911	0,5997804	0,22200773	Unpaired t-te	0,517314022	permutation FDR (250 perm	1
1739	Kctd12~Q6W	Kctd12	ko vs wt	ko	wt	27,7644698	0,64289451	27,538825	0,31149763	1,16929975	0,22564482	0,15761621	0,62517594	0,20399774	Unpaired t-te	0,718648417	permutation FDR (250 perm	1
1740	Kctd3~Q8BF	Kctd3	ko vs wt	ko	wt	24,8000612	0,75351234	23,4680646	0,75427539	2,51750851	1,33199665	0,78896782	0,12035317	0,91954248	Unpaired t-te	0,103540652	permutation FDR (250 perm	1
1741	Kdm3b~Q6ZI	Kdm3b	ko vs wt	ko	wt	24,313466	0,78397524	24,33625	0,86952875	0,98433138	-0,022784	-0,0129199	0,97763065	0,00982519	Unpaired t-te	0,977557531	permutation FDR (250 perm	1
1742	Kdsr~Q6GV1	Kdsr	ko vs wt	ko	wt	25,8290812	1,20123754	27,1816439	0,52728537	0,39159584	-1,3525627	-0,7567189	0,15398931	0,81250944	Unpaired t-te	0,114721649	permutation FDR (250 perm	1
1743	Keap1~Q9Z2	Keap1	ko vs wt	ko	wt	24,9288317	1,21994792	24,6006584	0,98071071	1,25542278	0,32817329	0,16504167	0,75380167	0,1227429	Unpaired t-te	0,704347178	permutation FDR (250 perm	1
1744	Khdrbs1~Q6	Khdrbs1	ko vs wt	ko	wt	29,5776911	0,29805086	29,5648379	0,3264536	1,00894897	0,0128532	0,00997982	0,96652547	0,0147867	Unpaired t-te	0,983520376	permutation FDR (250 perm	1
1745	Khdrbs3~Q9	Khdrbs3	ko vs wt	ko	wt	23,2587863	0,41052279	25,1529574	1,94057431	0,26902812	-1,8941711	-0,791708	0,30031931	0,52241674	Unpaired t-te	0,129817553	permutation FDR (250 perm	1
1746	Khsrp~Q3U0	Khsrp	ko vs wt	ko	wt	29,6326636	0,36771395	29,276819	0,13905442	1,27973459	0,35584464	0,28837517	0,20056298	0,69774924	Unpaired t-te	0,507067865	permutation FDR (250 perm	1
1747	Kiaa1671~Q	Kiaa1671	ko vs wt	ko	wt	28,0576937	0,13871154	27,2486054	1,05629571	1,7521038	0,80908825	0,46202065	0,39194045	0,40677991	Unpaired t-te	0,332336423	permutation FDR (250 perm	1
1748	Kidins220~E	Kidins220	ko vs wt	ko	wt	28,2884893	0,73236357	29,1072787	0,48908449	0,56691745	-0,8187894	-0,5295329	0,19416988	0,71181814	Unpaired t-te	0,236483728	permutation FDR (250 perm	1
1749	Kif13a~F8VC	Kif13a	ko vs wt	ko	wt	25,1592504	1,17432869	25,1966667	1,53999179	0,97439843	-0,0374163	-0,0163908	0,97831558	0,00952103	Unpaired t-te	0,971426925	permutation FDR (250 perm	1
1750	Kif13b~E9Q4	Kif13b	ko vs wt	ko	wt	28,254787	0,48493825	28,6324609	0,54481011	0,7696776	-0,3776738	-0,2558363	0,47278852	0,32533308	Unpaired t-te	0,556197456	permutation FDR (250 perm	1
1751	Kif1c~O3507	Kif1c	ko vs wt	ko	wt	26,9001141	0,23038758	27,4149567	0,19000253	0,69986932	-0,5148425	-0,4329832	0,04363265	1,36018837	Unpaired t-te	0,325239573	permutation FDR (250 perm	1
1752	Kif2a~P2874	Kif2a	ko vs wt	ko	wt	27,6955681	1,44979064	25,7410534	0,3416479	3,87585515	1,95451465	1,0445237	0,09821204	1,00783526	Unpaired t-te	0,044858469	permutation FDR (250 perm	1
1753	Kif3a~P2874	Kif3a	ko vs wt	ko	wt	26,2419573	0,25355155	26,1743921	0,09170447	1,04794665	0,06756527	0,05824054	0,69442185	0,15837662	Unpaired t-te	0,899599354	permutation FDR (250 perm	1
1754	Kif3b~Q6177	Kif3b	ko vs wt	ko	wt	26,5076246	0,34354904	26,560598	0,37595385	0,96394761	-0,0529734	-0,0397793	0,88081802	0,05511381	Unpaired t-te	0,931427507	permutation FDR (250 perm	1
1755	Kif5b~Q6176	Kif5b	ko vs wt	ko	wt	30,4952969	0,31957281	30,5910149	0,20362969	0,9358064	-0,095718	-0,0775647	0,69951368	0,15520379	Unpaired t-te	0,865342795	permutation FDR (250 perm	1
1756	Kifbp~Q6ZPL	Kifbp	ko vs wt	ko	wt	25,114866	1,52044616	26,4916324	0,1727335	0,38508094	-1,3767664	-0,7298819	0,21482753	0,66791007	Unpaired t-te	0,136741181	permutation FDR (250 perm	1
1757	Klc1~Q5UE5	Klc1	ko vs wt	ko	wt	27,2489339	0,67051384	27,6578943	0,20766776	0,75316594	-0,4089603	-0,2892149	0,3818207	0,41814053	Unpaired t-te	0,50828005	permutation FDR (250 perm	1
1758	Klkb1~P2626	Klkb1	ko vs wt	ko	wt	28,1765853	1,46839072	25,4640135	0,45692329	6,5548908	2,71257175	1,42223798	0,04285847	1,36796335	Unpaired t-te	0,011680544	permutation FDR (250 perm	1
1759	Kmt2a~P552	Kmt2a	ko vs wt	ko	wt	23,9788233	1,0887541	23,944666	0,5550892	1,0239585	0,03415724	0,01961849	0,96512351	0,01541711	Unpaired t-te	0,965810349	permutation FDR (250 perm	1
1760	Kng1~O0867	Kng1	ko vs wt	ko	wt	23,5311301	0,54020703	24,1535938	0,71250731	0,6495607	-0,6224637	-0,3908614	0,36080048	0,44273289	Unpaired t-te	0,381632544	permutation FDR (250 perm	1
1761	Kng1~O0867	Kng1	ko vs wt	ko	wt	30,0429161	0,81462226	30,049577	0,36165372	0,9953936	-0,006661	-0,0043384	0,99061065	0,00409701	Unpaired t-te	0,99232075	permutation FDR (250 perm	1
1762	Kng2~Q6S9I	Kng2	ko vs wt	ko	wt	24,7641605	1,38437483	23,236556	0,77074085	2,8830673	1,52760451	0,77646073	0,17679669	0,75252588	Unpaired t-te	0,104625514	permutation FDR (250 perm	1
1763	Kpna3~O353	Kpna3	ko vs wt	ko	wt	25,3573261	0,95916563	26,8149427	0,07667372	0,36409412	-1,4576166	-0,9365175	0,07749253	1,11074018	Unpaired t-te	0,070131431	permutation FDR (250 perm	1
1764	Kpna4~O353	Kpna4	ko vs wt	ko	wt	28,0961577	0,6358734	27,6569516	0,49384785	1,35585805	0,43920614	0,29150658	0,42654598	0,37003415	Unpaired t-te	0,498262601	permutation FDR (250 perm	1
1765	Kpna6~O353	Kpna6	ko vs wt	ko	wt	24,4812273	1,43963839	25,8393674	1,19584653	0,39008486	-1,3581401	-0,6213762	0,3062699	0,51389568	Unpaired t-te	0,1760761	permutation FDR (250 perm	1
1766	Kpnb1~P701	Kpnb1	ko vs wt	ko	wt	29,8745297	0,45710203	30,141846	0,44602301	0,83086371	-0,2673162	-0,1894198	0,54836504	0,26093024	Unpaired t-te	0,663654752	permutation FDR (250 perm	1
1767	Kras~P32883	Kras	ko vs wt	ko	wt	29,5388895	0,57794658	29,5626188	0,44111489	0,98368664	-0,0237293	-0,0162891	0,96061485	0,0174507	Unpaired t-te	0,971168981	permutation FDR (250 perm	1
1768	Krit1~Q6S5J	Krit1	ko vs wt	ko	wt	23,8493467	0,69454797	24,622008	0,68484891	0,58533673	-0,7726613	-0,4743934	0,28171654	0,55018765	Unpaired t-te	0,285944954	permutation FDR (250 perm	1
1769	Krt33a~Q8K	Krt33a	ko vs wt	ko	wt	27,3978674	2,36066938	30,4531076	0,04478548	0,12030427	-3,0552402	-1,2927851	0,11075661	0,95563033	Unpaired t-te	0,021007013	permutation FDR (250 perm	1
1770	Krt86~P9786	Krt86	ko vs wt	ko	wt	26,8251891	3,04176393	31,0438389	0,06342358	0,05371058	-4,2186498	-1,5303062	0,0956404	1,01935863	Unpaired t-te	0,010876761	permutation FDR (250 perm	1
1771	Krt87~Q6IM	Krt87	ko vs wt	ko	wt	25,0382503	1,58932872	23,904003	0,58924123	2,19504014	1,13424733	0,56493019	0,32186757	0,49232278	Unpaired t-te	0,215690683	permutation FDR (250 perm	1
1772	Krt8~P11679	Krt8	ko vs wt	ko	wt	26,3988087	0,66633482	26,1571065	0,09335631	1,18238686	0,24170214	0,17384501	0,57737566	0,23854153	Unpaired t-te	0,697003732	permutation FDR (250 perm	1
1773	Ktn1~F8VQC	Ktn1	ko vs wt	ko	wt	28,5494628	0,1740954	28,8882718	0,6801951	0,79069376	-0,3388091	-0,2271811	0,55667217	0,25440049	Unpaired t-te	0,620093105	permutation FDR (250 perm	1
1774	Lactb~Q9EP	Lactb	ko vs wt	ko	wt	27,3150889	0,42441799	27,0775389	0,11633486	1,17898882	0,23755004	0,18875992	0,41477258	0,38218996	Unpaired t-te	0,668953266	permutation FDR (250 perm	1
1775	Lama2~Q60	Lama2	ko vs wt	ko	wt	32,4175624	0,60338003	32,7235716	0,21620289	0,80887622	-0,3060092	-0,2216765	0,46582882	0,33177365	Unpaired t-te	0,609095858	permutation FDR (250 perm	1
1776	Lama3~Q61	Lama3	ko vs wt	ko	wt	26,336328	0,31806507	26,0477939	0,05699126	1,22139857	0,28853406	0,24287281	0,2146527	0,66826364	Unpaired t-te	0,580265789	permutation FDR (250 perm	1
1777	Lama4~P979	Lama4	ko vs wt	ko	wt	33,2243635	0,589787	33,4487983	0,28357697	0,85593029	-0,2244348	-0,1608658	0,59660615	0,22431228	Unpaired t-te	0,713157233	permutation FDR (250 perm	1
1778	Lama5~Q61	Lama5	ko vs wt	ko	wt	35,1353858	0,52231426	35,279682	0,21815401	0,90482069	-0,1442962	-0,1077865	0,69043539	0,16087696	Unpaired t-te	0,810864595	permutation FDR (250 perm	1
1779	Lamb1~P024	Lamb1	ko vs wt	ko	wt	32,4775962	0,42299354	31,5876895	0,52882409	1,85305618	0,88990662	0,61516305	0,12428341	0,90558683	Unpaired t-te	0,187719189	permutation FDR (250 perm	1
1780	Lamb2~Q61	Lamb2	ko vs wt	ko	wt	35,9465301	0,75987015	36,1824339	0,22699072	0,84915288	-0,2359038	-0,1607904	0,64174933	0,19263457	Unpaired t-te	0,716799315	permutation FDR (250 perm	1
1781	Lamc1~F8VC	Lamc1	ko vs wt	ko	wt	35,3297017	0,6493407	35,6950666	0,13644126	0,77627251	-0,3653649	-0,2633997	0,40757145	0,38979625	Unpaired t-te	0,54902399	permutation FDR (250 perm	1
1782	Lamp1~P114	Lamp1	ko vs wt	ko	wt	24,4500714	0,55234351	25,5499486	1,58754026	0,46655623	-1,0998771	-0,5075628	0,43321436	0,36329716	Unpaired t-te	0,282504422	permutation FDR (250 perm	1
1783	Lamp2~P170	Lamp2	ko vs wt	ko	wt	28,5323307	0,7489971	27,6138206	0,68878257	1,89016219	0,91851003	0,55622987	0,22339402	0,65092845	Unpaired t-te	0,218871332	permutation FDR (250 perm	1
1784	Lamtor1~Q9	Lamtor1	ko vs wt	ko	wt	26,9842708	0,12885764	27,392223	0,05481574	0,75369242	-0,4079522	-0,3763787	0,0065977	2,18060737	Unpaired t-te	0,391428761	permutation FDR (250 perm	1
1785	Lamtor3~O8	Lamtor3	ko vs wt	ko	wt	27,4219925	0,44824232	24,8104377	1,68723887	6,11161977	2,61155479	1,1759504	0,15469397	0,81052661	Unpaired t-te	0,040256684	permutation FDR (250 perm	1
1786	Lancl1~O891	Lancl1	ko vs wt	ko	wt	27,2922919	0,42494715	27,1975429	0,12046741	1,06787959	0,09474898	0,07521488	0,73519597	0,13359688	Unpaired t-te	0,873231524	permutation FDR (250 perm	1
1787	Lancl2~Q9JJ	Lancl2	ko vs wt	ko	wt	26,7994227	0,16637582	26,4282605	0,13739111	1,29339432	0,37116218	0,32654894	0,0439852	1,35669344	Unpaired t-te	0,450159055	permutation FDR (250 perm	1
1788	Lap3~Q9CPY	Lap3	ko vs wt	ko	wt	27,1862604	1,26435707	25,7236118	0,97226389	2,75613899	1,46264864	0,73031882	0,20563033	0,68691284	Unpaired t-te	0,123224274	permutation FDR (250 perm	1
1789	Larp1~Q6ZQ	Larp1	ko vs wt	ko	wt	25,05139	1,57974294	26,6661895	0,11405172	0,32651033	-1,6147995	-0,8429628	0,17458369	0,75799634	Unpaired t-te	0,095226954	permutation FDR (250 perm	1

1790	Larp7~Q05CL	Larp7	ko vs wt	ko	wt	26,3608336	0,62958662	26,507826	0,06831393	0,9031313	-0,1469923	-0,1075537	0,71451429	0,14598908	Unpaired t-te	0,814912815	permutation FDR (250 perm	1
1791	Lars1~Q8BM	Lars1	ko vs wt	ko	wt	30,0387626	0,3459208	29,7186024	0,08111372	1,24846915	0,32016017	0,26507928	0,20858404	0,68071893	Unpaired t-te	0,546042579	permutation FDR (250 perm	1
1792	Lasp1~Q617	Lasp1	ko vs wt	ko	wt	25,5673192	0,89239462	27,2087082	0,76654005	0,32054771	-1,641389	-0,9391017	0,08315722	1,08010005	Unpaired t-te	0,058299621	permutation FDR (250 perm	1
1793	Lbp~Q61805	Lbp	ko vs wt	ko	wt	27,6740877	0,48785055	27,2416734	0,27935514	1,34949005	0,43241434	0,32173116	0,26537358	0,57614231	Unpaired t-te	0,455698185	permutation FDR (250 perm	1
1794	Lbr~Q3U9G9	Lbr	ko vs wt	ko	wt	24,3357956	0,78326019	25,2877054	1,7937395	0,51694771	-0,9519097	-0,4056601	0,53946253	0,26803871	Unpaired t-te	0,379549315	permutation FDR (250 perm	1
1795	Lcn2~P11672	Lcn2	ko vs wt	ko	wt	25,1727985	1,46926706	24,6880998	0,500549	1,39929364	0,48469875	0,25255765	0,62615546	0,20331783	Unpaired t-te	0,560944377	permutation FDR (250 perm	1
1796	Lcp1~Q6123	Lcp1	ko vs wt	ko	wt	29,9784756	1,02494953	29,1244094	0,80579508	1,80758832	0,85406614	0,46888624	0,34736228	0,45921734	Unpaired t-te	0,287716602	permutation FDR (250 perm	1
1797	Lcp2~Q6078	Lcp2	ko vs wt	ko	wt	24,5062116	1,97792429	24,5847207	0,78694701	0,94703583	-0,0785091	-0,0345807	0,95352738	0,02066683	Unpaired t-te	0,940381862	permutation FDR (250 perm	1
1798	Ldb3~Q9JKS	Ldb3	ko vs wt	ko	wt	30,7506737	0,37236807	31,1249152	0,2853985	0,77151095	-0,3742415	-0,2890196	0,26126601	0,58291709	Unpaired t-te	0,502254274	permutation FDR (250 perm	1
1799	Ldha~P0615	Ldha	ko vs wt	ko	wt	31,9291472	0,12788972	31,3937178	0,09561165	1,44937347	0,53542939	0,48670375	0,00319121	2,49604502	Unpaired t-te	0,271204862	permutation FDR (250 perm	1
1800	Ldhb~P1612	Ldhb	ko vs wt	ko	wt	28,4918145	0,66727357	28,4551938	1,06503056	1,02570844	0,0366207	0,01983884	0,96815222	0,01405636	Unpaired t-te	0,96665721	permutation FDR (250 perm	1
1801	Ldlrap1~Q8C	Ldlrap1	ko vs wt	ko	wt	25,2615373	1,28290759	26,0074686	0,11862144	0,59628281	-0,7459313	-0,4273647	0,3890526	0,40999168	Unpaired t-te	0,346552757	permutation FDR (250 perm	1
1802	Lemd2~Q6D	Lemd2	ko vs wt	ko	wt	27,9984847	0,36805418	27,9884211	0,33697456	1,00699989	0,01006353	0,00762813	0,97619383	0,01046394	Unpaired t-te	0,986415016	permutation FDR (250 perm	1
1803	Lemd3~Q9W	Lemd3	ko vs wt	ko	wt	25,6599516	0,95642276	24,091288	1,14273083	2,96629808	1,56866358	0,79277934	0,18878035	0,72404321	Unpaired t-te	0,105654795	permutation FDR (250 perm	1
1804	Leng8~Q8CB	Leng8	ko vs wt	ko	wt	24,6970397	0,3917016	23,9601564	0,58047782	1,66657162	0,73688332	0,5017464	0,20822588	0,6814653	Unpaired t-te	0,272572448	permutation FDR (250 perm	1
1805	Letm1~Q9Z2	Letm1	ko vs wt	ko	wt	29,7057513	0,34601654	29,3541467	0,54416587	1,27597898	0,35160456	0,24526808	0,47542267	0,32292011	Unpaired t-te	0,578773381	permutation FDR (250 perm	1
1806	Letmd1~Q92	Letmd1	ko vs wt	ko	wt	28,097621	1,03142532	28,0184231	0,68226717	1,05643051	0,07919787	0,04483595	0,92171134	0,03540507	Unpaired t-te	0,922454715	permutation FDR (250 perm	1
1807	Lfng~O0901	Lfng	ko vs wt	ko	wt	27,4600793	0,62268398	25,8845752	0,34212688	2,98039616	1,57550411	1,09919534	0,01582694	1,80060303	Unpaired t-te	0,031269231	permutation FDR (250 perm	1
1808	Lgals1~P160	Lgals1	ko vs wt	ko	wt	32,789833	0,67673168	33,4581469	0,05334673	0,62924166	-0,6683139	-0,4799285	0,18552776	0,73159111	Unpaired t-te	0,294035827	permutation FDR (250 perm	1
1809	Lgals3bp~Q0	Lgals3bp	ko vs wt	ko	wt	28,1702081	0,9260922	27,3623757	1,02038529	1,75057935	0,80783246	0,4256141	0,41926199	0,3775145	Unpaired t-te	0,3385987	permutation FDR (250 perm	1
1810	Lgals3~P161	Lgals3	ko vs wt	ko	wt	33,4747769	0,42304627	32,7745578	0,45366873	1,62475159	0,70021916	0,49901882	0,15608417	0,80664113	Unpaired t-te	0,267364714	permutation FDR (250 perm	1
1811	Lgals8~Q9JL	Lgals8	ko vs wt	ko	wt	28,2641916	0,46369763	27,9108867	0,65903297	1,27748369	0,35330487	0,22980182	0,55392745	0,25654711	Unpaired t-te	0,600612281	permutation FDR (250 perm	1
1812	Lgals9~O085	Lgals9	ko vs wt	ko	wt	29,605953	0,24548374	29,1904231	0,7495449	1,33378851	0,41552993	0,26832078	0,51907765	0,28476767	Unpaired t-te	0,555887341	permutation FDR (250 perm	1
1813	Lgalsl~Q8VE	Lgalsl	ko vs wt	ko	wt	25,1979809	2,15435249	23,9682612	0,94007218	2,34521416	1,22971967	0,51019362	0,42806693	0,36848832	Unpaired t-te	0,255340595	permutation FDR (250 perm	1
1814	Lgi3~Q8K40	Lgi3	ko vs wt	ko	wt	24,3895824	0,98126449	24,2745661	1,09089952	1,08298733	0,11501637	0,05876955	0,9101812	0,04087214	Unpaired t-te	0,898468498	permutation FDR (250 perm	1
1815	Lgmn~O890	Lgmn	ko vs wt	ko	wt	28,8314906	0,47739287	28,0816561	0,32589106	1,68159984	0,74983443	0,55164747	0,0912608	1,03971574	Unpaired t-te	0,21947218	permutation FDR (250 perm	1
1816	Lilrb3~P9748	Lilrb3	ko vs wt	ko	wt	24,8458627	0,74219605	24,8260638	0,51320601	1,01381819	0,01979896	0,01267926	0,97323898	0,01178051	Unpaired t-te	0,977751046	permutation FDR (250 perm	1
1817	Lima1~Q9ER	Lima1	ko vs wt	ko	wt	27,9305366	0,80663948	27,6312056	0,43701056	1,23057365	0,29933101	0,19201325	0,61602918	0,21039872	Unpaired t-te	0,657254879	permutation FDR (250 perm	1
1818	Limch1~Q3U	Limch1	ko vs wt	ko	wt	26,9657043	0,40627227	26,7425083	0,27227285	1,16731663	0,22319594	0,17123402	0,49505918	0,30534288	Unpaired t-te	0,692532184	permutation FDR (250 perm	1
1819	Limd1~Q9Q	Limd1	ko vs wt	ko	wt	28,386193	0,43738104	28,833611	1,07032615	0,73335413	-0,4474181	-0,2488627	0,62206221	0,20616618	Unpaired t-te	0,581079916	permutation FDR (250 perm	1
1820	Limd2~Q8B	Limd2	ko vs wt	ko	wt	25,2531135	0,62606542	24,1834404	0,20077639	2,09895777	1,06967314	0,77046897	0,05320763	1,2740261	Unpaired t-te	0,113074573	permutation FDR (250 perm	1
1821	Limk1~P536	Limk1	ko vs wt	ko	wt	24,4589875	0,7519493	24,6711674	0,24998277	0,86323188	-0,2121799	-0,1444635	0,67473939	0,17086394	Unpaired t-te	0,744282486	permutation FDR (250 perm	1
1822	Lims1~E9QP	Lims1	ko vs wt	ko	wt	29,8332991	0,34936924	29,7546467	0,16005571	1,05603117	0,07865241	0,06387804	0,74905351	0,12548716	Unpaired t-te	0,889419076	permutation FDR (250 perm	1
1823	Lims2~Q91X	Lims2	ko vs wt	ko	wt	32,5773326	0,37320554	33,0298036	0,05098593	0,7307901	-0,452471	-0,3713449	0,12530216	0,90204145	Unpaired t-te	0,407556576	permutation FDR (250 perm	1
1824	Lin7c~O8895	Lin7c	ko vs wt	ko	wt	27,5888898	0,51992915	27,5772714	0,06592871	1,00808583	0,01161847	0,00891138	0,97179146	0,01242692	Unpaired t-te	0,98568564	permutation FDR (250 perm	1
1825	Lipa~Q9Z0M	Lipa	ko vs wt	ko	wt	25,1416273	1,15051322	23,7913609	0,46941365	2,54959211	1,35026646	0,77487333	0,13837216	0,85895127	Unpaired t-te	0,108107127	permutation FDR (250 perm	1
1826	Lipc~P27656	Lipc	ko vs wt	ko	wt	26,3989474	0,62983286	25,2876568	0,38473099	2,16038826	1,11129061	0,76422785	0,05849641	1,23287076	Unpaired t-te	0,108486388	permutation FDR (250 perm	1
1827	Lipe~P54310	Lipe	ko vs wt	ko	wt	28,9330387	0,41468988	28,7311474	0,55898855	1,15020522	0,20189129	0,13808102	0,68827321	0,16223914	Unpaired t-te	0,75782616	permutation FDR (250 perm	1
1828	Llgl1~Q80Y1	Llgl1	ko vs wt	ko	wt	26,3146714	0,3924513	26,7347097	0,32107876	0,74740477	-0,4200383	-0,3180284	0,24957444	0,60279989	Unpaired t-te	0,461119289	permutation FDR (250 perm	1
1829	Lman1~Q9D	Lman1	ko vs wt	ko	wt	28,5952599	0,51009433	28,2603563	0,52344034	1,26129307	0,33490353	0,22736186	0,51616198	0,28721399	Unpaired t-te	0,598810939	permutation FDR (250 perm	1
1830	Lman2~Q9D	Lman2	ko vs wt	ko	wt	28,1856082	1,0453365	27,1855274	0,08012175	2,00011191	1,00008072	0,62264601	0,19597394	0,70780168	Unpaired t-te	0,189869988	permutation FDR (250 perm	1
1831	Lmcd1~Q8V	Lmcd1	ko vs wt	ko	wt	31,7103857	0,61922106	32,0412559	0,81349432	0,79505679	-0,3308702	-0,1972668	0,65420847	0,18428384	Unpaired t-te	0,654749053	permutation FDR (250 perm	1
1832	Lmna~P4867	Lmna	ko vs wt	ko	wt	35,7975109	0,0977082	36,1359458	0,22518422	0,7908988	-0,338435	-0,2895262	0,1566161	0,80516358	Unpaired t-te	0,521619662	permutation FDR (250 perm	1
1833	Lmnb1~P147	Lmnb1	ko vs wt	ko	wt	32,8195472	0,09445191	32,7534234	0,09714537	1,04690015	0,06612384	0,06079202	0,49094038	0,30897124	Unpaired t-te	0,894523591	permutation FDR (250 perm	1
1834	Lmnb2~P216	Lmnb2	ko vs wt	ko	wt	31,2537271	0,44827136	31,6904566	0,17418843	0,73880751	-0,4367296	-0,3394384	0,19907331	0,70098696	Unpaired t-te	0,436777099	permutation FDR (250 perm	1
1835	Lmod1~F8VF	Lmod1	ko vs wt	ko	wt	33,0512427	0,87988252	33,9258011	0,12337773	0,54542079	-0,8745584	-0,5770998	0,18324841	0,73695978	Unpaired t-te	0,216352808	permutation FDR (250 perm	1
1836	Lnpep~Q8C1	Lnpep	ko vs wt	ko	wt	29,5780998	0,45702656	29,6902275	0,33172547	0,92522256	-0,1121276	-0,0828702	0,76371456	0,11706893	Unpaired t-te	0,855752532	permutation FDR (250 perm	1
1837	Lonp1~Q8CG	Lonp1	ko vs wt	ko	wt	29,4987016	0,17307748	28,8353173	0,4991441	1,58379356	0,6633843	0,48534832	0,19435715	0,71139947	Unpaired t-te	0,303411352	permutation FDR (250 perm	1
1838	Lonp2~Q9DB	Lonp2	ko vs wt	ko	wt	26,3107893	0,3682826	26,3999888	0,59536032	0,94004416	-0,0891996	-0,0606126	0,86199082	0,06449736	Unpaired t-te	0,897049286	permutation FDR (250 perm	1
1839	Loxl1~P9787	Loxl1	ko vs wt	ko	wt	33,8695061	0,40802881	34,1028584	0,44268006	0,85065597	-0,2333523	-0,1676666	0,58315877	0,23421319	Unpaired t-te	0,702936085	permutation FDR (250 perm	1
1840	Loxl2~P5802	Loxl2	ko vs wt	ko	wt	28,8259342	0,73990334	28,4778906	0,78204739	1,27283346	0,34804366	0,20487935	0,64384218	0,19122057	Unpaired t-te	0,637603984	permutation FDR (250 perm	1
1841	Loxl3~Q9Z17	Loxl3	ko vs wt	ko	wt	31,0672895	0,28474687	30,7268271	0,26073596	1,26616237	0,34046243	0,27302097	0,2322362	0,63407008	Unpaired t-te	0,526607362	permutation FDR (250 perm	1
1842	Loxl4~Q924C	Loxl4	ko vs wt	ko	wt	26,4687113	0,42455381	26,6083888	0,40188187	0,907722	-0,1396776	-0,1015629	0,72682957	0,13856741	Unpaired t-te	0,821573915	permutation FDR (250 perm	1
1843	Lox~P28301	Lox	ko vs wt	ko	wt	33,4451817	0,61512457	33,7215882	0,07152395	0,82564498	-0,2764065	-0,2034308	0,49511163	0,30529687	Unpaired t-te	0,646399299	permutation FDR (250 perm	1
1844	Lpcat2~Q8B	Lpcat2	ko vs wt	ko	wt	24,3974785	0,33072872	23,9868877	1,81671522	1,32922996	0,41059072	0,17861669	0,78070304	0,10751413	Unpaired t-te	0,700819042	permutation FDR (250 perm	1
1845	Lpcat3~Q91	Lpcat3	ko vs wt	ko	wt	23,977492	0,57246056	24,8041999	0,24397011	0,56381437	-0,8267079	-0,6021951	0,08514407	1,0698456	Unpaired t-te	0,189901869	permutation FDR (250 perm	1
1846	Lpgat1~Q91	Lpgat1	ko vs wt	ko	wt	24,3534803	0,80343563	23,7366988	0,22182914	1,53345035	0,61678146	0,41403984	0,28227026	0,54933488	Unpaired t-te	0,354264995	permutation FDR (250 perm	1
1847	Lpin1~Q91Z	Lpin1	ko vs wt	ko	wt	26,9417093	0,53711202	27,3884086	0,29718612	0,73371962	-0,4466992	-0,3249674	0,28809914	0,54045803	Unpaired t-te	0,451239796	permutation FDR (250 perm	1
1848	Lpin2~Q99P	Lpin2	ko vs wt	ko	wt	24,5966194	0,79911569	23,9990451	0,52091589	1,5131703	0,59757436	0,37574511	0,35791081	0,44622518	Unpaired t-te	0,388151344	permutation FDR (250 perm	1
1849	Lpl~P11152	Lpl	ko vs wt	ko	wt	30,8349299	0,71704029	30,1078272	0,2304756	1,65531146	0,7271027	0,50321783	0,18005981	0,74458322	Unpaired t-te	0,265317986	permutation FDR (250 perm	1
1850	Lpp~Q8BFW	Lpp	ko vs wt	ko	wt	33,6445663	0,56005998	34,2357368	0,2727768	0,66380409	-0,5911706	-0,4294708	0,18172985	0,74057372	Unpaired t-te	0,329531798	permutation FDR (250 perm	1
1851	Lpxn~Q99N6	Lpxn	ko vs wt	ko	wt	26,1151784	0,76667241	25,6740561	0,32393388	1,35766004	0,44112228	0,29439694	0,42229404	0,37438505	Unpaired t-te	0,496803238	permutation FDR (250 perm	1
1852	Lrba~Q9ESE	Lrba	ko vs wt	ko	wt	25,6381102	1,51293015	24,5699036	0,17346491	2,09682521	1,0682066	0,5675729	0,30962314	0,50916659	Unpaired t-te	0,223964112	permutation FDR (250 perm	1
1853	Lrch1~P6204	Lrch1	ko vs wt	ko	wt	26,6094721	0,25555263	26,8699463	0,50592907	0,83481351	-0,2604741	-0,1877999	0,55421053	0,25632523	Unpaired t-te	0,678356481	permutation FDR (250 perm	1

1854	Lrch3~Q8BVl	Lrch3	ko vs wt	ko	wt	27,6810682	0,10434962	27,3139457	0,24575362	1,28977773	0,36712246	0,31009026	0,15853782	0,79986713	Unpaired t-te	0,494141565	permutation FDR (250 perm	1
1855	Lrp1~Q91ZX7	Lrp1	ko vs wt	ko	wt	32,9968484	0,32307477	32,6046297	0,22956949	1,31241025	0,39221876	0,31446125	0,1737889	0,75997797	Unpaired t-te	0,465096897	permutation FDR (250 perm	1
1856	Lrpap1~P553	Lrpap1	ko vs wt	ko	wt	25,5059551	0,54514364	24,2022623	0,92386093	2,46859957	1,30369283	0,75570504	0,1722036	0,76395776	Unpaired t-te	0,128644993	permutation FDR (250 perm	1
1857	Lrpprc~Q6PB	Lrpprc	ko vs wt	ko	wt	30,4628692	0,38439451	30,5550225	0,18226585	0,93812156	-0,0921532	-0,0733331	0,73548114	0,13342846	Unpaired t-te	0,874663035	permutation FDR (250 perm	1
1858	Lrrc17~Q9CX	Lrrc17	ko vs wt	ko	wt	32,7622776	0,84042559	33,7257536	0,20539324	0,51281983	-0,963476	-0,6395505	0,13957793	0,85518325	Unpaired t-te	0,174985714	permutation FDR (250 perm	1
1859	Lrrc32~G3XA	Lrrc32	ko vs wt	ko	wt	24,8607317	0,35517468	23,3083726	0,11057951	2,93296333	1,55235903	1,27298674	0,00251461	2,59952875	Unpaired t-te	0,018712209	permutation FDR (250 perm	1
1860	Lrrc40~Q9CR	Lrrc40	ko vs wt	ko	wt	25,7321991	0,29788396	25,7943897	0,42453102	0,9578087	-0,0621906	-0,0462052	0,86784572	0,06155748	Unpaired t-te	0,922144308	permutation FDR (250 perm	1
1861	Lrrc41~Q8K1	Lrrc41	ko vs wt	ko	wt	24,5151327	0,48541182	24,2443694	0,37096988	1,20644593	0,27076326	0,19565755	0,51263969	0,29018777	Unpaired t-te	0,650052509	permutation FDR (250 perm	1
1862	Lrrc47~Q505	Lrrc47	ko vs wt	ko	wt	28,7489417	0,53541549	28,4481257	0,31034743	1,23184091	0,30081595	0,21812534	0,46405813	0,33342761	Unpaired t-te	0,610901567	permutation FDR (250 perm	1
1863	Lrrc57~Q8JZ	Lrrc57	ko vs wt	ko	wt	26,0171798	1,41408024	24,5360476	0,37291336	2,79167728	1,48113217	0,7971875	0,16726607	0,77659215	Unpaired t-te	0,105466179	permutation FDR (250 perm	1
1864	Lrrc59~Q922	Lrrc59	ko vs wt	ko	wt	29,8931168	0,50979344	29,533325	0,43625163	1,28324068	0,35979179	0,25224376	0,43956087	0,35698098	Unpaired t-te	0,557325666	permutation FDR (250 perm	1
1865	Lrrc8c~Q8R5	Lrrc8c	ko vs wt	ko	wt	25,5243665	1,29652734	24,6969345	0,43864224	1,77452385	0,82743197	0,45707752	0,36566003	0,43692251	Unpaired t-te	0,306709091	permutation FDR (250 perm	1
1866	Lrrfip1~G5E8	Lrrfip1	ko vs wt	ko	wt	25,4220929	2,1671578	27,6622046	0,73608592	0,21166993	-2,2401117	-0,951154	0,17455656	0,75806383	Unpaired t-te	0,059208155	permutation FDR (250 perm	1
1867	Lrrk2~Q5S00	Lrrk2	ko vs wt	ko	wt	25,7792033	0,44146341	25,176322	0,28813909	1,51874673	0,6028813	0,45455684	0,12395593	0,90673268	Unpaired t-te	0,30180939	permutation FDR (250 perm	1
1868	Lsm2~O3590	Lsm2	ko vs wt	ko	wt	27,3635003	0,41622916	27,0120543	0,23628906	1,27583871	0,35144596	0,27187261	0,28477869	0,54549251	Unpaired t-te	0,526603681	permutation FDR (250 perm	1
1869	Lsm4~Q9CY4	Lsm4	ko vs wt	ko	wt	27,784707	0,36403611	27,1549129	0,3146839	1,54734415	0,62979411	0,48220051	0,0985917	1,00615964	Unpaired t-te	0,276967972	permutation FDR (250 perm	1
1870	Lsm8~Q6ZW	Lsm8	ko vs wt	ko	wt	24,6663397	1,01379131	23,0299782	0,75980846	3,10880787	1,63636146	0,91187115	0,09525153	1,02112805	Unpaired t-te	0,062950578	permutation FDR (250 perm	1
1871	Lsp1~P19973	Lsp1	ko vs wt	ko	wt	24,1647231	0,88456808	24,4038712	0,24777457	0,84724547	-0,2391481	-0,1552988	0,68264837	0,16580294	Unpaired t-te	0,726192045	permutation FDR (250 perm	1
1872	Lta4h~P2452	Lta4h	ko vs wt	ko	wt	25,3051983	1,15376781	27,0414813	0,83722609	0,30014197	-1,736283	-0,9180954	0,10931569	0,96131748	Unpaired t-te	0,061419492	permutation FDR (250 perm	1
1873	Ltbp1~Q8CG	Ltbp1	ko vs wt	ko	wt	34,1299727	0,72535624	34,4120892	0,20156667	0,82238369	-0,2821164	-0,1955917	0,56132943	0,25078219	Unpaired t-te	0,656828671	permutation FDR (250 perm	1
1874	Ltbp2~E9Q1	Ltbp2	ko vs wt	ko	wt	32,6354464	0,30641187	32,2918955	0,37322963	1,26887582	0,34355088	0,26071607	0,34490251	0,46230364	Unpaired t-te	0,550526252	permutation FDR (250 perm	1
1875	Ltbp3~F8VQ	Ltbp3	ko vs wt	ko	wt	29,4146101	1,05805723	30,8485447	0,37550831	0,3701201	-1,4339346	-0,8606628	0,09724651	1,01212599	Unpaired t-te	0,081177734	permutation FDR (250 perm	1
1876	Ltbp4~D3Z5	Ltbp4	ko vs wt	ko	wt	33,1985879	1,02953441	33,7414426	0,36947299	0,68641133	-0,5428547	-0,3291458	0,44965831	0,34711738	Unpaired t-te	0,451039898	permutation FDR (250 perm	1
1877	Ltf~P08071	Ltf	ko vs wt	ko	wt	27,9855336	0,85604079	26,836156	1,27757984	2,21818182	1,14937762	0,56626715	0,34138518	0,46675534	Unpaired t-te	0,224014953	permutation FDR (250 perm	1
1878	Ltn1~Q6A00	Ltn1	ko vs wt	ko	wt	23,9670956	0,56323891	24,3054028	1,12993903	0,79096887	-0,3383072	-0,181629	0,72411167	0,14019445	Unpaired t-te	0,687759494	permutation FDR (250 perm	1
1879	Luc7l2~Q7TN	Luc7l2	ko vs wt	ko	wt	27,9608407	0,30765774	28,661917	0,73153334	0,61511313	-0,7010763	-0,4532078	0,30673841	0,51323184	Unpaired t-te	0,32995189	permutation FDR (250 perm	1
1880	Luc7l3~Q5SL	Luc7l3	ko vs wt	ko	wt	27,0866163	0,64896801	26,7293964	0,22311804	1,28095513	0,35721994	0,25397013	0,42953157	0,36700491	Unpaired t-te	0,558571083	permutation FDR (250 perm	1
1881	Lum~P51885	Lum	ko vs wt	ko	wt	34,1351538	0,2882396	34,3841706	0,33911013	0,84146967	-0,2490168	-0,1927559	0,44397346	0,35264299	Unpaired t-te	0,660960419	permutation FDR (250 perm	1
1882	Luzp1~Q8R4	Luzp1	ko vs wt	ko	wt	24,5570344	0,25908896	27,8389723	0,14944036	0,10281068	-3,2819379	-2,7739113	1,16E-05	4,93516515	Unpaired t-te	0,00077551	permutation FDR (250 perm	1
1883	Lxn~P70202	Lxn	ko vs wt	ko	wt	26,5526515	2,65961283	28,0571709	0,57904693	0,35244759	-1,5045194	-0,5810794	0,4058172	0,39166955	Unpaired t-te	0,211276725	permutation FDR (250 perm	1
1884	Lyg1~Q9D7Q	Lyg1	ko vs wt	ko	wt	26,364124	0,19307559	26,094111	0,25253855	1,20581869	0,27001299	0,22305745	0,27780785	0,55625549	Unpaired t-te	0,610520402	permutation FDR (250 perm	1
1885	Lyn~P25911	Lyn	ko vs wt	ko	wt	27,7655269	2,25573472	26,5246797	0,29585069	2,36337287	1,24084726	0,53506846	0,41316726	0,3838741	Unpaired t-te	0,247134503	permutation FDR (250 perm	1
1886	Lypla1~P978	Lypla1	ko vs wt	ko	wt	27,6123715	0,39241756	25,6231923	1,62941568	3,97011043	1,98917914	0,91488684	0,22328261	0,6511451	Unpaired t-te	0,089302857	permutation FDR (250 perm	1
1887	Lypla2~Q9W	Lypla2	ko vs wt	ko	wt	25,9738487	0,62343535	26,6653293	0,58790854	0,61921802	-0,6914806	-0,4461491	0,27047394	0,56787458	Unpaired t-te	0,314145391	permutation FDR (250 perm	1
1888	Lyst~P97412	Lyst	ko vs wt	ko	wt	24,3464332	0,35153968	23,8103209	0,97647589	1,45005967	0,53611227	0,31175034	0,52372633	0,28089559	Unpaired t-te	0,494374065	permutation FDR (250 perm	1
1889	Lyz2~P08905	Lyz2	ko vs wt	ko	wt	28,2966515	0,46189561	28,2466955	0,11965705	1,03524733	0,04997596	0,03905017	0,86787556	0,06154254	Unpaired t-te	0,9328557	permutation FDR (250 perm	1
1890	Lyz3~Q8BM2	Lyz3	ko vs wt	ko	wt	25,3566883	0,83467233	24,6551962	0,18264721	1,62618579	0,70149209	0,46800311	0,2437416	0,61307034	Unpaired t-te	0,301145461	permutation FDR (250 perm	1
1891	Macf1~E9PV	Macf1	ko vs wt	ko	wt	31,6060906	0,41330865	31,7584128	0,38526054	0,89980096	-0,1523222	-0,1118246	0,69342393	0,15900118	Unpaired t-te	0,803775056	permutation FDR (250 perm	1
1892	Macf1~F7AC	Macf1	ko vs wt	ko	wt	27,2247814	0,43754715	25,4449113	1,08312575	3,43395246	1,77987007	0,98527428	0,13475391	0,87045863	Unpaired t-te	0,067386997	permutation FDR (250 perm	1
1893	Macrod1~Q9	Macrod1	ko vs wt	ko	wt	27,9303523	0,78053311	28,5061586	0,48055713	0,67091119	-0,5758063	-0,368069	0,35468508	0,45015708	Unpaired t-te	0,397029664	permutation FDR (250 perm	1
1894	Macroh2a1~	Macroh2a1	ko vs wt	ko	wt	31,5806977	0,15134597	31,8167548	0,11168763	0,84906262	-0,2360571	-0,2111838	0,10197637	0,99150043	Unpaired t-te	0,623618262	permutation FDR (250 perm	1
1895	Macroh2a2~	Macroh2a2	ko vs wt	ko	wt	30,9287024	0,46200994	31,3323124	0,38211037	0,75596425	-0,4036101	-0,2925394	0,33854813	0,47037958	Unpaired t-te	0,497145347	permutation FDR (250 perm	1
1896	Mamdc2~Q8	Mamdc2	ko vs wt	ko	wt	29,4977748	1,16973039	30,380545	0,65787976	0,54232509	-0,8827702	-0,4850235	0,33207045	0,47876977	Unpaired t-te	0,273038516	permutation FDR (250 perm	1
1897	Man2a1~P27	Man2a1	ko vs wt	ko	wt	28,1307695	1,21969869	26,7371912	0,67022967	2,62729515	1,39357828	0,75376711	0,16345036	0,78661412	Unpaired t-te	0,113478222	permutation FDR (250 perm	1
1898	Man2c1~Q91	Man2c1	ko vs wt	ko	wt	27,3779032	0,2502671	27,2604918	0,52465034	1,08478671	0,11741141	0,08397751	0,78991113	0,10242177	Unpaired t-te	0,860695652	permutation FDR (250 perm	1
1899	Manf~Q80ZF	Manf	ko vs wt	ko	wt	27,4237738	0,54015921	26,8266206	1,25931774	1,51272863	0,5971532	0,30725613	0,5799513	0,23660847	Unpaired t-te	0,497984438	permutation FDR (250 perm	1
1900	Maoa~Q641	Maoa	ko vs wt	ko	wt	28,998389	0,21776052	29,2486224	0,02616515	0,8407604	-0,2502334	-0,2220195	0,13977311	0,85457636	Unpaired t-te	0,615639906	permutation FDR (250 perm	1
1901	Maob~Q8BW	Maob	ko vs wt	ko	wt	29,4261254	0,61252495	29,7007858	0,38140065	0,82664485	-0,2746605	-0,1901104	0,56400712	0,24871541	Unpaired t-te	0,66019674	permutation FDR (250 perm	1
1902	Map1a~Q9Q	Map1a	ko vs wt	ko	wt	29,9547381	0,43755797	30,6878338	0,17502405	0,6016116	-0,7330957	-0,5721452	0,05621902	1,25011677	Unpaired t-te	0,210109339	permutation FDR (250 perm	1
1903	Map1b~P148	Map1b	ko vs wt	ko	wt	32,46075	0,35966915	32,743572	0,1059579	0,82198163	-0,2828219	-0,2316773	0,27386666	0,56246083	Unpaired t-te	0,594140476	permutation FDR (250 perm	1
1904	Map1lc3a~Q	Map1lc3a	ko vs wt	ko	wt	27,7824759	0,28894583	27,7630181	0,03619573	1,0135785	0,01945783	0,01664806	0,91519628	0,03848575	Unpaired t-te	0,971310453	permutation FDR (250 perm	1
1905	Map1lc3b~Q	Map1lc3b	ko vs wt	ko	wt	28,4045519	0,25060865	28,3879177	0,05379017	1,01159665	0,01663416	0,01446946	0,91770573	0,03729656	Unpaired t-te	0,975004449	permutation FDR (250 perm	1
1906	Map1s~Q8C	Map1s	ko vs wt	ko	wt	27,7557901	0,53984294	28,3015947	1,04712101	0,68500928	-0,5458046	-0,3026611	0,55026053	0,25943164	Unpaired t-te	0,50094403	permutation FDR (250 perm	1
1907	Map2k1~P31	Map2k1	ko vs wt	ko	wt	28,5668538	0,21822647	29,1576911	0,33066279	0,66395746	-0,5908373	-0,4668437	0,10823817	0,96561956	Unpaired t-te	0,305650927	permutation FDR (250 perm	1
1908	Map2k2~Q63	Map2k2	ko vs wt	ko	wt	27,6324057	0,82994677	27,6449976	0,74131946	0,99130991	-0,0125919	-0,0073629	0,98659733	0,00586006	Unpaired t-te	0,986647902	permutation FDR (250 perm	1
1909	Map2k3~O09	Map2k3	ko vs wt	ko	wt	27,3143769	0,45043674	26,3773158	0,21978872	1,91462394	0,93706105	0,7191788	0,02991191	1,52415588	Unpaired t-te	0,129111207	permutation FDR (250 perm	1
1910	Map2k4~P47	Map2k4	ko vs wt	ko	wt	27,588908	0,76939262	28,0394632	0,62743929	0,73176119	-0,4505552	-0,2767839	0,50635423	0,29554556	Unpaired t-te	0,52034349	permutation FDR (250 perm	1
1911	Map2k7~Q8	Map2k7	ko vs wt	ko	wt	26,5651939	0,33960916	25,4648492	0,94052956	2,14405924	1,10034477	0,64980116	0,23483337	0,62924019	Unpaired t-te	0,18615697	permutation FDR (250 perm	1
1912	Map3k20~Q	Map3k20	ko vs wt	ko	wt	29,5839181	0,29771146	29,5177073	0,63953961	1,04696325	0,0662108	0,04462286	0,90109228	0,04523073	Unpaired t-te	0,925986133	permutation FDR (250 perm	1
1913	Map3k2~Q61	Map3k2	ko vs wt	ko	wt	27,6476448	0,28300802	24,1641546	0,26584829	11,1849761	3,48349027	2,78886876	7,41E-05	4,13018839	Unpaired t-te	0,001	permutation FDR (250 perm	1
1914	Map3k4~O08	Map3k4	ko vs wt	ko	wt	24,5157547	0,71252804	24,0493387	0,31832273	1,38167276	0,46641596	0,31752057	0,37071593	0,43095876	Unpaired t-te	0,463222293	permutation FDR (250 perm	1
1915	Map3k6~Q9	Map3k6	ko vs wt	ko	wt	25,6541594	0,32977661	26,2229866	0,25701107	0,67416461	-0,5688272	-0,4503038	0,08441141	1,07359887	Unpaired t-te	0,307020979	permutation FDR (250 perm	1
1916	Map3k7~Q62	Map3k7	ko vs wt	ko	wt	24,6068172	0,49468397	23,9988193	0,41378008	1,52414264	0,60799793	0,43154892	0,20014128	0,69866332	Unpaired t-te	0,326668503	permutation FDR (250 perm	1
1917	Map4k3~Q9	Map4k3	ko vs wt	ko	wt	25,0196196	1,30201285	23,8778878	0,3233192	2,20645727	1,14173181	0,63936984	0,22878041	0,64058117	Unpaired t-te	0,175018658	permutation FDR (250 perm	1

1918	Map4k4~A0A	Map4k4	ko vs wt	ko	wt	29,2618594	0,54470905	28,8324222	0,36428936	1,34670809	0,42943717	0,30531959	0,33913959	0,46962151	Unpaired t-te	0,47847703	permutation FDR (250 perm	1
1919	Map4k4~F8V	Map4k4	ko vs wt	ko	wt	23,9781577	0,85013889	23,5440551	0,47811126	1,35107019	0,43410263	0,27199538	0,49984847	0,30116164	Unpaired t-te	0,526542331	permutation FDR (250 perm	1
1920	Map4k5~Q8E	Map4k5	ko vs wt	ko	wt	24,4914244	1,06441529	26,270507	0,18695487	0,29136862	-1,7790826	-1,092401	0,05976941	1,22352102	Unpaired t-te	0,039129187	permutation FDR (250 perm	1
1921	Map4~P2754	Map4	ko vs wt	ko	wt	28,2807654	0,50633467	29,3285027	1,13780379	0,48372623	-1,0477373	-0,5645099	0,3225148	0,49145036	Unpaired t-te	0,235649149	permutation FDR (250 perm	1
1922	Map6~Q7TSJ	Map6	ko vs wt	ko	wt	27,7536799	3,03411503	24,1027874	0,64610001	12,5611139	3,6508925	1,29909158	0,12630429	0,89858189	Unpaired t-te	0,018703488	permutation FDR (250 perm	1
1923	Map7d1~A2A	Map7d1	ko vs wt	ko	wt	27,0870423	0,60749576	28,1852176	0,59667023	0,46710692	-1,0981753	-0,7091145	0,11040085	0,95702759	Unpaired t-te	0,135286385	permutation FDR (250 perm	1
1924	Mapk14~P47	Mapk14	ko vs wt	ko	wt	25,294541	1,39111428	24,8297837	0,96571308	1,38008523	0,46475737	0,22624584	0,67775415	0,16892782	Unpaired t-te	0,597019631	permutation FDR (250 perm	1
1925	Mapk1~P630	Mapk1	ko vs wt	ko	wt	31,0545437	0,08712724	30,5272935	0,12365463	1,44117964	0,52725018	0,47893768	0,01067782	1,97151739	Unpaired t-te	0,291572433	permutation FDR (250 perm	1
1926	Mapk3~Q63E	Mapk3	ko vs wt	ko	wt	29,0130816	0,06677455	28,7714265	0,29549303	1,18234831	0,2416551	0,19930778	0,36681119	0,43555742	Unpaired t-te	0,666590764	permutation FDR (250 perm	1
1927	Mapk9~Q9W	Mapk9	ko vs wt	ko	wt	24,3498799	1,26374963	24,1346167	0,75316855	1,16091566	0,21526317	0,11309882	0,82120899	0,0855463	Unpaired t-te	0,800523	permutation FDR (250 perm	1
1928	Mapkap1~Q8	Mapkap1	ko vs wt	ko	wt	24,5068863	0,68602195	23,8800176	0,3369353	1,54420975	0,62686872	0,4287132	0,23714516	0,62498574	Unpaired t-te	0,330247423	permutation FDR (250 perm	1
1929	Mapkapk2~P	Mapkapk2	ko vs wt	ko	wt	24,7548254	1,02296083	25,5305373	1,19314202	0,5841003	-0,775712	-0,3821506	0,49498817	0,30540518	Unpaired t-te	0,388330568	permutation FDR (250 perm	1
1930	Mapkapk3~C	Mapkapk3	ko vs wt	ko	wt	24,2864643	0,70335581	24,9510733	0,83449639	0,63085965	-0,664609	-0,3872321	0,40887798	0,38840628	Unpaired t-te	0,38345982	permutation FDR (250 perm	1
1931	Mapre1~Q61	Mapre1	ko vs wt	ko	wt	31,0774725	0,13952081	30,9923208	0,12915467	1,06079927	0,08515169	0,075908	0,51882906	0,2849757	Unpaired t-te	0,869657392	permutation FDR (250 perm	1
1932	Mapre2~Q8F	Mapre2	ko vs wt	ko	wt	28,0251628	1,0403351	28,0940531	0,41155665	0,95337106	-0,0688903	-0,0413154	0,92250575	0,03503092	Unpaired t-te	0,928348243	permutation FDR (250 perm	1
1933	Mapre3~Q6F	Mapre3	ko vs wt	ko	wt	29,7434054	0,35585027	29,6860564	0,42357076	1,04055198	0,05734903	0,04206935	0,88263247	0,0542201	Unpaired t-te	0,927966969	permutation FDR (250 perm	1
1934	Marf1~Q8BJ	Marf1	ko vs wt	ko	wt	23,9757658	0,89709864	23,8079785	0,08623678	1,12333426	0,16778728	0,11027628	0,76824367	0,11450101	Unpaired t-te	0,810880133	permutation FDR (250 perm	1
1935	Mark1~Q8VH	Mark1	ko vs wt	ko	wt	29,5802047	0,36239419	29,8941941	0,19530291	0,80441428	-0,3139894	-0,2510519	0,26780767	0,57217698	Unpaired t-te	0,558592861	permutation FDR (250 perm	1
1936	Mark2~Q055	Mark2	ko vs wt	ko	wt	26,6683994	0,40159063	27,1788936	0,49122221	0,70198194	-0,5104942	-0,360106	0,29383524	0,53189612	Unpaired t-te	0,415413107	permutation FDR (250 perm	1
1937	Mars1~Q68F	Mars1	ko vs wt	ko	wt	29,5435492	0,29921671	29,7870027	0,51771234	0,8447208	-0,2434535	-0,1733022	0,59138761	0,22812778	Unpaired t-te	0,700096358	permutation FDR (250 perm	1
1938	Masp1~P980	Masp1	ko vs wt	ko	wt	23,8730758	0,47031549	24,6054455	0,20190341	0,60191443	-0,7323697	-0,5604379	0,06974601	1,15648064	Unpaired t-te	0,216617935	permutation FDR (250 perm	1
1939	Masp2~Q91V	Masp2	ko vs wt	ko	wt	29,4145096	0,38515049	28,4297439	1,08368991	1,9789919	0,98476571	0,54773214	0,32663721	0,48593434	Unpaired t-te	0,25201312	permutation FDR (250 perm	1
1940	Mat2b~Q99L	Mat2b	ko vs wt	ko	wt	25,0290775	1,11933997	24,6675833	0,6515089	1,28475583	0,36149419	0,20154165	0,66816064	0,17511911	Unpaired t-te	0,640146413	permutation FDR (250 perm	1
1941	Matn2~O087	Matn2	ko vs wt	ko	wt	30,7792712	0,34978275	30,1236998	0,59229678	1,57523976	0,65557144	0,4475004	0,25531655	0,59292103	Unpaired t-te	0,327599312	permutation FDR (250 perm	1
1942	Matn4~F2Z4	Matn4	ko vs wt	ko	wt	25,4203131	1,1221495	24,3108153	0,29826253	2,15770528	1,10949782	0,65989021	0,18641791	0,72951237	Unpaired t-te	0,163559692	permutation FDR (250 perm	1
1943	Matr3~Q8K3	Matr3	ko vs wt	ko	wt	30,7610446	0,58410992	30,361576	0,034372	1,31902195	0,39946857	0,29853178	0,32175461	0,49247522	Unpaired t-te	0,502729251	permutation FDR (250 perm	1
1944	Mavs~Q8VCF	Mavs	ko vs wt	ko	wt	23,8464312	1,01246978	25,0155981	1,09661619	0,44467804	-1,1691669	-0,5931628	0,29424175	0,5312957	Unpaired t-te	0,197896685	permutation FDR (250 perm	1
1945	Mbd2~Q9Z2E	Mbd2	ko vs wt	ko	wt	25,7693899	0,29452419	24,9472044	0,19378397	1,76808242	0,82218553	0,67481721	0,01309554	1,88287647	Unpaired t-te	0,147185031	permutation FDR (250 perm	1
1946	Mbl1~P3903	Mbl1	ko vs wt	ko	wt	31,9792971	0,38732384	31,5768114	0,1541634	1,32178339	0,40248577	0,32230408	0,17702705	0,75196037	Unpaired t-te	0,458523976	permutation FDR (250 perm	1
1947	Mbnl1~Q9JK	Mbnl1	ko vs wt	ko	wt	29,7430613	0,518257	30,3834325	0,2215982	0,6415479	-0,6403711	-0,4786885	0,12468257	0,90419424	Unpaired t-te	0,281418259	permutation FDR (250 perm	1
1948	Mbnl2~Q8C1	Mbnl2	ko vs wt	ko	wt	25,4632273	1,19878812	25,4524164	0,8706116	1,00752172	0,01081094	0,00561231	0,99114244	0,00386393	Unpaired t-te	0,989580838	permutation FDR (250 perm	1
1949	Mb~P04247	Mb	ko vs wt	ko	wt	28,5503099	1,24593055	28,5900881	0,32943567	0,97280447	-0,0397782	-0,0226513	0,9608292	0,01735381	Unpaired t-te	0,960604786	permutation FDR (250 perm	1
1950	Mcam~Q8R2	Mcam	ko vs wt	ko	wt	30,1894843	0,77110015	30,2751381	0,19557183	0,94235739	-0,0856538	-0,0584197	0,86420939	0,06338102	Unpaired t-te	0,900340334	permutation FDR (250 perm	1
1951	Mcat~Q8R3F	Mcat	ko vs wt	ko	wt	24,3670351	0,64103671	24,4455801	0,23530425	0,94701222	-0,078545	-0,0558728	0,85576244	0,06764678	Unpaired t-te	0,90361828	permutation FDR (250 perm	1
1952	Mccc1~Q99M	Mccc1	ko vs wt	ko	wt	29,6524358	0,30845484	29,8864962	0,1768365	0,85023858	-0,2340604	-0,1922307	0,33216977	0,47863989	Unpaired t-te	0,656742424	permutation FDR (250 perm	1
1953	Mccc2~Q3UL	Mccc2	ko vs wt	ko	wt	28,0916793	0,70225965	27,6837177	0,16767479	1,32680981	0,40796158	0,28680612	0,39610809	0,40218628	Unpaired t-te	0,514302469	permutation FDR (250 perm	1
1954	Mcf2l~Q640	Mcf2l	ko vs wt	ko	wt	24,5856147	0,34019457	23,7718805	0,39410032	1,75775534	0,81373428	0,60684208	0,07798567	1,1079852	Unpaired t-te	0,190907537	permutation FDR (250 perm	1
1955	Mcm5~P497	Mcm5	ko vs wt	ko	wt	25,5616556	0,97956929	24,7030027	0,79518115	1,81334438	0,85865294	0,47769249	0,33262519	0,47804486	Unpaired t-te	0,27977305	permutation FDR (250 perm	1
1956	Mcm7~Q618	Mcm7	ko vs wt	ko	wt	24,9416396	1,02911162	24,2719486	0,95658993	1,59073224	0,66969101	0,3524117	0,49399681	0,30627585	Unpaired t-te	0,419240638	permutation FDR (250 perm	1
1957	Mcpt4~Q3U	Mcpt4	ko vs wt	ko	wt	29,9996429	0,28510782	29,9421319	0,81571353	1,04066879	0,05751098	0,0359483	0,93124455	0,03093626	Unpaired t-te	0,940696713	permutation FDR (250 perm	1
1958	Mcrs1~Q99L	Mcrs1	ko vs wt	ko	wt	24,9871633	0,3790302	24,6744745	0,58813933	1,24202035	0,31268881	0,21272242	0,55194425	0,25810479	Unpaired t-te	0,63221164	permutation FDR (250 perm	1
1959	Mcts1~Q9DB	Mcts1	ko vs wt	ko	wt	28,8024329	0,07280281	28,2500741	0,28159201	1,46648143	0,55235881	0,45895894	0,10322326	0,98622241	Unpaired t-te	0,331513569	permutation FDR (250 perm	1
1960	Mcur1~Q9CX	Mcur1	ko vs wt	ko	wt	27,7075909	0,41754684	27,5992382	0,09534144	1,07799665	0,10835269	0,08665997	0,69073724	0,16068713	Unpaired t-te	0,852071233	permutation FDR (250 perm	1
1961	Mcu~Q3UMF	Mcu	ko vs wt	ko	wt	25,5797247	2,45858845	25,0623751	1,37822476	1,4313233	0,51734958	0,19007541	0,77621939	0,11001551	Unpaired t-te	0,660724098	permutation FDR (250 perm	1
1962	Mdh1~P1415	Mdh1	ko vs wt	ko	wt	29,9830794	0,45673789	29,7339248	0,18000297	1,18851045	0,24915459	0,19272345	0,44043989	0,35611336	Unpaired t-te	0,658600291	permutation FDR (250 perm	1
1963	Mdh2~P0824	Mdh2	ko vs wt	ko	wt	32,2983005	0,16393323	31,9557819	0,24765766	1,26796819	0,34251855	0,28565571	0,17916058	0,74675754	Unpaired t-te	0,519151217	permutation FDR (250 perm	1
1964	Me1~P06801	Me1	ko vs wt	ko	wt	28,8378899	0,42470064	28,3621118	0,70036024	1,39066806	0,47577811	0,30643798	0,45292997	0,34396895	Unpaired t-te	0,491915106	permutation FDR (250 perm	1
1965	Mecp2~Q9Z2	Mecp2	ko vs wt	ko	wt	28,2781502	0,94765175	28,4156241	1,1189743	0,90910959	-0,1374739	-0,0700691	0,89363242	0,04884108	Unpaired t-te	0,881838043	permutation FDR (250 perm	1
1966	Mecr~Q9DCS	Mecr	ko vs wt	ko	wt	28,8706352	0,54690677	28,8946021	0,28778487	0,98352465	-0,0239669	-0,0174222	0,95171308	0,02149396	Unpaired t-te	0,969499082	permutation FDR (250 perm	1
1967	Med15~Q92	Med15	ko vs wt	ko	wt	24,384826	0,34614696	24,7175357	0,98911582	0,79404364	-0,3327098	-0,192607	0,68656393	0,16331902	Unpaired t-te	0,675355549	permutation FDR (250 perm	1
1968	Megf6~Q80V	Megf6	ko vs wt	ko	wt	25,9569904	2,009584	27,8953333	0,6314355	0,26091597	-1,9383428	-0,8641047	0,1971043	0,70530389	Unpaired t-te	0,081630424	permutation FDR (250 perm	1
1969	Meis2~P973	Meis2	ko vs wt	ko	wt	23,7474454	0,57058435	24,4886095	0,37692873	0,59825645	-0,7411641	-0,5205736	0,14069365	0,85172552	Unpaired t-te	0,24375	permutation FDR (250 perm	1
1970	Meltf~Q9R0	Meltf	ko vs wt	ko	wt	25,1087899	1,03045257	23,1854916	0,9740703	3,7928922	1,92329837	1,0068899	0,095159	1,02155013	Unpaired t-te	0,045917872	permutation FDR (250 perm	1
1971	Memo1~Q91	Memo1	ko vs wt	ko	wt	27,7781224	0,20165705	25,575415	1,81774132	4,60342412	2,20270736	0,96162922	0,22797549	0,64211184	Unpaired t-te	0,080606477	permutation FDR (250 perm	1
1972	Mesd~Q9ERI	Mesd	ko vs wt	ko	wt	24,3309848	0,81539742	24,9049246	0,87696016	0,67177973	-0,5739398	-0,3226994	0,50126469	0,29993289	Unpaired t-te	0,459259447	permutation FDR (250 perm	1
1973	Metap1~Q8E	Metap1	ko vs wt	ko	wt	28,2447493	0,3526829	28,5687077	0,24998469	0,79887499	-0,3239583	-0,2551568	0,2835703	0,54733926	Unpaired t-te	0,551961165	permutation FDR (250 perm	1
1974	Metap2~O08	Metap2	ko vs wt	ko	wt	28,5783217	0,34686283	28,4279894	0,13629278	1,10982504	0,15033225	0,12299691	0,53410402	0,27237415	Unpaired t-te	0,783119687	permutation FDR (250 perm	1
1975	Mettl7a1~Q8	Mettl7a1	ko vs wt	ko	wt	27,3311127	0,67252355	26,9825452	0,25447632	1,27329573	0,34856753	0,24410335	0,45961224	0,33760841	Unpaired t-te	0,573005418	permutation FDR (250 perm	1
1976	Mfap2~Q99F	Mfap2	ko vs wt	ko	wt	32,9365911	0,66550888	33,2861262	0,08662006	0,78483696	-0,3495351	-0,2516301	0,43229787	0,36421691	Unpaired t-te	0,568703045	permutation FDR (250 perm	1
1977	Mfap4~Q9D1	Mfap4	ko vs wt	ko	wt	31,8345589	1,18167933	32,876242	0,19501617	0,48576044	-1,0416831	-0,6141856	0,22478651	0,64822975	Unpaired t-te	0,192678045	permutation FDR (250 perm	1
1978	Mfap5~F8W	Mfap5	ko vs wt	ko	wt	32,8328317	0,87311559	33,489988	0,50559241	0,634127	-0,6571563	-0,4061538	0,33706934	0,47228074	Unpaired t-te	0,353811525	permutation FDR (250 perm	1
1979	Mfge8~P219	Mfge8	ko vs wt	ko	wt	34,2781345	0,38723553	34,3257817	0,5143562	0,96751289	-0,0476472	-0,0333915	0,91729169	0,03749254	Unpaired t-te	0,942936159	permutation FDR (250 perm	1
1980	Mfn2~Q80U	Mfn2	ko vs wt	ko	wt	27,1501121	0,15229124	27,2633571	0,28771887	0,92450626	-0,113245	-0,0926996	0,64751216	0,18875207	Unpaired t-te	0,843972543	permutation FDR (250 perm	1
1981	Mgll~O3567	Mgll	ko vs wt	ko	wt	29,0713131	0,54208162	28,5209357	0,76235436	1,46446866	0,55037732	0,33904173	0,43646489	0,36005069	Unpaired t-te	0,44449936	permutation FDR (250 perm	1

1982	Mgp~P19788	Mgp	ko vs wt	ko	wt	30,7332594	0,64296714	31,0966541	1,17300456	0,77733331	-0,3633948	-0,1903866	0,71770623	0,14405329	Unpaired t-te	0,673363109	permutation FDR (250 perm	1
1983	Mgst1~Q91V	Mgst1	ko vs wt	ko	wt	27,9669131	0,44757013	29,1651971	0,16389429	0,43579333	-1,198284	-0,9338229	0,01280218	1,89271617	Unpaired t-te	0,062136292	permutation FDR (250 perm	1
1984	Mia3~Q8BI84	Mia3	ko vs wt	ko	wt	24,8069087	0,4918621	25,0156354	1,3272988	0,86530055	-0,2087268	-0,1053876	0,84837709	0,07141107	Unpaired t-te	0,823277548	permutation FDR (250 perm	1
1985	Mib1~Q80SY	Mib1	ko vs wt	ko	wt	24,1552286	0,4967774	23,932375	0,33914167	1,16703969	0,22285363	0,16220988	0,57708741	0,2387584	Unpaired t-te	0,709113591	permutation FDR (250 perm	1
1986	Mical1~Q8VI	Mical1	ko vs wt	ko	wt	29,8610057	0,49457862	29,5311059	0,24492644	1,25692602	0,32989974	0,24730823	0,37164967	0,42986625	Unpaired t-te	0,565006942	permutation FDR (250 perm	1
1987	Mical2~Q8BI	Mical2	ko vs wt	ko	wt	29,3354604	0,24760111	29,1734528	0,42203074	1,11884303	0,16200765	0,1217285	0,6588203	0,18123303	Unpaired t-te	0,791461753	permutation FDR (250 perm	1
1988	Mical3~Q8CJ	Mical3	ko vs wt	ko	wt	25,1888227	1,46695558	26,611699	0,25594835	0,37296797	-1,4228764	-0,7624996	0,19140006	0,71805793	Unpaired t-te	0,121968226	permutation FDR (250 perm	1
1989	Micos13~Q8	Micos13	ko vs wt	ko	wt	25,4330052	1,84736008	27,4992782	0,23222081	0,23877554	-2,066273	-0,9938114	0,14727111	0,83188244	Unpaired t-te	0,057051715	permutation FDR (250 perm	1
1990	Micu1~Q8VC	Micu1	ko vs wt	ko	wt	27,0760335	0,35350884	24,6294845	1,35793621	5,45110618	2,44654902	1,23459728	0,11988231	0,92124488	Unpaired t-te	0,03461	permutation FDR (250 perm	1
1991	Mif~P34884	Mif	ko vs wt	ko	wt	25,4514637	1,74280789	25,4924674	1,81136305	0,97197849	-0,0410037	-0,0155979	0,98107461	0,00829796	Unpaired t-te	0,972190576	permutation FDR (250 perm	1
1992	Mink1~Q9JM	Mink1	ko vs wt	ko	wt	25,0708359	0,73454976	24,175451	0,97405587	1,86010606	0,89538488	0,49500084	0,33905517	0,46972962	Unpaired t-te	0,275189747	permutation FDR (250 perm	1
1993	Mios~Q8VE1	Mios	ko vs wt	ko	wt	26,3063958	0,31551164	25,9661114	0,21588663	1,26600611	0,34028437	0,27493996	0,21166966	0,67434139	Unpaired t-te	0,52156976	permutation FDR (250 perm	1
1994	Mipep~A6H6	Mipep	ko vs wt	ko	wt	25,4412726	1,15553583	25,1983068	0,20407906	1,18342289	0,2429657	0,14440091	0,74357644	0,12867438	Unpaired t-te	0,747507762	permutation FDR (250 perm	1
1995	Mkrn2~Q9ER	Mkrn2	ko vs wt	ko	wt	24,8833057	1,37832673	24,706876	0,81032083	1,1300838	0,17642975	0,08907906	0,86437657	0,06329702	Unpaired t-te	0,844104338	permutation FDR (250 perm	1
1996	Mlec~Q6ZQI	Mlec	ko vs wt	ko	wt	28,2548952	0,81045953	28,3986883	0,27168492	0,9051363	-0,143793	-0,0954915	0,79054907	0,10207117	Unpaired t-te	0,834819058	permutation FDR (250 perm	1
1997	Mlycd~Q99J3	Mlycd	ko vs wt	ko	wt	25,6724349	0,82853608	26,6208405	0,19473937	0,51820486	-0,9484056	-0,6332072	0,13998361	0,8539228	Unpaired t-te	0,178345154	permutation FDR (250 perm	1
1998	Mmaa~Q8C7	Mmaa	ko vs wt	ko	wt	24,987869	0,54109555	24,5505147	0,38751055	1,35411882	0,43735434	0,3089655	0,34098831	0,46726051	Unpaired t-te	0,472533249	permutation FDR (250 perm	1
1999	Mmp12~P34	Mmp12	ko vs wt	ko	wt	31,1052928	0,49199624	29,5113104	0,7170249	3,01881501	1,59398235	1,00810827	0,06534228	1,18480569	Unpaired t-te	0,053560488	permutation FDR (250 perm	1
2000	Mmp13~P33	Mmp13	ko vs wt	ko	wt	26,9985116	0,34593347	25,09839	1,99001015	3,73244655	1,9001216	0,78476788	0,30864761	0,51053708	Unpaired t-te	0,133695615	permutation FDR (250 perm	1
2001	Mmp14~P53	Mmp14	ko vs wt	ko	wt	27,8202634	0,85488392	27,5710996	0,21702137	1,18851808	0,24916385	0,16426148	0,65789419	0,18184395	Unpaired t-te	0,71143952	permutation FDR (250 perm	1
2002	Mmp17~Q9R	Mmp17	ko vs wt	ko	wt	24,8386691	0,46852455	24,8541096	0,62626332	0,98935455	-0,0154405	-0,0101655	0,97790164	0,00970483	Unpaired t-te	0,983308255	permutation FDR (250 perm	1
2003	Mmp19~Q9J	Mmp19	ko vs wt	ko	wt	30,8189826	1,00669418	28,471599	1,46232618	5,08900503	2,34738362	1,07374027	0,13518701	0,86906503	Unpaired t-te	0,042044864	permutation FDR (250 perm	1
2004	Mmp23~O88	Mmp23	ko vs wt	ko	wt	24,4534049	1,19808964	24,6673268	1,31755045	0,86219021	-0,2139219	-0,0990213	0,86269778	0,06414132	Unpaired t-te	0,82761269	permutation FDR (250 perm	1
2005	Mmp2~P334	Mmp2	ko vs wt	ko	wt	30,9070777	0,64347119	29,6461759	1,04762262	2,39645505	1,26090188	0,68950012	0,22531343	0,64721291	Unpaired t-te	0,155473986	permutation FDR (250 perm	1
2006	Mmp3~P288	Mmp3	ko vs wt	ko	wt	31,2542177	1,13582968	30,0418246	0,96781471	2,31721698	1,21239314	0,62243487	0,26022597	0,58464936	Unpaired t-te	0,175816019	permutation FDR (250 perm	1
2007	Mmp9~P412	Mmp9	ko vs wt	ko	wt	25,7670693	1,72244183	24,2960534	0,41199865	2,77217038	1,47101593	0,72241546	0,23837685	0,62273592	Unpaired t-te	0,136003407	permutation FDR (250 perm	1
2008	Mmrn1~B2R	Mmrn1	ko vs wt	ko	wt	26,8177029	3,37038438	24,1686967	0,37698686	6,27235066	2,64900622	0,8937068	0,26717679	0,57320127	Unpaired t-te	0,080029499	permutation FDR (250 perm	1
2009	Mmrn2~A6H	Mmrn2	ko vs wt	ko	wt	27,9097916	0,25341102	27,514706	0,2896664	1,31502084	0,39508567	0,31563619	0,19363915	0,71300683	Unpaired t-te	0,470151027	permutation FDR (250 perm	1
2010	Mms19~Q9D	Mms19	ko vs wt	ko	wt	24,5525997	0,59835247	24,2998914	0,85697368	1,19144166	0,25270831	0,14886851	0,7392336	0,1312183	Unpaired t-te	0,739484163	permutation FDR (250 perm	1
2011	Mmut~P1633	Mmut	ko vs wt	ko	wt	26,1102819	0,81256304	27,1449203	0,23261883	0,48813821	-1,0346384	-0,6910795	0,11152707	0,9526197	Unpaired t-te	0,14761794	permutation FDR (250 perm	1
2012	Mnda~Q0861	Mnda	ko vs wt	ko	wt	27,713835	0,48907446	26,6391707	0,68208495	2,1062319	1,07466429	0,68938058	0,14068867	0,85174086	Unpaired t-te	0,15157296	permutation FDR (250 perm	1
2013	Mob1a~Q921	Mob1a	ko vs wt	ko	wt	27,047063	0,82096731	23,8953272	0,18768403	8,88724173	3,15173573	2,1121198	0,00495805	2,30468947	Unpaired t-te	0,003488288	permutation FDR (250 perm	1
2014	Mob2~Q8VI6	Mob2	ko vs wt	ko	wt	24,8932064	1,38449902	23,8248069	0,62694466	2,09710555	1,06839948	0,55818974	0,30072035	0,52183718	Unpaired t-te	0,217550621	permutation FDR (250 perm	1
2015	Mogs~Q80UI	Mogs	ko vs wt	ko	wt	27,5872221	0,36382326	24,4056872	0,33586804	9,07271863	3,18153492	2,41563666	0,00029643	3,52808199	Unpaired t-te	0,002209524	permutation FDR (250 perm	1
2016	Mon2~B9EKJ	Mon2	ko vs wt	ko	wt	27,7814718	0,38277548	27,7887543	0,25543234	0,9949649	-0,0072825	-0,0056655	0,98063093	0,00849441	Unpaired t-te	0,989397553	permutation FDR (250 perm	1
2017	Morc3~F7BJI	Morc3	ko vs wt	ko	wt	24,5325316	0,22955274	23,9261928	1,14108429	1,52239082	0,60633877	0,33357829	0,53214792	0,27396763	Unpaired t-te	0,470895202	permutation FDR (250 perm	1
2018	Mpc1~P6303	Mpc1	ko vs wt	ko	wt	24,4870696	1,2823351	25,5920048	1,53727403	0,46492336	-1,1049352	-0,477254	0,45107506	0,34575118	Unpaired t-te	0,28824533	permutation FDR (250 perm	1
2019	Mpc2~Q9D0	Mpc2	ko vs wt	ko	wt	29,5089774	0,62728172	29,2771785	0,13126143	1,1742983	0,23179894	0,16872023	0,57451494	0,24069868	Unpaired t-te	0,704350616	permutation FDR (250 perm	1
2020	Mpeg1~E9QI	Mpeg1	ko vs wt	ko	wt	24,6972406	1,46126246	24,375862	0,29573647	1,24952396	0,32137857	0,1719346	0,73366231	0,13450379	Unpaired t-te	0,698919105	permutation FDR (250 perm	1
2021	Mpo~P11247	Mpo	ko vs wt	ko	wt	26,8509619	1,26255233	24,9575891	0,47456495	3,71502724	1,89337279	1,05043453	0,07569387	1,1209393	Unpaired t-te	0,040145238	permutation FDR (250 perm	1
2022	Mpp1~P7029	Mpp1	ko vs wt	ko	wt	29,2797251	0,51650581	28,807376	0,3704277	1,38736663	0,47234909	0,33813948	0,28770049	0,5410594	Unpaired t-te	0,434391234	permutation FDR (250 perm	1
2023	Mpp7~G5E8	Mpp7	ko vs wt	ko	wt	26,9039459	0,38417802	27,0310741	0,14002146	0,91565233	-0,1271282	-0,1022835	0,62790411	0,20210667	Unpaired t-te	0,821072119	permutation FDR (250 perm	1
2024	Mprip~P9743	Mprip	ko vs wt	ko	wt	26,2077821	1,21923657	24,0228436	0,65152564	4,54707391	2,18493845	1,18663976	0,05035873	1,29792522	Unpaired t-te	0,022207088	permutation FDR (250 perm	1
2025	Mprip~Q5SW	Mprip	ko vs wt	ko	wt	30,1242228	0,16571655	30,5207938	0,35069361	0,75966173	-0,396571	-0,3132979	0,24576367	0,60948232	Unpaired t-te	0,488265415	permutation FDR (250 perm	1
2026	Mpst~Q99J9	Mpst	ko vs wt	ko	wt	25,6866601	1,48017499	23,3537424	0,57270329	5,03823244	2,33291768	1,19902764	0,06644154	1,17756031	Unpaired t-te	0,023016575	permutation FDR (250 perm	1
2027	Mpz~E9QK82	Mpz	ko vs wt	ko	wt	28,831111	1,27761801	30,2570994	1,50365623	0,37216429	-1,4259885	-0,6216001	0,33531358	0,47454886	Unpaired t-te	0,18205257	permutation FDR (250 perm	1
2028	Mrc1~Q6183	Mrc1	ko vs wt	ko	wt	30,3464049	0,56751144	29,8628805	0,32300422	1,39815505	0,48352436	0,34552203	0,28126936	0,55087757	Unpaired t-te	0,426295359	permutation FDR (250 perm	1
2029	Mrc2~Q6444	Mrc2	ko vs wt	ko	wt	27,4800347	1,21100343	24,2568165	2,14294318	9,33867706	3,22321819	1,20773352	0,15357321	0,81368453	Unpaired t-te	0,029611979	permutation FDR (250 perm	1
2030	Mre11~Q612	Mre11	ko vs wt	ko	wt	24,8534429	0,6622098	25,8295853	0,02590492	0,50833716	-0,9761424	-0,7059349	0,08354524	1,07807828	Unpaired t-te	0,14813444	permutation FDR (250 perm	1
2031	Mroh1~E0CZ	Mroh1	ko vs wt	ko	wt	26,5796946	0,45350695	26,3501929	0,08460517	1,17242989	0,22950166	0,18091225	0,4502643	0,34653248	Unpaired t-te	0,68367128	permutation FDR (250 perm	1
2032	Mrpl10~Q3TI	Mrpl10	ko vs wt	ko	wt	25,1614356	0,53616534	24,1737689	0,64507285	1,98297533	0,98766673	0,63668814	0,15267059	0,81624462	Unpaired t-te	0,174693683	permutation FDR (250 perm	1
2033	Mrpl11~Q9C	Mrpl11	ko vs wt	ko	wt	25,4811694	1,3793709	24,6770267	0,63092595	1,74610792	0,80414273	0,42039467	0,42278967	0,37387564	Unpaired t-te	0,340162679	permutation FDR (250 perm	1
2034	Mrpl13~Q9D	Mrpl13	ko vs wt	ko	wt	24,6276647	1,30455576	23,9600949	0,68244118	1,58839508	0,6675698	0,35237034	0,49064887	0,3092292	Unpaired t-te	0,417538762	permutation FDR (250 perm	1
2035	Mrpl14~Q9D	Mrpl14	ko vs wt	ko	wt	25,1242954	0,55530222	24,8675315	0,18612922	1,19479572	0,25676398	0,19068039	0,50059927	0,30050979	Unpaired t-te	0,663546643	permutation FDR (250 perm	1
2036	Mrpl15~Q9C	Mrpl15	ko vs wt	ko	wt	25,563283	0,76935054	24,9989995	0,81835514	1,47865301	0,56428354	0,32627203	0,48137283	0,31751843	Unpaired t-te	0,453835084	permutation FDR (250 perm	1
2037	Mrpl16~Q99	Mrpl16	ko vs wt	ko	wt	26,9944645	0,07657678	24,9169595	1,19804205	4,22076658	2,07750505	1,12401067	0,13333981	0,87504017	Unpaired t-te	0,05085233	permutation FDR (250 perm	1
2038	Mrpl22~Q8B	Mrpl22	ko vs wt	ko	wt	24,1903444	0,31234857	25,201839	0,8815561	0,49603211	-1,0114946	-0,6134303	0,24152605	0,61703602	Unpaired t-te	0,208277439	permutation FDR (250 perm	1
2039	Mrpl3~Q99N	Mrpl3	ko vs wt	ko	wt	25,7428814	2,04256849	24,6819089	0,05170852	2,08633746	1,06097253	0,48671944	0,43467834	0,361832	Unpaired t-te	0,288287526	permutation FDR (250 perm	1
2040	Mrpl46~Q9E	Mrpl46	ko vs wt	ko	wt	24,4963936	0,82731012	24,4487738	1,58952867	1,0335583	0,04761977	0,02143829	0,97157103	0,01252544	Unpaired t-te	0,963780289	permutation FDR (250 perm	1
2041	Mrpl48~E9Q	Mrpl48	ko vs wt	ko	wt	25,0428657	0,51929586	23,3912617	0,68172922	3,14182773	1,65160408	1,05353028	0,05145585	1,28856524	Unpaired t-te	0,043317302	permutation FDR (250 perm	1
2042	Mrps23~Q8V	Mrps23	ko vs wt	ko	wt	24,7805366	1,39990298	24,5367966	0,52225089	1,18405826	0,24374007	0,12905838	0,79708098	0,09849756	Unpaired t-te	0,771925967	permutation FDR (250 perm	1
2043	Mrps27~Q8B	Mrps27	ko vs wt	ko	wt	24,7881301	1,75181062	23,8225017	0,93104631	1,95291406	0,96562847	0,4375692	0,46143671	0,33588786	Unpaired t-te	0,320403733	permutation FDR (250 perm	1
2044	Mrps35~Q8B	Mrps35	ko vs wt	ko	wt	24,9880153	1,15287675	26,2598372	0,89488308	0,41413646	-1,2718219	-0,6629614	0,2259984	0,64589464	Unpaired t-te	0,153163995	permutation FDR (250 perm	1
2045	Mrps9~Q9D7	Mrps9	ko vs wt	ko	wt	24,3488536	1,14550054	24,0057572	0,19969761	1,26847619	0,34309644	0,20467951	0,64417605	0,19099542	Unpaired t-te	0,643734425	permutation FDR (250 perm	1

2046	Msi2~Q920C	Msi2	ko vs wt	ko	wt	27,2819412	0,13972823	27,6862975	0,77888085	0,75557332	-0,4043563	-0,2597641	0,54036035	0,26731652	Unpaired t-te	0,572192655	permutation FDR (250 perm	1
2047	Msn~P26041	Msn	ko vs wt	ko	wt	34,4017181	0,54490834	34,9388564	0,39776686	0,68913651	-0,5371383	-0,3777343	0,25947622	0,58590244	Unpaired t-te	0,385894317	permutation FDR (250 perm	1
2048	Msra~Q9D6Y	Msra	ko vs wt	ko	wt	29,1825063	0,38624597	29,1112997	0,24000921	1,05059495	0,07120655	0,05561997	0,80958398	0,0917381	Unpaired t-te	0,902895402	permutation FDR (250 perm	1
2049	Msrb1~Q9JL	Msrb1	ko vs wt	ko	wt	25,1926641	0,58317992	24,7089127	1,17778527	1,39837512	0,48375142	0,25483309	0,63198186	0,19929539	Unpaired t-te	0,569357035	permutation FDR (250 perm	1
2050	Msrb3~Q8B	Msrb3	ko vs wt	ko	wt	28,6670717	0,41574635	28,7370736	0,53432294	0,95263675	-0,0700019	-0,0483565	0,88420145	0,05344878	Unpaired t-te	0,917541589	permutation FDR (250 perm	1
2051	Mstn~O0868	Mstn	ko vs wt	ko	wt	24,3265783	0,4159215	23,9085812	0,70222467	1,33607141	0,41799712	0,26940365	0,50470732	0,2969604	Unpaired t-te	0,545054169	permutation FDR (250 perm	1
2052	Mta1~F8WH	Mta1	ko vs wt	ko	wt	27,6523044	0,22628909	27,4475733	0,36434385	1,15247157	0,20473117	0,15884627	0,52916711	0,27640716	Unpaired t-te	0,723748649	permutation FDR (250 perm	1
2053	Mta2~Q9R1	Mta2	ko vs wt	ko	wt	27,5026581	0,3144863	27,3269371	0,24626518	1,12952876	0,17572101	0,14039979	0,51679454	0,28668208	Unpaired t-te	0,748064316	permutation FDR (250 perm	1
2054	Mtap~Q9CQ	Mtap	ko vs wt	ko	wt	28,2820079	0,24548241	28,3977702	0,37281602	0,92289452	-0,1157623	-0,0890956	0,72359965	0,14050165	Unpaired t-te	0,848889986	permutation FDR (250 perm	1
2055	Mtarc2~Q92	Mtarc2	ko vs wt	ko	wt	28,4793594	0,18905765	27,9247151	0,71242819	1,46880651	0,55464436	0,36599258	0,386083	0,41331933	Unpaired t-te	0,430462831	permutation FDR (250 perm	1
2056	Mtatp8~P03	Mtatp8	ko vs wt	ko	wt	28,4412011	0,37948996	28,532515	0,30184018	0,93866753	-0,0913138	-0,0699255	0,77766625	0,10920675	Unpaired t-te	0,879979342	permutation FDR (250 perm	1
2057	Mtch1~Q791	Mtch1	ko vs wt	ko	wt	25,2452016	1,44501458	24,9128514	1,50290068	1,25906272	0,33235015	0,14136151	0,81732801	0,08760362	Unpaired t-te	0,748404963	permutation FDR (250 perm	1
2058	Mtch2~Q791	Mtch2	ko vs wt	ko	wt	30,1973259	0,64034987	30,6770025	0,5978242	0,71713833	-0,4796767	-0,3071726	0,43628771	0,36022702	Unpaired t-te	0,478556325	permutation FDR (250 perm	1
2059	Mtco2~P004	Mtco2	ko vs wt	ko	wt	28,4759215	1,3147495	27,9407832	1,1155995	1,44908098	0,53513822	0,25546682	0,64682435	0,18921364	Unpaired t-te	0,552653928	permutation FDR (250 perm	1
2060	Mterf2~Q8B	Mterf2	ko vs wt	ko	wt	24,3172225	0,86307621	24,4703861	0,49279674	0,89927632	-0,1531636	-0,0952481	0,81135561	0,09078876	Unpaired t-te	0,832997245	permutation FDR (250 perm	1
2061	Mtfr1l~Q9CV	Mtfr1l	ko vs wt	ko	wt	25,6506874	0,72737588	26,6037072	0,74180908	0,51655009	-0,9530198	-0,5700095	0,22541584	0,64701557	Unpaired t-te	0,211722622	permutation FDR (250 perm	1
2062	Mthfd1l~Q3	Mthfd1l	ko vs wt	ko	wt	27,2631225	0,6170813	26,6574858	0,05539816	1,52165017	0,60563672	0,44583937	0,1875075	0,72698136	Unpaired t-te	0,327248193	permutation FDR (250 perm	1
2063	Mthfd1~Q92	Mthfd1	ko vs wt	ko	wt	29,5245357	0,62165097	29,1477147	0,17147367	1,29847752	0,37682104	0,27328883	0,38113881	0,41891683	Unpaired t-te	0,532637255	permutation FDR (250 perm	1
2064	Mtif2~Q91YJ	Mtif2	ko vs wt	ko	wt	26,640649	0,20404737	26,0451197	0,136594	1,51102684	0,59552929	0,51680008	0,01130425	1,94675842	Unpaired t-te	0,246544656	permutation FDR (250 perm	1
2065	Mtm1~Q9Z2	Mtm1	ko vs wt	ko	wt	25,3021708	1,01801558	25,8060559	0,04891733	0,70520515	-0,5038851	-0,3171545	0,45461065	0,34236039	Unpaired t-te	0,476993703	permutation FDR (250 perm	1
2066	Mtmr10~Q7	Mtmr10	ko vs wt	ko	wt	26,0491875	1,44110945	24,5125312	0,09971136	2,90121322	1,53665633	0,83741171	0,16168896	0,79131962	Unpaired t-te	0,096709167	permutation FDR (250 perm	1
2067	Mtor~Q9JLN	Mtor	ko vs wt	ko	wt	28,2000447	0,2862809	28,5939325	0,73885184	0,76107584	-0,3938879	-0,2544546	0,53570741	0,27107235	Unpaired t-te	0,574037895	permutation FDR (250 perm	1
2068	Mtpap~Q9D	Mtpap	ko vs wt	ko	wt	23,8232943	0,54364671	24,2303626	0,31124867	0,75415435	-0,4070683	-0,2942633	0,33781026	0,47132716	Unpaired t-te	0,494137824	permutation FDR (250 perm	1
2069	Mtpn~P6277	Mtpn	ko vs wt	ko	wt	23,7346677	0,88482304	24,3492385	0,18695202	0,65312417	-0,6145708	-0,4022903	0,31946854	0,4955719	Unpaired t-te	0,370477983	permutation FDR (250 perm	1
2070	Mtrex~Q9CZ	Mtrex	ko vs wt	ko	wt	27,3876014	0,37148701	26,9172215	0,31516197	1,38547417	0,47037982	0,35926146	0,19217583	0,71630124	Unpaired t-te	0,409085789	permutation FDR (250 perm	1
2071	Mtr~A6H5Y3	Mtr	ko vs wt	ko	wt	24,6683363	1,03629789	24,3820176	0,76974167	1,21952445	0,28631868	0,15828856	0,73794848	0,13197396	Unpaired t-te	0,716677134	permutation FDR (250 perm	1
2072	Mtus2~Q3U	Mtus2	ko vs wt	ko	wt	24,4339653	0,6010406	25,2332308	0,83409254	0,57464169	-0,7992654	-0,4745376	0,31903184	0,49616597	Unpaired t-te	0,295262899	permutation FDR (250 perm	1
2073	Mtx2~O8844	Mtx2	ko vs wt	ko	wt	27,6548042	0,31686725	24,3716203	2,51699202	9,7350194	3,28318385	1,1771229	0,20530005	0,68761095	Unpaired t-te	0,042978824	permutation FDR (250 perm	1
2074	Mug1~P286	Mug1	ko vs wt	ko	wt	33,7198799	0,39198923	32,9423574	0,4458997	1,71418466	0,77752253	0,56012908	0,11745526	0,93012752	Unpaired t-te	0,220691673	permutation FDR (250 perm	1
2075	Mup10~A2B	Mup10	ko vs wt	ko	wt	23,5165613	0,46050189	24,713888	0,46296071	0,43608258	-1,1973267	-0,8421636	0,04348402	1,36167028	Unpaired t-te	0,085163776	permutation FDR (250 perm	1
2076	Mvb12a~Q7	Mvb12a	ko vs wt	ko	wt	26,4345684	0,58212525	26,5261688	0,48099516	0,93848106	-0,0916005	-0,0619693	0,85595481	0,06754916	Unpaired t-te	0,892506616	permutation FDR (250 perm	1
2077	Mvp~E9Q3X	Mvp	ko vs wt	ko	wt	23,9512647	0,7920084	24,5658865	0,89181291	0,65310106	-0,6146218	-0,3454977	0,47494425	0,32335736	Unpaired t-te	0,431476913	permutation FDR (250 perm	1
2078	Mvp~Q9EQK	Mvp	ko vs wt	ko	wt	31,1867733	0,60036781	30,7240015	0,31817133	1,37818718	0,46277184	0,32745555	0,31579656	0,50059261	Unpaired t-te	0,448733312	permutation FDR (250 perm	1
2079	Mxra8~Q9DE	Mxra8	ko vs wt	ko	wt	25,811878	0,83487438	25,1212501	1,36584208	1,61398588	0,69062796	0,33212875	0,56784532	0,24576995	Unpaired t-te	0,457137405	permutation FDR (250 perm	1
2080	Mybbp1a~Q	Mybbp1a	ko vs wt	ko	wt	28,6112624	0,66173295	27,4119948	0,96566558	2,29623062	1,19926754	0,67282203	0,21633266	0,66487791	Unpaired t-te	0,16071028	permutation FDR (250 perm	1
2081	Mybpc1~Q6	Mybpc1	ko vs wt	ko	wt	24,5324416	1,83420199	24,5766097	0,48189656	0,96984887	-0,0441681	-0,0209085	0,97043445	0,01303379	Unpaired t-te	0,963735155	permutation FDR (250 perm	1
2082	Mybpc2~Q5	Mybpc2	ko vs wt	ko	wt	27,2089551	0,94659045	24,3629201	0,65255416	7,19021551	2,84603501	1,65924786	0,01056387	1,97617683	Unpaired t-te	0,005226463	permutation FDR (250 perm	1
2083	Mybphl~Q5F	Mybphl	ko vs wt	ko	wt	24,3564506	0,6675161	24,3469201	0,78154656	1,00662794	0,00953054	0,00569414	0,98942214	0,00461838	Unpaired t-te	0,989599583	permutation FDR (250 perm	1
2084	Mycbp2~F6S	Mycbp2	ko vs wt	ko	wt	27,0532769	0,15479314	27,1559121	0,503965	0,93133029	-0,1026352	-0,0750591	0,80355499	0,0949844	Unpaired t-te	0,878098967	permutation FDR (250 perm	1
2085	Myd88~P223	Myd88	ko vs wt	ko	wt	24,666144	0,67712112	24,080848	0,77369755	1,50034686	0,58529607	0,34997168	0,43449581	0,36201441	Unpaired t-te	0,426739708	permutation FDR (250 perm	1
2086	Myef2~Q8C8	Myef2	ko vs wt	ko	wt	27,4064887	0,64621098	27,1245574	0,59184542	1,21582139	0,2819313	0,18064907	0,63852493	0,19482214	Unpaired t-te	0,678552502	permutation FDR (250 perm	1
2087	Myh10~Q61	Myh10	ko vs wt	ko	wt	35,0107556	0,42116346	35,514874	0,37235899	0,70509112	-0,5041184	-0,3711114	0,22307184	0,65155526	Unpaired t-te	0,395674035	permutation FDR (250 perm	1
2088	Myh11~E9Q	Myh11	ko vs wt	ko	wt	38,8751231	0,73704667	39,6707016	0,14681579	0,57611211	-0,7955785	-0,5532479	0,15724464	0,80342414	Unpaired t-te	0,231828805	permutation FDR (250 perm	1
2089	Myh11~O08	Myh11	ko vs wt	ko	wt	28,3170375	1,30439989	29,8690961	0,59745165	0,34102312	-1,5520586	-0,8328741	0,13846163	0,85867056	Unpaired t-te	0,086826923	permutation FDR (250 perm	1
2090	Myh14~Q6U	Myh14	ko vs wt	ko	wt	31,6300525	1,37343259	32,8783723	0,14470862	0,42093815	-1,2483198	-0,6936935	0,21349256	0,67061725	Unpaired t-te	0,152251852	permutation FDR (250 perm	1
2091	Myh1~Q5SX	Myh1	ko vs wt	ko	wt	30,1842409	2,45936701	26,5978384	1,90530253	12,0119838	3,58640253	1,21270484	0,1277203	0,89374006	Unpaired t-te	0,019899785	permutation FDR (250 perm	1
2092	Myh2~G3UW	Myh2	ko vs wt	ko	wt	28,3769973	2,78069417	24,9554787	0,77202183	10,7146932	3,42151863	1,2692463	0,12029277	0,91976047	Unpaired t-te	0,019546303	permutation FDR (250 perm	1
2093	Myh3~P1354	Myh3	ko vs wt	ko	wt	25,5215564	2,23207356	24,3744097	0,54520231	2,21475437	1,1471467	0,48916391	0,44789841	0,34882048	Unpaired t-te	0,279547518	permutation FDR (250 perm	1
2094	Myh4~Q5SX	Myh4	ko vs wt	ko	wt	34,1556254	2,17327474	32,2701335	1,1992943	3,6947889	1,88549194	0,74986563	0,26991126	0,56877899	Unpaired t-te	0,11515233	permutation FDR (250 perm	1
2095	Myh6~Q025	Myh6	ko vs wt	ko	wt	30,743779	2,56420937	29,9701623	3,53770085	1,70955016	0,77361675	0,19801887	0,80559502	0,09388323	Unpaired t-te	0,654234917	permutation FDR (250 perm	1
2096	Myh8~P1354	Myh8	ko vs wt	ko	wt	28,7866971	1,92170098	24,4532897	0,55166631	20,1597725	4,33340746	1,99138946	0,02416822	1,61675539	Unpaired t-te	0,003730263	permutation FDR (250 perm	1
2097	Myh9~Q8VD	Myh9	ko vs wt	ko	wt	36,1380961	0,40397538	35,866131	0,4485412	1,20745139	0,27196511	0,19514005	0,52792332	0,27742916	Unpaired t-te	0,655522145	permutation FDR (250 perm	1
2098	Myl11~P974	Myl11	ko vs wt	ko	wt	26,6000484	2,38046264	23,6355657	1,06882341	7,8054551	2,96448275	1,15418793	0,12413331	0,90611165	Unpaired t-te	0,026383266	permutation FDR (250 perm	1
2099	Myl12a~Q6Z	Myl12a	ko vs wt	ko	wt	30,323953	1,07735835	28,9843903	0,35420622	2,530746	1,33956272	0,8018714	0,11826325	0,92715019	Unpaired t-te	0,100832029	permutation FDR (250 perm	1
2100	Myl1~P0597	Myl1	ko vs wt	ko	wt	30,1939356	0,74723042	29,5992437	0,15459373	1,51015007	0,59469192	0,41153795	0,26438916	0,57775635	Unpaired t-te	0,360714479	permutation FDR (250 perm	1
2101	Myl4~A2A6C	Myl4	ko vs wt	ko	wt	25,4790821	1,55022029	24,9976466	1,13621735	1,39613212	0,48143547	0,21856323	0,70561693	0,15143101	Unpaired t-te	0,609996452	permutation FDR (250 perm	1
2102	Myl6~Q6060	Myl6	ko vs wt	ko	wt	34,1577285	0,1168624	33,986302	0,17116635	1,12617139	0,17142641	0,1505631	0,29840465	0,52519441	Unpaired t-te	0,73707505	permutation FDR (250 perm	1
2103	Myl6~Q6060	Myl6	ko vs wt	ko	wt	25,3433456	1,12528039	25,6260822	0,79808427	0,82203026	-0,2827366	-0,1519636	0,75585665	0,12156056	Unpaired t-te	0,727634091	permutation FDR (250 perm	1
2104	Myl7~Q9QV	Myl7	ko vs wt	ko	wt	26,2410448	2,56314194	26,4114794	2,1254589	0,88857497	-0,1704346	-0,0548164	0,938877	0,0273913	Unpaired t-te	0,904677237	permutation FDR (250 perm	1
2105	Myl9~Q9CQ1	Myl9	ko vs wt	ko	wt	32,7927507	0,33421817	32,4470795	0,4493998	1,27074207	0,34567123	0,25198894	0,41307343	0,38397274	Unpaired t-te	0,565439952	permutation FDR (250 perm	1
2106	Mylk~Q6PDN	Mylk	ko vs wt	ko	wt	33,9227754	0,66490584	34,67061	0,36047039	0,5954967	-0,7478346	-0,5119352	0,16768087	0,77551647	Unpaired t-te	0,251265306	permutation FDR (250 perm	1
2107	Myo10~F8V	Myo10	ko vs wt	ko	wt	23,7132927	0,94926909	23,9887802	0,42062005	0,8261711	-0,2754875	-0,1696808	0,67927536	0,16795414	Unpaired t-te	0,697332951	permutation FDR (250 perm	1
2108	Myo18a~Q9J	Myo18a	ko vs wt	ko	wt	29,2146135	0,46843946	29,1197599	0,46688586	1,06795699	0,09485355	0,06648111	0,834286	0,07868504	Unpaired t-te	0,885925244	permutation FDR (250 perm	1
2109	Myo1b~P467	Myo1b	ko vs wt	ko	wt	26,6946273	2,09788326	26,1870472	0,45579718	1,42166357	0,5075801	0,22525468	0,70955786	0,14901218	Unpaired t-te	0,607198817	permutation FDR (250 perm	1

2110	Myo1c~Q9W	Myo1c	ko vs wt	ko	wt	34,6281858	0,23106538	34,8268	0,24201247	0,87138717	-0,1986142	-0,163202	0,41007215	0,38713972	Unpaired t-te	0,710235698	permutation FDR (250 perm	1
2111	Myo1c~Q9W	Myo1c	ko vs wt	ko	wt	25,1462853	1,33143018	27,6334789	0,34287464	0,17835288	-2,4871936	-1,3771611	0,04270033	1,36956875	Unpaired t-te	0,014316975	permutation FDR (250 perm	1
2112	Myo1d~Q5SY	Myo1d	ko vs wt	ko	wt	32,0569399	0,1661535	31,9186685	0,15689802	1,10058563	0,13827139	0,12058562	0,3941129	0,40437935	Unpaired t-te	0,78727354	permutation FDR (250 perm	1
2113	Myo1e~E9Q6	Myo1e	ko vs wt	ko	wt	31,5753957	0,26284918	31,2583057	0,6386265	1,24581511	0,31708998	0,21477319	0,56275453	0,249681	Unpaired t-te	0,636395656	permutation FDR (250 perm	1
2114	Myo1f~Q8CG	Myo1f	ko vs wt	ko	wt	27,5949906	1,27479481	27,1668358	0,53380944	1,34551163	0,42815486	0,23432936	0,6300178	0,20064718	Unpaired t-te	0,586591045	permutation FDR (250 perm	1
2115	Myo1g~Q5SU	Myo1g	ko vs wt	ko	wt	30,1503044	0,99506696	29,3308085	0,36606334	1,7647892	0,81949587	0,50272053	0,26187548	0,58190517	Unpaired t-te	0,263771058	permutation FDR (250 perm	1
2116	Myo5a~D3YZ	Myo5a	ko vs wt	ko	wt	28,0260402	1,17220915	27,578653	1,24926578	1,36356848	0,44738716	0,21174933	0,70775813	0,15011513	Unpaired t-te	0,626731822	permutation FDR (250 perm	1
2117	Myo6~E9PVU	Myo6	ko vs wt	ko	wt	29,1865892	0,47203599	28,794214	0,61461918	1,31255253	0,39237516	0,25933887	0,49238296	0,30769699	Unpaired t-te	0,554027879	permutation FDR (250 perm	1
2118	Myo7a~P974	Myo7a	ko vs wt	ko	wt	27,4072315	1,52188962	27,1905924	0,49441628	1,16202338	0,21663909	0,11134478	0,83036934	0,08072869	Unpaired t-te	0,805709839	permutation FDR (250 perm	1
2119	Myo9a~Q8C1	Myo9a	ko vs wt	ko	wt	24,3035164	0,5563491	24,1692169	0,18325905	1,09755972	0,13429944	0,09974987	0,71843096	0,14361496	Unpaired t-te	0,826280353	permutation FDR (250 perm	1
2120	Myo9b~E9PZ	Myo9b	ko vs wt	ko	wt	29,2381764	0,62743362	28,5591088	0,34150128	1,60110462	0,67906758	0,47310006	0,18160643	0,74086877	Unpaired t-te	0,283690569	permutation FDR (250 perm	1
2121	Myoc~O7062	Myoc	ko vs wt	ko	wt	24,685827	0,84523773	24,0196518	0,3700543	1,58686036	0,66617518	0,42875979	0,28934355	0,5385862	Unpaired t-te	0,331715562	permutation FDR (250 perm	1
2122	Myof~Q69ZN	Myof	ko vs wt	ko	wt	33,0921054	0,06102821	33,0911381	0,3285541	1,00067075	0,00096736	0,0007833	0,99707432	0,00127247	Unpaired t-te	0,998839615	permutation FDR (250 perm	1
2123	Myom1~Q62	Myom1	ko vs wt	ko	wt	27,7893647	1,07423896	24,5142811	0,46566648	9,68051356	3,27508359	1,9240291	0,00756217	2,12135342	Unpaired t-te	0,003721562	permutation FDR (250 perm	1
2124	Myom2~Q14	Myom2	ko vs wt	ko	wt	28,6074862	1,10295729	25,0961364	2,03693955	11,4030649	3,51134974	1,36372491	0,11880802	0,92515426	Unpaired t-te	0,018306227	permutation FDR (250 perm	1
2125	Myom3~A2A	Myom3	ko vs wt	ko	wt	24,6768384	0,83346296	24,6141493	0,23649348	1,0444107	0,06268914	0,04153169	0,90847267	0,04168813	Unpaired t-te	0,928599574	permutation FDR (250 perm	1
2126	Myoz1~Q9JK	Myoz1	ko vs wt	ko	wt	25,4186664	2,05133297	24,8880371	0,58597806	1,4445591	0,53062923	0,23534075	0,69579712	0,15751737	Unpaired t-te	0,588849642	permutation FDR (250 perm	1
2127	NARS1~Q8B	NARS1	ko vs wt	ko	wt	30,3095328	0,49813548	29,8599838	0,24891392	1,36561326	0,44954897	0,33619118	0,24362262	0,61328238	Unpaired t-te	0,437785347	permutation FDR (250 perm	1
2128	Naa10~Q9Q	Naa10	ko vs wt	ko	wt	26,9750043	0,37690028	26,8764855	0,36770543	1,07067363	0,09851878	0,07357364	0,78468876	0,10530257	Unpaired t-te	0,874573686	permutation FDR (250 perm	1
2129	Naa15~G3X8	Naa15	ko vs wt	ko	wt	29,7013494	0,39706206	29,9246435	0,49370743	0,85660733	-0,2232941	-0,1575107	0,62382229	0,20493911	Unpaired t-te	0,72227596	permutation FDR (250 perm	1
2130	Naa16~Q9DE	Naa16	ko vs wt	ko	wt	24,0748308	0,82575067	24,6278964	0,57169003	0,68157033	-0,5530656	-0,340335	0,4168234	0,38004791	Unpaired t-te	0,431585405	permutation FDR (250 perm	1
2131	Naa25~Q8B\	Naa25	ko vs wt	ko	wt	27,9117689	0,85791422	28,9027569	0,59877622	0,5031331	-0,990988	-0,6000106	0,1889051	0,72375631	Unpaired t-te	0,187443058	permutation FDR (250 perm	1
2132	Naa35~Q6PI	Naa35	ko vs wt	ko	wt	25,0375628	0,75676261	24,376428	0,54147633	1,581326	0,66113482	0,41819015	0,30691936	0,51297571	Unpaired t-te	0,339998632	permutation FDR (250 perm	1
2133	Naa50~Q6P(	Naa50	ko vs wt	ko	wt	27,4485733	0,83827542	27,3525572	0,32446104	1,06881789	0,09601606	0,06252654	0,86607928	0,06244235	Unpaired t-te	0,892280853	permutation FDR (250 perm	1
2134	Naalad2~G3	Naalad2	ko vs wt	ko	wt	27,5395996	0,10981462	27,8055837	0,01792527	0,83163123	-0,2659842	-0,2498311	0,0224497	1,64878943	Unpaired t-te	0,570447456	permutation FDR (250 perm	1
2135	Nab2~Q6112	Nab2	ko vs wt	ko	wt	24,4956963	0,77108695	23,9417823	0,49030883	1,46806308	0,55391396	0,35410532	0,37143963	0,43011176	Unpaired t-te	0,414015008	permutation FDR (250 perm	1
2136	Naca~P7067	Naca	ko vs wt	ko	wt	27,0792968	0,75593935	26,2924606	0,56035526	1,72528682	0,78683623	0,49502909	0,24009629	0,61961455	Unpaired t-te	0,264738395	permutation FDR (250 perm	1
2137	Naga~Q9QW	Naga	ko vs wt	ko	wt	27,4491297	0,17486682	27,0075939	0,27184539	1,3580493	0,44153586	0,36276987	0,13197435	0,87951048	Unpaired t-te	0,418664495	permutation FDR (250 perm	1
2138	Nagk~Q9QZ(	Nagk	ko vs wt	ko	wt	26,884539	0,25431939	24,9231444	1,56428052	3,89438255	1,96139461	0,92701551	0,21655572	0,66443035	Unpaired t-te	0,088310196	permutation FDR (250 perm	1
2139	Naglu~O883	Naglu	ko vs wt	ko	wt	25,3705705	1,46299231	25,1483836	0,81784592	1,16650045	0,22218687	0,10979443	0,83697205	0,07728905	Unpaired t-te	0,80591662	permutation FDR (250 perm	1
2140	Nampt~Q99I	Nampt	ko vs wt	ko	wt	25,2619513	0,64971627	24,5576659	0,4664936	1,6293374	0,70428539	0,46967441	0,21792821	0,66168654	Unpaired t-te	0,286364727	permutation FDR (250 perm	1
2141	Nans~Q99J7	Nans	ko vs wt	ko	wt	27,3680723	0,87115245	25,2276438	0,02900404	4,4089299	2,14042854	1,42374601	0,0236817	1,62558722	Unpaired t-te	0,014416	permutation FDR (250 perm	1
2142	Nap1l1~P28	Nap1l1	ko vs wt	ko	wt	29,7406973	0,34739451	28,7803529	0,74163396	1,94577432	0,96034439	0,6150295	0,19993123	0,69911937	Unpaired t-te	0,201829755	permutation FDR (250 perm	1
2143	Nap1l4~Q78	Nap1l4	ko vs wt	ko	wt	30,6701518	0,32690541	30,1407707	0,56366839	1,4433099	0,5293811	0,36736995	0,31878327	0,49650448	Unpaired t-te	0,415462842	permutation FDR (250 perm	1
2144	Napa~Q9DB(	Napa	ko vs wt	ko	wt	28,7598413	0,35081575	27,2906687	0,31880109	2,76863053	1,46917254	1,12748509	0,00598877	2,22266267	Unpaired t-te	0,028813604	permutation FDR (250 perm	1
2145	Napg~Q9CW	Napg	ko vs wt	ko	wt	24,5827941	0,70000439	24,4649135	0,25949627	1,08515838	0,11788063	0,08164316	0,80337173	0,09508346	Unpaired t-te	0,859987965	permutation FDR (250 perm	1
2146	Nat2~P5029	Nat2	ko vs wt	ko	wt	24,1285515	0,95646452	24,2713276	0,52368131	0,9057746	-0,142776	-0,0857577	0,83872766	0,07637903	Unpaired t-te	0,851210974	permutation FDR (250 perm	1
2147	Nav1~Q8CH7	Nav1	ko vs wt	ko	wt	24,6570997	0,81614144	24,5731204	1,13712343	1,05993759	0,08397933	0,04346841	0,93332727	0,02996604	Unpaired t-te	0,9266656	permutation FDR (250 perm	1
2148	Naxd~Q9CZ4	Naxd	ko vs wt	ko	wt	28,0479195	0,46059321	27,1751126	0,30867795	1,83122215	0,87280682	0,6493955	0,05208437	1,28329258	Unpaired t-te	0,159679414	permutation FDR (250 perm	1
2149	Naxe~Q8K4Z	Naxe	ko vs wt	ko	wt	26,965911	0,368868	25,0512637	0,94866561	3,77021641	1,91464734	1,12374967	0,09229536	1,03482012	Unpaired t-te	0,043806075	permutation FDR (250 perm	1
2150	Nbas~E9Q41	Nbas	ko vs wt	ko	wt	29,3410447	0,57983755	29,3970747	0,68673229	0,96190747	-0,05603	-0,0352433	0,92913398	0,03192166	Unpaired t-te	0,939569231	permutation FDR (250 perm	1
2151	Nbeal1~E9P	Nbeal1	ko vs wt	ko	wt	25,6289818	2,01849656	23,8279446	0,43570782	3,48470658	1,80103719	0,81664248	0,2213199	0,65497954	Unpaired t-te	0,099787823	permutation FDR (250 perm	1
2152	Nbeal2~Q6Z	Nbeal2	ko vs wt	ko	wt	25,2163387	0,49195265	24,2587687	0,07593505	1,94203616	0,95757006	0,74284382	0,04098062	1,38742148	Unpaired t-te	0,130483592	permutation FDR (250 perm	1
2153	Ncam1~E9Q	Ncam1	ko vs wt	ko	wt	30,195431	0,45563429	30,3597396	0,33215812	0,89235609	-0,1643086	-0,1214712	0,66101362	0,17978959	Unpaired t-te	0,784104027	permutation FDR (250 perm	1
2154	Ncapg~E9PW	Ncapg	ko vs wt	ko	wt	24,0807733	0,36929017	24,3870138	0,36556088	0,80874651	-0,3062405	-0,2293806	0,40890942	0,38837288	Unpaired t-te	0,594514286	permutation FDR (250 perm	1
2155	Ncbp1~Q3U	Ncbp1	ko vs wt	ko	wt	26,080756	0,23174234	26,0899048	0,23110743	0,9936786	-0,0091488	-0,0075535	0,96738889	0,01439891	Unpaired t-te	0,986381027	permutation FDR (250 perm	1
2156	Ncdn~Q9Z0E	Ncdn	ko vs wt	ko	wt	24,1732296	0,57533883	24,3748484	0,30481443	0,86957431	-0,2016188	-0,1444289	0,63322768	0,19844011	Unpaired t-te	0,741013002	permutation FDR (250 perm	1
2157	Nceh1~Q8BL	Nceh1	ko vs wt	ko	wt	27,8523204	0,78595106	27,3957355	0,49952729	1,37228951	0,45658488	0,28988794	0,46326604	0,33416954	Unpaired t-te	0,500381841	permutation FDR (250 perm	1
2158	Ncf1~Q0901	Ncf1	ko vs wt	ko	wt	26,6877588	0,67015913	25,4283773	0,50384546	2,39393089	1,25938151	0,82530408	0,06277536	1,2022108	Unpaired t-te	0,087272553	permutation FDR (250 perm	1
2159	Ncf2~O7014	Ncf2	ko vs wt	ko	wt	27,4032867	0,70162174	26,2972451	0,00802791	2,15254227	1,10604157	0,78715025	0,07190049	1,14326815	Unpaired t-te	0,115639946	permutation FDR (250 perm	1
2160	Ncf4~P9736	Ncf4	ko vs wt	ko	wt	26,1072251	0,37371577	24,8593774	0,46498698	2,37484867	1,24784773	0,8956255	0,03846928	1,41488596	Unpaired t-te	0,074748749	permutation FDR (250 perm	1
2161	Nck1~Q99M	Nck1	ko vs wt	ko	wt	25,0023274	1,38489492	27,0676975	0,06867089	0,23892504	-2,0653701	-1,1467636	0,08129187	1,08995287	Unpaired t-te	0,034771536	permutation FDR (250 perm	1
2162	Nckap1l~Q8	Nckap1l	ko vs wt	ko	wt	27,2596749	0,64012397	27,81235	0,58829114	0,68175484	-0,5526751	-0,3550884	0,37070722	0,43096895	Unpaired t-te	0,415401565	permutation FDR (250 perm	1
2163	Nckap1~P28	Nckap1	ko vs wt	ko	wt	29,2587378	0,37281156	29,3446717	0,11759343	0,94217444	-0,0859339	-0,0698226	0,72924764	0,13712497	Unpaired t-te	0,88228913	permutation FDR (250 perm	1
2164	Ncln~Q8VCN	Ncln	ko vs wt	ko	wt	28,2046354	0,67946077	27,2201342	0,72644623	1,97862912	0,98450122	0,59799509	0,20089635	0,69702796	Unpaired t-te	0,194682153	permutation FDR (250 perm	1
2165	Ncl~P09405	Ncl	ko vs wt	ko	wt	31,4317344	0,25745442	31,4895165	0,35580538	0,96073996	-0,0577821	-0,0447153	0,85460209	0,06823605	Unpaired t-te	0,924867058	permutation FDR (250 perm	1
2166	Ncor2~Q9W	Ncor2	ko vs wt	ko	wt	25,953328	1,86339861	25,3423154	1,55222138	1,52733088	0,61101264	0,2408485	0,7081546	0,14987192	Unpaired t-te	0,574922429	permutation FDR (250 perm	1
2167	Ncstn~P5771	Ncstn	ko vs wt	ko	wt	26,7507711	0,50666777	24,5295948	0,65369244	4,66273431	2,22117622	1,43578006	0,0195195	1,70953121	Unpaired t-te	0,011979678	permutation FDR (250 perm	1
2168	Ndc1~Q8VCE	Ndc1	ko vs wt	ko	wt	24,6642671	0,44862444	23,5706134	0,4945276	2,13413828	1,09365366	0,76204149	0,06591034	1,18104642	Unpaired t-te	0,115625448	permutation FDR (250 perm	1
2169	Nde1~Q9CZ	Nde1	ko vs wt	ko	wt	24,3564769	0,59956016	24,4639638	0,20260052	0,92820353	-0,1074869	-0,0781933	0,7886246	0,10312968	Unpaired t-te	0,866251092	permutation FDR (250 perm	1
2170	Ndrg1~Q624	Ndrg1	ko vs wt	ko	wt	27,9659805	0,59286544	28,0281883	0,46590706	0,95779721	-0,0622079	-0,0421726	0,90105989	0,04524634	Unpaired t-te	0,927114903	permutation FDR (250 perm	1
2171	Ndrg2~Q9Q	Ndrg2	ko vs wt	ko	wt	24,2681629	0,7921969	24,9243008	1,50671098	0,63457479	-0,6561379	-0,3038475	0,61441096	0,21154105	Unpaired t-te	0,498533126	permutation FDR (250 perm	1
2172	Ndufa10~Q9	Ndufa10	ko vs wt	ko	wt	30,6476443	0,23880269	30,5277607	0,15518381	1,08664714	0,11988354	0,1019236	0,52652398	0,27858185	Unpaired t-te	0,819765552	permutation FDR (250 perm	1
2173	Ndufa11~G5	Ndufa11	ko vs wt	ko	wt	26,7000509	0,26529877	26,8556703	0,22564334	0,89774685	-0,1556194	-0,1274341	0,51489017	0,2882854	Unpaired t-te	0,773109055	permutation FDR (250 perm	1

2174	Ndufa12~Q7	Ndufa12	ko vs wt	ko	wt	27,928325	0,34243421	27,7280596	0,36081886	1,14890967	0,20026538	0,15139818	0,56756062	0,24598774	Unpaired t-te	0,731501703	permutation FDR (250 perm	1
2175	Ndufa13~Q9	Ndufa13	ko vs wt	ko	wt	27,1612721	0,54000218	26,8842487	0,54122274	1,2116923	0,27702339	0,18547099	0,6027536	0,21986019	Unpaired t-te	0,671453596	permutation FDR (250 perm	1
2176	Ndufa2~Q9C	Ndufa2	ko vs wt	ko	wt	24,546268	0,13780056	27,8469786	0,72626992	0,10148155	-3,3007106	-2,1719824	0,02125247	1,6725906	Unpaired t-te	0,005142857	permutation FDR (250 perm	1
2177	Ndufa4~Q62	Ndufa4	ko vs wt	ko	wt	29,770309	0,50567549	30,5631041	0,33115496	0,57722471	-0,792795	-0,5768903	0,0877087	1,05695731	Unpaired t-te	0,202587965	permutation FDR (250 perm	1
2178	Ndufa5~Q9C	Ndufa5	ko vs wt	ko	wt	25,555704	1,37722023	26,9564392	0,18641386	0,37873611	-1,4007351	-0,775605	0,17586034	0,75483208	Unpaired t-te	0,118419269	permutation FDR (250 perm	1
2179	Ndufa6~Q9C	Ndufa6	ko vs wt	ko	wt	30,0788344	0,360194	29,9695505	0,30867382	1,07869263	0,10928383	0,08396931	0,73271059	0,13506753	Unpaired t-te	0,8541616	permutation FDR (250 perm	1
2180	Ndufa8~Q9D	Ndufa8	ko vs wt	ko	wt	27,69721	0,60451659	27,5919098	0,31565296	1,07571823	0,10530023	0,07445476	0,80997116	0,09153045	Unpaired t-te	0,872490864	permutation FDR (250 perm	1
2181	Ndufa9~Q9D	Ndufa9	ko vs wt	ko	wt	30,3215834	0,35987422	30,075145	0,37431505	1,18627496	0,24643844	0,18439225	0,50298943	0,29844114	Unpaired t-te	0,674367063	permutation FDR (250 perm	1
2182	Ndufab1~Q9	Ndufab1	ko vs wt	ko	wt	27,6148445	0,96176257	25,2868655	0,35260819	5,02101491	2,32797901	1,44713321	0,01800785	1,74453807	Unpaired t-te	0,010586331	permutation FDR (250 perm	1
2183	Ndufb10~Q9	Ndufb10	ko vs wt	ko	wt	26,4210151	1,74540386	27,390696	0,4531594	0,51061898	-0,9696809	-0,4713072	0,41661244	0,38026777	Unpaired t-te	0,295563662	permutation FDR (250 perm	1
2184	Ndufb4~Q9C	Ndufb4	ko vs wt	ko	wt	28,9152622	0,25270557	28,2222057	0,34105304	1,61670501	0,69305646	0,54066454	0,08038879	1,09480452	Unpaired t-te	0,23999115	permutation FDR (250 perm	1
2185	Ndufb5~Q9C	Ndufb5	ko vs wt	ko	wt	27,5661753	0,45913379	27,2120014	0,08924803	1,27825344	0,35417391	0,27833149	0,2762582	0,55868482	Unpaired t-te	0,527318001	permutation FDR (250 perm	1
2186	Ndufb6~Q3U	Ndufb6	ko vs wt	ko	wt	24,3122654	0,51202873	25,7779489	0,05534011	0,36206396	-1,4656835	-1,1290133	0,01487814	1,82745126	Unpaired t-te	0,035554736	permutation FDR (250 perm	1
2187	Ndufb7~Q9C	Ndufb7	ko vs wt	ko	wt	25,1023759	1,08978272	23,0804128	0,27978837	4,06136038	2,02196305	1,21837473	0,04352991	1,36121223	Unpaired t-te	0,023827042	permutation FDR (250 perm	1
2188	Ndufb9~Q9C	Ndufb9	ko vs wt	ko	wt	29,2138871	0,36984708	28,8298202	0,34360323	1,3050155	0,38406694	0,29019885	0,29401577	0,53162938	Unpaired t-te	0,502693192	permutation FDR (250 perm	1
2189	Ndufc2~Q9C	Ndufc2	ko vs wt	ko	wt	27,9250965	0,06366375	28,441019	0,46024652	0,69934564	-0,5159224	-0,3886385	0,25284017	0,59715393	Unpaired t-te	0,408217776	permutation FDR (250 perm	1
2190	Ndufs1~Q91	Ndufs1	ko vs wt	ko	wt	31,0252505	0,36264415	31,2442311	0,15800859	0,85917229	-0,2189806	-0,1769799	0,40376342	0,39387303	Unpaired t-te	0,684491066	permutation FDR (250 perm	1
2191	Ndufs2~Q91	Ndufs2	ko vs wt	ko	wt	29,9499495	0,36288904	29,3966874	0,5567111	1,46739996	0,55326215	0,38263228	0,29968764	0,52333116	Unpaired t-te	0,395014856	permutation FDR (250 perm	1
2192	Ndufs3~Q9D	Ndufs3	ko vs wt	ko	wt	28,4321587	0,18992304	28,7858447	0,26647396	0,7825821	-0,353686	-0,2903805	0,19415651	0,71184805	Unpaired t-te	0,510829208	permutation FDR (250 perm	1
2193	Ndufs5~Q99	Ndufs5	ko vs wt	ko	wt	24,3461432	1,03413348	24,4681263	0,28157133	0,91892363	-0,1219831	-0,0748648	0,85665706	0,067193	Unpaired t-te	0,873642507	permutation FDR (250 perm	1
2194	Ndufs7~Q9D	Ndufs7	ko vs wt	ko	wt	27,8356494	0,58430454	27,4138826	0,65941653	1,33956707	0,42176682	0,2677007	0,50501187	0,29669841	Unpaired t-te	0,539079743	permutation FDR (250 perm	1
2195	Ndufs8~Q8K	Ndufs8	ko vs wt	ko	wt	27,7399576	0,31132167	27,8912336	0,60961675	0,90045371	-0,151276	-0,1031167	0,76943999	0,11382524	Unpaired t-te	0,824853201	permutation FDR (250 perm	1
2196	Ndufv1~Q91	Ndufv1	ko vs wt	ko	wt	29,7228192	0,09929561	29,7393796	0,15208334	0,9885868	-0,0165604	-0,0147615	0,90018041	0,04567044	Unpaired t-te	0,974509291	permutation FDR (250 perm	1
2197	Nebl~B7ZCI2	Nebl	ko vs wt	ko	wt	25,1073197	0,7391613	23,9464186	0,54906097	2,23597042	1,1609011	0,7361757	0,10102706	0,99556227	Unpaired t-te	0,120649512	permutation FDR (250 perm	1
2198	Nebl~Q9DC0	Nebl	ko vs wt	ko	wt	24,4095972	0,73520114	24,9343761	0,37501898	0,69506562	-0,5247789	-0,3497377	0,3450886	0,46206939	Unpaired t-te	0,421434797	permutation FDR (250 perm	1
2199	Neb~A2AQB	Neb	ko vs wt	ko	wt	25,4442763	1,62541065	24,0051103	0,488124	2,71164071	1,43916603	0,71962156	0,22774395	0,64255315	Unpaired t-te	0,134849573	permutation FDR (250 perm	1
2200	Neb~E9Q1W	Neb	ko vs wt	ko	wt	28,8983548	1,8298744	24,8615353	0,1647923	16,4135968	4,03681952	1,95688032	0,03073229	1,51240511	Unpaired t-te	0,004287293	permutation FDR (250 perm	1
2201	Nedd4l~E9P	Nedd4l	ko vs wt	ko	wt	26,6053858	0,50336626	25,9206724	0,48023825	1,60738257	0,68471334	0,47320808	0,1935875	0,71312269	Unpaired t-te	0,286373192	permutation FDR (250 perm	1
2202	Nedd4~P469	Nedd4	ko vs wt	ko	wt	31,5013497	0,20816718	31,3810506	0,18843353	1,08696015	0,12029906	0,1019969	0,53500164	0,27164489	Unpaired t-te	0,820423438	permutation FDR (250 perm	1
2203	Nefh~P1924	Nefh	ko vs wt	ko	wt	26,2327394	1,47587927	28,1908778	0,72776516	0,25736034	-1,9581384	-0,9813082	0,11051838	0,95656548	Unpaired t-te	0,049713963	permutation FDR (250 perm	1
2204	Nefl~P08551	Nefl	ko vs wt	ko	wt	28,8734895	0,49532229	30,4473703	1,24934497	0,33590362	-1,5738807	-0,8160936	0,20988329	0,67802213	Unpaired t-te	0,114336474	permutation FDR (250 perm	1
2205	Nefm~P085	Nefm	ko vs wt	ko	wt	27,2152429	1,21821806	30,3755903	1,51782185	0,1118512	-3,1603474	-1,3841806	0,07573481	1,12070448	Unpaired t-te	0,013439222	permutation FDR (250 perm	1
2206	Nek6~Q9ES7	Nek6	ko vs wt	ko	wt	25,0199831	0,70420098	24,2005233	0,46855656	1,7647451	0,81945981	0,53753633	0,1789437	0,74728358	Unpaired t-te	0,230558867	permutation FDR (250 perm	1
2207	Nek7~Q9ES7	Nek7	ko vs wt	ko	wt	28,529772	0,40490914	28,6248376	0,17512006	0,93622967	-0,0950656	-0,0751777	0,73592019	0,13316928	Unpaired t-te	0,871774141	permutation FDR (250 perm	1
2208	Nek9~Q8K1R	Nek9	ko vs wt	ko	wt	27,38384	0,99774802	28,5398644	0,0986561	0,44874743	-1,1560244	-0,7315412	0,1377719	0,86083934	Unpaired t-te	0,13657897	permutation FDR (250 perm	1
2209	Nelfb~Q8C4	Nelfb	ko vs wt	ko	wt	25,659698	1,31522228	25,4868622	1,30335061	1,12727207	0,17283575	0,07877161	0,89143435	0,04991063	Unpaired t-te	0,864066594	permutation FDR (250 perm	1
2210	Nelfcd~Q3T	Nelfcd	ko vs wt	ko	wt	24,6804232	0,89743045	24,6479	0,71213866	1,02279938	0,03252319	0,0188812	0,96589017	0,01507225	Unpaired t-te	0,967328782	permutation FDR (250 perm	1
2211	Nemf~Q8CC	Nemf	ko vs wt	ko	wt	24,5601864	1,1872714	26,6056366	0,25474816	0,24224685	-2,0454502	-1,1970482	0,05430184	1,26518545	Unpaired t-te	0,026643385	permutation FDR (250 perm	1
2212	Nemp1~Q6Z	Nemp1	ko vs wt	ko	wt	24,0952056	0,33086623	24,1824647	0,87719994	0,9413094	-0,0872591	-0,0529156	0,90356835	0,04403899	Unpaired t-te	0,91322681	permutation FDR (250 perm	1
2213	Nes~Q6P5H2	Nes	ko vs wt	ko	wt	28,9766663	0,39030285	29,6337094	0,24989002	0,63417675	-0,6570431	-0,5107775	0,07033634	1,15282022	Unpaired t-te	0,251290087	permutation FDR (250 perm	1
2214	Nexn~A0A0G	Nexn	ko vs wt	ko	wt	25,4464156	0,96232323	25,6525785	0,59209361	0,86683972	-0,2061628	-0,1215809	0,77894895	0,108491	Unpaired t-te	0,783821029	permutation FDR (250 perm	1
2215	Nf2~P46662	Nf2	ko vs wt	ko	wt	26,2277319	0,2167382	26,1156424	0,1881087	1,08079244	0,11208949	0,09478046	0,56802456	0,24563289	Unpaired t-te	0,834248416	permutation FDR (250 perm	1
2216	Nfia~Q0278	Nfia	ko vs wt	ko	wt	26,1761534	1,64128871	25,9122433	1,92964606	1,20072866	0,26391017	0,09916821	0,88184987	0,05460534	Unpaired t-te	0,827812466	permutation FDR (250 perm	1
2217	Nfib~P97863	Nfib	ko vs wt	ko	wt	25,8152504	1,6855007	27,2654955	0,92027281	0,36595924	-1,4502451	-0,6681155	0,2721716	0,5651572	Unpaired t-te	0,151047934	permutation FDR (250 perm	1
2218	Nfic~P70255	Nfic	ko vs wt	ko	wt	28,6549673	0,53887205	28,3158371	0,2502137	1,26499373	0,33913024	0,24974472	0,39100652	0,407816	Unpaired t-te	0,561932326	permutation FDR (250 perm	1
2219	Nfix~P70257	Nfix	ko vs wt	ko	wt	27,7598439	0,37272015	28,1535932	0,57852607	0,76114898	-0,3937492	-0,2692807	0,4550212	0,34196837	Unpaired t-te	0,543525899	permutation FDR (250 perm	1
2220	Nfkb1~P257	Nfkb1	ko vs wt	ko	wt	27,6566061	0,46886021	26,9373457	0,24681777	1,64633778	0,71926037	0,54403656	0,07839008	1,10573891	Unpaired t-te	0,226865895	permutation FDR (250 perm	1
2221	Nfkb2~Q9W	Nfkb2	ko vs wt	ko	wt	25,4367971	0,89398306	23,8077306	0,98138357	3,09312804	1,62906655	0,87356622	0,13259006	0,87748905	Unpaired t-te	0,078043608	permutation FDR (250 perm	1
2222	Nfs1~Q9Z1J	Nfs1	ko vs wt	ko	wt	27,9693688	0,21202796	27,8312416	0,68219167	1,10047564	0,13812721	0,09222791	0,80472835	0,0943507	Unpaired t-te	0,847766255	permutation FDR (250 perm	1
2223	Nfyc~P7035	Nfyc	ko vs wt	ko	wt	24,7090649	0,91843896	25,0718538	0,23696526	0,77765981	-0,3627889	-0,2331391	0,55370111	0,2567246	Unpaired t-te	0,592775	permutation FDR (250 perm	1
2224	Ngef~Q8CHT	Ngef	ko vs wt	ko	wt	24,8514358	1,24720262	26,1645241	0,10422953	0,40246342	-1,3130704	-0,7617148	0,16542734	0,78139271	Unpaired t-te	0,124700525	permutation FDR (250 perm	1
2225	Ngf~A0A0G2	Ngf	ko vs wt	ko	wt	28,3688871	0,24205405	27,9493155	0,01707123	1,33753024	0,41957151	0,36795793	0,05699986	1,24412622	Unpaired t-te	0,412893826	permutation FDR (250 perm	1
2226	Ngly1~Q9JI7	Ngly1	ko vs wt	ko	wt	24,9333917	0,95873335	27,0817171	0,02632857	0,22557429	-2,1483254	-1,382593	0,03022156	1,51968313	Unpaired t-te	0,016498078	permutation FDR (250 perm	1
2227	Ngp~O08692	Ngp	ko vs wt	ko	wt	24,7776873	1,18575116	24,6052234	0,48038889	1,12698152	0,17246386	0,0977556	0,8318514	0,07995425	Unpaired t-te	0,829542195	permutation FDR (250 perm	1
2228	Nhej1~Q3KN	Nhej1	ko vs wt	ko	wt	23,7440085	1,48047297	24,4092158	0,66494638	0,63059806	-0,6652074	-0,3367219	0,52897805	0,27656235	Unpaired t-te	0,438224936	permutation FDR (250 perm	1
2229	Nhlrc2~Q8B	Nhlrc2	ko vs wt	ko	wt	24,2252306	0,36130975	25,9231254	1,35894923	0,30823556	-1,6978948	-0,8560941	0,21540333	0,66674759	Unpaired t-te	0,105862163	permutation FDR (250 perm	1
2230	Nhp2~Q9CR	Nhp2	ko vs wt	ko	wt	25,4880208	1,30941367	26,3743142	0,2567155	0,54100227	-0,8862934	-0,498624	0,32941721	0,48225371	Unpaired t-te	0,273498218	permutation FDR (250 perm	1
2231	Niban1~Q3U	Niban1	ko vs wt	ko	wt	31,2780059	0,41837472	31,79715	0,35809014	0,69778568	-0,5191441	-0,3845674	0,20166344	0,69537283	Unpaired t-te	0,380283517	permutation FDR (250 perm	1
2232	Niban2~Q8R	Niban2	ko vs wt	ko	wt	30,4547735	0,53580299	29,8364401	0,41761371	1,53510085	0,61833344	0,43310885	0,20923589	0,67936381	Unpaired t-te	0,324525517	permutation FDR (250 perm	1
2233	Nid1~P1049	Nid1	ko vs wt	ko	wt	34,2416065	0,10824253	34,0667115	0,21027157	1,12888223	0,17489499	0,15060483	0,36505739	0,43763885	Unpaired t-te	0,740450099	permutation FDR (250 perm	1
2234	Nid2~O8832	Nid2	ko vs wt	ko	wt	32,9469874	0,2044	32,7607143	0,20957862	1,13782053	0,18627301	0,15660545	0,37852865	0,42190124	Unpaired t-te	0,72141605	permutation FDR (250 perm	1
2235	Nipbl~Q6KC	Nipbl	ko vs wt	ko	wt	25,1308934	1,12592012	24,0109881	1,57442054	2,17332702	1,11990527	0,48921841	0,44298587	0,35361013	Unpaired t-te	0,281346072	permutation FDR (250 perm	1
2236	Nipsnap1~O	Nipsnap1	ko vs wt	ko	wt	26,0099547	1,42051676	26,9638741	0,3351536	0,51622814	-0,9539193	-0,5146056	0,3348696	0,47512428	Unpaired t-te	0,259206934	permutation FDR (250 perm	1
2237	Nipsnap2~O	Nipsnap2	ko vs wt	ko	wt	27,9204457	0,35726816	28,8988642	0,58839733	0,50753581	-0,9784185	-0,6681434	0,12622704	0,89884761	Unpaired t-te	0,166384213	permutation FDR (250 perm	1

2238	Nipsnap3b~(	Nipsnap3b	ko vs wt	ko	wt	23,9690795	0,52737999	25,0053714	1,37318056	0,48757909	-1,0362918	-0,5136244	0,40008009	0,39785306	Unpaired t-te	0,27642847	permutation FDR (250 perm	1
2239	Nisch~Q80TN	Nisch	ko vs wt	ko	wt	27,1804763	0,43640413	24,7171872	1,74306542	5,5147254	2,46328905	1,0909053	0,17936188	0,74626986	Unpaired t-te	0,053059086	permutation FDR (250 perm	1
2240	Nit1~Q8VDK	Nit1	ko vs wt	ko	wt	24,6156297	0,62697767	24,8411565	0,79172582	0,85528263	-0,2255269	-0,1353159	0,75392914	0,12266947	Unpaired t-te	0,762802813	permutation FDR (250 perm	1
2241	Nkiras2~Q9C	Nkiras2	ko vs wt	ko	wt	24,4289613	1,19076936	23,9643631	0,37175358	1,37993295	0,46459817	0,26762062	0,56369698	0,24895429	Unpaired t-te	0,539935247	permutation FDR (250 perm	1
2242	Nlrx1~Q3TL4	Nlrx1	ko vs wt	ko	wt	27,2736096	1,26837797	26,2643056	0,46410996	2,01293973	1,00930398	0,5599557	0,27567108	0,5596088	Unpaired t-te	0,219336842	permutation FDR (250 perm	1
2243	Nmd3~Q99L	Nmd3	ko vs wt	ko	wt	24,3654973	0,31809387	25,0554402	2,34505873	0,61987839	-0,6899429	-0,2585658	0,71847831	0,14358634	Unpaired t-te	0,574731209	permutation FDR (250 perm	1
2244	Nmes1~Q81	Nmes1	ko vs wt	ko	wt	24,9493843	1,35425945	24,0708548	0,95574057	1,8385004	0,87852949	0,43203471	0,43420645	0,36230373	Unpaired t-te	0,325049293	permutation FDR (250 perm	1
2245	Nmral1~Q8K	Nmral1	ko vs wt	ko	wt	26,5333697	1,65713567	25,3713996	0,95548615	2,23762786	1,16197012	0,53516009	0,36752532	0,43471273	Unpaired t-te	0,232667162	permutation FDR (250 perm	1
2246	Nmt1~O703	Nmt1	ko vs wt	ko	wt	29,4146951	0,29050629	28,90464	0,15110172	1,42410459	0,5100551	0,42544843	0,05278934	1,27745373	Unpaired t-te	0,333386886	permutation FDR (250 perm	1
2247	Nmt2~O703	Nmt2	ko vs wt	ko	wt	24,5342159	1,13378378	24,8695821	1,26099243	0,79258289	-0,3353663	-0,1592329	0,77697668	0,10959201	Unpaired t-te	0,718194468	permutation FDR (250 perm	1
2248	Nnt~Q61941	Nnt	ko vs wt	ko	wt	30,8273392	0,30021391	28,0365098	2,37268718	6,92027496	2,79082936	1,03876788	0,23723442	0,6248223	Unpaired t-te	0,064190676	permutation FDR (250 perm	1
2249	Nomo1~Q6G	Nomo1	ko vs wt	ko	wt	28,2688639	0,44069712	28,3690524	0,10891849	0,93291108	-0,1001885	-0,079148	0,72780243	0,1379865	Unpaired t-te	0,86534607	permutation FDR (250 perm	1
2250	None~D3Z6I3		ko vs wt	ko	wt	25,3374977	0,79448264	23,4594256	0,98745877	3,67583508	1,87807204	1,02323439	0,0943445	1,02528341	Unpaired t-te	0,047484536	permutation FDR (250 perm	1
2251	None~E9PWG2		ko vs wt	ko	wt	28,0120743	0,81195318	27,9962858	0,29605766	1,01100384	0,01578847	0,01043244	0,97691492	0,01014326	Unpaired t-te	0,982505747	permutation FDR (250 perm	1
2252	None~E9PYB0		ko vs wt	ko	wt	27,9299332	0,59155332	28,0450687	0,48326369	0,92329556	-0,1151355	-0,07763	0,82147971	0,08540316	Unpaired t-te	0,86555786	permutation FDR (250 perm	1
2253	None~E9PZ54		ko vs wt	ko	wt	24,8122541	0,80647218	24,0035319	0,76345439	1,75165927	0,80872217	0,47213547	0,3140912	0,50294423	Unpaired t-te	0,287038421	permutation FDR (250 perm	1
2254	None~E9Q035		ko vs wt	ko	wt	32,8888221	0,3814252	32,2758573	0,2465144	1,52939892	0,61296476	0,47855892	0,08090803	1,09200836	Unpaired t-te	0,278262167	permutation FDR (250 perm	1
2255	None~E9Q474		ko vs wt	ko	wt	27,5390023	0,17769625	27,4632309	0,65191634	1,05392436	0,07577133	0,05146625	0,88607231	0,05253084	Unpaired t-te	0,915434992	permutation FDR (250 perm	1
2256	None~E9QKL6		ko vs wt	ko	wt	27,99826	1,07033681	26,0276074	0,85181079	3,91945379	1,97065261	1,05781895	0,07288145	1,137383	Unpaired t-te	0,036164115	permutation FDR (250 perm	1
2257	None~E9QKV6		ko vs wt	ko	wt	24,0517046	0,41022694	24,0985331	0,97310456	0,96806207	-0,0468285	-0,0271044	0,9535171	0,02067151	Unpaired t-te	0,953394785	permutation FDR (250 perm	1
2258	None~F6RT34		ko vs wt	ko	wt	24,853519	1,44432896	26,8538747	2,43647042	0,24993836	-2,0003558	-0,686454	0,37420179	0,42689414	Unpaired t-te	0,157815661	permutation FDR (250 perm	1
2259	None~F6TBV1		ko vs wt	ko	wt	23,6366106	0,90666182	25,1591059	0,98168654	0,34808336	-1,5224953	-0,8144275	0,15394011	0,81264819	Unpaired t-te	0,095511693	permutation FDR (250 perm	1
2260	None~F7DBB3		ko vs wt	ko	wt	30,5769578	0,13485634	31,3471259	0,39088431	0,58634915	-0,7701681	-0,5983497	0,09866205	1,00584987	Unpaired t-te	0,216846704	permutation FDR (250 perm	1
2261	None~J3QNY1		ko vs wt	ko	wt	32,7321815	0,39549094	32,7685053	0,12221482	0,97513655	-0,0363238	-0,0291959	0,88927795	0,05096248	Unpaired t-te	0,949376287	permutation FDR (250 perm	1
2262	None~P01630		ko vs wt	ko	wt	24,6337309	0,67290792	23,0901187	0,60275035	2,91523508	1,54361222	0,97901124	0,04801056	1,31866319	Unpaired t-te	0,050425676	permutation FDR (250 perm	1
2263	None~P01638		ko vs wt	ko	wt	27,5854038	1,04605953	27,1541647	0,65783054	1,3483912	0,43123912	0,24470098	0,59611462	0,22467023	Unpaired t-te	0,567571902	permutation FDR (250 perm	1
2264	None~P01645		ko vs wt	ko	wt	30,4079069	0,90197711	29,8528014	0,56508024	1,46927613	0,55510556	0,33513012	0,43644422	0,36007125	Unpaired t-te	0,437573265	permutation FDR (250 perm	1
2265	None~P01654		ko vs wt	ko	wt	29,115269	0,53248821	28,4835531	1,12296383	1,54940675	0,63171593	0,34119292	0,5193081	0,28457491	Unpaired t-te	0,451257179	permutation FDR (250 perm	1
2266	None~P01663		ko vs wt	ko	wt	27,2714171	0,6380184	26,5243117	0,43048595	1,6784219	0,74710541	0,50553262	0,17872059	0,74782541	Unpaired t-te	0,255698113	permutation FDR (250 perm	1
2267	None~P01670		ko vs wt	ko	wt	24,5016599	0,67194844	24,0672358	0,20271489	1,35137125	0,43442407	0,30732106	0,35615595	0,4483598	Unpaired t-te	0,482835793	permutation FDR (250 perm	1
2268	None~P01678		ko vs wt	ko	wt	26,0025527	2,29349309	24,8564311	0,74510296	2,21318126	1,14612161	0,4726046	0,46765366	0,33007566	Unpaired t-te	0,291466948	permutation FDR (250 perm	1
2269	None~P01799		ko vs wt	ko	wt	23,5338556	0,71389661	26,0939311	0,41715577	0,16956667	-2,5600755	-1,6989636	0,00410482	2,3867062	Unpaired t-te	0,004815013	permutation FDR (250 perm	1
2270	None~P03977		ko vs wt	ko	wt	25,9226812	1,04181203	24,6811458	1,23437781	2,36450044	1,24153541	0,60268225	0,30996674	0,5086849	Unpaired t-te	0,194177313	permutation FDR (250 perm	1
2271	None~P06330		ko vs wt	ko	wt	27,3138694	0,85262713	25,2736747	1,30786907	4,11301037	2,04019471	0,99635572	0,14292077	0,84490464	Unpaired t-te	0,056524769	permutation FDR (250 perm	1
2272	None~P10404		ko vs wt	ko	wt	26,9525564	0,46485381	25,2546475	0,71960021	3,24430359	1,69790883	1,0778488	0,05739369	1,24113586	Unpaired t-te	0,042796231	permutation FDR (250 perm	1
2273	None~P18528		ko vs wt	ko	wt	24,7092489	0,76793124	24,9764913	2,05684037	0,83090624	-0,2672424	-0,1060282	0,87435419	0,0583126	Unpaired t-te	0,82219116	permutation FDR (250 perm	1
2274	None~Q6PIU9		ko vs wt	ko	wt	24,8200047	0,85362014	24,5259522	0,22703244	1,22607948	0,29405251	0,19366899	0,60390526	0,21903119	Unpaired t-te	0,66046915	permutation FDR (250 perm	1
2275	None~Q8C3W1		ko vs wt	ko	wt	25,4677789	1,20874295	25,4773322	1,96723384	0,99340004	-0,0095533	-0,0037372	0,9954868	0,00196449	Unpaired t-te	0,993327088	permutation FDR (250 perm	1
2276	None~Q91V76		ko vs wt	ko	wt	24,250353	1,08399995	23,9452825	0,44644384	1,23547907	0,30507057	0,17935227	0,68435122	0,16472095	Unpaired t-te	0,681086291	permutation FDR (250 perm	1
2277	Nono~Q99K	Nono	ko vs wt	ko	wt	32,0937929	0,36749985	32,1035653	0,14395721	0,99324918	-0,0097724	-0,0079108	0,96877364	0,01377769	Unpaired t-te	0,986123598	permutation FDR (250 perm	1
2278	Nop10~Q9C	Nop10	ko vs wt	ko	wt	25,071089	1,61434235	26,4478488	0,22327023	0,38508271	-1,3767597	-0,7077301	0,23660538	0,62597538	Unpaired t-te	0,145843072	permutation FDR (250 perm	1
2279	Nop56~Q9D	Nop56	ko vs wt	ko	wt	29,9917684	0,43570152	29,6023407	0,45284597	1,3098736	0,3894276	0,27673898	0,39097606	0,40784984	Unpaired t-te	0,523113294	permutation FDR (250 perm	1
2280	Nop58~Q6D	Nop58	ko vs wt	ko	wt	30,2411993	0,40025936	29,3984145	0,60022591	1,79350878	0,8427848	0,56819847	0,17442541	0,75839024	Unpaired t-te	0,222773493	permutation FDR (250 perm	1
2281	Notch3~Q61	Notch3	ko vs wt	ko	wt	27,6849348	0,52742062	27,8344158	0,60573297	0,90157474	-0,149481	-0,0979864	0,79063057	0,1020264	Unpaired t-te	0,829989523	permutation FDR (250 perm	1
2282	Nox4~Q9JHI	Nox4	ko vs wt	ko	wt	27,6447703	0,23531236	27,4970114	0,56093082	1,10784722	0,14775894	0,10410984	0,75234015	0,12358576	Unpaired t-te	0,824646978	permutation FDR (250 perm	1
2283	Npc1~O3560	Npc1	ko vs wt	ko	wt	26,5300036	0,57507016	26,0176181	0,03812368	1,42640674	0,51238543	0,3843536	0,22045812	0,6566739	Unpaired t-te	0,394454215	permutation FDR (250 perm	1
2284	Npepps~Q11	Npepps	ko vs wt	ko	wt	25,4541535	0,53146122	23,7482991	0,15909805	3,26222053	1,70585431	1,28567847	0,00761503	2,11832862	Unpaired t-te	0,018179148	permutation FDR (250 perm	1
2285	Nploc4~P60	Nploc4	ko vs wt	ko	wt	26,062304	0,49434203	26,2094896	0,71796777	0,90301033	-0,1471856	-0,0930138	0,81564681	0,08849786	Unpaired t-te	0,840996703	permutation FDR (250 perm	1
2286	Npm1~Q619	Npm1	ko vs wt	ko	wt	29,5200775	0,5181077	28,6162051	0,71080807	1,8710815	0,9038724	0,57030415	0,2097396	0,67831957	Unpaired t-te	0,218606471	permutation FDR (250 perm	1
2287	Npnt~Q91V8	Npnt	ko vs wt	ko	wt	33,5448586	0,88223005	33,9677658	0,13801742	0,74592004	-0,4229071	-0,278481	0,47082695	0,32713869	Unpaired t-te	0,528338546	permutation FDR (250 perm	1
2288	Npnt~Q91V8	Npnt	ko vs wt	ko	wt	26,1454407	2,13035289	28,7294899	0,10904851	0,1667722	-2,5840493	-1,157534	0,1262438	0,89878996	Unpaired t-te	0,033521411	permutation FDR (250 perm	1
2289	Npr1~P1829	Npr1	ko vs wt	ko	wt	27,4996097	0,43122994	27,7819128	0,18791645	0,82227733	-0,282303	-0,220169	0,3687758	0,43323759	Unpaired t-te	0,60971192	permutation FDR (250 perm	1
2290	Npr2~Q6VV	Npr2	ko vs wt	ko	wt	25,8109859	0,39526196	25,904149	0,23559376	0,93746515	-0,093163	-0,0726394	0,75511448	0,1219872	Unpaired t-te	0,875253813	permutation FDR (250 perm	1
2291	Nptn~H3BIX	Nptn	ko vs wt	ko	wt	27,8919868	0,01877735	27,7110766	0,09414997	1,13359886	0,18091021	0,1694787	0,10930498	0,96136003	Unpaired t-te	0,71629012	permutation FDR (250 perm	1
2292	Nqo1~Q646	Nqo1	ko vs wt	ko	wt	24,6356135	1,17434184	23,9851485	0,99302517	1,56967404	0,650465	0,32916849	0,53628168	0,27060704	Unpaired t-te	0,446591243	permutation FDR (250 perm	1
2293	Nr3c1~E9PU	Nr3c1	ko vs wt	ko	wt	25,0857855	0,45303459	25,8306128	0,27486854	0,59673932	-0,7448273	-0,5617659	0,07146194	1,1459252	Unpaired t-te	0,212700719	permutation FDR (250 perm	1
2294	Nrbp1~Q99J	Nrbp1	ko vs wt	ko	wt	26,6220507	0,92494355	25,0342511	1,25402598	3,00590546	1,58779964	0,78020124	0,21168308	0,67431385	Unpaired t-te	0,113375842	permutation FDR (250 perm	1
2295	Nrbp2~Q91V	Nrbp2	ko vs wt	ko	wt	25,017494	0,84313218	26,0865462	0,40579868	0,47663202	-1,0690522	-0,683074	0,12193957	0,91385533	Unpaired t-te	0,145610021	permutation FDR (250 perm	1
2296	Nrdc~Q8BH	Nrdc	ko vs wt	ko	wt	25,0994159	0,56278284	25,9651741	0,44118474	0,54875792	-0,8657582	-0,5968932	0,11416184	0,94247903	Unpaired t-te	0,190157915	permutation FDR (250 perm	1
2297	Nrp1~P9733	Nrp1	ko vs wt	ko	wt	27,0018861	1,03191838	25,0943506	1,48862825	3,7516767	1,90753551	0,86332248	0,20558025	0,68701862	Unpaired t-te	0,086825565	permutation FDR (250 perm	1
2298	Nsfl1c~Q9CZ	Nsfl1c	ko vs wt	ko	wt	26,3283563	0,36161135	27,2524686	0,75023722	0,52700467	-0,9241123	-0,5885686	0,2161811	0,66518228	Unpaired t-te	0,216940113	permutation FDR (250 perm	1
2299	Nsf~P46460	Nsf	ko vs wt	ko	wt	30,0051725	0,44396951	29,7737913	0,05029068	1,1739583	0,23138116	0,18381369	0,43492251	0,36158811	Unpaired t-te	0,680480508	permutation FDR (250 perm	1
2300	Nsun2~Q1HF	Nsun2	ko vs wt	ko	wt	25,4293772	1,005916	23,4058031	0,30724131	4,06589826	2,02357412	1,24906535	0,03355482	1,47424502	Unpaired t-te	0,020229624	permutation FDR (250 perm	1
2301	Nt5c2~Q3V1	Nt5c2	ko vs wt	ko	wt	25,9278306	1,17351545	24,2178389	0,26806845	3,27158941	1,7099917	1,00378389	0,08198615	1,0862595	Unpaired t-te	0,052440223	permutation FDR (250 perm	1

2302	Nt5c3a~Q9D	Nt5c3a	ko vs wt	ko	wt	25,8187977	2,22878871	24,4596422	0,22307394	2,56534976	1,35915554	0,59185751	0,36944655	0,43244838	Unpaired t-te	0,208630377	permutation FDR (250 perm	1
2303	Nt5c3b~Q3U	Nt5c3b	ko vs wt	ko	wt	23,9658067	0,48550304	24,834647	1,54243267	0,54758686	-0,8688403	-0,4086528	0,51271257	0,29012603	Unpaired t-te	0,381478464	permutation FDR (250 perm	1
2304	Nt5c~Q9JM1	Nt5c	ko vs wt	ko	wt	25,9335671	1,2658639	24,3009342	0,77627597	3,10078379	1,63263293	0,8529847	0,1344965	0,87128901	Unpaired t-te	0,078819802	permutation FDR (250 perm	1
2305	Nt5dc2~Q91	Nt5dc2	ko vs wt	ko	wt	24,7985019	0,50405622	24,2500893	0,84395297	1,46247558	0,54841253	0,32958629	0,47024457	0,32767621	Unpaired t-te	0,460980006	permutation FDR (250 perm	1
2306	Nt5e~Q6150	Nt5e	ko vs wt	ko	wt	25,6348765	1,87226622	27,4920708	0,08889929	0,27601254	-1,8571943	-0,8916902	0,18420135	0,73470718	Unpaired t-te	0,081319024	permutation FDR (250 perm	1
2307	Ntn1~O0911	Ntn1	ko vs wt	ko	wt	28,8260776	1,34983873	30,0610423	0,17279731	0,42485288	-1,2349648	-0,690368	0,21124927	0,67520478	Unpaired t-te	0,153207888	permutation FDR (250 perm	1
2308	Ntn4~Q9JI33	Ntn4	ko vs wt	ko	wt	27,2002517	0,47778313	25,8019823	1,44927509	2,63585204	1,39826939	0,67835375	0,30388429	0,51729175	Unpaired t-te	0,173235552	permutation FDR (250 perm	1
2309	Ntpcr~D3YXL	Ntpcr	ko vs wt	ko	wt	25,4790438	0,7203014	24,0998855	1,11276259	2,60116573	1,37915832	0,72972099	0,21579144	0,66596578	Unpaired t-te	0,136945176	permutation FDR (250 perm	1
2310	Nub1~P5472	Nub1	ko vs wt	ko	wt	26,1328684	1,00864076	26,6570927	0,21626014	0,69533284	-0,5242244	-0,3272144	0,44100148	0,35555995	Unpaired t-te	0,458794538	permutation FDR (250 perm	1
2311	Nubpl~Q9CW	Nubpl	ko vs wt	ko	wt	24,8455559	0,95415409	24,2809261	0,57441617	1,47900792	0,56462978	0,33520488	0,44729028	0,34941054	Unpaired t-te	0,437673522	permutation FDR (250 perm	1
2312	Nucb1~Q028	Nucb1	ko vs wt	ko	wt	24,8689562	0,68058651	24,3498312	0,21654284	1,43308585	0,51912504	0,36513965	0,28823439	0,54025421	Unpaired t-te	0,408221712	permutation FDR (250 perm	1
2313	Nucb2~P811	Nucb2	ko vs wt	ko	wt	24,0691434	0,73192004	23,6535486	0,32091681	1,33384848	0,4155948	0,28087398	0,43068089	0,3658444	Unpaired t-te	0,516251541	permutation FDR (250 perm	1
2314	Nudc~O3568	Nudc	ko vs wt	ko	wt	27,8107718	0,48349469	28,22408	0,16534081	0,75089953	-0,4133082	-0,317285	0,24443313	0,61183994	Unpaired t-te	0,467065444	permutation FDR (250 perm	1
2315	Nudt21~Q9C	Nudt21	ko vs wt	ko	wt	27,5863128	0,2040505	27,4543978	0,10655031	1,09574721	0,131915	0,11573111	0,39087799	0,40795879	Unpaired t-te	0,795575893	permutation FDR (250 perm	1
2316	Nudt4~Q8R2	Nudt4	ko vs wt	ko	wt	27,791714	0,77477081	24,9307959	1,98368516	7,26477487	2,86091809	1,15720064	0,16929689	0,77135103	Unpaired t-te	0,039242533	permutation FDR (250 perm	1
2317	Nudt5~Q9JK	Nudt5	ko vs wt	ko	wt	25,0619629	0,82738057	24,167184	0,18107839	1,85932492	0,8947789	0,59869404	0,156707	0,80491159	Unpaired t-te	0,200164363	permutation FDR (250 perm	1
2318	Numa1~E9Q	Numa1	ko vs wt	ko	wt	28,5377025	1,00325572	28,9109126	0,9318972	0,77206266	-0,3732102	-0,1987974	0,69013828	0,16106388	Unpaired t-te	0,646206776	permutation FDR (250 perm	1
2319	Numbl~O08	Numbl	ko vs wt	ko	wt	26,3993706	1,22596538	27,419907	0,55933842	0,49293303	-1,0205364	-0,5635774	0,26941458	0,56957891	Unpaired t-te	0,21391232	permutation FDR (250 perm	1
2320	Numb~Q9Q2	Numb	ko vs wt	ko	wt	26,9915755	0,33657649	26,7446681	0,2809711	1,18666068	0,24690746	0,19321202	0,41696942	0,3798958	Unpaired t-te	0,655271562	permutation FDR (250 perm	1
2321	Nup107~Q8E	Nup107	ko vs wt	ko	wt	27,1021069	0,32721674	26,7289081	0,4801677	1,29522142	0,37319874	0,26876876	0,40316993	0,39451186	Unpaired t-te	0,542960073	permutation FDR (250 perm	1
2322	Nup133~Q8F	Nup133	ko vs wt	ko	wt	27,4008845	0,38861218	27,4454354	0,30716262	0,96959159	-0,0445509	-0,0339494	0,89228103	0,04949834	Unpaired t-te	0,941005302	permutation FDR (250 perm	1
2323	Nup153~E9C	Nup153	ko vs wt	ko	wt	25,5343905	1,16730895	24,9406385	1,12833478	1,50916647	0,59375195	0,29042835	0,59755504	0,22362208	Unpaired t-te	0,502706431	permutation FDR (250 perm	1
2324	Nup155~Q9	Nup155	ko vs wt	ko	wt	28,5958181	0,48322853	28,6665043	0,45548024	0,95218503	-0,0706861	-0,0495658	0,87550559	0,05774108	Unpaired t-te	0,914473487	permutation FDR (250 perm	1
2325	Nup160~Q92	Nup160	ko vs wt	ko	wt	25,6128088	1,50103826	25,5675035	1,07912984	1,03190152	0,04530529	0,0210264	0,97022886	0,01312581	Unpaired t-te	0,96298633	permutation FDR (250 perm	1
2326	Nup205~B9E	Nup205	ko vs wt	ko	wt	27,0217562	0,27703142	26,4152335	0,99702996	1,52258693	0,60652271	0,35203139	0,48251976	0,3164849	Unpaired t-te	0,446002559	permutation FDR (250 perm	1
2327	Nup210~Q9C	Nup210	ko vs wt	ko	wt	25,0210211	0,74385424	24,8392296	0,38924179	1,13429155	0,18179151	0,12038436	0,73678353	0,13266009	Unpaired t-te	0,786772842	permutation FDR (250 perm	1
2328	Nup214~Q8C	Nup214	ko vs wt	ko	wt	24,436453	1,51470553	26,4456309	0,32691105	0,24841465	-2,0091778	-1,054935	0,1021845	0,99061499	Unpaired t-te	0,043698246	permutation FDR (250 perm	1
2329	Nup54~Q8B	Nup54	ko vs wt	ko	wt	26,6521156	0,23327361	26,6783018	0,28347626	0,98201287	-0,0261862	-0,0210925	0,91925974	0,03656176	Unpaired t-te	0,963508016	permutation FDR (250 perm	1
2330	Nup62~Q63	Nup62	ko vs wt	ko	wt	24,7407294	1,41764841	23,8361662	0,34440974	1,87197764	0,9045632	0,48791326	0,35679626	0,4475797	Unpaired t-te	0,280447434	permutation FDR (250 perm	1
2331	Nup85~Q8R	Nup85	ko vs wt	ko	wt	24,2933968	0,6824001	26,6571449	0,24504449	0,19428575	-2,363748	-1,652505	0,00518173	2,2855252	Unpaired t-te	0,006188235	permutation FDR (250 perm	1
2332	Nup88~Q8C	Nup88	ko vs wt	ko	wt	25,041076	1,17329096	25,9732579	0,13785396	0,52406517	-0,9321819	-0,5534281	0,26308361	0,57990621	Unpaired t-te	0,233921365	permutation FDR (250 perm	1
2333	Nup93~Q8BJ	Nup93	ko vs wt	ko	wt	27,468776	0,62093065	27,3243843	0,53469261	1,10526452	0,14439169	0,09493056	0,79348286	0,10046245	Unpaired t-te	0,83385734	permutation FDR (250 perm	1
2334	Nup98~Q6PF	Nup98	ko vs wt	ko	wt	27,565099	0,78758265	27,6754037	0,33329103	0,92639238	-0,1103047	-0,0729453	0,83918818	0,07614064	Unpaired t-te	0,87542826	permutation FDR (250 perm	1
2335	Nutf2~P6197	Nutf2	ko vs wt	ko	wt	26,6209809	0,87577621	26,2719361	0,46125117	1,27371698	0,34904475	0,21792202	0,58813351	0,23052408	Unpaired t-te	0,612018918	permutation FDR (250 perm	1
2336	Nxf1~Q99JX	Nxf1	ko vs wt	ko	wt	27,6578023	0,27497212	27,8534377	0,28132177	0,87318823	-0,1956354	-0,155946	0,48286581	0,31617354	Unpaired t-te	0,722503556	permutation FDR (250 perm	1
2337	Oas1a~P119	Oas1a	ko vs wt	ko	wt	26,213746	1,96762918	24,8818582	1,27887975	2,51731858	1,33188781	0,54318546	0,40110035	0,39676496	Unpaired t-te	0,226333333	permutation FDR (250 perm	1
2338	Oas3~Q8VI9	Oas3	ko vs wt	ko	wt	24,4690742	0,5537869	23,7032027	0,24874278	1,70039681	0,76587146	0,56111284	0,09641652	1,01584853	Unpaired t-te	0,21578725	permutation FDR (250 perm	1
2339	Oat~P29758	Oat	ko vs wt	ko	wt	28,1921277	0,50779308	25,4712888	0,66204607	6,59256072	2,72083895	1,75270908	0,01093414	1,96121531	Unpaired t-te	0,005706535	permutation FDR (250 perm	1
2340	Obscn~A2AA	Obscn	ko vs wt	ko	wt	24,4978315	1,34257752	24,4645748	0,14248741	1,02331951	0,03325666	0,01866613	0,96865544	0,01383068	Unpaired t-te	0,968705512	permutation FDR (250 perm	1
2341	Ocrl~Q6NVF	Ocrl	ko vs wt	ko	wt	24,8060328	0,57591445	24,9840813	0,20715749	0,88389781	-0,1780485	-0,1305973	0,64948054	0,18743386	Unpaired t-te	0,769191585	permutation FDR (250 perm	1
2342	Odf2~A3KGV	Odf2	ko vs wt	ko	wt	24,2148921	0,94582746	24,6888791	0,22234692	0,71997215	-0,473987	-0,3022382	0,4575956	0,33951816	Unpaired t-te	0,492150967	permutation FDR (250 perm	1
2343	Ogdh~Q605	Ogdh	ko vs wt	ko	wt	32,1089469	0,25302012	31,8064162	0,133845	1,23330591	0,30253069	0,25767902	0,14535172	0,83757982	Unpaired t-te	0,549006075	permutation FDR (250 perm	1
2344	Ogn~Q6200	Ogn	ko vs wt	ko	wt	35,3632376	0,33839834	35,2147636	0,20784989	1,10839649	0,14847405	0,11930581	0,57033123	0,24387284	Unpaired t-te	0,788482816	permutation FDR (250 perm	1
2345	Ogt~Q8CGY	Ogt	ko vs wt	ko	wt	28,0654968	0,45919998	27,9004369	0,08040976	1,12121264	0,16505991	0,12985109	0,58237569	0,23479676	Unpaired t-te	0,774224215	permutation FDR (250 perm	1
2346	Ola1~Q9CZ3	Ola1	ko vs wt	ko	wt	30,2259219	0,41343924	30,4812736	0,25882209	0,83778287	-0,2553517	-0,1963058	0,43479653	0,36171393	Unpaired t-te	0,648247375	permutation FDR (250 perm	1
2347	Olfml2a~Q8	Olfml2a	ko vs wt	ko	wt	24,5304427	0,63121819	24,0839166	1,24204022	1,36275491	0,44652611	0,22888623	0,67399149	0,17134559	Unpaired t-te	0,608820118	permutation FDR (250 perm	1
2348	Olfml2b~Q3	Olfml2b	ko vs wt	ko	wt	25,9216202	1,73795318	29,0018222	0,89620727	0,11824065	-3,0802019	-1,408563	0,05107768	1,29176884	Unpaired t-te	0,010664292	permutation FDR (250 perm	1
2349	Olfml3~Q8B	Olfml3	ko vs wt	ko	wt	27,9953987	0,80386646	26,9863244	1,09686747	2,01261932	1,00907432	0,53001549	0,33690848	0,47248806	Unpaired t-te	0,248106647	permutation FDR (250 perm	1
2350	Omd~O3510	Omd	ko vs wt	ko	wt	29,348567	0,04805464	28,4011213	1,09702119	1,92845534	0,94744574	0,53340949	0,34634253	0,46049417	Unpaired t-te	0,270859392	permutation FDR (250 perm	1
2351	Opa1~P5828	Opa1	ko vs wt	ko	wt	29,8264148	0,29009759	29,6005698	0,15849812	1,16946199	0,22584497	0,187965	0,31499162	0,50170099	Unpaired t-te	0,664349898	permutation FDR (250 perm	1
2352	Opa3~Q505	Opa3	ko vs wt	ko	wt	25,0785096	0,72906381	23,9558023	0,71511093	2,17755207	1,12270722	0,67717471	0,15745744	0,80283682	Unpaired t-te	0,149596353	permutation FDR (250 perm	1
2353	Ophn1~Q99J	Ophn1	ko vs wt	ko	wt	24,504603	1,36092723	24,7060884	1,31197795	0,86965469	-0,2014854	-0,0909338	0,87572217	0,05763366	Unpaired t-te	0,841407306	permutation FDR (250 perm	1
2354	Oplah~Q8K0	Oplah	ko vs wt	ko	wt	26,2308226	0,73770136	27,3533192	0,47932443	0,45929831	-1,1224966	-0,726858	0,09420152	1,0259421	Unpaired t-te	0,124189225	permutation FDR (250 perm	1
2355	Osbpl11~Q8	Osbpl11	ko vs wt	ko	wt	26,9213023	0,67378064	25,4519175	1,07475805	2,76903781	1,46938475	0,79265776	0,18233239	0,73913619	Unpaired t-te	0,112693933	permutation FDR (250 perm	1
2356	Osbpl3~D3Y	Osbpl3	ko vs wt	ko	wt	27,8673702	0,62400715	27,9170702	0,29768659	0,96613719	-0,0497	-0,0350678	0,91021747	0,04085483	Unpaired t-te	0,939089655	permutation FDR (250 perm	1
2357	Osbpl5~Q9E	Osbpl5	ko vs wt	ko	wt	25,2234903	0,21287608	24,4981948	0,83852765	1,65323916	0,72529545	0,45174757	0,34530041	0,4618029	Unpaired t-te	0,338992816	permutation FDR (250 perm	1
2358	Osbpl8~B9EJ	Osbpl8	ko vs wt	ko	wt	28,2466075	1,00375649	27,5526407	0,33302449	1,61772551	0,69396683	0,42691575	0,33075003	0,48050011	Unpaired t-te	0,337786448	permutation FDR (250 perm	1
2359	Osbpl9~A2A	Osbpl9	ko vs wt	ko	wt	27,6450281	0,17990218	27,3510987	0,24449815	1,22597483	0,29392936	0,24459713	0,23052237	0,63728693	Unpaired t-te	0,577455092	permutation FDR (250 perm	1
2360	Osbpl9~Q5F	Osbpl9	ko vs wt	ko	wt	23,9698303	0,96917735	24,068533	0,56739329	0,9338724	-0,0987027	-0,0584549	0,89169136	0,04978544	Unpaired t-te	0,897685968	permutation FDR (250 perm	1
2361	Osbp~Q3B7	Osbp	ko vs wt	ko	wt	28,0435753	0,24408326	27,8331873	1,03910808	1,15699931	0,210388	0,12034879	0,8033477	0,09509645	Unpaired t-te	0,800877614	permutation FDR (250 perm	1
2362	Osmr~O704	Osmr	ko vs wt	ko	wt	25,0826557	0,37410435	24,0919094	0,5183793	1,9872127	0,9907463	0,69503981	0,09288488	1,03205497	Unpaired t-te	0,149221577	permutation FDR (250 perm	1
2363	Ostc~Q78XF	Ostc	ko vs wt	ko	wt	25,1780245	1,60883182	26,0894371	0,78615225	0,53166427	-0,9114126	-0,4376539	0,4410276	0,35553423	Unpaired t-te	0,3212868	permutation FDR (250 perm	1
2364	Ostf1~Q624	Ostf1	ko vs wt	ko	wt	27,7099411	0,63062205	26,710495	0,51871036	1,99923233	0,99944613	0,65891361	0,11351687	0,9449396	Unpaired t-te	0,155785597	permutation FDR (250 perm	1
2365	Otub1~Q7TC	Otub1	ko vs wt	ko	wt	29,1377382	0,42176373	28,7846376	0,64237786	1,27730286	0,35310064	0,23301084	0,54041013	0,26727652	Unpaired t-te	0,597276241	permutation FDR (250 perm	1

2366	Otud7b~B2R	Otud7b	ko vs wt	ko	wt	26,0388584	1,01197423	24,1843295	0,42532368	3,61633649	1,85452892	1,11912393	0,04342045	1,36230569	Unpaired t-te	0,030645161	permutation FDR (250 perm	1
2367	Oxct1~Q9D0	Oxct1	ko vs wt	ko	wt	29,1122026	0,56463656	29,444898	0,32755727	0,79405153	-0,3326955	-0,2376569	0,44396695	0,35264936	Unpaired t-te	0,578847722	permutation FDR (250 perm	1
2368	Oxnad1~Q8V	Oxnad1	ko vs wt	ko	wt	25,2425924	0,84414518	24,2813374	0,74881308	1,94700281	0,96125497	0,55898485	0,24370352	0,6131382	Unpaired t-te	0,216242744	permutation FDR (250 perm	1
2369	Oxsr1~Q6P9	Oxsr1	ko vs wt	ko	wt	28,6084103	0,26793297	28,6035748	0,07105722	1,00335727	0,00483541	0,00415897	0,977854	0,00972599	Unpaired t-te	0,992770633	permutation FDR (250 perm	1
2370	P2rx1~P5157	P2rx1	ko vs wt	ko	wt	26,0065168	1,93876026	24,9111871	0,69587068	2,136619	1,09532967	0,49278758	0,42046486	0,3762703	Unpaired t-te	0,271625714	permutation FDR (250 perm	1
2371	P2rx4~Q9JJX	P2rx4	ko vs wt	ko	wt	24,7659835	1,16061142	24,8852656	0,53792772	0,92064571	-0,119282	-0,0673715	0,88361833	0,05373528	Unpaired t-te	0,884944942	permutation FDR (250 perm	1
2372	P3h1~A6PW	P3h1	ko vs wt	ko	wt	27,2087673	2,05242289	27,4833509	1,06220851	0,82668888	-0,2745836	-0,114269	0,85293308	0,06908504	Unpaired t-te	0,798906555	permutation FDR (250 perm	1
2373	P3h4~Q8K2B	P3h4	ko vs wt	ko	wt	25,6025461	1,93419757	24,648615	0,79140309	1,93714379	0,95393104	0,42414288	0,48488991	0,31435686	Unpaired t-te	0,337782341	permutation FDR (250 perm	1
2374	P4ha1~Q607	P4ha1	ko vs wt	ko	wt	29,273666	0,77903827	27,4977105	0,60510214	3,42464768	1,77595557	1,09573765	0,03633134	1,4397186	Unpaired t-te	0,03171885	permutation FDR (250 perm	1
2375	P4ha2~Q5SX	P4ha2	ko vs wt	ko	wt	26,5352771	0,23980071	25,9990157	0,42986325	1,4502095	0,53626133	0,40199349	0,21194873	0,67376918	Unpaired t-te	0,377862115	permutation FDR (250 perm	1
2376	P4hb~P0910	P4hb	ko vs wt	ko	wt	32,8681365	0,36338855	32,727083	0,20710927	1,10271002	0,14105345	0,11231624	0,60565844	0,21777223	Unpaired t-te	0,80202842	permutation FDR (250 perm	1
2377	Pa2g4~P505	Pa2g4	ko vs wt	ko	wt	30,2927529	0,28978334	29,9889205	0,2957672	1,23441916	0,30383237	0,23964838	0,31683826	0,49916238	Unpaired t-te	0,579028777	permutation FDR (250 perm	1
2378	Pabpc1~P29	Pabpc1	ko vs wt	ko	wt	31,2895697	0,141432	31,0670015	0,21645605	1,16680885	0,22256823	0,18966561	0,28621339	0,54331005	Unpaired t-te	0,671170293	permutation FDR (250 perm	1
2379	Pabpc4~Q6P	Pabpc4	ko vs wt	ko	wt	27,8917169	0,43710655	27,569035	0,16247568	1,25065332	0,32268193	0,25263126	0,30764525	0,51194979	Unpaired t-te	0,559727932	permutation FDR (250 perm	1
2380	Pacs1~Q8K2	Pacs1	ko vs wt	ko	wt	26,8002153	0,26241376	26,5316941	0,13703136	1,20457249	0,26852122	0,2275912	0,19848243	0,70227794	Unpaired t-te	0,595691667	permutation FDR (250 perm	1
2381	Pacsin2~Q9V	Pacsin2	ko vs wt	ko	wt	31,1029081	0,60016089	31,1842144	0,29914439	0,94520142	-0,0813063	-0,0578295	0,84967523	0,07074704	Unpaired t-te	0,899483037	permutation FDR (250 perm	1
2382	Pacsin3~Q99	Pacsin3	ko vs wt	ko	wt	25,4245238	1,00887395	25,1223419	0,91394915	1,23300776	0,30218188	0,16159324	0,74378627	0,12855184	Unpaired t-te	0,711831856	permutation FDR (250 perm	1
2383	Pafah1b1~P6	Pafah1b1	ko vs wt	ko	wt	30,1649356	0,48919518	29,936881	0,23372531	1,17125456	0,22805466	0,17182656	0,5195064	0,2844091	Unpaired t-te	0,692878161	permutation FDR (250 perm	1
2384	Paics~Q9DCL	Paics	ko vs wt	ko	wt	28,4219841	0,4334481	28,5231567	0,72497802	0,93227499	-0,1011725	-0,0644223	0,87068959	0,06013665	Unpaired t-te	0,891513908	permutation FDR (250 perm	1
2385	Pak1ip1~Q9D	Pak1ip1	ko vs wt	ko	wt	24,6832023	0,15576093	25,008676	2,36292605	0,79803634	-0,3254736	-0,1217516	0,86364837	0,06366304	Unpaired t-te	0,800227615	permutation FDR (250 perm	1
2386	Pak2~Q8CIN	Pak2	ko vs wt	ko	wt	28,9307658	0,58153653	28,6325335	0,45887342	1,22963685	0,29823231	0,20330564	0,55187498	0,2581593	Unpaired t-te	0,63714269	permutation FDR (250 perm	1
2387	Pak4~Q8BTV	Pak4	ko vs wt	ko	wt	23,9355509	0,60794398	24,6851848	0,22093786	0,59475445	-0,7496339	-0,5415669	0,12162138	0,91499006	Unpaired t-te	0,233171492	permutation FDR (250 perm	1
2388	Pald1~P7026	Pald1	ko vs wt	ko	wt	24,9968679	0,84947182	25,173735	0,71360807	0,88462191	-0,1768671	-0,1038153	0,81206966	0,09040671	Unpaired t-te	0,816862441	permutation FDR (250 perm	1
2389	Palld~Q9ET5	Palld	ko vs wt	ko	wt	31,2489703	0,25094887	31,3910123	0,22162134	0,90623555	-0,142042	-0,1170588	0,53743362	0,26967517	Unpaired t-te	0,793134245	permutation FDR (250 perm	1
2390	Palm~Q9Z0P	Palm	ko vs wt	ko	wt	24,9042127	1,6055965	26,6623576	0,22128135	0,29562805	-1,7581449	-0,9062116	0,15328761	0,81449295	Unpaired t-te	0,076015928	permutation FDR (250 perm	1
2391	Pals2~Q9JLB	Pals2	ko vs wt	ko	wt	27,4087097	0,16721206	27,1303299	0,21971	1,21283201	0,27837973	0,2353346	0,21265068	0,67233322	Unpaired t-te	0,590362064	permutation FDR (250 perm	1
2392	Pamr1~Q8B	Pamr1	ko vs wt	ko	wt	27,2426339	0,61994977	26,9608132	0,57044358	1,21572813	0,28182064	0,18308683	0,62583427	0,20354066	Unpaired t-te	0,675290229	permutation FDR (250 perm	1
2393	Pam~P97467	Pam	ko vs wt	ko	wt	27,3612417	1,04738133	27,3275789	0,44348144	1,0236076	0,03366277	0,02002329	0,96276923	0,0164778	Unpaired t-te	0,965037037	permutation FDR (250 perm	1
2394	Pan2~Q8BG	Pan2	ko vs wt	ko	wt	24,3948845	0,95330469	24,4797386	0,60889535	0,94287985	-0,0848542	-0,0499498	0,90808735	0,04187237	Unpaired t-te	0,913595283	permutation FDR (250 perm	1
2395	Papln~Q9EP	Papln	ko vs wt	ko	wt	27,5682427	0,62288313	27,9463294	0,23963632	0,76945734	-0,3780867	-0,2705367	0,39336133	0,40520834	Unpaired t-te	0,532764004	permutation FDR (250 perm	1
2396	Papola~Q61	Papola	ko vs wt	ko	wt	25,0239543	0,7850196	24,8666938	0,91018149	1,11516758	0,15726053	0,08799425	0,85177035	0,06967748	Unpaired t-te	0,848560768	permutation FDR (250 perm	1
2397	Papss2~O88	Papss2	ko vs wt	ko	wt	26,9153055	0,40904163	26,7939744	0,1228811	1,08773799	0,12133109	0,09693786	0,65637699	0,18284665	Unpaired t-te	0,83246832	permutation FDR (250 perm	1
2398	Pard3~Q99N	Pard3	ko vs wt	ko	wt	25,3953254	0,54567607	25,0270592	0,93949053	1,29080062	0,36826618	0,21222801	0,65195962	0,1857793	Unpaired t-te	0,635089841	permutation FDR (250 perm	1
2399	Park7~Q99L	Park7	ko vs wt	ko	wt	25,2341962	1,30757268	24,5840092	0,66544627	1,56937159	0,65018699	0,34409359	0,49951023	0,30145561	Unpaired t-te	0,42847767	permutation FDR (250 perm	1
2400	Parn~Q8VDG	Parn	ko vs wt	ko	wt	24,8113548	0,99284736	24,6712988	0,17201267	1,10194787	0,14005598	0,08830862	0,82532044	0,0833774	Unpaired t-te	0,850126749	permutation FDR (250 perm	1
2401	Parp12~Q8B	Parp12	ko vs wt	ko	wt	27,6604925	0,84414441	26,9987314	1,09137729	1,58201262	0,6617611	0,345977	0,51345279	0,28949948	Unpaired t-te	0,434291425	permutation FDR (250 perm	1
2402	Parp14~Q2E	Parp14	ko vs wt	ko	wt	26,3114131	0,21432982	26,418151	0,07640624	0,92868559	-0,1067378	-0,0940402	0,4731183	0,32503026	Unpaired t-te	0,837294441	permutation FDR (250 perm	1
2403	Parp1~Q921	Parp1	ko vs wt	ko	wt	29,7934287	0,29162043	29,6358975	0,23658249	1,11537686	0,15753125	0,12731382	0,53733691	0,26975333	Unpaired t-te	0,773105691	permutation FDR (250 perm	1
2404	Parp3~Q8CF	Parp3	ko vs wt	ko	wt	28,7775193	0,45181423	29,2470166	0,46189663	0,72221621	-0,4694973	-0,3310995	0,32114524	0,49329851	Unpaired t-te	0,447192074	permutation FDR (250 perm	1
2405	Parp4~E9PY	Parp4	ko vs wt	ko	wt	27,4120061	0,30389633	27,6812356	0,00818247	0,82976256	-0,2692295	-0,2290243	0,22247107	0,65272645	Unpaired t-te	0,604806872	permutation FDR (250 perm	1
2406	Parp9~Q8CA	Parp9	ko vs wt	ko	wt	26,8657536	0,74064045	26,0770804	0,28336225	1,72748505	0,78867322	0,53570153	0,16744678	0,77612319	Unpaired t-te	0,236897597	permutation FDR (250 perm	1
2407	Parva~Q9EP	Parva	ko vs wt	ko	wt	31,9759987	0,47630292	32,558684	0,17418183	0,66771979	-0,5826853	-0,4477663	0,12390311	0,90691778	Unpaired t-te	0,315350713	permutation FDR (250 perm	1
2408	Parvb~Q9ES	Parvb	ko vs wt	ko	wt	25,5966436	2,30135466	25,1486865	0,96619908	1,36410725	0,44795707	0,1796104	0,77812411	0,10895113	Unpaired t-te	0,680673405	permutation FDR (250 perm	1
2409	Pate4~Q090	Pate4	ko vs wt	ko	wt	25,2374701	1,51535877	24,5284276	0,24521126	1,63471883	0,70904251	0,37477622	0,48086889	0,31797332	Unpaired t-te	0,402423924	permutation FDR (250 perm	1
2410	Pawr~Q925B	Pawr	ko vs wt	ko	wt	26,0096929	1,41980983	28,6801365	0,3511398	0,15707836	-2,6704437	-1,4384243	0,04217183	1,37497754	Unpaired t-te	0,011932261	permutation FDR (250 perm	1
2411	Pbrm1~Q8B	Pbrm1	ko vs wt	ko	wt	25,2385956	0,60325726	23,9686987	1,08430005	2,4114434	1,26989695	0,68936853	0,23393703	0,63090103	Unpaired t-te	0,157986949	permutation FDR (250 perm	1
2412	Pbxip1~Q3T	Pbxip1	ko vs wt	ko	wt	25,3251302	1,23348813	27,3695609	0,37112664	0,24241809	-2,0444307	-1,1622902	0,05806573	1,23608011	Unpaired t-te	0,028173913	permutation FDR (250 perm	1
2413	Pcbp1~P603	Pcbp1	ko vs wt	ko	wt	31,7241639	0,28921767	31,7121427	0,2536495	1,00836727	0,01202119	0,00965516	0,96291436	0,01641234	Unpaired t-te	0,983773309	permutation FDR (250 perm	1
2414	Pcbp2~Q619	Pcbp2	ko vs wt	ko	wt	29,9721917	0,45973074	30,4557764	0,10243412	0,71519836	-0,4835847	-0,3792431	0,16531559	0,7816862	Unpaired t-te	0,395322772	permutation FDR (250 perm	1
2415	Pcca~Q91ZA	Pcca	ko vs wt	ko	wt	28,1817028	0,54431069	28,916172	0,40335818	0,60103912	-0,7344692	-0,5156377	0,14472305	0,83946229	Unpaired t-te	0,24752924	permutation FDR (250 perm	1
2416	Pccb~Q99M	Pccb	ko vs wt	ko	wt	27,1828026	0,62434245	26,6234168	0,82539306	1,47364167	0,55938576	0,33178631	0,46692978	0,33074843	Unpaired t-te	0,452022959	permutation FDR (250 perm	1
2417	Pcdh7~A0A0	Pcdh7	ko vs wt	ko	wt	27,2011113	0,58072376	27,6815985	0,491175	0,7167355	-0,4804873	-0,3240532	0,36775505	0,43444136	Unpaired t-te	0,45286879	permutation FDR (250 perm	1
2418	Pcdhb11~Q9	Pcdhb11	ko vs wt	ko	wt	24,7220307	0,49808671	24,4977864	0,88807935	1,16816517	0,22424427	0,13263553	0,76783737	0,11473075	Unpaired t-te	0,771734156	permutation FDR (250 perm	1
2419	Pcid2~Q8BF	Pcid2	ko vs wt	ko	wt	24,2459545	0,59492781	23,9551627	0,2816143	1,22331147	0,29079177	0,20814995	0,49965087	0,30133335	Unpaired t-te	0,630071723	permutation FDR (250 perm	1
2420	Pcif1~P5911	Pcif1	ko vs wt	ko	wt	24,2072983	0,36141286	25,0831036	0,47767113	0,54494961	-0,8758052	-0,6269098	0,10215292	0,99074921	Unpaired t-te	0,181922322	permutation FDR (250 perm	1
2421	Pck1~Q9Z2V	Pck1	ko vs wt	ko	wt	24,2756452	0,70544909	24,1355079	0,20405515	1,10200999	0,1401373	0,09785481	0,76313618	0,11739796	Unpaired t-te	0,831143093	permutation FDR (250 perm	1
2422	Pck2~Q8BH0	Pck2	ko vs wt	ko	wt	25,7717454	1,85852044	24,8301457	0,03962285	1,92065672	0,94159969	0,45413688	0,44486251	0,35177419	Unpaired t-te	0,319904597	permutation FDR (250 perm	1
2423	Pcm1~Q9R0	Pcm1	ko vs wt	ko	wt	24,7541162	0,88623436	25,4650176	0,10974915	0,61093828	-0,7109015	-0,4684629	0,25948324	0,58589068	Unpaired t-te	0,304532213	permutation FDR (250 perm	1
2424	Pcmt1~E0CY	Pcmt1	ko vs wt	ko	wt	28,2201772	0,77885222	28,0129859	0,47917779	1,15443854	0,20719137	0,13255702	0,7280321	0,13784947	Unpaired t-te	0,763093363	permutation FDR (250 perm	1
2425	Pcnt~F8VPV	Pcnt	ko vs wt	ko	wt	26,8615955	0,19278816	27,018199	0,26039818	0,89713466	-0,1566035	-0,1288751	0,51332216	0,28960999	Unpaired t-te	0,775405996	permutation FDR (250 perm	1
2426	Pcolce2~Q8R	Pcolce2	ko vs wt	ko	wt	28,4736684	1,04770103	27,9478521	0,57098256	1,43974799	0,5258163	0,3044227	0,50314912	0,29830328	Unpaired t-te	0,480549686	permutation FDR (250 perm	1
2427	Pcolce~Q613	Pcolce	ko vs wt	ko	wt	27,4617133	0,29174498	27,5274832	0,60411936	0,95543528	-0,0657699	-0,045073	0,89636855	0,04751339	Unpaired t-te	0,92579984	permutation FDR (250 perm	1
2428	Pcsk5~Q045	Pcsk5	ko vs wt	ko	wt	28,7171942	0,27187042	28,5394783	0,72367951	1,13109168	0,17771587	0,11575687	0,76688618	0,11526909	Unpaired t-te	0,805808783	permutation FDR (250 perm	1
2429	Pcx~G5E8R3	Pcx	ko vs wt	ko	wt	30,947938	0,32878781	31,2704695	0,67993635	0,79966547	-0,3225315	-0,2126247	0,58254119	0,23467336	Unpaired t-te	0,63667741	permutation FDR (250 perm	1

2430	Pcyox1~Q9C(	Pcyox1	ko vs wt	ko	wt	29,3834849	0,45922682	28,8499247	0,75053314	1,44749678	0,53356014	0,33488774	0,43476371	0,36174672	Unpaired t-t(	0,452941401	permutation FDR (250 perm	1
2431	Pcyt1a~P495	Pcyt1a	ko vs wt	ko	wt	26,1078276	1,53204808	27,0825489	0,76807805	0,50883814	-0,9747213	-0,4782827	0,39350451	0,40505029	Unpaired t-t(	0,279872386	permutation FDR (250 perm	1
2432	Pdcd10~Q8V	Pdcd10	ko vs wt	ko	wt	24,324909	0,40524591	24,8340169	1,29902871	0,70265681	-0,5091079	-0,2613649	0,63940531	0,19422376	Unpaired t-t(	0,565726862	permutation FDR (250 perm	1
2433	Pdcd11~Q6N	Pdcd11	ko vs wt	ko	wt	26,2212307	0,78000582	25,9768186	0,08739356	1,18460995	0,24441211	0,16803202	0,62686663	0,20282485	Unpaired t-t(	0,707224034	permutation FDR (250 perm	1
2434	Pdcd5~P5681	Pdcd5	ko vs wt	ko	wt	24,513756	0,4707115	24,7794809	0,14682019	0,83178069	-0,2657249	-0,2058415	0,41500511	0,38194655	Unpaired t-t(	0,637718805	permutation FDR (250 perm	1
2435	Pdcd6ip~Q9V	Pdcd6ip	ko vs wt	ko	wt	32,5395295	0,05904606	32,532722	0,24172665	1,0047298	0,00680757	0,00579716	0,97215713	0,01226353	Unpaired t-t(	0,990028638	permutation FDR (250 perm	1
2436	Pdcd6~P1281	Pdcd6	ko vs wt	ko	wt	28,8443385	0,15074007	28,6655928	0,31370069	1,13189938	0,17874572	0,14434978	0,51473544	0,28841593	Unpaired t-t(	0,751708333	permutation FDR (250 perm	1
2437	Pdcl~Q9DBX.	Pdcl	ko vs wt	ko	wt	24,4128565	0,82409266	24,460947	0,2276952	0,96721567	-0,0480905	-0,0320114	0,92874236	0,03210475	Unpaired t-t(	0,945324861	permutation FDR (250 perm	1
2438	Pde3a~Q9Z0	Pde3a	ko vs wt	ko	wt	27,926321	0,18684619	28,4571987	0,72454358	0,69213356	-0,5308776	-0,3484447	0,40974718	0,38748403	Unpaired t-t(	0,450958187	permutation FDR (250 perm	1
2439	Pde4dip~E9C	Pde4dip	ko vs wt	ko	wt	26,1905773	0,33918413	26,2333904	0,0519359	0,97076018	-0,0428132	-0,0357002	0,84280106	0,07427493	Unpaired t-t(	0,939477984	permutation FDR (250 perm	1
2440	Pde5a~Q8CG	Pde5a	ko vs wt	ko	wt	27,611566	1,27481923	25,2707674	1,2163516	5,06583005	2,34079868	1,09792354	0,10084214	0,99635794	Unpaired t-t(	0,033011407	permutation FDR (250 perm	1
2441	Pdgfa~Q99L!	Pdgfa	ko vs wt	ko	wt	25,1147968	0,64455435	24,8125947	0,79392784	1,23302504	0,3022021	0,18057752	0,67885549	0,16822267	Unpaired t-t(	0,681812518	permutation FDR (250 perm	1
2442	Pdgfb~P3124	Pdgfb	ko vs wt	ko	wt	23,8589507	0,94058635	25,121522	0,2893669	0,41680043	-1,2625713	-0,7989356	0,09884234	1,00505698	Unpaired t-t(	0,103310978	permutation FDR (250 perm	1
2443	Pdgfc~Q8CI1	Pdgfc	ko vs wt	ko	wt	29,0217135	0,68852565	28,7062117	0,52382539	1,24444437	0,31550174	0,20442781	0,5870704	0,23130982	Unpaired t-t(	0,635870816	permutation FDR (250 perm	1
2444	Pdgfd~Q925I	Pdgfd	ko vs wt	ko	wt	27,9265493	0,2179791	28,144847	0,22121094	0,85957911	-0,2182977	-0,1817993	0,33476535	0,47525951	Unpaired t-t(	0,677998263	permutation FDR (250 perm	1
2445	Pdgfra~P266	Pdgfra	ko vs wt	ko	wt	24,3187367	0,9028627	24,5624333	0,63300443	0,84457853	-0,2436965	-0,1444494	0,737327	0,13233986	Unpaired t-t(	0,740540571	permutation FDR (250 perm	1
2446	Pdgfrb~P056	Pdgfrb	ko vs wt	ko	wt	30,8406263	0,18681024	30,6811824	0,3845219	1,11685661	0,15944385	0,12336	0,62838796	0,20177214	Unpaired t-t(	0,790339475	permutation FDR (250 perm	1
2447	Pdgfrl~Q6PE	Pdgfrl	ko vs wt	ko	wt	25,5170855	0,82393308	24,7476659	0,29980191	1,70458385	0,76941957	0,50593192	0,21245714	0,67272866	Unpaired t-t(	0,261246753	permutation FDR (250 perm	1
2448	Pdha1~P354!	Pdha1	ko vs wt	ko	wt	29,7094436	0,45054521	29,8268437	0,65354853	0,92184745	-0,1174001	-0,0767166	0,8379835	0,07676453	Unpaired t-t(	0,87165521	permutation FDR (250 perm	1
2449	Pdhb~Q9D05	Pdhb	ko vs wt	ko	wt	30,6988856	0,21875742	30,3809593	0,22013934	1,24653753	0,31792632	0,26483815	0,18347576	0,7364213	Unpaired t-t(	0,541005491	permutation FDR (250 perm	1
2450	Pdhx~Q8BKZ	Pdhx	ko vs wt	ko	wt	27,3593309	0,53097857	27,3148118	0,19713706	1,03133936	0,04451913	0,03330362	0,90105577	0,04524833	Unpaired t-t(	0,942678325	permutation FDR (250 perm	1
2451	Pdia3~P2777	Pdia3	ko vs wt	ko	wt	32,9996996	0,39846746	32,5468698	0,25846097	1,36872232	0,45282979	0,34999614	0,18394007	0,73532365	Unpaired t-t(	0,419546727	permutation FDR (250 perm	1
2452	Pdia4~P0800	Pdia4	ko vs wt	ko	wt	31,7141525	0,51257175	31,6939751	0,45976209	1,01408421	0,02017747	0,01401581	0,96532203	0,01532778	Unpaired t-t(	0,975412719	permutation FDR (250 perm	1
2453	Pdia5~Q921)	Pdia5	ko vs wt	ko	wt	29,6585891	1,48852914	28,8322802	0,37204954	1,77314302	0,8263089	0,43518167	0,41613633	0,38076436	Unpaired t-t(	0,332407827	permutation FDR (250 perm	1
2454	Pdia6~Q3TM	Pdia6	ko vs wt	ko	wt	30,5970682	0,31861284	30,488338	0,29772163	1,07827875	0,10873019	0,08497427	0,71512318	0,14561915	Unpaired t-t(	0,852732055	permutation FDR (250 perm	1
2455	Pdk1~Q8BFP	Pdk1	ko vs wt	ko	wt	25,4699936	1,42190774	25,7212267	0,59240883	0,84017795	-0,2512332	-0,1307391	0,79768865	0,09816659	Unpaired t-t(	0,768126859	permutation FDR (250 perm	1
2456	Pdk2~Q9JK42	Pdk2	ko vs wt	ko	wt	25,1106161	1,00225286	26,7247802	0,02628498	0,32665415	-1,6141641	-1,0223026	0,06836669	1,16515542	Unpaired t-t(	0,053161003	permutation FDR (250 perm	1
2457	Pdk3~Q922H	Pdk3	ko vs wt	ko	wt	27,6965234	0,39081118	27,6214205	0,02344585	1,05343619	0,07510292	0,06124635	0,76154515	0,11830434	Unpaired t-t(	0,895991377	permutation FDR (250 perm	1
2458	Pdk4~O7057	Pdk4	ko vs wt	ko	wt	28,8212906	0,57700182	28,1142512	0,19009396	1,63245069	0,70703942	0,52017713	0,12220545	0,91290942	Unpaired t-t(	0,250925656	permutation FDR (250 perm	1
2459	Pdlim1~O70	Pdlim1	ko vs wt	ko	wt	31,8173134	0,35731162	32,0631235	0,10537822	0,84334208	-0,2458102	-0,201593	0,32920484	0,48253379	Unpaired t-t(	0,645996491	permutation FDR (250 perm	1
2460	Pdlim3~O70.	Pdlim3	ko vs wt	ko	wt	31,070378	0,40531936	31,6386706	0,1091622	0,67441445	-0,5682927	-0,4559424	0,08922462	1,04951528	Unpaired t-t(	0,311106309	permutation FDR (250 perm	1
2461	Pdlim4~P702	Pdlim4	ko vs wt	ko	wt	29,5171641	0,49958112	29,9054372	0,40116941	0,76404363	-0,3882731	-0,2764391	0,38271105	0,417129	Unpaired t-t(	0,520356813	permutation FDR (250 perm	1
2462	Pdlim5~Q8CI	Pdlim5	ko vs wt	ko	wt	31,7948061	0,19208269	31,9458093	0,33615597	0,90062398	-0,1510032	-0,1196259	0,60678215	0,2169672	Unpaired t-t(	0,795795759	permutation FDR (250 perm	1
2463	Pdlim5~Q8CI	Pdlim5	ko vs wt	ko	wt	24,40167	1,09359721	23,8555884	0,6570217	1,46011461	0,54608162	0,30611769	0,51737944	0,28619084	Unpaired t-t(	0,477386213	permutation FDR (250 perm	1
2464	Pdlim7~Q3TJ	Pdlim7	ko vs wt	ko	wt	33,9816257	0,38660964	34,2345365	0,22095512	0,83920154	-0,2529108	-0,1987578	0,39672647	0,40150882	Unpaired t-t(	0,645180117	permutation FDR (250 perm	1
2465	Pdlim7~Q3TJ	Pdlim7	ko vs wt	ko	wt	25,2819548	1,72085114	28,4766394	0,87806559	0,10922048	-3,1946846	-1,471131	0,04403246	1,35622706	Unpaired t-t(	0,008876819	permutation FDR (250 perm	1
2466	Pdp1~Q3UV7	Pdp1	ko vs wt	ko	wt	25,0724358	1,52075227	27,0090748	0,24094624	0,2612243	-1,936639	-1,0223052	0,11273001	0,94796044	Unpaired t-t(	0,050845592	permutation FDR (250 perm	1
2467	Pdpk1~Q9Z2.	Pdpk1	ko vs wt	ko	wt	26,9957802	0,43274811	26,6452923	0,20458264	1,27499175	0,35048792	0,27196905	0,28364635	0,54722279	Unpaired t-t(	0,528029439	permutation FDR (250 perm	1
2468	Pds5a~Q6A0	Pds5a	ko vs wt	ko	wt	27,3529515	0,35672656	27,3064522	0,68217639	1,03275591	0,04649931	0,03050135	0,9354425	0,0289829	Unpaired t-t(	0,948693446	permutation FDR (250 perm	1
2469	Pds5b~Q4VA	Pds5b	ko vs wt	ko	wt	29,8422086	0,18593113	29,7866569	0,14176142	1,03925668	0,05555174	0,04843758	0,72105482	0,14203172	Unpaired t-t(	0,915271463	permutation FDR (250 perm	1
2470	Pdxdc1~Q99I	Pdxdc1	ko vs wt	ko	wt	28,0220201	0,28697594	27,8989963	0,26072498	1,08901499	0,12302382	0,09858689	0,64268044	0,19200492	Unpaired t-t(	0,826788414	permutation FDR (250 perm	1
2471	Pdxk~Q8K18.	Pdxk	ko vs wt	ko	wt	26,4985066	0,27560756	25,4379679	0,65046574	2,08571015	1,06053868	0,7133529	0,13593474	0,86666953	Unpaired t-t(	0,152631839	permutation FDR (250 perm	1
2472	Peak1~Q69Z.	Peak1	ko vs wt	ko	wt	28,3611804	0,28294004	28,479447	0,51986778	0,92129391	-0,1182666	-0,0843398	0,78922058	0,1028016	Unpaired t-t(	0,859135667	permutation FDR (250 perm	1
2473	Pebp1~P702!	Pebp1	ko vs wt	ko	wt	27,648617	0,72318283	26,6497323	0,868194	1,99845446	0,9988847	0,57326902	0,25418983	0,59484183	Unpaired t-t(	0,213142209	permutation FDR (250 perm	1
2474	Pecam1~Q0!	Pecam1	ko vs wt	ko	wt	26,0412197	1,7267962	27,6030842	0,23714134	0,33871307	-1,5618644	-0,7766723	0,21531884	0,66691797	Unpaired t-t(	0,117944643	permutation FDR (250 perm	1
2475	Pef1~Q8BFY	Pef1	ko vs wt	ko	wt	28,6811852	0,2629378	27,8695722	0,74787438	1,75517272	0,811613	0,52355882	0,26091743	0,5834969	Unpaired t-t(	0,270121864	permutation FDR (250 perm	1
2476	Pelo~Q80X7.	Pelo	ko vs wt	ko	wt	26,7385842	0,22072924	27,0263286	0,10264324	0,81918184	-0,2877444	-0,250942	0,1127481	0,94789078	Unpaired t-t(	0,559972189	permutation FDR (250 perm	1
2477	Pes1~Q9EQ6	Pes1	ko vs wt	ko	wt	24,9768675	1,05042795	23,8385818	0,77854885	2,2011931	1,13828571	0,62575291	0,22392894	0,64988978	Unpaired t-t(	0,172803507	permutation FDR (250 perm	1
2478	Pf4~Q9Z126	Pf4	ko vs wt	ko	wt	28,3386489	1,0489219	25,9185317	0,68893847	5,35214501	2,42011721	1,36174668	0,0264435	1,5776811	Unpaired t-t(	0,011690739	permutation FDR (250 perm	1
2479	Pfdn2~O705!	Pfdn2	ko vs wt	ko	wt	24,4932143	1,13491803	24,6565302	0,41486537	0,89297028	-0,1633159	-0,0950663	0,8309985	0,08039976	Unpaired t-t(	0,835019824	permutation FDR (250 perm	1
2480	Pfdn6~Q039!	Pfdn6	ko vs wt	ko	wt	25,0826527	1,21816047	24,6881294	0,27945724	1,31450831	0,39452326	0,22797717	0,62201898	0,20619636	Unpaired t-t(	0,602320285	permutation FDR (250 perm	1
2481	Pfkl~P12382	Pfkl	ko vs wt	ko	wt	28,9035643	0,12045441	28,4762095	0,17021922	1,34476567	0,4273548	0,37519854	0,04771418	1,32135258	Unpaired t-t(	0,400654617	permutation FDR (250 perm	1
2482	Pfkm~P4785	Pfkm	ko vs wt	ko	wt	29,7304637	0,15155292	29,6803521	0,27633984	1,03534503	0,05011163	0,04127478	0,83095877	0,08042052	Unpaired t-t(	0,930673763	permutation FDR (250 perm	1
2483	Pfkp~Q9WU.	Pfkp	ko vs wt	ko	wt	30,9890618	0,16212676	31,0461549	0,42511674	0,9611989	-0,0570931	-0,0434221	0,87033748	0,06031232	Unpaired t-t(	0,928139547	permutation FDR (250 perm	1
2484	Pfn1~P62962	Pfn1	ko vs wt	ko	wt	32,1188135	0,73563527	31,7193678	0,1311185	1,31900109	0,39944576	0,27841352	0,42061055	0,37611984	Unpaired t-t(	0,527654094	permutation FDR (250 perm	1
2485	Pfn2~Q9JJV2	Pfn2	ko vs wt	ko	wt	25,1816137	1,42017975	26,9974246	0,06775	0,28404455	-1,8158109	-0,9969645	0,11341203	0,94534087	Unpaired t-t(	0,057436788	permutation FDR (250 perm	1
2486	Pgam1~Q9DI	Pgam1	ko vs wt	ko	wt	28,5810573	0,74870455	28,2210897	0,24892117	1,28339707	0,3599676	0,24542242	0,48445072	0,31475039	Unpaired t-t(	0,57222637	permutation FDR (250 perm	1
2487	Pgam2~O70.	Pgam2	ko vs wt	ko	wt	24,4383449	1,24548723	24,9818042	2,02503448	0,68612371	-0,5434594	-0,2088357	0,75677916	0,12103084	Unpaired t-t(	0,638993851	permutation FDR (250 perm	1
2488	Pgd~Q9DCD(	Pgd	ko vs wt	ko	wt	29,7120342	0,51436615	29,1751227	0,28742509	1,45086322	0,53691151	0,39482924	0,19746236	0,70451567	Unpaired t-t(	0,366854069	permutation FDR (250 perm	1
2489	Pgk1~P0941	Pgk1	ko vs wt	ko	wt	32,2399135	0,18350274	31,9572492	0,08985806	1,21643928	0,28266431	0,2515841	0,0748478	1,12582098	Unpaired t-t(	0,558498488	permutation FDR (250 perm	1
2490	Pgls~Q9CQ6	Pgls	ko vs wt	ko	wt	27,4396659	1,32300803	28,4665708	0,36394286	0,49076187	-1,0269049	-0,5685993	0,27773903	0,55636309	Unpaired t-t(	0,216763842	permutation FDR (250 perm	1
2491	Pglyrp2~Q8V	Pglyrp2	ko vs wt	ko	wt	25,1898878	1,01593331	24,1027046	0,25094853	2,12458817	1,08718322	0,67409541	0,16018805	0,7953699	Unpaired t-t(	0,156856793	permutation FDR (250 perm	1
2492	Pgm1~Q9D0	Pgm1	ko vs wt	ko	wt	28,994655	1,33760665	29,3916985	0,66492269	0,75941293	-0,3970435	-0,2085166	0,68011499	0,16741765	Unpaired t-t(	0,629254118	permutation FDR (250 perm	1
2493	Pgm3~Q9CYI	Pgm3	ko vs wt	ko	wt	27,35411	0,46028257	26,7713116	0,02318887	1,49775162	0,58279839	0,46025552	0,11569558	0,93668322	Unpaired t-t(	0,314329043	permutation FDR (250 perm	1

2494	Pgm5~Q8BZI	Pgm5	ko vs wt	ko	wt	32,7884241	0,59256387	33,3463003	0,34815024	0,67930143	-0,5578762	-0,3924606	0,24370085	0,61314296	Unpaired t-te	0,36971563	permutation FDR (250 perm	1
2495	Pgrmc1~O55	Pgrmc1	ko vs wt	ko	wt	27,6858963	0,78399181	27,8048274	0,34108884	0,92086963	-0,1189312	-0,0786121	0,8269909	0,08249927	Unpaired t-te	0,864168122	permutation FDR (250 perm	1
2496	Phaf1~Q922I	Phaf1	ko vs wt	ko	wt	24,6139278	0,7335486	26,5415523	0,25686271	0,26286164	-1,9276245	-1,3195509	0,01386997	1,85792446	Unpaired t-te	0,015903727	permutation FDR (250 perm	1
2497	Phb1~P6777	Phb1	ko vs wt	ko	wt	30,0169128	0,36911098	30,0235365	0,04620637	0,99541931	-0,0066237	-0,0054489	0,9773399	0,00995437	Unpaired t-te	0,990333767	permutation FDR (250 perm	1
2498	Phb2~O3512	Phb2	ko vs wt	ko	wt	30,9962449	0,2858475	30,7641234	0,42626093	1,17456084	0,23212144	0,17275624	0,54513737	0,26349404	Unpaired t-te	0,698750072	permutation FDR (250 perm	1
2499	Pheta2~Q14I	Pheta2	ko vs wt	ko	wt	25,2897837	0,48239545	24,58668	0,77286026	1,62800332	0,70310364	0,43579765	0,33389783	0,4763864	Unpaired t-te	0,338265572	permutation FDR (250 perm	1
2500	Phex~P7066	Phex	ko vs wt	ko	wt	24,689616	0,8019243	26,226563	0,64947042	0,34461394	-1,536947	-0,9302851	0,0669759	1,17408144	Unpaired t-te	0,059423056	permutation FDR (250 perm	1
2501	Phf11~A6H5	Phf11	ko vs wt	ko	wt	22,2036361	0,8381861	23,3691537	1,44928808	0,44580429	-1,1655176	-0,546341	0,38187955	0,4180736	Unpaired t-te	0,242765723	permutation FDR (250 perm	1
2502	Phf5a~P8387	Phf5a	ko vs wt	ko	wt	26,2176078	2,28456898	24,9821037	1,32551866	2,35463608	1,2355041	0,47190845	0,48016043	0,31861364	Unpaired t-te	0,284454963	permutation FDR (250 perm	1
2503	Phf6~Q9D4J	Phf6	ko vs wt	ko	wt	23,9659184	0,34264141	24,3154492	0,48038836	0,78483929	-0,3495308	-0,250903	0,43342642	0,36308462	Unpaired t-te	0,568080796	permutation FDR (250 perm	1
2504	Phgdh~Q617	Phgdh	ko vs wt	ko	wt	24,8305952	1,71781035	25,3033438	1,33446308	0,72059044	-0,4727486	-0,1995605	0,74421253	0,12830302	Unpaired t-te	0,643563615	permutation FDR (250 perm	1
2505	Phldb1~Q6PI	Phldb1	ko vs wt	ko	wt	27,7575976	0,33961239	27,6822768	0,31216694	1,05359531	0,07532083	0,0581518	0,8098223	0,09161027	Unpaired t-te	0,898978998	permutation FDR (250 perm	1
2506	Phldb2~Q8K1	Phldb2	ko vs wt	ko	wt	27,4456655	0,29551517	27,5439428	0,37884928	0,93414777	-0,0982773	-0,0745878	0,77423845	0,11112526	Unpaired t-te	0,874414601	permutation FDR (250 perm	1
2507	Phyhd1~Q9D	Phyhd1	ko vs wt	ko	wt	25,1104858	1,43899283	25,1890028	1,59464374	0,94703066	-0,078517	-0,0327072	0,95799626	0,01863619	Unpaired t-te	0,943549788	permutation FDR (250 perm	1
2508	Pi15~Q8BS0	Pi15	ko vs wt	ko	wt	32,9231432	0,26582011	33,2335918	0,27892222	0,80639097	-0,3104486	-0,2483779	0,27982213	0,55311795	Unpaired t-te	0,565536328	permutation FDR (250 perm	1
2509	Pi4k2a~Q2TE	Pi4k2a	ko vs wt	ko	wt	24,9934185	0,60847374	24,8588654	1,20638837	1,09775269	0,13455307	0,06998675	0,89429065	0,04852131	Unpaired t-te	0,884719284	permutation FDR (250 perm	1
2510	Pi4ka~E9Q3L	Pi4ka	ko vs wt	ko	wt	28,2001105	0,438267	28,0591541	0,46171752	1,10263587	0,14095644	0,09975271	0,74957042	0,12518756	Unpaired t-te	0,825459161	permutation FDR (250 perm	1
2511	Picalm~Q7M	Picalm	ko vs wt	ko	wt	30,4822698	0,44151639	30,0182377	0,14632715	1,37939164	0,46403213	0,36391578	0,1685616	0,77324136	Unpaired t-te	0,409126188	permutation FDR (250 perm	1
2512	Pik3c2a~Q61	Pik3c2a	ko vs wt	ko	wt	26,574521	0,09997087	26,5110088	0,04350603	1,04500668	0,06351217	0,05961318	0,38168703	0,41829259	Unpaired t-te	0,89629426	permutation FDR (250 perm	1
2513	Pik3c3~Q6PF	Pik3c3	ko vs wt	ko	wt	25,3505272	1,44499762	26,0489842	0,20073	0,61623093	-0,698457	-0,3783096	0,4666721	0,33098817	Unpaired t-te	0,398970036	permutation FDR (250 perm	1
2514	Pik3ca~P423	Pik3ca	ko vs wt	ko	wt	25,1510593	1,21498758	24,492312	0,49294862	1,57871117	0,65874725	0,3693972	0,44484439	0,35179189	Unpaired t-te	0,399468071	permutation FDR (250 perm	1
2515	Pik3r4~Q8VD	Pik3r4	ko vs wt	ko	wt	27,5960947	0,91257871	27,3393976	0,49104527	1,19474032	0,25669708	0,15738619	0,70161274	0,15390254	Unpaired t-te	0,718411178	permutation FDR (250 perm	1
2516	Pin1~Q9QUR	Pin1	ko vs wt	ko	wt	27,2233485	0,45519171	26,7615062	0,61765956	1,37729956	0,46184238	0,30591187	0,42427617	0,37235136	Unpaired t-te	0,488125196	permutation FDR (250 perm	1
2517	Pip4k2a~O70	Pip4k2a	ko vs wt	ko	wt	29,0538127	0,57827913	29,0149958	0,14193827	1,02727105	0,0388169	0,02878249	0,91739338	0,0374444	Unpaired t-te	0,9500417	permutation FDR (250 perm	1
2518	Pip4k2c~Q91	Pip4k2c	ko vs wt	ko	wt	26,9910444	0,68236295	24,0181781	0,26831588	7,85094454	2,97286623	2,06841501	0,00202167	2,69429013	Unpaired t-te	0,00311284	permutation FDR (250 perm	1
2519	Pip5k1a~P70	Pip5k1a	ko vs wt	ko	wt	24,9476257	0,78341328	23,9209806	0,44537524	2,03728114	1,02664508	0,6618637	0,12284789	0,9106323	Unpaired t-te	0,153667351	permutation FDR (250 perm	1
2520	Pip5k1c~O70	Pip5k1c	ko vs wt	ko	wt	26,4427298	0,02114334	26,4944894	0,10432837	0,96475896	-0,0517596	-0,0481585	0,55709825	0,25406821	Unpaired t-te	0,921982897	permutation FDR (250 perm	1
2521	Pitpna~P538	Pitpna	ko vs wt	ko	wt	28,0618826	0,1830488	28,1198704	1,11447818	0,96060297	-0,0579878	-0,0323032	0,94829313	0,0230574	Unpaired t-te	0,946855632	permutation FDR (250 perm	1
2522	Pitpnb~Q8JZ	Pitpnb	ko vs wt	ko	wt	25,967658	1,65542663	25,7987931	1,21957632	1,12417358	0,16886481	0,07382688	0,90079904	0,04537209	Unpaired t-te	0,873558767	permutation FDR (250 perm	1
2523	Pitpnm1~O3	Pitpnm1	ko vs wt	ko	wt	26,2019844	0,12928261	25,9672471	0,14393431	1,17669244	0,23473729	0,20843052	0,13705198	0,86311469	Unpaired t-te	0,63332824	permutation FDR (250 perm	1
2524	Pitpnm2~Q6	Pitpnm2	ko vs wt	ko	wt	25,9065203	0,31178605	25,360921	0,70342512	1,45962659	0,54559934	0,35684161	0,39071721	0,40813746	Unpaired t-te	0,434175371	permutation FDR (250 perm	1
2525	Pitrm1~Q8K	Pitrm1	ko vs wt	ko	wt	28,3308921	0,69110119	27,5380334	0,39932915	1,73250402	0,7928587	0,53254393	0,16679627	0,77781368	Unpaired t-te	0,234291219	permutation FDR (250 perm	1
2526	Pkd1~O0885	Pkd1	ko vs wt	ko	wt	24,1919516	0,39005106	24,3466744	0,53138878	0,89830496	-0,1547228	-0,107591	0,74471326	0,12801091	Unpaired t-te	0,813481728	permutation FDR (250 perm	1
2527	Pkd2~O3524	Pkd2	ko vs wt	ko	wt	24,8717306	0,42645373	24,4021473	0,98230411	1,38470949	0,46958333	0,27035081	0,57732611	0,2385788	Unpaired t-te	0,549139041	permutation FDR (250 perm	1
2528	Pkm~P52480	Pkm	ko vs wt	ko	wt	30,4389611	0,3760256	29,5677503	0,2576154	1,8291975	0,87121085	0,67883123	0,02767891	1,55785109	Unpaired t-te	0,145660125	permutation FDR (250 perm	1
2529	Pkm~P52480	Pkm	ko vs wt	ko	wt	33,6935082	0,30755376	33,0586327	0,16106907	1,55280375	0,63487551	0,52427735	0,03163207	1,49987242	Unpaired t-te	0,242206186	permutation FDR (250 perm	1
2530	Pkn1~P7026	Pkn1	ko vs wt	ko	wt	27,8670684	0,22964503	27,6213983	0,16878372	1,18564337	0,24567013	0,20847951	0,22744456	0,64312445	Unpaired t-te	0,628245955	permutation FDR (250 perm	1
2531	Pkn2~Q8BW	Pkn2	ko vs wt	ko	wt	27,4102488	0,37275341	27,0724793	0,29770832	1,26380116	0,33776949	0,25961331	0,31442405	0,50248424	Unpaired t-te	0,545919708	permutation FDR (250 perm	1
2532	Pkp4~A2AS4	Pkp4	ko vs wt	ko	wt	25,9424061	0,6254482	25,9952841	0,51035957	0,96401137	-0,0528779	-0,0350065	0,92169155	0,0354144	Unpaired t-te	0,939068435	permutation FDR (250 perm	1
2533	Pla2g4a~P47	Pla2g4a	ko vs wt	ko	wt	28,693799	0,6495415	27,3973274	0,20517171	2,45627419	1,29647161	0,92466578	0,03413435	1,46680836	Unpaired t-te	0,066114108	permutation FDR (250 perm	1
2534	Pla2g7~Q60	Pla2g7	ko vs wt	ko	wt	25,6880348	1,15281133	24,593173	1,45897535	2,13592622	1,09486182	0,49147196	0,42814734	0,36840675	Unpaired t-te	0,277105263	permutation FDR (250 perm	1
2535	Plaa~P27612	Plaa	ko vs wt	ko	wt	27,038924	0,11253547	23,701996	0,60429334	10,1045134	3,33692794	2,32991311	0,01436757	1,84261676	Unpaired t-te	0,004262482	permutation FDR (250 perm	1
2536	Plac9~Q8K2	Plac9	ko vs wt	ko	wt	28,4400096	0,4434111	28,5166718	0,13332645	0,94824895	-0,0766622	-0,0602306	0,79346411	0,10047271	Unpaired t-te	0,896165993	permutation FDR (250 perm	1
2537	Plat~P11214	Plat	ko vs wt	ko	wt	27,3346153	0,08312801	27,4539581	0,12215725	0,92060696	-0,1193427	-0,1086103	0,30829946	0,51102724	Unpaired t-te	0,812270285	permutation FDR (250 perm	1
2538	Plcb1~Q9Z1E	Plcb1	ko vs wt	ko	wt	28,1764766	0,58737326	28,118278	0,35330886	1,04116494	0,05819863	0,04095012	0,89554784	0,04791121	Unpaired t-te	0,928351438	permutation FDR (250 perm	1
2539	Plcb3~P5143	Plcb3	ko vs wt	ko	wt	29,720614	0,17243408	30,0665024	0,2084107	0,78682334	-0,3458883	-0,2936621	0,12890961	0,88971469	Unpaired t-te	0,502856077	permutation FDR (250 perm	1
2540	Plcb4~Q91U	Plcb4	ko vs wt	ko	wt	31,4356444	0,56968924	32,0003318	0,0610483	0,67610191	-0,5646874	-0,4240251	0,18422705	0,7346466	Unpaired t-te	0,350463946	permutation FDR (250 perm	1
2541	Plcd1~Q8R3E	Plcd1	ko vs wt	ko	wt	29,2477825	1,16224447	29,683698	0,7321653	0,73922448	-0,4359156	-0,2359453	0,62899761	0,201351	Unpaired t-te	0,581180485	permutation FDR (250 perm	1
2542	Plcg1~Q6207	Plcg1	ko vs wt	ko	wt	25,2898553	1,27518803	25,0435116	1,23841207	1,18619702	0,24634366	0,11489602	0,83919389	0,07613769	Unpaired t-te	0,798629847	permutation FDR (250 perm	1
2543	Plcg2~Q8CIH	Plcg2	ko vs wt	ko	wt	26,0687921	2,16464261	24,6746841	0,46539654	2,62826	1,394108	0,60815454	0,35110863	0,4545585	Unpaired t-te	0,19456446	permutation FDR (250 perm	1
2544	Pld3~O3540	Pld3	ko vs wt	ko	wt	25,2866288	1,73265545	25,1829719	1,70071997	1,07449367	0,10365698	0,04042367	0,95010811	0,02222698	Unpaired t-te	0,930086771	permutation FDR (250 perm	1
2545	Plec~Q9QXS	Plec	ko vs wt	ko	wt	35,5451083	0,28925438	35,584252	0,29760511	0,9732324	-0,0391437	-0,0308546	0,89088004	0,05018077	Unpaired t-te	0,946824028	permutation FDR (250 perm	1
2546	Plekhg3~Q4	Plekhg3	ko vs wt	ko	wt	27,8601261	0,47345771	27,5714626	0,17637562	1,22150823	0,28866359	0,22197074	0,38970702	0,40926177	Unpaired t-te	0,608002367	permutation FDR (250 perm	1
2547	Plekhh3~Q8	Plekhh3	ko vs wt	ko	wt	24,7566847	0,62366503	23,8451066	0,23981148	1,88110205	0,91157812	0,65205671	0,0811135	1,09090686	Unpaired t-te	0,163167274	permutation FDR (250 perm	1
2548	Plekho2~Q8	Plekho2	ko vs wt	ko	wt	25,0934668	1,24267783	24,6939002	1,15681646	1,31911152	0,39956653	0,19135833	0,73011292	0,13660996	Unpaired t-te	0,660052402	permutation FDR (250 perm	1
2549	Plek~Q9JHK5	Plek	ko vs wt	ko	wt	29,912421	2,0433155	27,4978038	0,81428815	5,33178	2,41461725	1,0440575	0,13565376	0,86756817	Unpaired t-te	0,040558195	permutation FDR (250 perm	1
2550	Plg~P20918	Plg	ko vs wt	ko	wt	33,0662358	0,94166194	31,9748189	0,31335303	2,13083207	1,0914169	0,68768574	0,13806128	0,8599281	Unpaired t-te	0,147537822	permutation FDR (250 perm	1
2551	Plin1~Q8CG	Plin1	ko vs wt	ko	wt	30,983208	0,34943179	31,3862672	0,21153731	0,75625293	-0,4030593	-0,3221518	0,16992525	0,76974208	Unpaired t-te	0,454577523	permutation FDR (250 perm	1
2552	Plin2~P4388	Plin2	ko vs wt	ko	wt	27,6776097	0,8487056	27,0559859	0,10968766	1,53860594	0,62162378	0,4154959	0,2951649	0,52993529	Unpaired t-te	0,358914015	permutation FDR (250 perm	1
2553	Plin3~Q9DB	Plin3	ko vs wt	ko	wt	26,1862226	0,37371504	26,2053557	0,22198286	0,98682548	-0,0191331	-0,0151033	0,9456465	0,02427118	Unpaired t-te	0,972925654	permutation FDR (250 perm	1
2554	Plin4~O8849	Plin4	ko vs wt	ko	wt	31,6825865	0,65423572	32,7909463	0,41578341	0,46382104	-1,1083598	-0,7495719	0,0685432	1,16403561	Unpaired t-te	0,114718708	permutation FDR (250 perm	1
2555	Plod1~Q9R0I	Plod1	ko vs wt	ko	wt	28,7585049	1,31419089	27,6806804	0,54268971	2,11085063	1,07782449	0,58252352	0,2691158	0,57006081	Unpaired t-te	0,203060176	permutation FDR (250 perm	1
2556	Plod2~Q9R0I	Plod2	ko vs wt	ko	wt	26,2231501	1,25388439	23,7980249	0,80318335	5,37075615	2,42512522	1,26300486	0,0462861	1,33454942	Unpaired t-te	0,016773212	permutation FDR (250 perm	1
2557	Plod3~Q9R0I	Plod3	ko vs wt	ko	wt	28,6313902	1,00260493	27,9413807	0,13493691	1,61329411	0,69000947	0,43488029	0,32043907	0,49425453	Unpaired t-te	0,337271732	permutation FDR (250 perm	1

2558	Plpbp~Q9Z2\	Plpbp	ko vs wt	ko	wt	27,7424861	0,69494516	26,8538663	1,18973822	1,8514041	0,88861982	0,4599357	0,41262442	0,38444508	Unpaired t-te	0,316240361	permutation FDR (250 perm	1
2559	Plrg1~Q922V	Plrg1	ko vs wt	ko	wt	28,316319	0,4325137	27,9403049	0,3864732	1,29775146	0,37601411	0,27442584	0,36018727	0,44347164	Unpaired t-te	0,524085942	permutation FDR (250 perm	1
2560	Pls1~Q3V0K	Pls1	ko vs wt	ko	wt	25,5979476	1,81607977	24,9624136	0,54337171	1,5535127	0,63553403	0,30024824	0,60155243	0,22072652	Unpaired t-te	0,492658765	permutation FDR (250 perm	1
2561	Pls3~Q99K51	Pls3	ko vs wt	ko	wt	32,3957052	0,19653547	32,2736479	0,17756575	1,0882857	0,12205735	0,10439086	0,50596007	0,29588376	Unpaired t-te	0,81595793	permutation FDR (250 perm	1
2562	Pltp~A2A5K2	Pltp	ko vs wt	ko	wt	28,3116062	0,49375003	27,5543824	0,38111734	1,69023492	0,75722377	0,54387088	0,11269241	0,94810533	Unpaired t-te	0,226352765	permutation FDR (250 perm	1
2563	Plvap~G3X92	Plvap	ko vs wt	ko	wt	25,3305418	1,17483372	25,1707457	0,30030509	1,11712928	0,15979615	0,09340692	0,83440034	0,07862553	Unpaired t-te	0,840036314	permutation FDR (250 perm	1
2564	Plxdc2~Q9D(	Plxdc2	ko vs wt	ko	wt	27,7121629	0,57289413	27,4614851	0,53059483	1,18976598	0,25067783	0,16709984	0,63976021	0,19398277	Unpaired t-te	0,701830897	permutation FDR (250 perm	1
2565	Plxna1~P702	Plxna1	ko vs wt	ko	wt	24,5109072	0,30106204	24,7667179	0,33655138	0,83751635	-0,2558107	-0,1975835	0,43488762	0,36162295	Unpaired t-te	0,651146107	permutation FDR (250 perm	1
2566	Plxnb2~B2R)	Plxnb2	ko vs wt	ko	wt	27,3077864	0,35092414	26,8604227	0,22448291	1,36354624	0,44736362	0,35578972	0,14278269	0,84532443	Unpaired t-te	0,411956863	permutation FDR (250 perm	1
2567	Pmfbp1~Q9\	Pmfbp1	ko vs wt	ko	wt	24,2174328	0,82028183	25,6192096	1,83643772	0,37846273	-1,4017768	-0,5884324	0,39726954	0,40091473	Unpaired t-te	0,218875847	permutation FDR (250 perm	1
2568	Pmm2~Q9Z2	Pmm2	ko vs wt	ko	wt	24,4902125	0,4939354	24,4532425	0,9549441	1,02595683	0,03697002	0,02133301	0,96324628	0,01626266	Unpaired t-te	0,963828692	permutation FDR (250 perm	1
2569	Pmpca~Q9D(	Pmpca	ko vs wt	ko	wt	26,6939029	0,7146243	25,118685	1,23435284	2,9798048	1,57521783	0,80146479	0,2044556	0,68940098	Unpaired t-te	0,111447096	permutation FDR (250 perm	1
2570	Pmpcb~Q9C)	Pmpcb	ko vs wt	ko	wt	25,1482783	1,23532933	23,574739	0,31271555	2,97633984	1,57353926	0,90085918	0,11144239	0,95294959	Unpaired t-te	0,07401003	permutation FDR (250 perm	1
2571	Pnkp~Q9JLV(	Pnkp	ko vs wt	ko	wt	25,054606	1,52289904	24,5755472	1,323902	1,39383406	0,47905881	0,20971779	0,7254838	0,13937228	Unpaired t-te	0,627430253	permutation FDR (250 perm	1
2572	Pnn~O35691	Pnn	ko vs wt	ko	wt	26,0380345	1,6110134	24,0371834	0,43257527	4,0023604	2,00085108	1,01097858	0,11782108	0,92877701	Unpaired t-te	0,049847371	permutation FDR (250 perm	1
2573	Pnpla2~Q8BJ	Pnpla2	ko vs wt	ko	wt	25,2396742	1,62681533	28,0149553	0,16660497	0,14606868	-2,7752812	-1,4257058	0,05864276	1,23178557	Unpaired t-te	0,01378173	permutation FDR (250 perm	1
2574	Pnpt1~Q8K1I	Pnpt1	ko vs wt	ko	wt	24,3743216	1,43385207	24,1828144	0,29937613	1,1419561	0,19150719	0,10326773	0,8355267	0,07803967	Unpaired t-te	0,82168997	permutation FDR (250 perm	1
2575	Pnp~P23492	Pnp	ko vs wt	ko	wt	27,1032668	1,00672551	26,7110026	0,42618732	1,31245157	0,39226418	0,23705866	0,57884973	0,23743416	Unpaired t-te	0,582393295	permutation FDR (250 perm	1
2576	Podn~Q7TQ(	Podn	ko vs wt	ko	wt	28,7563374	0,32845234	28,7780635	0,08024063	0,98505347	-0,0217261	-0,0181362	0,91856687	0,03688922	Unpaired t-te	0,968950682	permutation FDR (250 perm	1
2577	Podxl~Q9R0I	Podxl	ko vs wt	ko	wt	27,0607242	0,86054418	27,1166442	0,47589109	0,96198077	-0,05592	-0,0349485	0,92947801	0,03176088	Unpaired t-te	0,939129973	permutation FDR (250 perm	1
2578	Pofut1~A2AI	Pofut1	ko vs wt	ko	wt	25,6343575	1,53536013	24,9061057	0,25064818	1,65663038	0,72825175	0,38248815	0,47544429	0,32290036	Unpaired t-te	0,39398876	permutation FDR (250 perm	1
2579	Pofut2~Q8VI	Pofut2	ko vs wt	ko	wt	28,3968015	0,29234293	27,9593863	0,20691224	1,35417586	0,43741511	0,35754912	0,10773047	0,96766145	Unpaired t-te	0,409727749	permutation FDR (250 perm	1
2580	Poglut1~Q8B	Poglut1	ko vs wt	ko	wt	26,3097046	0,66599159	26,0705022	0,19164362	1,18033992	0,23920239	0,16992536	0,59125585	0,22822455	Unpaired t-te	0,700395756	permutation FDR (250 perm	1
2581	Poglut3~G5E	Poglut3	ko vs wt	ko	wt	26,972097	0,31158115	26,5642589	0,10831352	1,32669628	0,40783813	0,34113959	0,10549426	0,97677118	Unpaired t-te	0,436166023	permutation FDR (250 perm	1
2582	Pold1~P5243	Pold1	ko vs wt	ko	wt	25,4461495	0,14475197	24,9928037	0,3696286	1,36921196	0,4533458	0,35573189	0,21864726	0,66025596	Unpaired t-te	0,43712283	permutation FDR (250 perm	1
2583	Poldip3~Q8B	Poldip3	ko vs wt	ko	wt	24,9082626	0,26236243	26,4183492	0,48994172	0,35109013	-1,5100867	-1,0957675	0,03255874	1,48733246	Unpaired t-te	0,043286385	permutation FDR (250 perm	1
2584	Polr2a~P087	Polr2a	ko vs wt	ko	wt	26,4777879	0,36451218	26,4420267	0,16043077	1,02509757	0,03576124	0,02886115	0,88765632	0,05175515	Unpaired t-te	0,949268215	permutation FDR (250 perm	1
2585	Polr2b~Q8CF	Polr2b	ko vs wt	ko	wt	27,8263842	0,10642711	27,1380292	0,04580877	1,61144504	0,68835498	0,64364678	0,00036157	3,44180844	Unpaired t-te	0,166278114	permutation FDR (250 perm	1
2586	Pon1~P5243(	Pon1	ko vs wt	ko	wt	28,9825651	1,06151069	27,1568428	0,25005244	3,54484436	1,82572229	1,11470017	0,05396893	1,26785616	Unpaired t-te	0,035113594	permutation FDR (250 perm	1
2587	Pon2~Q6208	Pon2	ko vs wt	ko	wt	28,2510403	0,20505572	28,3577003	0,28704821	0,92873568	-0,10666	-0,086366	0,67782283	0,16888381	Unpaired t-te	0,853182466	permutation FDR (250 perm	1
2588	Pon3~Q6208	Pon3	ko vs wt	ko	wt	28,5457071	0,37015491	28,7358278	0,25545664	0,87653238	-0,1901207	-0,1485524	0,52707059	0,27813122	Unpaired t-te	0,734036302	permutation FDR (250 perm	1
2589	Pop1~Q9D4G	Pop1	ko vs wt	ko	wt	23,5315893	0,14925058	23,6642851	1,10032758	0,91212549	-0,1326958	-0,0744308	0,88065579	0,05519381	Unpaired t-te	0,880871976	permutation FDR (250 perm	1
2590	Pop4~Q9CR0	Pop4	ko vs wt	ko	wt	23,7107164	0,69109379	23,96022	0,60655026	0,84118581	-0,2495036	-0,1573366	0,68918403	0,1616648	Unpaired t-te	0,719012543	permutation FDR (250 perm	1
2591	Por~P37040	Por	ko vs wt	ko	wt	31,2046123	0,13212453	30,9902673	0,27746057	1,16017707	0,21434501	0,17707115	0,39347411	0,40508384	Unpaired t-te	0,696587019	permutation FDR (250 perm	1
2592	Postn~Q620(	Postn	ko vs wt	ko	wt	37,1379128	0,12464494	37,3872716	0,12995272	0,84127021	-0,2493588	-0,2232965	0,09680496	1,01410238	Unpaired t-te	0,605757109	permutation FDR (250 perm	1
2593	Postn~Q620(	Postn	ko vs wt	ko	wt	30,7700291	0,96832608	30,7444581	0,2528931	1,01788251	0,02557104	0,01611316	0,9675654	0,01431967	Unpaired t-te	0,971786332	permutation FDR (250 perm	1
2594	Ppa1~Q9D81	Ppa1	ko vs wt	ko	wt	25,3190103	1,09732865	23,7193239	0,30528033	3,03077412	1,59968634	0,95829243	0,08110104	1,0909736	Unpaired t-te	0,059620782	permutation FDR (250 perm	1
2595	Ppa2~Q91VI	Ppa2	ko vs wt	ko	wt	24,5541826	0,70246618	23,8934723	0,58490768	1,58086074	0,66071028	0,41836642	0,30814185	0,51124931	Unpaired t-te	0,340986676	permutation FDR (250 perm	1
2596	Ppfia1~B2RX	Ppfia1	ko vs wt	ko	wt	26,7310143	0,1372098	27,1724874	0,62219862	0,73638229	-0,4414732	-0,3050879	0,42197094	0,37471746	Unpaired t-te	0,508479554	permutation FDR (250 perm	1
2597	Ppfibp1~Q8C	Ppfibp1	ko vs wt	ko	wt	29,6208526	0,43211561	29,4116429	0,55826224	1,15605476	0,20920973	0,1426126	0,68017608	0,16737865	Unpaired t-te	0,748757824	permutation FDR (250 perm	1
2598	Ppfibp2~O35	Ppfibp2	ko vs wt	ko	wt	24,2711622	0,7054158	24,8102212	0,96668477	0,68821965	-0,539059	-0,3001973	0,54151404	0,26639028	Unpaired t-te	0,496039875	permutation FDR (250 perm	1
2599	Ppia~P17742	Ppia	ko vs wt	ko	wt	33,0600962	0,31535919	32,8014951	0,0634063	1,19631816	0,25860112	0,21776721	0,25259664	0,59757243	Unpaired t-te	0,621002946	permutation FDR (250 perm	1
2600	Ppib~P24369	Ppib	ko vs wt	ko	wt	33,0478985	0,1873594	32,5118682	0,30763978	1,44997726	0,53603028	0,43125823	0,11480061	0,94005582	Unpaired t-te	0,343388813	permutation FDR (250 perm	1
2601	Ppic~P30412	Ppic	ko vs wt	ko	wt	27,9132143	0,54376602	25,114964	1,66490767	6,95596326	2,79825031	1,26137738	0,13247936	0,87785178	Unpaired t-te	0,030388638	permutation FDR (250 perm	1
2602	Ppid~Q9CR1(	Ppid	ko vs wt	ko	wt	27,2635299	0,57411241	25,9811462	0,46045596	2,43240538	1,28238368	0,87557018	0,0413206	1,38383334	Unpaired t-te	0,073041289	permutation FDR (250 perm	1
2603	Ppif~Q99KR7	Ppif	ko vs wt	ko	wt	25,6669411	2,78513597	23,849617	0,59805129	3,5242693	1,81732417	0,6825178	0,34562232	0,46139822	Unpaired t-te	0,154068117	permutation FDR (250 perm	1
2604	Ppig~A2AR0	Ppig	ko vs wt	ko	wt	25,7850165	1,31386192	26,7029375	0,06154352	0,52927117	-0,917921	-0,5216036	0,31304633	0,50439138	Unpaired t-te	0,25938606	permutation FDR (250 perm	1
2605	Ppil4~Q9CXG	Ppil4	ko vs wt	ko	wt	24,4155483	0,71904004	24,9114634	0,41053198	0,70911175	-0,4959151	-0,3291695	0,37345843	0,42775774	Unpaired t-te	0,446046065	permutation FDR (250 perm	1
2606	Ppip5k1~A2A	Ppip5k1	ko vs wt	ko	wt	24,211786	0,80759473	24,6937644	0,8380782	0,7159951	-0,4819784	-0,2747804	0,55608604	0,25485801	Unpaired t-te	0,526034356	permutation FDR (250 perm	1
2607	Ppl~G5E898	Ppl	ko vs wt	ko	wt	23,636726	0,34285241	24,7849767	1,27361525	0,45117196	-1,1482507	-0,5974002	0,3295112	0,48212982	Unpaired t-te	0,220852213	permutation FDR (250 perm	1
2608	Ppm1a~P494	Ppm1a	ko vs wt	ko	wt	24,5304024	0,74334896	25,8600246	1,10389118	0,3978724	-1,3296223	-0,7032161	0,22689763	0,64417005	Unpaired t-te	0,147181364	permutation FDR (250 perm	1
2609	Ppm1f~Q8C(	Ppm1f	ko vs wt	ko	wt	24,8967797	0,98618834	26,6489218	0,73203778	0,29686068	-1,7521421	-0,990191	0,07241424	1,14017601	Unpaired t-te	0,046958118	permutation FDR (250 perm	1
2610	Ppm1g~Q61(	Ppm1g	ko vs wt	ko	wt	24,7747623	1,17050543	25,1896843	1,20390146	0,75006005	-0,414922	-0,1988208	0,72117502	0,14195933	Unpaired t-te	0,647081412	permutation FDR (250 perm	1
2611	Ppm1h~Q3U	Ppm1h	ko vs wt	ko	wt	24,0654149	0,72511488	23,8032036	0,68324266	1,19931561	0,26221136	0,15995557	0,70064631	0,15450116	Unpaired t-te	0,715576	permutation FDR (250 perm	1
2612	Ppme1~Q8B'	Ppme1	ko vs wt	ko	wt	27,4188335	0,51726545	27,4096942	0,12899785	1,00635495	0,00913925	0,0069645	0,97822527	0,00956112	Unpaired t-te	0,987446587	permutation FDR (250 perm	1
2613	Ppp1ca~P62	Ppp1ca	ko vs wt	ko	wt	29,5614677	0,81281022	28,7163284	0,6842908	1,79643823	0,84513933	0,5048463	0,26821901	0,57151044	Unpaired t-te	0,257875362	permutation FDR (250 perm	1
2614	Ppp1cb~P62	Ppp1cb	ko vs wt	ko	wt	32,1164057	0,07266901	32,1821285	0,25033105	0,95546651	-0,0657228	-0,055607	0,74933265	0,12532535	Unpaired t-te	0,909770615	permutation FDR (250 perm	1
2615	Ppp1cc~P63(	Ppp1cc	ko vs wt	ko	wt	25,2797027	1,4094177	24,4244767	0,27615786	1,80904212	0,855226	0,4655989	0,37503794	0,4259248	Unpaired t-te	0,304813725	permutation FDR (250 perm	1
2616	Ppp1r12a~Q	Ppp1r12a	ko vs wt	ko	wt	31,0747605	0,94928017	32,213243	0,43824483	0,45423712	-1,1384825	-0,6986233	0,13640594	0,86516671	Unpaired t-te	0,138413181	permutation FDR (250 perm	1
2617	Ppp1r12b~Q	Ppp1r12b	ko vs wt	ko	wt	27,8073935	1,02233253	28,8258242	0,75242168	0,49365304	-1,0184307	-0,5674838	0,25670269	0,59056958	Unpaired t-te	0,209359209	permutation FDR (250 perm	1
2618	Ppp1r12c~Q	Ppp1r12c	ko vs wt	ko	wt	28,3892613	0,34461823	28,7279545	0,52909618	0,79075731	-0,3386931	-0,2378891	0,48041834	0,31838042	Unpaired t-te	0,589564439	permutation FDR (250 perm	1
2619	Ppp1r13l~Q5	Ppp1r13l	ko vs wt	ko	wt	24,2020247	1,13904896	24,2730959	0,31059321	0,95193096	-0,0710712	-0,0419711	0,92375152	0,03444483	Unpaired t-te	0,928123569	permutation FDR (250 perm	1
2620	Ppp1r14a~Q	Ppp1r14a	ko vs wt	ko	wt	24,7687396	0,99773465	25,287889	0,37148209	0,69778315	-0,5191493	-0,3178906	0,45710145	0,3399874	Unpaired t-te	0,465456618	permutation FDR (250 perm	1
2621	Ppp1r21~Q3	Ppp1r21	ko vs wt	ko	wt	27,066868	0,01824647	26,7394119	0,06714745	1,25479882	0,32745608	0,31226887	0,01663758	1,77890988	Unpaired t-te	0,496861768	permutation FDR (250 perm	1

2622	Ppp1r37~Q8I	Ppp1r37	ko vs wt	ko	wt	24,9693037	1,21183203	24,2222287	0,50445966	1,67838652	0,747075	0,41845073	0,391549	0,40721388	Unpaired t-te	0,343597544	permutation FDR (250 perm	1
2623	Ppp1r7~Q3U	Ppp1r7	ko vs wt	ko	wt	26,5754208	1,01994907	27,1738031	0,66493247	0,66049417	-0,5983823	-0,3412411	0,46316574	0,33426357	Unpaired t-te	0,430442649	permutation FDR (250 perm	1
2624	Ppp1r9b~Q6I	Ppp1r9b	ko vs wt	ko	wt	23,6791766	0,69157989	24,9091976	1,64496316	0,42631125	-1,230021	-0,5516313	0,40472189	0,39284331	Unpaired t-te	0,246527319	permutation FDR (250 perm	1
2625	Ppp2cb~P62	Ppp2cb	ko vs wt	ko	wt	28,7098586	0,35166065	28,1610691	0,24478498	1,46285772	0,54878946	0,43320966	0,09490381	1,02271634	Unpaired t-te	0,324133932	permutation FDR (250 perm	1
2626	Ppp2r1a~Q7	Ppp2r1a	ko vs wt	ko	wt	31,8404722	0,24454448	31,7217652	0,1010062	1,08576131	0,11870698	0,10249044	0,49201004	0,30802604	Unpaired t-te	0,82026099	permutation FDR (250 perm	1
2627	Ppp2r1b~Q7	Ppp2r1b	ko vs wt	ko	wt	24,7429353	1,32269785	24,3689816	0,50671827	1,29589939	0,37395372	0,20284556	0,67960379	0,1677442	Unpaired t-te	0,641161593	permutation FDR (250 perm	1
2628	Ppp2r2a~Q6I	Ppp2r2a	ko vs wt	ko	wt	28,3312887	0,49265716	28,2348496	0,2328733	1,06913137	0,09643913	0,07258365	0,78202787	0,10677777	Unpaired t-te	0,875765859	permutation FDR (250 perm	1
2629	Ppp2r2d~Q9	Ppp2r2d	ko vs wt	ko	wt	23,7904446	1,18977253	24,1140106	0,76505685	0,79909226	-0,323566	-0,1726273	0,72651388	0,13875609	Unpaired t-te	0,69012996	permutation FDR (250 perm	1
2630	Ppp2r5c~Q6	Ppp2r5c	ko vs wt	ko	wt	24,9076191	2,05133402	24,5593161	0,76144018	1,27306231	0,34830303	0,15137151	0,80182764	0,09591898	Unpaired t-te	0,731660613	permutation FDR (250 perm	1
2631	Ppp2r5d~Q9	Ppp2r5d	ko vs wt	ko	wt	27,7221678	0,70135261	27,3021009	0,42160389	1,33798966	0,42006696	0,2795169	0,44192372	0,35465269	Unpaired t-te	0,515552404	permutation FDR (250 perm	1
2632	Ppp2r5e~Q6	Ppp2r5e	ko vs wt	ko	wt	24,7893126	0,95395882	25,4263606	0,96836474	0,64302737	-0,6370479	-0,3390798	0,50646781	0,29544815	Unpaired t-te	0,436966227	permutation FDR (250 perm	1
2633	Ppp3ca~P63	Ppp3ca	ko vs wt	ko	wt	27,2285643	0,32390322	27,2667969	0,01765242	0,97384722	-0,0382326	-0,032198	0,85130648	0,06991406	Unpaired t-te	0,945715193	permutation FDR (250 perm	1
2634	Ppp6c~Q9CQ	Ppp6c	ko vs wt	ko	wt	26,2116252	0,85768053	25,1671988	0,95660377	2,06254622	1,04442645	0,56814729	0,28143447	0,55062271	Unpaired t-te	0,214446206	permutation FDR (250 perm	1
2635	Ppp6r3~Q92	Ppp6r3	ko vs wt	ko	wt	25,1018699	1,12394543	24,6060923	1,30249326	1,41008058	0,49577761	0,23312665	0,68349268	0,16526613	Unpaired t-te	0,591207154	permutation FDR (250 perm	1
2636	Ppt1~O8853	Ppt1	ko vs wt	ko	wt	27,8351154	0,87740699	28,0083132	0,50913162	0,88687467	-0,1731978	-0,1068155	0,79182568	0,10137042	Unpaired t-te	0,811347392	permutation FDR (250 perm	1
2637	Prag1~E9QL	Prag1	ko vs wt	ko	wt	23,5698125	0,59865562	24,2341493	0,29228533	0,63097868	-0,6643368	-0,4736083	0,16445586	0,78395066	Unpaired t-te	0,284474744	permutation FDR (250 perm	1
2638	Prdx1~P3570	Prdx1	ko vs wt	ko	wt	33,9061492	0,12070256	33,482279	0,28300546	1,34152151	0,42387019	0,34975619	0,15777465	0,80196276	Unpaired t-te	0,442290653	permutation FDR (250 perm	1
2639	Prdx2~Q611	Prdx2	ko vs wt	ko	wt	30,5206008	0,34649967	30,5000789	0,31462478	1,01432637	0,02052193	0,01579593	0,94822187	0,02309003	Unpaired t-te	0,971943441	permutation FDR (250 perm	1
2640	Prdx3~P2010	Prdx3	ko vs wt	ko	wt	26,9041913	1,69909146	26,6949002	1,74637651	1,15611999	0,20929114	0,08121213	0,90053601	0,04549891	Unpaired t-te	0,860365327	permutation FDR (250 perm	1
2641	Prdx4~O088	Prdx4	ko vs wt	ko	wt	30,3562252	0,06715564	30,6832462	0,14189174	0,79718087	-0,327021	-0,2952616	0,06704752	1,17361729	Unpaired t-te	0,513035847	permutation FDR (250 perm	1
2642	Prdx5~P9902	Prdx5	ko vs wt	ko	wt	30,2542787	0,51165247	29,5964975	0,24519145	1,57765439	0,6577812	0,48995889	0,11783594	0,92872225	Unpaired t-te	0,270871685	permutation FDR (250 perm	1
2643	Prdx6~Q6GT	Prdx6	ko vs wt	ko	wt	28,7652248	0,0895496	27,9269388	0,42447315	1,7879248	0,83828606	0,64257743	0,10379562	0,98382098	Unpaired t-te	0,196027788	permutation FDR (250 perm	1
2644	Preb~Q9WU	Preb	ko vs wt	ko	wt	28,3426132	0,25613075	27,7786618	0,2383307	1,4783127	0,56395147	0,46066706	0,05897296	1,22934707	Unpaired t-te	0,2984446	permutation FDR (250 perm	1
2645	Prelp~Q9JK5	Prelp	ko vs wt	ko	wt	35,5819755	0,24221715	35,1706091	0,28827249	1,3299449	0,41136647	0,3298325	0,17577426	0,75504473	Unpaired t-te	0,451978316	permutation FDR (250 perm	1
2646	Prep~Q9QU	Prep	ko vs wt	ko	wt	24,631412	1,4020994	24,0404204	0,56859425	1,50628179	0,59099169	0,31041933	0,54673122	0,26222613	Unpaired t-te	0,474880907	permutation FDR (250 perm	1
2647	Prex1~Q69Z	Prex1	ko vs wt	ko	wt	24,7841551	0,96424763	24,0517788	0,21124756	1,66137334	0,73237631	0,46458779	0,28339824	0,54760286	Unpaired t-te	0,304848739	permutation FDR (250 perm	1
2648	Prg2~Q6187	Prg2	ko vs wt	ko	wt	28,2596429	1,84459027	27,365738	1,22278708	1,85819876	0,89390483	0,37689227	0,54339542	0,26488403	Unpaired t-te	0,386886379	permutation FDR (250 perm	1
2649	Prg4~E9QQ1	Prg4	ko vs wt	ko	wt	31,5738882	0,69368531	30,3564983	0,54386886	2,32525651	1,21738988	0,78276431	0,08127881	1,09002268	Unpaired t-te	0,102312011	permutation FDR (250 perm	1
2650	Prkaa1~Q5E	Prkaa1	ko vs wt	ko	wt	28,9830594	0,32975956	28,8160328	0,27477967	1,12274214	0,16702663	0,13130738	0,56736532	0,24613721	Unpaired t-te	0,765749438	permutation FDR (250 perm	1
2651	Prkaa2~Q8B	Prkaa2	ko vs wt	ko	wt	26,5133057	0,37680206	25,0553089	1,02109252	2,74726625	1,45799673	0,83120155	0,17276556	0,76254282	Unpaired t-te	0,109868956	permutation FDR (250 perm	1
2652	Prkab2~Q6P	Prkab2	ko vs wt	ko	wt	25,6411238	1,48795695	26,8418885	0,03281685	0,43504463	-1,2007647	-0,6457857	0,25662754	0,59069673	Unpaired t-te	0,176970797	permutation FDR (250 perm	1
2653	Prkaca~P051	Prkaca	ko vs wt	ko	wt	24,9568276	1,30769851	24,9620051	1,46418528	0,99641771	-0,0051774	-0,0022694	0,99697204	0,00131702	Unpaired t-te	0,996127992	permutation FDR (250 perm	1
2654	Prkacb~P681	Prkacb	ko vs wt	ko	wt	28,731863	0,89255343	29,0651365	0,67013464	0,79373344	-0,3332735	-0,1960358	0,65437669	0,18417218	Unpaired t-te	0,649143524	permutation FDR (250 perm	1
2655	Prkag1~O54	Prkag1	ko vs wt	ko	wt	27,6218606	0,55293166	26,1700224	0,13391571	2,73556377	1,45183819	1,08916461	0,01602818	1,79511578	Unpaired t-te	0,037968523	permutation FDR (250 perm	1
2656	Prkar1a~Q9	Prkar1a	ko vs wt	ko	wt	28,7349789	0,67058095	28,2304021	0,61375004	1,41870714	0,50457681	0,31903299	0,42912389	0,3674173	Unpaired t-te	0,461195431	permutation FDR (250 perm	1
2657	Prkar2a~Q8	Prkar2a	ko vs wt	ko	wt	28,6965941	0,37178248	28,6349307	0,45941305	1,04366837	0,06166336	0,04438244	0,88250912	0,0542808	Unpaired t-te	0,924791033	permutation FDR (250 perm	1
2658	Prkar2b~P31	Prkar2b	ko vs wt	ko	wt	26,5901576	1,48864483	26,5964334	0,04155255	0,99565935	-0,0062759	-0,0033742	0,99463445	0,0023365	Unpaired t-te	0,993693028	permutation FDR (250 perm	1
2659	Prkca~Q4VA	Prkca	ko vs wt	ko	wt	28,9170503	1,39215897	26,5427194	1,09063854	5,18495292	2,37433089	1,12319721	0,08768383	1,05708049	Unpaired t-te	0,028242424	permutation FDR (250 perm	1
2660	Prkcd~P2886	Prkcd	ko vs wt	ko	wt	28,7188816	0,54153395	28,5196508	0,08947093	1,14808606	0,19923079	0,15104783	0,57346321	0,24149444	Unpaired t-te	0,736137072	permutation FDR (250 perm	1
2661	Prkcq~Q0211	Prkcq	ko vs wt	ko	wt	25,4407035	1,74278157	23,8744598	0,47738712	2,96132686	1,56624374	0,75982939	0,22073707	0,65612473	Unpaired t-te	0,119375723	permutation FDR (250 perm	1
2662	Prkd1~Q621	Prkd1	ko vs wt	ko	wt	24,4178371	1,09733532	24,7894374	0,82282251	0,77292463	-0,3716003	-0,1997664	0,68397085	0,16496241	Unpaired t-te	0,643185489	permutation FDR (250 perm	1
2663	Prkd2~Q8BZ	Prkd2	ko vs wt	ko	wt	24,8272959	0,18977706	23,8072654	0,21779469	2,02796185	1,02003051	0,85788679	0,00624141	2,20471718	Unpaired t-te	0,083045587	permutation FDR (250 perm	1
2664	Prkd3~Q8K1	Prkd3	ko vs wt	ko	wt	25,1431611	0,15792476	24,9535588	0,20502915	1,14044923	0,18960223	0,16187809	0,33799562	0,47108893	Unpaired t-te	0,71608795	permutation FDR (250 perm	1
2665	Prkg1~P0C60	Prkg1	ko vs wt	ko	wt	29,4258567	0,35218829	29,7672477	0,2504353	0,78927993	-0,341391	-0,2688886	0,26147082	0,58257678	Unpaired t-te	0,531011029	permutation FDR (250 perm	1
2666	Prkra~Q9WT	Prkra	ko vs wt	ko	wt	25,0237804	0,99864599	24,435336	0,55105391	1,50362453	0,58844436	0,34698283	0,43750015	0,3590218	Unpaired t-te	0,424777633	permutation FDR (250 perm	1
2667	Prmt1~Q9JIF	Prmt1	ko vs wt	ko	wt	27,8895038	0,14410316	27,8884932	0,11735184	1,00070072	0,00101058	0,00090431	0,99348368	0,00283926	Unpaired t-te	0,998530803	permutation FDR (250 perm	1
2668	Prmt3~Q922	Prmt3	ko vs wt	ko	wt	25,5423313	0,52261317	25,6012397	0,06936068	0,95999016	-0,0589085	-0,0451167	0,85892442	0,06604505	Unpaired t-te	0,924380139	permutation FDR (250 perm	1
2669	Prmt5~Q8CI	Prmt5	ko vs wt	ko	wt	26,1761421	0,30179086	24,5411307	1,03755171	3,10590009	1,63501142	0,93212634	0,14929919	0,82594256	Unpaired t-te	0,083130941	permutation FDR (250 perm	1
2670	Proc~P33587	Proc	ko vs wt	ko	wt	26,1167138	1,1630031	25,5054043	0,87019946	1,52764515	0,61130946	0,31993014	0,53221985	0,27390893	Unpaired t-te	0,457798538	permutation FDR (250 perm	1
2671	Prodh~Q9W	Prodh	ko vs wt	ko	wt	24,6598325	1,29385756	23,5949239	0,44382955	2,09203744	1,06490867	0,58826413	0,25958807	0,58571527	Unpaired t-te	0,201685583	permutation FDR (250 perm	1
2672	Pros1~Q087	Pros1	ko vs wt	ko	wt	27,7138558	0,45275389	27,374379	0,46780279	1,26529763	0,33947679	0,23879872	0,4639273	0,33355007	Unpaired t-te	0,58026938	permutation FDR (250 perm	1
2673	Prpf19~Q99	Prpf19	ko vs wt	ko	wt	29,5263476	0,29670608	29,5748368	0,3200229	0,96694836	-0,0484893	-0,0377696	0,8725212	0,05922401	Unpaired t-te	0,934556559	permutation FDR (250 perm	1
2674	Prpf31~Q8C	Prpf31	ko vs wt	ko	wt	26,208466	0,67663792	26,4065655	0,29504404	0,87169816	-0,1980994	-0,1372949	0,67572851	0,17022776	Unpaired t-te	0,754677918	permutation FDR (250 perm	1
2675	Prpf38a~Q4	Prpf38a	ko vs wt	ko	wt	27,3085848	0,60354023	27,3608817	0,52148332	0,96439966	-0,052297	-0,0346949	0,92216929	0,03518934	Unpaired t-te	0,939853581	permutation FDR (250 perm	1
2676	Prpf38b~Q8	Prpf38b	ko vs wt	ko	wt	24,1860779	0,28933449	26,2683493	0,30393006	0,23614234	-2,0822714	-1,6367515	0,00136477	2,86494008	Unpaired t-te	0,006240741	permutation FDR (250 perm	1
2677	Prpf3~Q922	Prpf3	ko vs wt	ko	wt	24,3793954	0,14749556	25,336653	1,55064105	0,515035	-0,9572576	-0,4558867	0,47490103	0,32339689	Unpaired t-te	0,339138167	permutation FDR (250 perm	1
2678	Prpf40a~Q9	Prpf40a	ko vs wt	ko	wt	28,3953753	0,37984499	28,1415168	0,39395785	1,19239193	0,25385852	0,1874137	0,51196469	0,29075999	Unpaired t-te	0,668352975	permutation FDR (250 perm	1
2679	Prpf4b~Q611	Prpf4b	ko vs wt	ko	wt	26,7667778	0,50980822	26,0499536	0,71101992	1,64356016	0,71682426	0,45294416	0,2980123	0,52576581	Unpaired t-te	0,317038528	permutation FDR (250 perm	1
2680	Prpf4~Q9DA	Prpf4	ko vs wt	ko	wt	27,9171964	0,81790156	27,4205238	0,06039957	1,41095568	0,49667267	0,33692296	0,37069914	0,43097843	Unpaired t-te	0,450566592	permutation FDR (250 perm	1
2681	Prpf6~Q91Y	Prpf6	ko vs wt	ko	wt	29,0948179	0,69890678	28,6643683	0,48849145	1,34765349	0,4304496	0,28112567	0,45461933	0,3423521	Unpaired t-te	0,513470952	permutation FDR (250 perm	1
2682	Prpf8~Q99P	Prpf8	ko vs wt	ko	wt	30,5909843	0,52184878	30,1531146	0,26607919	1,35460256	0,43786963	0,32310084	0,27553762	0,5598191	Unpaired t-te	0,45443362	permutation FDR (250 perm	1
2683	Prph~G5E84	Prph	ko vs wt	ko	wt	30,8265937	0,43601984	31,5983534	0,71372015	0,58570262	-0,7717597	-0,49346	0,26478012	0,57711462	Unpaired t-te	0,282163424	permutation FDR (250 perm	1
2684	Prps1~Q9D7	Prps1	ko vs wt	ko	wt	25,0058453	1,67937298	23,7489665	0,41133781	2,38978165	1,25687881	0,62460641	0,29064884	0,5366314	Unpaired t-te	0,182726204	permutation FDR (250 perm	1
2685	Prps2~Q9CS	Prps2	ko vs wt	ko	wt	27,3878861	0,69726397	26,6239155	0,26246307	1,69815792	0,76397063	0,52932728	0,15763534	0,80234641	Unpaired t-te	0,242357011	permutation FDR (250 perm	1

2686	Prpsap1~Q9	Prpsap1	ko vs wt	ko	wt	26,8913675	0,5783646	24,4708449	1,7045694	5,35364916	2,4205226	1,07544754	0,17524344	0,75635824	Unpaired t-te	0,053007804	permutation FDR (250 perm	1
2687	Prpsap2~Q8	Prpsap2	ko vs wt	ko	wt	26,4164422	0,6573705	24,5669055	0,84988819	3,60384447	1,84953675	1,08111082	0,06779413	1,16880788	Unpaired t-te	0,038728144	permutation FDR (250 perm	1
2688	Prr12~E9PYL	Prr12	ko vs wt	ko	wt	24,842667	1,41265655	27,2562273	0,09376159	0,18769208	-2,4135603	-1,3273807	0,05907941	1,22856388	Unpaired t-te	0,018742193	permutation FDR (250 perm	1
2689	Prrc1~Q3UP	Prrc1	ko vs wt	ko	wt	25,8120494	1,65469241	25,5287457	1,08608792	1,21697848	0,28330366	0,12728459	0,82637848	0,082821	Unpaired t-te	0,772585366	permutation FDR (250 perm	1
2690	Prss23~Q9D	Prss23	ko vs wt	ko	wt	28,7576394	0,65876211	28,6256507	0,87267702	1,09580319	0,13198871	0,0765209	0,86563516	0,06266511	Unpaired t-te	0,87122204	permutation FDR (250 perm	1
2691	Prune1~Q8B	Prune1	ko vs wt	ko	wt	25,5747773	0,64706082	25,3479342	0,32744893	1,17027134	0,22684307	0,15758292	0,62909034	0,20128698	Unpaired t-te	0,71837696	permutation FDR (250 perm	1
2692	Prxl2a~Q9CY	Prxl2a	ko vs wt	ko	wt	26,9541295	0,18106446	26,9753442	0,25410361	0,98540264	-0,0212148	-0,0175637	0,92455559	0,03406697	Unpaired t-te	0,97010438	permutation FDR (250 perm	1
2693	Prxl2b~Q9DB	Prxl2b	ko vs wt	ko	wt	27,4825181	0,58032712	25,9324278	0,40874331	2,92835468	1,5500903	1,07459513	0,01731051	1,76169009	Unpaired t-te	0,034163112	permutation FDR (250 perm	1
2694	Prx~O55103	Prx	ko vs wt	ko	wt	24,5359887	0,45644364	24,3295008	1,28645157	1,15387572	0,20648784	0,10605101	0,84498556	0,07315072	Unpaired t-te	0,822501657	permutation FDR (250 perm	1
2695	Psap~E9PZ0	Psap	ko vs wt	ko	wt	27,1714237	1,13085272	25,5481573	1,91297369	3,08071747	1,62326638	0,64878699	0,36054254	0,44304349	Unpaired t-te	0,17660443	permutation FDR (250 perm	1
2696	Psip1~Q99JF	Psip1	ko vs wt	ko	wt	26,918217	2,0385972	25,4221863	1,75035363	2,82065588	1,49603067	0,55245465	0,42362834	0,37301499	Unpaired t-te	0,220549137	permutation FDR (250 perm	1
2697	Psma1~Q9R	Psma1	ko vs wt	ko	wt	28,070372	0,22001816	27,7337478	0,80701965	1,26279835	0,33662428	0,21243266	0,61821931	0,20885744	Unpaired t-te	0,643547236	permutation FDR (250 perm	1
2698	Psma2~P497	Psma2	ko vs wt	ko	wt	27,8050348	0,76905775	27,7660701	0,87740529	1,02737632	0,03896473	0,02210219	0,96178086	0,01692387	Unpaired t-te	0,961610308	permutation FDR (250 perm	1
2699	Psma3~O70	Psma3	ko vs wt	ko	wt	28,600377	0,69058904	28,3777325	0,82455382	1,16687047	0,22264442	0,13048072	0,76931567	0,11389542	Unpaired t-te	0,770520471	permutation FDR (250 perm	1
2700	Psma4~Q9R	Psma4	ko vs wt	ko	wt	26,0642053	0,94029676	26,8296712	1,19021064	0,58826338	-0,7654659	-0,3824444	0,49173957	0,30826484	Unpaired t-te	0,390530504	permutation FDR (250 perm	1
2701	Psma5~Q9Z	Psma5	ko vs wt	ko	wt	27,4479664	0,56225764	26,4997331	0,72241725	1,92950838	0,94823331	0,59070949	0,20098843	0,69682895	Unpaired t-te	0,20400766	permutation FDR (250 perm	1
2702	Psma6~Q9Q	Psma6	ko vs wt	ko	wt	28,954676	0,31815419	28,3048888	0,6858114	1,56893673	0,64978717	0,42789562	0,31175692	0,5061839	Unpaired t-te	0,354057608	permutation FDR (250 perm	1
2703	Psma7~Q9Z	Psma7	ko vs wt	ko	wt	28,4681031	0,49136024	26,8958517	1,28803235	2,97368394	1,57225132	0,80465897	0,22038132	0,65682522	Unpaired t-te	0,119168669	permutation FDR (250 perm	1
2704	Psmb1~O09	Psmb1	ko vs wt	ko	wt	28,171222	0,68452249	27,7209248	0,61329187	1,36632172	0,45029723	0,28378918	0,48038874	0,31840718	Unpaired t-te	0,511278689	permutation FDR (250 perm	1
2705	Psmb3~Q9R	Psmb3	ko vs wt	ko	wt	27,5622749	0,5909206	27,6013477	0,39488653	0,97328025	-0,0390728	-0,0271176	0,9328185	0,03020285	Unpaired t-te	0,951868109	permutation FDR (250 perm	1
2706	Psmb4~P990	Psmb4	ko vs wt	ko	wt	26,338102	0,29330908	25,363836	1,01984886	1,96464146	0,97426605	0,55967916	0,30771302	0,51185412	Unpaired t-te	0,24653612	permutation FDR (250 perm	1
2707	Psmb5~O55	Psmb5	ko vs wt	ko	wt	28,1710006	0,30521524	28,1782839	0,04330874	0,99496428	-0,0072834	-0,0061783	0,96994126	0,01325456	Unpaired t-te	0,989117999	permutation FDR (250 perm	1
2708	Psmb6~Q60	Psmb6	ko vs wt	ko	wt	25,4731292	1,26619305	24,4059187	0,56256084	2,09537806	1,06721056	0,5824567	0,2624125	0,58101548	Unpaired t-te	0,20204908	permutation FDR (250 perm	1
2709	Psmc1~P621	Psmc1	ko vs wt	ko	wt	27,737533	0,27857263	27,5602856	0,91964466	1,13072445	0,1772474	0,10614372	0,81365236	0,08956111	Unpaired t-te	0,822990334	permutation FDR (250 perm	1
2710	Psmc2~P464	Psmc2	ko vs wt	ko	wt	29,6502671	0,38798182	29,2666954	0,09138529	1,3045676	0,38357171	0,31105392	0,18570333	0,73118031	Unpaired t-te	0,480489308	permutation FDR (250 perm	1
2711	Psmc3~O88	Psmc3	ko vs wt	ko	wt	28,6772093	0,51150537	28,1048878	0,39800949	1,48691424	0,57232144	0,40649444	0,22091354	0,65577766	Unpaired t-te	0,353726687	permutation FDR (250 perm	1
2712	Psmc4~P547	Psmc4	ko vs wt	ko	wt	29,8066962	0,5593298	29,9108747	0,09159058	0,93033455	-0,1041785	-0,0783674	0,77107516	0,11290329	Unpaired t-te	0,868153888	permutation FDR (250 perm	1
2713	Psmc5~P621	Psmc5	ko vs wt	ko	wt	30,4602649	0,44506961	30,5748937	0,48003516	0,92361988	-0,1146289	-0,0804001	0,80086572	0,0964403	Unpaired t-te	0,86225041	permutation FDR (250 perm	1
2714	Psmc6~P623	Psmc6	ko vs wt	ko	wt	28,814339	0,1372271	28,867261	0,0921572	0,96398191	-0,052922	-0,0479982	0,62792798	0,20209016	Unpaired t-te	0,916120888	permutation FDR (250 perm	1
2715	Psmd10~Q9	Psmd10	ko vs wt	ko	wt	24,2335649	1,03754533	24,2054398	0,55176144	1,01968611	0,02812512	0,01640063	0,9702036	0,01313712	Unpaired t-te	0,970966728	permutation FDR (250 perm	1
2716	Psmd11~Q8	Psmd11	ko vs wt	ko	wt	29,7987485	0,30575559	29,7151411	0,08931832	1,05966439	0,08360742	0,07040707	0,68031915	0,16728731	Unpaired t-te	0,881461267	permutation FDR (250 perm	1
2717	Psmd12~Q9	Psmd12	ko vs wt	ko	wt	29,3432422	0,38895072	28,4635811	0,52625652	1,83994301	0,87966108	0,61316367	0,12484804	0,90361827	Unpaired t-te	0,191170485	permutation FDR (250 perm	1
2718	Psmd13~Q9	Psmd13	ko vs wt	ko	wt	28,9435186	0,31776181	28,3528792	0,23550868	1,50591399	0,59063937	0,47335618	0,06343266	1,19768706	Unpaired t-te	0,282894942	permutation FDR (250 perm	1
2719	Psmd14~O3	Psmd14	ko vs wt	ko	wt	25,0634596	1,8457725	27,8263987	0,15758703	0,14732364	-2,7629391	-1,3338077	0,08011186	1,09630321	Unpaired t-te	0,018357247	permutation FDR (250 perm	1
2720	Psmd1~Q3T	Psmd1	ko vs wt	ko	wt	29,3234626	0,20876847	28,4748516	1,31297957	1,80076631	0,84861097	0,43828498	0,45768056	0,33943753	Unpaired t-te	0,355565041	permutation FDR (250 perm	1
2721	Psmd2~Q8V	Psmd2	ko vs wt	ko	wt	27,7143856	0,76141213	27,9294623	0,92564955	0,8615004	-0,2150766	-0,1202583	0,79952954	0,09716548	Unpaired t-te	0,790023451	permutation FDR (250 perm	1
2722	Psmd3~P146	Psmd3	ko vs wt	ko	wt	29,5627699	0,43880614	29,4515071	0,12515307	1,08017327	0,11126275	0,08772198	0,70136156	0,15405804	Unpaired t-te	0,849672428	permutation FDR (250 perm	1
2723	Psmd5~Q8B	Psmd5	ko vs wt	ko	wt	27,5611667	0,90869775	27,3239132	0,00550401	1,17874652	0,23725351	0,15561165	0,68180977	0,16633678	Unpaired t-te	0,730100483	permutation FDR (250 perm	1
2724	Psmd6~Q99	Psmd6	ko vs wt	ko	wt	28,1961272	0,98504385	27,9047626	0,58960263	1,22379727	0,29136458	0,17087213	0,69676568	0,15691325	Unpaired t-te	0,69319391	permutation FDR (250 perm	1
2725	Psmd7~P265	Psmd7	ko vs wt	ko	wt	28,8062724	0,30640717	27,9315215	0,69293883	1,83369144	0,8747509	0,57513879	0,20846881	0,68095891	Unpaired t-te	0,228845091	permutation FDR (250 perm	1
2726	Psmd8~Q9C	Psmd8	ko vs wt	ko	wt	28,7208648	0,73933488	28,1886821	0,81897037	1,44611539	0,53218268	0,30951335	0,50071053	0,30041328	Unpaired t-te	0,47854248	permutation FDR (250 perm	1
2727	Psme1~P973	Psme1	ko vs wt	ko	wt	29,1356604	0,41328443	29,0158456	0,65842285	1,08659535	0,11981478	0,07866205	0,83335905	0,07916784	Unpaired t-te	0,86825648	permutation FDR (250 perm	1
2728	Psme2~P973	Psme2	ko vs wt	ko	wt	26,0413055	1,15067169	26,9251493	0,38850285	0,54192165	-0,8838438	-0,5141918	0,28721535	0,54179235	Unpaired t-te	0,255551524	permutation FDR (250 perm	1
2729	Pspc1~Q8R3	Pspc1	ko vs wt	ko	wt	28,490056	0,27200232	28,1767251	0,22440775	1,24257327	0,31333092	0,25614598	0,22198416	0,65367802	Unpaired t-te	0,55138695	permutation FDR (250 perm	1
2730	Ptbp1~Q8BG	Ptbp1	ko vs wt	ko	wt	31,5610692	0,28566326	31,4917443	0,24689201	1,04922557	0,06932488	0,05589974	0,78519271	0,10502374	Unpaired t-te	0,902646948	permutation FDR (250 perm	1
2731	Ptbp2~Q91Z	Ptbp2	ko vs wt	ko	wt	24,4046092	1,30693399	24,3542942	0,5552476	1,035491	0,05031501	0,02718859	0,95544118	0,01979604	Unpaired t-te	0,951932489	permutation FDR (250 perm	1
2732	Ptbp3~G8JL7	Ptbp3	ko vs wt	ko	wt	27,7189841	0,94489213	26,8744016	0,11895561	1,79574504	0,84458253	0,54419668	0,21946322	0,65863826	Unpaired t-te	0,240710129	permutation FDR (250 perm	1
2733	Ptges2~Q8B	Ptges2	ko vs wt	ko	wt	27,5973845	0,19771879	25,1847852	1,84352585	5,32432726	2,41259925	1,04506752	0,20462304	0,68904546	Unpaired t-te	0,063066246	permutation FDR (250 perm	1
2734	Ptges3~Q9R	Ptges3	ko vs wt	ko	wt	29,1610148	0,38034794	29,0922418	0,35595103	1,04882427	0,06877297	0,05155302	0,84590858	0,07267657	Unpaired t-te	0,911186695	permutation FDR (250 perm	1
2735	Ptgfrn~Q9W	Ptgfrn	ko vs wt	ko	wt	27,4523864	0,08207131	27,519242	0,25673239	0,95471658	-0,0668556	-0,0562938	0,75197138	0,12379869	Unpaired t-te	0,907939833	permutation FDR (250 perm	1
2736	Ptgis~O3507	Ptgis	ko vs wt	ko	wt	31,5150983	0,36766257	31,5021957	0,09007277	1,00898352	0,01290262	0,0105619	0,95672406	0,0192133	Unpaired t-te	0,982420063	permutation FDR (250 perm	1
2737	Ptgr3~Q8BG	Ptgr3	ko vs wt	ko	wt	27,376387	0,66596184	26,8805634	0,65490234	1,4101255	0,49582356	0,30952217	0,45299291	0,3439086	Unpaired t-te	0,475774488	permutation FDR (250 perm	1
2738	Ptgs1~P2243	Ptgs1	ko vs wt	ko	wt	24,3163537	1,80998437	25,1717779	1,05408466	0,5527028	-0,8554242	-0,3745989	0,53510573	0,2715604	Unpaired t-te	0,390393367	permutation FDR (250 perm	1
2739	Pth1r~P4159	Pth1r	ko vs wt	ko	wt	23,9435879	0,90594357	24,1487351	0,16849037	0,86745021	-0,2051471	-0,1335207	0,72542833	0,13940549	Unpaired t-te	0,767052484	permutation FDR (250 perm	1
2740	Ptk2~P34152	Ptk2	ko vs wt	ko	wt	28,7678741	0,16794381	28,9906656	0,32972077	0,85690578	-0,2227915	-0,1778765	0,44911948	0,34763811	Unpaired t-te	0,693959207	permutation FDR (250 perm	1
2741	Ptpa~P5838	Ptpa	ko vs wt	ko	wt	26,7881612	0,54826619	27,0363985	0,34943733	0,84192442	-0,2482374	-0,1771148	0,5635744	0,24904874	Unpaired t-te	0,682202481	permutation FDR (250 perm	1
2742	Ptpn11~P352	Ptpn11	ko vs wt	ko	wt	28,7847978	0,81458662	28,3058333	0,63106267	1,39374299	0,47896455	0,29057944	0,49394877	0,30631809	Unpaired t-te	0,499588308	permutation FDR (250 perm	1
2743	Ptpn12~P35	Ptpn12	ko vs wt	ko	wt	24,4872707	1,75107195	24,8646739	1,21104732	0,76982199	-0,3774032	-0,1623298	0,78719442	0,10391799	Unpaired t-te	0,708893848	permutation FDR (250 perm	1
2744	Ptpn1~P3582	Ptpn1	ko vs wt	ko	wt	28,4383628	0,92810063	27,632982	0,31494037	1,74760703	0,80538082	0,50964648	0,23832809	0,62282476	Unpaired t-te	0,258836705	permutation FDR (250 perm	1
2745	Ptpn23~Q6P	Ptpn23	ko vs wt	ko	wt	27,6202019	0,11050464	27,9102782	0,25260238	0,81785877	-0,2900764	-0,2438294	0,24039955	0,61906634	Unpaired t-te	0,587677612	permutation FDR (250 perm	1
2746	Ptpn2~Q061	Ptpn2	ko vs wt	ko	wt	24,7985715	0,68897116	24,5676631	0,75204476	1,17357372	0,23090847	0,13875978	0,74548511	0,12756103	Unpaired t-te	0,753838964	permutation FDR (250 perm	1
2747	Ptpn6~P293	Ptpn6	ko vs wt	ko	wt	28,3192808	0,77785071	28,4834742	0,45801442	0,89242731	-0,1641934	-0,1056797	0,77889764	0,10851961	Unpaired t-te	0,812472999	permutation FDR (250 perm	1
2748	Ptpn9~O352	Ptpn9	ko vs wt	ko	wt	24,9831493	0,90890051	26,0950311	0,31468403	0,46269012	-1,1118818	-0,708213	0,12316236	0,909522	Unpaired t-te	0,137899281	permutation FDR (250 perm	1
2749	Ptpra~P1805	Ptpra	ko vs wt	ko	wt	26,6589383	0,29545111	26,2060486	0,25800613	1,3687792	0,45288974	0,362381	0,13385429	0,87336769	Unpaired t-te	0,405799803	permutation FDR (250 perm	1

2750	Ptprc~P0680	Ptprc	ko vs wt	ko	wt	27,69871	1,26640273	27,1894362	0,500468	1,4233336	0,50927384	0,28101017	0,56299858	0,2494927	Unpaired t-te	0,517301695	permutation FDR (250 perm	1
2751	Ptprd~Q6448	Ptprd	ko vs wt	ko	wt	24,3755429	1,17859318	26,1014196	0,08727329	0,30231475	-1,7258767	-1,0253218	0,08437386	1,07379206	Unpaired t-te	0,052011192	permutation FDR (250 perm	1
2752	Ptpre~P4944	Ptpre	ko vs wt	ko	wt	24,8156225	0,88563778	23,974468	0,48035803	1,79148307	0,84115441	0,52120676	0,23093738	0,63650577	Unpaired t-te	0,244167524	permutation FDR (250 perm	1
2753	Ptprf~A2A8L	Ptprf	ko vs wt	ko	wt	24,3025266	0,51738853	23,8855121	0,76308888	1,33516178	0,41701456	0,25793359	0,54444422	0,26404661	Unpaired t-te	0,558494858	permutation FDR (250 perm	1
2754	Ptprg~Q059C	Ptprg	ko vs wt	ko	wt	25,4831286	1,28529901	23,864484	0,75924729	3,07086399	1,61864462	0,84484338	0,13820986	0,85946098	Unpaired t-te	0,080978953	permutation FDR (250 perm	1
2755	Ptprj~Q6445	Ptprj	ko vs wt	ko	wt	25,0381945	1,60960757	26,5013122	0,55218385	0,36270846	-1,4631177	-0,728644	0,22083688	0,65592839	Unpaired t-te	0,129278547	permutation FDR (250 perm	1
2756	Ptprs~B0V2N	Ptprs	ko vs wt	ko	wt	25,5979043	0,50189175	25,7341567	0,16772031	0,90987961	-0,1362524	-0,1037641	0,68629756	0,16348754	Unpaired t-te	0,81902323	permutation FDR (250 perm	1
2757	Ptprz1~B9EK	Ptprz1	ko vs wt	ko	wt	24,3269702	0,84842256	25,601646	1,77701292	0,41331804	-1,2746758	-0,5427295	0,42350779	0,3731386	Unpaired t-te	0,249781182	permutation FDR (250 perm	1
2758	Ptrh2~Q8R2Y	Ptrh2	ko vs wt	ko	wt	26,9936766	0,20713067	27,3575469	0,50489305	0,7770771	-0,3638703	-0,2643427	0,41917922	0,37760026	Unpaired t-te	0,558517846	permutation FDR (250 perm	1
2759	Puf60~Q3UE	Puf60	ko vs wt	ko	wt	27,4721032	0,25867516	27,7862427	0,02604988	0,80433059	-0,3141395	-0,2730513	0,12539395	0,90172342	Unpaired t-te	0,538175374	permutation FDR (250 perm	1
2760	Pum1~E9Q6I	Pum1	ko vs wt	ko	wt	25,2212765	0,64010311	24,3424038	0,20127424	1,83893775	0,87887264	0,62955526	0,09369346	1,02829071	Unpaired t-te	0,17720442	permutation FDR (250 perm	1
2761	Pum2~Q80U	Pum2	ko vs wt	ko	wt	24,7253357	0,86815174	24,5834138	1,66392003	1,10337397	0,14192184	0,06227691	0,91928018	0,0365521	Unpaired t-te	0,89540577	permutation FDR (250 perm	1
2762	Pum3~Q8BK	Pum3	ko vs wt	ko	wt	25,7764187	0,49821095	25,4565788	0,15424065	1,24819201	0,31983988	0,24459602	0,35995018	0,4437576	Unpaired t-te	0,574045701	permutation FDR (250 perm	1
2763	Pura~P4266	Pura	ko vs wt	ko	wt	28,9913713	0,14907003	28,9166978	0,35492751	1,05312267	0,0746735	0,05901556	0,80020488	0,0967988	Unpaired t-te	0,902130751	permutation FDR (250 perm	1
2764	Purb~O3529	Purb	ko vs wt	ko	wt	25,1779309	0,93157836	27,1215646	0,19513198	0,25996085	-1,9436337	-1,2497134	0,03257402	1,48712858	Unpaired t-te	0,022215427	permutation FDR (250 perm	1
2765	Pusl1~A2AD	Pusl1	ko vs wt	ko	wt	24,6453666	0,54850895	24,825847	0,71206288	0,88240909	-0,1804804	-0,1131671	0,7786058	0,10868236	Unpaired t-te	0,8042516	permutation FDR (250 perm	1
2766	Pvalb~P3284	Pvalb	ko vs wt	ko	wt	25,4873905	1,24238285	24,1980621	0,29139691	2,44414248	1,28932839	0,73832082	0,16972708	0,77024886	Unpaired t-te	0,129413495	permutation FDR (250 perm	1
2767	Pxdn~Q3UQ	Pxdn	ko vs wt	ko	wt	32,591843	0,65899094	31,7072274	0,24624125	1,84627271	0,88461566	0,62366354	0,10003699	0,9998394	Unpaired t-te	0,178330969	permutation FDR (250 perm	1
2768	Pxk~Q8BX57	Pxk	ko vs wt	ko	wt	27,0780621	0,46844511	24,736919	1,30971205	5,0670397	2,34114313	1,19154877	0,11750137	0,92995708	Unpaired t-te	0,036193509	permutation FDR (250 perm	1
2769	Pxn~Q8VI36	Pxn	ko vs wt	ko	wt	30,1890749	0,38849726	30,1531447	0,54003491	1,0252176	0,03593015	0,02490196	0,93994353	0,02689824	Unpaired t-te	0,955955801	permutation FDR (250 perm	1
2770	Pycard~Q9EF	Pycard	ko vs wt	ko	wt	24,7047157	0,43112625	24,8698572	0,76547368	0,89184105	-0,1651415	-0,1034876	0,80050871	0,09663394	Unpaired t-te	0,823403644	permutation FDR (250 perm	1
2771	Pycr1~Q922V	Pycr1	ko vs wt	ko	wt	27,0618702	0,80511808	27,2096703	0,1453079	0,90262575	-0,1478002	-0,1001318	0,77485653	0,1107787	Unpaired t-te	0,82796139	permutation FDR (250 perm	1
2772	Pycr2~Q922C	Pycr2	ko vs wt	ko	wt	29,277364	0,29430329	29,1750719	0,66895302	1,07347761	0,1022921	0,06807611	0,85401366	0,06853518	Unpaired t-te	0,887305383	permutation FDR (250 perm	1
2773	Pycr3~Q9DC	Pycr3	ko vs wt	ko	wt	27,9650344	0,48686111	28,087347	0,25516063	0,91871381	-0,1223126	-0,0916877	0,72990799	0,13673188	Unpaired t-te	0,839811933	permutation FDR (250 perm	1
2774	Pygb~Q8CI94	Pygb	ko vs wt	ko	wt	32,022586	0,36875316	31,7471263	0,21055318	1,21037968	0,27545967	0,21865464	0,33849157	0,47045214	Unpaired t-te	0,610126552	permutation FDR (250 perm	1
2775	Pygl~Q9ET01	Pygl	ko vs wt	ko	wt	27,9860199	1,27212612	25,5522207	0,52517726	5,40314402	2,43379914	1,33504651	0,03800028	1,42021321	Unpaired t-te	0,014164251	permutation FDR (250 perm	1
2776	Pygm~Q9WL	Pygm	ko vs wt	ko	wt	30,6257284	0,28184444	30,3149811	0,58223813	1,24035001	0,31074728	0,21539352	0,53969559	0,26785113	Unpaired t-te	0,632976785	permutation FDR (250 perm	1
2777	Pyhin1~Q8B	Pyhin1	ko vs wt	ko	wt	25,1923096	0,96646824	25,193133	0,75429916	0,99942944	-0,0008234	-0,0004647	0,99919115	0,00035142	Unpaired t-te	0,999394853	permutation FDR (250 perm	1
2778	Pzp~Q61838	Pzp	ko vs wt	ko	wt	34,3399069	0,44005039	33,5397034	0,29790087	1,7413468	0,80020355	0,6016427	0,05978641	1,22339756	Unpaired t-te	0,186255369	permutation FDR (250 perm	1
2779	QNG1~G3X8	QNG1	ko vs wt	ko	wt	24,6473176	0,94480628	24,8597069	1,40739742	0,86310661	-0,2123893	-0,0994857	0,86282044	0,06407957	Unpaired t-te	0,829682934	permutation FDR (250 perm	1
2780	Qars1~Q8BM	Qars1	ko vs wt	ko	wt	30,4815037	0,293646	30,0296445	0,16893541	1,36780182	0,45185921	0,3742434	0,08229843	1,08460844	Unpaired t-te	0,390837256	permutation FDR (250 perm	1
2781	Qdpr~Q8BVI	Qdpr	ko vs wt	ko	wt	26,562942	0,36316684	24,0532972	0,58807182	5,69479832	2,50964475	1,71224853	0,01234568	1,90848492	Unpaired t-te	0,007026911	permutation FDR (250 perm	1
2782	Qki~Q9QYS9	Qki	ko vs wt	ko	wt	27,8572984	0,30552987	27,5060939	0,33805606	1,2756252	0,35120451	0,27076529	0,30285477	0,51876559	Unpaired t-te	0,533682987	permutation FDR (250 perm	1
2783	Qpct~Q9CYK	Qpct	ko vs wt	ko	wt	25,5046996	1,37443644	26,4023267	0,68403057	0,53676885	-0,8976271	-0,465255	0,38136096	0,41866377	Unpaired t-te	0,292790436	permutation FDR (250 perm	1
2784	Qrich1~Q3U	Qrich1	ko vs wt	ko	wt	24,7671304	0,57097555	24,1750291	0,54750361	1,50744074	0,59210128	0,39251537	0,30337615	0,51801856	Unpaired t-te	0,372718121	permutation FDR (250 perm	1
2785	Qsox1~Q8BN	Qsox1	ko vs wt	ko	wt	28,2896495	0,10846557	27,7885507	0,22087456	1,41529113	0,50109885	0,42892429	0,06857891	1,1638094	Unpaired t-te	0,352515296	permutation FDR (250 perm	1
2786	RTRAF~Q9C	RTRAF	ko vs wt	ko	wt	28,2111909	0,41240473	27,9507042	0,98589456	1,19788277	0,26048673	0,14999184	0,75159062	0,12401865	Unpaired t-te	0,743963842	permutation FDR (250 perm	1
2787	Rab10~P610	Rab10	ko vs wt	ko	wt	29,5193235	0,8170479	28,2967273	0,28194085	2,33366301	1,22259625	0,80853079	0,07575861	1,12056802	Unpaired t-te	0,097311748	permutation FDR (250 perm	1
2788	Rab11b~P46	Rab11b	ko vs wt	ko	wt	30,8261592	0,77064664	30,2580018	0,56223016	1,48262873	0,56815737	0,35583863	0,38505071	0,41448207	Unpaired t-te	0,412082353	permutation FDR (250 perm	1
2789	Rab11fip5~C	Rab11fip5	ko vs wt	ko	wt	27,8727479	0,51895543	28,1075698	0,18656459	0,84978989	-0,2348219	-0,1769069	0,51241426	0,29037879	Unpaired t-te	0,68606563	permutation FDR (250 perm	1
2790	Rab12~P352	Rab12	ko vs wt	ko	wt	25,3213135	0,65021435	24,6806975	1,10932612	1,55899461	0,64061594	0,3426461	0,51605741	0,28730198	Unpaired t-te	0,444914213	permutation FDR (250 perm	1
2791	Rab14~Q91V	Rab14	ko vs wt	ko	wt	30,3110278	0,48827295	29,7366266	0,40761945	1,48905926	0,57440117	0,40935931	0,21642647	0,66468963	Unpaired t-te	0,352028562	permutation FDR (250 perm	1
2792	Rab18~P352	Rab18	ko vs wt	ko	wt	28,6317885	0,05031506	28,3758296	0,46105	1,19412915	0,25595888	0,1928413	0,51497175	0,28821659	Unpaired t-te	0,679250217	permutation FDR (250 perm	1
2793	Rab1A~P628	Rab1A	ko vs wt	ko	wt	31,209949	0,24132311	31,027365	0,33657689	1,13491475	0,18258394	0,1431156	0,55073015	0,25906115	Unpaired t-te	0,748965323	permutation FDR (250 perm	1
2794	Rab1a~Q5SV	Rab1a	ko vs wt	ko	wt	25,3532032	2,01095593	23,4367889	0,66827422	3,77483672	1,91641424	0,85041465	0,2025469	0,69347439	Unpaired t-te	0,084934489	permutation FDR (250 perm	1
2795	Rab1b~Q9D1	Rab1b	ko vs wt	ko	wt	27,717769	0,41764216	28,4303324	0,58952892	0,6102349	-0,7125634	-0,4809502	0,22712508	0,64373492	Unpaired t-te	0,28963156	permutation FDR (250 perm	1
2796	Rab21~P352	Rab21	ko vs wt	ko	wt	28,9588646	0,60610739	28,1916765	0,24832305	1,70194935	0,7671881	0,55133117	0,11677859	0,93263677	Unpaired t-te	0,224357383	permutation FDR (250 perm	1
2797	Rab23~Q9D4	Rab23	ko vs wt	ko	wt	26,5097763	0,18025978	25,6085338	0,98313338	1,8676738	0,9012425	0,52923134	0,32360639	0,48998291	Unpaired t-te	0,271396712	permutation FDR (250 perm	1
2798	Rab27b~Q99	Rab27b	ko vs wt	ko	wt	25,8099228	1,5141929	24,2169981	0,8233452	3,01660282	1,59292476	0,77690859	0,19186992	0,71699311	Unpaired t-te	0,104631001	permutation FDR (250 perm	1
2799	Rab28~Q99K	Rab28	ko vs wt	ko	wt	24,5280727	0,68032202	25,0560717	0,71737885	0,69351596	-0,527999	-0,3216452	0,45540017	0,34160681	Unpaired t-te	0,460251429	permutation FDR (250 perm	1
2800	Rab29~E9QL	Rab29	ko vs wt	ko	wt	24,8505204	1,93182044	24,0279127	0,55001841	1,76859983	0,82260766	0,37713511	0,52750896	0,27777016	Unpaired t-te	0,395239604	permutation FDR (250 perm	1
2801	Rab2a~P539	Rab2a	ko vs wt	ko	wt	29,8227111	0,44037927	29,7580987	0,47029792	1,04580389	0,06461235	0,04554622	0,88465233	0,05322738	Unpaired t-te	0,922147515	permutation FDR (250 perm	1
2802	Rab31~Q921	Rab31	ko vs wt	ko	wt	25,5992573	1,17747187	26,1582019	0,18709015	0,67879853	-0,5589447	-0,330235	0,47490422	0,32339397	Unpaired t-te	0,456960865	permutation FDR (250 perm	1
2803	Rab32~Q9CZ	Rab32	ko vs wt	ko	wt	25,6792668	1,51198484	24,746282	0,46237237	1,90922193	0,93298481	0,48287079	0,37644494	0,42429854	Unpaired t-te	0,282378493	permutation FDR (250 perm	1
2804	Rab34~Q640	Rab34	ko vs wt	ko	wt	27,2608806	0,11758662	26,7861188	0,07019332	1,38968869	0,47476173	0,43793261	0,00250821	2,60063678	Unpaired t-te	0,319008654	permutation FDR (250 perm	1
2805	Rab35~Q6PH	Rab35	ko vs wt	ko	wt	28,5334612	0,40799017	28,9969554	0,16881315	0,72522764	-0,4634942	-0,3666674	0,14887297	0,82718414	Unpaired t-te	0,402442838	permutation FDR (250 perm	1
2806	Rab38~Q8Q	Rab38	ko vs wt	ko	wt	24,2840686	0,39807302	24,3011367	0,28360613	0,98823899	-0,0170681	-0,0130788	0,95755199	0,01883764	Unpaired t-te	0,97673954	permutation FDR (250 perm	1
2807	Rab39a~Q8B	Rab39a	ko vs wt	ko	wt	26,3278229	1,98856483	24,4297846	0,31099492	3,72706083	1,89803837	0,87508775	0,19675004	0,70608517	Unpaired t-te	0,083897436	permutation FDR (250 perm	1
2808	Rab3gap1~Q	Rab3gap1	ko vs wt	ko	wt	28,5742356	0,46141204	28,3390077	0,33643468	1,17709267	0,23522791	0,17332401	0,53948063	0,26802415	Unpaired t-te	0,688598045	permutation FDR (250 perm	1
2809	Rab3gap2~A	Rab3gap2	ko vs wt	ko	wt	28,4285705	0,99242118	28,2604145	0,83951979	1,12362137	0,16815597	0,09213807	0,8469796	0,07212705	Unpaired t-te	0,839667584	permutation FDR (250 perm	1
2810	Rab43~Q8C	Rab43	ko vs wt	ko	wt	24,2590241	0,87470147	23,4923497	0,79419369	1,70134344	0,7666744	0,43678872	0,36088248	0,4426342	Unpaired t-te	0,322758287	permutation FDR (250 perm	1
2811	Rab4b~Q91Z	Rab4b	ko vs wt	ko	wt	27,0774533	0,90316214	25,878523	0,12196066	2,29569387	1,19893027	0,78437115	0,10351974	0,98497681	Unpaired t-te	0,114743831	permutation FDR (250 perm	1
2812	Rab5a~Q9C	Rab5a	ko vs wt	ko	wt	24,5950356	1,37094076	24,1922225	0,69592166	1,32208326	0,40281303	0,20849407	0,68465863	0,16452592	Unpaired t-te	0,629131765	permutation FDR (250 perm	1
2813	Rab5b~P610	Rab5b	ko vs wt	ko	wt	30,8268997	0,38852275	30,6804168	0,06154007	1,10686775	0,14648285	0,11923759	0,56416215	0,24859606	Unpaired t-te	0,79415625	permutation FDR (250 perm	1

2814	Rab5c~P352	Rab5c	ko vs wt	ko	wt	27,6181367	0,1923763	28,1033198	0,35526851	0,71440644	-0,485183	-0,380634	0,18245363	0,7388475	Unpaired t-te	0,401440684	permutation FDR (250 perm	1
2815	Rab6a~P352	Rab6a	ko vs wt	ko	wt	29,9404919	0,58730616	29,3748815	0,45460046	1,48001356	0,5656104	0,38549452	0,28130347	0,55082491	Unpaired t-te	0,377866131	permutation FDR (250 perm	1
2816	Rab7a~P511	Rab7a	ko vs wt	ko	wt	29,9949844	0,26223285	29,5431205	0,73068444	1,36780623	0,45186387	0,29372373	0,47813311	0,32045118	Unpaired t-te	0,519269704	permutation FDR (250 perm	1
2817	Rab8a~P552	Rab8a	ko vs wt	ko	wt	27,9153812	0,5152581	27,4666418	0,46549612	1,36484717	0,44873941	0,31083308	0,36233226	0,440893	Unpaired t-te	0,471711314	permutation FDR (250 perm	1
2818	Rab8b~P610	Rab8b	ko vs wt	ko	wt	25,4224263	2,11560369	23,5677148	0,66172659	3,61679419	1,85471151	0,80360072	0,2323338	0,6338876	Unpaired t-te	0,100819521	permutation FDR (250 perm	1
2819	Rabgap1~A2	Rabgap1	ko vs wt	ko	wt	26,8885851	0,63380543	27,0316763	0,1711229	0,9055767	-0,1430913	-0,1032841	0,73109567	0,13602579	Unpaired t-te	0,820752902	permutation FDR (250 perm	1
2820	Rabgef1~Q9	Rabgef1	ko vs wt	ko	wt	26,547349	0,58046602	26,0749635	0,42157114	1,3874016	0,47238546	0,3261148	0,34079203	0,46751057	Unpaired t-te	0,449800064	permutation FDR (250 perm	1
2821	Rac1~P6300	Rac1	ko vs wt	ko	wt	31,5340851	0,21289775	31,537081	0,14830424	0,99792554	-0,0029959	-0,0025792	0,98592471	0,00615625	Unpaired t-te	0,995199792	permutation FDR (250 perm	1
2822	Rac2~Q0514	Rac2	ko vs wt	ko	wt	27,9437076	1,01167463	28,137297	0,77716117	0,87442741	-0,1935895	-0,1074323	0,81902327	0,08670376	Unpaired t-te	0,810033296	permutation FDR (250 perm	1
2823	Rack1~P680	Rack1	ko vs wt	ko	wt	30,9244444	0,38873548	30,5927114	0,65063024	1,25852426	0,33173303	0,21941608	0,56379249	0,24888071	Unpaired t-te	0,622120212	permutation FDR (250 perm	1
2824	Rad21~Q615	Rad21	ko vs wt	ko	wt	27,3349217	0,40456647	27,5719962	0,43131417	0,84846411	-0,2370745	-0,1712776	0,56981003	0,24426991	Unpaired t-te	0,69619529	permutation FDR (250 perm	1
2825	Rad23b~P54	Rad23b	ko vs wt	ko	wt	27,3953111	0,59185147	26,9837756	0,57473482	1,33010069	0,41153546	0,26880806	0,47810168	0,32047973	Unpaired t-te	0,534175589	permutation FDR (250 perm	1
2826	Rae1~Q8C57	Rae1	ko vs wt	ko	wt	26,5855932	1,05450427	27,3718827	0,57770337	0,57983349	-0,7862894	-0,4536727	0,33406677	0,47616673	Unpaired t-te	0,303889317	permutation FDR (250 perm	1
2827	Raf1~Q99N5	Raf1	ko vs wt	ko	wt	26,3761843	0,65006936	26,2509359	0,05117587	1,09069555	0,12524846	0,09095364	0,76124376	0,11847625	Unpaired t-te	0,845498353	permutation FDR (250 perm	1
2828	Rai14~Q9EP	Rai14	ko vs wt	ko	wt	27,4946853	0,24179714	27,0912044	0,18097779	1,32269549	0,40348097	0,33923614	0,08708411	1,06006108	Unpaired t-te	0,433258949	permutation FDR (250 perm	1
2829	Ralb~Q9JIW	Ralb	ko vs wt	ko	wt	27,9922304	0,79069014	27,5888599	0,08644157	1,32259421	0,4033705	0,27617163	0,44358477	0,35302338	Unpaired t-te	0,53326232	permutation FDR (250 perm	1
2830	Ralgapa1~Q	Ralgapa1	ko vs wt	ko	wt	23,2849785	1,08816349	26,0184132	0,34671644	0,15036756	-2,7334348	-1,6324928	0,0166663	1,77816092	Unpaired t-te	0,006865406	permutation FDR (250 perm	1
2831	Ralgapb~Q8	Ralgapb	ko vs wt	ko	wt	27,3649207	0,40445139	27,8616226	0,80883676	0,70872511	-0,4967019	-0,3070294	0,48668434	0,31275262	Unpaired t-te	0,495421446	permutation FDR (250 perm	1
2832	Raly~Q6401	Raly	ko vs wt	ko	wt	28,2300267	0,41970645	27,6968598	0,22129473	1,44710227	0,53316689	0,41380481	0,12651474	0,89785886	Unpaired t-te	0,34609874	permutation FDR (250 perm	1
2833	Ranbp10~Q6	Ranbp10	ko vs wt	ko	wt	24,8567225	0,69377259	24,60003	0,78353652	1,19473652	0,25669249	0,15246009	0,72673499	0,13862393	Unpaired t-te	0,730509225	permutation FDR (250 perm	1
2834	Ranbp1~P34	Ranbp1	ko vs wt	ko	wt	25,0012812	0,90650198	23,5011006	0,60533768	2,82878123	1,50018061	0,89503416	0,07724261	1,11214306	Unpaired t-te	0,067314757	permutation FDR (250 perm	1
2835	Ranbp2~Q9E	Ranbp2	ko vs wt	ko	wt	30,4511178	0,25258976	30,9832435	0,42421699	0,69153509	-0,5321256	-0,3990331	0,21049498	0,67675825	Unpaired t-te	0,379581411	permutation FDR (250 perm	1
2836	Rangap1~P4	Rangap1	ko vs wt	ko	wt	27,8303183	0,21229475	27,9634356	0,2694623	0,91185903	-0,1331173	-0,1085292	0,59178861	0,2278334	Unpaired t-te	0,811603993	permutation FDR (250 perm	1
2837	Ran~P62827	Ran	ko vs wt	ko	wt	30,4973584	0,37250933	30,4437883	0,35328533	1,03783001	0,05357016	0,04028935	0,87803718	0,05648709	Unpaired t-te	0,930280543	permutation FDR (250 perm	1
2838	Rap1a~P628	Rap1a	ko vs wt	ko	wt	24,5191346	0,6773191	25,1022577	1,98743113	0,66751719	-0,5831231	-0,2371654	0,72329626	0,14068378	Unpaired t-te	0,600754448	permutation FDR (250 perm	1
2839	Rap1b~Q99J	Rap1b	ko vs wt	ko	wt	30,6592667	0,95025338	30,6688499	0,22120726	0,99337942	-0,0095832	-0,0061021	0,98751719	0,00545534	Unpaired t-te	0,989195103	permutation FDR (250 perm	1
2840	Rap1gds1~E	Rap1gds1	ko vs wt	ko	wt	26,2323876	0,51338977	23,9933838	0,24188007	4,72070983	2,23900381	1,66814416	0,00174856	2,75731929	Unpaired t-te	0,005475	permutation FDR (250 perm	1
2841	Rap2a~Q80Z	Rap2a	ko vs wt	ko	wt	24,8936552	0,87071199	23,7255005	1,0063442	2,24724068	1,16815465	0,62426417	0,25355321	0,59593089	Unpaired t-te	0,180220212	permutation FDR (250 perm	1
2842	Rap2c~Q8BL	Rap2c	ko vs wt	ko	wt	26,4520987	1,64817138	26,8903025	0,05674956	0,73805297	-0,4382037	-0,2244416	0,67664187	0,16964113	Unpaired t-te	0,612975466	permutation FDR (250 perm	1
2843	Rapgef2~Q8	Rapgef2	ko vs wt	ko	wt	24,8113189	0,8088007	25,4500321	0,19325748	0,64228559	-0,6387132	-0,4296625	0,26877117	0,57061731	Unpaired t-te	0,33897092	permutation FDR (250 perm	1
2844	Raph1~F2Z3	Raph1	ko vs wt	ko	wt	24,5298177	1,17986282	24,0962638	1,47945951	1,3505564	0,43355389	0,19283038	0,74759903	0,12633127	Unpaired t-te	0,662097788	permutation FDR (250 perm	1
2845	Rarres2~Q9D	Rarres2	ko vs wt	ko	wt	31,5464655	1,03296241	32,1939211	0,17666387	0,63840525	-0,6474556	-0,4023153	0,36027478	0,44336614	Unpaired t-te	0,371873741	permutation FDR (250 perm	1
2846	Rars1~Q9D0	Rars1	ko vs wt	ko	wt	31,0933384	0,17614344	31,0578702	0,43001341	1,02488941	0,03546824	0,02685724	0,92032581	0,0360584	Unpaired t-te	0,954052146	permutation FDR (250 perm	1
2847	Rars2~Q3U1	Rars2	ko vs wt	ko	wt	24,8405133	0,54872728	23,9931135	0,30831696	1,79925527	0,84739988	0,61202948	0,08014146	1,09614276	Unpaired t-te	0,180416339	permutation FDR (250 perm	1
2848	Rasa1~E9PY	Rasa1	ko vs wt	ko	wt	26,4171385	0,16381641	26,6063797	0,20525244	0,87706694	-0,1892411	-0,1612989	0,34190084	0,46609984	Unpaired t-te	0,716743575	permutation FDR (250 perm	1
2849	Rasa2~P580	Rasa2	ko vs wt	ko	wt	24,2042503	0,46324327	25,0508146	0,43834855	0,55610751	-0,8465643	-0,6006569	0,10050727	0,99780252	Unpaired t-te	0,190346812	permutation FDR (250 perm	1
2850	Rasa3~Q607	Rasa3	ko vs wt	ko	wt	28,9683102	1,94916066	26,4646721	2,11411434	5,67113712	2,50363804	0,87200268	0,25117975	0,60001537	Unpaired t-te	0,07824777	permutation FDR (250 perm	1
2851	Rasa4~Q6PF	Rasa4	ko vs wt	ko	wt	24,7623729	0,93343712	24,6438675	0,35546614	1,08560961	0,11850539	0,07431419	0,85142036	0,06985597	Unpaired t-te	0,873439782	permutation FDR (250 perm	1
2852	Rasgrp2~Q9	Rasgrp2	ko vs wt	ko	wt	26,6800335	2,54191521	24,2510823	0,93504144	5,38501811	2,4289512	0,93076236	0,20428535	0,68976278	Unpaired t-te	0,062788198	permutation FDR (250 perm	1
2853	Rasip1~Q3U	Rasip1	ko vs wt	ko	wt	24,9373031	1,10384195	26,6415482	0,4439505	0,30688178	-1,7042451	-0,9963877	0,07058196	1,15130627	Unpaired t-te	0,048725	permutation FDR (250 perm	1
2854	Rasl12~Q08	Rasl12	ko vs wt	ko	wt	28,5359646	0,40391365	29,0707708	0,74010118	0,6902514	-0,5348062	-0,3400048	0,4238877	0,37274918	Unpaired t-te	0,44997381	permutation FDR (250 perm	1
2855	Raver1~Q9C	Raver1	ko vs wt	ko	wt	26,7812604	0,29269068	26,4409383	0,40168715	1,26603916	0,34032202	0,25578449	0,37104305	0,4305757	Unpaired t-te	0,560210399	permutation FDR (250 perm	1
2856	Rbbp7~Q609	Rbbp7	ko vs wt	ko	wt	26,4725804	2,0400596	27,3620106	0,6796554	0,53982725	-0,8894303	-0,3914568	0,5237071	0,28091154	Unpaired t-te	0,377352075	permutation FDR (250 perm	1
2857	Rbbp9~O888	Rbbp9	ko vs wt	ko	wt	25,8943505	2,25646267	26,6668407	1,67679391	0,58540616	-0,7724902	-0,2797316	0,67950777	0,16780557	Unpaired t-te	0,515295931	permutation FDR (250 perm	1
2858	Rbfox2~Q8B	Rbfox2	ko vs wt	ko	wt	25,6989717	0,45508432	24,1128871	0,99096396	3,00233429	1,58608462	0,90718562	0,13933403	0,85594279	Unpaired t-te	0,083416667	permutation FDR (250 perm	1
2859	Rbm14~Q8C	Rbm14	ko vs wt	ko	wt	30,1025468	0,29385796	29,5958373	0,36184806	1,42080599	0,50670957	0,38768798	0,18050976	0,7434993	Unpaired t-te	0,383738754	permutation FDR (250 perm	1
2860	Rbm25~B2R	Rbm25	ko vs wt	ko	wt	27,2948372	0,23063121	27,070031	0,21771711	1,16862024	0,22480619	0,18678667	0,32549362	0,48745752	Unpaired t-te	0,668173004	permutation FDR (250 perm	1
2861	Rbm26~Q6N	Rbm26	ko vs wt	ko	wt	25,0353831	0,3077726	24,8357568	1,64900066	1,14840086	0,19962631	0,0915935	0,88055296	0,05524452	Unpaired t-te	0,850976132	permutation FDR (250 perm	1
2862	Rbm28~Q8C	Rbm28	ko vs wt	ko	wt	24,9047646	0,88048516	24,2881273	1,02252876	1,53329711	0,61663728	0,32732727	0,52531023	0,27958414	Unpaired t-te	0,454595352	permutation FDR (250 perm	1
2863	Rbm39~Q8V	Rbm39	ko vs wt	ko	wt	29,2546026	0,26856519	29,1070529	0,30095289	1,1076866	0,14754976	0,11679676	0,60550827	0,21787992	Unpaired t-te	0,795829289	permutation FDR (250 perm	1
2864	Rbms2~Q8V	Rbms2	ko vs wt	ko	wt	24,3427443	0,51077521	24,1094825	1,2500592	1,17548965	0,23326184	0,12074722	0,82180918	0,08522901	Unpaired t-te	0,797028731	permutation FDR (250 perm	1
2865	Rbms3~Q8B	Rbms3	ko vs wt	ko	wt	24,5311555	0,71807478	26,0228255	0,30722702	0,35560069	-1,49167	-1,016089	0,0293314	1,5326672	Unpaired t-te	0,044574085	permutation FDR (250 perm	1
2866	Rbmxl1~Q91	Rbmxl1	ko vs wt	ko	wt	28,6895293	0,45607357	28,6420928	0,39624902	1,03342701	0,0474365	0,03426252	0,90694883	0,04241722	Unpaired t-te	0,940445387	permutation FDR (250 perm	1
2867	Rbp1~Q0091	Rbp1	ko vs wt	ko	wt	29,1784253	0,40296213	29,1186638	0,64353388	1,04229343	0,05976148	0,03954908	0,91420301	0,03895735	Unpaired t-te	0,932783417	permutation FDR (250 perm	1
2868	Rbpj~P3126	Rbpj	ko vs wt	ko	wt	25,9969258	1,82540049	27,8073676	0,23330247	0,2851036	-1,8104418	-0,8759938	0,18397468	0,73524194	Unpaired t-te	0,084487427	permutation FDR (250 perm	1
2869	Rbpms2~Q8	Rbpms2	ko vs wt	ko	wt	26,3692422	0,0568694	25,9258248	0,2378858	1,35982163	0,44341742	0,3785411	0,11377863	0,9439393	Unpaired t-te	0,41672751	permutation FDR (250 perm	1
2870	Rbpms~Q9W	Rbpms	ko vs wt	ko	wt	28,6497071	0,29003876	28,7799667	0,25219591	0,91366701	-0,1302596	-0,1046576	0,6187632	0,20847552	Unpaired t-te	0,81508331	permutation FDR (250 perm	1
2871	Rcc1~Q8VE3	Rcc1	ko vs wt	ko	wt	24,3046541	0,4887982	25,0617036	1,26624279	0,59170522	-0,7570495	-0,3904752	0,49211206	0,30793599	Unpaired t-te	0,3971278	permutation FDR (250 perm	1
2872	Rcc2~Q8BK6	Rcc2	ko vs wt	ko	wt	28,4148853	0,90394095	28,7718442	0,82592279	0,78080874	-0,3569589	-0,2001758	0,66952879	0,17423075	Unpaired t-te	0,643987131	permutation FDR (250 perm	1
2873	Rcn1~Q0518	Rcn1	ko vs wt	ko	wt	24,1577616	1,42552745	23,8698894	0,12636705	1,22083837	0,28787221	0,15749096	0,750521	0,12463715	Unpaired t-te	0,726393405	permutation FDR (250 perm	1
2874	Rcn2~Q8BP9	Rcn2	ko vs wt	ko	wt	25,2277308	1,34560363	24,5398376	0,43368231	1,61092927	0,68789315	0,37482903	0,45792105	0,33920939	Unpaired t-te	0,396368074	permutation FDR (250 perm	1
2875	Rcn3~Q8BH	Rcn3	ko vs wt	ko	wt	25,6727774	1,68469281	27,8206411	0,2527473	0,22564649	-2,1478638	-1,0799033	0,11260451	0,94844421	Unpaired t-te	0,041449378	permutation FDR (250 perm	1
2876	Rdh11~Q9Q	Rdh11	ko vs wt	ko	wt	27,6565018	0,33330821	25,4326842	0,86185853	4,67127883	2,22381756	1,35674207	0,05626343	1,24977378	Unpaired t-te	0,021353803	permutation FDR (250 perm	1
2877	Rdh13~Q8CE	Rdh13	ko vs wt	ko	wt	23,9120588	0,56563719	25,2429653	0,44362874	0,39751839	-1,3309065	-0,9160809	0,03333337	1,47712083	Unpaired t-te	0,062389857	permutation FDR (250 perm	1

2878	Rdx~P26043	Rdx	ko vs wt	ko	wt	29,2689199	0,79256257	29,9713398	0,75800173	0,61454053	-0,7024199	-0,4120376	0,37036093	0,43137483	Unpaired t-te	0,351038422	permutation FDR (250 perm	1
2879	Reep5~G3X8	Reep5	ko vs wt	ko	wt	29,2478536	0,43973738	29,3557215	0,10807715	0,92795846	-0,1078679	-0,0852619	0,70759597	0,15021465	Unpaired t-te	0,854516571	permutation FDR (250 perm	1
2880	Reps1~O549	Reps1	ko vs wt	ko	wt	24,2322118	0,43351246	24,0450062	0,56369418	1,13855625	0,18720557	0,12729366	0,7137159	0,14647463	Unpaired t-te	0,777409854	permutation FDR (250 perm	1
2881	Retn~Q99P8	Retn	ko vs wt	ko	wt	27,0390194	0,30630326	27,0264952	0,77975646	1,00871896	0,01252428	0,0079316	0,98440127	0,00682784	Unpaired t-te	0,986561001	permutation FDR (250 perm	1
2882	Rfc2~Q9WU	Rfc2	ko vs wt	ko	wt	24,7945915	0,47284371	24,3872516	0,6738134	1,3262381	0,4073398	0,26294815	0,50759773	0,29448033	Unpaired t-te	0,550614871	permutation FDR (250 perm	1
2883	Rfc3~Q8R32	Rfc3	ko vs wt	ko	wt	25,7251831	1,25362048	26,3822222	0,33254648	0,63417852	-0,6570391	-0,3731019	0,44155569	0,35501451	Unpaired t-te	0,400055227	permutation FDR (250 perm	1
2884	Rfc4~Q99J6	Rfc4	ko vs wt	ko	wt	24,6963383	0,8365012	25,4447394	0,29284738	0,59526292	-0,7484011	-0,4906018	0,22746131	0,64309247	Unpaired t-te	0,273913787	permutation FDR (250 perm	1
2885	Rfc5~Q9D0F	Rfc5	ko vs wt	ko	wt	25,245217	0,76767177	23,4626937	0,13976374	3,44027361	1,78252331	1,22586008	0,02478376	1,6058328	Unpaired t-te	0,024840382	permutation FDR (250 perm	1
2886	Rfk~Q8CFV9	Rfk	ko vs wt	ko	wt	25,255132	0,8926303	27,0826521	0,23972348	0,28174851	-1,8275201	-1,1847613	0,03270714	1,48535747	Unpaired t-te	0,026633557	permutation FDR (250 perm	1
2887	Rfx1~P48377	Rfx1	ko vs wt	ko	wt	25,5177565	1,39638976	25,0899735	0,06408116	1,34516491	0,42778305	0,23667395	0,63275688	0,19876312	Unpaired t-te	0,592159619	permutation FDR (250 perm	1
2888	Rgs7~O5482	Rgs7	ko vs wt	ko	wt	24,1880123	1,24933584	24,4713	0,96091556	0,8217163	-0,2832877	-0,1422885	0,78666296	0,1042113	Unpaired t-te	0,744846089	permutation FDR (250 perm	1
2889	Rheb~Q921J	Rheb	ko vs wt	ko	wt	27,6005081	0,17480116	28,4766232	0,19489237	0,5448326	-0,8761151	-0,7482971	0,00702574	2,1533077	Unpaired t-te	0,121394609	permutation FDR (250 perm	1
2890	Rhoa~Q9QU	Rhoa	ko vs wt	ko	wt	32,0044107	0,66808244	31,8266738	0,33404661	1,13110822	0,17773696	0,12238343	0,71158716	0,1477719	Unpaired t-te	0,782887025	permutation FDR (250 perm	1
2891	Rhob~P6274	Rhob	ko vs wt	ko	wt	28,4282379	0,33687531	27,8742472	0,48364927	1,46814115	0,55399068	0,39757357	0,2465957	0,60801451	Unpaired t-te	0,376741633	permutation FDR (250 perm	1
2892	Rhoc~Q6215	Rhoc	ko vs wt	ko	wt	27,6199483	0,58065681	27,2121806	0,36439421	1,32663159	0,40776778	0,28659072	0,37928385	0,42103565	Unpaired t-te	0,505326714	permutation FDR (250 perm	1
2893	Rhog~P8409	Rhog	ko vs wt	ko	wt	28,5328843	0,40915452	27,7164934	0,6238237	1,76099509	0,81639088	0,54412262	0,19723753	0,70501045	Unpaired t-te	0,240848619	permutation FDR (250 perm	1
2894	Rhot1~Q8BG	Rhot1	ko vs wt	ko	wt	28,0487881	0,10232228	27,7510239	0,24765515	1,22923793	0,29776419	0,25131709	0,22491658	0,64797853	Unpaired t-te	0,577808466	permutation FDR (250 perm	1
2895	Rhot2~Q8JZ	Rhot2	ko vs wt	ko	wt	26,6511349	0,16574815	26,2498448	0,07532066	1,32068839	0,40129011	0,36167984	0,01750465	1,75684665	Unpaired t-te	0,407727124	permutation FDR (250 perm	1
2896	Ric8a~Q3TIR	Ric8a	ko vs wt	ko	wt	24,0557796	1,30487569	24,1801562	0,1773752	0,91740035	-0,1243766	-0,0705187	0,88048258	0,05527923	Unpaired t-te	0,882759033	permutation FDR (250 perm	1
2897	Rigi~Q6Q89	Rigi	ko vs wt	ko	wt	28,5445077	0,49185985	28,5065589	0,11341427	1,02665307	0,03794874	0,02930219	0,90478239	0,04345586	Unpaired t-te	0,949277719	permutation FDR (250 perm	1
2898	Rilpl1~Q9JJC	Rilpl1	ko vs wt	ko	wt	25,1742912	1,5310866	26,845151	0,05079278	0,3140661	-1,6708599	-0,8865377	0,15506464	0,80948724	Unpaired t-te	0,082948494	permutation FDR (250 perm	1
2899	Rimoc1~Q8B	Rimoc1	ko vs wt	ko	wt	24,506809	0,53400872	24,107914	0,30478787	1,31849772	0,39889507	0,28985913	0,33845008	0,47050537	Unpaired t-te	0,500906716	permutation FDR (250 perm	1
2900	Rin3~P5972	Rin3	ko vs wt	ko	wt	24,6304806	0,75352979	24,9593801	0,1667423	0,79614359	-0,3288994	-0,2267108	0,51227185	0,29049951	Unpaired t-te	0,604667062	permutation FDR (250 perm	1
2901	Riok2~Q9CQ	Riok2	ko vs wt	ko	wt	25,0560724	0,44816789	24,5069921	0,26858561	1,46315258	0,54908022	0,41566497	0,14843105	0,82847524	Unpaired t-te	0,342835892	permutation FDR (250 perm	1
2902	Riox2~Q8CD	Riox2	ko vs wt	ko	wt	25,9285602	0,83718865	24,8449748	0,46312692	2,11929648	1,08358543	0,68414993	0,1243734	0,90527249	Unpaired t-te	0,143691405	permutation FDR (250 perm	1
2903	Ripk1~Q608	Ripk1	ko vs wt	ko	wt	26,844166	0,2168773	27,3824987	0,02982439	0,68856621	-0,5383327	-0,4776782	0,02152754	1,66700566	Unpaired t-te	0,294610955	permutation FDR (250 perm	1
2904	Ripk3~Q9QZ	Ripk3	ko vs wt	ko	wt	25,2404828	0,98964094	24,3063644	0,92094974	1,91072266	0,93411839	0,50050935	0,33540773	0,47442693	Unpaired t-te	0,262526998	permutation FDR (250 perm	1
2905	Ripor1~Q68	Ripor1	ko vs wt	ko	wt	26,3368629	0,23267433	26,4683999	0,12254744	0,9128584	-0,131537	-0,1134078	0,44971922	0,34705856	Unpaired t-te	0,800228603	permutation FDR (250 perm	1
2906	Ripor2~Q80	Ripor2	ko vs wt	ko	wt	24,498685	0,95609787	24,7561325	0,36763452	0,8365667	-0,2574475	-0,1598902	0,69391554	0,15869339	Unpaired t-te	0,716662479	permutation FDR (250 perm	1
2907	Rit1~P70426	Rit1	ko vs wt	ko	wt	26,8760777	0,16062826	26,1591993	0,2094219	1,64362185	0,71687841	0,61025149	0,01921625	1,71633141	Unpaired t-te	0,191891389	permutation FDR (250 perm	1
2908	Rmdn1~Q9D	Rmdn1	ko vs wt	ko	wt	26,9146379	0,52173616	26,6167102	0,57073359	1,22937733	0,29792778	0,19814407	0,58579777	0,23225229	Unpaired t-te	0,649632439	permutation FDR (250 perm	1
2909	Rmdn3~Q3U	Rmdn3	ko vs wt	ko	wt	27,343641	0,37324681	27,6357166	0,40937978	0,81672622	-0,2920756	-0,2146227	0,46360526	0,33385165	Unpaired t-te	0,621855038	permutation FDR (250 perm	1
2910	Rmnd5a~Q8	Rmnd5a	ko vs wt	ko	wt	25,1347172	0,74046816	24,1129557	0,7387444	2,03039655	1,02176152	0,61000391	0,19988715	0,69921513	Unpaired t-te	0,185169664	permutation FDR (250 perm	1
2911	Rnase4~Q8C	Rnase4	ko vs wt	ko	wt	25,1652871	0,74787315	25,0076021	1,53732917	1,11549574	0,157685	0,07267694	0,90237765	0,04461167	Unpaired t-te	0,880615385	permutation FDR (250 perm	1
2912	Rnasel~Q05	Rnasel	ko vs wt	ko	wt	25,2109831	0,1291917	24,230169	0,32564828	1,97357884	0,98081415	0,78967536	0,04005409	1,39735309	Unpaired t-te	0,123992979	permutation FDR (250 perm	1
2913	Rnaset2b~Q	Rnaset2b	ko vs wt	ko	wt	24,5758388	1,27906959	23,8325521	0,46327442	1,67428663	0,74328674	0,4111398	0,4088771	0,38840721	Unpaired t-te	0,353759404	permutation FDR (250 perm	1
2914	Rnf213~F7A	Rnf213	ko vs wt	ko	wt	27,6997169	1,88645565	26,681693	0,40327283	2,02514315	1,01802389	0,47887706	0,42530091	0,37130369	Unpaired t-te	0,290479409	permutation FDR (250 perm	1
2915	Rnf2~Q9CQ	Rnf2	ko vs wt	ko	wt	24,6610941	1,06293115	24,4359364	0,53977014	1,16890501	0,2251577	0,13070109	0,76868815	0,11424981	Unpaired t-te	0,766746001	permutation FDR (250 perm	1
2916	Rngtt~O552	Rngtt	ko vs wt	ko	wt	24,8733434	1,06148742	24,6188445	0,24456279	1,19292131	0,25449888	0,15548739	0,71279784	0,14703363	Unpaired t-te	0,727311364	permutation FDR (250 perm	1
2917	Rnh1~Q91V	Rnh1	ko vs wt	ko	wt	28,3357907	1,1737512	28,0627669	0,7741126	1,20833773	0,27302374	0,14591435	0,76661898	0,11542044	Unpaired t-te	0,737999434	permutation FDR (250 perm	1
2918	Rnpep~Q8V	Rnpep	ko vs wt	ko	wt	26,3976571	0,77568551	26,3812343	0,42789529	1,01144841	0,01642274	0,01066085	0,9769702	0,01011868	Unpaired t-te	0,981730408	permutation FDR (250 perm	1
2919	Rnps1~Q99N	Rnps1	ko vs wt	ko	wt	27,1141149	0,46008653	27,0616168	0,29779859	1,0370591	0,05249811	0,03920778	0,88298282	0,05404775	Unpaired t-te	0,931128955	permutation FDR (250 perm	1
2920	Rock1~P703	Rock1	ko vs wt	ko	wt	31,5945026	0,07795054	31,8862892	0,20061024	0,81688985	-0,2917865	-0,2539878	0,16698981	0,77731003	Unpaired t-te	0,574649429	permutation FDR (250 perm	1
2921	Rock2~F8VP	Rock2	ko vs wt	ko	wt	29,7740572	0,79013998	30,1540141	0,65659952	0,76846058	-0,3799568	-0,2301516	0,58592769	0,23215598	Unpaired t-te	0,591145156	permutation FDR (250 perm	1
2922	Rp2~Q9EPK2	Rp2	ko vs wt	ko	wt	27,0373605	0,29271275	26,6591466	0,07373049	1,29973178	0,37821393	0,32137633	0,10795348	0,96676337	Unpaired t-te	0,465150095	permutation FDR (250 perm	1
2923	Rpa1~Q8VE	Rpa1	ko vs wt	ko	wt	24,966945	1,40481783	26,7725821	0,01232968	0,2860547	-1,805637	-0,9969733	0,11238206	0,949303	Unpaired t-te	0,057689805	permutation FDR (250 perm	1
2924	Rpe~Q8VEE	Rpe	ko vs wt	ko	wt	25,0185945	0,74951484	25,1965671	0,73941707	0,88394433	-0,1779726	-0,1060184	0,80516654	0,09411428	Unpaired t-te	0,813541955	permutation FDR (250 perm	1
2925	Rpl10a~Q5X	Rpl10a	ko vs wt	ko	wt	31,6898956	0,27409865	31,3161093	0,20101465	1,29574904	0,37378633	0,30822325	0,13974675	0,85465828	Unpaired t-te	0,473838689	permutation FDR (250 perm	1
2926	Rpl10~Q6ZW	Rpl10	ko vs wt	ko	wt	32,7174119	0,25439146	32,8057243	0,12901725	0,94062242	-0,0883124	-0,075294	0,63253751	0,19891372	Unpaired t-te	0,870747409	permutation FDR (250 perm	1
2927	Rpl11~Q9CX	Rpl11	ko vs wt	ko	wt	31,3732882	0,364907	31,6393652	0,34504536	0,83157774	-0,266077	-0,2012142	0,4511319	0,34569647	Unpaired t-te	0,642612818	permutation FDR (250 perm	1
2928	Rpl12~P3597	Rpl12	ko vs wt	ko	wt	30,196031	0,30276845	30,5033599	0,15179509	0,80813664	-0,3073288	-0,2550178	0,19812313	0,70306482	Unpaired t-te	0,553518496	permutation FDR (250 perm	1
2929	Rpl13a~P192	Rpl13a	ko vs wt	ko	wt	31,2125944	0,27093321	31,3093733	0,41511386	0,93511854	-0,0967788	-0,0726236	0,78930265	0,10275644	Unpaired t-te	0,878616639	permutation FDR (250 perm	1
2930	Rpl13~P479	Rpl13	ko vs wt	ko	wt	31,3071044	0,35056165	30,6411506	0,78382087	1,58661689	0,66595381	0,41882725	0,35432728	0,45059541	Unpaired t-te	0,36469407	permutation FDR (250 perm	1
2931	Rpl14~Q9CR	Rpl14	ko vs wt	ko	wt	32,1405182	0,13246563	32,3070647	0,04799614	0,89097289	-0,1665466	-0,1536874	0,11631349	0,93436991	Unpaired t-te	0,727472877	permutation FDR (250 perm	1
2932	Rpl15~Q9CZ	Rpl15	ko vs wt	ko	wt	31,5691311	0,133285	32,0215985	0,49279571	0,73079193	-0,4524674	-0,3334678	0,32261953	0,49130934	Unpaired t-te	0,469077118	permutation FDR (250 perm	1
2933	Rpl17~Q9CP	Rpl17	ko vs wt	ko	wt	31,8889914	0,38615186	31,9155538	0,20393303	0,98175682	-0,0265624	-0,0209894	0,92437133	0,03415353	Unpaired t-te	0,96318612	permutation FDR (250 perm	1
2934	Rpl18a~P627	Rpl18a	ko vs wt	ko	wt	30,8587704	0,55880784	31,8536892	0,44439834	0,50176408	-0,9949189	-0,6859756	0,07988797	1,09751861	Unpaired t-te	0,142803691	permutation FDR (250 perm	1
2935	Rpl18~P3598	Rpl18	ko vs wt	ko	wt	29,8752438	0,52603089	29,7733681	0,57198485	1,07316784	0,10187573	0,06765618	0,85011486	0,07052239	Unpaired t-te	0,884806723	permutation FDR (250 perm	1
2936	Rpl19~P840	Rpl19	ko vs wt	ko	wt	30,6411768	0,58075905	30,2432203	0,07128343	1,31764016	0,39795643	0,29718893	0,32193066	0,49223766	Unpaired t-te	0,503219876	permutation FDR (250 perm	1
2937	Rpl21~Q9CQ	Rpl21	ko vs wt	ko	wt	31,0101094	0,30278096	31,2209638	0,55775448	0,8640254	-0,2108544	-0,1473066	0,66080981	0,17992352	Unpaired t-te	0,745676363	permutation FDR (250 perm	1
2938	Rpl22~P679	Rpl22	ko vs wt	ko	wt	26,47876	2,02449645	28,1032601	0,3776613	0,32432225	-1,6245001	-0,7387578	0,26055995	0,58409234	Unpaired t-te	0,131035791	permutation FDR (250 perm	1
2939	Rpl23a~P627	Rpl23a	ko vs wt	ko	wt	29,5288084	0,89075991	29,959726	0,69074453	0,74178981	-0,4309176	-0,2521079	0,57059509	0,24367197	Unpaired t-te	0,556755677	permutation FDR (250 perm	1
2940	Rpl23~P628	Rpl23	ko vs wt	ko	wt	31,581944	0,40624199	31,6519677	0,22773874	0,95262231	-0,0700238	-0,0545142	0,81561894	0,0885127	Unpaired t-te	0,905278151	permutation FDR (250 perm	1
2941	Rpl24~Q8BP	Rpl24	ko vs wt	ko	wt	31,2541247	0,30602249	31,1085259	0,19872142	1,10618974	0,14559887	0,11878379	0,54739007	0,26170309	Unpaired t-te	0,789334078	permutation FDR (250 perm	1

2942	Rpl26~P6125	Rpl26	ko vs wt	ko	wt	31,5866993	0,65254931	30,8616008	0,27283657	1,6530135	0,72509851	0,50945846	0,15633494	0,80594394	Unpaired t-te	0,256381436	permutation FDR (250 perm	1
2943	Rpl27a~P141	Rpl27a	ko vs wt	ko	wt	31,4993294	0,22921043	32,1793968	0,09093415	0,62413612	-0,6800674	-0,5928429	0,0086278	2,06409977	Unpaired t-te	0,196569444	permutation FDR (250 perm	1
2944	Rpl27~P6135	Rpl27	ko vs wt	ko	wt	31,1788787	0,52222567	30,7131555	0,19954019	1,38100942	0,46572317	0,34940897	0,23178722	0,6349105	Unpaired t-te	0,425984405	permutation FDR (250 perm	1
2945	Rpl28~P411C	Rpl28	ko vs wt	ko	wt	29,9417978	0,46001362	30,4458116	0,46316814	0,70514222	-0,5040138	-0,3545241	0,2941637	0,53141092	Unpaired t-te	0,417923127	permutation FDR (250 perm	1
2946	Rpl30~P6288	Rpl30	ko vs wt	ko	wt	30,660687	0,40757073	30,8892678	0,27969505	0,85347404	-0,2285808	-0,1748381	0,49057232	0,30929696	Unpaired t-te	0,686036845	permutation FDR (250 perm	1
2947	Rpl31~P629C	Rpl31	ko vs wt	ko	wt	29,6945369	0,29985784	29,4203444	0,47902989	1,20931698	0,27419244	0,19863218	0,52245144	0,28195407	Unpaired t-te	0,656118881	permutation FDR (250 perm	1
2948	Rpl32~P6291	Rpl32	ko vs wt	ko	wt	28,9666916	0,51166534	30,2686254	0,10874809	0,40558219	-1,3019338	-0,997456	0,01835434	1,73626127	Unpaired t-te	0,054146018	permutation FDR (250 perm	1
2949	Rpl34~Q9D1	Rpl34	ko vs wt	ko	wt	30,8540192	0,51502613	31,0366937	0,62739447	0,88106814	-0,1826745	-0,1190784	0,7508419	0,1244515	Unpaired t-te	0,792439978	permutation FDR (250 perm	1
2950	Rpl35a~O55	Rpl35a	ko vs wt	ko	wt	29,8154559	0,32810458	30,1401382	0,14175188	0,7984742	-0,3246823	-0,2673789	0,19800417	0,70332567	Unpaired t-te	0,536332518	permutation FDR (250 perm	1
2951	Rpl35~Q6ZW	Rpl35	ko vs wt	ko	wt	29,7067495	0,49702429	29,3626572	0,26921513	1,26935213	0,34409235	0,25595282	0,36516308	0,43751314	Unpaired t-te	0,551519272	permutation FDR (250 perm	1
2952	Rpl36a~P838	Rpl36a	ko vs wt	ko	wt	28,5192959	0,56935925	28,1638382	0,07889292	1,27939139	0,35545768	0,26657599	0,36072987	0,4428179	Unpaired t-te	0,546254634	permutation FDR (250 perm	1
2953	Rpl36~Q6ZW	Rpl36	ko vs wt	ko	wt	26,8193006	0,13408028	26,7466299	0,10817599	1,05166171	0,0726707	0,0655383	0,53490665	0,27172201	Unpaired t-te	0,886526658	permutation FDR (250 perm	1
2954	Rpl37a~P615	Rpl37a	ko vs wt	ko	wt	28,8793966	0,38567394	28,8649343	0,67382721	1,01007492	0,01446231	0,0094777	0,97985725	0,00883719	Unpaired t-te	0,98451906	permutation FDR (250 perm	1
2955	Rpl38~Q9JJI	Rpl38	ko vs wt	ko	wt	29,6298563	0,35898217	29,9928561	0,2608607	0,77754614	-0,3629998	-0,284159	0,24806739	0,60543032	Unpaired t-te	0,508832456	permutation FDR (250 perm	1
2956	Rpl3~P2765	Rpl3	ko vs wt	ko	wt	31,7407216	0,4118056	32,2413229	0,25507891	0,70681214	-0,5006013	-0,3855435	0,15459203	0,81081289	Unpaired t-te	0,37731593	permutation FDR (250 perm	1
2957	Rpl4~Q9D8E	Rpl4	ko vs wt	ko	wt	31,3258269	0,32666915	31,5248191	0,43913095	0,8711589	-0,1989922	-0,1459633	0,61747994	0,20937715	Unpaired t-te	0,743827119	permutation FDR (250 perm	1
2958	Rpl5~P47962	Rpl5	ko vs wt	ko	wt	30,8623616	0,22553822	31,2323847	0,26312362	0,77377011	-0,3700231	-0,3015436	0,18177218	0,74047259	Unpaired t-te	0,490390503	permutation FDR (250 perm	1
2959	Rpl6~P47911	Rpl6	ko vs wt	ko	wt	31,7657884	0,3632111	32,086152	0,36305537	0,80086799	-0,3203636	-0,2406072	0,3851131	0,41441171	Unpaired t-te	0,577821568	permutation FDR (250 perm	1
2960	Rpl7a~P1297	Rpl7a	ko vs wt	ko	wt	32,2958521	0,21312324	32,2329524	0,12114357	1,0445631	0,06289964	0,05469877	0,69261984	0,15950507	Unpaired t-te	0,904808385	permutation FDR (250 perm	1
2961	Rpl7l1~Q9D8	Rpl7l1	ko vs wt	ko	wt	25,3938612	1,01299282	24,9738509	0,48841492	1,33793712	0,42001031	0,25012377	0,56555309	0,24752662	Unpaired t-te	0,560859474	permutation FDR (250 perm	1
2962	Rpl7~P14148	Rpl7	ko vs wt	ko	wt	30,2282338	0,2373665	31,5529685	0,39316904	0,39922261	-1,3247347	-1,0112828	0,02394774	1,62073538	Unpaired t-te	0,05545863	permutation FDR (250 perm	1
2963	Rpl8~P6291	Rpl8	ko vs wt	ko	wt	32,3159404	0,11063587	32,4527577	0,09690884	0,90952344	-0,1368173	-0,1250982	0,20827479	0,68136329	Unpaired t-te	0,777285195	permutation FDR (250 perm	1
2964	Rpl9~P5141C	Rpl9	ko vs wt	ko	wt	32,1505702	0,19676918	31,9840339	0,07277192	1,12236061	0,16653629	0,14806972	0,25110771	0,60013996	Unpaired t-te	0,737019253	permutation FDR (250 perm	1
2965	Rplp0~P1486	Rplp0	ko vs wt	ko	wt	32,0931246	0,12780256	32,1477982	0,34071897	0,96281223	-0,0546736	-0,0436701	0,84544601	0,07291412	Unpaired t-te	0,927912627	permutation FDR (250 perm	1
2966	Rplp1~P4795	Rplp1	ko vs wt	ko	wt	26,1798993	0,94110904	27,410154	0,05506808	0,42628315	-1,2301161	-0,7963241	0,10821137	0,96572712	Unpaired t-te	0,111560558	permutation FDR (250 perm	1
2967	Rpn1~Q91YC	Rpn1	ko vs wt	ko	wt	32,3780706	0,26577338	32,2308255	0,3413445	1,10745274	0,14724514	0,11449743	0,63700213	0,19585911	Unpaired t-te	0,801977152	permutation FDR (250 perm	1
2968	Rpn2~Q9DB	Rpn2	ko vs wt	ko	wt	30,3964309	0,59784738	30,3876835	0,4922286	1,00608164	0,00874738	0,00586998	0,98648276	0,0059105	Unpaired t-te	0,989230208	permutation FDR (250 perm	1
2969	Rpp30~O887	Rpp30	ko vs wt	ko	wt	24,4635818	0,61422407	25,2361012	0,26324349	0,58539428	-0,7725194	-0,5516	0,11976258	0,92167887	Unpaired t-te	0,223372989	permutation FDR (250 perm	1
2970	Rpp40~Q8R1	Rpp40	ko vs wt	ko	wt	24,134479	0,67412848	24,5315479	0,22960439	0,75939961	-0,3970688	-0,279289	0,40029038	0,39762485	Unpaired t-te	0,521113161	permutation FDR (250 perm	1
2971	Rps10~P633	Rps10	ko vs wt	ko	wt	27,6330409	0,63965157	28,1832629	1,10173831	0,68291502	-0,550222	-0,2954771	0,57012635	0,24402889	Unpaired t-te	0,508364312	permutation FDR (250 perm	1
2972	Rps11~P622	Rps11	ko vs wt	ko	wt	32,1403256	0,10462022	32,2749634	0,36989937	0,91089849	-0,1346378	-0,1061442	0,6614437	0,17950712	Unpaired t-te	0,823215686	permutation FDR (250 perm	1
2973	Rps12~Q6ZV	Rps12	ko vs wt	ko	wt	31,2081269	0,27354651	30,8375087	0,12965574	1,2929067	0,37061817	0,31338896	0,10332996	0,98577372	Unpaired t-te	0,468700601	permutation FDR (250 perm	1
2974	Rps13~P623	Rps13	ko vs wt	ko	wt	29,9215455	0,81474831	29,0995931	0,39777606	1,76779674	0,82195241	0,53095182	0,19840443	0,70244863	Unpaired t-te	0,237574279	permutation FDR (250 perm	1
2975	Rps14~P622	Rps14	ko vs wt	ko	wt	31,1816631	0,27394322	31,3566017	0,49277945	0,88580523	-0,1749386	-0,126523	0,68035971	0,16726141	Unpaired t-te	0,783142058	permutation FDR (250 perm	1
2976	Rps15a~P62	Rps15a	ko vs wt	ko	wt	31,9130912	0,4679369	31,3448365	0,13953691	1,48272871	0,56825465	0,4413227	0,12401732	0,90651767	Unpaired t-te	0,324296654	permutation FDR (250 perm	1
2977	Rps15~P628	Rps15	ko vs wt	ko	wt	27,6343578	0,6987193	25,5083801	1,79188253	4,36504254	2,12599571	0,91255382	0,22968453	0,63886826	Unpaired t-te	0,084833977	permutation FDR (250 perm	1
2978	Rps16~P141	Rps16	ko vs wt	ko	wt	33,224194	0,31543703	33,2118681	0,4395354	1,00858028	0,01232593	0,00906168	0,97464466	0,01115369	Unpaired t-te	0,985368146	permutation FDR (250 perm	1
2979	Rps17~P632	Rps17	ko vs wt	ko	wt	30,4030068	0,51232491	30,1101892	0,10096867	1,22503039	0,29281754	0,22450419	0,40021782	0,39770358	Unpaired t-te	0,609416568	permutation FDR (250 perm	1
2980	Rps18~P622	Rps18	ko vs wt	ko	wt	30,4010606	0,27522189	30,8525334	0,41430739	0,73129596	-0,4514727	-0,3386185	0,26407224	0,57827725	Unpaired t-te	0,446643656	permutation FDR (250 perm	1
2981	Rps19~Q9CZ	Rps19	ko vs wt	ko	wt	29,3749131	0,51893505	29,3399073	0,8651996	1,02456092	0,03500577	0,02082175	0,96228666	0,01669553	Unpaired t-te	0,96451379	permutation FDR (250 perm	1
2982	Rps20~P608	Rps20	ko vs wt	ko	wt	31,7714055	0,45384232	31,5392173	0,47654083	1,17461519	0,2321882	0,16272752	0,61444267	0,21151863	Unpaired t-te	0,711232485	permutation FDR (250 perm	1
2983	Rps21~Q9CC	Rps21	ko vs wt	ko	wt	27,7828447	0,64624402	27,5590045	0,27057253	1,16783805	0,22384022	0,15771134	0,61965995	0,20784658	Unpaired t-te	0,719355942	permutation FDR (250 perm	1
2984	Rps23~P622	Rps23	ko vs wt	ko	wt	31,5416942	0,25851445	31,6489553	0,4275317	0,92834885	-0,1072611	-0,0802163	0,77139334	0,11272411	Unpaired t-te	0,865237991	permutation FDR (250 perm	1
2985	Rps24~P628	Rps24	ko vs wt	ko	wt	29,1939019	0,4435255	30,5863665	0,37848053	0,38091352	-1,3924646	-1,0161016	0,01470639	1,83249395	Unpaired t-te	0,04314554	permutation FDR (250 perm	1
2986	Rps25~P628	Rps25	ko vs wt	ko	wt	30,2269655	0,0839282	30,3787466	0,5557049	0,9001385	-0,1517811	-0,108732	0,73739495	0,13229984	Unpaired t-te	0,820358209	permutation FDR (250 perm	1
2987	Rps26~P628	Rps26	ko vs wt	ko	wt	30,4198353	0,6998208	30,4942481	0,61507877	0,94972857	-0,0744128	-0,0466936	0,90549617	0,04311338	Unpaired t-te	0,919611661	permutation FDR (250 perm	1
2988	Rps27a~P62	Rps27a	ko vs wt	ko	wt	32,0680564	0,49916453	32,6413716	0,48071399	0,67207065	-0,5733152	-0,3965813	0,26186428	0,58192373	Unpaired t-te	0,367846723	permutation FDR (250 perm	1
2989	Rps27l~Q6Z	Rps27l	ko vs wt	ko	wt	30,2371381	0,58426801	30,5710562	0,18606022	0,79337891	-0,3339181	-0,2451538	0,41050869	0,38667765	Unpaired t-te	0,573189645	permutation FDR (250 perm	1
2990	Rps27~Q6ZV	Rps27	ko vs wt	ko	wt	26,5637778	2,18198519	29,2442819	0,36720719	0,1559868	-2,6805041	-1,1724455	0,12146081	0,91556381	Unpaired t-te	0,030129199	permutation FDR (250 perm	1
2991	Rps28~P628	Rps28	ko vs wt	ko	wt	25,2518619	1,76444249	27,3829569	0,20696852	0,22828453	-2,131095	-1,0502351	0,12655985	0,89770405	Unpaired t-te	0,046935169	permutation FDR (250 perm	1
2992	Rps29~P622	Rps29	ko vs wt	ko	wt	29,9403928	0,4093966	29,3129329	1,25482576	1,54484263	0,62745988	0,32710208	0,55710109	0,254066	Unpaired t-te	0,475588031	permutation FDR (250 perm	1
2993	Rps2~P2544	Rps2	ko vs wt	ko	wt	32,2893542	0,18979209	32,7039216	0,24404864	0,75024443	-0,4145674	-0,3442055	0,12126566	0,91626217	Unpaired t-te	0,435916345	permutation FDR (250 perm	1
2994	Rps3a~P973	Rps3a	ko vs wt	ko	wt	33,1468314	0,15295203	33,193595	0,14745884	0,96810569	-0,0467635	-0,041142	0,74792847	0,12613993	Unpaired t-te	0,928534611	permutation FDR (250 perm	1
2995	Rps3~P6290	Rps3	ko vs wt	ko	wt	32,9773696	0,2596734	33,2299399	0,08135222	0,83939961	-0,2525703	-0,2176243	0,19409013	0,71199655	Unpaired t-te	0,617563422	permutation FDR (250 perm	1
2996	Rps4x~P627	Rps4x	ko vs wt	ko	wt	33,2968826	0,30431158	33,3435781	0,13243314	0,96815135	-0,0466955	-0,0389424	0,82505188	0,08351874	Unpaired t-te	0,932180755	permutation FDR (250 perm	1
2997	Rps5~Q91V5	Rps5	ko vs wt	ko	wt	28,6905256	0,68456408	29,3748522	0,46921914	0,62229623	-0,6843266	-0,4513922	0,24218644	0,61585017	Unpaired t-te	0,305465873	permutation FDR (250 perm	1
2998	Rps6ka1~P1	Rps6ka1	ko vs wt	ko	wt	24,7583876	1,5343377	24,5211091	0,18719939	1,17876698	0,23727855	0,12516768	0,80755575	0,09282749	Unpaired t-te	0,783824385	permutation FDR (250 perm	1
2999	Rps6ka3~P1	Rps6ka3	ko vs wt	ko	wt	28,3920592	0,28403534	27,7825824	0,28519525	1,52570582	0,60946762	0,48374116	0,07478596	1,12617992	Unpaired t-te	0,27804476	permutation FDR (250 perm	1
3000	Rps6ka4~Q9	Rps6ka4	ko vs wt	ko	wt	27,1879199	0,3070168	27,0206522	0,20721957	1,12292982	0,16726777	0,13599252	0,49964805	0,30133581	Unpaired t-te	0,755697328	permutation FDR (250 perm	1
3001	Rps6~P6275	Rps6	ko vs wt	ko	wt	31,9351501	0,34822811	31,9278645	0,41221194	1,00506277	0,00728561	0,00538044	0,98462288	0,00673008	Unpaired t-te	0,990330643	permutation FDR (250 perm	1
3002	Rps7~P6208	Rps7	ko vs wt	ko	wt	30,6582067	0,51372034	31,3564609	0,52527855	0,61631757	-0,6982542	-0,4732902	0,212151	0,67335492	Unpaired t-te	0,287863236	permutation FDR (250 perm	1
3003	Rps8~P6224	Rps8	ko vs wt	ko	wt	31,8527472	0,54401823	31,9400525	0,21439215	0,94127927	-0,0873053	-0,06473	0,81405812	0,08934459	Unpaired t-te	0,888569884	permutation FDR (250 perm	1
3004	Rps9~Q6ZW	Rps9	ko vs wt	ko	wt	32,1766435	0,50715821	31,8614712	0,20060209	1,24416022	0,31517229	0,23780183	0,3848291	0,4147321	Unpaired t-te	0,581846154	permutation FDR (250 perm	1
3005	Rpsa~P1420	Rpsa	ko vs wt	ko	wt	30,5466542	0,46202054	29,9540726	0,37247188	1,50794264	0,59258155	0,43101147	0,17688118	0,75231836	Unpaired t-te	0,326800688	permutation FDR (250 perm	1

3006	Rptor~Q8K4(	Rptor	ko vs wt	ko	wt	26,797041	0,13997292	26,6488153	0,24613635	1,10820574	0,14822575	0,12436177	0,49850936	0,30232668	Unpaired t-te	0,787126013	permutation FDR (250 perm	1
3007	Rrad~O8866	Rrad	ko vs wt	ko	wt	28,8138314	0,75989296	29,36361	0,02907984	0,68312496	-0,5497786	-0,3820013	0,29902274	0,52429579	Unpaired t-te	0,396789717	permutation FDR (250 perm	1
3008	Rraga~Q80X	Rraga	ko vs wt	ko	wt	27,6658242	0,64764459	27,0213185	0,3064466	1,5632036	0,64450569	0,45002098	0,2012987	0,69615903	Unpaired t-te	0,309268667	permutation FDR (250 perm	1
3009	Rragc~Q99K	Rragc	ko vs wt	ko	wt	25,3106087	1,13460425	25,5235421	0,57818142	0,86278117	-0,2129334	-0,1201536	0,79440019	0,09996066	Unpaired t-te	0,787431126	permutation FDR (250 perm	1
3010	Rras2~P6207	Rras2	ko vs wt	ko	wt	24,5547024	0,53697388	24,054466	0,1451423	1,41444536	0,50023645	0,37709063	0,20744844	0,68308984	Unpaired t-te	0,395977565	permutation FDR (250 perm	1
3011	Rras~P10833	Rras	ko vs wt	ko	wt	30,4500385	0,44290038	30,5887204	0,43213431	0,90834866	-0,1386819	-0,0991688	0,74396769	0,12844592	Unpaired t-te	0,826128035	permutation FDR (250 perm	1
3012	Rrbp1~Q99P	Rrbp1	ko vs wt	ko	wt	32,3624643	0,21034017	32,893951	0,34902076	0,69184142	-0,5314867	-0,4168342	0,14976815	0,82458053	Unpaired t-te	0,360031123	permutation FDR (250 perm	1
3013	Rsl1d1~Q8B	Rsl1d1	ko vs wt	ko	wt	26,9370521	0,36005035	27,2537455	0,53519201	0,80290803	-0,3166934	-0,2211898	0,51340219	0,28954229	Unpaired t-te	0,616422544	permutation FDR (250 perm	1
3014	Rsu1~Q9D03	Rsu1	ko vs wt	ko	wt	30,3103089	0,30856773	30,8339672	0,34052954	0,69560571	-0,5236583	-0,4029598	0,15548271	0,8083179	Unpaired t-te	0,363958179	permutation FDR (250 perm	1
3015	RtcA~Q9D7H	RtcA	ko vs wt	ko	wt	26,5761595	0,27105504	25,2189757	0,87790186	2,56184599	1,35718375	0,82745407	0,15346969	0,81397739	Unpaired t-te	0,113827648	permutation FDR (250 perm	1
3016	Rtcb~Q99LF	Rtcb	ko vs wt	ko	wt	30,985565	0,13616449	30,8992359	0,29647132	1,06166537	0,08632911	0,07053651	0,72947684	0,13698849	Unpaired t-te	0,88409764	permutation FDR (250 perm	1
3017	Rtn3~Q9ES9	Rtn3	ko vs wt	ko	wt	27,5365964	0,5875092	27,28953	0,41933874	1,18679145	0,24706644	0,17032827	0,60711401	0,21672974	Unpaired t-te	0,694414249	permutation FDR (250 perm	1
3018	Rtn4~Q99P7	Rtn4	ko vs wt	ko	wt	26,733117	0,10428651	27,2172385	1,18107256	0,71493228	-0,4841215	-0,2634944	0,62103885	0,20688123	Unpaired t-te	0,568137474	permutation FDR (250 perm	1
3019	Rufy1~Q8BIJ	Rufy1	ko vs wt	ko	wt	27,2908423	0,33738191	26,9434465	0,30607573	1,272262	0,3473958	0,26905383	0,29121361	0,53578834	Unpaired t-te	0,532860728	permutation FDR (250 perm	1
3020	Rufy2~Q8R4	Rufy2	ko vs wt	ko	wt	24,2946509	0,44604837	24,1343043	1,08529086	1,11755555	0,16034655	0,08861502	0,85820584	0,06640854	Unpaired t-te	0,853179178	permutation FDR (250 perm	1
3021	Runx1~Q033	Runx1	ko vs wt	ko	wt	23,8810517	0,77385786	24,1090947	0,73949569	0,85379228	-0,228043	-0,1351139	0,75536377	0,12184385	Unpaired t-te	0,759438537	permutation FDR (250 perm	1
3022	Ruvbl1~P601	Ruvbl1	ko vs wt	ko	wt	29,1111435	0,34718377	28,5445495	0,50039444	1,481023	0,56659405	0,40279255	0,25084281	0,60059835	Unpaired t-te	0,371478495	permutation FDR (250 perm	1
3023	Ruvbl2~Q9W	Ruvbl2	ko vs wt	ko	wt	28,5210226	0,2789765	28,3194481	0,07790948	1,14995267	0,20157448	0,17225232	0,30737624	0,5123297	Unpaired t-te	0,696483491	permutation FDR (250 perm	1
3024	Ryr2~E9Q40	Ryr2	ko vs wt	ko	wt	24,5426265	1,70642536	24,2926648	0,77934388	1,18917554	0,24996169	0,11741593	0,8344318	0,07860915	Unpaired t-te	0,792783701	permutation FDR (250 perm	1
3025	S100a10~P0	S100a10	ko vs wt	ko	wt	30,9176723	0,54503541	30,4763315	0,45429408	1,35786566	0,44134076	0,30443997	0,37359555	0,4275983	Unpaired t-te	0,480920755	permutation FDR (250 perm	1
3026	S100a11~P5	S100a11	ko vs wt	ko	wt	30,7139206	0,37016453	30,3834694	0,06183434	1,25740659	0,33045122	0,27127493	0,2202508	0,6570825	Unpaired t-te	0,539297281	permutation FDR (250 perm	1
3027	S100a13~P9	S100a13	ko vs wt	ko	wt	24,8724397	0,92530758	25,6160463	0,80533319	0,59724444	-0,7436066	-0,4175643	0,38764434	0,41156656	Unpaired t-te	0,342461433	permutation FDR (250 perm	1
3028	S100a4~P07	S100a4	ko vs wt	ko	wt	31,7091381	0,46880763	31,6495938	0,66987841	1,04213652	0,05954428	0,03852618	0,91941743	0,03648727	Unpaired t-te	0,934146082	permutation FDR (250 perm	1
3029	S100a6~P14	S100a6	ko vs wt	ko	wt	29,680041	0,37032322	30,1111979	0,34704598	0,7416668	-0,4311569	-0,325285	0,24892573	0,60393022	Unpaired t-te	0,452610191	permutation FDR (250 perm	1
3030	S100a8~P27	S100a8	ko vs wt	ko	wt	29,0196479	1,16358077	26,9319575	0,0032661	4,25067044	2,08769041	1,2487698	0,0529781	1,27590364	Unpaired t-te	0,024824573	permutation FDR (250 perm	1
3031	S100a9~P31	S100a9	ko vs wt	ko	wt	28,8638408	2,43326405	28,229093	0,31954028	1,55266637	0,63474787	0,26197857	0,68438728	0,16469807	Unpaired t-te	0,553543966	permutation FDR (250 perm	1
3032	Saa1~P0536	Saa1	ko vs wt	ko	wt	24,8355506	1,16640791	26,3601508	3,48895427	0,34757586	-1,5246002	-0,4285807	0,60449775	0,21860531	Unpaired t-te	0,360010825	permutation FDR (250 perm	1
3033	Saa2~P0536	Saa2	ko vs wt	ko	wt	24,8390836	0,15906189	25,6629641	2,63277726	0,56492043	-0,8238804	-0,2876759	0,70149506	0,15397538	Unpaired t-te	0,534688685	permutation FDR (250 perm	1
3034	Saa4~P3153	Saa4	ko vs wt	ko	wt	30,2451722	0,89993164	29,7568384	0,6669788	1,40282379	0,4883338	0,28696743	0,51781953	0,28582158	Unpaired t-te	0,504979212	permutation FDR (250 perm	1
3035	Sacm1l~Q9E	Sacm1l	ko vs wt	ko	wt	30,1065469	0,49540568	30,022694	0,03195922	1,0598447	0,08385288	0,06515811	0,78891774	0,10296828	Unpaired t-te	0,889813665	permutation FDR (250 perm	1
3036	Sacs~Q9JLC8	Sacs	ko vs wt	ko	wt	28,0280238	0,40902043	28,3754777	0,50846673	0,78596997	-0,3474539	-0,2429481	0,46863364	0,32916654	Unpaired t-te	0,577934554	permutation FDR (250 perm	1
3037	Safb~D3YXK	Safb	ko vs wt	ko	wt	26,4624089	0,3135645	26,4606082	0,3548087	1,00124898	0,00180077	0,00137528	0,99564028	0,00189754	Unpaired t-te	0,998118626	permutation FDR (250 perm	1
3038	Samd4a~Q8	Samd4a	ko vs wt	ko	wt	27,0209091	1,06292846	27,607257	0,85825275	0,6660268	-0,5863479	-0,3147199	0,52814312	0,27724837	Unpaired t-te	0,465320253	permutation FDR (250 perm	1
3039	Samd4b~Q8	Samd4b	ko vs wt	ko	wt	24,6104718	0,63722105	24,6387211	0,29745562	0,98060953	-0,0282493	-0,0198411	0,94967943	0,02242297	Unpaired t-te	0,965292356	permutation FDR (250 perm	1
3040	Samd9l~Q69	Samd9l	ko vs wt	ko	wt	27,2778713	0,44910708	26,9945994	0,15597932	1,21695175	0,28327197	0,22099976	0,37178537	0,4297077	Unpaired t-te	0,610689533	permutation FDR (250 perm	1
3041	Samhd1~Q6	Samhd1	ko vs wt	ko	wt	29,9073893	0,52120172	29,8457841	0,42088512	1,04362634	0,06160526	0,04328557	0,89015803	0,05053289	Unpaired t-te	0,925641847	permutation FDR (250 perm	1
3042	Samm50~Q8	Samm50	ko vs wt	ko	wt	28,8257603	0,21128068	28,9031952	0,07738274	0,94774126	-0,0774349	-0,0683032	0,59292613	0,22699941	Unpaired t-te	0,883964189	permutation FDR (250 perm	1
3043	Sar1a~Q99J	Sar1a	ko vs wt	ko	wt	28,5972794	0,92196276	28,0393009	0,10883203	1,47220495	0,55797853	0,36283509	0,37303855	0,42824629	Unpaired t-te	0,418174593	permutation FDR (250 perm	1
3044	Sardh~Q99L	Sardh	ko vs wt	ko	wt	24,2918778	0,87521172	24,0277695	0,48130987	1,2008935	0,26410822	0,16412084	0,68326725	0,1654094	Unpaired t-te	0,705728027	permutation FDR (250 perm	1
3045	Sars1~P2663	Sars1	ko vs wt	ko	wt	28,2109993	0,76079005	28,0259066	0,17693581	1,13689008	0,18509278	0,12706192	0,70898162	0,14936502	Unpaired t-te	0,777831514	permutation FDR (250 perm	1
3046	Sart1~Q9Z31	Sart1	ko vs wt	ko	wt	27,2573505	0,50792178	24,9972121	1,17125473	4,79037427	2,26013838	1,20310604	0,09865617	1,00587574	Unpaired t-te	0,033506297	permutation FDR (250 perm	1
3047	Sart3~Q9JLI	Sart3	ko vs wt	ko	wt	27,2114544	0,33635614	27,4301537	0,13772778	0,85933982	-0,2186994	-0,1796672	0,36742469	0,43483167	Unpaired t-te	0,680640878	permutation FDR (250 perm	1
3048	Sbds~P7012	Sbds	ko vs wt	ko	wt	28,3624721	0,42745	27,5971883	0,33401401	1,69970436	0,76528383	0,57042834	0,07654106	1,11610555	Unpaired t-te	0,207785061	permutation FDR (250 perm	1
3049	Sbf1~Q6ZPE	Sbf1	ko vs wt	ko	wt	27,5675473	0,73223299	26,9833916	0,80607594	1,49916134	0,58415566	0,34168174	0,45667187	0,34039574	Unpaired t-te	0,435587894	permutation FDR (250 perm	1
3050	Sbf2~E9Q0D	Sbf2	ko vs wt	ko	wt	25,9084499	0,02347658	25,9083326	0,36173614	1,00008134	0,00011734	9,34E-05	0,99967586	0,00014079	Unpaired t-te	0,999785918	permutation FDR (250 perm	1
3051	Sbspon~Q3U	Sbspon	ko vs wt	ko	wt	27,2871592	0,95040579	27,8651672	0,437561	0,66988809	-0,578008	-0,3546199	0,40510247	0,39243511	Unpaired t-te	0,416033898	permutation FDR (250 perm	1
3052	Scaf4~Q6PF	Scaf4	ko vs wt	ko	wt	26,1366608	1,24637788	24,4534205	1,31202039	3,21148441	1,68324029	0,77422286	0,22277136	0,65214065	Unpaired t-te	0,109281448	permutation FDR (250 perm	1
3053	Scara3~Q8C	Scara3	ko vs wt	ko	wt	26,6039712	0,50745578	26,5422192	0,17337033	1,04373251	0,06175202	0,04686727	0,85539955	0,06783098	Unpaired t-te	0,920313453	permutation FDR (250 perm	1
3054	Scarb2~O351	Scarb2	ko vs wt	ko	wt	25,6177501	1,4741666	24,44593	0,68967009	2,25295752	1,17182011	0,59155234	0,29069362	0,5365645	Unpaired t-te	0,195325599	permutation FDR (250 perm	1
3055	Sccpdh~Q8R	Sccpdh	ko vs wt	ko	wt	25,5737385	1,85370386	24,9277397	1,85694118	1,56482226	0,64599879	0,2397947	0,72112152	0,14199154	Unpaired t-te	0,578658668	permutation FDR (250 perm	1
3056	Scfd1~Q8BR	Scfd1	ko vs wt	ko	wt	27,2292694	0,50579681	27,162488	0,21942121	1,04737737	0,06678134	0,05018585	0,84894153	0,07112222	Unpaired t-te	0,913602358	permutation FDR (250 perm	1
3057	Scfd2~Q8BT	Scfd2	ko vs wt	ko	wt	24,6930315	0,9605056	26,6145349	0,04677438	0,26397927	-1,9215034	-1,2352696	0,04023053	1,39544423	Unpaired t-te	0,025691057	permutation FDR (250 perm	1
3058	Scin~Q60604	Scin	ko vs wt	ko	wt	25,8414708	0,90853452	25,1026933	1,37528575	1,66876112	0,73877745	0,35097633	0,54941349	0,26010068	Unpaired t-te	0,431932882	permutation FDR (250 perm	1
3059	Scn7a~B1AY	Scn7a	ko vs wt	ko	wt	24,7601472	1,10911086	26,4940883	0,15283251	0,30062957	-1,7339412	-1,0512553	0,07137903	1,14642937	Unpaired t-te	0,046625072	permutation FDR (250 perm	1
3060	Scp2~P3202	Scp2	ko vs wt	ko	wt	28,2492434	0,93864619	28,4749037	1,08128633	0,85520352	-0,2256603	-0,1164901	0,82195068	0,08515424	Unpaired t-te	0,796980753	permutation FDR (250 perm	1
3061	Scpep1~Q92	Scpep1	ko vs wt	ko	wt	23,7897185	0,16264033	23,4141471	0,33587354	1,29735329	0,3755714	0,29916769	0,24982406	0,60236574	Unpaired t-te	0,506905485	permutation FDR (250 perm	1
3062	Scrib~Q80U7	Scrib	ko vs wt	ko	wt	26,2111387	0,11743815	26,6545673	0,39790415	0,73538486	-0,4434286	-0,343899	0,25239573	0,59791799	Unpaired t-te	0,4550156	permutation FDR (250 perm	1
3063	Scube3~Q66	Scube3	ko vs wt	ko	wt	29,7542476	1,07164951	30,7176054	0,15613743	0,51286186	-0,9633578	-0,5915649	0,21755366	0,66243361	Unpaired t-te	0,207653831	permutation FDR (250 perm	1
3064	Scyl1~Q9EQ	Scyl1	ko vs wt	ko	wt	28,6000989	0,36860559	28,6865798	0,17605375	0,94181725	-0,0864809	-0,0693762	0,74126191	0,13002831	Unpaired t-te	0,881598913	permutation FDR (250 perm	1
3065	Scyl2~Q8CFE	Scyl2	ko vs wt	ko	wt	26,2190432	0,34971038	24,8170372	1,04612659	2,64268788	1,40200604	0,79353606	0,19218761	0,7162746	Unpaired t-te	0,125045912	permutation FDR (250 perm	1
3066	Sdcbp~O089	Sdcbp	ko vs wt	ko	wt	30,8078995	0,38092818	30,5613054	0,58400495	1,186403	0,24659415	0,16799487	0,63325931	0,19841842	Unpaired t-te	0,707390956	permutation FDR (250 perm	1
3067	Sdha~Q8K2B	Sdha	ko vs wt	ko	wt	30,7328586	0,0611532	30,3783376	0,17450156	1,27856101	0,354521	0,31419608	0,0928727	1,03211192	Unpaired t-te	0,491589263	permutation FDR (250 perm	1
3068	Sdhb~Q9CQA	Sdhb	ko vs wt	ko	wt	30,2302696	0,51071914	30,3505868	0,60807098	0,91998536	-0,1203172	-0,0790851	0,82947408	0,08119718	Unpaired t-te	0,864735808	permutation FDR (250 perm	1
3069	Sec11a~Q9R	Sec11a	ko vs wt	ko	wt	24,5613422	0,80898636	26,6262498	0,14253851	0,23900164	-2,0649076	-1,3972663	0,01905517	1,7199871	Unpaired t-te	0,0144256	permutation FDR (250 perm	1

3070	Sec13~Q9D1	Sec13	ko vs wt	ko	wt	27,6904223	0,22303908	26,9773022	0,18750515	1,63934571	0,71312012	0,60187671	0,01317696	1,88018467	Unpaired t-te	0,187667707	permutation FDR (250 perm	1
3071	Sec16a~A2AI	Sec16a	ko vs wt	ko	wt	28,1138977	0,71485992	27,8125019	0,36563237	1,23233608	0,30139576	0,20268488	0,56461437	0,24824808	Unpaired t-te	0,638713909	permutation FDR (250 perm	1
3072	Sec22a~Q8B	Sec22a	ko vs wt	ko	wt	23,6329351	0,98118901	23,6680432	1,183573	0,97595865	-0,0351081	-0,0174614	0,9740659	0,01141166	Unpaired t-te	0,970018358	permutation FDR (250 perm	1
3073	Sec22b~O08	Sec22b	ko vs wt	ko	wt	28,641501	0,4899249	28,7478821	0,47699076	0,92891525	-0,1063811	-0,0738659	0,81997382	0,08620001	Unpaired t-te	0,874041417	permutation FDR (250 perm	1
3074	Sec23a~Q01	Sec23a	ko vs wt	ko	wt	30,8019431	0,18958307	30,8621014	0,08716045	0,95915889	-0,0601583	-0,0534448	0,6542526	0,18425454	Unpaired t-te	0,907902229	permutation FDR (250 perm	1
3075	Sec23b~Q9D	Sec23b	ko vs wt	ko	wt	27,2328555	0,29874284	26,605474	0,84845881	1,54475874	0,62738154	0,3862588	0,40749815	0,38987436	Unpaired t-te	0,403607096	permutation FDR (250 perm	1
3076	Sec23ip~G3X	Sec23ip	ko vs wt	ko	wt	29,79411	0,46280969	29,3664449	0,27616087	1,34505488	0,42766504	0,32132237	0,25337299	0,59623969	Unpaired t-te	0,455965627	permutation FDR (250 perm	1
3077	Sec24a~Q3U	Sec24a	ko vs wt	ko	wt	26,3234304	0,40935932	26,144834	0,33016263	1,13178221	0,17859637	0,13406044	0,6147153	0,21132598	Unpaired t-te	0,760380309	permutation FDR (250 perm	1
3078	Sec24b~Q80	Sec24b	ko vs wt	ko	wt	28,450597	0,82217889	27,8668501	0,58609946	1,49873671	0,58374696	0,35809518	0,39694496	0,4012697	Unpaired t-te	0,409143418	permutation FDR (250 perm	1
3079	Sec24c~G3X	Sec24c	ko vs wt	ko	wt	30,1662109	0,41416384	29,8603911	0,15015126	1,23612078	0,30581972	0,2424006	0,30642171	0,51368046	Unpaired t-te	0,576048077	permutation FDR (250 perm	1
3080	Sec24d~Q6N	Sec24d	ko vs wt	ko	wt	31,1679702	0,34647267	31,0359469	0,14851127	1,09582945	0,13202328	0,10769276	0,58774616	0,2308102	Unpaired t-te	0,810900111	permutation FDR (250 perm	1
3081	Sec31a~Q3U	Sec31a	ko vs wt	ko	wt	31,7909121	0,29677318	31,3311611	0,32148089	1,37530438	0,45975095	0,35787666	0,18076975	0,74287423	Unpaired t-te	0,415080535	permutation FDR (250 perm	1
3082	Sec61a1~P61	Sec61a1	ko vs wt	ko	wt	24,2037182	1,20215882	24,3360614	0,75983303	0,91234842	-0,1323432	-0,0704806	0,88605881	0,05253745	Unpaired t-te	0,878769984	permutation FDR (250 perm	1
3083	Sec62~Q8BU	Sec62	ko vs wt	ko	wt	24,5321358	0,64921816	25,0710917	2,16421817	0,68826881	-0,538956	-0,2092571	0,76174535	0,11819019	Unpaired t-te	0,647408229	permutation FDR (250 perm	1
3084	Sec63~Q8VH	Sec63	ko vs wt	ko	wt	28,1185065	0,52332234	27,2954515	0,57573793	1,76914835	0,82305503	0,54616291	0,17979995	0,74521044	Unpaired t-te	0,230590164	permutation FDR (250 perm	1
3085	Seh1l~Q8R2	Seh1l	ko vs wt	ko	wt	26,989536	0,34394918	26,8829249	0,10183504	1,07669608	0,10661107	0,08801872	0,64201017	0,19245809	Unpaired t-te	0,848853772	permutation FDR (250 perm	1
3086	Sel1l~Q9Z2G	Sel1l	ko vs wt	ko	wt	25,0309018	0,99328812	27,0923851	0,29207605	0,23956959	-2,0614833	-1,2807975	0,0308298	1,51102934	Unpaired t-te	0,018554745	permutation FDR (250 perm	1
3087	Selenbp1~P1	Selenbp1	ko vs wt	ko	wt	25,2122742	1,5273378	26,1767437	0,89960102	0,51246683	-0,9644695	-0,4620649	0,41621986	0,3806772	Unpaired t-te	0,294305585	permutation FDR (250 perm	1
3088	Selenof~Q9E	Selenof	ko vs wt	ko	wt	26,7950092	0,8253784	27,1182498	0,80121892	0,79927251	-0,3232406	-0,1857374	0,68308802	0,16552333	Unpaired t-te	0,670382361	permutation FDR (250 perm	1
3089	Selenoh~Q3	Selenoh	ko vs wt	ko	wt	24,687618	0,51482702	25,1087296	0,46785918	0,74684897	-0,4211116	-0,2914788	0,39119239	0,4076096	Unpaired t-te	0,500141791	permutation FDR (250 perm	1
3090	Selenos~Q9E	Selenos	ko vs wt	ko	wt	24,8063746	1,24646918	25,3657972	1,81575787	0,67857372	-0,5594225	-0,2263161	0,72744456	0,1382001	Unpaired t-te	0,606999704	permutation FDR (250 perm	1
3091	Sema3b~Q6	Sema3b	ko vs wt	ko	wt	28,2533189	1,20692641	28,8958513	0,9674769	0,6405875	-0,6425324	-0,3250851	0,54057945	0,26714047	Unpaired t-te	0,45135311	permutation FDR (250 perm	1
3092	Sema3c~Q6	Sema3c	ko vs wt	ko	wt	28,2932186	0,47927615	28,7685245	0,2848446	0,71931423	-0,475306	-0,3541107	0,22425417	0,64925947	Unpaired t-te	0,414299511	permutation FDR (250 perm	1
3093	Sema3f~O88	Sema3f	ko vs wt	ko	wt	24,8486161	1,25131032	26,1785878	0,18780273	0,39777605	-1,3299717	-0,766754	0,16209606	0,79022754	Unpaired t-te	0,121223009	permutation FDR (250 perm	1
3094	Semg1~Q8B	Semg1	ko vs wt	ko	wt	28,9804819	2,88979128	26,8890961	1,68971206	4,26157255	2,09138589	0,68522411	0,35557481	0,44906901	Unpaired t-te	0,142765101	permutation FDR (250 perm	1
3095	Sephs1~Q8B	Sephs1	ko vs wt	ko	wt	25,96439	1,13524839	23,870028	0,6143961	4,27037276	2,09436201	1,17242899	0,04638785	1,33359578	Unpaired t-te	0,023281981	permutation FDR (250 perm	1
3096	Sephs2~P97	Sephs2	ko vs wt	ko	wt	24,0866289	0,95579412	24,3425175	0,74849425	0,83747111	-0,2558887	-0,1450111	0,75180197	0,12389654	Unpaired t-te	0,739668646	permutation FDR (250 perm	1
3097	Septin10~Q8	Septin10	ko vs wt	ko	wt	26,650194	0,34792444	26,6823087	0,1188015	0,97798576	-0,0321146	-0,0263725	0,88994607	0,05063631	Unpaired t-te	0,953221286	permutation FDR (250 perm	1
3098	Septin11~Q8	Septin11	ko vs wt	ko	wt	30,2733736	0,33134818	29,650048	0,36089105	1,54042195	0,62332559	0,4725983	0,1217932	0,91437694	Unpaired t-te	0,290292958	permutation FDR (250 perm	1
3099	Septin2~P42	Septin2	ko vs wt	ko	wt	31,8683424	0,2810684	31,9580665	0,12862665	0,93970247	-0,089724	-0,075651	0,65205169	0,18571797	Unpaired t-te	0,870379705	permutation FDR (250 perm	1
3100	Septin4~P28	Septin4	ko vs wt	ko	wt	25,548686	0,36407294	24,1976853	0,53282205	2,55088992	1,35100064	0,94381122	0,04704883	1,32745113	Unpaired t-te	0,066678405	permutation FDR (250 perm	1
3101	Septin5~Q9Z	Septin5	ko vs wt	ko	wt	28,8000025	0,49039932	29,1964122	0,14071979	0,75974661	-0,3964098	-0,3049052	0,26226245	0,58126389	Unpaired t-te	0,486941103	permutation FDR (250 perm	1
3102	Septin6~Q9R	Septin6	ko vs wt	ko	wt	29,4831315	0,54968703	29,7915066	0,10158973	0,8075508	-0,3083751	-0,2326674	0,40733854	0,3900445	Unpaired t-te	0,596161808	permutation FDR (250 perm	1
3103	Septin7~E9Q	Septin7	ko vs wt	ko	wt	32,05328	0,22202703	31,8511356	0,30502429	1,15040706	0,20214443	0,16159891	0,47324359	0,32491526	Unpaired t-te	0,717313552	permutation FDR (250 perm	1
3104	Septin8~Q8C	Septin8	ko vs wt	ko	wt	31,3488888	0,35280326	30,9335316	0,35680679	1,3336288	0,41535716	0,31365189	0,26561678	0,5757445	Unpaired t-te	0,470133923	permutation FDR (250 perm	1
3105	Septin9~Q80	Septin9	ko vs wt	ko	wt	30,8940219	0,31278324	31,3049072	0,27328335	0,75216165	-0,4108854	-0,3249425	0,18530445	0,73211414	Unpaired t-te	0,452079006	permutation FDR (250 perm	1
3106	Serbp1~Q9C	Serbp1	ko vs wt	ko	wt	27,2183024	0,81247225	26,8189474	0,44557432	1,31891808	0,39935496	0,2551673	0,51229112	0,29048318	Unpaired t-te	0,552535032	permutation FDR (250 perm	1
3107	Serinc3~Q9C	Serinc3	ko vs wt	ko	wt	25,3344064	0,63855295	24,4723159	0,65213868	1,81767025	0,8620905	0,5420627	0,21436419	0,66884776	Unpaired t-te	0,231955357	permutation FDR (250 perm	1
3108	Serpina10~Q	Serpina10	ko vs wt	ko	wt	26,2283117	1,70992953	24,5863682	0,21986599	3,12085991	1,6419436	0,82122025	0,19466536	0,71071133	Unpaired t-te	0,101384261	permutation FDR (250 perm	1
3109	Serpina1a~P	Serpina1a	ko vs wt	ko	wt	30,1343124	0,28078309	29,9442414	0,34789214	1,14081733	0,19007096	0,14681733	0,55707117	0,25408932	Unpaired t-te	0,740911249	permutation FDR (250 perm	1
3110	Serpina1b~P	Serpina1b	ko vs wt	ko	wt	29,3888949	0,57544553	29,3625008	0,72881379	1,01846344	0,02639419	0,0163618	0,96794807	0,01414794	Unpaired t-te	0,971423782	permutation FDR (250 perm	1
3111	Serpina1c~Q	Serpina1c	ko vs wt	ko	wt	24,3059883	0,29058377	23,9857566	0,18944054	1,24853112	0,32023178	0,26363334	0,1960268	0,70768455	Unpaired t-te	0,539361026	permutation FDR (250 perm	1
3112	Serpina1d~Q	Serpina1d	ko vs wt	ko	wt	32,1157392	0,3598084	32,2403887	0,07850583	0,91722685	-0,1246495	-0,1025901	0,59815167	0,22318868	Unpaired t-te	0,822446838	permutation FDR (250 perm	1
3113	Serpina1e~Q	Serpina1e	ko vs wt	ko	wt	28,6413222	0,83348142	28,5547127	0,24160967	1,06187177	0,08660956	0,0573331	0,87414604	0,058416	Unpaired t-te	0,901791768	permutation FDR (250 perm	1
3114	Serpina3k~P	Serpina3k	ko vs wt	ko	wt	31,1925671	0,1437698	31,0257853	0,09520364	1,12255161	0,16678177	0,15067836	0,17937934	0,74622757	Unpaired t-te	0,729838206	permutation FDR (250 perm	1
3115	Serpina3m~	Serpina3m	ko vs wt	ko	wt	28,1103275	0,75128801	29,6189241	0,52830623	0,35145292	-1,5085966	-0,9593928	0,04636199	1,33383792	Unpaired t-te	0,053001115	permutation FDR (250 perm	1
3116	Serpina3n~G	Serpina3n	ko vs wt	ko	wt	31,1281058	0,19526318	31,7760538	0,53489793	0,6381874	-0,647948	-0,4645876	0,22318552	0,65133399	Unpaired t-te	0,321855613	permutation FDR (250 perm	1
3117	Serpinb1a~Q	Serpinb1a	ko vs wt	ko	wt	27,1803576	1,05641309	24,9550708	1,65364835	4,67603871	2,22528687	0,95966456	0,18763003	0,72669766	Unpaired t-te	0,064280628	permutation FDR (250 perm	1
3118	Serpinb2~P1	Serpinb2	ko vs wt	ko	wt	24,685355	0,91518421	23,6557547	0,12259058	2,04145856	1,02960028	0,67055487	0,14553472	0,8370334	Unpaired t-te	0,162907175	permutation FDR (250 perm	1
3119	Serpinb6b~O	Serpinb6b	ko vs wt	ko	wt	27,7852744	0,55711455	26,5822781	0,87621621	2,302173	1,20299625	0,70843882	0,18109478	0,74209406	Unpaired t-te	0,14646	permutation FDR (250 perm	1
3120	Serpinb6~Q6	Serpinb6	ko vs wt	ko	wt	30,852545	0,2633298	30,9253567	0,15185417	0,95078322	-0,0728116	-0,0613859	0,71206291	0,14748163	Unpaired t-te	0,89337473	permutation FDR (250 perm	1
3121	Serpinb8~O0	Serpinb8	ko vs wt	ko	wt	25,1033713	0,5135395	23,6094734	0,86705179	2,81648927	1,49389798	0,88868202	0,1177602	0,92900148	Unpaired t-te	0,083105723	permutation FDR (250 perm	1
3122	Serpinb9~O0	Serpinb9	ko vs wt	ko	wt	26,5986781	0,58969915	27,4607356	0,03892587	0,55016738	-0,8620575	-0,6425718	0,08481152	1,07154516	Unpaired t-te	0,178285264	permutation FDR (250 perm	1
3123	Serpinc1~P3	Serpinc1	ko vs wt	ko	wt	31,801112	0,4737158	30,8328391	0,09345788	1,95649699	0,96827289	0,75565407	0,03482872	1,45806244	Unpaired t-te	0,123933304	permutation FDR (250 perm	1
3124	Serpind1~P4	Serpind1	ko vs wt	ko	wt	28,4663267	0,5231693	28,0420493	0,37360327	1,34190026	0,42427745	0,30277865	0,33896574	0,46984419	Unpaired t-te	0,481967955	permutation FDR (250 perm	1
3125	Serpine1~G5	Serpine1	ko vs wt	ko	wt	28,1195378	0,93453745	27,9546846	0,85563944	1,12105198	0,16485318	0,0910456	0,84766389	0,07177632	Unpaired t-te	0,84100989	permutation FDR (250 perm	1
3126	Serpine2~Q0	Serpine2	ko vs wt	ko	wt	32,5289199	0,21437998	32,5269974	0,29608127	1,00133348	0,00192253	0,00154642	0,99413835	0,00255317	Unpaired t-te	0,997784599	permutation FDR (250 perm	1
3127	Serpinf1~P9	Serpinf1	ko vs wt	ko	wt	30,2439172	0,19839914	29,990498	0,85002909	1,19202893	0,25341925	0,15721981	0,71650916	0,14477825	Unpaired t-te	0,735721246	permutation FDR (250 perm	1
3128	Serpinf2~Q6	Serpinf2	ko vs wt	ko	wt	30,1757597	1,0773159	27,2534005	0,87647447	7,58084756	2,92235916	1,55605951	0,02209353	1,65573492	Unpaired t-te	0,006914661	permutation FDR (250 perm	1
3129	Serping1~P9	Serping1	ko vs wt	ko	wt	29,8490233	1,16382338	28,7541502	0,30847245	2,13594281	1,09487302	0,64160835	0,20373711	0,69092986	Unpaired t-te	0,173258964	permutation FDR (250 perm	1
3130	Serpinh1~P1	Serpinh1	ko vs wt	ko	wt	35,0318239	0,51190178	34,3927161	0,34674513	1,55736572	0,63910778	0,46177964	0,15695055	0,80423716	Unpaired t-te	0,294214261	permutation FDR (250 perm	1
3131	Sestd1~Q80	Sestd1	ko vs wt	ko	wt	25,8229073	1,24611284	25,5870893	1,03602706	1,17757429	0,23581808	0,11635116	0,82788278	0,08203115	Unpaired t-te	0,794331008	permutation FDR (250 perm	1
3132	Setd3~Q91W	Setd3	ko vs wt	ko	wt	27,1133723	0,44167985	27,4012956	0,24716869	0,81908026	-0,2879233	-0,2199316	0,39551426	0,40283785	Unpaired t-te	0,607743195	permutation FDR (250 perm	1
3133	Setd7~Q8VH	Setd7	ko vs wt	ko	wt	26,5283874	0,39358136	26,2605707	0,17202275	1,20398433	0,26781661	0,21293433	0,35221804	0,45318841	Unpaired t-te	0,622645066	permutation FDR (250 perm	1

3134	Sf1~F6SRV1	Sf1	ko vs wt	ko	wt	23,9508024	0,42847553	23,8242576	0,30152985	1,09167604	0,12654479	0,09539146	0,71439037	0,14606441	Unpaired t-te	0,832477135	permutation FDR (250 perm	1
3135	Sf1~Q64213	Sf1	ko vs wt	ko	wt	26,4760947	0,83819099	26,6241267	0,9354322	0,90248069	-0,148032	-0,0813552	0,86553612	0,0627148	Unpaired t-te	0,860755811	permutation FDR (250 perm	1
3136	Sf3a1~Q8K4	Sf3a1	ko vs wt	ko	wt	28,6405547	0,80851147	27,9682269	0,35590236	1,59364221	0,67232776	0,43934049	0,26734265	0,57293176	Unpaired t-te	0,320987211	permutation FDR (250 perm	1
3137	Sf3a2~G3UV	Sf3a2	ko vs wt	ko	wt	27,0104771	0,26764438	26,9723069	0,05370907	1,02681065	0,03817016	0,03293021	0,82434742	0,08388972	Unpaired t-te	0,943833686	permutation FDR (250 perm	1
3138	Sf3a3~Q9D5	Sf3a3	ko vs wt	ko	wt	27,4417073	0,53654132	26,5347516	0,20108457	1,87508464	0,90695572	0,67640531	0,05459648	1,26283534	Unpaired t-te	0,151271074	permutation FDR (250 perm	1
3139	Sf3b1~Q99N	Sf3b1	ko vs wt	ko	wt	28,9581941	0,46868812	28,056825	1,17859214	1,86783769	0,9013691	0,48041743	0,39509181	0,40330197	Unpaired t-te	0,305577327	permutation FDR (250 perm	1
3140	Sf3b2~Q3UJ	Sf3b2	ko vs wt	ko	wt	28,9698468	0,32794104	28,4177962	0,31069522	1,46616821	0,55205063	0,4279381	0,12261265	0,91146473	Unpaired t-te	0,332455887	permutation FDR (250 perm	1
3141	Sf3b3~Q921	Sf3b3	ko vs wt	ko	wt	29,1980547	0,91052542	28,9321172	0,80854096	1,20241709	0,26593742	0,14968279	0,7470888	0,12662777	Unpaired t-te	0,733309875	permutation FDR (250 perm	1
3142	Sf3b6~P597	Sf3b6	ko vs wt	ko	wt	25,2743965	1,49832453	26,7958077	0,47080327	0,34834503	-1,5214111	-0,789562	0,17924093	0,7465628	Unpaired t-te	0,106038251	permutation FDR (250 perm	1
3143	Sfpq~Q8VIJ6	Sfpq	ko vs wt	ko	wt	32,0140216	0,48112009	31,8482434	0,12400474	1,12177102	0,16577822	0,12838228	0,60310446	0,21960746	Unpaired t-te	0,775602129	permutation FDR (250 perm	1
3144	Sfrp1~Q8C4	Sfrp1	ko vs wt	ko	wt	27,4243743	0,24067124	27,0531226	0,35464591	1,29347452	0,37125163	0,28853104	0,28050661	0,55205691	Unpaired t-te	0,514309685	permutation FDR (250 perm	1
3145	Sfrp2~P9729	Sfrp2	ko vs wt	ko	wt	27,646677	0,67300232	27,8248602	1,55153178	0,88381529	-0,1781832	-0,0823445	0,88979012	0,05071242	Unpaired t-te	0,863948648	permutation FDR (250 perm	1
3146	Sfxn1~Q99JR	Sfxn1	ko vs wt	ko	wt	29,0554361	0,61559539	29,0023678	0,86802357	1,03746905	0,05306829	0,0310475	0,94464388	0,02473189	Unpaired t-te	0,946908225	permutation FDR (250 perm	1
3147	Sfxn3~Q91V	Sfxn3	ko vs wt	ko	wt	30,6715633	0,29704331	31,1355751	0,098972	0,72496748	-0,4640118	-0,3914959	0,0676827	1,16952232	Unpaired t-te	0,377331995	permutation FDR (250 perm	1
3148	Sgca~P8235	Sgca	ko vs wt	ko	wt	29,4314089	1,04829122	28,7394462	0,56243666	1,61547972	0,69196264	0,40132281	0,38488704	0,41466671	Unpaired t-te	0,360631935	permutation FDR (250 perm	1
3149	Sgcb~P8234	Sgcb	ko vs wt	ko	wt	30,4547245	0,60875047	30,4332325	0,34755757	1,01500864	0,02149201	0,01504133	0,96201188	0,01681956	Unpaired t-te	0,973122513	permutation FDR (250 perm	1
3150	Sgcd~P8234	Sgcd	ko vs wt	ko	wt	31,7158878	0,26571863	32,0372082	0,14219237	0,80033703	-0,3213204	-0,2715173	0,14261503	0,84583469	Unpaired t-te	0,52757191	permutation FDR (250 perm	1
3151	Sgce~O7025	Sgce	ko vs wt	ko	wt	25,9488306	1,39880153	27,1878955	0,70316811	0,42364717	-1,2390649	-0,6359439	0,25173139	0,59906263	Unpaired t-te	0,168907631	permutation FDR (250 perm	1
3152	Sgcg~P8234	Sgcg	ko vs wt	ko	wt	28,7168409	0,31001343	28,2941836	0,0746396	1,34039413	0,42265727	0,35619019	0,09564097	1,01935605	Unpaired t-te	0,422448635	permutation FDR (250 perm	1
3153	Sgf29~Q9DA	Sgf29	ko vs wt	ko	wt	23,8576367	0,08283145	23,4602652	0,36401868	1,31710604	0,39737151	0,31492309	0,26052919	0,58414361	Unpaired t-te	0,494810474	permutation FDR (250 perm	1
3154	Sgpl1~Q8R0	Sgpl1	ko vs wt	ko	wt	29,7098116	0,63553682	29,3880922	0,85025864	1,24981918	0,32171938	0,18876395	0,67510532	0,17062847	Unpaired t-te	0,670321532	permutation FDR (250 perm	1
3155	Sh3bgrl~Q9J	Sh3bgrl	ko vs wt	ko	wt	24,8704494	0,53660145	26,0753216	0,26390527	0,43380776	-1,2048722	-0,8848506	0,02312755	1,63587046	Unpaired t-te	0,071370878	permutation FDR (250 perm	1
3156	Sh3bp4~Q92	Sh3bp4	ko vs wt	ko	wt	24,6417267	0,44388418	24,8737012	0,46254676	0,85146874	-0,2319745	-0,16388	0,60531602	0,21801783	Unpaired t-te	0,709050644	permutation FDR (250 perm	1
3157	Sh3gl1~Q62	Sh3gl1	ko vs wt	ko	wt	28,1505625	0,43461586	27,9351611	0,3666635	1,16102695	0,21540146	0,15828901	0,57791562	0,23813556	Unpaired t-te	0,717159486	permutation FDR (250 perm	1
3158	Sh3glb1~Q9J	Sh3glb1	ko vs wt	ko	wt	28,7989886	0,56253538	28,8408361	0,26451038	0,97141012	-0,0418476	-0,0304393	0,9158348	0,03818286	Unpaired t-te	0,946878604	permutation FDR (250 perm	1
3159	Sh3glb2~Q8	Sh3glb2	ko vs wt	ko	wt	28,2255917	0,3400017	27,5537733	0,0563754	1,5930796	0,67181836	0,55970553	0,03908447	1,40799576	Unpaired t-te	0,228283688	permutation FDR (250 perm	1
3160	Sh3pxd2b~A	Sh3pxd2b	ko vs wt	ko	wt	28,3686246	0,59309109	28,5462119	0,12885819	0,88418039	-0,1775874	-0,131125	0,64680011	0,18922992	Unpaired t-te	0,770981492	permutation FDR (250 perm	1
3161	Shc1~P9808	Shc1	ko vs wt	ko	wt	26,7998006	0,27682442	26,4918753	0,11861589	1,23792617	0,30792528	0,26084399	0,15694216	0,80426038	Unpaired t-te	0,546175737	permutation FDR (250 perm	1
3162	Shmt2~Q9C	Shmt2	ko vs wt	ko	wt	29,4151563	0,34115212	28,1151039	1,0770713	2,46237836	1,30005246	0,72764343	0,22529301	0,64725228	Unpaired t-te	0,150963636	permutation FDR (250 perm	1
3163	Shoc2~O885	Shoc2	ko vs wt	ko	wt	26,218349	0,0789433	26,8227167	0,2992316	0,65775958	-0,6043677	-0,4968324	0,097711	1,01005652	Unpaired t-te	0,295710979	permutation FDR (250 perm	1
3164	Sik2~Q8CFH	Sik2	ko vs wt	ko	wt	27,0311278	0,09057093	23,987038	0,43566111	8,24826006	3,04408982	2,31936756	0,00859705	2,06565045	Unpaired t-te	0,004160229	permutation FDR (250 perm	1
3165	Sik3~Q6P4S	Sik3	ko vs wt	ko	wt	24,2948538	0,23613823	23,9412865	0,260861	1,2777161	0,35356731	0,28760004	0,1982627	0,70275898	Unpaired t-te	0,509034974	permutation FDR (250 perm	1
3166	Sin3a~Q605	Sin3a	ko vs wt	ko	wt	26,3610223	0,28358048	25,7566954	0,66860754	1,52026929	0,60432689	0,40279762	0,32943273	0,48223325	Unpaired t-te	0,382958319	permutation FDR (250 perm	1
3167	Sipa1l1~Q8C	Sipa1l1	ko vs wt	ko	wt	24,2004612	0,21297383	24,1794753	0,43101079	1,01465265	0,02098592	0,01579504	0,95357799	0,02064378	Unpaired t-te	0,972839791	permutation FDR (250 perm	1
3168	Sipa1l2~Q80	Sipa1l2	ko vs wt	ko	wt	24,4830981	0,42140798	24,1863848	0,48616998	1,22834289	0,29671335	0,20878255	0,52129265	0,2829184	Unpaired t-te	0,633339994	permutation FDR (250 perm	1
3169	Sipa1~E9Q0	Sipa1	ko vs wt	ko	wt	24,8465798	1,14964675	25,8741425	0,26501252	0,49053816	-1,0275627	-0,6081352	0,22109345	0,65542413	Unpaired t-te	0,193835075	permutation FDR (250 perm	1
3170	Sirt2~Q8VD	Sirt2	ko vs wt	ko	wt	28,3132405	0,55986569	28,0765318	0,20597231	1,17830144	0,23670866	0,17475263	0,54014847	0,26748685	Unpaired t-te	0,689760782	permutation FDR (250 perm	1
3171	Skap2~Q3U	Skap2	ko vs wt	ko	wt	25,5968636	2,15083061	25,714861	0,10042659	0,92146589	-0,1179973	-0,0525879	0,93036533	0,03134648	Unpaired t-te	0,91181867	permutation FDR (250 perm	1
3172	Skic2~Q6NZ	Skic2	ko vs wt	ko	wt	26,6671419	0,64424988	26,9974939	0,46517054	0,79534237	-0,3303521	-0,220745	0,53547928	0,27125733	Unpaired t-te	0,606440758	permutation FDR (250 perm	1
3173	Skic3~F8VPK	Skic3	ko vs wt	ko	wt	27,4749934	0,28386003	27,1149624	0,35165669	1,28345349	0,36003102	0,27741454	0,29903482	0,52427824	Unpaired t-te	0,525699387	permutation FDR (250 perm	1
3174	Skic8~Q9ER	Skic8	ko vs wt	ko	wt	27,5742143	0,61177874	25,3778034	1,44931025	4,58337673	2,19641087	1,05395119	0,1547318	0,81042043	Unpaired t-te	0,053866076	permutation FDR (250 perm	1
3175	Skp1~Q9WT	Skp1	ko vs wt	ko	wt	25,9273948	0,98862062	24,4508624	1,16564041	2,78279082	1,47653246	0,73731833	0,21849756	0,6605534	Unpaired t-te	0,127238592	permutation FDR (250 perm	1
3176	Skt~A2AQ25	Skt	ko vs wt	ko	wt	25,6830375	1,10080648	27,2125586	0,29424318	0,34639233	-1,5295211	-0,9165719	0,09118584	1,04007258	Unpaired t-te	0,069774375	permutation FDR (250 perm	1
3177	Slc12a4~Q9J	Slc12a4	ko vs wt	ko	wt	27,7655286	0,45113234	27,7468211	0,12264449	1,0130515	0,01870752	0,0146781	0,94925021	0,0226193	Unpaired t-te	0,974377388	permutation FDR (250 perm	1
3178	Slc25a10~Q	Slc25a10	ko vs wt	ko	wt	27,2131569	0,16934847	26,8993029	0,34737933	1,24302385	0,31385398	0,24822793	0,3309518	0,48023525	Unpaired t-te	0,579047619	permutation FDR (250 perm	1
3179	Slc25a11~Q	Slc25a11	ko vs wt	ko	wt	30,176573	0,44181873	30,3404102	0,31354866	0,89264773	-0,1638371	-0,122452	0,64840786	0,18815173	Unpaired t-te	0,781699105	permutation FDR (250 perm	1
3180	Slc25a12~Q	Slc25a12	ko vs wt	ko	wt	29,6477531	0,13971661	29,2671174	0,10501781	1,30191542	0,38063572	0,34302582	0,01823391	1,73912009	Unpaired t-te	0,4288	permutation FDR (250 perm	1
3181	Slc25a13~Q	Slc25a13	ko vs wt	ko	wt	24,5324313	1,77392307	26,4400129	0,19706803	0,26653897	-1,9075816	-0,9380267	0,15886268	0,7989781	Unpaired t-te	0,069329918	permutation FDR (250 perm	1
3182	Slc25a17~O	Slc25a17	ko vs wt	ko	wt	24,6323075	1,13927591	24,4708188	0,91860303	1,11844067	0,16148872	0,08391522	0,86841734	0,06127152	Unpaired t-te	0,853954533	permutation FDR (250 perm	1
3183	Slc25a19~Q	Slc25a19	ko vs wt	ko	wt	24,6806972	0,34717368	24,4550924	0,66113715	1,16926725	0,22560471	0,14954051	0,69002459	0,16113543	Unpaired t-te	0,742026561	permutation FDR (250 perm	1
3184	Slc25a1~Q8J	Slc25a1	ko vs wt	ko	wt	30,1450139	0,54234006	30,8502892	0,50774833	0,61332546	-0,7052753	-0,4777022	0,20548046	0,68722948	Unpaired t-te	0,28155925	permutation FDR (250 perm	1
3185	Slc25a20~Q	Slc25a20	ko vs wt	ko	wt	28,5894234	0,59129051	28,5490389	0,2272943	1,02838787	0,0403845	0,029321	0,91971901	0,03634484	Unpaired t-te	0,948701347	permutation FDR (250 perm	1
3186	Slc25a22~Q	Slc25a22	ko vs wt	ko	wt	27,5571927	0,88506515	25,6328506	0,97715417	3,79563743	1,92434219	1,0349393	0,08886188	1,05128452	Unpaired t-te	0,043275073	permutation FDR (250 perm	1
3187	Slc25a24~Q	Slc25a24	ko vs wt	ko	wt	29,3864661	1,16115433	28,7240751	1,09032914	1,58270342	0,66239094	0,32764339	0,54861166	0,26073496	Unpaired t-te	0,45043756	permutation FDR (250 perm	1
3188	Slc25a35~Q	Slc25a35	ko vs wt	ko	wt	25,5600719	0,4370433	25,9890156	0,80895835	0,74280544	-0,4289437	-0,2639328	0,54541287	0,26327462	Unpaired t-te	0,554662224	permutation FDR (250 perm	1
3189	Slc25a3~Q8	Slc25a3	ko vs wt	ko	wt	31,5506227	0,32128505	31,7923748	0,31394218	0,84571763	-0,241752	-0,1875081	0,44675994	0,34992578	Unpaired t-te	0,667325203	permutation FDR (250 perm	1
3190	Slc25a4~P48	Slc25a4	ko vs wt	ko	wt	33,929028	0,18592544	34,0433905	0,12140609	0,92379043	-0,1143625	-0,1005429	0,44331663	0,35328598	Unpaired t-te	0,822114917	permutation FDR (250 perm	1
3191	Slc25a5~P51	Slc25a5	ko vs wt	ko	wt	32,1042957	0,23101239	32,1147979	0,3963918	0,99274682	-0,0105023	-0,0080145	0,97519667	0,01090779	Unpaired t-te	0,986349935	permutation FDR (250 perm	1
3192	Slc27a1~Q6	Slc27a1	ko vs wt	ko	wt	27,2428926	0,19224397	28,0912947	0,49715622	0,55539958	-0,848402	-0,6198833	0,12655259	0,89772896	Unpaired t-te	0,202650824	permutation FDR (250 perm	1
3193	Slc27a4~Q91	Slc27a4	ko vs wt	ko	wt	26,4337868	0,68303629	26,4241021	0,57161644	1,00673548	0,00968466	0,00618947	0,98701369	0,00567682	Unpaired t-te	0,988794999	permutation FDR (250 perm	1
3194	Slc2a3~P320	Slc2a3	ko vs wt	ko	wt	24,3518392	0,87261226	24,4687189	0,33814712	0,92218003	-0,1168797	-0,0750352	0,84379912	0,07376093	Unpaired t-te	0,872379602	permutation FDR (250 perm	1
3195	Slc33a1~Q9	Slc33a1	ko vs wt	ko	wt	24,1016164	0,30570906	24,5234819	0,16475298	0,74645878	-0,4218655	-0,3482228	0,10483099	0,97951033	Unpaired t-te	0,423049415	permutation FDR (250 perm	1
3196	Slc3a2~P108	Slc3a2	ko vs wt	ko	wt	28,4026725	0,34056937	27,8540468	0,82430361	1,46269168	0,5486257	0,33967627	0,45099991	0,34582355	Unpaired t-te	0,455770774	permutation FDR (250 perm	1
3197	Slc44a2~Q8	Slc44a2	ko vs wt	ko	wt	28,0577492	0,49064103	27,3590448	0,51088821	1,62304658	0,6987044	0,47886934	0,19988035	0,69922989	Unpaired t-te	0,28283127	permutation FDR (250 perm	1

3198	Slc4a1~P049	Slc4a1	ko vs wt	ko	wt	31,170838	0,72903789	30,8253215	0,41837025	1,27060582	0,34551653	0,22814311	0,53218647	0,27393617	Unpaired t-te	0,593728571	permutation FDR (250 perm	1
3199	Slc4a4~E9Q8	Slc4a4	ko vs wt	ko	wt	24,5365695	0,83381844	24,3257493	0,90080811	1,15734596	0,21082019	0,11722505	0,80462126	0,0944085	Unpaired t-te	0,794491071	permutation FDR (250 perm	1
3200	Slfn14~V9GX	Slfn14	ko vs wt	ko	wt	25,1480483	1,60891269	24,9179685	0,38168801	1,17289981	0,23007978	0,11695082	0,82529039	0,08339321	Unpaired t-te	0,797617852	permutation FDR (250 perm	1
3201	Slfn5~Q8CBA	Slfn5	ko vs wt	ko	wt	27,0852424	0,60287384	24,8888775	1,0715425	4,58323073	2,19636492	1,19770001	0,08238928	1,08412929	Unpaired t-te	0,030637419	permutation FDR (250 perm	1
3202	Slirp~Q9D8T	Slirp	ko vs wt	ko	wt	24,7058393	1,06768008	24,3475568	0,93782769	1,2818989	0,35828249	0,18803578	0,70984589	0,14883593	Unpaired t-te	0,665172915	permutation FDR (250 perm	1
3203	Slit3~Q9WVE	Slit3	ko vs wt	ko	wt	29,0068647	0,40918623	29,2607853	0,25406578	0,83861432	-0,2539206	-0,1958067	0,43149986	0,36501934	Unpaired t-te	0,649430572	permutation FDR (250 perm	1
3204	Slk~O54988	Slk	ko vs wt	ko	wt	27,7671947	0,50271511	27,3968607	0,44695886	1,29265207	0,37033401	0,2591379	0,43071041	0,36581463	Unpaired t-te	0,547385176	permutation FDR (250 perm	1
3205	Slmap~D3Z7	Slmap	ko vs wt	ko	wt	31,6357995	0,6656551	32,1157704	0,20845016	0,71699208	-0,4799709	-0,3400161	0,31113224	0,50705498	Unpaired t-te	0,438517186	permutation FDR (250 perm	1
3206	Sltm~Q8CH2	Sltm	ko vs wt	ko	wt	24,309665	0,52854993	25,1687498	0,82525913	0,55130217	-0,8590848	-0,5179829	0,28071571	0,55173328	Unpaired t-te	0,261601732	permutation FDR (250 perm	1
3207	Smad3~Q8B	Smad3	ko vs wt	ko	wt	27,2065554	0,41315397	26,9215364	0,7790764	1,21842628	0,28501896	0,17810173	0,67000605	0,17392127	Unpaired t-te	0,693009195	permutation FDR (250 perm	1
3208	Smad4~P974	Smad4	ko vs wt	ko	wt	24,4890739	1,4058871	23,4993766	0,67458499	1,9857682	0,98969723	0,50976608	0,3451514	0,46199036	Unpaired t-te	0,254255814	permutation FDR (250 perm	1
3209	Smap1~Q91	Smap1	ko vs wt	ko	wt	24,3361892	0,46759872	25,1733846	1,44111945	0,55973061	-0,8371954	-0,407557	0,50125957	0,29993733	Unpaired t-te	0,381820607	permutation FDR (250 perm	1
3210	Smap2~Q7TI	Smap2	ko vs wt	ko	wt	24,0235699	0,56126024	25,7180133	0,35056304	0,30897382	-1,6944435	-1,203453	0,008958	2,04778914	Unpaired t-te	0,020195884	permutation FDR (250 perm	1
3211	Smap~Q9R0	Smap	ko vs wt	ko	wt	24,4420142	0,18262329	23,8432665	0,41042593	1,51440142	0,59874767	0,4574872	0,16453431	0,78374353	Unpaired t-te	0,324878318	permutation FDR (250 perm	1
3212	Smarca2~Q6	Smarca2	ko vs wt	ko	wt	28,2702533	0,56647717	28,3863156	0,31621644	0,92270268	-0,1160622	-0,0831282	0,78164154	0,10699237	Unpaired t-te	0,855507119	permutation FDR (250 perm	1
3213	Smarca4~Q3	Smarca4	ko vs wt	ko	wt	23,9098146	0,46559013	25,3474685	2,12128076	0,36916714	-1,437654	-0,5696232	0,43982466	0,35672043	Unpaired t-te	0,24187187	permutation FDR (250 perm	1
3214	Smarca5~Q9	Smarca5	ko vs wt	ko	wt	28,6676236	0,98423596	28,2659107	0,65939354	1,32107542	0,40171283	0,23152736	0,60821134	0,21594549	Unpaired t-te	0,588374925	permutation FDR (250 perm	1
3215	Smarcad1~Q	Smarcad1	ko vs wt	ko	wt	24,1468198	0,84646899	24,6767635	0,554053	0,69258175	-0,5299437	-0,325847	0,4361332	0,36038085	Unpaired t-te	0,449962312	permutation FDR (250 perm	1
3216	Smarcal1~Q8	Smarcal1	ko vs wt	ko	wt	24,4540657	0,464155	23,5583554	0,18278537	1,86052572	0,89571033	0,69032453	0,03695068	1,43237752	Unpaired t-te	0,144548954	permutation FDR (250 perm	1
3217	Smarcb1~Q9	Smarcb1	ko vs wt	ko	wt	24,5609214	1,76232334	24,4308736	1,19845031	1,09432998	0,13004783	0,05595474	0,92558144	0,03358536	Unpaired t-te	0,902663438	permutation FDR (250 perm	1
3218	Smarcc1~P97	Smarcc1	ko vs wt	ko	wt	24,7624145	0,96847153	24,3455911	0,40280712	1,33498489	0,41682341	0,25611066	0,54030131	0,26736398	Unpaired t-te	0,55354956	permutation FDR (250 perm	1
3219	Smarcc2~Q6	Smarcc2	ko vs wt	ko	wt	29,5605253	0,23514215	28,5323149	0,32140326	2,03949282	1,02821043	0,81298971	0,02416454	1,6168215	Unpaired t-te	0,101632766	permutation FDR (250 perm	1
3220	Smarcd3~Q6	Smarcd3	ko vs wt	ko	wt	27,3697076	0,26773914	25,2569805	1,68135429	4,32508102	2,11272716	0,96080917	0,2159585	0,66562969	Unpaired t-te	0,080053097	permutation FDR (250 perm	1
3221	Smc1a~Q9CU	Smc1a	ko vs wt	ko	wt	29,2521172	0,2589547	29,8646564	0,3929872	0,65404456	-0,6125392	-0,4656144	0,14316725	0,84415631	Unpaired t-te	0,307097902	permutation FDR (250 perm	1
3222	Smc3~Q9CW	Smc3	ko vs wt	ko	wt	29,1969081	0,44919139	28,7790344	0,56536929	1,33595705	0,41787363	0,28301099	0,43498369	0,36152702	Unpaired t-te	0,518637512	permutation FDR (250 perm	1
3223	Smchd1~Q6F	Smchd1	ko vs wt	ko	wt	27,8456829	0,84260404	27,3736994	0,13107229	1,38701508	0,47198347	0,31565992	0,4065713	0,39086328	Unpaired t-te	0,476642317	permutation FDR (250 perm	1
3224	Smg6~P614C	Smg6	ko vs wt	ko	wt	24,5864792	0,58095873	24,099098	0,99234616	1,40189785	0,48738123	0,27415774	0,57648863	0,23920925	Unpaired t-te	0,538654445	permutation FDR (250 perm	1
3225	Smg8~Q8VE	Smg8	ko vs wt	ko	wt	24,191883	0,61511054	23,6904769	0,36638388	1,41559261	0,50140613	0,348298	0,3062599	0,51390987	Unpaired t-te	0,422089727	permutation FDR (250 perm	1
3226	Smoc1~E9QK	Smoc1	ko vs wt	ko	wt	28,6209892	1,33728157	29,6287255	0,40997388	0,49732596	-1,0077364	-0,5522721	0,29220443	0,5343132	Unpaired t-te	0,227847704	permutation FDR (250 perm	1
3227	Smoc2~Q8CD	Smoc2	ko vs wt	ko	wt	25,8878797	1,91979512	24,5831173	1,92424005	2,47043045	1,30476244	0,47360471	0,49595444	0,30455822	Unpaired t-te	0,286952045	permutation FDR (250 perm	1
3228	Smtn~D3Z3C	Smtn	ko vs wt	ko	wt	33,700827	0,7850888	34,7182298	0,30005472	0,49400489	-1,0174028	-0,6780556	0,10915723	0,96194748	Unpaired t-te	0,150358592	permutation FDR (250 perm	1
3229	Smu1~Q3UK	Smu1	ko vs wt	ko	wt	28,6595133	0,41871507	28,6033465	0,26189509	1,03969964	0,0561668	0,04305538	0,86094152	0,06502635	Unpaired t-te	0,926024019	permutation FDR (250 perm	1
3230	Smyd2~Q8R5	Smyd2	ko vs wt	ko	wt	24,4474559	0,48078364	23,509805	0,46669864	1,91540689	0,9376509	0,65513883	0,08952961	1,04803331	Unpaired t-te	0,160242375	permutation FDR (250 perm	1
3231	Snd1~Q78PY	Snd1	ko vs wt	ko	wt	32,8058334	0,41865676	32,5564733	0,3510617	1,18867981	0,24936016	0,18519445	0,50557451	0,29621483	Unpaired t-te	0,669952409	permutation FDR (250 perm	1
3232	Sned1~Q70E	Sned1	ko vs wt	ko	wt	24,4409872	0,24233117	24,469057	0,28498162	0,98073158	-0,0280698	-0,0225402	0,91468751	0,03872725	Unpaired t-te	0,960694189	permutation FDR (250 perm	1
3233	Snrk~Q8VDU	Snrk	ko vs wt	ko	wt	24,5647138	0,48577469	24,140701	0,47153723	1,34165421	0,42401289	0,29533572	0,3811765	0,41887388	Unpaired t-te	0,495592269	permutation FDR (250 perm	1
3234	Snrnp200~Q6	Snrnp200	ko vs wt	ko	wt	30,7247963	0,50219939	30,3606284	0,2062839	1,28713908	0,36416795	0,27493331	0,32060232	0,49403333	Unpaired t-te	0,525120589	permutation FDR (250 perm	1
3235	Snrnp40~Q6I	Snrnp40	ko vs wt	ko	wt	27,9461484	0,18209775	28,1712193	0,20700168	0,85555301	-0,2250708	-0,190703	0,28136389	0,55073164	Unpaired t-te	0,663651264	permutation FDR (250 perm	1
3236	Snrnp70~Q62	Snrnp70	ko vs wt	ko	wt	28,5752067	0,30877624	28,5401051	0,50531507	1,02462893	0,03510153	0,02508479	0,93549886	0,02895674	Unpaired t-te	0,956048408	permutation FDR (250 perm	1
3237	Snrpa1~P577	Snrpa1	ko vs wt	ko	wt	24,6525319	0,81202539	24,8078875	1,7511562	0,89791103	-0,1553556	-0,0668473	0,91511947	0,0385222	Unpaired t-te	0,889160314	permutation FDR (250 perm	1
3238	Snrpb~P2704	Snrpb	ko vs wt	ko	wt	31,2480433	0,42878988	31,0147329	0,34023091	1,17552923	0,23331041	0,17343875	0,52999427	0,27572882	Unpaired t-te	0,688830362	permutation FDR (250 perm	1
3239	Snrpc~Q6224	Snrpc	ko vs wt	ko	wt	27,611844	0,2715063	27,5615485	0,35097137	1,03547702	0,05029554	0,03888227	0,8732953	0,05883888	Unpaired t-te	0,932941552	permutation FDR (250 perm	1
3240	Snrpd1~P623	Snrpd1	ko vs wt	ko	wt	29,4431859	0,319297	29,3219836	0,75629162	1,08764086	0,12120226	0,07741285	0,84670724	0,07226673	Unpaired t-te	0,873515812	permutation FDR (250 perm	1
3241	Snrpd2~P623	Snrpd2	ko vs wt	ko	wt	30,0936462	0,1835624	29,9189723	0,14163244	1,12870921	0,17467385	0,15244526	0,28571145	0,54407235	Unpaired t-te	0,726779545	permutation FDR (250 perm	1
3242	Snrpd3~P623	Snrpd3	ko vs wt	ko	wt	29,5373369	0,48833411	29,4541638	0,35582221	1,05934538	0,08317303	0,06036296	0,83456073	0,07854206	Unpaired t-te	0,894362901	permutation FDR (250 perm	1
3243	Snrpe~P623C	Snrpe	ko vs wt	ko	wt	24,3247289	0,25454416	26,8463424	0,71173341	0,17414808	-2,5216135	-1,6542875	0,02941114	1,53148821	Unpaired t-te	0,010688	permutation FDR (250 perm	1
3244	Snrpf~P6230	Snrpf	ko vs wt	ko	wt	27,8281033	0,79048278	27,5996556	0,49990205	1,17157369	0,2284477	0,14483756	0,70866018	0,14956197	Unpaired t-te	0,739672038	permutation FDR (250 perm	1
3245	Snrpg~P623C	Snrpg	ko vs wt	ko	wt	26,0239668	0,67468109	24,539027	0,89206483	2,79905489	1,48493978	0,85274494	0,12622287	0,89886195	Unpaired t-te	0,088099569	permutation FDR (250 perm	1
3246	Snta1~A2AKI	Snta1	ko vs wt	ko	wt	29,6117156	0,52633622	29,8544131	0,34236614	0,8451636	-0,2426975	-0,1747882	0,55960081	0,25212166	Unpaired t-te	0,686161197	permutation FDR (250 perm	1
3247	Sntb1~Q99L8	Sntb1	ko vs wt	ko	wt	30,8464501	0,20413758	31,1278242	0,26921147	0,82280695	-0,2813741	-0,2299009	0,28647051	0,54292007	Unpaired t-te	0,599148203	permutation FDR (250 perm	1
3248	Sntb2~Q6123	Sntb2	ko vs wt	ko	wt	32,118659	0,23584822	32,2982271	0,31528799	0,8829673	-0,1795681	-0,1423746	0,53523118	0,2714586	Unpaired t-te	0,749865238	permutation FDR (250 perm	1
3249	Sntg2~Q925I	Sntg2	ko vs wt	ko	wt	27,1411222	0,03231559	27,1251025	0,26853422	1,01116588	0,01601969	0,01345291	0,94060541	0,02659253	Unpaired t-te	0,978032427	permutation FDR (250 perm	1
3250	Snu13~Q9D0	Snu13	ko vs wt	ko	wt	28,0581508	0,42922822	27,9851023	0,28162057	1,05193717	0,07304854	0,05542752	0,8273716	0,08229939	Unpaired t-te	0,903362194	permutation FDR (250 perm	1
3251	Snw1~A0A0E	Snw1	ko vs wt	ko	wt	26,3739294	1,48735662	25,5632618	1,39134479	1,75402298	0,81066765	0,35156497	0,56496944	0,24797504	Unpaired t-te	0,42038399	permutation FDR (250 perm	1
3252	Snx12~Q6ZW	Snx12	ko vs wt	ko	wt	27,6198884	1,07963288	27,8560366	1,19237023	0,84900902	-0,2361482	-0,1152771	0,83287561	0,07941985	Unpaired t-te	0,79916318	permutation FDR (250 perm	1
3253	Snx15~Q91W	Snx15	ko vs wt	ko	wt	24,1129109	0,38409176	24,2646736	0,63843831	0,90014994	-0,1517628	-0,1009754	0,78276884	0,10636647	Unpaired t-te	0,827809156	permutation FDR (250 perm	1
3254	Snx17~Q8BV	Snx17	ko vs wt	ko	wt	27,3157856	0,37727121	25,0669828	1,73310656	4,75288262	2,24880277	1,00182855	0,20496441	0,68832155	Unpaired t-te	0,069932653	permutation FDR (250 perm	1
3255	Snx18~Q8C7	Snx18	ko vs wt	ko	wt	31,0582852	0,28408211	30,9828772	0,2792934	1,05365896	0,07540798	0,06000398	0,78248825	0,10652218	Unpaired t-te	0,895736927	permutation FDR (250 perm	1
3256	Snx1~Q9WV	Snx1	ko vs wt	ko	wt	28,2961952	0,51353762	28,3238644	0,29341638	0,98100395	-0,0276692	-0,020317	0,94207835	0,02591298	Unpaired t-te	0,964304782	permutation FDR (250 perm	1
3257	Snx21~Q3UR	Snx21	ko vs wt	ko	wt	25,6574587	1,16350327	24,1701682	0,64103389	2,80361938	1,4872905	0,82153692	0,12774834	0,89364472	Unpaired t-te	0,08865999	permutation FDR (250 perm	1
3258	Snx27~Q3UH	Snx27	ko vs wt	ko	wt	27,2043797	0,27471368	26,9435089	0,23245705	1,19820168	0,26087076	0,21236348	0,30795389	0,51151431	Unpaired t-te	0,622282852	permutation FDR (250 perm	1
3259	Snx2~Q9CWI	Snx2	ko vs wt	ko	wt	29,8053168	0,10551536	29,8352378	0,20886864	0,97947395	-0,029921	-0,0257992	0,86467281	0,0631482	Unpaired t-te	0,955375625	permutation FDR (250 perm	1
3260	Snx33~Q4VA	Snx33	ko vs wt	ko	wt	26,4647658	0,44645814	26,4886271	0,07510298	0,98359662	-0,0238613	-0,0188899	0,93305853	0,03009111	Unpaired t-te	0,967914961	permutation FDR (250 perm	1
3261	Snx3~Q78ZM	Snx3	ko vs wt	ko	wt	28,2371246	0,50241651	27,70095	0,62900802	1,45012233	0,53617461	0,35021087	0,37517808	0,42576255	Unpaired t-te	0,42842589	permutation FDR (250 perm	1

3262	Snx4~Q91YJ	Snx4	ko vs wt	ko	wt	28,3237468	0,50691971	27,2814614	0,38974413	2,0594875	1,04228537	0,74342612	0,04962469	1,30430222	Unpaired t-te	0,118026786	permutation FDR (250 perm	1
3263	Snx5~Q9D8U	Snx5	ko vs wt	ko	wt	29,893797	0,21679586	29,3312462	0,27910444	1,47687813	0,56255078	0,45598609	0,08193607	1,08652485	Unpaired t-te	0,311859282	permutation FDR (250 perm	1
3264	Snx6~Q6P8X	Snx6	ko vs wt	ko	wt	29,9077254	0,36458376	29,8900529	0,08537718	1,01232501	0,01767254	0,01449782	0,94010189	0,02682508	Unpaired t-te	0,9748715	permutation FDR (250 perm	1
3265	Snx7~F8WI3	Snx7	ko vs wt	ko	wt	27,3142664	0,48021617	26,8669557	0,29885211	1,36349618	0,44731066	0,33168396	0,25592943	0,59187976	Unpaired t-te	0,441940404	permutation FDR (250 perm	1
3266	Snx8~Q8CFD	Snx8	ko vs wt	ko	wt	26,8085949	0,23226511	25,9637265	0,18936409	1,79610091	0,84486841	0,71026954	0,00731478	2,13579848	Unpaired t-te	0,132441189	permutation FDR (250 perm	1
3267	Snx9~Q91VH	Snx9	ko vs wt	ko	wt	30,7942272	0,42744113	30,644133	0,29818763	1,10964193	0,15009421	0,11331355	0,66325049	0,17832242	Unpaired t-te	0,800293445	permutation FDR (250 perm	1
3268	Soat1~Q612	Soat1	ko vs wt	ko	wt	25,9799548	0,32060141	24,9542245	0,56303536	2,03598978	1,02573032	0,71278225	0,10556333	0,97648694	Unpaired t-te	0,147018303	permutation FDR (250 perm	1
3269	Sod2~P0967	Sod2	ko vs wt	ko	wt	24,0085282	0,40526866	25,791855	0,59982722	0,29051271	-1,7833268	-1,2013784	0,03105818	1,50782393	Unpaired t-te	0,027428191	permutation FDR (250 perm	1
3270	Sod3~O0916	Sod3	ko vs wt	ko	wt	32,3267868	0,50586051	31,9875191	0,20703809	1,26511427	0,3392677	0,25572365	0,35398762	0,45101193	Unpaired t-te	0,554300606	permutation FDR (250 perm	1
3271	Soga1~A2AC	Soga1	ko vs wt	ko	wt	25,2318273	0,83349031	24,7889868	0,34296251	1,35927789	0,44284043	0,28776997	0,45431366	0,3426442	Unpaired t-te	0,506925318	permutation FDR (250 perm	1
3272	Son~Q9QX47	Son	ko vs wt	ko	wt	28,7041701	0,43100563	28,6839022	0,36368488	1,01414778	0,0202679	0,01492648	0,95714614	0,01902175	Unpaired t-te	0,973211201	permutation FDR (250 perm	1
3273	Sorbs1~E9Q	Sorbs1	ko vs wt	ko	wt	31,3584327	0,27316563	31,6283473	0,15176556	0,82936863	-0,2699146	-0,2266741	0,21785825	0,66182599	Unpaired t-te	0,596763831	permutation FDR (250 perm	1
3274	Sorbs2~Q3U	Sorbs2	ko vs wt	ko	wt	32,4207351	0,49428979	32,2594092	0,12822688	1,11831444	0,16132589	0,12415071	0,62176988	0,20637032	Unpaired t-te	0,783362416	permutation FDR (250 perm	1
3275	Sorbs3~Q9R	Sorbs3	ko vs wt	ko	wt	30,2841405	0,21719041	29,9955981	0,47799631	1,22140565	0,28854243	0,21208474	0,49112972	0,30880379	Unpaired t-te	0,63864246	permutation FDR (250 perm	1
3276	Sord~Q6444	Sord	ko vs wt	ko	wt	25,7498481	1,13565349	23,8504369	0,76354937	3,73060907	1,89941119	1,02706591	0,07556908	1,12165585	Unpaired t-te	0,040228164	permutation FDR (250 perm	1
3277	Sorl1~O8830	Sorl1	ko vs wt	ko	wt	27,7706054	0,25036418	28,4721346	0,23252902	0,6149201	-0,7015291	-0,5755302	0,02775462	1,55666472	Unpaired t-te	0,206643239	permutation FDR (250 perm	1
3278	Sort1~Q6PH	Sort1	ko vs wt	ko	wt	25,3225198	1,12152869	24,6754039	0,95763256	1,56603433	0,64711584	0,33409653	0,52233234	0,28205308	Unpaired t-te	0,440211742	permutation FDR (250 perm	1
3279	Sos2~Q0238	Sos2	ko vs wt	ko	wt	24,4734457	0,34015104	24,1147338	0,15468117	1,28228054	0,35871193	0,29287618	0,17784815	0,74995065	Unpaired t-te	0,498258867	permutation FDR (250 perm	1
3280	Sost~B2RQA	Sost	ko vs wt	ko	wt	30,1460642	0,12724484	30,7017441	0,62440536	0,68033636	-0,5556799	-0,3838651	0,33459999	0,47547408	Unpaired t-te	0,411503106	permutation FDR (250 perm	1
3281	Sp100~Q8C4	Sp100	ko vs wt	ko	wt	24,4178144	1,58925713	24,4097288	0,42654863	1,00562027	0,00808564	0,00411303	0,99376131	0,00271792	Unpaired t-te	0,992900807	permutation FDR (250 perm	1
3282	Sp110~Q8BV	Sp110	ko vs wt	ko	wt	25,5649114	1,64776113	23,428139	0,80536116	4,39777068	2,13677238	1,01328523	0,1163457	0,93424967	Unpaired t-te	0,043815528	permutation FDR (250 perm	1
3283	Spag9~Q58A	Spag9	ko vs wt	ko	wt	26,7749141	0,7384368	27,0978345	0,23487599	0,79944995	-0,3229204	-0,2215511	0,52072105	0,28339487	Unpaired t-te	0,610674157	permutation FDR (250 perm	1
3284	Sparc~Q5NC	Sparc	ko vs wt	ko	wt	28,2624486	0,7844698	27,5680873	1,70362891	1,6181679	0,69436131	0,30361551	0,63268373	0,19881333	Unpaired t-te	0,502088903	permutation FDR (250 perm	1
3285	Spart~Q8R1	Spart	ko vs wt	ko	wt	27,627047	0,38625447	28,0079765	0,10818745	0,76794263	-0,3809296	-0,3082551	0,18776629	0,72638239	Unpaired t-te	0,482578699	permutation FDR (250 perm	1
3286	Spata13~E9	Spata13	ko vs wt	ko	wt	24,7032073	0,27503666	24,9799189	0,73133201	0,82547045	-0,2767115	-0,1795708	0,65255069	0,18538574	Unpaired t-te	0,695048549	permutation FDR (250 perm	1
3287	Spats2l~Q91	Spats2l	ko vs wt	ko	wt	24,5574082	0,88557807	27,9372584	0,06598759	0,09606467	-3,3798502	-2,233263	0,00677608	2,1690216	Unpaired t-te	0,003284916	permutation FDR (250 perm	1
3288	Spcs2~Q9CY	Spcs2	ko vs wt	ko	wt	29,4285845	0,64491736	28,302875	0,7977306	2,18208827	1,12570946	0,67170867	0,17773828	0,75021904	Unpaired t-te	0,156851897	permutation FDR (250 perm	1
3289	Spcs3~Q6ZW	Spcs3	ko vs wt	ko	wt	25,9280081	2,18695055	27,7301757	0,01066064	0,28674344	-1,8021676	-0,7964822	0,24877699	0,60418978	Unpaired t-te	0,111960378	permutation FDR (250 perm	1
3290	Specc1l~Q2K	Specc1l	ko vs wt	ko	wt	24,4275899	0,40068666	27,3912912	0,41373699	0,12818494	-2,9637013	-2,1586078	0,00111587	2,95238794	Unpaired t-te	0,003050549	permutation FDR (250 perm	1
3291	Specc1~Q5S	Specc1	ko vs wt	ko	wt	27,643742	0,22625452	28,0086308	0,17161404	0,77652875	-0,3648888	-0,309675	0,09700507	1,01320557	Unpaired t-te	0,471488182	permutation FDR (250 perm	1
3292	Speg~Q6240	Speg	ko vs wt	ko	wt	27,7764473	0,42452295	28,7624248	0,50223009	0,50488351	-0,9859775	-0,6887731	0,0880666	1,05518877	Unpaired t-te	0,148026556	permutation FDR (250 perm	1
3293	Sphk2~Q9JIA	Sphk2	ko vs wt	ko	wt	24,9128185	0,40690937	23,9096876	0,98620273	2,00434506	1,0031309	0,57788705	0,28420719	0,54636494	Unpaired t-te	0,228200075	permutation FDR (250 perm	1
3294	Spon1~Q8VC	Spon1	ko vs wt	ko	wt	27,4820292	0,64901178	26,5943785	0,08844774	1,85016088	0,88765072	0,64327612	0,09695764	1,01341797	Unpaired t-te	0,176525316	permutation FDR (250 perm	1
3295	Spp1~P1092	Spp1	ko vs wt	ko	wt	25,7344681	1,84444472	29,0620142	1,38674179	0,09961134	-3,3275461	-1,3595191	0,0706438	1,15092596	Unpaired t-te	0,011959356	permutation FDR (250 perm	1
3296	Spp2~Q8K1	Spp2	ko vs wt	ko	wt	28,0071343	0,33476905	25,5645045	2,024109	5,43631804	2,44262986	0,99933613	0,22844959	0,64120962	Unpaired t-te	0,071439878	permutation FDR (250 perm	1
3297	Spryd3~E9Q	Spryd3	ko vs wt	ko	wt	24,9753689	1,25921068	23,7783916	1,24304573	2,29258835	1,19697734	0,55916123	0,3490879	0,4570652	Unpaired t-te	0,218182913	permutation FDR (250 perm	1
3298	Spr~Q91XH5	Spr	ko vs wt	ko	wt	29,2002923	0,65826501	29,1700105	0,69127549	1,02121155	0,03028177	0,01870206	0,96326214	0,01625551	Unpaired t-te	0,967634646	permutation FDR (250 perm	1
3299	Spta1~P080	Spta1	ko vs wt	ko	wt	32,0089125	0,86939511	31,6848322	0,98233725	1,25186612	0,32408028	0,17451854	0,72489124	0,13972715	Unpaired t-te	0,69120414	permutation FDR (250 perm	1
3300	Sptan1~A3K	Sptan1	ko vs wt	ko	wt	24,7495365	0,30585035	25,5050725	1,80661062	0,5923263	-0,755536	-0,3299841	0,61534569	0,21088084	Unpaired t-te	0,476894207	permutation FDR (250 perm	1
3301	Sptan1~P165	Sptan1	ko vs wt	ko	wt	34,601484	0,17640712	34,7669711	0,18973029	0,89162742	-0,1654871	-0,1416308	0,3806184	0,41951022	Unpaired t-te	0,74848519	permutation FDR (250 perm	1
3302	Sptbn1~Q62	Sptbn1	ko vs wt	ko	wt	34,9338782	0,15437809	35,0788306	0,130806	0,90440927	-0,1449523	-0,1284526	0,31315067	0,50424666	Unpaired t-te	0,771099271	permutation FDR (250 perm	1
3303	Sptb~Q3UG	Sptb	ko vs wt	ko	wt	31,3823768	0,91579252	31,3151314	0,88272667	1,04771431	0,06724538	0,03698819	0,93809584	0,02775279	Unpaired t-te	0,935712238	permutation FDR (250 perm	1
3304	Sptlc2~P973	Sptlc2	ko vs wt	ko	wt	25,622239	2,66628184	27,790503	0,11019957	0,22247821	-2,168264	-0,853194	0,25376793	0,59556326	Unpaired t-te	0,092514851	permutation FDR (250 perm	1
3305	Sqor~Q9R11	Sqor	ko vs wt	ko	wt	30,0456571	0,42161522	30,0923855	0,29248389	0,96812929	-0,0467284	-0,0354159	0,88941374	0,05089617	Unpaired t-te	0,938105598	permutation FDR (250 perm	1
3306	Sqstm1~Q64	Sqstm1	ko vs wt	ko	wt	26,7461914	0,89896741	26,8397369	0,49930223	0,93721667	-0,0935455	-0,0574703	0,88752201	0,05182087	Unpaired t-te	0,900058158	permutation FDR (250 perm	1
3307	Srbd1~Q497	Srbd1	ko vs wt	ko	wt	24,8868292	1,02259165	24,7737883	1,06600428	1,08150538	0,11304085	0,05774847	0,91149805	0,04024426	Unpaired t-te	0,900588045	permutation FDR (250 perm	1
3308	Src~P05480	Src	ko vs wt	ko	wt	28,1349974	1,02446982	27,4773102	0,13060657	1,57755163	0,65768722	0,41140306	0,34883683	0,45737767	Unpaired t-te	0,363251857	permutation FDR (250 perm	1
3309	Srgap2~Q91	Srgap2	ko vs wt	ko	wt	27,1024458	0,86903231	27,1811789	0,60784569	0,9468888	-0,0787331	-0,0474106	0,90978874	0,04105944	Unpaired t-te	0,917544798	permutation FDR (250 perm	1
3310	Sri~Q6P069	Sri	ko vs wt	ko	wt	29,808909	0,54872937	29,1822156	0,23205503	1,54402209	0,62669339	0,46189721	0,14796627	0,82983726	Unpaired t-te	0,298042076	permutation FDR (250 perm	1
3311	Srl~Q7TQ48	Srl	ko vs wt	ko	wt	24,2925213	0,93144642	25,1273617	1,03601354	0,56064505	-0,8348404	-0,4373713	0,41065025	0,38652791	Unpaired t-te	0,326050964	permutation FDR (250 perm	1
3312	Srm~Q64674	Srm	ko vs wt	ko	wt	26,8416517	0,94539523	24,500587	0,45810223	5,06676421	2,34106469	1,43210056	0,01628217	1,78828778	Unpaired t-te	0,010175503	permutation FDR (250 perm	1
3313	Srp14~P162	Srp14	ko vs wt	ko	wt	24,5855334	0,46678232	27,0152427	0,74453619	0,18560284	-2,4297093	-1,5267409	0,02535914	1,59586547	Unpaired t-te	0,010856134	permutation FDR (250 perm	1
3314	Srp19~G5E8	Srp19	ko vs wt	ko	wt	24,4420735	0,48455341	24,4971802	0,4852896	0,96252328	-0,0551067	-0,0381959	0,90657256	0,04259743	Unpaired t-te	0,933555792	permutation FDR (250 perm	1
3315	Srp54~P145	Srp54	ko vs wt	ko	wt	28,6589838	0,41951314	28,4933004	0,59448454	1,12169727	0,16568337	0,11156007	0,75329381	0,1230356	Unpaired t-te	0,807237989	permutation FDR (250 perm	1
3316	Srp68~Q8B	Srp68	ko vs wt	ko	wt	28,5259621	0,55596735	28,1207339	0,31370982	1,32429832	0,40522815	0,29149337	0,34769931	0,45879618	Unpaired t-te	0,498304916	permutation FDR (250 perm	1
3317	Srp72~F8VQ	Srp72	ko vs wt	ko	wt	29,9470443	0,43485178	30,0089744	0,29819348	0,95798158	-0,0619302	-0,0466391	0,85761047	0,06670993	Unpaired t-te	0,919440492	permutation FDR (250 perm	1
3318	Srp9~P49962	Srp9	ko vs wt	ko	wt	27,8129509	0,82937144	25,8260375	1,39761342	3,96388029	1,98691339	0,94698063	0,16993964	0,7697053	Unpaired t-te	0,069022063	permutation FDR (250 perm	1
3319	Srpra~Q9DB	Srpra	ko vs wt	ko	wt	28,1247384	0,57103925	27,7424697	0,41659954	1,30338987	0,38226869	0,26507354	0,42707429	0,36949658	Unpaired t-te	0,537168704	permutation FDR (250 perm	1
3320	Srpx2~Q8R0	Srpx2	ko vs wt	ko	wt	31,4879911	0,41949465	31,0234149	0,41772456	1,37991199	0,46457625	0,33616816	0,28643414	0,54297523	Unpaired t-te	0,439987163	permutation FDR (250 perm	1
3321	Srpx~Q9R0	Srpx	ko vs wt	ko	wt	29,2790959	0,35635828	28,8652854	0,51089546	1,33219988	0,41381055	0,29229332	0,38724553	0,41201359	Unpaired t-te	0,508496748	permutation FDR (250 perm	1
3322	Srrm1~Q52K	Srrm1	ko vs wt	ko	wt	27,0351049	0,80357125	27,6371633	0,11204966	0,65881331	-0,6020584	-0,40938	0,28634702	0,54310733	Unpaired t-te	0,364947439	permutation FDR (250 perm	1
3323	Srrm2~Q8BT	Srrm2	ko vs wt	ko	wt	26,7717605	0,05536482	26,9478604	0,43733725	0,88509245	-0,1760999	-0,134336	0,62713267	0,20264058	Unpaired t-te	0,776441333	permutation FDR (250 perm	1
3324	Srrt~Q99MR	Srrt	ko vs wt	ko	wt	28,9529057	0,22640899	28,7147991	0,54485255	1,17944374	0,2381066	0,16924919	0,60755319	0,21641569	Unpaired t-te	0,711722047	permutation FDR (250 perm	1
3325	Srsf10~Q9R	Srsf10	ko vs wt	ko	wt	26,7064673	0,54891346	26,3596007	0,55587569	1,27179539	0,34686658	0,2304899	0,52776176	0,27756208	Unpaired t-te	0,592817857	permutation FDR (250 perm	1

3326	Srsf11~F6RD	Srsf11	ko vs wt	ko	wt	27,1043638	0,38323465	24,0351098	0,14453385	8,39339174	3,06925392	2,46779386	0,00018803	3,72577786	Unpaired t-te	0,002198758	permutation FDR (250 perm	1
3327	Srsf1~Q6PDN	Srsf1	ko vs wt	ko	wt	29,7210591	0,29581288	29,4653937	0,67148772	1,19388633	0,25566548	0,1699231	0,65327992	0,18490069	Unpaired t-te	0,709546911	permutation FDR (250 perm	1
3328	Srsf2~Q6209	Srsf2	ko vs wt	ko	wt	28,5756464	0,39730085	28,0559696	0,48417291	1,43363409	0,51967685	0,36801662	0,28092079	0,55141612	Unpaired t-te	0,405795865	permutation FDR (250 perm	1
3329	Srsf3~P8410	Srsf3	ko vs wt	ko	wt	30,8356497	0,49555885	30,9350512	0,84468019	0,93342017	-0,0994014	-0,0597986	0,89045536	0,05038785	Unpaired t-te	0,899207324	permutation FDR (250 perm	1
3330	Srsf5~O3532	Srsf5	ko vs wt	ko	wt	24,7506707	1,76102331	28,3294498	1,00524261	0,08369127	-3,578779	-1,5972694	0,03525838	1,45273763	Unpaired t-te	0,006156028	permutation FDR (250 perm	1
3331	Srsf6~Q3TW	Srsf6	ko vs wt	ko	wt	28,3192781	0,71833373	27,977406	0,90343072	1,26740015	0,34187209	0,19406473	0,67911738	0,16805515	Unpaired t-te	0,65995575	permutation FDR (250 perm	1
3332	Srsf7~Q8BL9	Srsf7	ko vs wt	ko	wt	30,8313038	0,30705577	30,7373885	0,77646876	1,06726266	0,09391527	0,05955474	0,88329592	0,05389378	Unpaired t-te	0,901818573	permutation FDR (250 perm	1
3333	Ssb~P32067	Ssb	ko vs wt	ko	wt	29,366483	0,16057522	28,8985302	0,25137476	1,38314529	0,46795271	0,38980694	0,09937245	1,00273402	Unpaired t-te	0,387273572	permutation FDR (250 perm	1
3334	Ssh3~Q8K33	Ssh3	ko vs wt	ko	wt	23,7326656	0,48291267	24,9543427	0,71976243	0,42878396	-1,2216771	-0,77306	0,12055276	0,91882283	Unpaired t-te	0,118916704	permutation FDR (250 perm	1
3335	Ssr4~Q6218	Ssr4	ko vs wt	ko	wt	28,3894831	0,68377562	27,9516507	0,23109654	1,35456764	0,43783244	0,30676397	0,36419436	0,43866678	Unpaired t-te	0,482515866	permutation FDR (250 perm	1
3336	Ssrp1~Q089	Ssrp1	ko vs wt	ko	wt	28,7977696	0,39064537	27,4121903	1,0552169	2,61276846	1,38557928	0,77863712	0,19744019	0,70456444	Unpaired t-te	0,129019913	permutation FDR (250 perm	1
3337	St13~Q99L4	St13	ko vs wt	ko	wt	25,8473235	1,41552797	27,0712774	0,70865278	0,42810781	-1,2239539	-0,6248982	0,26129201	0,58287387	Unpaired t-te	0,174666667	permutation FDR (250 perm	1
3338	St3gal2~Q11	St3gal2	ko vs wt	ko	wt	24,0384599	0,74523843	24,9185036	0,69340272	0,543351	-0,8800436	-0,5326087	0,24132766	0,6173929	Unpaired t-te	0,237012939	permutation FDR (250 perm	1
3339	Stab1~G3X9	Stab1	ko vs wt	ko	wt	29,3106875	0,43612143	28,8591709	1,08641865	1,3674771	0,45151667	0,24967378	0,62366613	0,20504784	Unpaired t-te	0,580159281	permutation FDR (250 perm	1
3340	Stag1~Q9D3	Stag1	ko vs wt	ko	wt	27,4125009	0,6829735	27,2361967	0,50343557	1,12998545	0,17630419	0,11513875	0,75357122	0,12287569	Unpaired t-te	0,796322455	permutation FDR (250 perm	1
3341	Stag2~O356	Stag2	ko vs wt	ko	wt	27,3796004	0,38278211	26,5031507	0,16465562	1,83585196	0,87644972	0,70127601	0,02121652	1,67332585	Unpaired t-te	0,138314008	permutation FDR (250 perm	1
3342	Stam2~O888	Stam2	ko vs wt	ko	wt	24,1958834	0,4314531	24,2420697	1,19590019	0,96849308	-0,0461864	-0,0245469	0,96233744	0,01667262	Unpaired t-te	0,958231457	permutation FDR (250 perm	1
3343	Stambp~Q9C	Stambp	ko vs wt	ko	wt	24,9192333	0,48794557	26,5413445	2,24711619	0,32485972	-1,6221112	-0,6206113	0,41523219	0,38170898	Unpaired t-te	0,20891933	permutation FDR (250 perm	1
3344	Stam~P7029	Stam	ko vs wt	ko	wt	24,9628403	1,36720979	25,227388	1,05076754	0,83245968	-0,2645477	-0,1269398	0,81708761	0,08773138	Unpaired t-te	0,773438341	permutation FDR (250 perm	1
3345	Stat1~Q99K	Stat1	ko vs wt	ko	wt	25,5893243	2,24246487	26,6568111	0,42994711	0,47714945	-1,0674869	-0,4581678	0,47557815	0,32277811	Unpaired t-te	0,312303621	permutation FDR (250 perm	1
3346	Stat3~P4222	Stat3	ko vs wt	ko	wt	29,3274399	0,35028912	28,8001068	0,18483289	1,44126243	0,52733305	0,42499598	0,08261474	1,08294247	Unpaired t-te	0,333677419	permutation FDR (250 perm	1
3347	Stat5b~P422	Stat5b	ko vs wt	ko	wt	26,6996711	1,29828292	26,7944189	0,54451549	0,93643593	-0,0947478	-0,0514187	0,91548902	0,03834686	Unpaired t-te	0,91161588	permutation FDR (250 perm	1
3348	Stat6~P5263	Stat6	ko vs wt	ko	wt	27,1234275	0,39151705	27,5079792	0,90939083	0,766017	-0,3845517	-0,2286808	0,61921873	0,20815592	Unpaired t-te	0,612075672	permutation FDR (250 perm	1
3349	Stau1~A2A5	Stau1	ko vs wt	ko	wt	24,2888598	0,36175887	25,3968922	1,03448761	0,46392632	-1,1080324	-0,6293039	0,26558764	0,57579214	Unpaired t-te	0,198782274	permutation FDR (250 perm	1
3350	Stbd1~Q8C7	Stbd1	ko vs wt	ko	wt	25,2934565	0,58989737	24,7794422	1,52646012	1,42801811	0,51401428	0,2411144	0,68752752	0,16270992	Unpaired t-te	0,593154762	permutation FDR (250 perm	1
3351	Steap3~Q8C	Steap3	ko vs wt	ko	wt	28,1289259	0,35028509	28,2302286	0,15039115	0,93219088	-0,1013027	-0,082461	0,67851416	0,16844108	Unpaired t-te	0,857809524	permutation FDR (250 perm	1
3352	Steap4~Q92	Steap4	ko vs wt	ko	wt	24,9965435	1,83425336	25,4572292	1,93112496	0,72664081	-0,4606857	-0,1688707	0,8025575	0,09552384	Unpaired t-te	0,700139948	permutation FDR (250 perm	1
3353	Stim1~P703	Stim1	ko vs wt	ko	wt	28,2405961	0,2296783	28,0241398	0,26071725	1,16187615	0,21645629	0,17639776	0,39566888	0,40266811	Unpaired t-te	0,687663982	permutation FDR (250 perm	1
3354	Sting1~Q3TB	Sting1	ko vs wt	ko	wt	25,4630417	0,71424301	24,1931899	0,58387631	2,41136788	1,26985177	0,80191368	0,08380587	1,07672555	Unpaired t-te	0,095460927	permutation FDR (250 perm	1
3355	Stip1~Q6086	Stip1	ko vs wt	ko	wt	29,1241724	0,63685182	28,4420777	0,92798497	1,60446772	0,68209476	0,38928399	0,42681623	0,36975907	Unpaired t-te	0,385902293	permutation FDR (250 perm	1
3356	Stk10~O550	Stk10	ko vs wt	ko	wt	26,4669816	0,2773128	26,2638443	0,09195644	1,15119904	0,20313729	0,17320613	0,30634181	0,51379372	Unpaired t-te	0,693149095	permutation FDR (250 perm	1
3357	Stk24~Q99K	Stk24	ko vs wt	ko	wt	29,4884813	0,66850839	29,0756671	0,53658015	1,33128015	0,4128142	0,26784772	0,48117681	0,31769531	Unpaired t-te	0,533353733	permutation FDR (250 perm	1
3358	Stk25~Q9Z2	Stk25	ko vs wt	ko	wt	26,5842151	0,05917787	26,4990974	0,22215157	1,06077424	0,08511765	0,0733294	0,64528078	0,19025127	Unpaired t-te	0,881873913	permutation FDR (250 perm	1
3359	Stk38l~Q7TS	Stk38l	ko vs wt	ko	wt	27,5259697	0,27327754	27,0541687	0,17432509	1,38683967	0,47180101	0,39309535	0,06513425	1,1861906	Unpaired t-te	0,368358992	permutation FDR (250 perm	1
3360	Stk39~Q9Z1	Stk39	ko vs wt	ko	wt	26,9380037	0,2826709	27,3945632	0,2743326	0,72872202	-0,4565595	-0,3642271	0,1399309	0,85408638	Unpaired t-te	0,405356979	permutation FDR (250 perm	1
3361	Stk3~Q9JI10	Stk3	ko vs wt	ko	wt	25,6407242	2,42863109	25,3856457	1,88438629	1,19340071	0,25507855	0,08692978	0,90034316	0,04559193	Unpaired t-te	0,848160219	permutation FDR (250 perm	1
3362	Stoml2~Q99	Stoml2	ko vs wt	ko	wt	26,3275906	0,77365384	24,1662825	2,22199732	4,47320271	2,16130814	0,82071447	0,30081763	0,52169671	Unpaired t-te	0,114655312	permutation FDR (250 perm	1
3363	Stom~P5411	Stom	ko vs wt	ko	wt	29,4778477	0,38948797	29,2796415	0,26555835	1,14727104	0,19820626	0,15329611	0,52872881	0,27676702	Unpaired t-te	0,724837077	permutation FDR (250 perm	1
3364	Ston1~Q8CD	Ston1	ko vs wt	ko	wt	27,7622929	0,23412446	28,2784556	0,12746465	0,69922919	-0,5161627	-0,4440241	0,02584239	1,58766728	Unpaired t-te	0,314064022	permutation FDR (250 perm	1
3365	Ston2~Q8BZ	Ston2	ko vs wt	ko	wt	24,7384712	0,53784125	24,250228	0,32117758	1,40273565	0,48824316	0,35259592	0,26084727	0,58361371	Unpaired t-te	0,416342894	permutation FDR (250 perm	1
3366	Strap~Q9Z12	Strap	ko vs wt	ko	wt	28,0141949	1,07896889	28,1232879	0,67915225	0,92717076	-0,109093	-0,0610631	0,89511966	0,04811891	Unpaired t-te	0,893220394	permutation FDR (250 perm	1
3367	Strbp~Q91W	Strbp	ko vs wt	ko	wt	24,0828774	0,64149305	24,3706867	0,43762594	0,81914496	-0,2878093	-0,1941211	0,57693245	0,23887504	Unpaired t-te	0,652883056	permutation FDR (250 perm	1
3368	Strn3~B2RQ	Strn3	ko vs wt	ko	wt	28,7158062	0,65577168	29,2978508	0,50638453	0,66801636	-0,5820447	-0,3826441	0,31583937	0,50053374	Unpaired t-te	0,381064441	permutation FDR (250 perm	1
3369	Strn~O5510	Strn	ko vs wt	ko	wt	25,0371249	1,49727857	25,4235002	1,21821848	0,76504934	-0,3863753	-0,1740137	0,76482936	0,11643545	Unpaired t-te	0,687893042	permutation FDR (250 perm	1
3370	Stt3a~P4697	Stt3a	ko vs wt	ko	wt	26,0919139	1,14986659	26,2139955	2,25109519	0,91886087	-0,1220817	-0,0448062	0,94847588	0,02297371	Unpaired t-te	0,926085935	permutation FDR (250 perm	1
3371	Stt3b~Q3TD	Stt3b	ko vs wt	ko	wt	28,352598	0,45591147	27,9364466	0,19999119	1,33436327	0,41615148	0,32041151	0,22956619	0,63909208	Unpaired t-te	0,459983492	permutation FDR (250 perm	1
3372	Stub1~Q9W	Stub1	ko vs wt	ko	wt	27,0103204	0,27062463	26,4998946	0,24860484	1,42447061	0,51042585	0,41323642	0,08784823	1,05626699	Unpaired t-te	0,348406941	permutation FDR (250 perm	1
3373	Stx11~Q9D3	Stx11	ko vs wt	ko	wt	24,5262156	1,74307179	23,7645119	0,4920932	1,69549164	0,76170367	0,3688957	0,51713547	0,28639567	Unpaired t-te	0,404830213	permutation FDR (250 perm	1
3374	Stx4~P70452	Stx4	ko vs wt	ko	wt	25,0761973	1,25772292	24,3355299	0,42881256	1,6709486	0,74066736	0,41449592	0,40041743	0,39748703	Unpaired t-te	0,351927916	permutation FDR (250 perm	1
3375	Stx5~Q8K1E	Stx5	ko vs wt	ko	wt	24,8225044	1,81722163	24,4319422	0,28680948	1,3109041	0,39056215	0,18880554	0,7374373	0,1322749	Unpaired t-te	0,672168213	permutation FDR (250 perm	1
3376	Stxbp1~O08	Stxbp1	ko vs wt	ko	wt	28,3993658	0,51316332	28,9136754	0,03631302	0,70012789	-0,5143096	-0,39642	0,18081737	0,74275984	Unpaired t-te	0,380953589	permutation FDR (250 perm	1
3377	Stxbp2~Q64	Stxbp2	ko vs wt	ko	wt	27,7236966	2,30833284	26,4659164	0,17460376	2,39127536	1,25778027	0,53787537	0,41561365	0,3813102	Unpaired t-te	0,24677306	permutation FDR (250 perm	1
3378	Stxbp3~Q60	Stxbp3	ko vs wt	ko	wt	29,1964116	0,36420235	29,080354	0,15631061	1,08376922	0,11605757	0,09377996	0,6485939	0,18802714	Unpaired t-te	0,836978805	permutation FDR (250 perm	1
3379	Stxbp5~Q8K	Stxbp5	ko vs wt	ko	wt	25,2947048	1,88294002	23,8378683	0,35423973	2,74505769	1,45683647	0,68861613	0,27487341	0,56086728	Unpaired t-te	0,152256896	permutation FDR (250 perm	1
3380	Sub1~P1103	Sub1	ko vs wt	ko	wt	26,64262	0,80016919	27,408084	0,93827137	0,58826413	-0,765464	-0,4232695	0,39979429	0,39816342	Unpaired t-te	0,342509724	permutation FDR (250 perm	1
3381	Sucla2~Q9Z2	Sucla2	ko vs wt	ko	wt	30,8104062	0,46287067	30,6813558	0,4302525	1,09357369	0,12905044	0,09185488	0,76423296	0,11677423	Unpaired t-te	0,840039593	permutation FDR (250 perm	1
3382	Suclg1~Q9W	Suclg1	ko vs wt	ko	wt	31,3049352	0,28666644	31,735478	0,2267283	0,74198254	-0,4305429	-0,3499141	0,12236092	0,91235727	Unpaired t-te	0,420270745	permutation FDR (250 perm	1
3383	Suclg2~Q9Z2	Suclg2	ko vs wt	ko	wt	26,0309564	0,8366672	25,9557506	0,08517496	1,05351129	0,07520578	0,05058264	0,88675474	0,05219648	Unpaired t-te	0,914830209	permutation FDR (250 perm	1
3384	Sugt1~Q9CX	Sugt1	ko vs wt	ko	wt	28,1790164	0,11526631	28,1853145	0,29742718	0,99564401	-0,0062981	-0,0051599	0,97942046	0,00903083	Unpaired t-te	0,991147097	permutation FDR (250 perm	1
3385	Sulf1~Q8K00	Sulf1	ko vs wt	ko	wt	26,5643964	1,73150152	28,3437048	0,04489112	0,29132301	-1,7793085	-0,8895712	0,173111	0,76167534	Unpaired t-te	0,082274854	permutation FDR (250 perm	1
3386	Sun1~Q9D66	Sun1	ko vs wt	ko	wt	30,375121	0,20000963	30,6769665	0,32008065	0,81121404	-0,3018455	-0,2406893	0,31932893	0,49576173	Unpaired t-te	0,586248507	permutation FDR (250 perm	1
3387	Sun2~Q8BJS	Sun2	ko vs wt	ko	wt	31,5704493	0,318293	31,9590396	0,09516011	0,76387567	-0,3885902	-0,3249902	0,12259222	0,91153707	Unpaired t-te	0,458465545	permutation FDR (250 perm	1
3388	Supt16~G3X	Supt16	ko vs wt	ko	wt	28,7538988	0,53557074	28,6309666	0,13641521	1,08894588	0,12293226	0,09285557	0,72582179	0,13917	Unpaired t-te	0,840463991	permutation FDR (250 perm	1
3389	Supt5h~O55	Supt5h	ko vs wt	ko	wt	29,1382631	0,31345786	28,7846974	0,59585527	1,27771461	0,35356563	0,24240798	0,50159523	0,2996466	Unpaired t-te	0,586700896	permutation FDR (250 perm	1

3390	Supt6h~Q62	Supt6h	ko vs wt	ko	wt	26,6488314	0,11214641	24,7186318	1,12634135	3,8110793	1,93019963	1,07288677	0,1353879	0,86842014	Unpaired t-te	0,058716289	permutation FDR (250 perm	1
3391	Surf4~Q6431	Surf4	ko vs wt	ko	wt	25,3548693	1,06233539	24,4923979	0,55825401	1,81815024	0,86247142	0,49814434	0,29257205	0,53376716	Unpaired t-te	0,262376665	permutation FDR (250 perm	1
3392	Susd2~Q9DB	Susd2	ko vs wt	ko	wt	27,8875835	0,88663101	28,2684326	0,13232606	0,76798546	-0,3808491	-0,2504963	0,51420824	0,28886097	Unpaired t-te	0,56970241	permutation FDR (250 perm	1
3393	Susd5~G3UV	Susd5	ko vs wt	ko	wt	26,4369296	0,33392898	27,5105619	0,51528567	0,47512126	-1,0736323	-0,7602419	0,07706218	1,11315868	Unpaired t-te	0,124692072	permutation FDR (250 perm	1
3394	Suz12~Q80U	Suz12	ko vs wt	ko	wt	24,4333185	0,5499236	24,6877013	0,44667526	0,8383457	-0,2543828	-0,1756976	0,59550916	0,22511155	Unpaired t-te	0,68555389	permutation FDR (250 perm	1
3395	Svep1~A2AV	Svep1	ko vs wt	ko	wt	23,9085156	1,04039902	24,3074446	0,5078848	0,7584211	-0,398929	-0,2346855	0,59514916	0,22537418	Unpaired t-te	0,584378847	permutation FDR (250 perm	1
3396	Svil~E9Q3Z5	Svil	ko vs wt	ko	wt	31,4839358	0,52885892	32,0390121	0,12141968	0,68062106	-0,5550763	-0,4214132	0,16607256	0,77970213	Unpaired t-te	0,348415107	permutation FDR (250 perm	1
3397	Svs3a~F2Z46	Svs3a	ko vs wt	ko	wt	25,7031065	1,75011349	24,2909079	0,8133952	2,66142445	1,41219861	0,65299237	0,28383378	0,54693592	Unpaired t-te	0,160287922	permutation FDR (250 perm	1
3398	Svs3b~Q8BZ	Svs3b	ko vs wt	ko	wt	29,3726309	2,44322258	26,6091849	2,77622095	6,79016172	2,76344594	0,80865665	0,31817683	0,49733145	Unpaired t-te	0,09796488	permutation FDR (250 perm	1
3399	Svs4~P18419	Svs4	ko vs wt	ko	wt	25,1078217	0,97541003	24,4588235	0,94549622	1,56807901	0,64899825	0,34629098	0,49570474	0,30477693	Unpaired t-te	0,427783609	permutation FDR (250 perm	1
3400	Svs5~P30933	Svs5	ko vs wt	ko	wt	24,9581212	2,00012925	24,5634085	0,94077962	1,31468086	0,39471263	0,16920986	0,78008213	0,10785967	Unpaired t-te	0,697819747	permutation FDR (250 perm	1
3401	Swap70~Q6A	Swap70	ko vs wt	ko	wt	29,066648	0,57251785	28,6676295	0,48126521	1,31861053	0,3990185	0,27062885	0,44064951	0,35590671	Unpaired t-te	0,529437155	permutation FDR (250 perm	1
3402	Syde2~E9PU	Syde2	ko vs wt	ko	wt	24,5221403	0,40317138	24,6990193	0,62560716	0,88461464	-0,176879	-0,1179292	0,74614303	0,12717792	Unpaired t-te	0,797772943	permutation FDR (250 perm	1
3403	Syk~P48025	Syk	ko vs wt	ko	wt	29,6945365	1,56161526	27,9792332	0,70470095	3,28365662	1,71530327	0,84491991	0,16345166	0,78661067	Unpaired t-te	0,083078565	permutation FDR (250 perm	1
3404	Sympk~Q80X	Sympk	ko vs wt	ko	wt	24,5794976	0,2299248	24,8657773	1,39284649	0,82001391	-0,2862797	-0,1435852	0,79955757	0,09715026	Unpaired t-te	0,759787342	permutation FDR (250 perm	1
3405	Syncrip~Q7T	Syncrip	ko vs wt	ko	wt	31,7559286	0,17091027	31,8062679	0,07562034	0,9657092	-0,0503393	-0,0452597	0,67477599	0,17084038	Unpaired t-te	0,922306788	permutation FDR (250 perm	1
3406	Syne1~Q6ZW	Syne1	ko vs wt	ko	wt	31,8631576	0,32427215	32,3725324	0,17355854	0,70252684	-0,5093747	-0,4162044	0,07412529	1,13003359	Unpaired t-te	0,342914052	permutation FDR (250 perm	1
3407	Syne2~E9QP	Syne2	ko vs wt	ko	wt	30,2480124	0,18581707	30,8346703	0,4999396	0,66588368	-0,5866579	-0,4283954	0,23365611	0,63142285	Unpaired t-te	0,358418416	permutation FDR (250 perm	1
3408	Syne3~Q4FZ	Syne3	ko vs wt	ko	wt	27,6751719	0,21079013	27,2232184	0,32228043	1,36789118	0,45195346	0,35916442	0,17461153	0,75792709	Unpaired t-te	0,422877763	permutation FDR (250 perm	1
3409	Synj1~Q8CH	Synj1	ko vs wt	ko	wt	26,8184504	0,23666786	26,7209957	0,03652012	1,06988425	0,09745473	0,08555722	0,53080996	0,27506094	Unpaired t-te	0,855206793	permutation FDR (250 perm	1
3410	Synj2~Q9D2	Synj2	ko vs wt	ko	wt	24,0308268	1,27993417	26,2722266	0,22102037	0,21148103	-2,2413998	-1,276921	0,05323743	1,2737829	Unpaired t-te	0,020647887	permutation FDR (250 perm	1
3411	Synm~Q70IV	Synm	ko vs wt	ko	wt	29,838525	0,17950177	30,3341945	0,37911675	0,70923243	-0,4956696	-0,3850127	0,19679982	0,7059753	Unpaired t-te	0,399495887	permutation FDR (250 perm	1
3412	Synpo2~E9Q	Synpo2	ko vs wt	ko	wt	32,4796883	0,67724955	33,3609221	0,3977821	0,54290295	-0,8812338	-0,5947578	0,12768778	0,89385066	Unpaired t-te	0,191209627	permutation FDR (250 perm	1
3413	Synpo~E9Q3	Synpo	ko vs wt	ko	wt	30,5733731	0,62240391	30,8076814	0,32500402	0,85009247	-0,2343083	-0,1642485	0,6075549	0,21641447	Unpaired t-te	0,705811623	permutation FDR (250 perm	1
3414	Synpo~Q8CC	Synpo	ko vs wt	ko	wt	24,2525826	0,64733571	26,2210852	0,18371188	0,2555181	-1,9685026	-1,4104337	0,00939416	2,02714209	Unpaired t-te	0,012519263	permutation FDR (250 perm	1
3415	Tacc1~Q6Y6	Tacc1	ko vs wt	ko	wt	24,7856645	1,13511722	23,7697384	0,34256267	2,0222006	1,01592612	0,59806347	0,22377655	0,65018542	Unpaired t-te	0,197060185	permutation FDR (250 perm	1
3416	Tacc2~E9Q8	Tacc2	ko vs wt	ko	wt	24,8820284	1,29127078	27,8489477	0,02911079	0,12789934	-2,9669193	-1,6994615	0,02833232	1,54771792	Unpaired t-te	0,007311371	permutation FDR (250 perm	1
3417	Tada1~Q99L	Tada1	ko vs wt	ko	wt	24,9242832	0,99279123	23,9321597	1,05968325	1,9891107	0,99212357	0,51050842	0,35106369	0,45461408	Unpaired t-te	0,257445451	permutation FDR (250 perm	1
3418	Taf15~Q8BQ	Taf15	ko vs wt	ko	wt	24,4668653	0,80880615	25,8010971	1,64764676	0,39660318	-1,3342318	-0,5916352	0,37497275	0,4260003	Unpaired t-te	0,214666667	permutation FDR (250 perm	1
3419	Taf5l~Q91W	Taf5l	ko vs wt	ko	wt	23,7421545	0,74492309	23,5818461	0,49497644	1,11752603	0,16030844	0,10312536	0,7841053	0,10562561	Unpaired t-te	0,817382019	permutation FDR (250 perm	1
3420	Tagln2~Q9W	Tagln2	ko vs wt	ko	wt	32,2322068	0,24021561	32,2103332	0,06061345	1,01527719	0,02187366	0,01910096	0,88838591	0,05139834	Unpaired t-te	0,967685924	permutation FDR (250 perm	1
3421	Tagln~P3780	Tagln	ko vs wt	ko	wt	34,1782791	0,49432037	34,2579247	0,24752707	0,94629009	-0,0796456	-0,0596689	0,82200943	0,0851232	Unpaired t-te	0,895975202	permutation FDR (250 perm	1
3422	Taldo1~Q93	Taldo1	ko vs wt	ko	wt	30,1447369	0,4305905	29,3349804	0,10532036	1,7529155	0,80975645	0,64291046	0,04226202	1,37404971	Unpaired t-te	0,1732728	permutation FDR (250 perm	1
3423	Tanc1~Q0VG	Tanc1	ko vs wt	ko	wt	24,5674784	0,50454158	24,5602917	1,07650177	1,00499392	0,00718678	0,00395958	0,99359823	0,00278919	Unpaired t-te	0,992983607	permutation FDR (250 perm	1
3424	Taok1~Q5F2	Taok1	ko vs wt	ko	wt	27,11568	0,38729426	27,3686653	0,37326218	0,8391582	-0,2529853	-0,1879645	0,50168505	0,29956884	Unpaired t-te	0,666507844	permutation FDR (250 perm	1
3425	Taok3~Q8BY	Taok3	ko vs wt	ko	wt	27,2697878	0,75527419	26,7857637	0,7754683	1,39863951	0,48402416	0,28462185	0,52586996	0,27912164	Unpaired t-te	0,5121966	permutation FDR (250 perm	1
3426	Tap1~P21958	Tap1	ko vs wt	ko	wt	25,3617426	1,16878873	25,5776537	0,21719933	0,86100223	-0,2159111	-0,1276027	0,77371158	0,1114209	Unpaired t-te	0,77763672	permutation FDR (250 perm	1
3427	Tardbp~Q92	Tardbp	ko vs wt	ko	wt	28,9979655	0,33339688	28,8938654	0,70591669	1,07482371	0,10410005	0,06781823	0,85990236	0,06555086	Unpaired t-te	0,887500135	permutation FDR (250 perm	1
3428	Tars1~Q9D0	Tars1	ko vs wt	ko	wt	29,5587939	0,14100455	29,7265719	0,3977677	0,89021273	-0,167778	-0,1297779	0,61699804	0,20971621	Unpaired t-te	0,780944072	permutation FDR (250 perm	1
3429	Tars3~Q8BL	Tars3	ko vs wt	ko	wt	25,205112	0,970848	27,4071278	0,10529286	0,21733376	-2,2020158	-1,4066411	0,02821811	1,54947203	Unpaired t-te	0,014756542	permutation FDR (250 perm	1
3430	Tax1bp3~Q9	Tax1bp3	ko vs wt	ko	wt	24,384873	1,30065642	24,7983472	0,45009776	0,75081317	-0,4134741	-0,2277347	0,63896848	0,19452056	Unpaired t-te	0,599302643	permutation FDR (250 perm	1
3431	Tbc1d10b~Q	Tbc1d10b	ko vs wt	ko	wt	26,4768964	0,09707629	26,900194	0,2607844	0,74571815	-0,4232976	-0,3548976	0,13894181	0,85716704	Unpaired t-te	0,438815291	permutation FDR (250 perm	1
3432	Tbc1d15~Q9	Tbc1d15	ko vs wt	ko	wt	26,9159558	0,20720562	27,4645481	0,48466234	0,68368692	-0,5485923	-0,4024924	0,24549671	0,60995433	Unpaired t-te	0,383225075	permutation FDR (250 perm	1
3433	Tbc1d1~Q60	Tbc1d1	ko vs wt	ko	wt	26,9490092	0,43889484	26,739799	0,16857358	1,15605515	0,20921022	0,16344136	0,49495087	0,30543791	Unpaired t-te	0,709720824	permutation FDR (250 perm	1
3434	Tbc1d2b~Q3	Tbc1d2b	ko vs wt	ko	wt	26,3970271	0,3208482	26,3529991	0,48441099	1,03098832	0,04402798	0,03168822	0,91678301	0,03773344	Unpaired t-te	0,946307489	permutation FDR (250 perm	1
3435	Tbc1d5~Q80	Tbc1d5	ko vs wt	ko	wt	24,7473265	0,76115025	25,4613227	0,16366762	0,60962918	-0,7139961	-0,49091	0,20337781	0,69169644	Unpaired t-te	0,279167376	permutation FDR (250 perm	1
3436	Tbc1d8b~A3	Tbc1d8b	ko vs wt	ko	wt	25,2684603	0,59244546	26,1135978	0,24482722	0,55665772	-0,8451376	-0,6109296	0,0873168	1,05890219	Unpaired t-te	0,184394366	permutation FDR (250 perm	1
3437	Tbc1d9b~Q5	Tbc1d9b	ko vs wt	ko	wt	26,8653462	0,68331638	26,4739552	0,36810512	1,31165743	0,39139098	0,26577457	0,4467258	0,34995896	Unpaired t-te	0,536700489	permutation FDR (250 perm	1
3438	Tbcd~Q8BYA	Tbcd	ko vs wt	ko	wt	24,8866087	1,65614844	24,3518129	0,10064116	1,4487371	0,53479582	0,27301906	0,61552944	0,21075117	Unpaired t-te	0,538988084	permutation FDR (250 perm	1
3439	Tbcel~Q8C5	Tbcel	ko vs wt	ko	wt	24,7063681	1,15281586	24,5143046	0,74651576	1,14239654	0,19206352	0,1038466	0,83008843	0,08087564	Unpaired t-te	0,816084141	permutation FDR (250 perm	1
3440	Tbce~E0CZ81	Tbce	ko vs wt	ko	wt	26,4674572	2,96429994	26,5560084	3,9117913	0,94046673	-0,0885512	-0,0208223	0,97976745	0,008877	Unpaired t-te	0,964076721	permutation FDR (250 perm	1
3441	Tbl1x~Q9QX	Tbl1x	ko vs wt	ko	wt	26,9606621	0,14268109	27,1915159	0,13504405	0,85213046	-0,2308538	-0,2050006	0,1339541	0,87304399	Unpaired t-te	0,636536385	permutation FDR (250 perm	1
3442	Tbl2~Q9R09	Tbl2	ko vs wt	ko	wt	28,4372806	0,90439329	27,9114302	0,43399949	1,43978202	0,5258504	0,32749863	0,42817602	0,36837766	Unpaired t-te	0,449815394	permutation FDR (250 perm	1
3443	Tbl3~Q8C4J7	Tbl3	ko vs wt	ko	wt	24,8146182	1,1018307	25,004553	0,54088532	0,87664539	-0,1899347	-0,1090161	0,80897009	0,09206754	Unpaired t-te	0,807341477	permutation FDR (250 perm	1
3444	Tbxas1~P364	Tbxas1	ko vs wt	ko	wt	24,8392732	1,51604939	24,2085383	0,42579027	1,54835356	0,63073494	0,32754796	0,53611578	0,27074141	Unpaired t-te	0,4558345	permutation FDR (250 perm	1
3445	Tcaf2~Q921	Tcaf2	ko vs wt	ko	wt	24,2681393	0,66411898	25,1172906	1,3469749	0,55511119	-0,8491513	-0,4189746	0,47596806	0,32242219	Unpaired t-te	0,362631543	permutation FDR (250 perm	1
3446	Tcerg1~Q8C	Tcerg1	ko vs wt	ko	wt	27,4300315	0,55741756	25,3438856	1,46271777	4,24612216	2,08614588	1,00140949	0,17273165	0,76262807	Unpaired t-te	0,064721003	permutation FDR (250 perm	1
3447	Tcf25~Q8R3	Tcf25	ko vs wt	ko	wt	25,5956808	0,20439978	24,9365706	0,37397069	1,57910847	0,65911028	0,51110673	0,11355198	0,94480528	Unpaired t-te	0,270839957	permutation FDR (250 perm	1
3448	Tcirg1~Q9JH	Tcirg1	ko vs wt	ko	wt	27,9304692	0,64447598	25,3130863	1,25647008	6,13635895	2,61738288	1,33320368	0,08136158	1,08958061	Unpaired t-te	0,020291578	permutation FDR (250 perm	1
3449	Tcn2~O8896	Tcn2	ko vs wt	ko	wt	28,0962329	0,37145892	27,6817502	0,21370672	1,33282069	0,4144827	0,32834131	0,17609903	0,75424303	Unpaired t-te	0,446889102	permutation FDR (250 perm	1
3450	Tcof1~O087	Tcof1	ko vs wt	ko	wt	24,3056913	1,38463011	23,9313937	0,81134564	1,29620836	0,37429765	0,18866042	0,71950312	0,14296732	Unpaired t-te	0,663198371	permutation FDR (250 perm	1
3451	Tcp1~P11983	Tcp1	ko vs wt	ko	wt	32,5649273	0,32814358	32,4047098	0,28061408	1,11745559	0,16021749	0,12572542	0,58610095	0,23202757	Unpaired t-te	0,776447059	permutation FDR (250 perm	1
3452	Tdrd3~Q91W	Tdrd3	ko vs wt	ko	wt	23,8306289	0,49702862	24,9123299	0,46624109	0,47247144	-1,081701	-0,7527092	0,06205324	1,20723554	Unpaired t-te	0,11645658	permutation FDR (250 perm	1
3453	Tdrd7~Q8K1	Tdrd7	ko vs wt	ko	wt	23,7676386	1,01380787	25,2114246	0,88005966	0,36760135	-1,443786	-0,7786087	0,15597143	0,80695495	Unpaired t-te	0,104875686	permutation FDR (250 perm	1

3454	Tecr~Q9CY27	Tecr	ko vs wt	ko	wt	29,7851862	0,3380566	30,0818519	0,29336689	0,81413181	-0,2966657	-0,2308994	0,34851182	0,45778248	Unpaired t-te	0,590635252	permutation FDR (250 perm	1
3455	Tep1~P97499	Tep1	ko vs wt	ko	wt	24,4145697	0,27419478	25,2627753	0,56873267	0,55547518	-0,8482057	-0,5922433	0,15743533	0,80289779	Unpaired t-te	0,214686297	permutation FDR (250 perm	1
3456	Tes~Q921W7	Tes	ko vs wt	ko	wt	31,4017904	0,4952924	31,9310474	0,42699604	0,6929115	-0,529257	-0,3738077	0,26262884	0,58065758	Unpaired t-te	0,392788602	permutation FDR (250 perm	1
3457	Tfg~Q9Z1A1	Tfg	ko vs wt	ko	wt	28,7331672	0,31735866	27,8336984	0,37962327	1,86537903	0,8994688	0,67884116	0,05415844	1,26633382	Unpaired t-te	0,152705882	permutation FDR (250 perm	1
3458	Tfrc~Q62351	Tfrc	ko vs wt	ko	wt	27,5898882	0,73331677	27,2378566	0,27472053	1,2763567	0,35203157	0,24016042	0,49070439	0,30918006	Unpaired t-te	0,578977804	permutation FDR (250 perm	1
3459	Tf~Q921I1	Tf	ko vs wt	ko	wt	30,0717106	0,73875632	29,9390005	0,44275473	1,09635123	0,13271006	0,08679022	0,81202879	0,09042857	Unpaired t-te	0,848435665	permutation FDR (250 perm	1
3460	Tgfb1i1~Q62	Tgfb1i1	ko vs wt	ko	wt	32,1934671	0,68520684	32,6678922	0,37715885	0,71975353	-0,4744251	-0,3211862	0,3670653	0,43525667	Unpaired t-te	0,456722876	permutation FDR (250 perm	1
3461	Tgfb1~P042C	Tgfb1	ko vs wt	ko	wt	29,6739346	0,71105483	29,0597226	0,75469704	1,53072173	0,61421204	0,36706892	0,41196883	0,38513564	Unpaired t-te	0,403526938	permutation FDR (250 perm	1
3462	Tgfb2~A0A0	Tgfb2	ko vs wt	ko	wt	28,1055394	0,08538142	28,2616545	0,12551458	0,89743844	-0,1561151	-0,1417266	0,2156046	0,66634198	Unpaired t-te	0,752051802	permutation FDR (250 perm	1
3463	Tgfb3~P1712	Tgfb3	ko vs wt	ko	wt	26,0088418	0,11273473	24,8982948	0,59608489	2,15927499	1,11054699	0,77851639	0,11564204	0,93688424	Unpaired t-te	0,135255655	permutation FDR (250 perm	1
3464	Tgfbi~P8219	Tgfbi	ko vs wt	ko	wt	34,8875399	0,24678311	34,7093611	0,73802679	1,13145469	0,17817882	0,11562813	0,76948814	0,11379807	Unpaired t-te	0,80659072	permutation FDR (250 perm	1
3465	Tgfbr2~Q623	Tgfbr2	ko vs wt	ko	wt	24,5275249	0,65145223	24,38429	0,44405447	1,10437867	0,14323492	0,09613375	0,78177201	0,10691988	Unpaired t-te	0,83128536	permutation FDR (250 perm	1
3466	Tgm2~P2198	Tgm2	ko vs wt	ko	wt	35,2656749	0,30762396	35,7477595	0,31372124	0,71594239	-0,4820846	-0,3754045	0,16117703	0,79269685	Unpaired t-te	0,393607415	permutation FDR (250 perm	1
3467	Thada~A8C7	Thada	ko vs wt	ko	wt	24,3907118	0,842381	22,7604279	1,29511829	3,09573912	1,6302839	0,80036758	0,21043733	0,67687722	Unpaired t-te	0,108829025	permutation FDR (250 perm	1
3468	Thap11~Q9J	Thap11	ko vs wt	ko	wt	24,2953407	1,37158265	24,256309	0,8988046	1,02742402	0,03903171	0,01936689	0,9708246	0,01285923	Unpaired t-te	0,966349356	permutation FDR (250 perm	1
3469	Thbd~P1530	Thbd	ko vs wt	ko	wt	24,7869618	1,29419485	26,8747907	0,22087372	0,23523442	-2,087829	-1,1840115	0,06537028	1,18461968	Unpaired t-te	0,028871158	permutation FDR (250 perm	1
3470	Thbs1~Q80Y	Thbs1	ko vs wt	ko	wt	33,8106514	1,00751208	31,7362656	0,98273449	4,21165066	2,07438577	1,08821665	0,07850167	1,10512113	Unpaired t-te	0,034530663	permutation FDR (250 perm	1
3471	Thbs2~Q033	Thbs2	ko vs wt	ko	wt	28,0177349	1,94275901	25,5090857	0,12580043	5,69086988	2,50864919	1,18044401	0,11093691	0,95492392	Unpaired t-te	0,030881748	permutation FDR (250 perm	1
3472	Thbs3~Q058	Thbs3	ko vs wt	ko	wt	30,5206554	0,47272624	30,1849178	0,64019879	1,26202252	0,33573766	0,21963745	0,56533892	0,24769112	Unpaired t-te	0,617164945	permutation FDR (250 perm	1
3473	Thbs4~Q9Z1	Thbs4	ko vs wt	ko	wt	25,062915	1,17065864	24,1675125	0,13442857	1,8601288	0,89540252	0,53217575	0,27778869	0,55628544	Unpaired t-te	0,249906637	permutation FDR (250 perm	1
3474	Themis2~Q9	Themis2	ko vs wt	ko	wt	24,0595223	0,53835195	24,6365674	0,69931181	0,67033536	-0,5770451	-0,3642823	0,38601729	0,41339324	Unpaired t-te	0,411647059	permutation FDR (250 perm	1
3475	Thoc1~Q8R3	Thoc1	ko vs wt	ko	wt	25,443301	0,64074243	23,5909533	0,84632	3,61087305	1,8523477	1,08734727	0,06691916	1,1744495	Unpaired t-te	0,038427021	permutation FDR (250 perm	1
3476	Thoc2~B1AZ	Thoc2	ko vs wt	ko	wt	27,4908868	0,46276561	27,2840779	0,4735669	1,15413255	0,20680893	0,14478478	0,65326124	0,18491311	Unpaired t-te	0,742381464	permutation FDR (250 perm	1
3477	Thoc5~Q8BK	Thoc5	ko vs wt	ko	wt	23,8804459	0,31630509	26,4886067	0,08155753	0,16400812	-2,6081608	-2,1889572	0,00032933	3,48236615	Unpaired t-te	0,003009901	permutation FDR (250 perm	1
3478	Thrap3~Q56	Thrap3	ko vs wt	ko	wt	29,4638762	0,33531253	29,0335791	0,47024355	1,34751105	0,43029711	0,31073698	0,33753502	0,47168116	Unpaired t-te	0,482365064	permutation FDR (250 perm	1
3479	Thrsp~Q622	Thrsp	ko vs wt	ko	wt	25,9465676	1,94083465	27,9925509	0,1289608	0,24215735	-2,0459833	-0,9631575	0,16514048	0,78214646	Unpaired t-te	0,063855194	permutation FDR (250 perm	1
3480	Thsd4~Q3UT	Thsd4	ko vs wt	ko	wt	31,7862561	0,63023921	33,0182295	0,43420174	0,42573469	-1,2319735	-0,8346169	0,04902403	1,30959098	Unpaired t-te	0,083932269	permutation FDR (250 perm	1
3481	Thumpd1~Q	Thumpd1	ko vs wt	ko	wt	25,5742626	1,99745521	24,0299505	0,37052295	2,91664955	1,54431205	0,70755021	0,2750795	0,56054177	Unpaired t-te	0,144309623	permutation FDR (250 perm	1
3482	Thy1~P01831	Thy1	ko vs wt	ko	wt	25,8601067	2,00164148	24,8139059	0,10872539	2,06508449	1,04620081	0,48475559	0,43242027	0,36409396	Unpaired t-te	0,290147235	permutation FDR (250 perm	1
3483	Thyn1~Q91Y	Thyn1	ko vs wt	ko	wt	27,1281314	0,05670629	27,2429194	0,11547499	0,923518	-0,114788	-0,1055064	0,29373222	0,53204842	Unpaired t-te	0,821342912	permutation FDR (250 perm	1
3484	Tial1~P7031	Tial1	ko vs wt	ko	wt	27,1537841	0,178289	26,8993053	0,13358354	1,19290463	0,25447871	0,2232844	0,12895183	0,88957248	Unpaired t-te	0,60180427	permutation FDR (250 perm	1
3485	Tifa~Q793I8	Tifa	ko vs wt	ko	wt	24,8387123	1,10530393	24,1565949	0,70320351	1,60449292	0,68211742	0,37706893	0,4376774	0,35884588	Unpaired t-te	0,386700332	permutation FDR (250 perm	1
3486	Timm44~O3	Timm44	ko vs wt	ko	wt	27,9725156	0,34997136	27,0827385	1,17850373	1,85288986	0,88977713	0,47902527	0,39840248	0,39967797	Unpaired t-te	0,311630533	permutation FDR (250 perm	1
3487	Timm50~Q9	Timm50	ko vs wt	ko	wt	25,0703341	1,35583229	26,9510744	0,27407263	0,27154433	-1,8807403	-1,0411405	0,09256119	1,03357106	Unpaired t-te	0,046624351	permutation FDR (250 perm	1
3488	Timp1~P120	Timp1	ko vs wt	ko	wt	29,3979892	0,42670127	26,8751922	0,08227875	5,74695214	2,52279704	2,01319206	0,0013641	2,8651548	Unpaired t-te	0,003757764	permutation FDR (250 perm	1
3489	Timp2~Q6PI	Timp2	ko vs wt	ko	wt	27,7518048	0,38458139	24,7793822	1,42458178	7,84853051	2,97242256	1,46315757	0,09182316	1,03704774	Unpaired t-te	0,017955817	permutation FDR (250 perm	1
3490	Timp3~P398	Timp3	ko vs wt	ko	wt	32,3371894	0,53912363	31,5405718	0,40638093	1,73702395	0,79661765	0,55957016	0,11967742	0,92198779	Unpaired t-te	0,214226757	permutation FDR (250 perm	1
3491	Tinagl1~Q99	Tinagl1	ko vs wt	ko	wt	33,9017345	0,75464227	34,3818098	0,2607258	0,71694021	-0,4800753	-0,325896	0,36789121	0,43428059	Unpaired t-te	0,455689271	permutation FDR (250 perm	1
3492	Tjp1~B9EHJ3	Tjp1	ko vs wt	ko	wt	29,5186383	0,13438698	30,0917239	0,53275683	0,6721776	-0,5730856	-0,4138929	0,26524932	0,57634572	Unpaired t-te	0,378409639	permutation FDR (250 perm	1
3493	Tjp2~Q9Z0U	Tjp2	ko vs wt	ko	wt	29,5185429	0,51549117	29,9870999	0,14802035	0,7226871	-0,4685569	-0,3561852	0,21719075	0,66315867	Unpaired t-te	0,42026684	permutation FDR (250 perm	1
3494	Tkt~P40142	Tkt	ko vs wt	ko	wt	31,6287575	0,28870573	31,653746	0,13480914	0,98282842	-0,0249885	-0,0209632	0,90200947	0,0447889	Unpaired t-te	0,963080158	permutation FDR (250 perm	1
3495	Tln1~P26039	Tln1	ko vs wt	ko	wt	36,3945733	0,09461478	36,7928549	0,10630184	0,75876151	-0,3982816	-0,3644199	0,01322096	1,87873715	Unpaired t-te	0,408325353	permutation FDR (250 perm	1
3496	Tln2~E9PUM	Tln2	ko vs wt	ko	wt	30,6163933	0,42164419	30,6489846	0,46387016	0,97766267	-0,0325913	-0,0231395	0,9402371	0,02676261	Unpaired t-te	0,959241452	permutation FDR (250 perm	1
3497	Tm9sf2~P58	Tm9sf2	ko vs wt	ko	wt	27,8495117	0,68289472	28,619281	0,26323527	0,58651125	-0,7697693	-0,5360516	0,14921619	0,82618405	Unpaired t-te	0,236593195	permutation FDR (250 perm	1
3498	Tm9sf3~Q9E	Tm9sf3	ko vs wt	ko	wt	28,2245346	0,64148697	28,0642236	0,36915415	1,11752804	0,16031103	0,11032294	0,73818404	0,13183535	Unpaired t-te	0,80525584	permutation FDR (250 perm	1
3499	Tm9sf4~Q8B	Tm9sf4	ko vs wt	ko	wt	25,3028352	1,22659906	24,4071332	0,65136265	1,86051488	0,89570193	0,48553343	0,33953322	0,46911773	Unpaired t-te	0,273081312	permutation FDR (250 perm	1
3500	Tmco3~Q8B	Tmco3	ko vs wt	ko	wt	24,3643909	0,85660339	24,1898864	0,37539951	1,12857672	0,17450449	0,11176907	0,76997792	0,11352173	Unpaired t-te	0,803838575	permutation FDR (250 perm	1
3501	Tmed10~Q9	Tmed10	ko vs wt	ko	wt	30,6405365	0,56574874	30,4026399	0,25669204	1,17927213	0,23789668	0,17318215	0,55534885	0,25543412	Unpaired t-te	0,691029327	permutation FDR (250 perm	1
3502	Tmed3~Q78	Tmed3	ko vs wt	ko	wt	27,7299724	0,58441692	28,2585549	0,63247715	0,69323554	-0,5285825	-0,3387843	0,39823097	0,39986496	Unpaired t-te	0,438273049	permutation FDR (250 perm	1
3503	Tmed7~D3Y	Tmed7	ko vs wt	ko	wt	28,3756963	0,6712011	28,2361449	0,09565091	1,10156256	0,13955143	0,10015344	0,74500102	0,12784313	Unpaired t-te	0,828531715	permutation FDR (250 perm	1
3504	Tmed9~Q99	Tmed9	ko vs wt	ko	wt	25,3142971	1,30707529	25,0583393	1,79565383	1,19412826	0,25595781	0,10333183	0,87228856	0,05933983	Unpaired t-te	0,82161039	permutation FDR (250 perm	1
3505	Tmem11~Q8	Tmem11	ko vs wt	ko	wt	25,332322	2,04511728	27,4306523	0,14626781	0,23352836	-2,0983303	-0,9602155	0,1734206	0,76089932	Unpaired t-te	0,06456874	permutation FDR (250 perm	1
3506	Tmem214~C	Tmem214	ko vs wt	ko	wt	25,9681222	1,55421286	27,0566515	0,36970886	0,47024049	-1,0885293	-0,5626548	0,31748282	0,49827977	Unpaired t-te	0,223108948	permutation FDR (250 perm	1
3507	Tmem263~C	Tmem263	ko vs wt	ko	wt	23,971647	0,55734122	24,6706663	0,61083705	0,61599081	-0,6990193	-0,4543178	0,2639356	0,57850203	Unpaired t-te	0,309114136	permutation FDR (250 perm	1
3508	Tmem43~Q9	Tmem43	ko vs wt	ko	wt	30,8167338	0,18308707	30,7311655	0,38850518	1,06110566	0,08556832	0,06610912	0,79290402	0,10077938	Unpaired t-te	0,889937331	permutation FDR (250 perm	1
3509	Tmem59~Q9	Tmem59	ko vs wt	ko	wt	24,5467649	0,82672455	23,4436393	0,80268411	2,14819606	1,10312567	0,63339665	0,20517644	0,6878725	Unpaired t-te	0,171854575	permutation FDR (250 perm	1
3510	Tmlhe~Q91Z	Tmlhe	ko vs wt	ko	wt	24,9947131	1,18908908	24,3160087	0,33322375	1,6007017	0,67870447	0,39326252	0,40706585	0,39033533	Unpaired t-te	0,377497992	permutation FDR (250 perm	1
3511	Tmod3~Q9J	Tmod3	ko vs wt	ko	wt	27,1586847	0,77532214	26,809835	1,0220212	1,2735448	0,34884971	0,18855911	0,70547288	0,15151967	Unpaired t-te	0,670422519	permutation FDR (250 perm	1
3512	Tmpo~Q610	Tmpo	ko vs wt	ko	wt	28,0132529	0,47774674	27,5339989	0,91813018	1,39402259	0,47925394	0,28102458	0,55001665	0,25962416	Unpaired t-te	0,530634804	permutation FDR (250 perm	1
3513	Tmpo~Q610	Tmpo	ko vs wt	ko	wt	26,1065575	2,10966433	29,0160428	0,39223474	0,13309374	-2,9094853	-1,2935681	0,09412901	1,02627649	Unpaired t-te	0,019454415	permutation FDR (250 perm	1
3514	Tmsb4x~P20	Tmsb4x	ko vs wt	ko	wt	27,8076215	0,55832481	28,6297141	1,08442999	0,5656209	-0,8220927	-0,4487878	0,40276379	0,39494958	Unpaired t-te	0,329789691	permutation FDR (250 perm	1
3515	Tmtc3~Q8B	Tmtc3	ko vs wt	ko	wt	27,1275733	0,80766061	26,621427	0,73233505	1,42025145	0,50614638	0,29828638	0,50317791	0,29827843	Unpaired t-te	0,49033875	permutation FDR (250 perm	1
3516	Tmx1~Q8VB	Tmx1	ko vs wt	ko	wt	25,1390474	1,36717349	26,3664797	0,07744257	0,42707689	-1,2274323	-0,6852435	0,21777794	0,66198612	Unpaired t-te	0,157006936	permutation FDR (250 perm	1
3517	Tmx3~Q8BX	Tmx3	ko vs wt	ko	wt	27,2542769	0,37379121	27,3950247	0,9060206	0,90704888	-0,1407478	-0,0839771	0,85108804	0,07002551	Unpaired t-te	0,861678972	permutation FDR (250 perm	1

3518	Tmx4~Q8C0l	Tmx4	ko vs wt	ko	wt	26,9375183	0,44923501	27,2181525	0,08913236	0,82322907	-0,2806342	-0,2215101	0,36297604	0,44012204	Unpaired t-te	0,614865583	permutation FDR (250 perm	1
3519	Tnc~Q80YX1	Tnc	ko vs wt	ko	wt	35,3626823	0,41114295	34,5686799	1,3566204	1,73387791	0,79400232	0,3993557	0,49787272	0,30288167	Unpaired t-te	0,391908549	permutation FDR (250 perm	1
3520	Tnc~Q80YX1·	Tnc	ko vs wt	ko	wt	25,9830014	1,51924937	25,3238855	1,37079813	1,57911467	0,65911594	0,285671	0,63746189	0,19554577	Unpaired t-te	0,508514091	permutation FDR (250 perm	1
3521	Tnfaip2~D3Z	Tnfaip2	ko vs wt	ko	wt	24,8870143	0,73799649	23,6338302	0,30629916	2,38366926	1,25318407	0,84790868	0,05432536	1,26499741	Unpaired t-te	0,083154219	permutation FDR (250 perm	1
3522	Tnfaip8l2~Q	Tnfaip8l2	ko vs wt	ko	wt	24,849032	1,28088811	23,6584117	0,54519943	2,28250856	1,19062027	0,64920237	0,22079792	0,65600503	Unpaired t-te	0,163226224	permutation FDR (250 perm	1
3523	Tnfrsf11b~O	Tnfrsf11b	ko vs wt	ko	wt	30,4306779	0,57110213	29,6951315	0,9138053	1,66502796	0,7355464	0,42629905	0,38438774	0,41523048	Unpaired t-te	0,348411024	permutation FDR (250 perm	1
3524	Tnik~P83510	Tnik	ko vs wt	ko	wt	24,4187702	0,91726478	24,0721403	0,96149282	1,27158675	0,34662989	0,18618038	0,70740233	0,15033351	Unpaired t-te	0,670708442	permutation FDR (250 perm	1
3525	Tnip1~Q9WL	Tnip1	ko vs wt	ko	wt	24,3434907	0,45583428	25,1477695	0,45471812	0,57264829	-0,8042788	-0,5681914	0,12013833	0,92031841	Unpaired t-te	0,212240727	permutation FDR (250 perm	1
3526	Tnks1bp1~P5	Tnks1bp1	ko vs wt	ko	wt	27,2564485	0,19138071	26,9748673	0,39026971	1,21552635	0,28158117	0,21705823	0,42171992	0,37497589	Unpaired t-te	0,629744706	permutation FDR (250 perm	1
3527	Tnmd~Q9EP(	Tnmd	ko vs wt	ko	wt	25,4995667	1,4659716	27,3988959	0,24381006	0,26806797	-1,8993293	-1,0190853	0,10849723	0,96458135	Unpaired t-te	0,051176207	permutation FDR (250 perm	1
3528	Tnni2~P1341	Tnni2	ko vs wt	ko	wt	25,1753784	0,82269148	23,8961991	0,8747648	2,42700874	1,27917931	0,71868825	0,17451321	0,75817171	Unpaired t-te	0,133033147	permutation FDR (250 perm	1
3529	Tnnt3~Q9QZ	Tnnt3	ko vs wt	ko	wt	24,2851368	0,32520635	24,2248096	0,33391263	1,04270215	0,06032711	0,0463461	0,85079253	0,07017633	Unpaired t-te	0,920551724	permutation FDR (250 perm	1
3530	Tnn~Q80Z71	Tnn	ko vs wt	ko	wt	31,4598812	0,33504871	28,5153995	2,70321314	7,69798965	2,94448173	1,0079618	0,26265622	0,58061231	Unpaired t-te	0,070552672	permutation FDR (250 perm	1
3531	Tnpo1~Q8BF	Tnpo1	ko vs wt	ko	wt	27,7670105	0,32727831	28,1490175	0,46795582	0,76736934	-0,382007	-0,2766073	0,38412514	0,41552727	Unpaired t-te	0,530535539	permutation FDR (250 perm	1
3532	Tnpo2~Q99L	Tnpo2	ko vs wt	ko	wt	24,2498291	0,88782678	23,806623	1,03722844	1,3596225	0,44320614	0,23390657	0,64749124	0,1887661	Unpaired t-te	0,590328661	permutation FDR (250 perm	1
3533	Tnpo3~Q6P2	Tnpo3	ko vs wt	ko	wt	24,5650862	0,26366347	24,8435049	1,7933029	0,82449426	-0,2784186	-0,1222657	0,84714693	0,07204126	Unpaired t-te	0,798491492	permutation FDR (250 perm	1
3534	Tns1~A0A08	Tns1	ko vs wt	ko	wt	34,4127224	0,53912332	34,933564	0,12460853	0,69696518	-0,5208415	-0,3935353	0,19346729	0,71339244	Unpaired t-te	0,379399532	permutation FDR (250 perm	1
3535	Tns1~A0A08	Tns1	ko vs wt	ko	wt	24,7549653	0,34037322	25,2787952	2,16869366	0,69552297	-0,5238299	-0,2057432	0,76609762	0,11571588	Unpaired t-te	0,657095252	permutation FDR (250 perm	1
3536	Tns1~E9Q0S	Tns1	ko vs wt	ko	wt	29,5178038	1,00829844	30,5673402	0,47109881	0,48312339	-1,0495364	-0,6281968	0,18404149	0,73508427	Unpaired t-te	0,173408675	permutation FDR (250 perm	1
3537	Tns2~Q8CGB	Tns2	ko vs wt	ko	wt	31,5272975	0,5809218	31,979537	0,1114812	0,73090741	-0,4522394	-0,3363539	0,27256086	0,56453651	Unpaired t-te	0,447985938	permutation FDR (250 perm	1
3538	Tns3~Q5SSZ	Tns3	ko vs wt	ko	wt	28,6510876	0,16985569	28,5681132	0,43992718	1,0591995	0,08297434	0,06256704	0,81936357	0,08652335	Unpaired t-te	0,896150943	permutation FDR (250 perm	1
3539	Tnxb~O3545	Tnxb	ko vs wt	ko	wt	35,1874903	0,48745031	35,4767285	0,17184673	0,8183341	-0,2892381	-0,2213768	0,39865654	0,39940111	Unpaired t-te	0,609852706	permutation FDR (250 perm	1
3540	Tollip~Q9QZ	Tollip	ko vs wt	ko	wt	27,4276895	0,41541987	26,8871764	0,30226445	1,45448971	0,54051309	0,40908986	0,1537415	0,81320888	Unpaired t-te	0,350962258	permutation FDR (250 perm	1
3541	Tom1l1~Q92	Tom1l1	ko vs wt	ko	wt	24,0840406	1,51615574	23,3623129	0,81991442	1,64915581	0,72172771	0,35207272	0,52350781	0,28107683	Unpaired t-te	0,417576547	permutation FDR (250 perm	1
3542	Tom1l2~Q5S	Tom1l2	ko vs wt	ko	wt	24,7352228	1,09126584	24,4471865	0,53696707	1,22097727	0,28803635	0,16595694	0,71266222	0,14711626	Unpaired t-te	0,703361605	permutation FDR (250 perm	1
3543	Tom1~O887	Tom1	ko vs wt	ko	wt	24,913637	1,34002314	25,7457971	0,72426776	0,56168759	-0,8321602	-0,4316603	0,41234208	0,38474235	Unpaired t-te	0,326089532	permutation FDR (250 perm	1
3544	Tomm34~Q9	Tomm34	ko vs wt	ko	wt	27,5281753	0,39687529	27,2646384	0,28736882	1,20041802	0,26353688	0,20174954	0,42908405	0,36745763	Unpaired t-te	0,63947877	permutation FDR (250 perm	1
3545	Tomm40~Q9	Tomm40	ko vs wt	ko	wt	25,0440613	1,60859705	26,4500566	0,89668291	0,37735772	-1,4059953	-0,6617939	0,26797392	0,57190748	Unpaired t-te	0,153827586	permutation FDR (250 perm	1
3546	Tomm70~Q9	Tomm70	ko vs wt	ko	wt	28,7480387	0,61600518	28,6887087	0,60060312	1,04198172	0,05932997	0,03818035	0,91945596	0,03646907	Unpaired t-te	0,933475027	permutation FDR (250 perm	1
3547	Top1~Q0475	Top1	ko vs wt	ko	wt	28,7703429	0,38554786	28,5134562	0,61772317	1,19489739	0,25688673	0,17237887	0,63611904	0,1964616	Unpaired t-te	0,700602237	permutation FDR (250 perm	1
3548	Top2b~Q645	Top2b	ko vs wt	ko	wt	30,5538957	0,37728473	30,5311462	0,53803554	1,01589369	0,02274944	0,01581587	0,96160644	0,01700264	Unpaired t-te	0,972249215	permutation FDR (250 perm	1
3549	Top3b~Q9Z3	Top3b	ko vs wt	ko	wt	27,7091414	0,52004522	27,781371	0,13487911	0,95116688	-0,0722296	-0,0549261	0,83114179	0,08032488	Unpaired t-te	0,906726149	permutation FDR (250 perm	1
3550	Tor1aip1~Q9	Tor1aip1	ko vs wt	ko	wt	28,6597603	0,40624638	29,1884209	0,09919833	0,69319804	-0,5286605	-0,4246916	0,10630216	0,9734579	Unpaired t-te	0,343826798	permutation FDR (250 perm	1
3551	Tor1aip2~Q8	Tor1aip2	ko vs wt	ko	wt	24,4925419	0,69344877	24,9165219	0,61478751	0,74536552	-0,42398	-0,2664877	0,50776197	0,29433983	Unpaired t-te	0,536579462	permutation FDR (250 perm	1
3552	Tor1b~Q9ER	Tor1b	ko vs wt	ko	wt	27,9596377	0,48956291	27,8170116	0,10471289	1,10391268	0,14262606	0,11037574	0,65521444	0,18361654	Unpaired t-te	0,809288173	permutation FDR (250 perm	1
3553	Tor2a~P0C7V	Tor2a	ko vs wt	ko	wt	24,5297158	0,60415758	24,9196589	0,35475344	0,76335544	-0,3899431	-0,2727544	0,4062821	0,39117231	Unpaired t-te	0,525161092	permutation FDR (250 perm	1
3554	Tor3a~Q9ER	Tor3a	ko vs wt	ko	wt	24,8138976	1,26101659	24,7602355	1,26286043	1,03789612	0,05366206	0,0249341	0,96494413	0,01549783	Unpaired t-te	0,955418794	permutation FDR (250 perm	1
3555	Tpbg~Q9Z0L	Tpbg	ko vs wt	ko	wt	24,3657849	0,48502046	24,177256	0,61482387	1,13960108	0,18852889	0,12426714	0,73586732	0,13320048	Unpaired t-te	0,78308613	permutation FDR (250 perm	1
3556	Tpi1~P17751	Tpi1	ko vs wt	ko	wt	31,047303	0,63521052	30,3021159	0,40106068	1,67619157	0,74518704	0,50914765	0,16903847	0,77201444	Unpaired t-te	0,252821559	permutation FDR (250 perm	1
3557	Tpm1~B7ZN	Tpm1	ko vs wt	ko	wt	27,7909884	1,31198961	28,9417959	0,37156374	0,45037306	-1,1508076	-0,6387168	0,23030241	0,63770151	Unpaired t-te	0,173923688	permutation FDR (250 perm	1
3558	Tpm1~F8WI	Tpm1	ko vs wt	ko	wt	32,8009196	0,74113552	33,3723839	0,50041168	0,67293342	-0,5714643	-0,3674427	0,35057909	0,45521399	Unpaired t-te	0,397424242	permutation FDR (250 perm	1
3559	Tpm2~P5877	Tpm2	ko vs wt	ko	wt	31,239016	1,24274471	32,5073025	0,64183772	0,41515254	-1,2682866	-0,6859351	0,19852995	0,70217396	Unpaired t-te	0,143473154	permutation FDR (250 perm	1
3560	Tpm3~P211C	Tpm3	ko vs wt	ko	wt	24,8676915	0,653046	25,5015109	1,22193455	0,64446799	-0,6338194	-0,3262538	0,55321364	0,25710712	Unpaired t-te	0,467653494	permutation FDR (250 perm	1
3561	Tpm3~Q8K0	Tpm3	ko vs wt	ko	wt	29,3604394	0,98458164	29,4363857	0,78218265	0,94871959	-0,0759464	-0,0423543	0,92754446	0,03266527	Unpaired t-te	0,927003733	permutation FDR (250 perm	1
3562	Tpm4~Q6IRL	Tpm4	ko vs wt	ko	wt	27,7841641	1,16763191	25,432707	0,22360529	5,10339434	2,35145712	1,38939987	0,03643526	1,43847807	Unpaired t-te	0,014667729	permutation FDR (250 perm	1
3563	Tpp1~O8902	Tpp1	ko vs wt	ko	wt	30,005103	0,83068603	25,9355255	2,92708468	16,7905484	4,06957744	1,30243146	0,18294938	0,73766905	Unpaired t-te	0,027631544	permutation FDR (250 perm	1
3564	Tpp2~Q6451	Tpp2	ko vs wt	ko	wt	28,017486	1,10716719	26,3187596	0,44384582	3,24614276	1,69872645	0,99218085	0,07174936	1,14418199	Unpaired t-te	0,049582343	permutation FDR (250 perm	1
3565	Tppp3~Q9CR	Tppp3	ko vs wt	ko	wt	29,4071364	1,00828912	30,2017105	0,28521108	0,57651337	-0,794574	-0,4916674	0,27215642	0,56518142	Unpaired t-te	0,275975801	permutation FDR (250 perm	1
3566	Tprg1l~Q9D	Tprg1l	ko vs wt	ko	wt	25,4936483	1,63321126	25,4511105	1,21949547	1,02992395	0,04253781	0,01867515	0,97474687	0,01110815	Unpaired t-te	0,967579942	permutation FDR (250 perm	1
3567	Tprkb~Q8QZ	Tprkb	ko vs wt	ko	wt	24,9991628	0,07388855	24,0562442	0,29515544	1,92241341	0,94291862	0,77733043	0,04085234	1,38878311	Unpaired t-te	0,134070266	permutation FDR (250 perm	1
3568	Tpr~Q7M73	Tpr	ko vs wt	ko	wt	27,9656118	0,48562341	28,7427472	0,54681418	0,5835243	-0,7771354	-0,5259402	0,18027478	0,74406504	Unpaired t-te	0,246434273	permutation FDR (250 perm	1
3569	Tpt1~P6302	Tpt1	ko vs wt	ko	wt	29,3150779	0,14266068	29,0983293	0,20492607	1,16211158	0,2167486	0,1857828	0,27744814	0,55681819	Unpaired t-te	0,677084589	permutation FDR (250 perm	1
3570	Tra2a~Q6PF	Tra2a	ko vs wt	ko	wt	24,8378336	1,01392882	26,6683461	0,46116555	0,28116473	-1,8305125	-1,0960561	0,04607815	1,33650498	Unpaired t-te	0,032932826	permutation FDR (250 perm	1
3571	Tra2b~P629	Tra2b	ko vs wt	ko	wt	29,3654918	0,38614348	29,3793493	0,63685889	0,99044071	-0,0138575	-0,009223	0,97974121	0,00888863	Unpaired t-te	0,985154047	permutation FDR (250 perm	1
3572	Trabd~Q99J	Trabd	ko vs wt	ko	wt	24,6338978	0,24877674	24,0907283	0,5139931	1,45717026	0,54316946	0,3905447	0,27050013	0,56783252	Unpaired t-te	0,393664572	permutation FDR (250 perm	1
3573	Tradd~Q3U0	Tradd	ko vs wt	ko	wt	24,5357709	0,65001261	24,3297151	0,19029309	1,1535302	0,20605578	0,14732176	0,63439635	0,19763932	Unpaired t-te	0,739651682	permutation FDR (250 perm	1
3574	Traf7~Q922E	Traf7	ko vs wt	ko	wt	24,2404697	0,39878226	24,1830582	0,78871494	1,04059701	0,05741147	0,03580694	0,93083521	0,0311272	Unpaired t-te	0,940182445	permutation FDR (250 perm	1
3575	Trap1~Q9CQ	Trap1	ko vs wt	ko	wt	29,0389224	0,39728476	29,0529037	0,34215583	0,99035571	-0,0139813	-0,0104856	0,96827617	0,01400076	Unpaired t-te	0,982189133	permutation FDR (250 perm	1
3576	Trappc11~B2	Trappc11	ko vs wt	ko	wt	27,4306339	0,62371277	27,2882001	0,25550686	1,10376557	0,14243379	0,10152937	0,74037343	0,13054917	Unpaired t-te	0,821990605	permutation FDR (250 perm	1
3577	Trappc13~Q	Trappc13	ko vs wt	ko	wt	25,5738625	0,01601659	25,3086569	0,21420501	1,20180732	0,26520562	0,23026361	0,22170323	0,65422797	Unpaired t-te	0,618497935	permutation FDR (250 perm	1
3578	Trappc1~Q5I	Trappc1	ko vs wt	ko	wt	24,6698866	0,78196628	23,7431646	0,45246717	1,90095186	0,92672199	0,59659893	0,15588262	0,80720232	Unpaired t-te	0,190139245	permutation FDR (250 perm	1
3579	Trappc2l~Q9	Trappc2l	ko vs wt	ko	wt	25,2333642	1,1504233	24,3799811	0,5244564	1,80673267	0,85338306	0,48468022	0,31814339	0,4973771	Unpaired t-te	0,275548754	permutation FDR (250 perm	1
3580	Trappc6b~Q	Trappc6b	ko vs wt	ko	wt	24,7341289	0,76941498	24,2214545	0,43547394	1,42669241	0,51267433	0,33279503	0,38740644	0,41183316	Unpaired t-te	0,441082745	permutation FDR (250 perm	1
3581	Trappc9~Q3l	Trappc9	ko vs wt	ko	wt	24,9047151	0,90636421	25,2037451	0,45842301	0,81279869	-0,29903	-0,1850943	0,64888897	0,18782961	Unpaired t-te	0,67033314	permutation FDR (250 perm	1

3582	Trex1~Q91XE	Trex1	ko vs wt	ko	wt	24,4542425	0,91240427	24,0624885	0,17193543	1,31198759	0,39175407	0,25428266	0,51648944	0,28693855	Unpaired t-te	0,562881305	permutation FDR (250 perm	1
3583	Trim14~Q8B	Trim14	ko vs wt	ko	wt	24,4114821	0,69228103	23,9122909	0,21861514	1,41342091	0,49919116	0,34944128	0,31136504	0,50673015	Unpaired t-te	0,42773437	permutation FDR (250 perm	1
3584	Trim25~Q61	Trim25	ko vs wt	ko	wt	27,0719385	0,50731973	27,01402	0,30840526	1,04096281	0,05791853	0,04242592	0,88023195	0,05540287	Unpaired t-te	0,9267584	permutation FDR (250 perm	1
3585	Trim28~Q62	Trim28	ko vs wt	ko	wt	29,3019465	0,39178447	29,075613	0,13677153	1,1698581	0,22633355	0,18164745	0,40965612	0,38758055	Unpaired t-te	0,679257374	permutation FDR (250 perm	1
3586	Trim2~Q9ESI	Trim2	ko vs wt	ko	wt	24,098751	0,36645072	24,3678414	0,81003239	0,82984259	-0,2690904	-0,1670741	0,69404452	0,15861267	Unpaired t-te	0,714762286	permutation FDR (250 perm	1
3587	Trim32~Q8CI	Trim32	ko vs wt	ko	wt	26,152373	0,55146889	26,3469707	0,5111579	0,87381655	-0,1945977	-0,131336	0,70468271	0,15200639	Unpaired t-te	0,765899523	permutation FDR (250 perm	1
3588	Trim3~Q9R1	Trim3	ko vs wt	ko	wt	28,4127907	0,33510881	27,7802514	0,08323678	1,55029134	0,63253936	0,52613856	0,04173145	1,37953647	Unpaired t-te	0,249657185	permutation FDR (250 perm	1
3589	Trim47~Q8C	Trim47	ko vs wt	ko	wt	29,1662581	0,04843353	28,7436356	0,36930659	1,34036175	0,42262242	0,33471541	0,24609933	0,60888957	Unpaired t-te	0,471039141	permutation FDR (250 perm	1
3590	Trim56~Q80	Trim56	ko vs wt	ko	wt	26,1321305	0,30079224	25,9589494	0,01344503	1,12754193	0,17318109	0,14752344	0,39237312	0,40630075	Unpaired t-te	0,743552542	permutation FDR (250 perm	1
3591	Triobp~Q99K	Triobp	ko vs wt	ko	wt	27,0912054	0,13961986	27,5358476	0,08934592	0,73476651	-0,4446422	-0,403334	0,00744532	2,12811652	Unpaired t-te	0,356884824	permutation FDR (250 perm	1
3592	Trio~Q0KL02	Trio	ko vs wt	ko	wt	27,3679722	0,18054802	27,6011123	0,2302774	0,85078113	-0,2331401	-0,1953679	0,30156875	0,52061367	Unpaired t-te	0,657741917	permutation FDR (250 perm	1
3593	Trip10~Q8CJ	Trip10	ko vs wt	ko	wt	27,5598423	0,42646609	28,3681511	0,57585406	0,57105085	-0,8083089	-0,5476923	0,17649565	0,753266	Unpaired t-te	0,234356428	permutation FDR (250 perm	1
3594	Trip11~E9Q5	Trip11	ko vs wt	ko	wt	27,3737632	0,54477381	27,5067252	0,63163361	0,91195719	-0,132962	-0,0859891	0,82012503	0,08611993	Unpaired t-te	0,852199452	permutation FDR (250 perm	1
3595	Trip12~G5E8	Trip12	ko vs wt	ko	wt	26,8693827	0,46441829	26,5222457	0,16795332	1,27203375	0,34713695	0,26842096	0,30118557	0,52116584	Unpaired t-te	0,536811736	permutation FDR (250 perm	1
3596	Trip6~Q9Z1Y	Trip6	ko vs wt	ko	wt	29,2298604	0,81921166	29,5863153	0,6694202	0,78108151	-0,356455	-0,2135546	0,61797489	0,20902917	Unpaired t-te	0,61954023	permutation FDR (250 perm	1
3597	Trmt61a~Q8	Trmt61a	ko vs wt	ko	wt	24,4811424	0,29286637	24,287149	0,88857026	1,14392572	0,19399338	0,11752417	0,79048875	0,10210431	Unpaired t-te	0,803972168	permutation FDR (250 perm	1
3598	Trpc6~Q6114	Trpc6	ko vs wt	ko	wt	26,3773293	0,37016513	26,9817568	0,21789235	0,65773233	-0,6044275	-0,4783897	0,07101841	1,14862908	Unpaired t-te	0,278534611	permutation FDR (250 perm	1
3599	Trrap~F7CGC	Trrap	ko vs wt	ko	wt	24,8870599	1,41965676	24,0196567	0,34677121	1,82437619	0,86740325	0,46746897	0,37519865	0,42573874	Unpaired t-te	0,300379951	permutation FDR (250 perm	1
3600	Try10~Q7922	Try10	ko vs wt	ko	wt	24,6890783	1,03666716	25,8497381	1,75187201	0,44730792	-1,1606598	-0,4885396	0,46205066	0,3353104	Unpaired t-te	0,287554459	permutation FDR (250 perm	1
3601	Tsen34~H7B	Tsen34	ko vs wt	ko	wt	25,3410045	0,49163915	25,1080247	0,91119203	1,17525984	0,23297975	0,13672013	0,76405453	0,11687564	Unpaired t-te	0,764733914	permutation FDR (250 perm	1
3602	Tsfm~Q9CZR	Tsfm	ko vs wt	ko	wt	26,6287611	0,70400594	25,5472351	0,48325162	2,11627334	1,08152598	0,70641228	0,09732074	1,01179458	Unpaired t-te	0,133211183	permutation FDR (250 perm	1
3603	Tsg101~Q61	Tsg101	ko vs wt	ko	wt	27,3062484	0,34469546	27,5753352	0,43426931	0,82984466	-0,2690868	-0,1969999	0,50703342	0,29496342	Unpaired t-te	0,654330516	permutation FDR (250 perm	1
3604	Tsku~Q8CBR	Tsku	ko vs wt	ko	wt	26,5588241	0,88921751	24,4459388	0,94360815	4,32555533	2,11288536	1,14713315	0,06443763	1,19086042	Unpaired t-te	0,028274045	permutation FDR (250 perm	1
3605	Tsnax~Q9QZ	Tsnax	ko vs wt	ko	wt	25,5882137	0,77768935	25,7441588	0,48598234	0,89754424	-0,155945	-0,0996192	0,7937728	0,10030379	Unpaired t-te	0,823728327	permutation FDR (250 perm	1
3606	Tst~P52196	Tst	ko vs wt	ko	wt	24,6155851	1,05539251	25,0734102	1,26593292	0,72808308	-0,457825	-0,2198063	0,69557379	0,15765679	Unpaired t-te	0,614714328	permutation FDR (250 perm	1
3607	Ttc21b~Q0H	Ttc21b	ko vs wt	ko	wt	24,6937305	0,69204365	24,5577843	1,02060326	1,09881317	0,13594611	0,07449517	0,87892854	0,05604643	Unpaired t-te	0,875458606	permutation FDR (250 perm	1
3608	Ttc28~Q80XJ	Ttc28	ko vs wt	ko	wt	27,397071	0,26391719	26,9285125	0,23240492	1,38372626	0,46855857	0,38277617	0,0952178	1,02128187	Unpaired t-te	0,382071357	permutation FDR (250 perm	1
3609	Ttc38~A3KM	Ttc38	ko vs wt	ko	wt	24,4361919	0,85256737	24,0900935	0,34153661	1,27111843	0,34609845	0,22353698	0,5602564	0,25161317	Unpaired t-te	0,604550948	permutation FDR (250 perm	1
3610	Ttc7a~Q8BG	Ttc7a	ko vs wt	ko	wt	25,4919626	0,72048547	24,4158811	0,71294201	2,10830196	1,07608151	0,65075582	0,16977469	0,77012705	Unpaired t-te	0,162808269	permutation FDR (250 perm	1
3611	Ttc7b~E9Q6I	Ttc7b	ko vs wt	ko	wt	27,1188997	0,63265633	25,5212821	1,07610341	3,02643148	1,59761769	0,86636441	0,15709924	0,80382592	Unpaired t-te	0,089628504	permutation FDR (250 perm	1
3612	Ttll12~Q3UD	Ttll12	ko vs wt	ko	wt	25,5867507	0,71243321	24,7894903	0,54156654	1,73779801	0,7972604	0,51041284	0,21618078	0,66518292	Unpaired t-te	0,252266764	permutation FDR (250 perm	1
3613	Ttn~A2ASS6	Ttn	ko vs wt	ko	wt	31,1582747	3,04826081	28,1375214	2,58750938	8,115912	3,02075322	0,85363822	0,29048152	0,53688149	Unpaired t-te	0,079891358	permutation FDR (250 perm	1
3614	Ttr~P07309	Ttr	ko vs wt	ko	wt	29,6635738	0,82791746	28,7459207	2,0062666	1,88903974	0,91765305	0,36750093	0,59183928	0,22779621	Unpaired t-te	0,423364109	permutation FDR (250 perm	1
3615	Tuba1a~P68	Tuba1a	ko vs wt	ko	wt	28,5743574	0,97981683	28,8723551	0,27495547	0,8133805	-0,2979977	-0,1864612	0,64669225	0,18930234	Unpaired t-te	0,673293503	permutation FDR (250 perm	1
3616	Tuba1b~P05	Tuba1b	ko vs wt	ko	wt	35,1869382	0,258844	35,036768	0,25922433	1,10970042	0,15017025	0,12144788	0,55780582	0,25351696	Unpaired t-te	0,78592456	permutation FDR (250 perm	1
3617	Tuba3b~P05	Tuba3b	ko vs wt	ko	wt	25,4604497	2,00485201	24,6966556	0,29296794	1,6979501	0,76379406	0,35102547	0,55973193	0,25201992	Unpaired t-te	0,431657733	permutation FDR (250 perm	1
3618	Tuba4a~P68	Tuba4a	ko vs wt	ko	wt	29,2687693	2,08858457	27,8166532	0,2351947	2,73609068	1,45211605	0,65491486	0,31581498	0,50056727	Unpaired t-te	0,171665734	permutation FDR (250 perm	1
3619	Tubb1~A2AC	Tubb1	ko vs wt	ko	wt	29,0716556	4,08325259	24,4044128	0,21278034	25,4085627	4,66724286	1,3881253	0,14190549	0,84800079	Unpaired t-te	0,016220155	permutation FDR (250 perm	1
3620	Tubb2a~Q7T	Tubb2a	ko vs wt	ko	wt	29,0751246	0,78315266	28,8711535	0,70614242	1,15186451	0,20397103	0,1218743	0,77532302	0,11051732	Unpaired t-te	0,784163265	permutation FDR (250 perm	1
3621	Tubb2b~Q9C	Tubb2b	ko vs wt	ko	wt	30,2256308	0,57923829	30,0282076	0,18867055	1,14664848	0,19742318	0,14515858	0,61346506	0,21221016	Unpaired t-te	0,742916384	permutation FDR (250 perm	1
3622	Tubb3~Q9ER	Tubb3	ko vs wt	ko	wt	29,5567303	0,48041292	29,2228572	0,22406593	1,26039253	0,33387311	0,25304361	0,34823432	0,45812843	Unpaired t-te	0,556874357	permutation FDR (250 perm	1
3623	Tubb4a~Q9D	Tubb4a	ko vs wt	ko	wt	27,8749385	0,5633157	28,2448903	0,30758226	0,77380834	-0,3699518	-0,2659128	0,38920595	0,40982053	Unpaired t-te	0,536361858	permutation FDR (250 perm	1
3624	Tubb4b~P68	Tubb4b	ko vs wt	ko	wt	34,749201	0,09161861	34,6840684	0,10412915	1,04618113	0,06513265	0,0597185	0,51314423	0,28976055	Unpaired t-te	0,896752155	permutation FDR (250 perm	1
3625	Tubb5~P990	Tubb5	ko vs wt	ko	wt	32,4437289	0,33218014	32,4469448	0,04628397	0,99777335	-0,003216	-0,0026922	0,98779677	0,0053324	Unpaired t-te	0,995155047	permutation FDR (250 perm	1
3626	Tubb6~Q922	Tubb6	ko vs wt	ko	wt	28,3480257	0,52099643	28,4131674	0,30431525	0,95585141	-0,0651417	-0,0475542	0,86720503	0,06187821	Unpaired t-te	0,91726023	permutation FDR (250 perm	1
3627	Tubg1~P838	Tubg1	ko vs wt	ko	wt	25,0405694	0,74162444	24,3675087	0,57977755	1,59445208	0,67306074	0,42256568	0,30892098	0,5101526	Unpaired t-te	0,33575334	permutation FDR (250 perm	1
3628	Tubgcp2~Q9	Tubgcp2	ko vs wt	ko	wt	24,8271316	0,37828452	25,2360354	0,91701422	0,75319545	-0,4089038	-0,2427852	0,60057909	0,22142979	Unpaired t-te	0,590256563	permutation FDR (250 perm	1
3629	Tubgcp3~P5	Tubgcp3	ko vs wt	ko	wt	25,0611647	0,68194796	24,2952113	0,62567476	1,70049332	0,76595334	0,48105295	0,25793667	0,5884869	Unpaired t-te	0,278506212	permutation FDR (250 perm	1
3630	Tufm~Q8BFI	Tufm	ko vs wt	ko	wt	31,6684332	0,25065359	31,8295559	0,66064797	0,89432882	-0,1611227	-0,108205	0,76851973	0,11434498	Unpaired t-te	0,818449115	permutation FDR (250 perm	1
3631	Tut7~Q5BLK	Tut7	ko vs wt	ko	wt	25,1497007	0,8718333	24,8271896	0,67233735	1,25050528	0,32251115	0,19056719	0,66134161	0,17957415	Unpaired t-te	0,659860262	permutation FDR (250 perm	1
3632	Twf1~Q91YR	Twf1	ko vs wt	ko	wt	30,2100786	0,5970741	29,9976376	0,28721025	1,15864697	0,21244106	0,15173313	0,61999911	0,20760894	Unpaired t-te	0,729328032	permutation FDR (250 perm	1
3633	Twf2~Q9Z0P	Twf2	ko vs wt	ko	wt	25,5900234	1,59326299	25,4961286	1,24282702	1,06724752	0,09389481	0,04132345	0,94409516	0,02498423	Unpaired t-te	0,927958455	permutation FDR (250 perm	1
3634	Txndc12~Q9	Txndc12	ko vs wt	ko	wt	25,4518021	1,07312719	23,5337275	0,45261864	3,77918345	1,91807455	1,13003967	0,04683093	1,32946725	Unpaired t-te	0,029301173	permutation FDR (250 perm	1
3635	Txndc5~Q91	Txndc5	ko vs wt	ko	wt	30,8862716	0,40728802	30,6539091	0,27239088	1,17475715	0,23236254	0,17819733	0,4791057	0,31956866	Unpaired t-te	0,680549654	permutation FDR (250 perm	1
3636	Txnip~Q8BG	Txnip	ko vs wt	ko	wt	25,2578161	0,75070337	24,3089854	0,35309871	1,93030746	0,94883066	0,63247317	0,12179853	0,91435795	Unpaired t-te	0,171413035	permutation FDR (250 perm	1
3637	Txnl1~Q8CD	Txnl1	ko vs wt	ko	wt	22,9066987	0,36205606	25,5857678	1,00035949	0,15614204	-2,6790691	-1,5418211	0,05314401	1,2745457	Unpaired t-te	0,013432981	permutation FDR (250 perm	1
3638	Txn~P10639	Txn	ko vs wt	ko	wt	31,4871336	0,35787158	29,6675855	1,65902755	3,52970628	1,81954814	0,8304017	0,25910123	0,58653052	Unpaired t-te	0,1162453	permutation FDR (250 perm	1
3639	U2af1~Q9D8	U2af1	ko vs wt	ko	wt	28,2859006	0,03891992	28,244836	0,25182722	1,02887277	0,04106459	0,03481581	0,83970817	0,07587162	Unpaired t-te	0,943021186	permutation FDR (250 perm	1
3640	U2af2~P263	U2af2	ko vs wt	ko	wt	29,3161923	0,51181033	29,6638965	0,29978866	0,78583361	-0,3477042	-0,2549775	0,38355425	0,41617321	Unpaired t-te	0,552407643	permutation FDR (250 perm	1
3641	U2surp~Q6N	U2surp	ko vs wt	ko	wt	27,4201344	0,54056103	27,5195079	0,1270228	0,93343825	-0,0993735	-0,0750124	0,77701726	0,10956933	Unpaired t-te	0,873848501	permutation FDR (250 perm	1
3642	Uaca~Q8CGE	Uaca	ko vs wt	ko	wt	27,9733824	0,57476788	28,4038924	0,34391128	0,74199944	-0,43051	-0,3050216	0,34377137	0,4637303	Unpaired t-te	0,479292637	permutation FDR (250 perm	1
3643	Uap1l1~Q3T	Uap1l1	ko vs wt	ko	wt	26,3400685	1,18203213	26,0692637	0,9106431	1,20648071	0,27080485	0,13971402	0,78468983	0,10530198	Unpaired t-te	0,749123203	permutation FDR (250 perm	1
3644	Uba1~Q0205	Uba1	ko vs wt	ko	wt	30,6654711	0,60328919	30,1889003	0,1064608	1,39143239	0,4765708	0,35136237	0,26638108	0,57449663	Unpaired t-te	0,430361991	permutation FDR (250 perm	1
3645	Uba2~Q9Z1F	Uba2	ko vs wt	ko	wt	24,7974781	0,3600075	24,6515463	0,59023387	1,10644499	0,14593173	0,09952713	0,774834	0,11079133	Unpaired t-te	0,830827681	permutation FDR (250 perm	1

3646	Uba5~Q8VE4	Uba5	ko vs wt	ko	wt	26,1863134	0,246644	24,0811213	0,25333091	4,30255046	2,10519211	1,71315919	0,00061003	3,21464732	Unpaired t-te	0,005287532	permutation FDR (250 perm	1
3647	Uba6~Q8C7F	Uba6	ko vs wt	ko	wt	27,4072808	0,90082986	26,8994909	0,54192414	1,42187029	0,50778986	0,30849596	0,46777432	0,32996363	Unpaired t-te	0,473320731	permutation FDR (250 perm	1
3648	Uba7~Q9DBI	Uba7	ko vs wt	ko	wt	24,2669157	2,09442648	24,0209705	0,60593421	1,18586942	0,24594516	0,10773451	0,85798968	0,06651794	Unpaired t-te	0,811832733	permutation FDR (250 perm	1
3649	Ubap1~Q8BI	Ubap1	ko vs wt	ko	wt	24,1199806	0,47166043	24,3154178	0,50739887	0,87330824	-0,1954371	-0,1347449	0,686386	0,16343158	Unpaired t-te	0,761619128	permutation FDR (250 perm	1
3650	Ubap2l~Q80	Ubap2l	ko vs wt	ko	wt	25,024434	1,61994319	27,2715904	0,20232231	0,21063888	-2,2471563	-1,1546657	0,09420387	1,02593124	Unpaired t-te	0,0329981	permutation FDR (250 perm	1
3651	Ubash3a~Q3	Ubash3a	ko vs wt	ko	wt	24,034141	1,11024966	24,5737058	0,74412129	0,68797839	-0,5395648	-0,2949566	0,54399887	0,264402	Unpaired t-te	0,492727386	permutation FDR (250 perm	1
3652	Ubash3b~Q8	Ubash3b	ko vs wt	ko	wt	25,3306214	2,80250723	24,5117404	0,57755622	1,76403712	0,81888092	0,30683806	0,65380132	0,18455421	Unpaired t-te	0,487130598	permutation FDR (250 perm	1
3653	Ube2d3~P61	Ube2d3	ko vs wt	ko	wt	28,8278793	0,5341604	29,2573813	0,13909998	0,74251805	-0,429502	-0,3244701	0,26274026	0,58047337	Unpaired t-te	0,460729927	permutation FDR (250 perm	1
3654	Ube2i~P632	Ube2i	ko vs wt	ko	wt	25,6209568	1,28958841	24,1603745	1,11194436	2,75219434	1,46058235	0,70124344	0,23892274	0,62174252	Unpaired t-te	0,136739796	permutation FDR (250 perm	1
3655	Ube2l3~P68	Ube2l3	ko vs wt	ko	wt	27,3768989	0,58323725	27,179801	0,07734762	1,14638997	0,19709789	0,1469624	0,60207356	0,22035045	Unpaired t-te	0,743656497	permutation FDR (250 perm	1
3656	Ube2m~P61	Ube2m	ko vs wt	ko	wt	28,9218479	0,12592312	28,0958279	0,25271346	1,77278798	0,82602	0,69243618	0,02952515	1,5298079	Unpaired t-te	0,159109121	permutation FDR (250 perm	1
3657	Ube2n~P610	Ube2n	ko vs wt	ko	wt	29,7410052	1,006643	30,2814545	0,41532086	0,68755674	-0,5404493	-0,3273129	0,45009716	0,34669373	Unpaired t-te	0,451369697	permutation FDR (250 perm	1
3658	Ube2o~Q6ZF	Ube2o	ko vs wt	ko	wt	26,7817092	1,93181518	25,7512426	0,62053849	2,04268472	1,03046655	0,46870441	0,44001227	0,35653522	Unpaired t-te	0,295377061	permutation FDR (250 perm	1
3659	Ube2v1~Q9C	Ube2v1	ko vs wt	ko	wt	28,6842343	0,30460776	28,0740738	0,60504231	1,52642897	0,61016046	0,41718509	0,28845712	0,53991874	Unpaired t-te	0,364009444	permutation FDR (250 perm	1
3660	Ube3a~O087	Ube3a	ko vs wt	ko	wt	25,1285838	1,37099776	24,1726738	0,10973194	1,93980274	0,95590995	0,53243955	0,31416598	0,50284085	Unpaired t-te	0,250565075	permutation FDR (250 perm	1
3661	Ube4b~Q9ES	Ube4b	ko vs wt	ko	wt	23,6981467	0,51074504	24,9601504	1,12513164	0,41696446	-1,2620037	-0,682726	0,2486444	0,60442132	Unpaired t-te	0,16580718	permutation FDR (250 perm	1
3662	Ubl4a~P211	Ubl4a	ko vs wt	ko	wt	24,8943906	0,98911691	24,614976	0,53856686	1,21370228	0,27941457	0,16568704	0,70136779	0,15405418	Unpaired t-te	0,703277937	permutation FDR (250 perm	1
3663	Ubp1~Q811S	Ubp1	ko vs wt	ko	wt	25,1113898	1,42922832	26,305667	0,02211753	0,43700535	-1,1942771	-0,654286	0,24362093	0,61328541	Unpaired t-te	0,173230769	permutation FDR (250 perm	1
3664	Ubr4~A2ANC	Ubr4	ko vs wt	ko	wt	29,2522708	0,44409764	28,5895596	1,00101163	1,58305475	0,66271115	0,37808067	0,4542584	0,34269703	Unpaired t-te	0,409111402	permutation FDR (250 perm	1
3665	Ubtf~A2AW1	Ubtf	ko vs wt	ko	wt	24,794953	0,44700207	26,4388415	0,46302106	0,31999283	-1,6438885	-1,1602084	0,01552195	1,8090538	Unpaired t-te	0,026627267	permutation FDR (250 perm	1
3666	Ubxn1~Q922	Ubxn1	ko vs wt	ko	wt	24,8802119	0,66135039	26,2800061	0,3841193	0,3789832	-1,3997942	-0,9531607	0,03119361	1,5059343	Unpaired t-te	0,054486784	permutation FDR (250 perm	1
3667	Ubxn6~Q99P	Ubxn6	ko vs wt	ko	wt	26,4223407	0,37595101	26,6110649	0,19816496	0,87738129	-0,1887242	-0,1499768	0,4993258	0,301616	Unpaired t-te	0,731945516	permutation FDR (250 perm	1
3668	Uchl1~Q9R0	Uchl1	ko vs wt	ko	wt	26,659184	2,11414128	28,0822232	0,75479866	0,37292586	-1,4230393	-0,6101735	0,34511831	0,46203201	Unpaired t-te	0,187177526	permutation FDR (250 perm	1
3669	Uchl5~Q9WU	Uchl5	ko vs wt	ko	wt	25,2715532	1,64394101	26,3484146	0,3092392	0,47405902	-1,0768614	-0,5455245	0,34265162	0,46514722	Unpaired t-te	0,2378116	permutation FDR (250 perm	1
3670	Uck1~P5262	Uck1	ko vs wt	ko	wt	26,7135388	0,14063979	24,8586499	1,41448794	3,61723894	1,8548889	0,9258314	0,20413531	0,69008187	Unpaired t-te	0,089311164	permutation FDR (250 perm	1
3671	Uckl1~Q91Y	Uckl1	ko vs wt	ko	wt	26,2520933	0,53522506	24,1792127	0,45032805	4,20725879	2,07288056	1,43579254	0,00626655	2,20297175	Unpaired t-te	0,009896104	permutation FDR (250 perm	1
3672	Ucp1~P1224	Ucp1	ko vs wt	ko	wt	32,388681	0,7299789	32,3461507	0,55661563	1,02991857	0,04253028	0,02697497	0,9441162	0,02497455	Unpaired t-te	0,952058017	permutation FDR (250 perm	1
3673	Uevld~Q3U1	Uevld	ko vs wt	ko	wt	24,9875956	1,3838382	24,7801764	1,34639402	1,15462086	0,2074192	0,09247939	0,87490758	0,05803782	Unpaired t-te	0,839500413	permutation FDR (250 perm	1
3674	Ufc1~Q9CR0	Ufc1	ko vs wt	ko	wt	27,2753429	0,31753783	27,4442021	0,22117964	0,88954583	-0,1688592	-0,1360694	0,51478923	0,28837055	Unpaired t-te	0,755516174	permutation FDR (250 perm	1
3675	Ufd1~P7036	Ufd1	ko vs wt	ko	wt	26,3236653	0,67928096	26,8344756	0,35268345	0,70182813	-0,5108103	-0,3487335	0,32427546	0,48908591	Unpaired t-te	0,422686606	permutation FDR (250 perm	1
3676	Ufl1~Q8CCJ3	Ufl1	ko vs wt	ko	wt	28,8463874	0,46422449	28,7994518	0,12626771	1,03306829	0,04693563	0,03659697	0,87686773	0,05706591	Unpaired t-te	0,937216561	permutation FDR (250 perm	1
3677	Ufm1~P6196	Ufm1	ko vs wt	ko	wt	26,0041211	1,94130871	27,5257166	0,4311582	0,34830053	-1,5215954	-0,7039404	0,27098727	0,56705111	Unpaired t-te	0,144590548	permutation FDR (250 perm	1
3678	Ufsp2~Q99K	Ufsp2	ko vs wt	ko	wt	27,1640211	0,50407826	26,7109221	0,05900867	1,36897769	0,45309894	0,35015222	0,21743435	0,66267184	Unpaired t-te	0,433554337	permutation FDR (250 perm	1
3679	Ugdh~O7047	Ugdh	ko vs wt	ko	wt	29,4494978	0,17607466	28,2456422	0,87441608	2,30354469	1,20385559	0,74010248	0,18806882	0,7256832	Unpaired t-te	0,149639752	permutation FDR (250 perm	1
3680	Uggt1~Q6P5	Uggt1	ko vs wt	ko	wt	30,4516507	0,35649643	30,3681362	0,312714	1,05959613	0,08351447	0,0641388	0,79407691	0,10013743	Unpaired t-te	0,889136523	permutation FDR (250 perm	1
3681	Ugp2~Q91ZJ	Ugp2	ko vs wt	ko	wt	30,0763332	0,36709788	30,2484305	0,90860195	0,8875515	-0,1720973	-0,1026506	0,81908408	0,08667152	Unpaired t-te	0,828118036	permutation FDR (250 perm	1
3682	Ugt1a7~Q6Z	Ugt1a7	ko vs wt	ko	wt	28,0000657	0,45881296	27,2517148	0,36121968	1,67987154	0,74835092	0,54704796	0,09922978	1,00335798	Unpaired t-te	0,22370243	permutation FDR (250 perm	1
3683	Uhrf2~Q7TM	Uhrf2	ko vs wt	ko	wt	24,3193859	0,65160396	24,2201554	0,22728969	1,07120194	0,09923048	0,07042138	0,82028886	0,08603319	Unpaired t-te	0,880698016	permutation FDR (250 perm	1
3684	Umps~P1343	Umps	ko vs wt	ko	wt	25,5142429	1,13111672	25,0489197	1,12072981	1,38062662	0,4653232	0,22957554	0,67231853	0,17242492	Unpaired t-te	0,59412381	permutation FDR (250 perm	1
3685	Unc45a~Q99	Unc45a	ko vs wt	ko	wt	29,6472199	0,39005165	29,716343	0,27097619	0,95321722	-0,0691231	-0,0533488	0,82444406	0,0838388	Unpaired t-te	0,907797958	permutation FDR (250 perm	1
3686	Upf1~Q9EPU	Upf1	ko vs wt	ko	wt	30,8202276	0,36181681	30,4567802	0,2652007	1,28649641	0,36344743	0,28378402	0,25245893	0,59780926	Unpaired t-te	0,509192201	permutation FDR (250 perm	1
3687	Upf2~A2AT3	Upf2	ko vs wt	ko	wt	26,5043336	0,24068789	26,6434997	0,14827292	0,90804387	-0,1391661	-0,1185324	0,46050258	0,33676793	Unpaired t-te	0,789418202	permutation FDR (250 perm	1
3688	Uqcrb~Q9CQ	Uqcrb	ko vs wt	ko	wt	27,0383035	0,74384621	25,935019	0,12486355	2,14843257	1,1032845	0,76699976	0,07999415	1,09694175	Unpaired t-te	0,120552153	permutation FDR (250 perm	1
3689	Uqcrc1~Q9C	Uqcrc1	ko vs wt	ko	wt	31,4239169	0,32808378	32,0159872	0,06197829	0,66339024	-0,5920703	-0,4956957	0,04883946	1,31122911	Unpaired t-te	0,276572242	permutation FDR (250 perm	1
3690	Uqcrc2~Q9D	Uqcrc2	ko vs wt	ko	wt	32,1098876	0,3937003	32,2892408	0,12169397	0,88309885	-0,1793532	-0,1442852	0,50347311	0,29802372	Unpaired t-te	0,745118328	permutation FDR (250 perm	1
3691	Uqcrfs1~Q9	Uqcrfs1	ko vs wt	ko	wt	27,1843366	2,04667934	27,2882841	0,73460858	0,93048355	-0,1039475	-0,0453764	0,93964272	0,02703725	Unpaired t-te	0,922413038	permutation FDR (250 perm	1
3692	Uqcrq~Q9CQ	Uqcrq	ko vs wt	ko	wt	29,3272604	0,59201451	29,0001574	0,41368389	1,25449176	0,327103	0,22560656	0,49984326	0,30116616	Unpaired t-te	0,598022592	permutation FDR (250 perm	1
3693	Urah~Q9CRB	Urah	ko vs wt	ko	wt	26,7601846	0,43998391	25,3466039	1,05431703	2,66397527	1,41358069	0,79076815	0,18996225	0,7213327	Unpaired t-te	0,122752423	permutation FDR (250 perm	1
3694	Use1~Q9CQ	Use1	ko vs wt	ko	wt	23,953795	0,67155049	24,976984	0,6052778	0,49202754	-1,023189	-0,6486139	0,14205474	0,84754426	Unpaired t-te	0,162297769	permutation FDR (250 perm	1
3695	Uso1~Q9Z1Z	Uso1	ko vs wt	ko	wt	28,3435282	0,57185916	28,6300927	0,91125959	0,81985202	-0,2865646	-0,1662188	0,71833616	0,14367227	Unpaired t-te	0,711356019	permutation FDR (250 perm	1
3696	Usp10~P524	Usp10	ko vs wt	ko	wt	26,3493959	0,24969637	26,4171665	0,19417923	0,9541112	-0,0677707	-0,0565186	0,74770237	0,12627124	Unpaired t-te	0,901821313	permutation FDR (250 perm	1
3697	Usp14~Q9JM	Usp14	ko vs wt	ko	wt	27,4756475	0,49910843	27,2586616	0,632864	1,16230273	0,21698588	0,14161217	0,70663114	0,15080723	Unpaired t-te	0,750295858	permutation FDR (250 perm	1
3698	Usp15~Q8R5	Usp15	ko vs wt	ko	wt	27,2189611	0,68949639	26,1038764	0,40351361	2,16607717	1,11508464	0,74849173	0,07264004	1,13882395	Unpaired t-te	0,11547491	permutation FDR (250 perm	1
3699	Usp16~Q99L	Usp16	ko vs wt	ko	wt	23,8398253	0,47694365	23,8005573	0,87271565	1,02759233	0,03926803	0,02343307	0,95755419	0,01883664	Unpaired t-te	0,960028406	permutation FDR (250 perm	1
3700	Usp24~B1AY	Usp24	ko vs wt	ko	wt	25,2537151	0,70171724	25,2612693	0,08280969	0,99477749	-0,0075542	-0,0053601	0,98639199	0,00595046	Unpaired t-te	0,990494142	permutation FDR (250 perm	1
3701	Usp39~Q3TI	Usp39	ko vs wt	ko	wt	25,9895279	0,64677432	26,7183827	0,45415551	0,60338268	-0,7288548	-0,4883411	0,19912896	0,70086557	Unpaired t-te	0,270101112	permutation FDR (250 perm	1
3702	Usp47~Q8BY	Usp47	ko vs wt	ko	wt	26,3620063	0,3177576	24,6474142	0,45261035	3,28203834	1,71459209	1,25253587	0,01523754	1,81708527	Unpaired t-te	0,022499307	permutation FDR (250 perm	1
3703	Usp4~P3512	Usp4	ko vs wt	ko	wt	26,0371481	1,88823274	26,7583866	0,33785202	0,60657651	-0,7212385	-0,3408434	0,56046438	0,25145198	Unpaired t-te	0,44223375	permutation FDR (250 perm	1
3704	Usp5~P5639	Usp5	ko vs wt	ko	wt	27,2056377	0,76046554	27,4607021	0,38563395	0,83794974	-0,2550644	-0,1681548	0,64379971	0,19124923	Unpaired t-te	0,699290878	permutation FDR (250 perm	1
3705	Usp7~F8VPX	Usp7	ko vs wt	ko	wt	29,0635396	0,29578467	28,2702147	0,84823113	1,73306396	0,7933249	0,48861272	0,31560593	0,50085484	Unpaired t-te	0,299978962	permutation FDR (250 perm	1
3706	Usp8~Q80U	Usp8	ko vs wt	ko	wt	26,1154802	0,1767181	25,9222925	0,40683165	1,14328705	0,19318767	0,14801037	0,579702	0,2367952	Unpaired t-te	0,747046866	permutation FDR (250 perm	1
3707	Usp9x~P703	Usp9x	ko vs wt	ko	wt	29,515117	0,48982628	28,9552326	1,17598457	1,47415104	0,55988435	0,29807715	0,57797741	0,23808913	Unpaired t-te	0,511305908	permutation FDR (250 perm	1
3708	Utp11~Q9CZ	Utp11	ko vs wt	ko	wt	23,8057288	0,63826126	24,9508224	0,42417009	0,45216036	-1,1450936	-0,7762639	0,0608614	1,21565804	Unpaired t-te	0,104119211	permutation FDR (250 perm	1
3709	Utrn~E9Q6R	Utrn	ko vs wt	ko	wt	33,613359	0,36690308	34,1098787	0,08948551	0,70881461	-0,4965198	-0,4066234	0,09725124	1,01210487	Unpaired t-te	0,364188806	permutation FDR (250 perm	1

3710	Uvrag~Q8K2	Uvrag	ko vs wt	ko	wt	26,0845461	0,12508904	25,9133442	0,04135545	1,12599617	0,17120192	0,15882678	0,09455435	1,02431849	Unpaired t-te	0,719150755	permutation FDR (250 perm	1
3711	Vac14~Q80V	Vac14	ko vs wt	ko	wt	27,0080824	0,46726895	26,9778187	0,51092343	1,02119878	0,03026372	0,02085874	0,94969255	0,02241697	Unpaired t-te	0,963727727	permutation FDR (250 perm	1
3712	Vapa~Q9WV	Vapa	ko vs wt	ko	wt	29,6868725	0,25002672	28,9342083	0,47032856	1,68490151	0,75266427	0,552394	0,1373859	0,86205784	Unpaired t-te	0,240365512	permutation FDR (250 perm	1
3713	Vapb~Q9QY	Vapb	ko vs wt	ko	wt	29,6399123	0,35720275	30,2274686	0,27849193	0,66546912	-0,5875564	-0,4571892	0,09575662	1,01883121	Unpaired t-te	0,299666199	permutation FDR (250 perm	1
3714	Vars1~Q9Z1	Vars1	ko vs wt	ko	wt	30,5191471	0,63681873	30,0996688	0,08599956	1,33744384	0,41947832	0,30559465	0,33842358	0,47053938	Unpaired t-te	0,490757415	permutation FDR (250 perm	1
3715	Vasp~P7046	Vasp	ko vs wt	ko	wt	28,8430764	0,24437656	29,4498866	0,55241172	0,65664693	-0,6068102	-0,4287459	0,25611252	0,59156919	Unpaired t-te	0,354231108	permutation FDR (250 perm	1
3716	Vat1~Q6246	Vat1	ko vs wt	ko	wt	31,0766153	0,20450461	30,6455931	0,24303568	1,3481885	0,43102222	0,3566577	0,11175375	0,95173788	Unpaired t-te	0,419146858	permutation FDR (250 perm	1
3717	Vav1~P2787	Vav1	ko vs wt	ko	wt	27,0484874	1,68054197	26,3097191	0,2859193	1,66875055	0,73876831	0,371035	0,50618104	0,29569413	Unpaired t-te	0,406850394	permutation FDR (250 perm	1
3718	Vav2~Q6099	Vav2	ko vs wt	ko	wt	24,0457795	0,68942288	23,9099011	0,57975891	1,0987616	0,1358784	0,0864698	0,82197067	0,08514368	Unpaired t-te	0,849590123	permutation FDR (250 perm	1
3719	Vav3~Q9R0C	Vav3	ko vs wt	ko	wt	25,622327	1,17071437	24,287065	0,78392981	2,52321291	1,33526195	0,7124644	0,18717085	0,72776179	Unpaired t-te	0,130331606	permutation FDR (250 perm	1
3720	Vbp1~P6175	Vbp1	ko vs wt	ko	wt	24,2978129	1,15713841	24,8931675	1,26470045	0,66188173	-0,5953546	-0,2813227	0,62191674	0,20626775	Unpaired t-te	0,518268105	permutation FDR (250 perm	1
3721	Vcam1~P295	Vcam1	ko vs wt	ko	wt	29,6233214	0,6061494	29,9087626	1,34533217	0,82049065	-0,2854412	-0,141755	0,79953642	0,09716175	Unpaired t-te	0,757238819	permutation FDR (250 perm	1
3722	Vcan~E9QMI	Vcan	ko vs wt	ko	wt	26,4115768	0,64792816	26,3586732	0,24555399	1,0373506	0,05290357	0,03745614	0,90391578	0,04387203	Unpaired t-te	0,934943456	permutation FDR (250 perm	1
3723	Vcan~G3XA3	Vcan	ko vs wt	ko	wt	32,3617942	0,79010711	33,0837473	0,23166963	0,60627611	-0,7219531	-0,486265	0,21556993	0,66641183	Unpaired t-te	0,279904998	permutation FDR (250 perm	1
3724	Vcl~Q64727	Vcl	ko vs wt	ko	wt	35,6712337	0,35437367	36,0928093	0,2273663	0,74660883	-0,4215755	-0,3345287	0,16620634	0,77935242	Unpaired t-te	0,438394602	permutation FDR (250 perm	1
3725	Vcpip1~Q8C	Vcpip1	ko vs wt	ko	wt	26,9894144	0,37314611	26,4914759	0,27908204	1,41219422	0,49793852	0,38535432	0,14981082	0,82445682	Unpaired t-te	0,37778581	permutation FDR (250 perm	1
3726	Vcp~Q01853	Vcp	ko vs wt	ko	wt	31,7947064	0,64857086	31,5732993	0,31834111	1,16587011	0,22140707	0,15408618	0,63542553	0,19693534	Unpaired t-te	0,724374183	permutation FDR (250 perm	1
3727	Vdac1~Q609	Vdac1	ko vs wt	ko	wt	32,6654676	0,26097977	32,7294149	0,17852354	0,9566431	-0,0639473	-0,0534423	0,75811941	0,12026239	Unpaired t-te	0,907550779	permutation FDR (250 perm	1
3728	Vdac2~Q609	Vdac2	ko vs wt	ko	wt	31,9171481	0,14752225	32,0069497	0,15553933	0,93965192	-0,0898017	-0,0788352	0,55269647	0,25751331	Unpaired t-te	0,864365721	permutation FDR (250 perm	1
3729	Vdac3~Q609	Vdac3	ko vs wt	ko	wt	31,4847212	0,27917165	31,8530948	0,33143168	0,77465533	-0,3683735	-0,2867985	0,26878363	0,57059718	Unpaired t-te	0,512025966	permutation FDR (250 perm	1
3730	Vezf1~Q5SX	Vezf1	ko vs wt	ko	wt	26,6413521	0,20875768	25,335605	1,95540051	2,47211709	1,30574708	0,54681356	0,4448421	0,35179411	Unpaired t-te	0,260607942	permutation FDR (250 perm	1
3731	Vim~P20152	Vim	ko vs wt	ko	wt	38,0164891	0,24869582	38,5622568	0,2017893	0,68502678	-0,5457677	-0,4538891	0,04471828	1,34951495	Unpaired t-te	0,30381366	permutation FDR (250 perm	1
3732	Vipas39~Q8	Vipas39	ko vs wt	ko	wt	25,3798783	1,14818097	24,5732772	0,74383539	1,74908586	0,80660111	0,43689369	0,38430293	0,41532631	Unpaired t-te	0,319773246	permutation FDR (250 perm	1
3733	Vit~Q8VHI5	Vit	ko vs wt	ko	wt	24,7972624	0,75225311	24,7374169	1,18774552	1,04235413	0,0598455	0,03076075	0,9534331	0,02070978	Unpaired t-te	0,947578113	permutation FDR (250 perm	1
3734	Vps11~Q91V	Vps11	ko vs wt	ko	wt	27,3890415	0,57360465	27,2218592	1,07605394	1,12286327	0,16718226	0,09136484	0,85424018	0,06842	Unpaired t-te	0,846070782	permutation FDR (250 perm	1
3735	Vps13a~Q5H	Vps13a	ko vs wt	ko	wt	26,6685065	1,61186359	26,6690151	0,87343866	0,99964755	-0,0005086	-0,0002402	0,99965493	0,00014989	Unpaired t-te	0,999357755	permutation FDR (250 perm	1
3736	Vps13c~Q8B	Vps13c	ko vs wt	ko	wt	30,2929854	0,28578261	30,2321277	0,27394491	1,04308566	0,06085764	0,04851325	0,82168896	0,08529255	Unpaired t-te	0,915703664	permutation FDR (250 perm	1
3737	Vps13d~B1A	Vps13d	ko vs wt	ko	wt	25,4350012	1,02783431	25,0564632	1,49823311	1,30002382	0,37853806	0,17095238	0,77424075	0,11112398	Unpaired t-te	0,701402121	permutation FDR (250 perm	1
3738	Vps16~Q920	Vps16	ko vs wt	ko	wt	27,5261362	0,49875181	26,9630226	0,58359094	1,47745438	0,56311359	0,3746107	0,32852334	0,48343377	Unpaired t-te	0,396793672	permutation FDR (250 perm	1
3739	Vps18~Q8R3	Vps18	ko vs wt	ko	wt	26,5646462	1,00526739	26,2614143	0,38320355	1,2339055	0,30323191	0,18483795	0,65973086	0,1806332	Unpaired t-te	0,673528559	permutation FDR (250 perm	1
3740	Vps25~Q9CC	Vps25	ko vs wt	ko	wt	24,8446141	1,35385469	26,9205116	0,03972976	0,23718793	-2,0758975	-1,1648256	0,07651675	1,11624347	Unpaired t-te	0,032923272	permutation FDR (250 perm	1
3741	Vps26a~P40	Vps26a	ko vs wt	ko	wt	27,1399905	0,90885802	26,0779717	0,19342888	2,08785108	1,06201881	0,68861105	0,13423472	0,87213514	Unpaired t-te	0,151481848	permutation FDR (250 perm	1
3742	Vps26b~Q8C	Vps26b	ko vs wt	ko	wt	24,8716544	0,16661528	24,3999088	1,17122481	1,38678641	0,47174561	0,25725746	0,62721557	0,20258317	Unpaired t-te	0,576593572	permutation FDR (250 perm	1
3743	Vps28~Q9D1	Vps28	ko vs wt	ko	wt	26,9526838	0,20288382	27,5723386	0,02121795	0,65082663	-0,6196548	-0,5542074	0,01242848	1,90558193	Unpaired t-te	0,233749907	permutation FDR (250 perm	1
3744	Vps29~Q9Q	Vps29	ko vs wt	ko	wt	26,7892325	1,7576829	24,4581227	0,97453284	5,03192271	2,33110976	1,04691278	0,11752439	0,92987198	Unpaired t-te	0,037684466	permutation FDR (250 perm	1
3745	Vps33a~Q9D	Vps33a	ko vs wt	ko	wt	26,8306649	0,69108207	26,6652386	0,22972512	1,12149738	0,16542625	0,11561836	0,72091631	0,14211515	Unpaired t-te	0,798728033	permutation FDR (250 perm	1
3746	Vps33b~P59	Vps33b	ko vs wt	ko	wt	25,3101044	0,46621189	24,4853991	0,62536322	1,77117329	0,82470537	0,54339935	0,19770694	0,70397809	Unpaired t-te	0,237993351	permutation FDR (250 perm	1
3747	Vps35l~Q8B	Vps35l	ko vs wt	ko	wt	27,2873561	0,17962083	26,4937403	0,6199507	1,7334134	0,79361577	0,54714355	0,20727615	0,68345066	Unpaired t-te	0,255103523	permutation FDR (250 perm	1
3748	Vps35~Q9EC	Vps35	ko vs wt	ko	wt	31,1009747	0,58622247	30,3043103	0,29885053	1,73708022	0,79666438	0,56944913	0,10577153	0,97563122	Unpaired t-te	0,209283105	permutation FDR (250 perm	1
3749	Vps36~Q91X	Vps36	ko vs wt	ko	wt	28,3117216	0,12232806	28,074681	0,20226256	1,1785726	0,23704063	0,2044319	0,23465213	0,6295755	Unpaired t-te	0,646238832	permutation FDR (250 perm	1
3750	Vps37c~Q8R	Vps37c	ko vs wt	ko	wt	23,8319461	0,84259077	25,4118991	0,14248349	0,33449278	-1,579953	-1,0555584	0,0447482	1,34922442	Unpaired t-te	0,044851508	permutation FDR (250 perm	1
3751	Vps39~Q8R5	Vps39	ko vs wt	ko	wt	27,6105127	1,01211919	26,8053227	0,71159228	1,74737593	0,80519002	0,45461904	0,34436341	0,462983	Unpaired t-te	0,301813595	permutation FDR (250 perm	1
3752	Vps41~Q5KU	Vps41	ko vs wt	ko	wt	27,1501677	0,81756005	26,7398917	0,35357599	1,32894	0,41027597	0,26742995	0,48158713	0,31732513	Unpaired t-te	0,536216381	permutation FDR (250 perm	1
3753	Vps45~P973	Vps45	ko vs wt	ko	wt	27,456831	0,30032316	27,6254482	0,38743822	0,889695	-0,1686172	-0,1273332	0,63378753	0,19805631	Unpaired t-te	0,777218365	permutation FDR (250 perm	1
3754	Vps4a~Q8VE	Vps4a	ko vs wt	ko	wt	28,3937235	0,21072349	27,8466727	0,17853714	1,46109584	0,54705082	0,46544601	0,02834014	1,54759801	Unpaired t-te	0,292175809	permutation FDR (250 perm	1
3755	Vps52~Q8C7	Vps52	ko vs wt	ko	wt	24,7042999	1,91312184	23,6762563	0,92241472	2,03925707	1,02804366	0,45035403	0,46212305	0,33524237	Unpaired t-te	0,308619895	permutation FDR (250 perm	1
3756	Vps53~Q8CC	Vps53	ko vs wt	ko	wt	26,8262514	0,45707365	25,934238	1,20809773	1,85576425	0,89201345	0,47094678	0,40864753	0,38865112	Unpaired t-te	0,315503994	permutation FDR (250 perm	1
3757	Vrk1~Q80X4	Vrk1	ko vs wt	ko	wt	24,7191245	0,52665421	24,6338792	0,27116087	1,06086808	0,08524527	0,06270476	0,82245945	0,08488551	Unpaired t-te	0,89110343	permutation FDR (250 perm	1
3758	Vrk2~Q8BN2	Vrk2	ko vs wt	ko	wt	24,335316	1,00893544	24,4477995	0,77128692	0,92499441	-0,1124834	-0,0625613	0,89350267	0,04890414	Unpaired t-te	0,891131515	permutation FDR (250 perm	1
3759	Vta1~Q9CR2	Vta1	ko vs wt	ko	wt	25,024502	1,29852052	26,223332	0,35519483	0,43562844	-1,19883	-0,6694928	0,21084138	0,67604415	Unpaired t-te	0,158528548	permutation FDR (250 perm	1
3760	Vtn~P29788	Vtn	ko vs wt	ko	wt	32,8761105	0,38014328	32,3584125	0,47543076	1,43166899	0,51769797	0,36939355	0,27328017	0,56339188	Unpaired t-te	0,404731691	permutation FDR (250 perm	1
3761	Vwa1~Q8R2	Vwa1	ko vs wt	ko	wt	31,1312132	0,38171996	31,1851381	0,18313708	0,96331199	-0,053925	-0,042947	0,84193044	0,07472379	Unpaired t-te	0,926017605	permutation FDR (250 perm	1
3762	Vwa5a~Q99I	Vwa5a	ko vs wt	ko	wt	31,2160018	0,3810779	31,6740406	0,17194956	0,72797515	-0,4580389	-0,3660281	0,13474082	0,87050082	Unpaired t-te	0,402228064	permutation FDR (250 perm	1
3763	Vwa8~Q8CC	Vwa8	ko vs wt	ko	wt	29,7588677	0,35965322	30,0106353	0,15231537	0,83986672	-0,2517677	-0,2040395	0,33772335	0,47143891	Unpaired t-te	0,637954306	permutation FDR (250 perm	1
3764	Vwf~E9QPU	Vwf	ko vs wt	ko	wt	33,6048314	0,53666348	33,6929309	0,20694344	0,94076121	-0,0880995	-0,0656157	0,80927216	0,0919054	Unpaired t-te	0,887186367	permutation FDR (250 perm	1
3765	Wapl~Q65Z4	Wapl	ko vs wt	ko	wt	24,6163356	0,44612821	24,4438697	0,47629685	1,12698317	0,17246597	0,12111406	0,70456722	0,15207756	Unpaired t-te	0,787460184	permutation FDR (250 perm	1
3766	Wars1~P329	Wars1	ko vs wt	ko	wt	28,8462533	0,56730113	28,7036307	0,27919198	1,10391001	0,14262257	0,10316819	0,72556874	0,13932144	Unpaired t-te	0,818160996	permutation FDR (250 perm	1
3767	Wasf2~Q8B	Wasf2	ko vs wt	ko	wt	25,6121207	1,25637358	28,1385589	0,73953909	0,17356666	-2,5264382	-1,3337666	0,03745968	1,42643591	Unpaired t-te	0,012845395	permutation FDR (250 perm	1
3768	Washc2~Q6	Washc2	ko vs wt	ko	wt	24,7702857	0,70210691	24,7188277	0,35739486	1,03631173	0,05145804	0,0348234	0,918646	0,03685181	Unpaired t-te	0,939524668	permutation FDR (250 perm	1
3769	Washc4~Q3	Washc4	ko vs wt	ko	wt	27,9499903	0,34089003	27,1919707	0,13081096	1,69116751	0,75801956	0,62262116	0,02360781	1,62694427	Unpaired t-te	0,178437795	permutation FDR (250 perm	1
3770	Washc5~Q8	Washc5	ko vs wt	ko	wt	27,3922814	0,3071668	27,1904685	0,27375117	1,15014269	0,20181286	0,15984838	0,47960293	0,31911818	Unpaired t-te	0,715051429	permutation FDR (250 perm	1
3771	Wasl~Q91Y	Wasl	ko vs wt	ko	wt	26,9685883	0,62770537	27,2439864	0,36363132	0,82622229	-0,2753981	-0,190672	0,56301686	0,2494786	Unpaired t-te	0,659557496	permutation FDR (250 perm	1
3772	Wbp2~P977	Wbp2	ko vs wt	ko	wt	25,7298856	1,58706279	27,2992375	0,06091974	0,33695973	-1,5693519	-0,8185205	0,18523563	0,73227548	Unpaired t-te	0,104174232	permutation FDR (250 perm	1
3773	Wdfy1~E9Q4	Wdfy1	ko vs wt	ko	wt	24,8737897	1,53967171	24,5349982	0,49578219	1,26469677	0,33879152	0,1732452	0,74142128	0,12993495	Unpaired t-te	0,693405343	permutation FDR (250 perm	1

3774	Wdfy3~Q6VI	Wdfy3	ko vs wt	ko	wt	27,0134391	0,4509889	26,1253414	0,4856378	1,85073425	0,88809775	0,6206345	0,10723873	0,96964835	Unpaired t-te	0,18043205	permutation FDR (250 perm	1
3775	Wdfy3~Q6VI	Wdfy3	ko vs wt	ko	wt	24,3500252	0,31189435	24,2508941	0,35370596	1,07112817	0,09913113	0,07577742	0,76416946	0,11681032	Unpaired t-te	0,871600655	permutation FDR (250 perm	1
3776	Wdfy4~E9PV	Wdfy4	ko vs wt	ko	wt	25,3077411	0,96168209	24,7599696	0,18639836	1,46182595	0,54777155	0,34875308	0,40146535	0,39635194	Unpaired t-te	0,432610126	permutation FDR (250 perm	1
3777	Wdr11~Q8K1	Wdr11	ko vs wt	ko	wt	27,5029605	0,67010272	27,1816717	0,40696246	1,24944619	0,32128877	0,2167691	0,53492756	0,27170502	Unpaired t-te	0,613503546	permutation FDR (250 perm	1
3778	Wdr13~Q91V	Wdr13	ko vs wt	ko	wt	24,5891447	0,80473424	23,7632453	0,37948951	1,7726398	0,82589941	0,53750704	0,18971642	0,72189508	Unpaired t-te	0,232812779	permutation FDR (250 perm	1
3779	Wdr1~O8834	Wdr1	ko vs wt	ko	wt	31,891662	0,29609567	31,9377954	0,30926593	0,96852861	-0,0461334	-0,0361102	0,87577086	0,05760951	Unpaired t-te	0,937355267	permutation FDR (250 perm	1
3780	Wdr26~Q8C6	Wdr26	ko vs wt	ko	wt	24,7753505	0,84156513	25,5123156	0,16204206	0,6000002	-0,7369651	-0,4915704	0,22765698	0,64271902	Unpaired t-te	0,279565957	permutation FDR (250 perm	1
3781	Wdr35~Q8BI	Wdr35	ko vs wt	ko	wt	24,3821894	0,73380749	24,0641094	0,90925925	1,24667031	0,31807999	0,17970836	0,70260575	0,1532883	Unpaired t-te	0,68371272	permutation FDR (250 perm	1
3782	Wdr37~Q8CE	Wdr37	ko vs wt	ko	wt	26,787779	0,20571596	26,7240247	0,15146878	1,04518204	0,06375424	0,05496384	0,7067696	0,15072214	Unpaired t-te	0,904213921	permutation FDR (250 perm	1
3783	Wdr41~Q3UI	Wdr41	ko vs wt	ko	wt	24,7255576	0,79541302	24,1550669	0,46756904	1,48502854	0,57049065	0,36432916	0,36026671	0,44337587	Unpaired t-te	0,401683865	permutation FDR (250 perm	1
3784	Wdr43~Q6Z(	Wdr43	ko vs wt	ko	wt	24,1181312	1,24559409	24,3642928	0,56910985	0,8431367	-0,2461615	-0,1349509	0,7784289	0,10878105	Unpaired t-te	0,759441913	permutation FDR (250 perm	1
3785	Wdr44~Q6N'	Wdr44	ko vs wt	ko	wt	26,6387882	0,82060491	26,5298663	0,34048369	1,07842205	0,1089219	0,07112383	0,8469106	0,07216243	Unpaired t-te	0,87851441	permutation FDR (250 perm	1
3786	Wdr45b~Q9(	Wdr45b	ko vs wt	ko	wt	26,8072411	0,05195136	26,7025314	0,14568292	1,07527803	0,10470974	0,09456387	0,41885095	0,37794049	Unpaired t-te	0,843020319	permutation FDR (250 perm	1
3787	Wdr45~A2AE	Wdr45	ko vs wt	ko	wt	23,9704128	0,67980064	23,2702484	0,4442844	1,6246899	0,70016438	0,46592831	0,22248152	0,65270605	Unpaired t-te	0,290194366	permutation FDR (250 perm	1
3788	Wdr47~Q8C(	Wdr47	ko vs wt	ko	wt	25,4092152	0,52788159	25,4096301	0,07453392	0,99971245	-0,0004149	-0,0003169	0,99900948	0,00043039	Unpaired t-te	0,999192518	permutation FDR (250 perm	1
3789	Wdr48~Q8BI	Wdr48	ko vs wt	ko	wt	25,5777934	1,36048425	26,4051063	0,31829217	0,56357793	-0,827313	-0,4552969	0,37646449	0,42427598	Unpaired t-te	0,313401531	permutation FDR (250 perm	1
3790	Wdr5~P6196	Wdr5	ko vs wt	ko	wt	27,8339658	0,59489338	25,5653077	1,82071916	4,81874705	2,26865807	0,97264208	0,21484342	0,66787793	Unpaired t-te	0,072825013	permutation FDR (250 perm	1
3791	Wdr6~Q99M	Wdr6	ko vs wt	ko	wt	26,0211423	0,31039349	26,490911	0,16685458	0,72208036	-0,4697687	-0,3867818	0,08230635	1,08456664	Unpaired t-te	0,376669344	permutation FDR (250 perm	1
3792	Wdr7~Q920I	Wdr7	ko vs wt	ko	wt	26,5433389	0,48524094	26,7078375	0,28106113	0,8922386	-0,1644985	-0,1224414	0,65255908	0,18538016	Unpaired t-te	0,781938479	permutation FDR (250 perm	1
3793	Wdr82~Q8BI	Wdr82	ko vs wt	ko	wt	28,0139274	0,31328182	27,6309855	0,18223978	1,30399821	0,38294189	0,3133518	0,14622937	0,8349654	Unpaired t-te	0,466820999	permutation FDR (250 perm	1
3794	Wdr91~Q7TI	Wdr91	ko vs wt	ko	wt	25,3673617	0,96529298	25,7991022	0,03493892	0,74136681	-0,4317406	-0,277137	0,4951673	0,30524804	Unpaired t-te	0,532760331	permutation FDR (250 perm	1
3795	Wfs1~P5669	Wfs1	ko vs wt	ko	wt	25,4637876	1,22125042	26,7402295	0,10651044	0,41281238	-1,2764418	-0,7468502	0,16761704	0,77568184	Unpaired t-te	0,130499784	permutation FDR (250 perm	1
3796	Wif1~H3BKC	Wif1	ko vs wt	ko	wt	23,8563617	0,83196606	24,2974307	0,66758482	0,73658865	-0,4410689	-0,263566	0,54240291	0,26567799	Unpaired t-te	0,540404398	permutation FDR (250 perm	1
3797	Wls~Q6DID7	Wls	ko vs wt	ko	wt	24,4964541	0,93120996	23,8857629	0,47473781	1,52699059	0,61069118	0,37377922	0,3820313	0,41790106	Unpaired t-te	0,392548708	permutation FDR (250 perm	1
3798	Wnk1~P8374	Wnk1	ko vs wt	ko	wt	26,592487	0,37293232	24,9274603	1,02242073	3,1711953	1,66502673	0,94908912	0,13843187	0,85876391	Unpaired t-te	0,076773744	permutation FDR (250 perm	1
3799	Wnt2b~O702	Wnt2b	ko vs wt	ko	wt	24,5404976	1,07518204	24,1326234	1,13028742	1,32672943	0,40787418	0,20272228	0,70687971	0,15065448	Unpaired t-te	0,641546838	permutation FDR (250 perm	1
3800	Wrnip1~Q91	Wrnip1	ko vs wt	ko	wt	25,0510243	1,18835274	26,3008761	0,08938201	0,42049139	-1,2498518	-0,7399949	0,16578593	0,78045233	Unpaired t-te	0,133841788	permutation FDR (250 perm	1
3801	Wwp1~Q8B2	Wwp1	ko vs wt	ko	wt	24,9774896	1,00915811	24,6745616	1,1295472	1,23364558	0,30292798	0,15232937	0,77476614	0,11082937	Unpaired t-te	0,730669316	permutation FDR (250 perm	1
3802	Xab2~Q9DCD	Xab2	ko vs wt	ko	wt	26,4587234	0,63848988	25,0745162	1,32494784	2,61028463	1,38420713	0,68976062	0,27474812	0,56106526	Unpaired t-te	0,161154002	permutation FDR (250 perm	1
3803	Xdh~Q00519	Xdh	ko vs wt	ko	wt	26,1038571	1,55451383	25,1102101	0,5729166	1,99121222	0,99364699	0,50065515	0,36881745	0,43318854	Unpaired t-te	0,26574964	permutation FDR (250 perm	1
3804	Xirp1~E9QQ9	Xirp1	ko vs wt	ko	wt	29,0463244	0,48687199	29,7627085	0,77768755	0,60862093	-0,7163841	-0,4428721	0,32891422	0,48291735	Unpaired t-te	0,330629553	permutation FDR (250 perm	1
3805	Xpnpep1~Q3	Xpnpep1	ko vs wt	ko	wt	25,6081478	1,57666729	24,2283976	0,70680207	2,60223319	1,37975025	0,67685721	0,24771905	0,6060406	Unpaired t-te	0,149060581	permutation FDR (250 perm	1
3806	Xpnpep3~B7	Xpnpep3	ko vs wt	ko	wt	25,1287645	0,38219814	25,5475933	0,0720691	0,74803165	-0,4188288	-0,3414912	0,15273302	0,81606707	Unpaired t-te	0,441448364	permutation FDR (250 perm	1
3807	Xpo1~Q6P5F	Xpo1	ko vs wt	ko	wt	28,9959728	0,36600042	29,009866	0,10401844	0,99041618	-0,0138932	-0,011353	0,95370416	0,02058632	Unpaired t-te	0,980966022	permutation FDR (250 perm	1
3808	Xpo7~Q9EPK	Xpo7	ko vs wt	ko	wt	24,0870077	0,44214546	26,6034465	0,14890213	0,17477385	-2,5164388	-1,9719232	0,00086626	3,06235225	Unpaired t-te	0,003710438	permutation FDR (250 perm	1
3809	Xrcc5~P2764	Xrcc5	ko vs wt	ko	wt	25,2944563	0,29212821	25,4743607	0,39817183	0,88276147	-0,1799044	-0,1354496	0,61754544	0,20933108	Unpaired t-te	0,763470191	permutation FDR (250 perm	1
3810	Xrcc6~P2347	Xrcc6	ko vs wt	ko	wt	24,3313243	0,51155635	24,2292212	0,2160742	1,07333698	0,10210309	0,07662367	0,77293908	0,11185473	Unpaired t-te	0,868588267	permutation FDR (250 perm	1
3811	Xrn1~A0A08	Xrn1	ko vs wt	ko	wt	26,9405365	0,1717215	26,800444	0,20236551	1,10197573	0,14009245	0,11932061	0,46832242	0,32945505	Unpaired t-te	0,791602457	permutation FDR (250 perm	1
3812	Xrn2~Q9DBR	Xrn2	ko vs wt	ko	wt	26,2742299	0,22542722	26,1202499	0,04888565	1,1126347	0,15398001	0,13570554	0,32667174	0,48588844	Unpaired t-te	0,762885264	permutation FDR (250 perm	1
3813	Yap1~P4693	Yap1	ko vs wt	ko	wt	25,0408462	1,13365836	24,0554347	0,70448173	1,97987799	0,98541153	0,54068593	0,28482128	0,54542757	Unpaired t-te	0,228324001	permutation FDR (250 perm	1
3814	Yars~A2A7S	Yars	ko vs wt	ko	wt	30,6098423	0,15957448	30,3223244	0,13058099	1,22053855	0,28751786	0,25434232	0,08097316	1,0916589	Unpaired t-te	0,554089118	permutation FDR (250 perm	1
3815	Yif1a~Q91XB	Yif1a	ko vs wt	ko	wt	24,2981522	0,99462778	25,5250774	0,8913567	0,42722702	-1,2269252	-0,6622525	0,21469911	0,66816975	Unpaired t-te	0,155214754	permutation FDR (250 perm	1
3816	Yif1b~Q9CX3	Yif1b	ko vs wt	ko	wt	24,9657212	0,88584587	25,3468317	0,16109132	0,7678463	-0,3811105	-0,2500767	0,51531317	0,28792876	Unpaired t-te	0,569436145	permutation FDR (250 perm	1
3817	Ykt6~Q9CQW	Ykt6	ko vs wt	ko	wt	25,8307429	1,35234691	26,619576	0,08369417	0,57881206	-0,7888331	-0,4424147	0,3871803	0,41208674	Unpaired t-te	0,331146685	permutation FDR (250 perm	1
3818	Yme1l1~O88	Yme1l1	ko vs wt	ko	wt	24,4092626	1,56990381	25,2864197	1,26223674	0,54443924	-0,877157	-0,3860613	0,52220242	0,28216112	Unpaired t-te	0,377493976	permutation FDR (250 perm	1
3819	Ythdf1~P593	Ythdf1	ko vs wt	ko	wt	27,0626426	0,11160468	26,9710507	0,13992796	1,06554534	0,09159199	0,08191933	0,48544017	0,31386429	Unpaired t-te	0,860521739	permutation FDR (250 perm	1
3820	Ythdf3~Q8BY	Ythdf3	ko vs wt	ko	wt	26,3597218	0,20584928	26,3812672	0,37781871	0,98517682	-0,0215454	-0,0166708	0,94621076	0,02401212	Unpaired t-te	0,971441592	permutation FDR (250 perm	1
3821	Ywhab~Q9C(	Ywhab	ko vs wt	ko	wt	29,2389565	0,33833926	30,0319028	0,62150087	0,57716419	-0,7929463	-0,5354396	0,2043373	0,68965236	Unpaired t-te	0,25244153	permutation FDR (250 perm	1
3822	Ywhae~P622	Ywhae	ko vs wt	ko	wt	30,9664699	0,14200438	31,2261893	0,18134773	0,83525038	-0,2597194	-0,2254114	0,17164447	0,76537019	Unpaired t-te	0,606118483	permutation FDR (250 perm	1
3823	Ywhag~P619	Ywhag	ko vs wt	ko	wt	30,1302833	0,18421622	30,2446311	0,25190873	0,92379981	-0,1143478	-0,0947009	0,61576357	0,21058601	Unpaired t-te	0,838170611	permutation FDR (250 perm	1
3824	Ywhah~P685	Ywhah	ko vs wt	ko	wt	29,8246499	0,43501669	29,6651238	0,21747847	1,11692019	0,1595261	0,12323408	0,61278846	0,21268942	Unpaired t-te	0,780846756	permutation FDR (250 perm	1
3825	Ywhaq~P682	Ywhaq	ko vs wt	ko	wt	30,7415076	0,45891741	31,1485649	0,24939443	0,75416009	-0,4070573	-0,3087797	0,25896026	0,58676688	Unpaired t-te	0,473590422	permutation FDR (250 perm	1
3826	Ywhaz~P631	Ywhaz	ko vs wt	ko	wt	31,4222958	0,12825039	31,9935283	0,29660657	0,67304155	-0,5712325	-0,4672966	0,09908998	1,00397027	Unpaired t-te	0,316301284	permutation FDR (250 perm	1
3827	Zbtb16~Q3U	Zbtb16	ko vs wt	ko	wt	23,9328785	0,51477624	26,1134944	0,46746369	0,22058156	-2,1806159	-1,5095845	0,00571042	2,243332	Unpaired t-te	0,008362157	permutation FDR (250 perm	1
3828	Zbtb20~Q8K(	Zbtb20	ko vs wt	ko	wt	27,8891138	0,48770997	27,8803003	0,38685238	1,00612774	0,00881348	0,00632891	0,98298247	0,00745423	Unpaired t-te	0,988389685	permutation FDR (250 perm	1
3829	Zc2hc1a~Q8I	Zc2hc1a	ko vs wt	ko	wt	26,7428886	0,60754075	27,0847077	0,52345317	0,78904579	-0,3418191	-0,2263795	0,53426255	0,27224527	Unpaired t-te	0,59793698	permutation FDR (250 perm	1
3830	Zc3h11a~Q6I	Zc3h11a	ko vs wt	ko	wt	24,4462714	0,78916692	24,3356645	0,52560298	1,07968237	0,11060695	0,06965248	0,85818473	0,06641922	Unpaired t-te	0,880445773	permutation FDR (250 perm	1
3831	Zc3h15~Q3TI	Zc3h15	ko vs wt	ko	wt	25,5217917	1,02690163	25,9582522	0,3939255	0,73894528	-0,4364606	-0,2637136	0,54031122	0,26735601	Unpaired t-te	0,543334349	permutation FDR (250 perm	1
3832	Zc3h7b~F8VI	Zc3h7b	ko vs wt	ko	wt	25,3489286	0,82663267	24,7126005	0,2536286	1,55436797	0,63632808	0,42145377	0,28348348	0,54747225	Unpaired t-te	0,344746848	permutation FDR (250 perm	1
3833	Zc3hav1~Q3I	Zc3hav1	ko vs wt	ko	wt	26,2267984	1,50981274	26,7928272	0,02937901	0,67547354	-0,5660288	-0,3023758	0,56245358	0,24991332	Unpaired t-te	0,496954871	permutation FDR (250 perm	1
3834	Zdhhc17~Q8I	Zdhhc17	ko vs wt	ko	wt	24,8306862	0,69456927	24,7051386	0,28494	1,0909217	0,12554756	0,08665739	0,79262792	0,10093063	Unpaired t-te	0,850356104	permutation FDR (250 perm	1
3835	Zfp820~E9Q	Zfp820	ko vs wt	ko	wt	24,0533042	0,88122258	24,9354786	0,82626613	0,5425491	-0,8821744	-0,4970748	0,3121596	0,5056233	Unpaired t-te	0,266195683	permutation FDR (250 perm	1
3836	Zfr~O88532	Zfr	ko vs wt	ko	wt	23,4865699	0,92466136	25,2257348	1,06244188	0,29954302	-1,7391649	-0,9050495	0,13455964	0,87108519	Unpaired t-te	0,070731807	permutation FDR (250 perm	1
3837	Zfyve1~Q81(	Zfyve1	ko vs wt	ko	wt	24,5678528	0,52171552	23,9308846	1,07873964	1,55505779	0,6369682	0,34996276	0,5011447	0,30003686	Unpaired t-te	0,439838254	permutation FDR (250 perm	1

3838	Zmiz2~Q8CII	Zmiz2	ko vs wt	ko	wt	24,417392	0,27264739	24,6286335	0,50467399	0,86379357	-0,2112415	-0,1519686	0,62841342	0,20175455	Unpaired t-te	0,737496745	permutation FDR (250 perm	1
3839	Zmpste24~Q	Zmpste24	ko vs wt	ko	wt	25,08412	1,61147425	26,8225111	0,28767357	0,29970372	-1,7383911	-0,8904038	0,15746777	0,80280831	Unpaired t-te	0,07951731	permutation FDR (250 perm	1
3840	Znf207~Q9JI	Znf207	ko vs wt	ko	wt	25,1173513	0,85383121	24,2100061	0,53961541	1,87559104	0,90734529	0,55892381	0,20544057	0,68731379	Unpaired t-te	0,21440514	permutation FDR (250 perm	1
3841	Znf326~O88	Znf326	ko vs wt	ko	wt	26,9885017	0,27277883	27,0983179	0,49908564	0,92670612	-0,1098162	-0,0792066	0,79597689	0,09909954	Unpaired t-te	0,868259754	permutation FDR (250 perm	1
3842	Znf512~Q69	Znf512	ko vs wt	ko	wt	26,9957016	0,51544732	27,9107585	1,29083541	0,53032295	-0,9150569	-0,4668546	0,42544747	0,37115406	Unpaired t-te	0,318178163	permutation FDR (250 perm	1
3843	Znf638~Q61	Znf638	ko vs wt	ko	wt	28,1647098	0,33420011	28,2522903	0,41197073	0,94109967	-0,0875806	-0,0649027	0,81548646	0,08858324	Unpaired t-te	0,889198919	permutation FDR (250 perm	1
3844	Znfx1~Q8R1	Znfx1	ko vs wt	ko	wt	27,2548817	0,08542581	27,5019967	0,44347102	0,84257967	-0,247115	-0,1875726	0,51440779	0,28869246	Unpaired t-te	0,685876657	permutation FDR (250 perm	1
3845	Zswim8~Q3	Zswim8	ko vs wt	ko	wt	26,4686505	0,07689307	25,2433494	1,29659477	2,33804242	1,22530111	0,63887472	0,31305943	0,50437321	Unpaired t-te	0,201534331	permutation FDR (250 perm	1
3846	Zw10~O546	Zw10	ko vs wt	ko	wt	26,6888209	0,84326128	26,7445447	0,67605828	0,96211159	-0,0557239	-0,0331233	0,9381836	0,02771216	Unpaired t-te	0,942440911	permutation FDR (250 perm	1
3847	Zyx~Q62523	Zyx	ko vs wt	ko	wt	32,0487865	0,62598505	32,3821422	0,05774055	0,79368821	-0,3333557	-0,2444474	0,42544619	0,37115536	Unpaired t-te	0,579888623	permutation FDR (250 perm	1
3848	mt-Nd2~P03	mt-Nd2	ko vs wt	ko	wt	24,6754488	0,55395183	24,457159	1,0463522	1,16335367	0,21828975	0,12086595	0,80555017	0,0939074	Unpaired t-te	0,794180296	permutation FDR (250 perm	1

Supplemental Figures

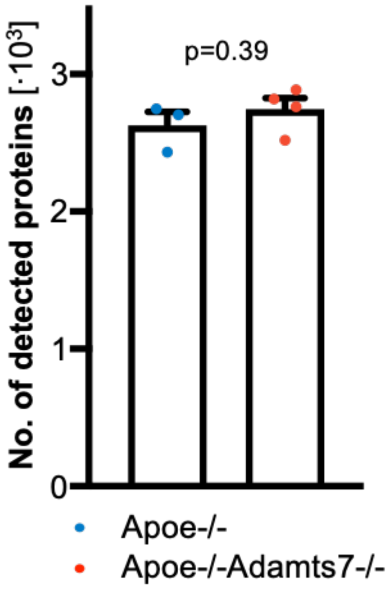

Supplemental Figure S1: Number of detected proteins by mass spectrometry in Apoe-/- (n=3) and Apoe-/-Adamts7-/- mice (n=4). *Student's t-test. Symbols indicate independent animals. Data are mean and s.e.m.*

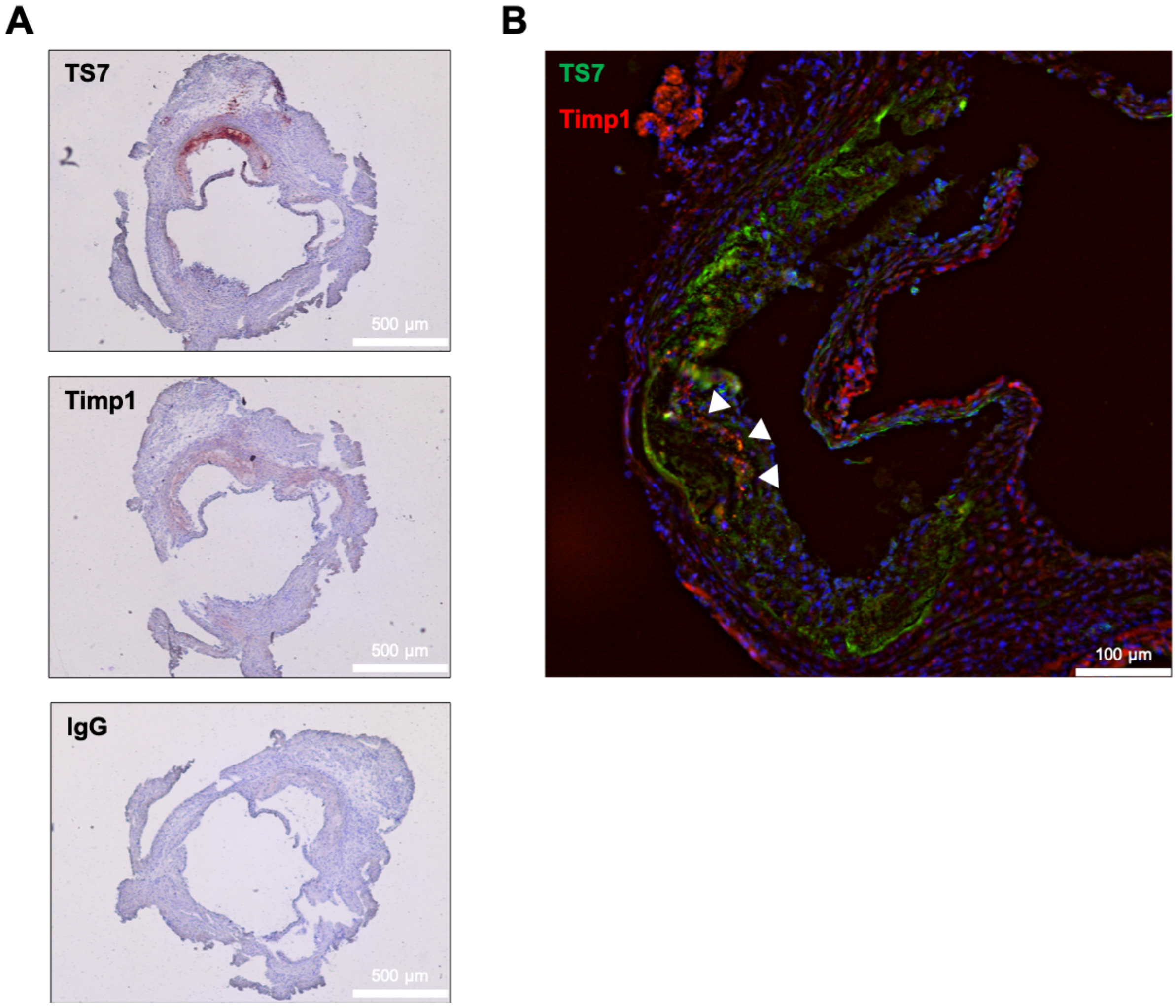

Supplemental Figure S2: Adamts-7 and Timp-1 in atherosclerotic plaques. **A**. Immunohistochemistry of aortic root sections indicate abundance of Adamts-7 (TS7) and Timp-1 (Timp1) in atherosclerotic plaques. **B**. Immunofluorescence staining of Adamts-7 and Timp-1 in the aortic root. White arrows indicate co-localized Adamts-7 and Timp-1.

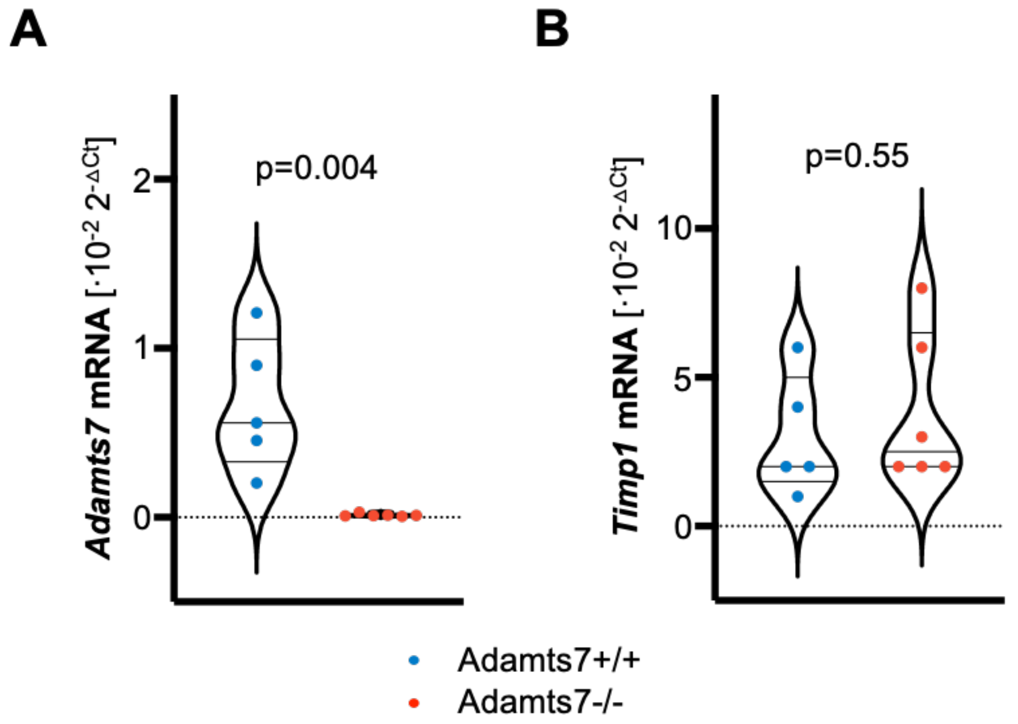

Supplemental Figure S3: *Adamts7* (A) and *Timp1* (B) expression in aortic tissue of Adamts7-/- (n=5) and Adamts7+/+ mice (n=6). *Mann-Whitney test. Symbols indicate independent animals.*

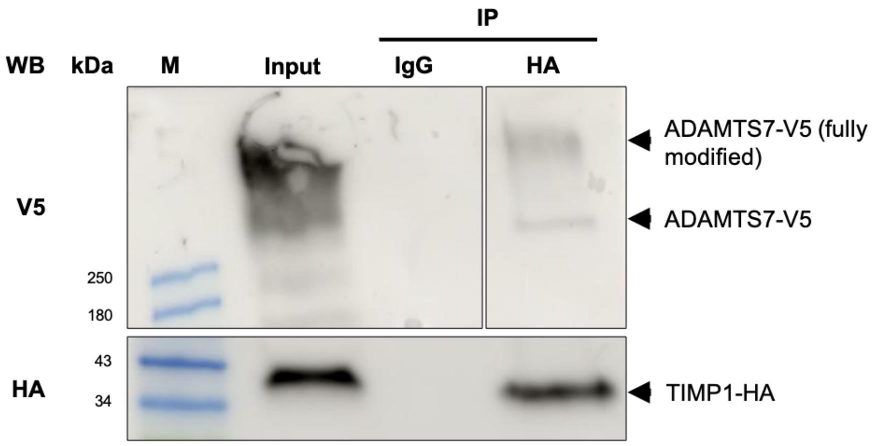

Supplemental Figure S4: Co-IP of ADAMTS-7 and TIMP1-HA from supernatant. After precipitation of TIMP1-HA, ADAMTS7-V5 was detectable. Different ADAMTS-7 bands might represent different states of post-translational modification. The V5-Blot is cropped because of different exposure times between input and HA-IP lanes. Supernatant was concentrated using a vacuum concentrator and 45% of total supernatant were used for IP and 10% were used for input. *M*, molecular weight marker.

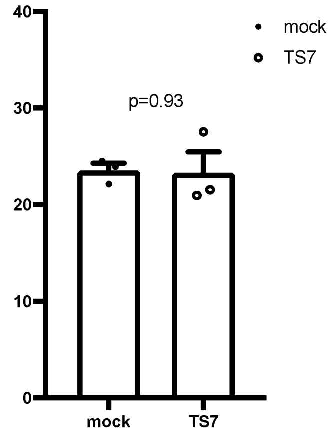

Supplemental Figure S5: Influence of ADAMTS-7 alone on rMMP-9 activity. Recombinant MMP-9 activity was not altered in presence of ADAMTS-7 in comparison to the absence of ADAMTS-7 (mock transfection) The y-axis denotes rMMP-9 activity (%). *Student's t-test. Symbols indicate independent experiments (n=3). Data are mean and s.e.m.*

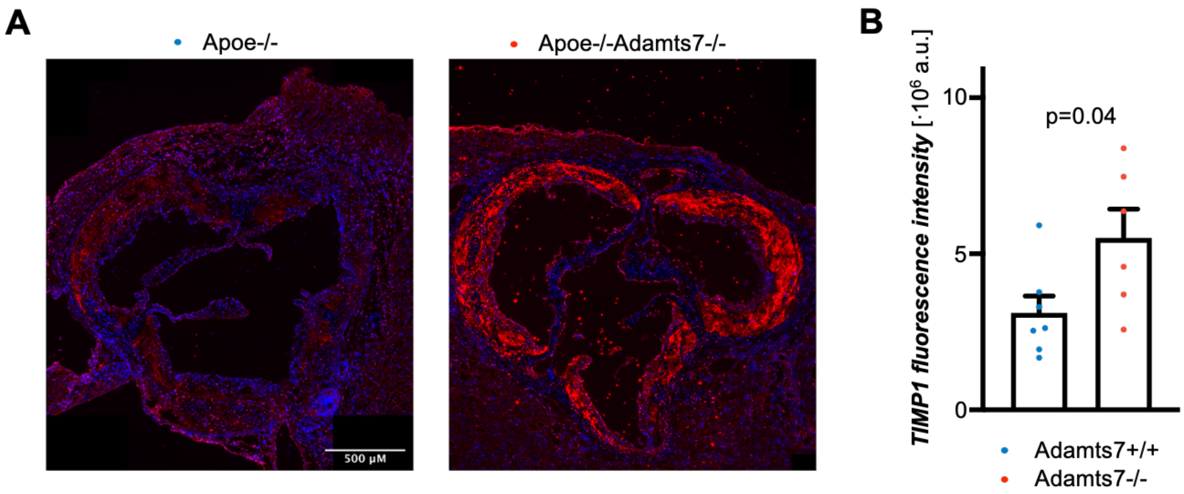

Supplemental Figure S6: TIMP-1 immunofluorescence in Apoe-/- (n=7) and Apoe-/-Adamts7-/- mice (n=6) after 12 weeks of Western diet. **A**. Representative immunofluorescence scan stacks. **B**. Quantification. *Student's t- test. Symbols indicate independent animals.*

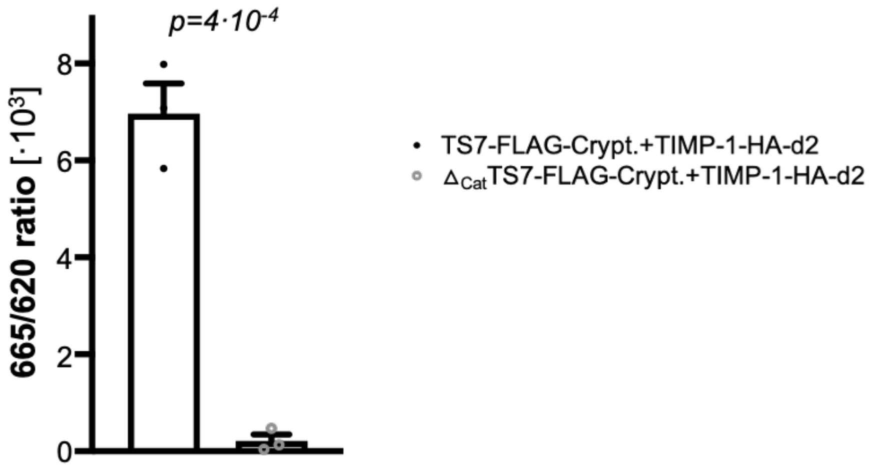

Supplemental Figure S7: Confirmation of TIMP-1 binding to the catalytic domain and validation of FRET assay specificity for TIMP-1 binding to the catalytic domain of ADAMTS-7. *Student's t-test. Symbols indicate independent experiments (n=3 each).* $_{\Delta cat}$*TS7-FLAG-Crypt.*, ADAMTS-7 construct lacking the catalytic domain.
